# Enantioselective Bifunctional Ammonium Salt‐Catalyzed Syntheses of 3‐CF_3_S‐, 3‐RS‐, and 3‐F‐Substituted Isoindolinones

**DOI:** 10.1002/adsc.202100029

**Published:** 2021-02-17

**Authors:** Andreas Eitzinger, Jan Otevrel, Victoria Haider, Antonio Macchia, Antonio Massa, Kirill Faust, Bernhard Spingler, Albrecht Berkessel, Mario Waser

**Affiliations:** ^1^ Institute of Organic Chemistry Johannes Kepler University Linz Altenbergerstr. 69 4040 Linz Austria; ^2^ Department of Chemical Drugs Faculty of Pharmacy Masaryk University Palackeho 1946/1 612 00 Brno Czechia; ^3^ Dipartimento di Chimica e Biologia Università di Salerno Via Giovanni Paolo II, 132 84084 Fisciano SA Italy; ^4^ Institute of Catalysis Johannes Kepler University Linz Altenbergerstr. 69 4040 Linz Austria; ^5^ Department of Chemistry University of Zurich Winterthurerstrasse 190 8057 Zurich Switzerland; ^6^ Department of Chemistry Cologne University Greinstrasse 4 50939 Cologne Germany

**Keywords:** Organocatalysis, Asymmetric phase-transfer catalysis, Bifunctional catalysis, Heterofunctionalization, Organofluorine chemistry

## Abstract

We herein report the ammonium salt‐catalyzed synthesis of chiral 3,3‐disubstituted isoindolinones bearing a heteroatom functionality in the 3‐position. A broad variety of differently substituted CF_3_S‐ and RS‐derivatives were obtained with often high enantioselectivities when using Maruoka's bifunctional chiral ammonium salt catalyst. In addition, a first proof‐of‐concept for the racemic synthesis of the analogous F‐containing products was obtained as well, giving access to one of the rare examples of a fairly stable α‐F‐α‐amino acid derivative.

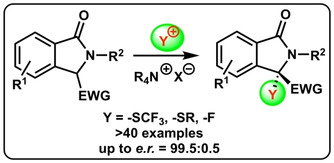

## Introduction

**Scheme 1:**
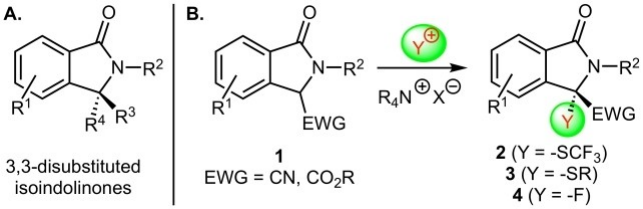
Targeted asymmetric synthesis of 3‐heterofunctionalized 3,3‐disubstituted isoindolinones.

Isoindolinones have emerged as a promising class of chiral small molecule heterocycles with interesting biological properties over the course of the last years.[^1^, ^2^] A variety of different strategies to access these versatile targets in an enantioselective manner have been introduced^[2]^ and especially the asymmetric synthesis of 3,3‐disubstituted derivatives (Scheme 1A) became a contemporary field of interest.[^3^, ^4^, ^5^, ^6^, ^7^, ^8^, ^9^] Noteworthy, whereas several conceptually complementary methods to access enantioenriched 3,3‐disubstituted isoindolinones bearing an all‐carbon stereogenic center have been reported,[^3^, ^4^] the enantioselective (catalytic) synthesis of 3,3‐disubstituted isoindolinones with a heteroatom‐functionality in the 3‐position has so far received less attention.[^5^, ^6^, ^7^]

One recently introduced strategy for the synthesis of 3,3‐disubstituted isoindolinones relies on the asymmetric α‐functionalization of EWG‐containing isoindolinones **1**.[^8^, ^9^] Until now, this has mainly been used for asymmetric C−C‐bond forming reactions,^[8]^ whereas, to the best of our knowledge, so far no enantioselective α‐heterofunctionalizations of compounds **1** with electrophilic heteroatom‐transfer reagents have been reported. We have a strong research interest in asymmetric ammonium salt ion pairing catalysis,[^10^, ^11^] with a special focus on asymmetric α‐heterofunctionalizations of different (pro)‐nucleophiles.^[12]^ Considering the general potential of chiral ammonium salt catalysis for stereoselective α‐heterofunctionalization reactions,^[13]^ as well as for the asymmetric control of pronucleophiles **1**,^[8b]^ we now became interested in the development of new strategies for the synthesis of 3‐heterofunctionalized isoindolinones **2** (i. e. CF_3_S‐, RS‐, F‐substituted ones, Scheme 1B).[^14^, ^15^, ^16^, ^17^, ^18^]

## Results and Discussion

**Figure 1:**
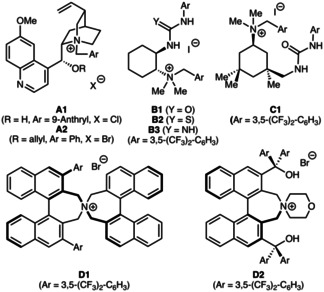
Asymmetric ammonium salt catalysts used herein.

Based on the widespread interest in asymmetric trifluoromethylthiolation reactions[^14^, ^15^] and our own recent experience in this field,^[12a]^ we first focused on the asymmetric α‐trifluoromethylthiolation of 3‐cyano p‐methoxybenzyl‐(PMB)‐protected isoindolinone **1 a** (Table 1) using different established, as well as newly designed, chiral ammonium salt catalysts **A**–**D** (Figure 1).

**Table 1:** Identification of the best‐suited catalyst and conditions for the synthesis of compounds **2 a**.^[a]^

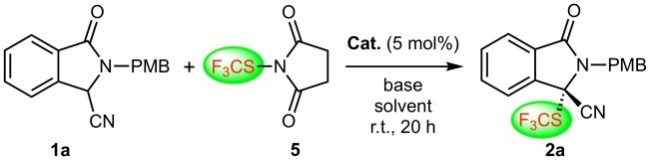					
Entry	Cat.	Base (equiv.)	Solv.	Yield^[b]^ [%]	*e.r*.^[c]^
1	**A1**	K_2_CO_3_ (1)	MTBE	70	50:50
2	**A2**	K_2_CO_3_ (1)	MTBE	74	50:50
3	**D1**	K_2_CO_3_ (1)	MTBE	82	52:48
4	**B1**	K_2_CO_3_ (1)	MTBE	26	39:61
5	**B2**	K_2_CO_3_ (1)	MTBE	25	43:57
6	**C1**	K_2_CO_3_ (1)	MTBE	86	40:60
7	**B3**	K_2_CO_3_ (1)	MTBE	80	30:70
8	**D2**	K_2_CO_3_ (1)	MTBE	90	97:3 (86:14)^[d]^
9	**D2**	K_2_CO_3_ (1)	toluene	50^[e]^	76:24
10	**D2**	K_2_CO_3_ (1)	CH_2_Cl_2_	44^[e]^	72:28
11	**D2**	K_2_CO_3_ (1)	Et_2_O	82	95:5
12	**D2**	K_2_CO_3_ (1)	THF	75	84:16
13	**D2**	K_2_CO_3_ (10% aq.) (1)	MTBE	77	81:19
14	**D2**	K_2_CO_3_ (0.2)	MTBE	95	97:3
15	**D2**	Cs_2_CO_3_ (0.2)	MTBE	92	96:4
16	**D2**	K_2_HPO_4_ (0.2)	MTBE	95	97:3
17^[f]^	**D2**	K_2_CO_3_ (0.2)	MTBE	95	98:2

^[a]^ Unless otherwise stated all reactions were carried out using 0.1 mmol **1 a**, 0.12 mmol **5** (1.2 equiv.) and 5 mol% of the catalyst with the indicated base and solvent at room temperature (20 h reaction time, 0.05 M with respect to **1 a**); PMB=p‐methoxy‐benzyl. ^[b]^ Isolated yields. ^[c]^ Determined by HPLC using a chiral stationary phase. The absolute configuration of the major enantiomer was assigned in analogy to derivative **2 e** ^[26]^ which was analyzed by single crystal X‐ray diffraction. ^[d]^ Using 1 mol% catalyst. ^[e]^ Incomplete conversion. ^[f]^ Reaction carried out at −20 °C.

Table 1 gives an illustrative overview about the most significant results obtained in a broad screening of different catalysts and conditions for the enantioselective synthesis of the CF_3_S‐containing isoindolinone **2 a**. As CF_3_S‐transfer agent we used the well‐documented succinimide‐based reagent **5** ^[14]^ but we also tested the analogous phthalimide‐based derivative which performed literally identical herein. First experiments with the classical well‐established Cinchona alkaloid‐based catalysts **A1** and **A2** ^[10]^ and with the commercially available Maruoka salt **D1** ^[19]^ were rather disappointing, providing **2 a** in a racemic manner only (entries 1–3). First measurable levels of enantioselectivities (*e.r*.∼60:40) could be obtained by using our bifunctional urea‐ and thiourea‐containing ammonium salts **B1** and **B2**,^[20]^ albeit with low yields only and accompanied by the formation of significant amounts of uncharacterized side‐products (entries 4 and 5).

Based on these results, that indicated the potential of bifunctional ammonium salt catalysts for this application, we next wanted to use the synthesis of **2 a** as a test reaction for the development of novel alternative bifunctional ammonium salts. Here we followed two different strategies. First, we wanted to test an alternative chiral diamine backbone with a different linker length between the two functional groups. One easily accessible chiral diamine that has so far not been explored for asymmetric ammonium salt catalysis is isophorone diamine.^[21]^ The synthesis of the corresponding chiral ammonium salt **C1** was possible in an analogous manner as reported previously for compounds **B**.[^20^, ^22^] In contrast to catalysts **B1** and **B2** the new derivative **C1** gave product **2 a** in a good isolated yield of 86%, but unfortunately the enantioselectivity could not be improved (entry 6) and no further optimization was possible.

Alternatively, we wanted to expand the diversity and applicability of our cyclohexanediamine‐system **B** by introducing guanidines as potential H‐bonding motives. Surprisingly, despite of all the value of chiral guanidines for asymmetric catalysis,[^23^, ^24^] such a chiral ammonium salt‐guanidine hybrid system has, to the best of our knowledge, so far not been reported. We found that the synthesis of the unprecedented bifunctional ammonium salt **B3** was indeed possible^[22]^ and that this catalyst also allowed for a better catalytic performance than **B1** and **B2** (entry 7). Unfortunately, we were not able to improve this result further and other ammonium salt‐guanidine derivatives could so far not be accessed. Thus, despite this interesting proof‐of‐concept for this novel class of catalysts, we stopped testing our own hybrid systems and finally tested Maruoka's bifunctional ammonium salt **D2** ^[25]^ as well. Literally the first experiment with 5 mol% of this nowadays commercially available catalyst gave product **2 a** in 90% isolated yield with an excellent enantiomeric ratio of 97:3 (unfortunately the *e.r*. dropped to 86:14 when using 1 mol% **D2**; entry 8).

Based on these promising initial results, we screened a variety of different bases and temperatures (entries 8–16) and realized that actually our initial conditions using solid K_2_CO_3_ in MTBE were almost the best. The only minor improvement that could be achieved in this optimization was to carry out the reaction with catalytic amounts of external base only (see entries 14–16), which can be explained by the fact that the in situ formed succinimide acts as a base itself (which is in analogy to our previous observations in the trifluoromethylthiolation of masked β‐amino acids^[12a]^). In addition, a further slight increase in enantioselectivity was observed when carrying out the reaction at −20 °C, thus allowing for the asymmetric synthesis of **2 a** in high yields and with excellent enantioselectivities under biphasic conditions (entry 17).

**Scheme 2:**
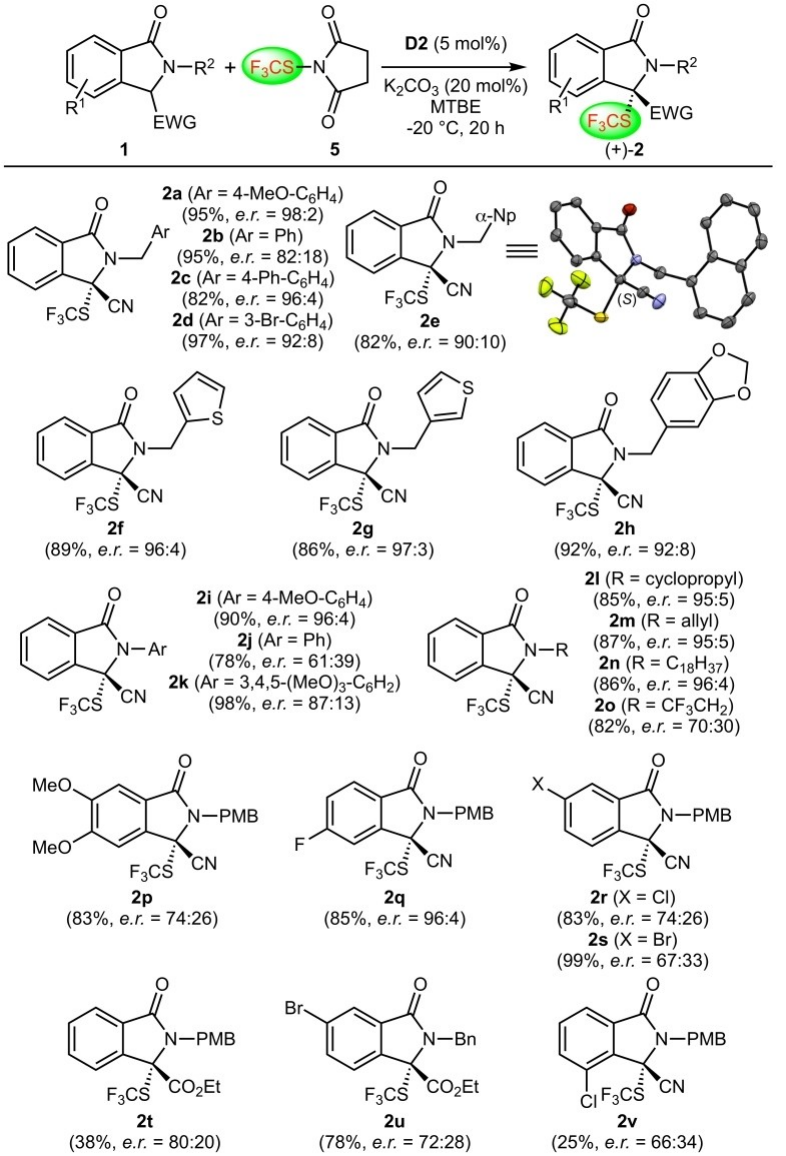
Application scope of the asymmetric α‐trifluoromethylthiolation of various isoindolinones **1** (the (+)‐enantiomers of products **2** were favored in all cases); α‐Np=1‐naphthyl.

With these optimized conditions at hand, we next investigated the generality of this reaction (Scheme 2).

First, we carried out a systematic variation of the N‐protecting group by introducing different benzylic groups (giving products **2 a**–**2 h**), several aryl substituents (products **2 i**–**2 k**) and various alkyl groups (products **2 l**–**2 o**). Surprisingly, the obtained enantioselectivities varied significantly. For example, the presence of a simple N‐benzyl‐group (product **2 b**) led to clearly inferior results (*e.r*.=80:20) compared to the other N‐benzyl derivatives **2 a**, **2 c**–**2 h** (which varied between *e.r*.=90:10–98:2). X‐ray diffraction analysis of single crystals of target **2 e** allowed us to assign the (*S*)‐configuration for this product^[26]^ and the other derivatives were then assigned in analogy. When testing different N‐aryl‐substituents (products **2 i**–**2 k**) the outcome was also very much dependent on the nature of this group, which was especially pronounced when comparing the results obtained for the N‐PMP‐containing **2 i** (*e.r*.=96:4) and the N‐phenyl‐substituted **2 j** (*e.r*.=61:39). On the other hand, several N‐alkyl groups were well‐tolerated (**2 l**–**2 n**; *e.r*.>95:5) and only the trifluoroethyl‐substituted **2 o** was obtained with a significantly reduced selectivity (*e.r*.=70:30). It has to be pointed out that this strong difference in performance really came as a surprise, illustrating that the N‐substituent has a pronounced (steric and/or electronic) impact on the catalyst‐substrate interaction in this reaction.

Interestingly, substitutions at the isoindolinone‐backbone also strongly influenced the outcome (**2 p**–**2 s**). While the presence of fluorine in the 5‐position was well‐tolerated (**2 q**; *e.r*.=96:4), substituents in the 6‐position led to significantly lower enantioselectivities (**2 p**, **2 r**, **2 s**; *e.r*.<76:24). Even more striking, a substituent in the 4‐position (**2 v**) not only decreased the enantioselectivity, but also resulted in a significantly lower conversion and yield. Finally, we also tested analogous ester derivatives but as can be seen for products **2 t** and **2 u**, the ester group led to lower selectivities (*e.r*.<80:20) compared to the initially used nitrile group.

Overall, these investigations revealed a rather strong and somewhat unexpected influence of the nature of the substrates on the enantioselectivities of these α‐trifluoromethylthiolation reactions. The exact reasons for these remarkable selectivity dependencies on seemingly subtle variations in the starting materials are not yet clearly understood. However, these results demonstrate that the stereo‐defining non‐covalent catalyst‐substrate interactions are supposed to be rather sensitive to disturbance by steric and/or electronic alterations in the starting materials. Nevertheless, despite the somewhat lower generality of this asymmetric α‐trifluoromethylthiolation protocol, this methodology still gives access to a broad variety of differently substituted novel 3‐SCF_3_‐containing 3,3‐disubstituted isoindolinones **2** in high yields with moderate to excellent enantioselectivities.

**Scheme 3:**
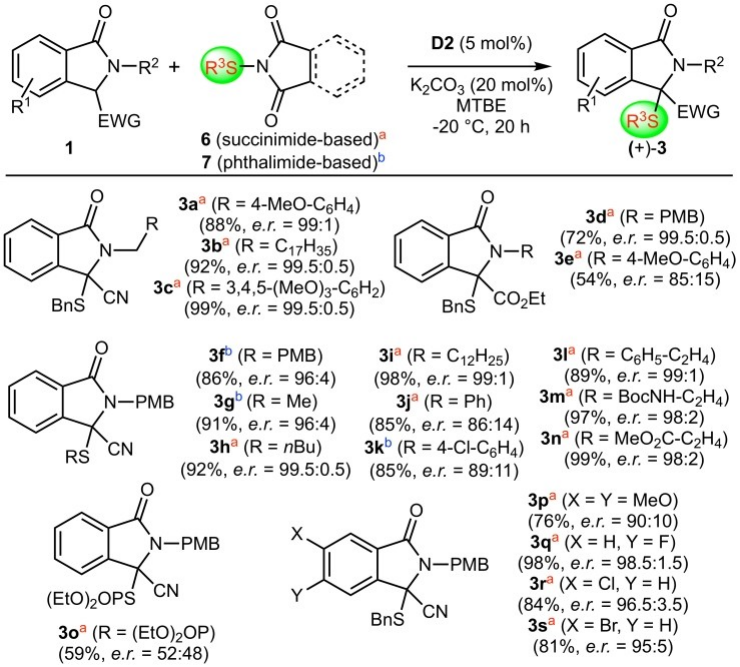
Application scope for the asymmetric α‐sulfanylation of isoindolinones **1** (the (+)‐enantiomers of products **3** were favored in all cases).

Building on the knowledge gathered hereby, we also tested analogous α‐sulfanylation reactions[^16^, ^17^] to access products **3** (Scheme 3). Literally the first experiment using the conditions developed for the trifluoromethylthiolation (Scheme 2) gave product **3 a** with almost perfect enantioselectivity (*e.r*.=99:1). Based on this very encouraging initial result, which makes further optimization more or less unnecessary, we immediately used these conditions for a variety of different combinations of isoindolinones **1** and electrophiles **6** or **7** (it should be noted that we carried out a few experiments with different conditions, but as expected, no further improvement was possible).

As outlined in Scheme 3, a variety of different nucleophile/electrophile combinations were well tolerated. Most targets **3** were obtained in satisfying yields with good to excellent enantioselectivities (up to *e.r*.=99.5:0.5) and overall this reaction was found to be more generally applicable compared to the synthesis of the SCF_3_‐products **2**. More specifically, also ester‐containing products **3 d** and **3 e** were accessed with reasonable selectivities, although again the nature of the N‐protecting group turned out to have a significant effect. In addition, a broad variety of different S‐transfer agents were well tolerated (compare products **3 f**–**3 n**) and only the ArS‐containing targets **3 j** and **3 k** were obtained with slightly reduced selectivities. Synthesis of the thiophosphate **3 o** was in principle possible as well, but unfortunately in this case only racemic product could be obtained. Also, in sharp contrast to the synthesis of products **2**, ring substitutions were much better tolerated herein (products **3 p**–**3 s**), underscoring the broad applicability of this α‐sulfanylation reaction.

**Scheme 4:**
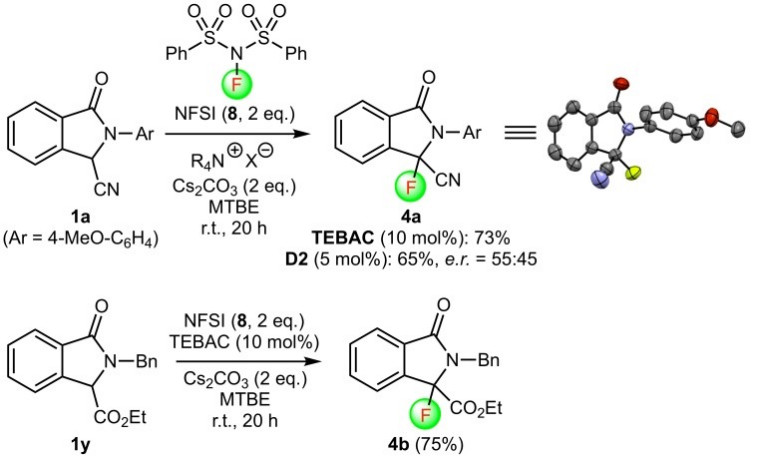
Proof‐of‐concept for the α‐fluorination of isoindolinones **1**.

Next, we also investigated the (asymmetric) electrophilic α‐fluorination of isoindolinones **1** (Scheme 4).^[27]^ Hereby, our initial focus was on the feasibility of this transformation and the stability of the α‐fluorinated products **4** in general. Our concerns related to the stability of compounds **4** are based on the fact that these compounds contain the fragile N−C−F motive. This structural element is well‐known to rapidly eliminate fluoride, which is one of the main reasons why α‐F‐α‐amino acid derivatives are very rare and sensitive motives.[^28^, ^29^, ^30^] However, given the fact that the presence of an electron‐withdrawing N‐substituent is supposed to increase the stability of the α‐F‐α‐amino acid motive[^29^, ^30^] we tested the α‐fluorination of nucleophilic isoindolinones **1**.

Indeed, it was possible to carry out the racemic fluorination of the nitrile‐based **1 a** with NFSI (**8**) in the presence of benzyltriethylammonium chloride (TEBAC) as an achiral ammonium salt phase‐transfer catalyst (Scheme 4). Noteworthy, product **4 a** turned out to be sufficiently stable to be fully characterized by means of NMR, HRMS and even single crystal X‐ray analysis.^[26]^ However, it was also found that this compound decomposes in the presence of solvents, acids and bases. When testing the α‐fluorination of differently substituted starting materials **1** the outcome was unfortunately less encouraging. For example, the corresponding N‐PMB‐containing starting material **1 a** only gave traces of the corresponding α‐fluorinated product (accompanied with significant amounts of decomposition products). Analogous ester‐based substrates were tested either and although product **4 b** could be obtained in reasonable yield, it was found to be relatively unstable as well and other ester derivatives turned out to be capricious too (mainly formation of decomposition products again, indicating that α‐fluorination proceeds but that the products **4** are not stable).

Nevertheless, based on the proof‐of‐concept for the racemic formation of **4 a** and **4 b** we also tested the asymmetric variant for the synthesis of **4 a**. Maruoka's bifunctional catalyst **D2** allowed for reasonable conversion (65% isolated yield), while our bifunctional systems **B** did not lead to any product formation. Noteworthy, we observed almost quantitative decomposition when subjecting **4 a** to HPLC analysis and we thus measured the *e.r*. by using Kim's chiral Al‐based NMR shift reagent,^[31]^ which was recently found to be a powerful tool to determine the *e.r*. of chiral fluorinated targets.^[32]^ Unfortunately however, we only observed very low levels of asymmetric induction (*e.r*.=55:45)^[22]^ and therefore, considering the pronounced lability of these compounds as well, did not investigate the asymmetric synthesis of compounds **4** further.

**Scheme 5:**
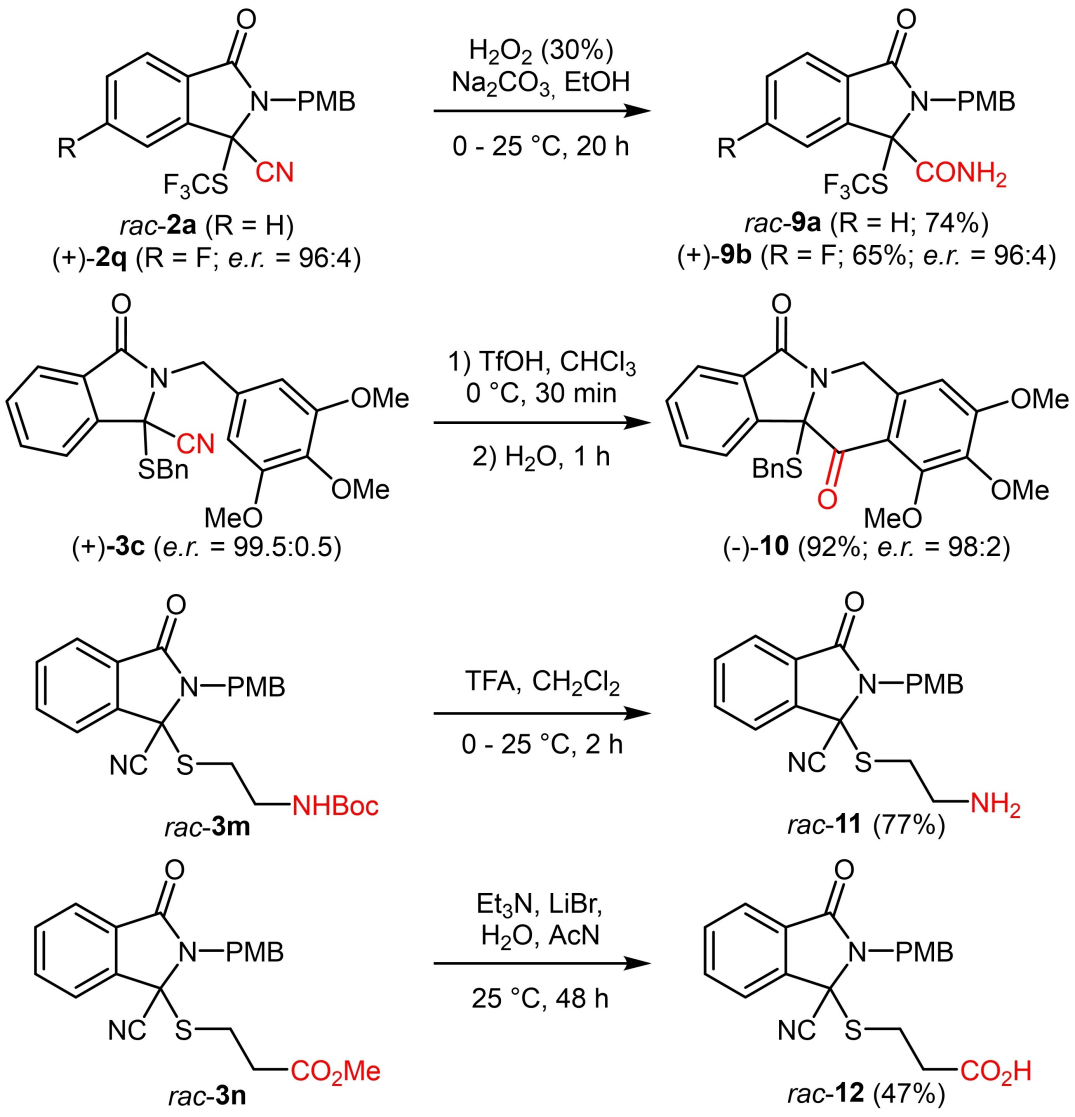
Further transformations of products **2** and **3**.

Finally, we wanted to demonstrate the suitability of products **2** and **3** for further transformations. As outlined in Scheme 5, standard functional group manipulations like nitrile hydrolysis (giving products **9 a** and **9 b** without any loss of enantiomeric composition when carried out on enantioenriched starting material **2 q**), N‐Boc‐deprotection (product **11**) and ester hydrolysis (product **12**) were carried out straightforwardly. In addition, product **3 c** could undergo an intramolecular Houben‐Hoesch reaction to access the tetracyclic product **10** directly (with almost complete preservation of its enantioenrichment). It should be emphasized that no further optimizations to improve the isolated yields were undertaken and that these reactions mainly serve as a proof‐of‐concept to illustrate the versatility of these compounds.

## Conclusion

We succeeded in developing efficient protocols to access a broad variety of differently substituted 3‐CF_3_S‐ and 3‐RS‐containing 3,3‐disubstituted isoindolinones **2** and **3** by means of asymmetric ammonium salt‐catalyzed electrophilic α‐heterofunctionalizations of the nucleophilic isoindolinones **1**. Key to success was the use of bifunctional chiral ammonium salt catalysts, i. e. Maruoka's free OH‐containing binaphthyl‐based salt **D2**. In addition, we also carried out the racemic α‐fluorination of starting materials **1** to access the compounds **4**. These products contain the labile α‐F‐α‐amino acid motive but, depending on conditions and substitution pattern, we were able to isolate and characterize these sensitive compounds.

## Experimental Section^[22]^

### General Procedure for the Asymmetric Syntheses of Products 2 and 3

Isoindolinone **1** (0.10 mmol), catalyst **D2** (0.005 mmol) and K_2_CO_3_ (0.02 mmol) were dissolved in MTBE (2 mL) at −20 °C and stirred for 15 min. Reagent **5**, **6** or **7** (0.105 mmol) was added at once and the suspension was stirred at −20 °C. After 20 h, the reaction mixture was diluted with Et_2_O (5 mL) and filtered through a pad of Na_2_SO_4_ (washed with Et_2_O). The solvent was evaporated and the crude product was purified by column chromatography (silica gel, heptanes/EtOAc) to give pure enantioenriched isoindolinones **2** and **3**. All the analytical details for the new compounds can be found in the online supporting information.

### Representative Analytic Details for Products 2 a and 3 a

**2 a**: Prepared following the general procedure and obtained as a colorless powder (35.9 mg, 0.095 mmol, 95%, *e.r*.=98:2, m.p.=98–100 °C). Rf (heptanes/EtOAc=2/1)=0.50. [α]_D_^22^ (c=0.80, CHCl_3_)=+25.1°. ^1^H NMR (300 MHz, δ, CDCl_3_, 298 K): 7.94 (d, *J*=7.4 Hz, 1H), 7.83 (d, *J*=7.6 Hz, 1H), 7.75 (td, *J*=7.6, 7.5, 1.3 Hz, 1H), 7.66 (td, *J*=7.4, 7.4, 1.2 Hz, 1H), 7.41 (d, *J*=8.6 Hz, 2H), 6.88 (d, *J*=8.7 Hz, 2H), 5.26 (d, *J*=15.3 Hz, 1H), 4.57 (d, *J*=15.3 Hz, 1H), 3.78 (s, 3H).^13^C NMR (75 MHz, δ, CDCl_3_, 298 K): 166.0, 159.9, 139.8, 133.9, 131.8, 130.6, 129.5, 127.5 (q, CF_3_, *J*_CF_=311.9 Hz), 126.7, 124.8, 124.0, 123.9, 114.2, 112.1, 65.5, 65.4, 65.4, 65.4, 55.3, 43.9. ^19^F NMR (471 MHz, δ, CDCl_3_, 298 K): −37.3 (s, 3F). HRMS (ESI): calcd m/z for C_18_H_13_F_3_N_2_O_2_S: 379.0723 [M+H]^+^; found: 379.0722. HPLC (Chiralpak AD‐H, eluent: hexane:*i*‐PrOH=10:1, 0.5 mL/min, 10 °C) retention times: t_minor_=20.6 min, t_major_=18.9 min).

**3 a**: Prepared according to the general procedure and obtained as a colorless oil (35.2 mg, 0.088 mmol, 88%, *e.r*.=99:1). Rf (heptanes/EtOAc=2/1)=0.45. [α]_D_^22^ (c=1.00, CHCl_3_)=+31.5°; ^1^H NMR (300 MHz, δ, CDCl_3_, 298 K): 7.97–7.84 (m, 1H), 7.76–7.57 (m, 3H), 7.51–7.43 (m, 2H), 7.34–7.29 (m, 1H), 7.18–7.16 (m, 2H), 6.90–6.84 (m, 2H), 6.84–6.76 (m, 2H), 4.80 (d, *J*=15.1 Hz, 1H), 4.69 (d, *J*=15.1 Hz, 1H), 3.79 (s, 3H), 3.07 (d, *J*=12.4 Hz, 1H), 3.00 (d, *J*=12.3 Hz, 1H). ^13^C NMR (75 MHz, δ, CDCl_3_, 298 K): 166.7, 159.6, 141.0, 134.3, 133.8, 131.1, 130.8, 130.4, 129.0, 128.7, 128.0, 127.8, 124.3, 123.6, 114.2, 114.1, 65.2, 55.4, 43.8, 34.0. HRMS (ESI): calcd m/z for C_24_H_21_N_2_O_2_S: 401.1318 [M+H]^+^; found: 401.1325. HPLC (YMC CHIRAL ART Amylose‐SA, eluent: hexane:*i*‐PrOH=100:1, 0.5 mL/min, 10 °C) retention times: t_minor_=50.3 min, t_major_=42.6 min.

### Racemic Synthesis of 4 a

Isoindolinone **1 a** (0.1 mmol), NFSI **8** (0.2 mmol), TEBAC (0.01 mmol) and Cs_2_CO_3_ (0.2 mmol) were mixed together in MTBE (2 mL) and stirred at room temperature overnight. After completion of the reaction (as indicated by TLC), the suspension was diluted with Et_2_O (5 mL) and filtered through a pad of celite (washed with Et_2_O). The solvent was evaporated and the crude mixture was purified by column chromatography (silica gel, heptanes/EtOAc) to give product **4 a** as light‐yellow solid (23.1 mg, 0.078 mmol, 78%). Rf (heptanes/EtOAc=2/1)=0.50. m.p.=143–145 °C. ^1^H NMR (700 MHz, δ, CDCl_3_, 298 K): 7.96 (d, *J*=7.5 Hz, 1H), 7.85 (d, *J*=7.6 Hz, 1H), 7.80 (t, *J*=7.5, 7.5 Hz, 1H), 7.75 (t, *J*=7.5, 7.5 Hz, 1H), 7.42 (d, *J*=9.0 Hz, 2H), 7.04 (d, *J*=8.9 Hz, 2H), 3.86 (s, 3H). ^13^C NMR (176 MHz, δ, CDCl_3_, 298 K): 165.9, 165.9, 160.3, 137.8, 137.6, 134.5, 134.4, 133.0, 133.0, 129.6, 129.1, 125.4, 125.0, 123.6, 115.2, 113.2, 112.9, 95.0, 93.8, 55.6. ^19^F NMR (282 MHz, δ, CDCl_3_, 298 K): −105.0. HRMS (ESI): calcd m/z for C_17_H_13_FN_2_O_2_: 297.1034 [M+H]^+^; found: 297.1040.

## Supporting information

As a service to our authors and readers, this journal provides supporting information supplied by the authors. Such materials are peer reviewed and may be re‐organized for online delivery, but are not copy‐edited or typeset. Technical support issues arising from supporting information (other than missing files) should be addressed to the authors.

Supplementary
