# Supplementary material for: Enantioselective Bifunctional Ammonium Salt‐Catalyzed Syntheses of 3‐CF3S‐, 3‐RS‐, and 3‐F‐Substituted Isoindolinones
Source: Adv Synth Catal. 2021 Feb 17;363(7):1955–62. doi: 10.1002/adsc.202100029 (PMC8050839; doi:10.1002/adsc.202100029)
Supplement: Supplementary file 1 — Supplementary [file ADSC-363-1955-s001.pdf]

Advanced Synthesis & Catalysis

Supporting Information

Enantioselective Bifunctional Ammonium Salt-Catalyzed Syntheses of 3-CF_3S-, 3-RS-, and 3-F-Substituted Isoindolinones

Andreas Eitzinger, Jan Otevrel, Victoria Haider, Antonio Macchia, Antonio Massa, Kirill Faust, Bernhard Spingler, Albrecht Berkessel, and Mario Waser*© 2021 The Authors. Advanced Synthesis & Catalysis published by Wiley-VCH GmbH. This is an open access article under the terms of the Creative Commons Attribution License, which permits use, distribution and reproduction in any medium, provided the original work is properly cited.

SUPPORTING INFORMATION

Enantioselective Bifunctional Ammonium Salt-Catalyzed Syntheses of 3-CF_3S-, 3-RS-, and 3-F-Substituted Isoindolinones

Andreas Eitzinger,[a] Jan Otevrel,[a,b] Victoria Haider,[a] Antonio Macchia,[c] Antonio Massa,[c] Kirill Faust,[d] Bernhard Spingler,[e] Albrecht Berkessel,[f] and Mario Waser[a]*

e-mail:

mario.waser@jku.at

[a] *Institute of Organic Chemistry, Johannes Kepler University Linz, Altenbergerstr. 69, 4040 Linz, Austria; +4373224685411*

[b] *Department of Chemical Drugs, Faculty of Pharmacy, Masaryk University, Palackeho 1946/1, 612 00 Brno, Czechia*

[c] *Dipartimento di Chimica e Biologia, Università di Salerno, Via Giovanni Paolo II, 132, 84084 Fisciano (SA), Italy*

[d] *Institute of Catalysis, Johannes Kepler University Linz, Altenbergerstr. 69, 4040 Linz, Austria*

[e] *Department of Chemistry, University of Zurich, Winterthurerstrasse 190, 8057 Zurich, Switzerland*

[f] *Department of Chemistry, Cologne University, Greinstrasse 4, 50939 Cologne, Germany*

List of Contents

1. General Information: 3
1.1. General Methods....................3
1.2. Single-Crystal Analysis 4
2. Detailed Screening Tables 8
2.1 Catalyst Screening 8
2.2 Solvent Screening 10
2.3 Base Screening 10
2.4 Further Conditions Tested....................10
3. Syntheses 12
3.1 Synthesis of Starting Materials **1**....................12
3.2 Synthesis of Sulfur Electrophiles **5–7**....................16
3.3 Synthesis of Racemic S-R-Isoindolinones **2–3** 19
3.4 Asymmetric Synthesis of Enantioenriched S-R-Isoindolinones **2–3** 19
3.3 Synthesis of Fluoro-Isoindolinones **4** 36
4. Further Transformations....................37
4.1 H_2O_2-Promoted Hydrolysis of the SCF_3-Cyano-Isoindolinones **2a** and **2q** 37
4.2 Houben–Hoesch Cyclization of the SBn-Cyano-Isoindolinone **3c**....................39
4.3 *N*-Boc Deprotection of the SCH_2CH_2NHBoc-Cyano-Isoindolinone **3m** 40
4.4 Methyl-Ester Hydrolysis of the $SCH_2CH_2CO_2Me$-Cyano-Isoindolinone **3n** 41
4.5 Synthesis of Bifunctional Guanidine Phase-Transfer Catalyst **B3**....................42
4.6 Synthesis of Bifunctional Isophorone Diamine-Based Phase-Transfer Catalyst **C1** 43
5. Copies of Product NMR Spectra....................46
6. Copies of Catalyst & Catalyst-Precursor NMR Spectra....................128
7. Copies of ^{19}F-NMR Spectra of Fluoro-Isoindolinone **4a** with Kim's Chiral NMR Shift Reagent 132
8. Copies of HPLC Chromatograms 134

1. General Information:

1.1. General Methods

^{1}H- and ^{13}C-NMR spectra were recorded on a Bruker Avance III 300 MHz spectrometer with a broad band observe probe and a sample changer for 16 samples, a Bruker Avance DRX 500 MHz spectrometer, and on a Bruker Avance III 700 MHz spectrometer with an Ascend magnet and TCI cryoprobe, which are both property of the Austro-Czech NMR-Research Center "RERI-uasb". NMR spectra were referenced on the solvent peak and chemical shifts are given in ppm.

High resolution mass spectra were obtained using a Thermo Fisher Scientific LTQ Orbitrap XL with an Ion Max API Source. Analyses were made in the positive ionization mode if not otherwise stated. Purine (exact mass for $[M\text{+H}]^+$ = 121.050873) and 1,2,3,4,5,6-hexakis(2,2,3,3-tetrafluoropropoxy)-1,3,5,2,4,6-triazatriphosphinane (exact mass for $[M\text{+H}]^+$ = 922.009798) were used for internal mass calibration.

HPLC was performed using a Thermo Scientific Dionex Ultimate 3000 system with diode array detector with a CHIRALPAK AD-H, CHIRAL ART Cellulose-SB or Amylose-SA (250 × 4.6 mm, 5 µm) chiral stationary phase. Optical rotations were recorded on a Schmidt + Haensch Polarimeter Model UniPol L1000 at 589 nm.

All chemicals were purchased from commercial suppliers and used without further purification unless otherwise stated. Anhydrous chloroform was distilled over calcium hydride, other dry solvents were obtained from an MBraun-SPS-800 solvent purification system. All reactions were carried out under argon atmosphere, unless stated otherwise.

1.2. Single-Crystal Analysis

Compound 2e

Crystallographic data were collected at 160.0(1) K on a Rigaku-Oxford Diffraction XtaLAB Synergy-S dual source diffractometer. This is a kappa-axis four-circle goniometer with a Dectris Pilatus3 R 200K HPC (Hybrid Photon Counting) detector and Cu and Mo PhotonJet microfocus X-ray sources. A suitable crystal of **2e** was covered with oil (Infineum V8512, formerly known as Paratone N), placed on a nylon loop that is mounted on a CrystalCap Magnetic™ pin (Hampton Research) and immediately transferred to the diffractometer. The program suite *CrysAlis^{Pro}* was used for data collection, numerical and multi-scan absorption correction as well as data reduction[1]. The structure was solved with the dual-space algorithm using *SHELXT*[2] and was refined by full-matrix least-squares methods on F^2 with *SHELXL-2018*[3] using the *Olex2* GUI[4]. The graphical output was produced with the help of the program *Mercury*[5]. CCDC 2043501 contains the supplementary crystallographic data for compound **2e**. These data can be obtained free of charge from The Cambridge Crystallographic Data Centre at www.ccdc.cam.ac.uk.

Table 1: Crystal Data, Data Collection and Structure Refinement Details for Compound **2e**.

Empirical formula	$C_{21}H_{13}F_3N_2OS$
Formula weight	398.39
Crystal system	Monoclinic
Space group	$P2_1$
a [Å]	12.6085(2)
b [Å]	7.68450(10)
c [Å]	18.8168(3)
α [°]	90
β [°]	92.081(2)
γ [°]	90
Volume [Å^3]	1821.96(5)
Z	4
Density (calculated) [Mg/m^3]	1.452
Temperature [K]	160.00(10)
Wavelength [Å]	0.71073
Absorption coefficient [mm^{-1}]	0.221
F(000)	816
Crystal size [mm^3]	0.294 × 0.193 × 0.057
Crystal description	colourless plate
Theta range for data collection [°]	1.913 to 35.230

[1] *CrysAlis^{Pro} Software system*; Rigaku Oxford Diffraction, vers. 1.171.40; Rigaku Corporation, 2019.
[2] G. M. Sheldrick, *Acta Cryst.* **2015**, *A71*, 3.
[3] G. M. Sheldrick, *Acta Cryst.* **2015**, *C71*, 3.
[4] O. V. Dolomanov, L. J. Bourhis, R. J. Gildea, J. A. K. Howard, H. Puschmann, *J. Appl. Cryst.* **2009**, *42*, 339.
[5] C. F. Macrae, L. Sovago, S. J. Cottrell, P. T. A. Galek, P. McCabe, E. Pidcock, M. Platings, G. P. Shields, J. S. Stevens, M. Towler, P. A. Wood, *J. Appl. Cryst.* **2020**, *53*, 226.

Index ranges	-20<=h<=19, -12<=k<=12, -29<=l<=29
Reflections collected	59957
Independent reflections	15126 [R(int) = 0.0333]
Reflections observed	12829
Criterion for observation	I > 2 δ (I)
Completeness to theta	99.9 % to 25.242°
Absorption correction	Gaussian
Max. and min. transmission	1.000 and 0.503
Data / restraints / parameters	15126 / 1 / 505
Goodness-of-fit on F^2	1.074
Final R indices [I > 2 ∫ (I)]	R1 = 0.0367, wR2 = 0.0909
R indices (all data)	R1 = 0.0472, wR2 = 0.0950
Absolute structure parameter	-0.024(15)
Largest diff. peak and hole [e.Å^{-3}]	0.303 and -0.175

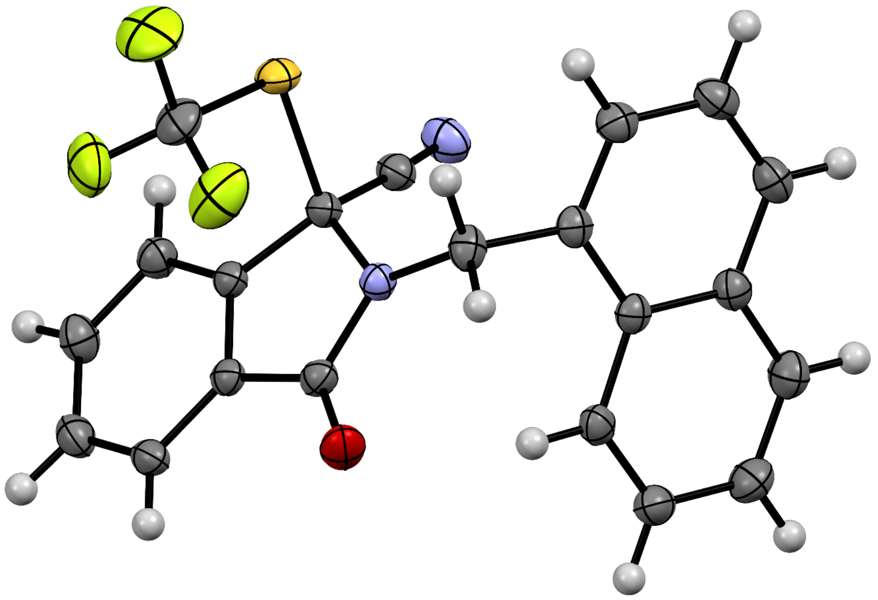

Figure 1: Displacement ellipsoid representation of **2e** at 50 % probability. A second molecule in the asymmetric unit was omitted for clarity.

Compound 4a

X-ray quality crystals were selected in Fomblin® Y H-VAC 140/13 perfluoropolyether at ambient temperature. The data was collected at 296(2) K on a *Bruker **D8 Quest Eco*** diffractometer using graphite monochromated Mo K_α radiation ($\lambda = 0.71073$ Å). The data was processed using APEX3[6],the structures were solved by intrinsic phasing (XT, Version 2014/5)[2] and refined by full matrix least squares procedures on F^2 (SHELXL, Version 2016/6)[3] using the graphical interface Shelxle[7] within the SHELXTL suite of programs by Bruker. All non-hydrogen atoms were refined anisotropically. All hydrogen atoms were calculated geometrically and a riding model was applied in the refinement process. CCDC 2042252 contains the supplementary crystallographic data for compound **4a**. These data can be obtained free of charge from The Cambridge Crystallographic Data Centre at www.ccdc.cam.ac.uk.

Table 2: Crystal Data, Data Collection and Structure Refinement Details for Compound **4a**.

Empirical formula	$C_{16}H_{11}N_2O_2F$
Formula weight [g/mol]	282.27
Color	colorless
Crystal size [mm]	0.89 × 0.84 × 0.75
Crystal system	monoclinic
Space group	$P2_1/n$
a [Å]	8.0852(7)
b [Å]	7.8019(6)
c [Å]	21.7718(19)
α [°]	90
β [°]	97.796(3)
γ [°]	90
V [Å^3]	1360.7(2)
Z	4
D_{calc} [g/cm^3]	1.378
μ [mm^{-1}]	0.10
T [K]	296
θ range [°]	2.0-19.5
No. of reflections measured	39934
No. of independent reflections	2499
Obs. Reflections with $I > 2\sigma(I)$	2070
No. of Parameters refined/restraints	192/0
Absorption correction	multi-scan
T_{min}, T_{max}	0.83, 0.93
$\Delta\rho_{min}/\Delta\rho_{max}$ [e Å^{-3}]	−0.17/0.20
F(000)	584
R_{int}	0.033
R_1 ($R[F^2 \geq 2\sigma(F^2)]$)	0.042

[6] Bruker (2017), *APEX3 v2017.3-0, SAINT V8.37A, SHELXTL-2014*, Bruker AXS Inc.: Madison (WI), USA, **2017**.
[7] C. B. Hübschle, G. M. Sheldrick, B. Dittrich, *Shelxle: a Qt graphical user interface for SHELXL, J. Appl. Crystallogr.* **2011**, *44*, 1281−1284.

wR_2 ($wR(F^2)$)	0.110
GooF	1.07
CCDC no.	2042252

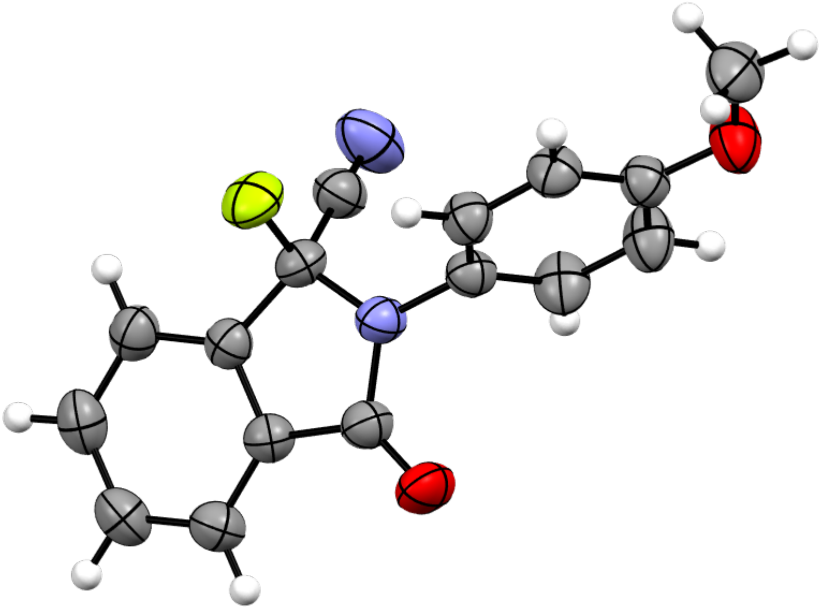

Figure 2: Displacement ellipsoid representation of **4a** at 50 % probability.

2. Detailed Screening Tables

Screening and optimization of reaction conditions for the synthesis of **2** was carried out following a general procedure: Isoindolinone **1a** (0.10 mmol), SuccN-SCF_3 reagent **5** (0.12 mmol), base and catalyst were dissolved in the respective solvent and stirred at the conditions indicated in tables 3 – 5. For reactions running below room temperature, **5** was added with a delay of 20 min after mixing substrate, catalyst and base at once. After 20 h of reaction time, the mixture was diluted with Et_2O, warmed to room temperature and filtered through a short plug of Celite®. The solvent was removed, filtered and concentrated *in vacuo*. The crude mixture was purified by silica gel column chromatography (heptanes/EtOAc = 5/1) through a short column to yield pure **2**. Note: The PhthN-SCF_3 reagent was tested as well for some of the conditions listed below and performed identically in comparison with the SuccN-SCF_3 reagent in every case. For reasons of atom economy and ease of separation, SuccN-SCF_3 **5** was used for further optimization.

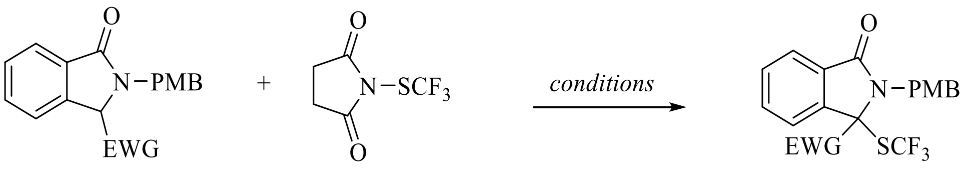

1 **5** **2**

1a (EWG = CN)
1t (EWG = CO_2Et)

2.1 Catalyst Screening

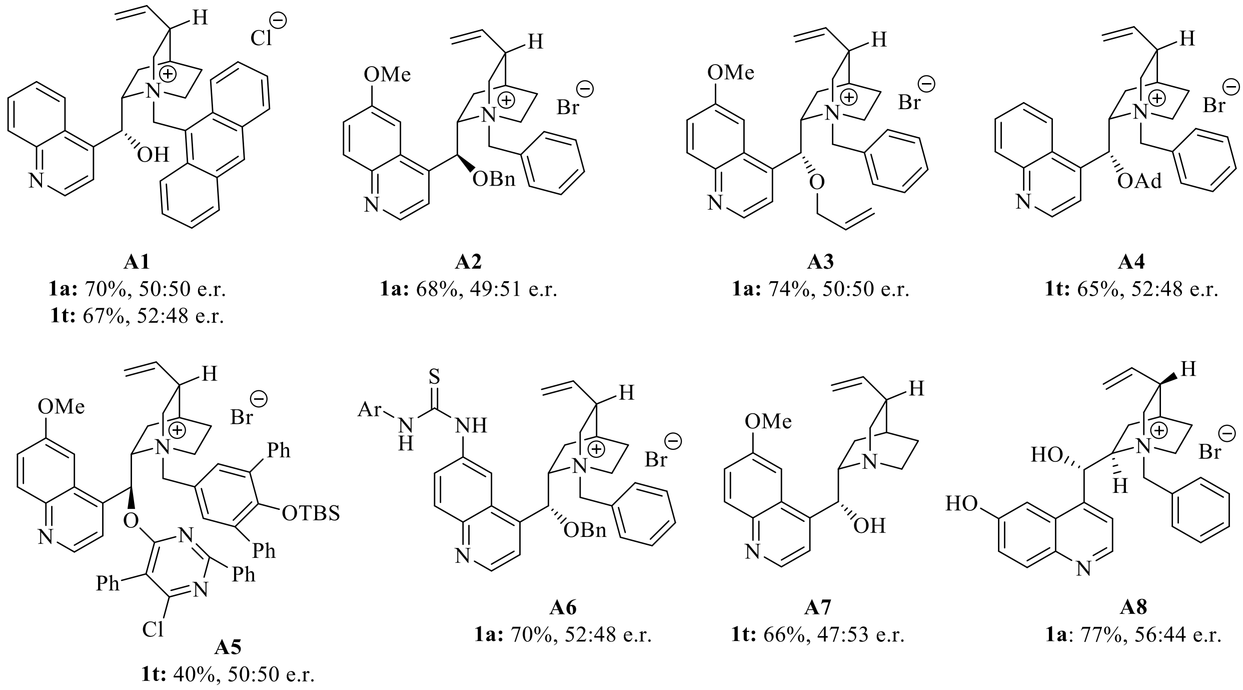

Figure 4: Cinchona-based catalysts screened for the trifluoromethylthiolation of **1a** / **1t**.

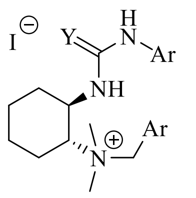

B1 (Y = O, Ar = 3,5-CF_3-C_6H_3)
B2 (Y = S, Ar = 3,5-CF_3-C_6H_3)
B3 (Y = N, Ar = 3,5-CF_3-C_6H_3)
1a (B1): 25%, 43:57 e.r.
1a (B2): 26%, 39:61 e.r.
1a (B3): 80%, 30:70 e.r.

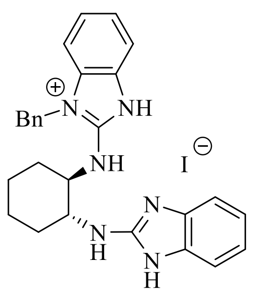

B4
1a: 76%, 50:50 e.r.

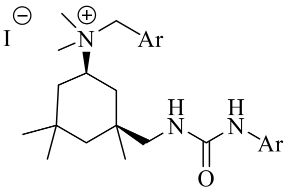

B5 (Ar = 3,5-CF_3-C_6H_3)
1a: 86%, 40:60 e.r.

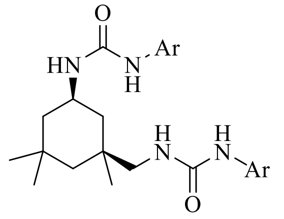

B6 (Ar = 3,5-OMe-C_6H_3)
1a: 84%, 51:49 e.r.

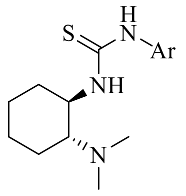

B7 (Ar = 3,5-CF_3-C_6H_3)
1a: 77%, 50:50 e.r.

Figure 5: Cyclohexane diamine and isophorone diamine-based catalysts screened for the trifluoromethylthiolation of **1a**.

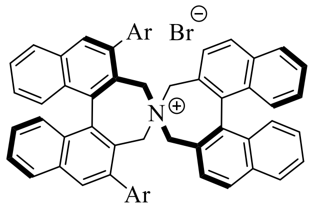

D1 (Ar = 3,5-CF_3-C_6H_3)
1a: 82%, 52:48 e.r.
1t: 48%, 51:49 e.r.

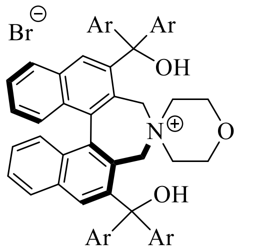

D2 (Ar = 3,5-CF_3-C_6H_3)
1a: 85%, 98:2 (MTBE)
1t: 40%, 20:80 e.r. (DCM)
73% 17:83 e.r. (MTBE)
50% conv. 24:76 e.r. (toluene)

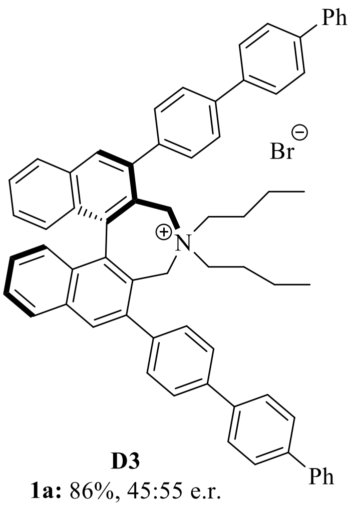

D3
1a: 86%, 45:55 e.r.

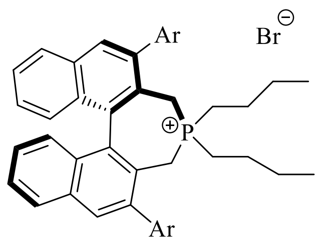

D4 (Ar = 3,5-CF_3-C_6H_3)
1a: 79%, 51:49 e.r.

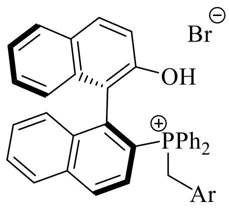

D5 (Ar = 3,4,5-F-C_6H_2)
1a: 79%, 49 :51 e.r.

Figure 6: Binaphthyl-derived catalysts screened for the trifluoromethylthiolation of **1a** / **1t**.

2.2 Solvent Screening

Table 3: Solvent Screening: Reactions were run at r.t. for 20 h using 0.10 mmol **1a**, 0.105 mmol $SuccNSCF_3$, 0.005 mmol **D2** and 0.20 mmol K_2CO_3 at c = 0.05 M (with respect to **1a**); [a] Isolated yields; [b] Determined by HPLC using a chiral stationary phase.

entry	solvent	yield / %[a]	e.r.[b]
1	**toluene**	n.d. (50 % conv.)	76 : 24
2	**dichloromethane**	44%	72 : 28
3	**methyl-*tert*-butyl ether**	95	97 : 3
4	**diethyl ether**	82	95 : 5
5	**diisopropyl ether**	94	96 : 4
6	**tetrahydrofuran**	75	84 : 16
7	**dioxane**	73	70 : 30

2.3 Base Screening

Table 4: Base Screening: Reactions were run at r.t. for 20 h using 0.10 mmol **1a**, 0.105 mmol $SuccNSCF_3$, 0.005 mmol of catalyst **D2** and indicated amount of base in MTBE at c = 0.05 M (with respect to **1a**); [a] Isolated yields; [b] Determined by HPLC using a chiral stationary phase. [c] Determined by ^1H NMR of the crude product.

entry	base	base equivalents	yield / %[a]	e.r.[b]
8	**K_2CO_3**	1	90	97 : 3
9	**K_2CO_3**	0.2	95	97 : 3
10	**Cs_2CO_3**	0.2	92	96 : 4
11	**Li_2CO_3**	0.2	85	95 : 5
12	**K_2HPO_4**	0.2	95	97 : 3
13	**K_2CO_3 (aq; 10 vol% H_2O)**	1	77	81 : 19
14	**KOH (powder)**	0.2	62	82 : 18
15	**PhthNK**	0.2	90	96 : 4

2.4 Further Conditions Tested

Table 5: LG-SCF_3^+-Reagent Screening: Reactions were run for 20 h using 0.1 mmol **1a** and 0.105 mmol $SuccNSCF_3$, indicated amounts of **D2** and K_2CO_3 in MTBE; [a] Isolated yields; [b] Determined by HPLC using a chiral stationary phase; [c] 1 mol% catalyst used; [d] 0.1 M $SuccNSCF_3$-solution (in MTBE) added over 4 h via syringe pump. The final conditions are highlighted in grey.

entry	conc. / M	base equivalents	*T* / °C	yield / %[a]	e.r.[b]
16	0.05	**2**	25	85	97 : 3
17	**0.10**	2	25	88	96 : 4
18	**0.005**	1	25	92	96 : 4
19[c]	0.05	2	25	80	86 : 14

20	0.05	2	**-20**	90	98 : 2
21	0.05	2	**-40**	82	98 : 2
22	0.05	0.2	**-20**	95	98 : 2
23[d]	0.05	1	-20	81	93 : 7

3. Syntheses

3.1 Synthesis of Starting Materials 1

Isoindolinones **1** were prepared following the procedures reported by Massa and Singh[8]. Analytical data for new compounds are given below.

Compound **1c (2-([1,1'-biphenyl]-4-ylmethyl)-3-oxoisoindoline-1-carbonitrile)**: Purified by column chromatography (heptanes/EtOAc = 2/1) and obtained as a colorless powder (75 % yield). R_f (heptanes/EtOAc = 1/1) = 0.42. m.p. = 159 – 161 °C. ^{1}H NMR (300 MHz, δ, $CDCl_3$, 298 K): 7.97 – 7.95 (m, 1H), 7.71 – 7.57 (m, 7H), 7.48 – 7.34 (m, 5H), 5.56 (d, *J* = 15.0 Hz, 1H), 5.17 (s, 1H), 4.36 (d, *J* = 15.0 Hz, 1H). ^{13}C NMR (75 MHz, δ, $CDCl_3$, 298 K): 166.9, 141.3, 140.2, 136.7, 134.1, 132.9, 131.1, 130.4, 129, 128.8, 127.8, 127.5, 127.0, 124.6, 123.1, 114.5, 48.8, 44.6. HRMS (ESI): calcd *m/z* for $C_{22}H_{16}N_2O$: 325.1335 $[M+H]^+$; found: 325.1342.

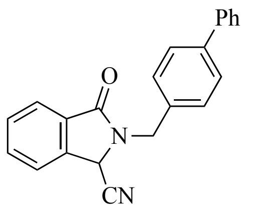

1c

Compound **1d (2-(3-bromobenzyl)-3-oxoisoindoline-1-carbonitrile)**: Purified by column chromatography (heptanes/EtOAc = 2/1) and obtained as a colorless powder (78 % yield). R_f (heptanes/EtOAc = 1/1) = 0.45. m.p. = 94 – 96 °C. ^{1}H NMR (700 MHz, δ, $CDCl_3$, 298 K): 7.94 (d, *J* = 7.5 Hz, 1H), 7.70 – 7.68 (m, 1H), 7.64 – 7.61 (m, 2H), 7.50 (s, 1H), 7.46 (d, *J* = 8.0 Hz, 1H), 7.30 (d, *J* = 7.5 Hz, 1H), 7.25 (t, *J* = 7.7 Hz, 1H), 5.43 (d, *J* = 15.3 Hz, 1H), 5.13 (s, 1H), 4.30 (d, *J* = 15.3 Hz, 1H). ^{13}C NMR (176 MHz, δ, $CDCl_3$, 298 K): 166.9, 137.5, 136.7, 133.1, 131.6, 131.4, 130.8, 130.7, 130.5, 127.1, 124.7, 123.16, 123.14, 114.3, 48.9, 44.4. HRMS (ESI): calcd *m/z* for $C_{16}H_{11}BrN_2O$: 327.0128 $[M+H]^+$; found: 327.0130.

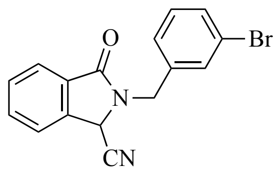

1d

Compound **1e (2-(naphthalen-1-ylmethyl)-3-oxoisoindoline-1-carbonitrile)**: Purified by column chromatography (heptanes/EtOAc = 2/1) and obtained as a colorless powder (73 % yield). R_f (heptanes/EtOAc = 1/1) = 0.47. m.p. = 157 – 159 °C. ^{1}H NMR (700 MHz, δ, $CDCl_3$, 298 K): 8.18 (d, *J* = 8.2 Hz, 1H), 7.95 (d, *J* = 7.1 Hz, 1H), 7.90 (t, *J* = 8.4 Hz, 2H), 6.68 (d, *J* = 6.7 Hz, 1H), 7.61 (dt, *J* = 14.7, 7.2 Hz, 2H), 7.58 – 7.47 (m, 4H), 6.07 (d, *J* = 14.8 Hz, 1H), 4.82 (s, 1H), 4.65 (d, *J* = 14.8 Hz, 1H). ^{13}C NMR (176 MHz, δ, $CDCl_3$, 298 K): 166.5, 136.8, 134.0,

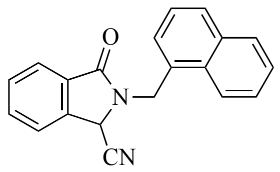

1e

[8] (a) F. Scorzelli, A. Di Mola, F. De Piano, C. Tedesco, L. Palombi, R. Filosa, M. Waser, A. Massa, *Tetrahedron,* **2017**, 73, 819-828; (b) S. Dhanasekaran, A. Suneja, V. Bisai, V. K. Singh, *Org. Lett.*, **2016**, 18, 634-637.

132.9, 131.3, 131.1, 130.6, 130.4, 129.8, 128.8, 128.7, 127.3, 126.4, 125.4, 124.7, 123.5, 123.0, 114.5, 48.9, 43.2. HRMS (ESI): calcd *m/z* for $C_{20}H_{14}N_2O$: 299.1179 $[M+H]^+$; found: 299.1184.

Compound **1f (3-oxo-2-(thiophen-2-ylmethyl)isoindoline-1-carbonitrile)**: Purified by column chromatography (heptanes/EtOAc = 2/1) and obtained as a colorless powder (70 % yield). R_f(heptanes/EtOAc = 2/1) = 0.50 °C. m.p. = 101 – 103 °C. ^{1}H NMR (700 MHz, δ, $CDCl_3$, 298 K): 7.89 (d, *J* = 7.5 Hz, 1H), 7.66 – 7.64 (m, 1H), 7.60 – 7.58 (m, 2H), 7.27 (dd, *J* = 5.2, 0.9 Hz, 1H), 7.13 (d, *J* = 3.7 Hz, 1H), 6.98 (dd, *J* = 5.1, 3.5 Hz, 1H), 5.53 (d, *J* = 15.7 Hz, 1H), 5.23 (s, 1H), 4.59 (d, *J* = 15.7 Hz, 1H). ^{13}C NMR (176 MHz, δ, $CDCl_3$, 298 K): 166.4, 137.1, 136.7, 132.9, 130.8, 130.3, 127.9, 127.1, 126.6, 124.5, 123.0, 114.4, 48.6, 39.2. HRMS (ESI): calcd *m/z* for $C_{14}H_{10}N_2OS$: 255.0587 $[M+H]^+$; found: 255.0594.

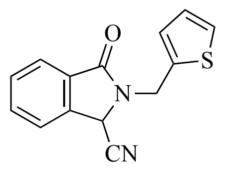

1f

Compound **1g (3-oxo-2-(thiophen-3-ylmethyl)isoindoline-1-carbonitrile)**: Purified by column chromatography (heptanes/EtOAc = 2/1) and obtained as a light-yellow powder (71 % yield). R_f(heptanes/EtOAc = 2/1) = 0.50. m.p. = 126 – 128 °C. ^{1}H NMR (700 MHz, δ, $CDCl_3$, 298 K): 7.89 (d, *J* = 7.5 Hz, 1H), 7.65 (td, *J* = 7.5, 1.1 Hz, 1H), 7.61 – 7.58 (m, 2H), 7.32 – 7.31 (m, 2H), 7.05 (dd, *J* = 4.3, 2.2 Hz, 1H), 5.36 (d, *J* = 15.3 Hz, 1H), 5.13 (s, 1H), 4.42 (d, *J* = 15.3 Hz, 1H). ^{13}C NMR (176 MHz, δ, $CDCl_3$, 298 K): 166.6, 136.7, 135.7, 132.9, 131.0, 130.3, 127.4, 127.2, 124.44, 124.39, 123.0, 114.5, 48.8, 39.7. HRMS (ESI): calcd *m/z* for $C_{14}H_{10}N_2OS$: 255.0587 $[M+H]^+$; found: 255.0593.

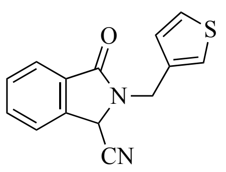

1g

Compound **1k (3-oxo-2-(3,4,5-trimethoxyphenyl)isoindoline-1-carbonitrile)**: Purified by column chromatography (heptanes/EtOAc = 1/1) and obtained as a colorless powder (77 % yield). R_f(heptanes/EtOAc = 1/1) = 0.40. m.p. = 166 – 169 °C. ^{1}H NMR (300 MHz, δ, $CDCl_3$, 298 K): 7.94 (d, *J* = 7.3 Hz, 1H), 7.74 (t, *J* = 7.3 Hz, 1H), 7.69 (d, *J* = 7.3 Hz, 1H), 7.65 (t, *J* = 7.3 Hz, 1H), 6.92 (s, 2H), 5.85 (s, 1H), 3.86 (s, 6H), 3.87 (s, 3H). ^{13}C NMR (75 MHz, δ, $CDCl_3$, 298 K): 166.1, 153.6, 136.8, 136.2, 133.5, 132.0, 131.2, 130.7, 124.8, 122.9, 115.0, 100.8, 60.9, 56.2, 51.6. HRMS (ESI): calcd *m/z* for $C_{18}H_{16}N_2O_4$: 325.1183 $[M+H]^+$; found: 325.1191.

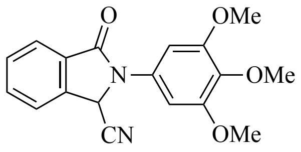

1k

Compound **1n** **(2-octadecyl-3-oxoisoindoline-1-carbonitrile)**: Purified by flash column chromatography (heptanes/EtOAc = 10/1) and obtained as a colorless powder (56 % yield). R_f (heptanes/EtOAc = 5/1) = 0.60. m.p. = 70 – 72 °C. ^{1}H NMR (300 MHz, δ, $CDCl_3$, 298 K): 7.90 (d, J = 7.3 Hz, 1H), 7.69 – 7.59 (m, 3H), 5.36 (s, 1H), 3.98 (ddd, J = 14.3, 7.2, 5.5 Hz, 1H), 3.46 (ddd, J = 14.2, 8.5, 5.7, 1H), 1.80 – 1.65 (m, 2H), 1.28 (br s, 30H), 0.89 (t, J = 6.7 Hz, 3H). ^{13}C NMR (75 MHz, δ, $CDCl_3$, 298 K): 167.2, 136.7, 132.8, 131.6, 130.4, 124.5, 123.0, 114.9, 49.6, 41.6, 31.9, 29.69, 29.66, 29.62, 29.55, 29.5, 29.4, 29.2, 28.0, 26.8, 22.7, 14.1 (some carbon signals are missing due to overlap). HRMS (ESI): calcd m/z for $C_{27}H_{42}N_2O$: 411.3370 $[M+H]^+$; found: 411.3374.

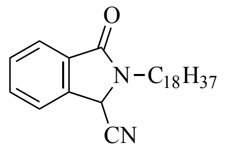

1n

Compound **1o (3-oxo-2-(2,2,2-trifluoroethyl)isoindoline-1-carbonitrile)**: Purified by crystallization (from DCM/*n*-hexane = 1/2) and obtained as a light yellow powder (65 % yield). m.p. = 128 – 130 °C. ^{1}H NMR (700 MHz, δ, $CDCl_3$, 298 K): 7.92 (d, J = 7.5 Hz, 1H), 7.74 (td, J = 7.6, 0.9 Hz, 1H), 7.69 (d, J = 7.7 Hz, 1H), 7.64 (t, J = 7.4 Hz, 1H), 5.60 (s, 1H), 4.80 (dq, J = 15.7, 9.4 Hz, 1H), 3.84 (dq, J = 15.7, 8.0 Hz, 1H). ^{13}C NMR (176 MHz, δ, $CDCl_3$, 298 K): 167.0, 136.9, 133.8, 130.7, 129.4, 124.9, 123.7 (q, J_{CF} = 280.2 Hz), 123.3, 113.8, 50.1, 42.2 (q, J_{CF} = 35.2 Hz). ^{19}F NMR (471 MHz, δ, $CDCl_3$, 298 K): -69.79 (t, J_{HF} = 8.7 Hz, 3F). HRMS (ESI): calcd m/z for $C_{11}H_7F_3N_2O$: 241.0583 $[M+H]^+$; found: 241.0582.

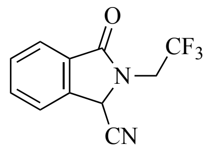

1o

Compound **1q (6-fluoro-2-(4-methoxybenzyl)-3-oxoisoindoline-1-carbonitrile)**: Purified by column chromatography (heptanes/EtOAc = 2/1) and obtained as a colorless powder (69 % yield). R_f (heptanes/EtOAc = 2/1) = 0.35. m.p. = 115 – 117 °C. ^{1}H NMR (700 MHz, δ, $CDCl_3$, 298 K): 7.90 (dd, J = 8.4, 4.7 Hz, 1H), 7.31 – 7.27 (4H), 6.89 (app d, J = 8.6 Hz, 2H), 5.39 (d, J = 14.8 Hz, 1H), 5.05 (s, 1H), 4.23 (d, J = 15.1 Hz, 1H), 3.79 (s, 3H). ^{13}C NMR (176 MHz, δ, $CDCl_3$, 298 K): 165.7, 165.5 (d, J_{CF} = 255.0 Hz), 159.7, 139.0 (d, J_{CF} = 10.0 Hz), 130.0, 127.3 (d, J_{CF} = 1.9 Hz), 126.9, 126.7 (d, J_{CF} = 10.0 Hz), 118.3 (d, J_{CF} = 23.0 Hz), 114.5, 114.0, 110.8 (d, J_{CF} = 25.5 Hz), 55.3, 48.3, 44.5. ^{19}F NMR (471 MHz, δ, $CDCl_3$, 298 K): -104.04 to -103.99 (m, 1F). HRMS (ESI): calcd m/z for $C_{17}H_{13}FN_2O_2$: 297.1034 $[M+H]^+$; found: 297.1040.

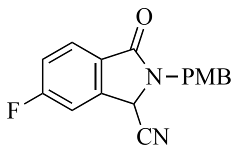

1q

Compound **1r (5-chloro-2-(4-methoxybenzyl)-3-oxoisoindoline-1-carbonitrile)**: Purified by column chromatography (heptanes/EtOAc = 2/1) and obtained as a colorless powder (71 % yield). R_f (heptanes/EtOAc = 2/1) = 0.35. m.p. = 125 – 127 °C. ^{1}H NMR (700 MHz, δ, $CDCl_3$, 298 K): 7.90 (d, J = 1.9 Hz, 1H), 7.63 (dd, J = 8.2, 1.9 Hz, 1H), 7.52 (d, J = 8.0 Hz, 1H), 7.30 – 7.28 (m, 2H), 6.91 – 6.89 (m, 2H), 5.42 (d, J = 15.1 Hz, 1H), 5.04 (s, 1H), 4.24 (d, J = 15.1 Hz, 1H), 3.81 (s, 3H). ^{13}C NMR (176 MHz, δ, $CDCl_3$, 298 K): 165.5, 159.8, 137.0, 134.8, 133.08, 133.06, 130.1, 126.8, 124.9, 124.3, 114.6, 114.1, 55.3, 48.4, 44.6. HRMS (ESI): calcd *m/z* for $C_{17}H_{13}ClN_2O_2$: 313.0738 [M+H]$^+$; found: 313.0738.

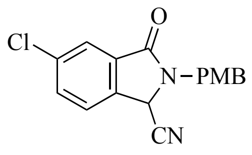

1r

Compound **1s (5-bromo-2-(4-methoxybenzyl)-3-oxoisoindoline-1-carbonitrile)**: Purified by column chromatography (heptanes/EtOAc = 2/1) and obtained as a colorless powder (63 % yield). R_f (heptanes/EtOAc = 2/1) = 0.40. m.p. = 130 – 132 °C. ^{1}H NMR (700 MHz, δ, $CDCl_3$, 298 K): 8.06 (d, J = 1.7 Hz, 1H), 7.78 (dd, J = 8.1, 1.8 Hz, 1H), 7.46 (d, J = 8.2 Hz, 1H), 7.30 – 7.28 (m, 2H), 6.91 – 6.89 (m, 2H), 5.42 (d, J = 14.8 Hz, 1H), 5.02 (s, 1H), 4.24 (d, J = 14.8 Hz, 1H), 3.81 (s, 3H). ^{13}C NMR (176 MHz, δ, $CDCl_3$, 298 K): 165.3, 159.8, 135.9, 135.3, 133.2, 130.1, 127.9, 126.8, 124.8, 124.6, 114.6, 114.0, 55.3, 48.5, 44.6. HRMS (ESI): calcd *m/z* for $C_{17}H_{13}BrN_2O_2$: 357.0233 [M+H]$^+$; found: 357.0235.

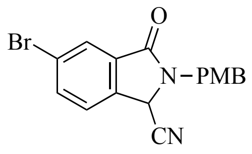

1s

Compound **1w** (**3-oxo-2-(3,4,5-trimethoxybenzyl)isoindoline-1-carbonitrile**): Purified by trituration with heptanes/EtOAc = 50/1 and obtained as a white powder (79 % yield). R_f (heptanes/EtOAc = 1/1) = 0.25. m.p. = 137 – 138 °C. ^{1}H NMR (300 MHz, δ, $CDCl_3$, 298 K): 7.94 (d, J = 7.3 Hz, 1H), 7.72 – 7.60 (m, 3H), 6.59 (s, 2H), 5.46 (d, J = 14.7, 1H), 5.11 (s, 1H), 4.20 (d, J = 14.7 Hz, 1H), 3.86 (s, 6H), 3.84 (s, 3H). ^{13}C NMR (75 MHz, δ, $CDCl_3$, 298 K): 166.6, 153.8, 138.0, 136.7, 133.0, 131.1, 130.7, 130.5, 124.7, 123.2, 114.6, 105.7, 60.8, 56.2, 48.9, 45.4. HRMS (ESI): calcd *m/z* for $C_{19}H_{18}N_2O_4$: 339.1339 [M+H]$^+$; found: 339.1346.

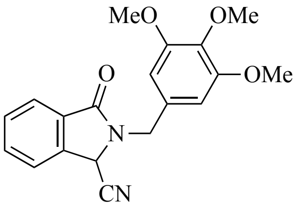

1w

3.2 Synthesis of Sulfur Electrophiles 5–7

Sulfur electrophiles: 1-((trifluoromethyl)thio)pyrrolidine-2,5-dione (**5**), 1-(benzylthio)pyrrolidine-2,5-dione (**6a**), 1-(phenylthio)pyrrolidine-2,5-dione (**6d**), 2-((4-methoxybenzyl)thio)isoindoline-1,3-dione (**7a**), 2-(methylthio)isoindoline-1,3-dione (**7b**), and 2-((4-chlorophenyl)thio)isoindoline-1,3-dione (**7c**) were prepared following established procedures[9-11].

3.2.1 General Procedure for the Synthesis of Electrophiles 6b–c, 6e–g

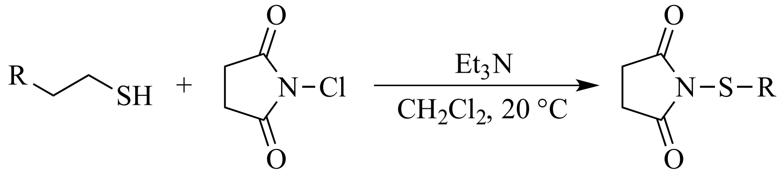

6

The following compounds were prepared by modification of the literature procedure[10]. A solution of the appropriate thiol (7.5 mmol) in dry dichloromethane (8 mL) was added at once at 20 °C (cold water bath) to a solution of *N*-chlorosuccinimide (1 equiv, 1.0 g) in dry dichloromethane (40 mL), the resulting mixture was left to stir 10 min and then treated with a solution of triethylamine (1 equiv, 1.1 mL) in dry dichloromethane (8 mL) dropwise over 30 min at 20 °C (cold water bath). The reaction medium was left to stir 1 h and then successively washed with water (5 × 50 mL). The organic phase was washed with brine once, dried over anhydrous Na_2SO_4, filtered and concentrated *in vacuo*.

Compound **6b** (**1-(butylthio)pyrrolidine-2,5-dione**): The crude residue obtained by the above method

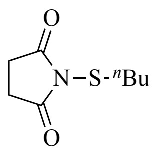

6b

was further purified by washing with *n*-pentane (5 × 10 mL), which provided **6b** as a colorless oil (1.31 g, 7.0 mmol, 93 %) that slowly decomposes at r.t. (1 month). For a longer time it should be stored in a refrigerator. R_f (heptanes/EtOAc = 2/1) = 0.23. ^{1}H NMR (300 MHz, δ, $CDCl_3$, 298 K): 2.88 – 2.84 (m, 6H), 1.58 – 1.37 (m, 4H), 0.90 (t, *J* = 7.1 Hz, 3H). ^{13}C NMR (75 MHz, δ, $CDCl_3$, 298 K): 171.1, 37.3, 29.9, 28.6, 21.6, 13.5. HRMS (ESI): calcd *m/z* for $C_8H_{14}NO_2S$: 188.0740 $[M+H]^+$; found: 188.0743.

[9] (a) C. Xu, Q. Shen, *Org. Lett.*, **2014**, 16, 7, 2046 – 2049; (b) C. Xu, B. Ma, Q. Shen, *Angew. Chem. Int. Ed.*, **2014**, 53, 9316 – 9320.
[10] A. Yasuo, N. Takeshige, T. Jitsuo, *Bull. Chem. Soc. Jpn.*, **1973**, *46*, 1898–1899.

Compound **6c** (**1-(dodecylthio)pyrrolidine-2,5-dione**): The crude residue obtained by the above method was further purified by trituration with *n*-pentane. The resulting yellowish precipitate was filtered off and dried (2.05 g, 6.8 mmol, 91 %). R_f (heptanes/EtOAc = 2/1) = 0.28. m.p. = 61 – 62 °C. ^{1}H NMR (300 MHz, δ, $CDCl_3$, 298 K): 2.87 – 2.82 (m, 6H), 1.58 – 1.48 (m, 2H), 1.40 – 1.36 (m, 2H), 1.24 (br s, 16H), 0.87 (t, J = 7.0 Hz, 3H). ^{13}C NMR (75 MHz, δ, $CDCl_3$, 298 K): 171.1, 37.6, 31.9, 29.6, 29.5, 29.4, 29.3, 29.1, 28.6, 28.4, 27.9, 22.6, 14.1. HRMS (ESI): calcd *m/z* for $C_{16}H_{30}NO_2S$: 300.1992 $[M+H]^+$; found: 300.2002.

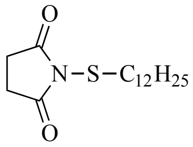

6c

Compound **6e** (**1-(phenethylthio)pyrrolidine-2,5-dione**)[11]: The crude residue obtained by the above method was further purified by trituration with *n*-pentane. The resulting white precipitate was filtered off and dried (1.70 g, 7.2 mmol, 96 %). R_f (heptanes/EtOAc = 2/1) = 0.17. m.p. = 122 – 123 °C. ^{1}H NMR (300 MHz, δ, $CDCl_3$, 298 K): 7.32 – 7.27 (m, 2H), 7.23 – 7.19 (m, 3H), 3.20 (t, J = 7.3 Hz, 2H), 2.97 (t, J = 7.2 Hz, 2H), 2.64 (s, 4H). ^{13}C NMR (75 MHz, δ, $CDCl_3$, 298 K): 177.0, 139.0, 128.5, 128.4, 126.6, 37.5, 35.3, 28.5. HRMS (ESI): calcd *m/z* for $C_{12}H_{14}NO_2S$: 236.0740 $[M+H]^+$; found: 236.0746.

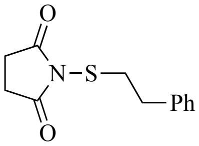

6e

Compound **6f** (***tert*-butyl (2-((2,5-dioxopyrrolidin-1-yl)thio)ethyl)carbamate**)[12]: The crude residue obtained by the above method was further purified by trituration with *n*-pentane. The resulting white precipitate was filtered off and dried (1.24 g, 4.5 mmol, 60 %). R_f (heptanes/EtOAc = 1/1) = 0.17. m.p. = 131 – 132 °C. ^{1}H NMR (300 MHz, δ, $CDCl_3$, 298 K): 5.48 (br s, 1H), 3.22 (app q, J = 6.1 Hz, 2H), 2.9 (t, J = 5.8 Hz, 2H), 2.85 (s, 4H), 1.43 (s, 9H). ^{13}C NMR (75 MHz, δ, $CDCl_3$, 298 K): 171.5, 155.7, 79.5, 38.7, 38.3, 28.6, 28.3. HRMS (ESI): calcd *m/z* for $C_{11}H_{18}N_2NaO_4S$: 297.0879 $[M+Na]^+$; found: 297.0883.

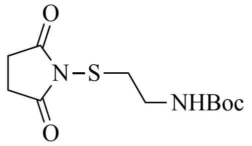

6f

Compound **6g** (**methyl 3-((2,5-dioxopyrrolidin-1-yl)thio)propanoate**): The crude residue obtained by the above method was further purified by subsequent trituration with heptanes/EtOAc = 20/1 and *n*-pentane. The resulting white precipitate was filtered off and dried (0.64 g, 3.0 mmol, 39 %). R_f (heptanes/EtOAc = 1/1) = 0.14. m.p. = 96 – 97 °C. ^{1}H NMR (300 MHz, δ, $CDCl_3$, 298 K): 3.69 (s, 3H),

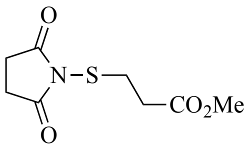

6g

[11] Y. Liang, X. Zhao, *ACS Catal.* **2019**, *9*, 6896–6902.
[12] F. Zhu, E. Miller, S.-q. Zhang, D. Yi, S. O'Neill, X. Hong, M. A. Walczak, *J. Am. Chem. Soc.* **2018**, 140, 18140–18150.

3.09 (t, J = 6.6 Hz, 2H), 2.84 (s, 4H), 2.75 (t, J = 6.5 Hz, 2H). ^{13}C NMR (75 MHz, δ, $CDCl_3$, 298 K): 177.2, 172.0, 52.0, 34.6, 32.7, 28.6. HRMS (ESI): calcd m/z for $C_8H_{12}NO_4S$: 218.0482 $[M+H]^+$; found: 218.0486.

3.2.2 Synthesis of Sulphur Electrophile 6h

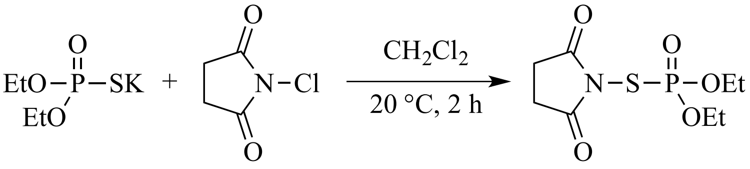

6h

The compound was prepared by modification of the literature procedure[13]. To a suspension of potassium *O*,*O*-diethyl phosphorothioate (4.8 mmol, 1.00 g) in dry dichloromethane (20 mL) was added *N*-chlorosuccinimide (1 equiv, 0.65 g) at once at 20 °C (cold water bath) and the resulting suspension was left to stir for 2 h at ambient temperature. Then the reaction mixture was diluted with dichloromethane and successively washed with water (5 × 50 mL). The organic phase was washed with brine once, dried over anhydrous Na_2SO_4, filtered and concentrated *in vacuo* to provide a crude product, which was used as is.

Compound **6h** (***S*-(2,5-dioxopyrrolidin-1-yl) *O*,*O*-diethyl phosphorothioate**): Prepared following the above procedure and obtained as a colorless oil (1.18 g, 4.4 mmol, 92 %). R_f (heptanes/EtOAc = 2/1) = 0.13. ^{1}H NMR (300 MHz, δ, $CDCl_3$, 298 K): 4.47 – 4.28 (m, 4H), 2.88 (s, 4H), 1.38 (t, J = 7.1 Hz, 6H). ^{13}C NMR (75 MHz, δ, $CDCl_3$, 298 K): 175.4, 64.8 (d, J_{CP} = 3.9 Hz), 28.7, 15.8 (d, J_{CP} = 8.3 Hz). ^{31}P NMR (202 MHz, δ, $CDCl_3$, 298 K): 21.28 – 21.11 (m, 0.24P), 19.55 – 19.39 (m, 0.76P). HRMS (ESI): calcd m/z for $C_8H_{15}NO_5PS$: 268.0403 $[M+H]^+$; found: 268.0411.

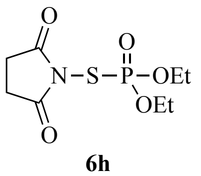

6h

[13] T. M. Chapman, D. G. Kleid, *J. Org. Chem.*, **1973**, *8*, 250–252.

3.3 Synthesis of Racemic S-R-Isoindolinones 2–3

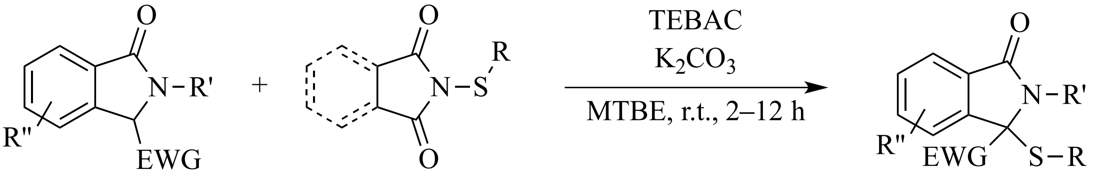

1 **5–7** **2–3**

Isoindolinone **1** (0.10 mmol), N-S-R-Succinimide or N-S-R-Phthalimide **5–7** (0.12 mmol), TEBAC (triethylbenzylammonium chloride, 0.01 mmol) and K_2CO_3 (0.20 mmol) were dissolved in MTBE (2 mL) and stirred for 2 – 12 h. After completion of the reaction (as indicated by TLC), the mixture was diluted with Et_2O (5 mL) and filtered through a pad of Celite® (washed with Et_2O) and the solvent was evaporated. The crude mixture was purified by column chromatography (silica gel, heptanes/EtOAc) to give the respective racemic N-S-R-Isoindolinones **2-3**.

3.4 Asymmetric Synthesis of Enantioenriched S-R-Isoindolinones 2–3

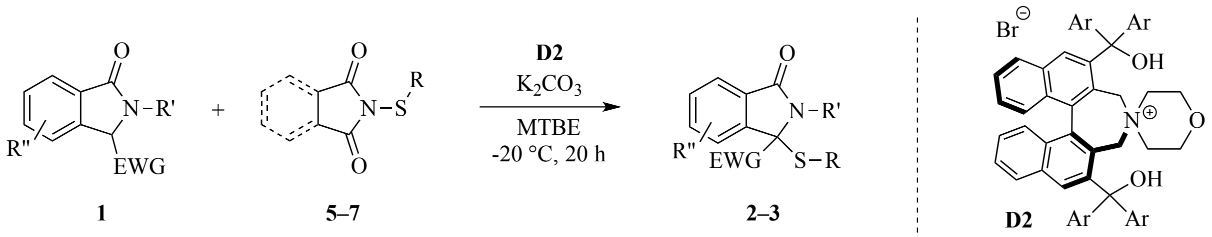

1 **5–7** **2–3** **D2**

Isoindolinone **1** (0.10 mmol), catalyst **D2** (0.005 mmol) and K_2CO_3 (0.02 mmol) were dissolved in MTBE (2 mL) at -20 °C and stirred for 15 min. N-S-R-Succinimide or N-S-R-Phthalimide **5–7** (0.105 mmol) was added at once and the suspension was stirred at -20 °C. (*Note:* In case of liquid N-S-R-Succinimide reagent, the reagent was first dissolved in MTBE at -20 °C and the remaining reaction components were added at once after 15 min of stirring). After 20 h, the reaction mixture was diluted with Et_2O (5 mL) and filtered through a pad of Na_2SO_4 (washed with Et_2O). The solvent was evaporated and the crude product was purified by column chromatography (silica gel, heptanes/EtOAc) to give pure enantioenriched N-S-R-Isoindolinones **2–3**. Analytical data for new compounds are given below.

Compound **2a** (**(*S*)-2-(4-methoxybenzyl)-3-oxo-1-((trifluoromethyl)thio)isoindoline-1-carbonitrile**): Prepared from **1a** and **5** following the general procedure and obtained as a colourless powder (35.9 mg, 0.095 mmol, 95 %, e.r. = 98:2, m.p. = 98 – 100 °C). R_f (heptanes/EtOAc = 2/1) = 0.50. $[\alpha]_D^{22}$ (c = 0.80, $CHCl_3$) = +25.1°. ^1H NMR (300 MHz, δ, $CDCl_3$, 298 K): 7.94 (d, *J* = 7.4 Hz, 1H), 7.83 (d, *J* = 7.6 Hz, 1H), 7.75 (td, *J* = 7.6, 7.5, 1.3 Hz, 1H), 7.66 (td, *J* = 7.4, 7.4, 1.2 Hz, 1H), 7.41 (d, *J* = 8.6 Hz, 2H), 6.88 (d, *J* = 8.7 Hz, 2H), 5.26 (d, *J* = 15.3 Hz, 1H), 4.57 (d, *J* = 15.3 Hz, 1H), 3.78 (s, 3H). ^{13}C NMR (75 MHz, δ, $CDCl_3$, 298 K): 166.0, 159.9, 139.8, 133.9, 131.8, 130.6, 129.5, 127.5 (q, CF3, J_{CF} = 311.9 Hz), 126.7, 124.8, 124.0, 123.9, 114.2, 112.1, 65.5, 65.4, 65.4, 65.4, 55.3, 43.9. ^{19}F NMR (282 MHz, δ, $CDCl_3$, 298 K): -37.3 (s, 3F). HRMS (ESI): calcd *m/z* for $C_{18}H_{13}F_3N_2O_2S$: 379.0723 $[M+H]^+$; found: 379.0722. HPLC (Chiralpak AD-H, eluent: hexane:*i*-PrOH = 10:1, 0.5 mL/min, 10 °C) retention times: t_{minor} = 20.6 min, t_{major} = 18.9 min.

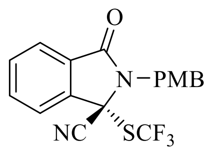

2a

Compound **2b** (**(*S*)-2-benzyl-3-oxo-1-((trifluoromethyl)thio)isoindoline-1-carbonitrile**): Prepared from **1b** and **5** according to the general procedure and obtained as a colourless oil (33.1 mg, 0.095 mmol, 95 %, e.r. = 80:20). R_f (heptanes/EtOAc = 2/1) = 0.60. $[\alpha]_D^{22}$ (c = 0.45, $CHCl_3$) = +6.9°; ^1H NMR (300 MHz, δ, $CDCl_3$, 298 K): 7.96 (d, *J* = 7.3 Hz, 1H), 7.84 (d, *J* = 7.5 Hz, 1H), 7.77 (td, *J* = 7.7, 7.5, 1.2 Hz, 1H), 7.69 (td, *J* = 7.4, 7.3, 1.1 Hz, 1H), 7.52 – 7.42 (m, 2H), 7.42 – 7.30 (m, 3H), 5.33 (d, *J* = 15.4 Hz, 1H), 4.63 (d, *J* = 15.4 Hz, 1H). ^{13}C NMR (75 MHz, δ, $CDCl_3$, 298 K): 166.1, 139.8, 134.0, 133.8, 131.9, 129.5, 129.2, 128.9, 128.7, 127.5 (q, CF_3, J_{CF} = 312.5 Hz) 125.0, 124.1, 112.1, 112.1, 65.6, 44.5. ^{19}F NMR (471 MHz, δ, $CDCl_3$, 298 K): -37.3 (s, 3F). HRMS (ESI): calcd *m/z* for $C_{17}H_{11}F_3N_2OS$: 349.0617 $[M+H]^+$; found: 349.0620. HPLC (Chiralpak AD-H, eluent: hexane:*i*-PrOH = 10:1, 0.5 mL/min, 10 °C) retention times: t_{minor} = 14.9 min, t_{major} = 13.0 min.

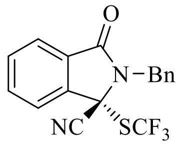

2b

Compound **2c** (**(*S*)-2-([1,1'-biphenyl]-4-ylmethyl)-3-oxo-1-((trifluoromethyl)thio)isoindoline-1-carbonitrile**): Prepared from **1c** and **5** according to the general procedure and obtained as a colourless oil (34.8 mg, 0.082 mmol, 82 %, e.r. = 90:10). R_f (heptanes/EtOAc = 5/1) = 0.35. $[\alpha]_D^{22}$ (c = 0.75, $CHCl_3$) = +12.3°; ^1H NMR (700 MHz, δ, $CDCl_3$, 298 K): 7.97 (d, *J* = 7.6 Hz, 2H), 7.85 (d, *J* = 7.7 Hz, 2H), 7.78 (td, *J* = 7.7, 7.6, 1.2 Hz, 2H), 7.70 (td, *J* = 7.5, 7.5, 0.9 Hz, 2H), 7.61 – 7.57 (m, 8H), 7.55 – 7.53 (m, 4H), 7.43 (t, *J* = 7.7, 7.7 Hz, 4H), 7.34 (t, *J* = 7.4, 7.4 Hz, 2H), 7.26 (s, 1H), 5.32 (d, *J* = 15.5 Hz, 2H), 4.70 (d, *J* = 15.5 Hz, 2H). ^{13}C NMR (176 MHz, δ, $CDCl_3$, 298 K): 166.2, 141.5, 140.7, 139.8, 134.1, 133.7, 132.0, 129.6, 129.5, 128.9, 127.6, 127.6, 127.6 (q, CF_3, J_{CF} = 312.5 Hz) 127.3, 125.0, 124.1, 112.2, 77.3, 77.0, 65.7, 44.3. ^{19}F NMR (471 MHz,

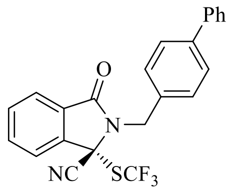

2c

δ, $CDCl_3$, 298 K): -37.2 (s, 3F). HRMS (ESI): calcd *m/z* for $C_{23}H_{15}F_3N_2OS$: 425.0930 $[M+H]^+$; found: 425.0936. HPLC (Chiralpak AD-H, eluent: hexane:*i*-PrOH = 10:1, 0.5 mL/min, 10 °C) retention times: t_{minor} = 23.7 min, t_{major} = 21.1 min.

Compound **2d** (**(*S*)-2-(3-bromobenzyl)-3-oxo-1-((trifluoromethyl)thio)isoindoline-1-carbonitrile**):

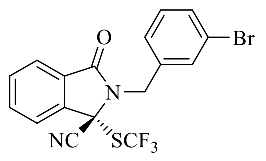

2d

Prepared from **1d** and **5** according to the general procedure and obtained as a colourless oil (41.4 mg, 0.097 mmol, 97 %, e.r. = 92:8). R_f (heptanes/EtOAc = 2/1) = 0.45. $[\alpha]_D^{22}$ (c = 1.87, $CHCl_3$) = +87.6°; ^{1}H NMR (700 MHz, δ, $CDCl_3$, 298 K): 7.97 (d, *J* = 7.6 Hz, 1H), 7.85 (d, *J* = 7.8 Hz, 1H), 7.79 (td, *J* = 7.7, 7.6, 1.2 Hz, 1H), 7.71 (td, *J* = 7.5, 7.5, 1.0 Hz, 1H), 7.64 – 7.59 (m, 1H), 7.49 – 7.45 (m, 1H), 7.41 – 7.37 (m, 1H), 7.23 (t, *J* = 7.9, 7.9 Hz, 1H), 5.25 (d, *J* = 15.7 Hz, 1H), 4.61 (d, *J* = 15.7 Hz, 1H). ^{13}C NMR (176 MHz, δ, $CDCl_3$, 298 K): 166.1, 139.6, 137.0, 134.2, 132.1, 132.1, 131.8, 130.4, 129.2, 127.7, 127.5 (q, CF_3, J_{CF} = 312.0 Hz), 125.1, 124.1, 123.0, 112.1, 77.3, 77.0, 65.5, 43.9. ^{19}F NMR (471 MHz, δ, $CDCl_3$, 298 K): -37.2 (s, 3F). HRMS (ESI): calcd *m/z* for $C_{17}H_{10}BrF_3N_2OS$: 426.9722 $[M+H]^+$; found: 426.9723. HPLC (Chiralpak AD-H, eluent: hexane:*i*-PrOH = 10:1, 0.5 mL/min, 10 °C) retention times: t_{minor} = 15.5 min, t_{major} = 13.0 min.

Compound **2e** (**(*S*)-2-(naphthalen-1-ylmethyl)-3-oxo-1-((trifluoromethyl)thio)isoindoline-1-carbonitrile**):

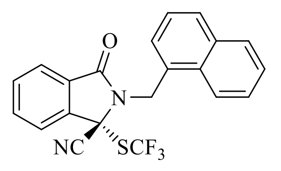

2e

Prepared from **1e** and **5** according to the general procedure and obtained as light-yellow crystals (32.7 mg, 0.082 mmol, 82 %, e.r. = 90:10, m.p. = 126 – 128 °C). R_f (heptanes/EtOAc = 2/1) = 0.62. $[\alpha]_D^{22}$ (c = 0.60, $CHCl_3$) = +41.8°; ^{1}H NMR (700 MHz, δ, $CDCl_3$, 298 K): 8.18 (d, *J* = 8.3 Hz, 1H), 7.99 (d, *J* = 7.3 Hz, 1H), 7.92 (d, *J* = 8.3 Hz, 1H), 7.89 (d, *J* = 8.0 Hz, 1H), 7.78 (d, *J* = 7.7 Hz, 1H), 7.73 (td, *J* = 7.6, 7.5, 1.1 Hz, 1H), 7.71 – 7.66 (m, 2H), 7.58 – 7.53 (m, 1H), 7.53 – 7.46 (m, 2H), 6.00 (d, *J* = 15.3 Hz, 1H), 4.92 (d, *J* = 15.3 Hz, 1H). ^{13}C NMR (176 MHz, δ, $CDCl_3$, 298 K): 165.8, 139.9, 134.1, 134.0, 132.0, 131.9, 130.3, 129.6, 129.3, 129.3, 129.1, 127.6 (q, CF_3, J_{CF} = 312.5 Hz), 127.2, 126.4, 125.2, 125.0, 124.1, 123.3, 111.6, 111.6, 77.3, 77.0, 65.4, 42.7. ^{19}F NMR (471 MHz, δ, $CDCl_3$, 298 K): -37.4 (s, 3F). HRMS (ESI): calcd *m/z* for $C_{21}H_{13}F_3N_2OS$: 399.0773 $[M+H]^+$; found: 399.0772. HPLC (Chiralpak AD-H, eluent: hexane:*i*-PrOH = 10:1, 0.5 mL/min, 10 °C) retention times: t_{minor} = 17.7 min, t_{major} = 16.4 min.

Compound **2f** (**(*S*)-3-oxo-2-(thiophen-2-ylmethyl)-1-((trifluoromethyl)thio)isoindoline-1-carbonitrile**): Prepared from **1f** and **5** according to the general procedure and obtained as a colourless oil (31.5 mg, 0.089 mmol, 89 %, e.r. = 96:4). R_f (heptanes/EtOAc = 2/1) = 0.55. $[\alpha]_D^{22}$ (c = 1.70, $CHCl_3$) = +94.3°; ^{1}H NMR (700 MHz, δ, $CDCl_3$, 298 K): 7.95 (d, *J* = 7.5 Hz, 1H), 7.84 (d, *J* = 7.8 Hz, 1H), 7.77 (td, *J* = 7.7, 7.6, 1.1 Hz, 1H), 7.69 (td, *J* = 7.5, 7.5, 0.9 Hz, 1H), 7.32 (dd, *J* = 5.1, 0.9 Hz, 1H), 7.23 (d, *J* = 3.5 Hz, 1H), 6.99 (dd, *J* = 5.1, 3.5 Hz, 1H), 5.42 (d, *J* = 15.9 Hz, 1H), 4.86 (d, *J* = 15.9 Hz, 1H). ^{13}C NMR (176 MHz, δ, $CDCl_3$, 298 K): 165.6, 139.8, 136.5, 134.1, 132.0, 129.4, 129.2, 127.6 (q, CF_3, J_{CF} = 312.4 Hz) 127.2, 127.1, 125.0, 124.1, 111.9, 111.9, 77.3, 77.2, 77.0, 65.2, 65.2, 38.9. ^{19}F NMR (471 MHz, δ, $CDCl_3$, 298 K): -37.3 (s, 3F). HRMS (ESI): calcd *m/z* for $C_{15}H_9F_3N_2OS_2$: 355.0181 $[M+H]^+$; found: 355.0180. HPLC (Chiralpak AD-H, eluent: hexane:*i*-PrOH = 10:1, 0.5 mL/min, 10 °C) retention times: t_{minor} = 17.1 min, t_{major} = 15.6 min.

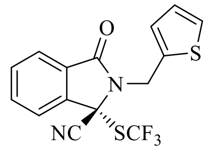

2f

Compound **2g** (**(*S*)-3-oxo-2-(thiophen-3-ylmethyl)-1-((trifluoromethyl)thio)isoindoline-1-carbonitrile**): Prepared from **1g** and **5** according to the general procedure and obtained as a colourless oil (30.5 mg, 0.086 mmol, 86 %, e.r. = 97:3). R_f (heptanes/EtOAc = 2/1) = 0.55. $[\alpha]_D^{22}$ (c = 1.60, $CHCl_3$) = +102.7°; ^{1}H NMR (700 MHz, δ, $CDCl_3$, 298 K): 7.94 (d, *J* = 7.5 Hz, 1H), 7.84 (d, *J* = 7.8 Hz, 1H), 7.77 (td, *J* = 7.7, 7.6, 1.1 Hz, 1H), 7.68 (td, *J* = 7.5, 7.5, 0.9 Hz, 1H), 7.44 – 7.39 (m, 1H), 7.32 (dd, *J* = 5.0, 3.0 Hz, 1H), 7.17 (dd, *J* = 5.0, 1.3 Hz, 1H), 5.25 (d, *J* = 15.5 Hz, 1H), 4.69 (d, *J* = 15.5 Hz, 1H).^{13}C NMR (176 MHz, δ, $CDCl_3$, 298 K): 165.8, 139.8, 135.1, 134.0, 132.0, 132.0, 129.5, 128.3, 127.6 (q, CF_3, J_{CF} = 312.4 Hz) 126.9, 125.7, 124.9, 124.1, 112.1, 112.0, 77.3, 77.0, 65.4, 39.3. ^{19}F NMR (471 MHz, δ, $CDCl_3$, 298 K): -37.3 (s, 3F). HRMS (ESI): calcd *m/z* for $C_{15}H_9F_3N_2OS_2$: 355.0181 $[M+H]^+$; found: 355.0180. HPLC (Chiralpak AD-H, eluent: hexane:*i*-PrOH = 10:1, 0.5 mL/min, 10 °C) retention times: t_{minor} = 17.7 min, t_{major} = 15.1 min.

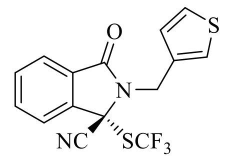

2g

Compound **2h** (**(*S*)-2-(3,4-dimethoxybenzyl)-3-oxo-1-((trifluoromethyl)thio)isoindoline-1-carbonitrile**): Prepared from **1h** and **5** according to the general procedure and obtained as a colourless oil (36.1 mg, 0.092 mmol, 92 %, e.r. = 92:8). R_f (heptanes/EtOAc = 2/1) = 0.35. $[\alpha]_D^{22}$ (c = 1.75, $CHCl_3$) = +73.2°; ^{1}H NMR (700 MHz, δ, $CDCl_3$, 298 K): 7.95 (d, *J* = 7.6 Hz, 1H), 7.84 (d, *J* = 7.8 Hz, 1H), 7.77 (td, *J* = 7.6, 1.2 Hz, 1H), 7.68 (td, *J* = 7.5, 0.9 Hz, 1H), 6.94 (d, *J* = 7.3 Hz, 2H), 6.78 (d, *J* = 8.5 Hz, 1H), 5.95 (dd, *J* = 9.1, 1.5 Hz, 2H), 5.25 (d, *J* = 15.4 Hz, 1H), 4.50 (d, *J* = 15.4 Hz, 1H). ^{13}C NMR (176 MHz, δ, $CDCl_3$, 298 K): 166.1, 148.2, 148.0, 139.8, 134.0, 131.9, 130.3, 129.5, 128.5, 128.4, 127.6 (q, CF_3, J_{CF} = 312.5 Hz) 126.7, 124.9, 124.0, 123.0, 112.1, 109.5,

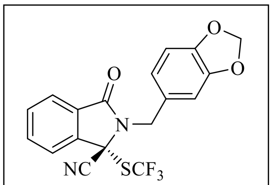

2h

108.4, 101.4, 77.3, 77.2, 77.0, 65.4, 44.3. ^{19}F NMR (471 MHz, δ, $CDCl_3$, 298 K): -37.3 (s, 3F). HRMS (ESI): calcd *m/z* for $C_{18}H_{11}F_3N_2O_3S$: 393.0515 $[M+H]^+$; found: 393.0512. HPLC (Chiralpak AD-H, eluent: hexane:*i*-PrOH = 10:1, 0.5 mL/min, 10 °C) retention times: t_{minor} = 29.4 min, t_{major} = 25.4 min.

Compound **2i** (**(*S*)-2-(4-methoxyphenyl)-3-oxo-1-((trifluoromethyl)thio)isoindoline-1-carbonitrile**): Prepared from **1i** and **5** according to the general procedure and obtained as a colourless solid (32.8 mg, 0.090 mmol, 90 %, e.r. = 96:4). R_f (heptanes/EtOAc = 2/1) = 0.50. $[\alpha]_D^{22}$ (c = 1.20, $CHCl_3$) = +32.9°; ^{1}H NMR (700 MHz, δ, $CDCl_3$, 298 K): 8.00 (d, *J* = 7.6 Hz, 1H), 7.94 – 7.90 (m, 1H), 7.83 (td, *J* = 7.7, 7.6, 1.2 Hz, 1H), 7.76 – 7.72 (m, 1H), 7.43 (d, *J* = 8.9 Hz, 2H), 7.07 (d, *J* = 8.9 Hz, 2H), 3.88 (s, 3H). ^{13}C NMR (176 MHz, δ, $CDCl_3$, 298 K): 165.9, 160.6, 138.7, 134.2, 132.2, 130.3, 129.8, 127.8 (q, CF_3, J_{CF} = 312.5 Hz), 125.7, 125.3, 124.5, 115.2, 113.3, 55.7. ^{19}F NMR (282 MHz, δ, $CDCl_3$, 298 K): -36.5 (s, 3F). HRMS (ESI): calcd *m/z* for $C_{17}H_{11}F_3N_2O_2S$: 365.0566 $[M+H]^+$; found: 365.0570. HPLC (Chiralpak AD-H, eluent: hexane:*i*-PrOH = 10:1, 0.5 mL/min, 10 °C) retention times: t_{minor} = 41.5 min, t_{major} = 32.3 min.

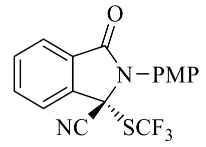

2i

Compound **2j** (**(*S*)-2-benzyl-3-oxo-1-((trifluoromethyl)thio)isoindoline-1-carbonitrile**): Prepared from **1j** and **5** according to the general procedure and obtained as a colourless oil (26.1 mg, 0.078 mmol, 78 %, e.r. = 61:39). m.p. = 84 – 86 °C. R_f (heptanes/EtOAc = 2/1) = 0.55. $[\alpha]_D^{22}$ (c = 0.90, $CHCl_3$) = +4.1°; ^{1}H NMR (700 MHz, δ, $CDCl_3$, 298 K): 8.02 (dt, *J* = 7.6, 1.0 Hz, 1H), 7.93 (d, *J* = 7.8 Hz, 1H), 7.85 (td, *J* = 7.6, 1.2 Hz, 1H), 7.75 (td, *J* = 7.5, 1.0 Hz, 1H), 7.59 – 7.54 (m, 4H), 7.54 – 7.50 (m, 1H). ^{13}C NMR (175 MHz, δ, $CDCl_3$, 298 K): 165.7, 138.7, 134.3, 133.6, 132.3, 130.0, 129.7, 129.7, 128.7, 127.7 (q, CF_3, J_{CF} = 312.5 Hz), 125.4, 124.5, 123.9, 113.3, 77.3, 77.0, 67.2. ^{19}F NMR (471 MHz, δ, $CDCl_3$, 298 K): -36.5 (s, 3F). HRMS (ESI): calcd *m/z* for $C_{16}H_9F_3N_2OS$: 335.0460 $[M+NH_4]^+$; found: 335.0465. HPLC (Chiralpak AD-H, eluent: hexane:*i*-PrOH = 10:1, 0.5 mL/min, 10 °C) retention times: t_{minor} = 18.7 min, t_{major} = 14.6 min.

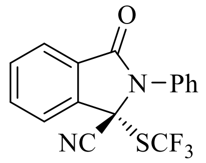

2j

Compound **2k** (**(*S*)-3-oxo-1-((trifluoromethyl)thio)-2-(3,4,5-trimethoxyphenyl)isoindoline-1-carbonitrile**): Prepared from **1k** and **5** according to the general procedure and obtained as a colourless oil (41.6 mg, 0.098 mmol, 98 %, e.r. = 87:13). R_f (heptanes/EtOAc = 2/1) = 0.22. $[\alpha]_D^{22}$ (c = 1.10, $CHCl_3$) = +24.0°; ^{1}H NMR (700 MHz, δ, $CDCl_3$, 298 K): 8.02 (d, *J* = 7.6 Hz, 1H), 7.93 (d, *J* = 7.8 Hz, 1H), 7.85 (td, *J* = 7.7, 7.6, 1.1 Hz, 1H), 7.76

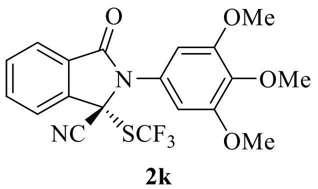

2k

(td, J = 7.5, 7.5, 0.8 Hz, 1H), 6.75 (s, 2H), 3.92 (s, 3H), 3.88 (s, 6H). ^{13}C NMR (176 MHz, δ, $CDCl_3$, 298 K): 165.7, 154.0, 139.1, 138.6, 134.4, 132.3, 129.6, 128.8, 127.8 (q, CF_3, J_{CF} = 312.1 Hz), 125.4, 124.5, 113.5, 106.1, 67.4, 61.1, 56.4. ^{19}F NMR (471 MHz, δ, $CDCl_3$, 298 K): -36.5 (s, 3F). HRMS (ESI): calcd *m/z* for $C_{15}H_{15}BrF_3NO_4S$: 425.0777 $[M+H]^+$; found: 425.0773. HPLC (Chiralpak AD-H, eluent: hexane:*i*-PrOH = 10:1, 0.5 mL/min, 10 °C) retention times: t_{minor} = 21.2 min, t_{major} = 17.8 min.

Compound **2l** ((***S***)**-2-cyclopropyl-3-oxo-1-((trifluoromethyl)thio)isoindoline-1-carbonitrile**):

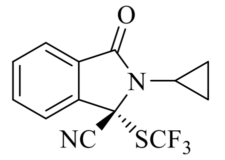

2l

Prepared from **1l** and **5** according to the general procedure and obtained as a colourless oil (25.4 mg, 0.085 mmol, 85 %, e.r. = 95:5). R_f (heptanes/EtOAc = 2/1) = 0.60. $[\alpha]_D^{22}$ (c = 0.65, $CHCl_3$) = +36.4°; ^{1}H NMR (300 MHz, δ, $CDCl_3$, 298 K): 7.88 (d, J = 7.5 Hz, 1H), 7.83 (d, J = 7.6 Hz, 1H), 7.76 (td, J = 7.7, 7.5, 1.2 Hz, 1H), 7.66 (td, J = 7.4, 7.3, 1.2 Hz, 1H), 2.85 – 2.72 (m, 1H), 1.35 – 1.20 (m, 3H), 1.01 – 0.92 (m, 1H). ^{13}C NMR (75 MHz, δ, $CDCl_3$, 298 K): 166.8, 139.0, 134.0, 132.0, 130.0, 127.8 (q, CF_3, J_{CF} = 312.1 Hz), 124.8, 124.0, 113.5, 67.0, 24.2, 6.1, 4.6. ^{19}F NMR (471 MHz, δ, $CDCl_3$, 298 K): -37.1 (s, 3F). HRMS (ESI): calcd *m/z* for $C_{13}H_9F_3N_2OS$: 299.0460 $[M+H]^+$; found: 299.0462. HPLC (Chiralpak AD-H, eluent: hexane:*i*-PrOH = 10:1, 0.5 mL/min, 10 °C) retention times: t_{minor} = 14.1 min, t_{major} = 10.7 min.

Compound **2m** ((***S***)**-2-allyl-3-oxo-1-((trifluoromethyl)thio)isoindoline-1-carbonitrile**): Prepared

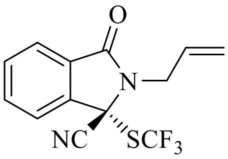

2m

from **1m** and **5** according to the general procedure and obtained as a colourless oil (26.0 mg, 0.087 mmol, 87 %, e.r. = 95:5). R_f (heptanes/EtOAc = 2/1) = 0.55. $[\alpha]_D^{22}$ (c = 0.50, $CHCl_3$) = +34.4°; ^{1}H NMR (700 MHz, δ, $CDCl_3$, 298 K): 7.92 (d, J = 7.6 Hz, 1H), 7.86 (d, J = 7.8 Hz, 1H), 7.78 (td, J = 7.7, 7.7, 1.0 Hz, 1H), 7.71 – 7.66 (m, 1H), 6.05 – 5.96 (m, 1H), 5.48 (dd, J = 17.0, 1.4 Hz, 1H), 5.37 (dd, J = 10.1, 1.3 Hz, 1H), 4.64 (ddt, J = 15.9, 5.6, 1.6, 1.6 Hz, 1H), 4.18 (dd, J = 15.9, 7.2 Hz, 1H). ^{13}C NMR (175 MHz, δ, $CDCl_3$, 298 K): 165.8, 139.7, 134.0, 132.0, 130.8, 129.7, 124.9, 124.0, 120.6, 112.8, 43.7, 29.9. ^{19}F NMR (471 MHz, δ, $CDCl_3$, 298 K): -37.2 (s, 3F). HRMS (ESI): calcd *m/z* for $C_{13}H_9F_3N_2OS$: 299.0460 $[M+H]^+$; found: 299.0462. HPLC (Chiralpak AD-H, eluent: hexane:*i*-PrOH = 10:1, 0.5 mL/min, 10 °C) retention times: t_{minor} = 11.3 min, t_{major} = 10.0 min.

Compound **2n** (**(*S*)-2-octadecyl-3-oxo-1-((trifluoromethyl)thio)isoindoline-1-carbonitrile**):

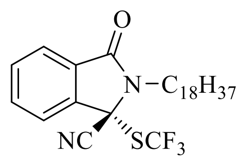

2n

Prepared from **1n** and **5** according to the general procedure and obtained as a colourless oil (43.9 mg, 0.086 mmol, 86 %, e.r. = 96:4). R_f (heptanes/EtOAc = 5/1) = 0.65. $[\alpha]_D^{22}$ (c = 0.50, $CHCl_3$) = +6.0°; ^1H NMR (700 MHz, δ, $CDCl_3$, 298 K): 7.90 (d, *J* = 7.5 Hz, 1H), 7.84 (d, *J* = 7.7 Hz, 1H), 7.76 (td, *J* = 7.7, 7.7, 1.0 Hz, 1H), 7.67 (t, *J* = 7.5, 7.5 Hz, 1H), 3.92 – 3.85 (m, 1H), 3.61 – 3.54 (m, 1H), 1.93 – 1.85 (m, 2H), 1.45 – 1.35 (m, 4H), 1.32 – 1.23 (m, 26H), 0.88 (t, *J* = 7.1, 7.1 Hz, 3H). ^{13}C NMR (176 MHz, δ, $CDCl_3$, 298 K): 166.2, 139.5, 133.8, 131.9, 130.0, 127.7 (q, CF_3, J_{CF} = 312.2 Hz), 124.7, 124.0, 113.2, 66.0, 41.7, 32.1, 29.8, 29.8, 29.8, 29.7, 29.6, 29.5, 29.3, 27.8, 27.2, 22.8. ^{19}F NMR (471 MHz, δ, $CDCl_3$, 298 K): -37.2 (s, 3F). HRMS (ESI): calcd *m/z* for $C_{28}H_{41}F_3N_2OS$: 511.2964 $[M+H]^+$; found: 511.2966. HPLC (Chiralpak AD-H, eluent: hexane:*i*-PrOH = 500:1, 0.5 mL/min, 10 °C) retention times: t_{minor} = 28.8 min, t_{major} = 27.0 min.

Compound **2o** (**(*S*)-3-oxo-2-(2,2,2-trifluoroethyl)-1-((trifluoromethyl)thio)isoindoline-1-carbonitrile**):

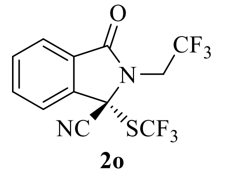

2o

Prepared from **1o** and **5** according to the general procedure and obtained as a colourless oil (27.9 mg, 0.082 mmol, 82 %, e.r. = 70:30). R_f (heptanes/EtOAc = 2/1) = 0.62. $[\alpha]_D^{22}$ (c = 0.90, $CHCl_3$) = +11.0°; ^1H NMR (700 MHz, δ, $CDCl_3$, 298 K): 7.98 (d, *J* = 7.6 Hz, 1H), 7.93 (d, *J* = 7.8 Hz, 1H), 7.86 (td, *J* = 7.7, 1.1 Hz, 1H), 7.74 (td, *J* = 7.5, 0.9 Hz, 1H), 4.60 (dq, *J* = 15.9, 9.3 Hz, 1H), 4.12 (dq, *J* = 16.6, 8.3 Hz, 1H). ^{13}C NMR (176 MHz, δ, $CDCl_3$, 298 K): 166.2, 139.6, 135.0, 132.4, 127.9, 127.3 (q, CF_3, J_{CF} = 312.4 Hz), 125.4, 124.4, 124.3, 122.7, 111.8, 77.3, 77.2, 77.0, 66.0, 42.3 (q, CF_3, J_{CF} = 37.1 Hz). ^{19}F NMR (471 MHz, δ, $CDCl_3$, 298 K): -37.5 (s, 3F), -67.8 (t, *J* = 8.3 Hz, 3F). HRMS (ESI): calcd *m/z* for $C_{12}H_6F_6N_2OS$: 373.0440 $[M+CH_3OH+H]^+$; found: 373.0447. HPLC (Chiralpak AD-H, eluent: hexane:*i*-PrOH = 10:1, 0.5 mL/min, 10 °C) retention times: t_{minor} = 13.7 min, t_{major} = 12.1 min.

Compound **2p** (**(*S*)-5,6-dimethoxy-2-(4-methoxybenzyl)-3-oxo-1-((trifluoromethyl)thio) isoindoline-1-carbonitrile**):

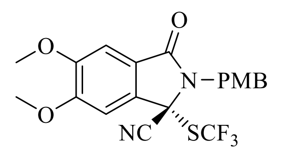

2p

Prepared from **1p** and **5** according to the general procedure and obtained as a colourless oil (36.4 mg, 0.083 mmol, 83 %, e.r. = 74:26). R_f (heptanes/EtOAc = 2/1) = 0.28. $[\alpha]_D^{22}$ (c = 1.35, $CHCl_3$) = +9.6°; ^1H NMR (300 MHz, δ, $CDCl_3$, 298 K): 7.38 (d, *J* = 8.6 Hz, 2H), 7.34 (s, 1H), 7.18 (s, 1H), 6.87 (d, *J* = 8.7 Hz, 2H), 5.22 (d, *J* = 15.4 Hz, 1H), 4.53 (d, *J* = 15.4 Hz, 1H), 4.01 (s, 3H), 3.98 (s, 3H), 3.79 (s, 3H). ^{13}C NMR (75 MHz, δ, $CDCl_3$, 298 K): 166.4, 159.8, 154.4, 152.5, 133.0, 130.6, 127.6 (q, CF_3, J_{CF} = 312.0 Hz), 127.0, 122.3, 114.2, 112.4, 105.7, 105.3, 65.2, 56.9, 56.7, 55.4, 44.0. ^{19}F NMR (471 MHz, δ, $CDCl_3$, 298 K): -37.4 (s, 3F). HRMS (ESI): calcd *m/z* for $C_{20}H_{17}F_3N_2O_4S$: 439.0934 $[M+H]^+$; found: 439.0945. HPLC (YMC

CHIRAL ART Amylose-SA, eluent: hexane:*i*-PrOH = 10:1, 0.5 mL/min, 10 °C) retention times: t_{minor} = 35.6 min, t_{major} = 52.9 min.

Compound **2q** (**(*S*)-6-fluoro-2-(4-methoxybenzyl)-3-oxo-1-((trifluoromethyl)thio)isoindoline-1-carbonitrile**): Prepared from **1q** and **5** according to the general procedure and obtained as a colourless oil (33.7 mg, 0.085 mmol, 85 %, e.r. = 96:4). R_f (heptanes/EtOAc = 2/1) = 0.45. $[\alpha]_D^{22}$ (c = 1.60, $CHCl_3$) = +90.4°; ^1H NMR (700 MHz, δ, $CDCl_3$, 298 K): 7.94 (dd, *J* = 8.4, 4.7 Hz, 1H), 7.52 (dd, *J* = 7.3, 2.2 Hz, 1H), 7.41 – 7.35 (m, 3H), 6.90 – 6.86 (m, 2H), 5.24 (d, *J* = 15.4 Hz, 1H), 4.53 (d, *J* = 15.4 Hz, 1H), 3.79 (s, 3H). ^{13}C NMR (176 MHz, δ, $CDCl_3$, 298 K): 166.8, 165.4, 165.0, 160.0, 142.3, 130.7, 127.5 (q, CF_3, J_{CF} = 312.1 Hz), 127.1, 127.1, 126.5, 125.6, 120.0, 119.9, 114.3, 111.8, 111.6, 64.8, 55.4, 44.1. ^{19}F NMR (471 MHz, δ, $CDCl_3$, 298 K): -37.2, -101.7 (m, 1F). HRMS (ESI): calcd *m/z* for $C_{18}H_{12}F_4N_2O_2S$: 397.0628 $[M+H]^+$; found: 397.0634. HPLC (Chiralpak AD-H, eluent: hexane:*i*-PrOH = 10:1, 0.5 mL/min, 10 °C) retention times: t_{minor} = 15.5 min, t_{major} = 16.3 min.

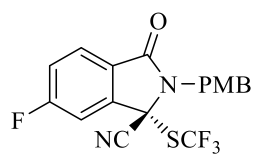

2q

Compound **2r** (**(*S*)-5-chloro-2-(4-methoxybenzyl)-3-oxo-1-((trifluoromethyl)thio)isoindoline-1-carbonitrile**): Prepared from **1r** and **5** according to the general procedure and obtained as a colourless oil (34.3 mg, 0.083 mmol, 83 %, e.r. = 76:24). R_f (heptanes/EtOAc = 2/1) = 0.50. $[\alpha]_D^{22}$ (c = 1.60, $CHCl_3$) = +71.5°; ^1H NMR (700 MHz, δ, $CDCl_3$, 298 K): 7.93 – 7.88 (m, 1H), 7.76 (d, *J* = 8.2 Hz, 1H), 7.72 (dd, *J* = 8.2, 1.9 Hz, 1H), 7.38 (d, *J* = 8.7 Hz, 2H), 6.88 (d, *J* = 8.7 Hz, 2H), 5.24 (d, *J* = 15.3 Hz, 1H), 4.54 (d, *J* = 15.3 Hz, 1H), 3.79 (s, 3H). ^{13}C NMR (176 MHz, δ, $CDCl_3$, 298 K): 164.8, 160.0, 138.6, 137.9, 134.2, 131.3, 130.8, 127.5 (q, CF_3, J_{CF} = 311.9 Hz), 126.3, 125.2, 125.1, 114.3, 111.7, 65.1, 55.4, 44.2. ^{19}F NMR (471 MHz, δ, $CDCl_3$, 298 K): -37.2 (s, 3F). HRMS (ESI): calcd *m/z* for $C_{18}H_{12}ClF_3N_2O_2S$: 413.0333 $[M+H]^+$; found: 413.0330. HPLC (Chiralpak AD-H, eluent: hexane:*i*-PrOH = 10:1, 0.5 mL/min, 10 °C) retention times: t_{minor} = 25.6 min, t_{major} = 21.3 min.

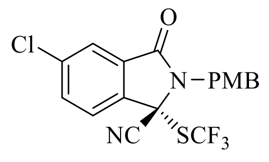

2r

Compound **2s** (**(*S*)-5-bromo-2-(4-methoxybenzyl)-3-oxo-1-((trifluoromethyl)thio)isoindoline-1-carbonitrile**): Prepared from **1s** and **5** according to the general procedure and obtained as a colourless oil (44.8 mg, 0.098 mmol, 98 %, e.r. = 67:33). R_f (heptanes/EtOAc = 2/1) = 0.45. $[\alpha]_D^{22}$ (c = 0.50, $CHCl_3$) = +17.1°; ^1H NMR (700 MHz, δ, $CDCl_3$, 298 K): 8.09 – 8.05 (m, 1H), 7.88 (dd, *J* = 8.2, 1.8 Hz, 1H), 7.69 (d, *J* = 8.2 Hz, 1H), 7.40 – 7.36 (m, 2H), 6.89 – 6.86 (m,

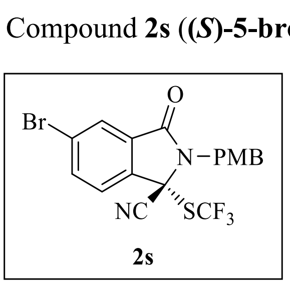

2s

2H), 5.24 (d, J = 15.3 Hz, 1H), 4.54 (d, J = 15.3 Hz, 1H), 3.79 (s, 3H). ^{13}C NMR (176 MHz, δ, $CDCl_3$, 298 K): 164.6, 160.0, 138.5, 137.0, 131.4, 130.8, 128.1, 127.5 (q, CF_3, J_{CF} = 312.1 Hz), 126.5, 126.3, 125.4, 114.3, 111.6, 77.3, 77.2, 77.0, 65.2, 55.4, 44.2. ^{19}F NMR (471 MHz, δ, $CDCl_3$, 298 K): -37.1 (s, 3F). HRMS (ESI): calcd *m/z* for $C_{18}H_{12}BrF_3N_2O_2S$: 456.9828 $[M+H]^+$; found: 456.9829. HPLC (Chiralpak AD-H, eluent: hexane:*i*-PrOH = 10:1, 0.5 mL/min, 10 °C) retention times: t_{minor} = 32.6 min, t_{major} = 22.6 min.

Compound **2t** (**ethyl (*S*)-2-(4-methoxybenzyl)-3-oxo-1-((trifluoromethyl)thio)isoindoline-1-carboxylate**): Prepared from **1t** and **5** according to the general procedure and obtained as a colourless oil (16.2 mg, 0.038 mmol, 38 %, e.r. = 80:20). R_f (heptanes/EtOAc = 2/1) = 0.45. $[\alpha]_D^{22}$ (c = 1.00, $CHCl_3$) = +40.2°; ^{1}H NMR (300 MHz, δ, $CDCl_3$, 298 K): 7.96 – 7.87 (m, 1H), 7.66 – 7.51 (m, 3H), 7.34 (d, J = 8.6 Hz, 2H), 6.81 (d, J = 8.7 Hz, 2H), 5.35 (d, J = 15.1 Hz, 1H), 4.37 (d, J = 15.1 Hz, 1H), 3.76 (s, 3H), 3.66 (dq, J = 10.7, 7.1, 7.1, 7.1 Hz, 1H), 3.18 (dq, J = 10.7, 7.2, 7.2, 7.2 Hz, 1H), 0.73 (t, J = 7.1, 7.1 Hz, 3H). ^{13}C NMR (75 MHz, δ, $CDCl_3$, 298 K): 167.7, 166.7, 159.4, 142.7, 132.9, 131.1, 130.5, 128.5 (q, CF_3, J_{CF} = 309.8 Hz), 127.9, 124.4, 122.8, 113.8, 79.3, 63.7, 55.4, 43.1, 13.2. ^{19}F NMR (471 MHz, δ, $CDCl_3$, 298 K): -38.9 (s, 3F). HRMS (ESI): calcd *m/z* for $C_{20}H_{18}F_3NO_4S$: 426.0981 $[M+H]^+$; found: 426.0989. HPLC (YMC CHIRAL ART Cellulose-SB, eluent: hexane:*i*-PrOH = 10:1, 0.5 mL/min, 10 °C) retention times: t_{minor} = 36.2 min, t_{major} = 41.8 min.

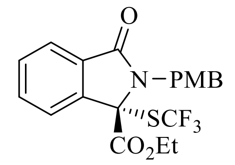

2t

Compound **2u** (**ethyl (*S*)-2-benzyl-5-bromo-3-oxo-1-((trifluoromethyl)thio)isoindoline-1-carboxylate**): Prepared from **1u** and **5** according to the general procedure and obtained as a colourless oil (37.0 mg, 0.078 mmol, 78 %, e.r. = 72:28). R_f (heptanes/EtOAc = 2/1) = 0.40. $[\alpha]_D^{22}$ (c = 0.80, $CHCl_3$) = +47.1°; ^{1}H NMR (700 MHz, δ, $CDCl_3$, 298 K): 8.06 (d, J = 1.6 Hz, 1H), 7.74 (dd, J = 8.1, 1.8 Hz, 1H), 7.43 – 7.38 (m, 3H), 7.30 (t, J = 7.4, 7.4 Hz, 2H), 7.26 – 7.24 (m, 1H), 5.39 (d, J = 15.2 Hz, 1H), 4.40 (d, J = 15.2 Hz, 1H), 3.60 (dq, J = 10.7, 7.1, 7.1, 7.1 Hz, 1H), 3.09 (dq, J = 10.8, 7.2, 7.2, 7.2 Hz, 1H), 0.71 (t, J = 7.1, 7.1 Hz, 3H). ^{13}C NMR (176 MHz, δ, $CDCl_3$, 298 K): 166.3, 166.0, 141.4, 136.0, 135.5, 132.2, 129.7, 128.6, 128.5 (q, CF_3, J_{CF} = 310.0 Hz), 128.2, 127.7, 124.9, 124.4, 79.1, 64.0, 43.9, 13.2. ^{19}F NMR (471 MHz, δ, $CDCl_3$, 298 K): -38.7 (s, 3F). HRMS (ESI): calcd *m/z* for $C_{19}H_{15}BrF_3NO_3S$: 504.0087 $[M+H]^+$; found: 504.0092. HPLC (Chiralpak AD-H, eluent: hexane:*i*-PrOH = 10:1, 0.5 mL/min, 10 °C) retention times: t_{minor} = 23.0 min, t_{major} = 15.7 min.

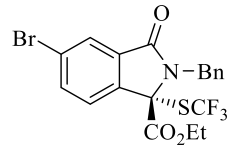

2u

Compound **2v** (**7-chloro-2-(4-methoxybenzyl)-3-oxo-1-((trifluoromethyl)thio)isoindoline-1-carbonitrile**): Prepared from **1v** and **5** according to the general procedure and obtained as a colourless oil (10.3 mg, 0.025 mmol, 25 %, e.r. = 66:34); contains 50 % impurity. R_f (heptanes/EtOAc = 2/1) = 0.50. $[\alpha]_D^{22}$ (c = 0.60, $CHCl_3$) = +4.1°; ^1H NMR (700 MHz, δ, $CDCl_3$, 298 K): 7.89 – 7.88 (m, 1H), 7.70 (dd, J = 8.0, 1.1 Hz, 1H), 7.67 – 7.64 (m, 1H), 7.41 (d, J = 8.7 Hz, 2H), 6.88 (d, J = 8.7 Hz, 2H), 5.26 (d, J = 15.4 Hz, 1H), 4.60 (d, J = 15.4 Hz, 1H), 3.79 (s, 3H). ^{13}C NMR (176 MHz, δ, $CDCl_3$, 298 K): 165.9, 159.9, 135.6, 134.9, 133.4, 131.6, 131.0, 130.7, 128.1 (q, CF_3, J_{CF} = 310.0 Hz), 126.5, 124.9, 123.4, 114.2, 55.4, 44.1. ^{19}F NMR (471 MHz, δ, $CDCl_3$, 298 K): -38.9 (s, 3F). HRMS (ESI): calcd m/z for $C_{18}H_{12}ClF_3N_2O_2S$: 413.0333 $[M+H]^+$; found: 413.0336. HPLC (YMC CHIRAL ART Cellulose-SB, eluent: hexane:i-PrOH = 10:1, 0.5 mL/min, 10 °C) retention times: t_{minor} = 25.4 min, t_{major} = 28.7 min.

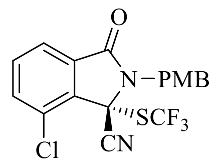

2v

Compound **3a** (**(+)-1-(benzylthio)-2-(4-methoxybenzyl)-3-oxoisoindoline-1-carbonitrile**): Prepared from **1a** and **6a** according to the general procedure and obtained as a colourless oil (35.2 mg, 0.088 mmol, 88 %, e.r. = 99:1). R_f (heptanes/EtOAc = 2/1) = 0.45. $[\alpha]_D^{22}$ (c = 1.00, $CHCl_3$) = +31.5°; ^{1}H NMR (300 MHz, δ, $CDCl_3$, 298 K): 7.97 – 7.84 (m, 1H), 7.76 – 7.57 (m, 3H), 7.51 – 7.43 (m, 2H), 7.34 – 7.29 (m, 1H), 7.18 – 7.16 (m, 2H), 6.90 – 6.84 (m, 2H), 6.84 – 6.76 (m, 2H), 4.80 (d, *J* = 15.1 Hz, 1H), 4.69 (d, *J* = 15.1 Hz, 1H), 3.79 (s, 3H), 3.07 (d, *J* = 12.4 Hz, 1H), 3.00 (d, *J* = 12.3 Hz, 1H). ^{13}C NMR (75 MHz, δ, $CDCl_3$, 298 K): 166.7, 159.6, 141.0, 134.3, 133.8, 131.1, 130.8, 130.4, 129.0, 128.7, 128.0, 127.8, 124.3, 123.6, 114.2, 114.1, 65.2, 55.4, 43.8, 34.0. HRMS (ESI): calcd *m/z* for $C_{24}H_{21}N_2O_2S$: 401.1318 $[M+H]^+$; found: 401.1325. HPLC (YMC CHIRAL ART Amylose-SA, eluent: hexane:*i*-PrOH = 100:1, 0.5 mL/min, 10 °C) retention times: t_{minor} = 50.3 min, t_{major} = 42.6 min.

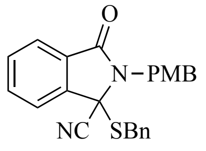

3a

Compound **3b** (**(+)-1-(benzylthio)-2-octadecyl-3-oxoisoindoline-1-carbonitrile**): Prepared from **1n** and **6a** according to the general procedure and obtained as a colourless oil (49.0 mg, 0.092 mmol, 92 %, e.r. = 99.5:0.5). R_f (heptanes/EtOAc = 2/1) = 0.70. $[\alpha]_D^{22}$ (c = 0.40, $CHCl_3$) = +26.4°; ^{1}H NMR (300 MHz, δ, $CDCl_3$, 298 K): 7.91 – 7.83 (m, 1H), 7.74 – 7.56 (m, 3H), 7.25 – 7.14 (m, 3H), 7.01 – 6.89 (m, 2H), 3.68 – 3.45 (m, 2H), 3.27 (d, *J* = 12.8 Hz, 1H), 3.16 (d, *J* = 12.8 Hz, 1H), 1.94 – 1.78 (m, 2H), 1.39 – 1.22 (m, 30H), 0.91 – 0.85 (m, 3H). ^{13}C NMR (75 MHz, δ, $CDCl_3$, 298 K): 166.6, 140.8, 134.6, 133.6, 131.0, 130.8, 128.9, 128.8, 127.8, 124.1, 123.6, 115.0, 77.4, 65.2, 41.1, 34.1, 32.1, 29.8, 29.8, 29.7, 29.7, 29.5, 29.4, 27.9, 27.3, 22.8, 14.3. HRMS (ESI): calcd *m/z* for $C_{34}H_{49}N_2OS$: 533.3560 $[M+H]^+$; found: 533.3563. HPLC (Chiralpak AD-H, eluent: hexane:*i*-PrOH = 30:1, 0.5 mL/min, 10 °C) retention times: t_{minor} = 21.7 min, t_{major} = 15.4 min.

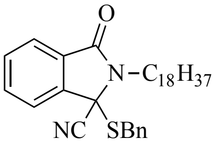

3b

Compound **3c** (**(+)-1-(benzylthio)-3-oxo-2-(3,4,5-trimethoxybenzyl)isoindoline-1-carbonitrile**): Prepared from **1w** and **6a** according to the general procedure and obtained as a colorless oil (45.6 mg, 0.099 mmol, 99 %, e.r. = 99.5:0.5). R_f (heptanes/EtOAc = 2/1) = 0.22. $[\alpha]_D^{24}$ (c = 2.00, $CHCl_3$) = +21.3°; ^{1}H NMR (300 MHz, δ, $CDCl_3$, 298 K): 7.92 (d, *J* = 7.2 Hz, 1H), 7.79 – 7.68 (m, 2H), 7.66 – 7.60 (m, 1H), 7.20 – 7.18 (m, 3H), 6.85 – 6.81 (m, 4H), 4.73, 4.69 (q, AB, *J* = 15.4 Hz, 2H), 3.85 (s, 6H), 3.84 (s, 3H), 3.10, 3.06 (q, AB, *J* = 12.4 Hz, 2H). ^{13}C NMR (75 MHz, δ, $CDCl_3$, 298 K): 166.5, 153.2, 140.8, 137.8, 133.9, 133.8, 131.2, 131.0, 130.1, 128.8, 128.7, 127.8, 124.2, 123.5, 114.2, 106.6, 65.3, 60.9, 56.2, 44.6, 34.0. HRMS (ESI): calcd *m/z* for $C_{26}H_{25}N_2O_4S$: 461.1530 $[M+H]^+$; found: 461.1537. HPLC (YMC CHIRAL

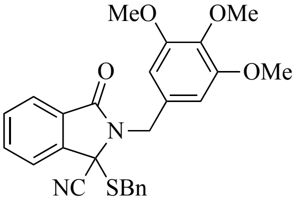

3c

ART Cellulose-SB, eluent: *n*-hexane:*i*-PrOH = 4:1, 0.5 mL/min, 10 °C) retention times: t_{minor} = 23.4 min, t_{major} = 42.5 min.

Compound **3d** (**ethyl (+)-1-(benzylthio)-2-(4-methoxybenzyl)-3-oxoisoindoline-1-carboxylate**):

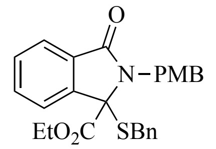

3d

Prepared from **1t** and **6a** according to the general procedure and obtained as colorless waxy solid (32.2 mg, 0.072 mmol, 72 %, e.r. = 99.5:0.5). R_f (heptanes/EtOAc = 2/1) = 0.39. $[\alpha]_D^{24}$ (c = 1.00, $CHCl_3$) = +76.2°; ^{1}H NMR (300 MHz, δ, $CDCl_3$, 298 K): 7.91 – 7.89 (m, 1H), 7.59 – 7.49 (m, 3H), 7.48 – 7.43 (m, *J* = 7.4, 2H), 7.17 – 7.12 (m, 3H), 7.86 – 7.82 (m, 4H), 4.76, 4.62 (q, AB, *J* = 14.9 Hz, 2H), 3.87 – 3.76 (m, 4H), 3.58 (dq, *J* = 10.7, 7.3 Hz, 1H), 2.86, 2.76 (q, AB, *J* = 12.3 Hz, 2H), 0.90 (t, *J* = 7.1 Hz, 3H). ^{13}C NMR (75 MHz, δ, $CDCl_3$, 298 K): 168.2, 167.3, 159.0, 143.5, 135.8, 132.7, 131.2, 130.9, 129.7, 129.0, 128.8, 128.3, 127.1, 123.6, 122.8, 113.6, 78.4, 62.9, 55.3, 43.4, 32.6, 13.4. HRMS (ESI): calcd *m/z* for $C_{26}H_{26}NO_4S$: 448.1577 $[M+H]^+$; found: 448.1587. HPLC (YMC CHIRAL ART Cellulose-SB, eluent: *n*-hexane:*i*-PrOH = 4:1, 0.5 mL/min, 10 °C) retention times: t_{minor} = 29.2 min, t_{major} = 21.3 min.

Compound **3e** (**ethyl (+)-1-(benzylthio)-2-(4-methoxyphenyl)-3-oxoisoindoline-1-carboxylate**): Prepared from **1x** and **6a** according to the general procedure and obtained as a colorless oil (23.6 mg,

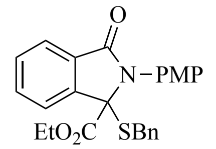

3e

0.054 mmol, 54 %, e.r. = 84.5:15.5). R_f (heptanes/EtOAc = 2/1) = 0.33. $[\alpha]_D^{24}$ (c = 0.4, $CHCl_3$) = +6.5°; ^{1}H NMR (300 MHz, δ, $CDCl_3$, 298 K): 7.96 – 7.93 (m, 1H), 7.66 – 7.55 (m, 3H), 7.52 – 7.46 (m, 2H), 7.17 – 7.15 (m, 3H), 7.01 – 6.94 (m, 4H), 4.25 – 4.07 (m, 2H), 3.85 (s, 3H), 3.36 (d, *J* = 12.3 Hz, 1H), 3.01 (d, *J* = 12.1 Hz, 1H), 1.14 (t, *J* = 7.2 Hz, 3H). ^{13}C NMR (75 MHz, δ, $CDCl_3$, 298 K): 166.3, 159.6, 140.8, 137.9, 137.8, 133.0, 130.7, 130.6, 129.7, 129.3, 127.4, 124.3, 124.0, 123.3, 114.0, 113.9, 66.6, 55.2, 43.9. HRMS (ESI): calcd *m/z* for $C_{25}H_{24}NO_4S$: 434.1421 $[M+H]^+$; found: 434.1429. HPLC (YMC CHIRAL ART Cellulose-SB, eluent: *n*-hexane:*i*-PrOH = 4:1, 0.5 mL/min, 10 °C) retention times: t_{minor} = 40.7 min, t_{major} = 27.2 min.

Compound **3f** (**(+)-2-(4-methoxybenzyl)-1-((4-methoxybenzyl)thio)-3-oxoisoindoline-1-**

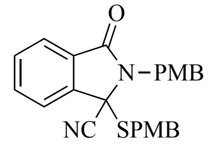

3f

carbonitrile): Prepared from **1a** and **7a** according to the general procedure and obtained as colorless waxy solid (37.0 mg, 0.086 mmol, 86 %, e.r. = 96:4). R_f (heptanes/EtOAc = 2/1) = 0.33. $[\alpha]_D^{24}$ (c = 1.00, $CHCl_3$) = +14.6°; ^{1}H NMR (300 MHz, δ, $CDCl_3$, 298 K): 7.92 – 7.86 (m, 1H), 7.80 – 7.75 (m, 1H), 7.70 (td, *J* = 7.4, 1.0 Hz, 1H), 7.62 (td, *J* = 7.4, 1.0 Hz, 1H), 7.51 – 7.46 (m, 2H),

6.91 – 6.86 (m, 2H), 6.76 – 6.68 (m, 4H), 4.83, 4.73 (q, AB, J = 15.1 Hz, 2H), 3.81 (s, 3H), 3.78 (s, 3H), 3.04, 2.98 (q, AB, J = 12.1 Hz, 2H). ^{13}C NMR (126 MHz, δ, CDCl$_3$, 298 K): 166.6, 159.5, 159.1, 141.0, 134.4, 133.7, 130.9, 130.7, 130.3, 130.1, 127.9, 125.7, 124.1, 123.6, 123.4, 114.0, 113.9, 65.0, 55.3, 43.7, 33.3. HRMS (ESI): calcd *m/z* for $C_{25}H_{23}N_2O_3S$: 431.1424 [M+H]$^+$; found: 431.1436. HPLC (Chiralpak AD-H, eluent: *n*-hexane:*i*-PrOH = 4:1, 0.5 mL/min, 10 °C) retention times: t_{minor} = 55.4 min, t_{major} = 57.3 min.

Compound **3g** (**(+)-2-(4-methoxybenzyl)-1-(methylthio)-3-oxoisoindoline-1-carbonitrile**): Prepared

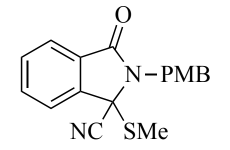

3g

from **1a** and **7b** according to the general procedure and obtained as a yellow waxy solid (29.4 mg, 0.091 mmol, 91 %, e.r. = 96:4). R_f (heptanes/EtOAc = 4/1) = 0.36. $[\alpha]_D^{24}$ (c = 1.00, CHCl$_3$) = +40.0°; ^{1}H NMR (300 MHz, δ, CDCl$_3$, 298 K): 7.92 – 7.87 (m, 1H), 7.79 – 7.71 (m, 2H), 7.65 – 7.59 (m, 1H), 7.50 – 7.45 (m, 2H), 6.89 – 6.85 (m, 2H), 4.98 (d, J = 15.0 Hz, 1H), 4.71 (d, J = 15.0 Hz, 1H), 3.80 (s, 3H), 1.46 (s, 3H). ^{13}C NMR (75 MHz, δ, CDCl$_3$, 298 K): 166.7, 159.4, 134.3, 133.7, 130.8, 130.6, 127.6, 124.0, 123.6, 123.2, 114.3, 113.8, 64.3, 55.2, 43.5, 11.1. HRMS (ESI): calcd *m/z* for $C_{18}H_{17}N_2O_2S$: 325.1005 [M+H]$^+$; found: 325.1016. HPLC (YMC CHIRAL ART Cellulose-SB, eluent: *n*-hexane:*i*-PrOH = 4:1, 0.5 mL/min, 10 °C) retention times: t_{minor} = 25.6 min, t_{major} = 23.1 min.

Compound **3h** (**(+)-1-(butylthio)-2-(4-methoxybenzyl)-3-oxoisoindoline-1-carbonitrile**): Prepared

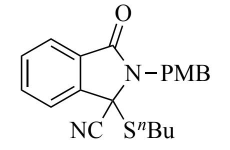

3h

from **1a** and **6b** according to the general procedure and obtained as a colorless oil (33.8 mg, 0.092 mmol, 92 %, e.r. = 99.5:0.5). R_f (heptanes/EtOAc = 2/1) = 0.50. $[\alpha]_D^{24}$ (c = 1.00, CHCl$_3$) = +37.0°; ^{1}H NMR (300 MHz, δ, CDCl$_3$, 298 K): 7.90 (d, J = 7.5 Hz, 1H), 7.79 – 7.70 (m, 2H), 7.64 – 7.59 (m, 1H), 7.48 – 7.45 (m, 2H), 7.89 – 7.83 (m, 4H), 4.96, 4.74 (q, AB, J = 15.0 Hz, 2H), 3.79 (s, 3H), 1.92 – 1.84 (m, 1H), 1.81 – 1.72 (m, 1H), 1.17 – 1.10 (m, 4H), 0.72 (t, J = 6.9 Hz, 3H). ^{13}C NMR (75 MHz, δ, CDCl$_3$, 298 K): 166.6, 159.4, 141.4, 133.6, 130.8, 130.6, 130.2, 127.8, 124.0, 123.4, 114.3, 113.8, 64.4, 55.2, 43.5, 29.5, 28.5, 21.9, 13.3. HRMS (ESI): calcd *m/z* for $C_{21}H_{23}N_2O_2S$: 367.1475 [M+H]$^+$; found: 367.1485. HPLC (YMC CHIRAL ART Cellulose-SB, eluent: *n*-hexane:*i*-PrOH = 9:1, 0.5 mL/min, 10 °C) retention times: t_{minor} = 19.8 min, t_{major} = 20.8 min.

Compound **3i** (**(+)-1-(dodecylthio)-2-(4-methoxybenzyl)-3-oxoisoindoline-1-carbonitrile**): Prepared

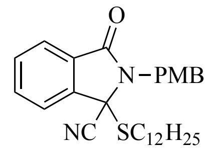

3i

from **1a** and **6c** according to the general procedure and obtained as a colorless oil (47.0 mg, 0.098 mmol, 98 %, e.r. = 99:1). R_f (heptanes/EtOAc = 2/1) = 0.58. $[\alpha]_D^{24}$ (c = 2.00, $CHCl_3$) = +22.0°; ^{1}H NMR (300 MHz, δ, $CDCl_3$, 298 K): 7.90 (d, *J* = 7.5 Hz, 1H), 7.79 – 7.69 (m, 2H), 7.64 – 7.58 (m, 1H), 7.49 – 7.44 (m, 2H), 6.89 – 6.84 (m, 2H), 4.96, 4.74 (q, AB, *J* = 15.0 Hz, 2H), 3.79 (s, 3H), 1.92 – 1.83 (m, 1H), 1.80 – 1.71 (m, 1H), 1.33 –1.07 (m, 20H), 0.89 (t, *J* = 6.7 Hz, 3H). ^{13}C NMR (75 MHz, δ, $CDCl_3$, 298 K): 166.6, 159.4, 141.5, 133.6, 130.8, 130.6, 130.2, 127.8, 124.0, 123.4, 114.3, 113.8, 64.4, 59.2, 43.5, 31.9, 29.6, 29.4, 29.31, 29.25, 28.9, 28.8, 28.7, 27.5, 22.7, 14.1. HRMS (ESI): calcd *m/z* for $C_{29}H_{39}N_2O_2S$: 479.2727 $[M+H]^+$; found: 479.2733. HPLC (YMC CHIRAL ART Cellulose-SB, eluent: *n*-hexane:*i*-PrOH = 9:1, 0.5 mL/min, 10 °C) retention times: t_{minor} = 14.9 min, t_{major} = 16.0 min.

Compound **3j** (**(+)-2-(4-methoxybenzyl)-3-oxo-1-(phenylthio)isoindoline-1-carbonitrile**): Prepared

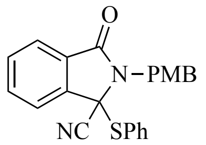

3j

from **1a** and **6d** according to the general procedure and obtained as a colourless oil (32.8 mg, 0.085 mmol, 85 %, e.r. = 86:14). R_f (heptanes/EtOAc = 2/1) = 0.45. $[\alpha]_D^{22}$ (c = 0.75, $CHCl_3$) = +38.2°; ^{1}H NMR (700 MHz, δ, $CDCl_3$, 298 K): 7.66 – 7.64 (m, 1H), 7.64 – 7.61 (m, 1H), 7.56 (dt, *J* = 7.6, 0.8, 0.8 Hz, 1H), 7.45 – 7.42 (m, 3H), 7.29 – 7.26 (m, 1H), 7.12 – 7.09 (m, 2H), 7.02 – 6.99 (m, 2H), 6.89 – 6.86 (m, 2H), 5.21 (d, *J* = 15.1 Hz, 1H), 4.74 (d, *J* = 15.1 Hz, 1H), 3.79 (s, 3H). ^{13}C NMR (176 MHz, δ, $CDCl_3$, 298 K): 166.5, 159.6, 141.2, 137.0, 133.0, 131.1, 130.7, 130.6, 129.8, 129.2, 127.7, 126.0, 123.9, 123.6, 114.2, 114.1, 66.7, 55.4, 44.0. HRMS (ESI): calcd *m/z* for $C_{23}H_{19}N_2O_2S$: 387.1162 $[M+H]^+$; found: 387.1172. HPLC (Chiralpak AD-H, eluent: hexane:*i*-PrOH = 4:1, 0.5 mL/min, 10 °C) retention times: t_{minor} = 34.3 min, t_{major} = 71.4 min.

Compound **3k** (**(+)-1-((4-chlorophenyl)thio)-2-(4-methoxybenzyl)-3-oxoisoindoline-1-**

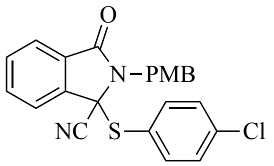

3k

carbonitrile): Prepared from **1a** and **7c** according to the general procedure and obtained as a colorless oil (35.0 mg, 0.083 mmol, 83 %, e.r. = 89:11). R_f (heptanes/EtOAc = 2/1) = 0.44. $[\alpha]_D^{24}$ (c = 1.00, $CHCl_3$) = +43.7°; ^{1}H NMR (300 MHz, δ, $CDCl_3$, 298 K): 7.67 – 7.61 (m, 3H), 7.53 – 7.43 (m, 3H), 7.11 – 7.06 (m, 2H), 6.93 – 6.88 (m, 4H), 5.18 (d, *J* = 14.9 Hz, 1H), 4.75 (d, *J* = 15.0 Hz, 1H), 3.80 (s, 3H). ^{13}C NMR (75 MHz, δ, $CDCl_3$, 298 K): 166.3, 159.6, 140.8, 137.9, 137.8, 133.0, 130.7, 130.6, 129.7, 129.3, 127.4, 124.3, 124.0, 123.3, 114.0, 113.9, 66.6, 55.2, 43.9. HRMS (ESI): calcd *m/z* for $C_{23}H_{18}ClN_2O_2S$: 421.0772 $[M+H]^+$; found: 421.0785. HPLC (YMC CHIRAL ART Cellulose-SB, eluent: *n*-hexane:*i*-PrOH = 4:1, 0.5 mL/min, 10 °C) retention times: t_{minor} = 22.8 min, t_{major} = 35.4 min.

Compound **3l** (**(+)-2-(4-methoxybenzyl)-3-oxo-1-(phenethylthio)isoindoline-1-carbonitrile**):

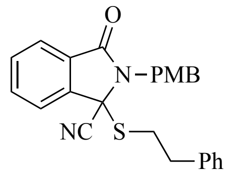

3l

Prepared from **1a** and **6e** according to the general procedure and obtained as a colorless oil (41.2 mg, 0.099 mmol, 99 %, e.r. = 99:1). R_f (heptanes/EtOAc = 2/1) = 0.42. $[\alpha]_D^{24}$ (c = 2.00, $CHCl_3$) = +18.5°; ^{1}H NMR (300 MHz, δ, $CDCl_3$, 298 K): 7.91 (d, *J* = 7.4 Hz, 1H), 7.76 – 7.69 (m, 2H), 7.64 – 7.59 (m, 1H), 7.48 – 7.44 (m, 2H), 7.26 – 7.19 (m, 3H), 6.89 – 6.86 (m, 4H), 4.88, 4.70 (q, AB, *J* = 15.0 Hz, 2H), 3.79 (s, 3H), 2.50 – 2.34 (m, 2H), 2.14 – 1.96 (m, 2H). ^{13}C NMR (75 MHz, δ, $CDCl_3$, 298 K): 166.6, 159.4, 141.2, 138.7, 133.7, 130.9, 130.6, 130.1, 128.5, 128.2, 127.8, 126.8, 124.1, 123.4, 114.2, 113.8, 64.5, 55.2, 43.6, 34.0, 30.2. HRMS (ESI): calcd *m/z* for $C_{25}H_{23}N_2O_2S$: 415.1475 $[M+H]^+$; found: 415.1482. HPLC (YMC CHIRAL ART Cellulose-SB, eluent: *n*-hexane:*i*-PrOH = 4:1, 0.5 mL/min, 10 °C) retention times: t_{minor} = 30.2 min, t_{major} = 25.6 min.

Compound **3m** (***tert*-butyl (+)-(2-((1-cyano-2-(4-methoxybenzyl)-3-oxoisoindolin-1-yl)thio)ethyl)carbamate**):

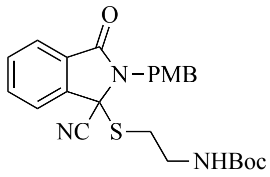

3m

Prepared from **1a** and **6f** according to the general procedure and obtained as a colorless oil (44.0 mg, 0.097 mmol, 97 %, e.r. = 98:2). R_f (heptanes/EtOAc = 2/1) = 0.19. $[\alpha]_D^{24}$ (c = 2.00, $CHCl_3$) = +9.6°; ^{1}H NMR (300 MHz, δ, $CDCl_3$, 298 K): 7.90 (d, *J* = 7.5 Hz, 1H), 7.80 – 7.71 (m, 2H), 7.65 – 7.60 (m, 1H), 7.49 – 7.45 (m, 2H), 6.90 – 6.85 (m, 2H), 4.93, 4.75 (q, AB, *J* = 15.0 Hz, 2H), 4.40 (br s, 1H), 3.80 (s, 3H), 2.83 (app q, *J* = 6.4 Hz, 2H), 2.09 – 1.93 (m, 2H), 1.41 (s, 9H). ^{13}C NMR (75 MHz, δ, $CDCl_3$, 298 K): 166.5, 159.5, 155.3, 141.2, 133.8, 131.0, 130.6, 130.0, 127.7, 124.2, 123.4, 114.1, 113.9, 79.9, 64.3, 55.2, 43.6, 38.6, 29.6, 28.3. HRMS (ESI): calcd *m/z* for $C_{24}H_{27}N_3NaO_4S$: 476.1614 $[M+Na]^+$; found: 476.1616. HPLC (YMC CHIRAL ART Cellulose-SB, eluent: *n*-hexane:*i*-PrOH = 4:1, 0.5 mL/min, 10 °C) retention times: t_{minor} = 31.1 min, t_{major} = 33.3 min.

Compound **3n** (**methyl (+)-3-((1-cyano-2-(4-methoxybenzyl)-3-oxoisoindolin-1-yl)thio)-propanoate**):

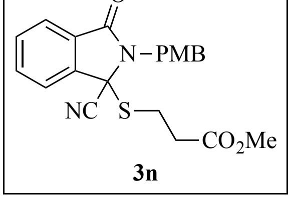

3n

Prepared from **1a** and **6g** according to the general procedure and obtained as a colorless oil (39.2 mg, 0.099 mmol, 99 %, e.r. = 98:2). R_f (heptanes/EtOAc = 2/1) = 0.22. $[\alpha]_D^{24}$ (c = 2.00, $CHCl_3$) = +19.5°; ^{1}H NMR (300 MHz, δ, $CDCl_3$, 298 K): 7.91 (d, *J* = 7.5 Hz, 1H), 7.80 – 7.71 (m, 2H), 7.66 – 7.61 (m, 1H), 7.49 – 7.44 (m, 2H), 7.89 – 6.84 (m, 2H), 4.86, 4.84 (q, AB, *J* = 15.1 Hz, 2H), 3.79 (s, 3H), 3.63 (s, 3H), 2.12 – 2.05 (m, 4H). ^{13}C NMR (75 MHz, δ, $CDCl_3$, 298 K): 171.1, 166.5, 159.4, 141.1, 133.8, 131.0, 130.6, 130.1, 127.6, 124.2, 123.4, 114.0, 113.8, 64.5, 55.2, 52.0, 43.6, 32.5, 23.7. HRMS (ESI): calcd *m/z* for $C_{21}H_{20}N_2NaO_4S$: 419.1036 $[M+Na]^+$;

found: 419.1041. HPLC (YMC CHIRAL ART Cellulose-SB, eluent: *n*-hexane:*i*-PrOH = 4:1, 0.5 mL/min, 10 °C) retention times: t_{minor} = 39.6 min, t_{major} = 48.8 min.

Compound **3o ((-)-(1-cyano-2-(4-methoxybenzyl)-3-oxoisoindolin-1-yl) *O,O*-diethyl phosphorothioate**): Prepared from **1a** and **6h** according to the general procedure and obtained as a colorless oil (26.6 mg, 0.059 mmol, 59 %, e.r. = 52:48). R_f (heptanes/EtOAc = 1/1) = 0.17. $[\alpha]_D^{24}$ (c = 0.9, $CHCl_3$) = -3.6°; ^{1}H NMR (300 MHz, δ, $CDCl_3$, 298 K): 7.93 – 7.89 (m, 2H), 7.75 – 7.61 (m, 2H), 7.46 – 7.43 (m, 2H), 6.90 – 6.85 (m, 2H), 5.25 (d, *J* = 15.2 Hz, 1H), 4.61 (d, *J* = 15.2 Hz, 1H), 4.12 – 3.81 (m, 4H), 3.79 (s, 3H), 1.22 (app q, *J* = 6.9 Hz, 6H). ^{13}C NMR (75 MHz, δ, $CDCl_3$, 298 K): 166.4, 159.5, 141.4 (d, J_{CP} = 2.2 Hz), 133.1, 131.1, 130.6, 130.0, 127.2, 124.2, 123.8, 113.9, 113.6 (d, J_{CP} = 12.1 Hz), 64.9 (d, J_{CP} = 7.7 Hz), 64.8 (d, J_{CP} = 6.6 Hz), 64.2 (d, J_{CP} = 3.9 Hz), 55.2, 44.0, 15.95 (d, J_{CP} = 6.6 Hz), 15.86 (d, J_{CP} = 6.6 Hz). ^{31}P NMR (202 MHz, δ, $CDCl_3$, 298 K): 16.59 – 16.42 (m, 1P). HRMS (ESI): calcd *m/z* for $C_{21}H_{24}N_2O_5PS$: 469.0958 $[M+Na]^+$; found: 469.0961. HPLC (YMC CHIRAL ART Cellulose-SB, eluent: *n*-hexane:*i*-PrOH = 4:1, 0.5 mL/min, 10 °C) retention times: t_{minor} = 40.2 min, t_{major} = 47.8 min.

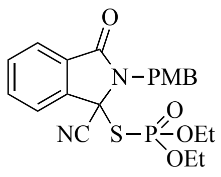

3o

Compound **3p ((+)-1-(benzylthio)-5,6-dimethoxy-2-(4-methoxybenzyl)-3-oxoisoindoline-1-carbonitrile**): Prepared from **1p** and **6a** according to the general procedure and obtained as a light-yellow oil (35.4 mg, 0.076 mmol, 76 %, e.r. = 90:10). R_f (heptanes/EtOAc = 2/1) = 0.50. $[\alpha]_D^{24}$ (c = 1.00, $CHCl_3$) = +17.1°; ^{1}H NMR (300 MHz, δ, $CDCl_3$, 298 K): 7.49 – 7.44 (m, 2H), 7.32 (s, 1H), 7.17 – 7.12 (m, 3H), 7.01 (s, 1H), 6.90 – 6.85 (m, 2H), 6.81 – 6.78 (m, 2H), 4.79, 4.69 (q, AB, *J* = 15.1 Hz, 2H), 3.99 (s, 3H), 3.90 (s, 3H), 3.80 (s, 3H), 3.13, 2.99 (q, AB, *J* = 12.9 Hz, 2H). ^{13}C NMR (75 MHz, δ, $CDCl_3$, 298 K): 166.8, 159.4, 154.1, 151.7, 134.8, 134.1, 130.5, 128.7, 128.4, 128.1, 127.5, 122.8, 114.3, 113.8, 104.9, 64.7, 56.5, 55.2, 53.4, 43.6, 33.6. HRMS (ESI): calcd *m/z* for $C_{26}H_{25}N_2O_4S$: 461.1530 $[M+H]^+$; found: 461.1540. HPLC (YMC CHIRAL ART Cellulose-SB, eluent: *n*-hexane:*i*-PrOH = 4:1, 0.5 mL/min, 10 °C) retention times: t_{minor} = 43.4 min, t_{major} = 46.4 min.

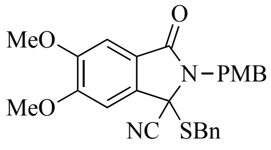

3p

Compound **3q ((+)-1-(benzylthio)-6-fluoro-2-(4-methoxybenzyl)-3-oxoisoindoline-1-carbonitrile**): Prepared from **1q** and **6a** according to the general procedure and obtained as a colorless oil (41.0 mg, 0.098 mmol, 98 %, e.r. = 98.5:1.5). R_f (heptanes/EtOAc = 2/1) = 0.42. $[\alpha]_D^{24}$ (c = 2.00, $CHCl_3$) = +13.2°; ^{1}H NMR (300 MHz, δ, $CDCl_3$, 298 K): 7.89 (dd, *J* = 8.3, 4.8 Hz, 1H), 7.49 – 7.45 (m, 2H), 7.36 (dd,

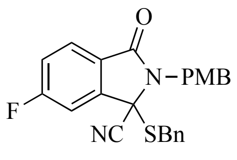

3q

J = 7.5, 2.2 Hz, 1H), 7.31 – 7.25 (m, 1H), 7.21 – 7.17 (m, 3H), 6.91 – 6.86 (m, 2H), 6.83 (dd, J = 6.6, 2.8 Hz, 2H), 4.80, 4.70 (q, AB, J = 15.0 Hz, 2H), 3.80 (s, 3H), 3.13, 3.07 (q, AB, J = 12.7 Hz, 2H). ^{13}C NMR (75 MHz, δ, $CDCl_3$, 298 K): 166.0 (d, J_{CF} = 256.4 Hz), 165.5, 159.5, 143.3 (d, J_{CF} = 9.9 Hz), 134.0, 130.7, 128.7, 163.6 (d, J_{CF} = 9.9 Hz), 127.8, 127.6, 126.24 (d, J_{CF} = 9.4 Hz), 126.23 (d, J_{CF} = 2.8 Hz), 118.9 (d, J_{CF} = 23.7 Hz), 113.9, 113.6, 111.1 (d, J_{CF} = 25.9 Hz), 64.5 (d, J_{CF} = 2.8 Hz), 55.2, 43.8, 33.9. ^{19}F (471 MHz, δ, $CDCl_3$, 298 K). -102.68 to -102.73 (m, 1F). HRMS (ESI): calcd *m/z* for $C_{24}H_{19}FN_2O_2S$: 419.1224 $[M+H]^+$; found: 419.1232. HPLC (YMC CHIRAL ART Cellulose-SB, eluent: *n*-hexane:*i*-PrOH = 4:1, 0.5 mL/min, 10 °C) retention times: t_{minor} = 38.7 min, t_{major} = 35.2 min.

Compound **3r** (**(+)-1-(benzylthio)-5-chloro-2-(4-methoxybenzyl)-3-oxoisoindoline-1-carbonitrile**):

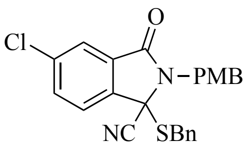

3r

Prepared from **1r** and **6a** according to the general procedure and obtained as a colorless oil (36.6 mg, 0.084 mmol, 84 %, e.r. = 96.5:3.5). R_f (heptanes/EtOAc = 2/1) = 0.53. $[\alpha]_D^{24}$ (c = 1.00, $CHCl_3$) = +17.3°; ^{1}H NMR (300 MHz, δ, $CDCl_3$, 298 K): 7.86 (d, J = 1.7 Hz, 1H), 7.61 – 7.54 (m, 2H), 7.49 – 7.44 (m, 2H), 7.19 – 7.14 (m, 3H), 6.91 – 6.86 (m, 2H), 6.83 – 6.80 (m, 2H), 4.79, 4.69 (q, AB, J = 15.0 Hz, 2H), 3.80 (s, 3H), 3.12, 3.04 (q, AB, J = 12.8 Hz, 2H). ^{13}C NMR (75 MHz, δ, $CDCl_3$, 298 K): 166.2, 159.6, 139.0, 137.4, 134.1, 133.7, 131.9, 130.7, 128.7, 128.6, 127.7, 127.5, 124.7, 124.2, 113.9, 113.6, 64.7, 55.2, 43.8, 33.9. HRMS (ESI): calcd *m/z* for $C_{24}H_{20}ClN_2O_2S$: 435.0929 $[M+H]^+$; found: 435.0938. HPLC (YMC CHIRAL ART Cellulose-SB, eluent: *n*-hexane:*i*-PrOH = 4:1, 0.5 mL/min, 10 °C) retention times: t_{minor} = 33.3 min, t_{major} = 36.9 min.

Compound **3s** (**(+)-1-(benzylthio)-5-bromo-2-(4-methoxybenzyl)-3-oxoisoindoline-1-carbonitrile**):

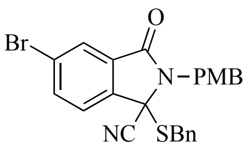

3s

Prepared from **1s** and **6a** according to the general procedure and obtained as a light-yellow oil (38.8 mg, 0.081 mmol, 81 %, e.r. = 95:5). R_f (heptanes/EtOAc = 2/1) = 0.50. $[\alpha]_D^{24}$ (c = 1.00, $CHCl_3$) = +19.1°; ^{1}H NMR (300 MHz, δ, $CDCl_3$, 298 K): 8.02 (d, J = 1.7 Hz, 1H), 7.71 (dd, J = 8.2, 1.8 Hz, 1H), 7.52 (d, J = 8.2 Hz, 1H), 7.49 – 7.44 (m, 2H), 7.21 – 7.14 (m, 3H), 6.90 – 6.86 (m, 2H), 6.82 – 6.79 (m, 2H), 4.79, 4.69 (q, AB, J = 15.0 Hz, 2H), 3.80 (s, 3H), 3.12, 3.04 (q, AB, J = 12.8 Hz, 2H). ^{13}C NMR (75 MHz, δ, $CDCl_3$, 298 K): 165.1, 159.6, 139.5, 136.6, 134.1, 132.0, 130.7, 128.7, 128.6, 127.7, 127.5, 127.2, 125.3, 125.0, 113.9, 113.5, 64.8, 55.3, 43.8, 34.0. HRMS (ESI): calcd *m/z* for $C_{24}H_{20}BrN_2O_2S$: 479.0423 $[M+H]^+$; found: 479.0432. HPLC (YMC CHIRAL ART Cellulose-SB, eluent: *n*-hexane:*i*-PrOH = 4:1, 0.5 mL/min, 10 °C) retention times: t_{minor} = 34.7 min, t_{major} = 39.3 min.

3.3 Synthesis of Fluoro-Isoindolinones 4

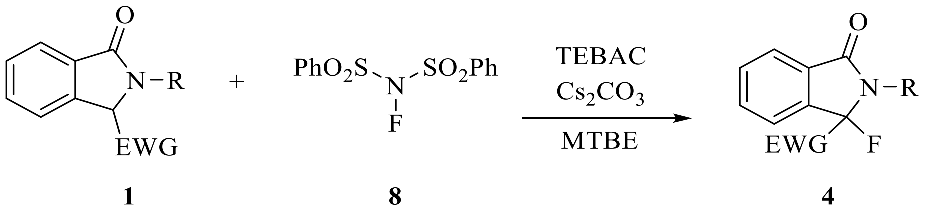

Isoindolinones **1** (0.1 mmol), NFSI **8** (0.2 mmol), TEBAC (0.01 mmol) and Cs_2CO_3 (0.2 mmol) were mixed together in MTBE (2 mL) and stirred at room temperature overnight. After completion of the reaction (as indicated by TLC), the suspension was diluted with Et_2O (5 mL) and filtered through a pad of Celite® (washed with Et_2O). The solvent was evaporated and the crude mixture was purified by column chromatography (silica gel, heptanes/EtOAc) to give the respective fluoro-isoindolinones **4** as light-yellow crystals.

Compound **4a** (**1-fluoro-2-(4-methoxybenzyl)-3-oxoisoindoline-1-carbonitrile**): Prepared from **1a** and **8** according to the general procedure and obtained as light yellow crystals (23.1 mg, 0.078 mmol, 78 %). R_f (heptanes/EtOAc = 2/1) = 0.50. m.p. = 143 – 145 °C. ^{1}H NMR (700 MHz, δ, $CDCl_3$, 298 K): 7.96 (d, J = 7.5 Hz, 1H), 7.85 (d, J = 7.6 Hz, 1H), 7.80 (t, J = 7.5, 7.5 Hz, 1H), 7.75 (t, J = 7.5, 7.5 Hz, 1H), 7.42 (d, J = 9.0 Hz, 2H), 7.04 (d, J = 8.9 Hz, 2H), 3.86 (s, 3H). ^{13}C NMR (176 MHz, δ, $CDCl_3$, 298 K): 165.9, 165.9, 160.3, 137.8, 137.6, 134.5, 134.4, 133.0, 133.0, 129.6, 129.1, 125.4, 125.0, 123.6, 115.2, 113.2, 112.9, 95.0, 93.8, 55.6. ^{19}F NMR (282 MHz, δ, $CDCl_3$, 298 K): -105.0. HRMS (ESI): calcd m/z for $C_{17}H_{13}FN_2O_2$: 297.1034 $[M+H]^+$; found: 297.1040.

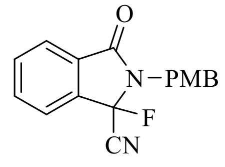

4a

Compound **4b** (**1-fluoro-2-benzyl-3-oxoisoindoline-1-carbonitrile**): Prepared from **1y** and **8** according to the general procedure and obtained as a colourless oil (23.5 mg, 0.075 mmol, 75 %). R_f (heptanes/EtOAc = 2/1) = 0.47. ^{1}H NMR (700 MHz, δ, $CDCl_3$, 298 K): 7.90 – 7.86 (m, 1H), 7.64 – 7.60 (m, 2H), 7.56 – 7.52 (m, 1H), 7.37 (d, J = 7.5 Hz, 2H), 7.30 (t, J = 7.6, 7.6 Hz, 2H), 7.26 – 7.24 (m, 1H), 4.86 (d, J = 15.5 Hz, 1H), 4.63 (d, J = 15.5 Hz, 1H), 3.97 – 3.90 (m, 1H), 3.80 – 3.72 (m, 1H), 1.00 (t, J = 7.1, 7.1 Hz, 3H).^{13}C NMR (75 MHz, δ, $CDCl_3$, 298 K): 171.2, 168.2, 143.8, 136.6, 132.7, 131.5, 130.4, 129.2, 128.4, 127.7, 123.9, 121.6, 87.1, 63.6, 42.5, 13.4. ^{19}F NMR (282 MHz, δ, $CDCl_3$, 298 K): -135.0. HRMS (ESI): calcd m/z for $C_{18}H_{16}FNO_3$: 314.1187 $[M+H]^+$; found: 314.1194.

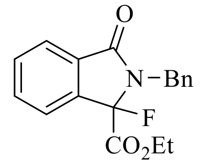

4b

4. Further Transformations

4.1 H_2O_2-Promoted Hydrolysis of the SCF_3-Cyano-Isoindolinones 2a and 2q

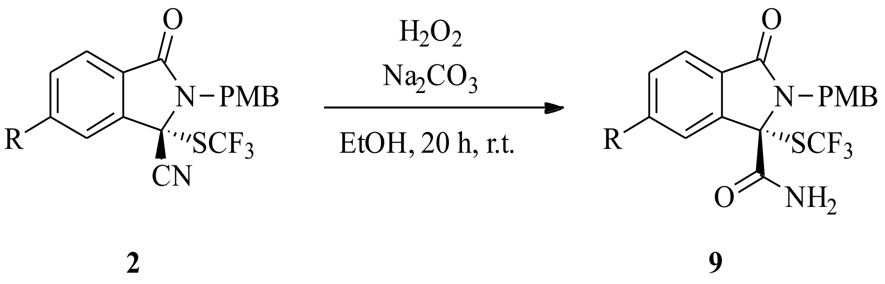

Products **9** were synthesized in analogy to a recent literature procedure[14]: A suspension of **2** (0.1 mmol) and Na_2CO_3 (5 mmol) in EtOH (2 mL, 0.05 M) was cooled to 0° C. An aqueous solution of H_2O_2 (30 % w/w) was added dropwise over a period of 10 min and the suspension was stirred at 0° C for another 20 min. The mixtures was then allowed to warm to room temperature and stirred overnight. After completion of the reaction (as indicated by TLC), water (20 mL) and DCM (20 mL) were added and the phases separated. The aqueous phase was extracted two more times with DCM (20 mL) and the combined organic layers were dried over Na_2SO_4. After filtration and evaporation of the solvent, the crude products were purified by silica gel column chromatography (heptanes/EtOAc = 1/1) and isolated as clear oils.

Compound **9a** (**2-(4-methoxybenzyl)-3-oxo-1-((trifluoromethyl)thio)isoindoline-1-carboxamide**):

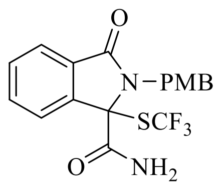
9a

Prepared from **2a** following the method described above and obtained as a colourless oil (29.3 mg, 0.074 mmol, 74 %). R_f (heptanes/EtOAc = 2/1) = 0.35. m.p. = 166 – 168 °C. ^{1}H NMR (300 MHz, δ, $CDCl_3$, 298 K): 7.89 (d, J = 7.3 Hz, 1H), 7.70 – 7.56 (m, 3H), 7.43 (d, J = 8.4 Hz, 2H), 6.83 (d, J = 8.5 Hz, 2H), 5.28 (d, J = 15.0 Hz, 1H), 4.91 (s, 2H), 4.44 (d, J = 15.0 Hz, 1H), 3.78 (s, 3H). ^{13}C NMR (75 MHz, δ, $CDCl_3$, 298 K): 168.1, 167.8, 159.5, 143.3, 133.6, 131.1, 130.8, 129.4, 128.8 (q, CF_3, J_{CF} = 308.4 Hz), 128.4, 124.4, 123.6, 114.2, 81.5, 55.4, 43.9. ^{19}F NMR (471 MHz, δ, $CDCl_3$, 298 K): -39.2. HRMS (ESI): calcd m/z for $C_{18}H_{15}F_3N_2O_3S$: 397.0828 $[M+H]^+$; found: 397.0830.

[14] P. V. Balaji, L. Brewitz, N. Kumagai, M. Shibasaki, *Angew. Chem.*, **2019**, 58, 9, 2644-2648.

Compound **9b** (**(*S*)-6-fluoro-2-(4-methoxybenzyl)-3-oxo-1-((trifluoromethyl)thio)isoindoline-1-carbonitrile**): Prepared from **2q** following the method described above and obtained as a colourless oil (26.9 mg, 0.065 mmol, 65 %, e.r. = 96:4). R_f (heptanes/EtOAc = 1/1) = 0.45. $[\alpha]_D^{22}$ (c = 0.40, $CHCl_3$) = +29.5°; ^1H NMR (300 MHz, δ, $CDCl_3$, 298 K): 7.89 (dd, J = 8.1, 4.6 Hz, 1H), 7.45 – 7.37 (m, 3H), 7.33 – 7.27 (m, 1H), 6.84 (d, J = 8.2 Hz, 2H), 5.28 (d, J = 12.4 Hz, 1H), 4.87 (s, 2H), 4.41 (d, J = 15.2 Hz, 1H), 3.78 (s, 3H). ^{13}C NMR (75 MHz, δ, $CDCl_3$, 298 K): 167.2, 167.1, 164.4, 159.7, 131.1, 128.3, 126.7, 126.6, 124.8 (q, CF_3, J_{CF} = 310.6 Hz), 119.0, 118.7, 118.7, 114.4, 111.3, 111.0, 77.6, 77.2, 76.7, 55.4, 44.2, 29.9. ^{19}F NMR (471 MHz, δ, $CDCl_3$, 298 K): -39.2 (s, 3F), -102.9 to -103.0 (m, 1F). HRMS (ESI): calcd m/z for $C_{18}H_{14}F_4N_2O_3S$: 437.0553 $[M+Na]^+$; found: 437.0567. HPLC (YMC CHIRAL ART Amylose-SA, eluent: hexane:*i*-PrOH = 10:1, 0.5 mL/min, 10 °C) retention times: t_{minor} = 28.7 min, t_{major} = 25.4 min.

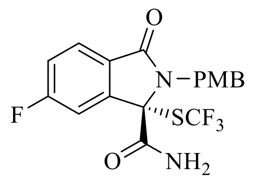

9b

4.2 Houben–Hoesch Cyclization of the SBn-Cyano-Isoindolinone 3c

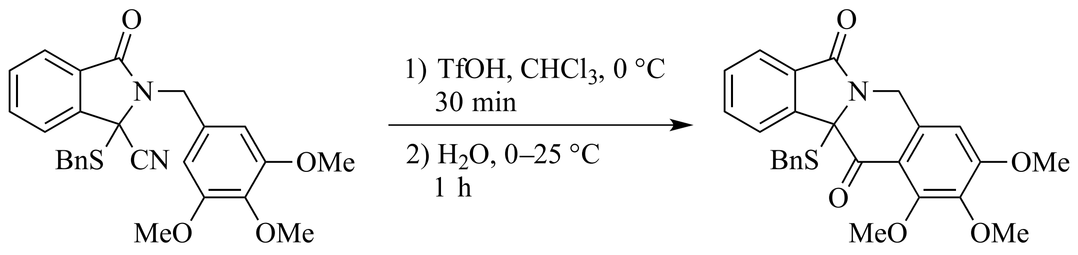

3c **10**

10 was prepared according to a modified literature procedure [8b]: Trifluoromethanesulfonic acid (0.2 mL) was added dropwise over 5 min at 0 °C to a solution of **3c** (36 mg, 0.078 mmol) in dry $CHCl_3$ (1.0 mL) under Ar. Then the reaction mixture was left to stir at 0 °C for 30 min. After the complete conversion was revealed by TLC (heptanes/EtOAc = 1/2), the reaction was carefully quenched with H_2O (1 mL) and left to stir 1 h at 25 °C. The mixture was diluted with $CHCl_3$ and water, phases were separated, the organic phase was washed with brine, dried over anhydrous Na_2SO_4, filtered, and evaporated *in vacuo*. The crude residue was purified by filtration through a silica plug (100 % EtOAc).

Compound **10** (**(-)-11b-(benzylthio)-1,2,3-trimethoxyisoindolo[2,1-*b*]isoquinoline-7,12(5*H*,11b*H*)-dione**): Prepared following the method described above and obtained as viscous yellow oil (33.1 mg, 0.072 mmol, 92 %, e.r. = 98:2). R_f (heptanes/EtOAc = 1/2) = 0.59. $[\alpha]_D^{22}$ (c = 1.0, $CHCl_3$) = -78.2°; ^{1}H NMR (300 MHz, δ, $CDCl_3$, 298 K): 8.14 (d, *J* = 7.7 Hz, , 1H), 7.85 (d, *J* = 7.5 Hz, 2H), 7.66 (td, *J* = 7.5, 1.1 Hz, 1H), 7.53 (td, *J* = 7.5, 1.1 Hz, 1H), 7.21 – 7.17 (m, 3H), 7.01 – 6.98 (m, 3H), 6.60 (s, 1H), 5.19 (d, *J* = 17.1 Hz, 1H), 4.35 (d, *J* = 16.9 Hz, 1H), 3.95 (s, 3H), 3.87 (s, 3H), 3.83 (s, 3H), 3.27, 3.21 (q, AB, *J* = 13.2 Hz, 2H). ^{13}C NMR (75 MHz, δ, $CDCl_3$, 298 K): 184.3, 166.0, 158.6, 156.5, 142.6, 142.4, 138.6, 136.5, 132.8, 131.0, 129.5, 128.7, 128.4, 127.2, 126.1, 123.1, 117.0, 104.5, 75.7, 61.5, 61.1, 56.3, 38.6, 33.6. HRMS (ESI): calcd *m/z* for $C_{26}H_{23}NO_5S$: 462.1370 $[M+H]^+$; found: 462.1371. HPLC (Chiralpak AD-H, eluent: hexane:*i*-PrOH = 3:1, 0.5 mL/min, 10 °C) retention times: t_{minor} = 61.3 min, t_{major} = 67.1 min.

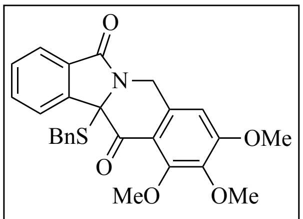

10

4.3 *N*-Boc Deprotection of the SCH_2CH_2NHBoc-Cyano-Isoindolinone 3m

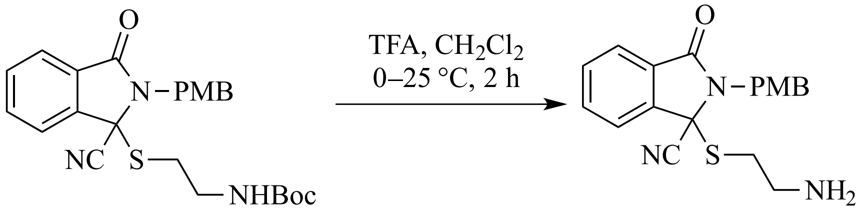

3m **11**

Trifluoroacetic acid (75 μL) was added dropwise over 5 min at 0 °C to a solution of **3m** (30 mg, 0.066 mmol) in dry dichloromethane (0.3 mL) under Ar. Then the reaction mixture was allowed to warm to 25 °C and left to stir for 2 h (based on the reaction conversion determined by TLC). Then the mixture was cooled to 0 °C before carefully quenching with H_2O (1 mL) and alkalinization with saturated aqueous solution of $NaHCO_3$ (10 mL). The mixture was further diluted with dichloromethane, phases were separated, the organic phase was washed with brine, dried with anhydrous Na_2SO_4, filtered, and evaporated *in vacuo*.

Compound **11** (**(+)-1-((2-aminoethyl)thio)-2-(4-methoxybenzyl)-3-oxoisoindoline-1-carbonitrile**):

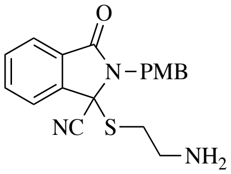

11

Prepared following the method described above and obtained as a yellowish oil (18.1 mg, 0.051 mmol, 77 %). R_f (CH_2Cl_2/MeOH = 15/1) = 0.20. (when starting from enantioenriched (+)-**3m**, (+)-**11** was obtained, $[\alpha]_D^{22}$ (c = 1.0, $CHCl_3$) = +23.5°); ^1H NMR (300 MHz, δ, $CDCl_3$, 298 K): 7.90 (d, *J* = 7.5 Hz, 1H), 7.80 – 7.70 (m, 2H), 7.64 – 7.59 (m, 1H), 7.50 – 7.45 (m, 2H), 6.89 – 6.84 (m, 2H), 4.95, 4.77 (q, AB, *J* = 15.0 Hz, 2H), 3.79 (s, 3H), 2.51 – 2.36 (m, 2H), 2.03 – 1.86 (m, 2H), 1.10 (br s, 2H). ^{13}C NMR (75 MHz, δ, $CDCl_3$, 298 K): 166.6, 159.4, 141.4, 133.7, 130.9, 130.6, 130.1, 127.7, 124.2, 123.4, 114.2, 113.8, 64.3, 55.2, 43.6, 40.2, 33.0. HRMS (ESI): calcd *m/z* for $C_{19}H_{19}N_3O_2S$: 354.1271 $[M+H]^+$; found: 354.1278.

4.4 Methyl-Ester Hydrolysis of the $SCH_2CH_2CO_2Me$-Cyano-Isoindolinone 3n

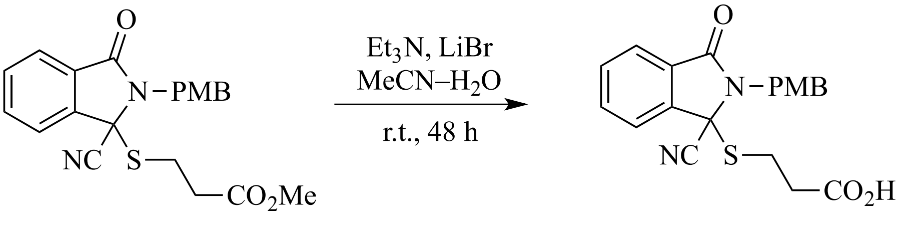

3n **12**

Compound **12** was prepared following a literature procedure[15]. To a solution of **3n** (30 mg, 0.076 mmol) in MeCN (0.3 mL) was added H_2O (20 μL), triethylamine (3 equiv, 32 μL) and LiBr (10 equiv, 70 mg). The resulting mixture was left to stir 48 h (the reaction conversion was determined by TLC). Then the reaction mixture was diluted with H_2O (1 mL) and extracted with EtOAc (3 × 1 mL). The water phase was separated, acidified with 2 M aqueous HCl and extracted with EtOAc (3 × 1 mL). The combined organic phase was washed with brine, dried with anhydrous Na_2SO_4, filtered, and evaporated *in vacuo*. The crude residue was purified by silica gel column chromatography (dichloromethane/MeOH = 15/1).

Compound **12** (**3-((1-cyano-2-(4-methoxybenzyl)-3-oxoisoindolin-1-yl)thio)propanoic acid**):

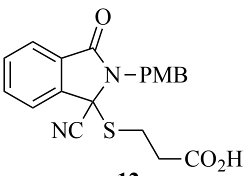

12

Prepared following the method described above and obtained as white waxy solid (13.8 mg, 0.036 mmol, 47%). R_f (CH_2Cl_2/MeOH = 15/1) = 0.20. (when starting from enantioenriched (+)-**3n**, (-)-**12** was obtained, $[\alpha]_D^{22}$ (c = 0.1, $CHCl_3$) = -27.2°); ^{1}H NMR (300 MHz, δ, DMSO-d_6, 298 K): 7.94 – 7.90 (m, 1H), 7.86 – 7.78 (m, 2H), 7.72 – 7.67 (m, 1H), 7.40 – 7.37 (m, 2H), 6.90 – 6.87 (m, 2H), 4.79, 4.67 (q, AB, J = 15.6 Hz, 2H), 3.72 (s, 3H), 2.79 – 2.61 (m, 2H), 1.67 – 1.57 (m, 1H), 1.43 – 1.30 (m, 1H). ^{13}C NMR (75 MHz, δ, DMSO-d_6, 298 K): 172.0, 166.7, 158.8, 140.7, 133.7, 130.8, 130.1, 129.7, 128.3, 123.7, 122.9, 117.4, 113.8, 60.2, 55.1, 42.6, 29.8, 26.4. HRMS (ESI): calcd *m/z* for $C_{20}H_{18}N_2O_4S$: 383.1060 $[M+H]^+$; found: 383.1063.

[15] S. Mattsson, M. Dahlström, S. Karlsson, *Tetrahedron Lett.* **2007**, 48, 2497-2499.

4.5 Synthesis of Bifunctional Guanidine Phase-Transfer Catalyst B3

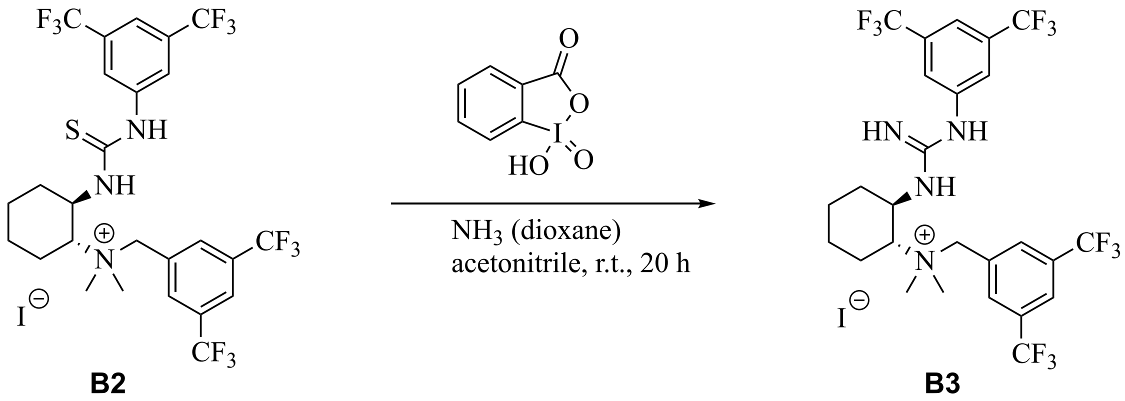

46.2 mg of 2-iodoxybenzoic acid (IBX; 0.165 mmol, 3.3 equiv.) were added in portions to a solution of 38.4 mg of bifunctional thiourea **B2** (0.050 mmol, 1.0 equiv.) in 0.8 mL dry acetonitrile at room temperature (Ar atmosphere). After stirring for 10 min 3.5 mL of a solution of NH_3 in dioxane (0.4 M, 1.5 mmol, 30 equiv.) were added dropwise and the resulting reaction mixture was stirred at room temperature for 20 h. 8 mL of saturated aq. $NaHCO_3$ were added and the mixture was extracted with ethyl acetate (3 × 15 mL). The organic phases were combined and washed with saturated aq. $NaHCO_3$ (20 mL), dried over Na_2SO_4, filtered and evaporated. The crude mixture was purified by preparative TLC (silica gel, DCM/MeOH = 7/1) to obtain 5.1 mg (0.007 mmol) of product **B3** in a yield of 14 %. R_f (DCM/MeOH = 7/1) = 0.61. ^{1}H NMR (300 MHz, δ, $CDCl_3$, 298 K): 8.78 – 8.72 (m, 1H), 8.02 (s, 1H), 7.96 (s, 1H), 7.41 (s, 1H), 7.28 (s, 2H), 5.71 (s, 1H), 5.38 (s, 2H), 4.42 – 4.37 (m, 2H), 3.24 (s, 3H), 3.08 (s, 3H), 2.47 – 2.44 (m, 1H), 2.26 – 2.21 (m, 1H), 2.05 – 1.99 (m, 2H), 1.89 – 1.79 (m, 2H), 1.61 – 1.54 (m, 2H), 1.42 – 1.35 (m, 2H). ^{13}C NMR (125 MHz, δ, $CDCl_3$, 298 K): 152.2, 133.2, 132.9, 130.7, 124.9, 124.6, 123.7, 123.5, 115.1, 78.2, 65.8, 51.5, 50.5, 47.5, 35.4, 29.8, 27.3, 25.3, 24.6. HRMS (ESI): calcd *m/z* for $C_{26}H_{27}F_{12}N_4^+$: 623.2039 $[M]^+$; found: 623.2038.

4.6 Synthesis of Bifunctional Isophorone Diamine-Based Phase-Transfer Catalyst C1

4.6.1 Synthesis of Boc-protected Diamine III

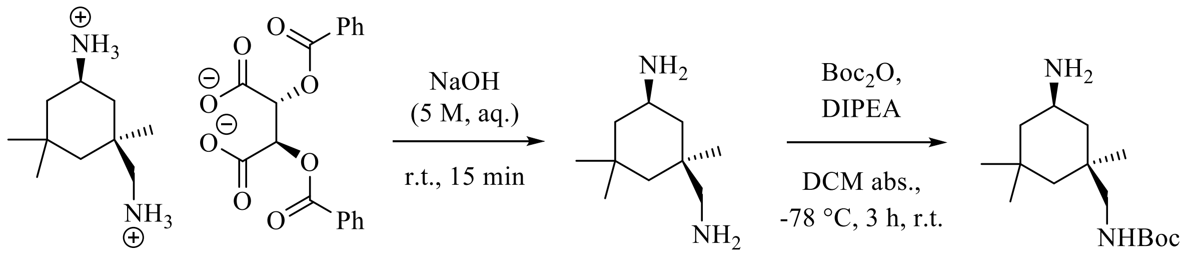

I **II** **III**

300.0 mg of **I** (0.568 mmol, 1 equiv.) were mixed with 1.8 mL aq. NaOH (5 M) and stirred for 15 min until all solid is dissolved. The resulting mixture was extracted with DCM (4 × 5 mL). The organic phase was dried over Na_2SO_4, filtered and carefully evaporated. The quantitatively obtained product **II** was directly subjected to the Boc-protection. 96.7 mg of **II** (0.568 mmol, 1 equiv.) were dissolved in 1.2 mL dry DCM under argon atmosphere. After addition of 193.2 µL DIPEA (1.136 mmol, 2 equiv.) the resulting mixture was stirred for 30 min at room temperature and subsequently cooled to -78 °C. A solution of 130.2 mg Boc_2O (0.596 mmol, 1.05 equiv.) was added dropwise at this temperature and the reaction mixture was stirred for another 3 h at -78 °C and overnight at room temperature. The reaction was quenched using 5 mL H_2O and the pH was adjusted to 10 by addition of aq. NaOH (5 M). The crude mixture was extracted with DCM (3 × 10 mL). The organic phase was dried over Na_2SO_4, filtered and evaporated. The crude product was purified by column chromatography (silica gel, DCM/MeOH = 15/1) to obtain 86.0 mg of product **III** (0.318 mmol) with a yield of 56 %. R_f (DCM/MeOH = 15/1) = 0.08. ^{1}H NMR (300 MHz, δ, $CDCl_3$, 298 K): 5.41 (s, 2H), 4.79 (t, J = 6.2 Hz, 1H), 3.21 (t, J = 11.0 Hz, 1H), 2.84 (d, J = 6.5 Hz, 2H), 1.72 (dd, J_1 = 21.4 Hz, J_2 = 12.6 Hz, 2H), 1.40 (s, 9H), 1.21 – 1.17 (m, 1H), 1.13 – 1.04 (m, 3H), 0.99 (s, 3H), 0.97 (s, 3H), 0.92 (s, 3H).

4.6.2 Reductive Amination

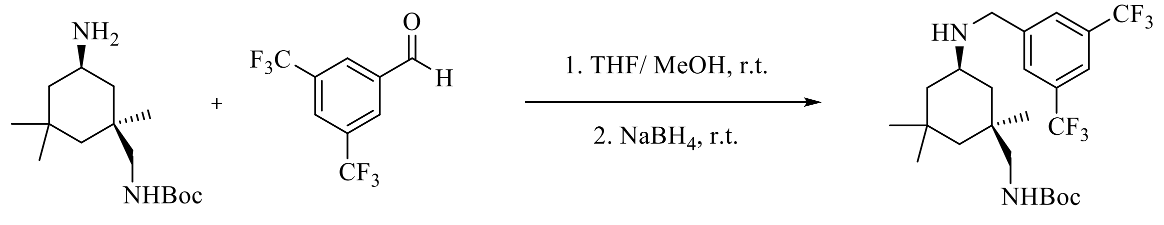

III **IV**

33.5 mg of **III** (0.124 mmol, 1 equiv.) were dissolved in 1 mL THF/MeOH (1/1) under argon atmosphere. 40.8 µL of 3,5-bis(trifluoromethyl)benzaldehyde (0.248 mmol, 2 equiv.) were added at room temperature and the resulting reaction mixture was stirred overnight. After addition of 7.04 mg

$NaBH_4$ (0.186 mmol, 1.5 equiv.) in one portion at 0 °C, the mixture is stirred at room temperature for further 2 h. The reaction was quenched using 10 mL H_2O and extracted with Et_2O (4 × 10 mL). The organic phase was washed with saturated aq. NaCl, dried over Na_2SO_4, filtered and evaporated. The crude product was purified by column chromatography (silica gel, DCM/MeOH = 40/1) to obtain 35.6 mg of product **IV** (0.072 mmol) with a yield of 58 %. R_f (DCM/MeOH = 40/1) = 0.21. ^{1}H NMR (300 MHz, δ, $CDCl_3$, 298 K): 7.81 (s, 2H), 7.75 (s, 1H), 4.62 (t, J = 6.8 Hz, 2H), 3.95 (s, 2H), 2.87 (d, J = 6.5 Hz, 2H), 2.85 – 2.77 (m, 1H), 1.75 – 1.62 (m, 2H), 1.44 (s, 9H), 1.39 (s, 1H), 1.24 – 1.19 (m, 1H), 1.05 (d, J = 13.7 Hz, 1H), 1.00 (s, 3H), 0.99 (s, 3H), 0.94 (s, 3H), 0.91 – 0.83 (m, 2H).

4.6.3 Methylation

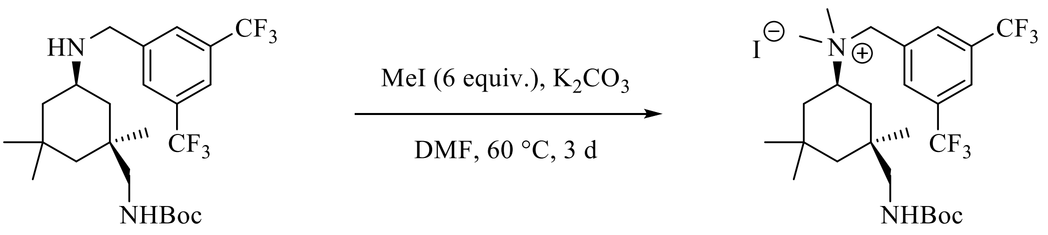

IV **V**

A solution of 35.6 mg of **IV** (0.072 mmol, 1 equiv.) in 0.7 mL DMF was added to 11.9 mg of K_2CO_3 (0.086 mmol, 1.2 equiv.) and set under argon atmosphere. 26.9 µL MeI (0.432 mmol, 6 equiv.) were added and the reaction flask was closed tightly. The reaction mixture was stirred for 3 d at 60 °C before excess MeI was removed over NH_3/ethanolamine/H_2O (1/1/8) using reduced pressure. The crude mixture was extracted with DCM (3 × 20 mL). The organic phase was washed with saturated aq. NaCl (1 × 25 mL), dried over Na_2SO_4, filtered and evaporated to obtain 46.5 mg of product **V** (0.071 mmol) with a yield of 99 %. ^{1}H NMR (300 MHz, δ, $CDCl_3$, 298 K): 7.99 (s, 2H), 7.95 (s, 1H), 5.67 (t, J = 6.4 Hz, 1H), 5.39 (dd, J_1 = 23.4 Hz, J_2 = 13.0 Hz, 2H), 3.79 (t, J = 11.6 Hz, 1H), 3.29 (s, 3H), 3.25 (s, 3H), 2.27 – 2.14 (m, 2H), 2.02 (d, J = 11.2 Hz, 1H), 1.62 (t, J = 12.3 Hz, 1H), 1.40 (s, 9H), 1.25 – 1.20 (m, 3H), 1.14 (s, 1H), 1.06 (s, 3H), 1.04 (s, 3H), 1.02 (s, 3H).

4.6.4 Boc-Deprotection

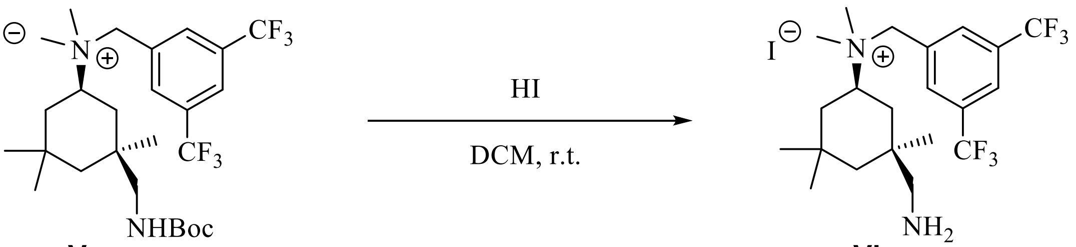

V **VI**

46.5 mg of **V** (0.071 mmol, 1 equiv.) were dissolved in 2 mL of DCM and 0.281 mL of HI (57% in H_2O, 2.130 mmol, 30 equiv.) were added. After stirring at room temperature for 2 h the resulting mixture was

transferred to a separatory funnel using H_2O and DCM. The pH of the aqueous phase was adjusted to 11 using saturated aq. Na_2CO_3 and was subsequently extracted with DCM (3 × 15 mL). The organic phase was dried over Na_2SO_4, filtered and evaporated to obtain 20.0 mg of product **VI** (0.036 mmol) in a yield of 51 %. ^{1}H NMR (300 MHz, δ, $CDCl_3$, 298 K): 8.38 (s, 2H), 8.00 (s, 1H), 5.47 – 5.34 (m, 2H), 4.31 – 4.28 (m, 1H), 3.68 – 3.64 (m, 1H), 3.22 (s, 6H), 2.75 (dd, J_1 = 30.9 Hz, J_2 = 13.3 Hz, 2H), 2.56 (d, J = 9.5 Hz, 1H), 2.25 – 2.22 (m, 1H), 1.99 – 1.85 (m, 3H), 1.68 – 1.54 (m, 3H), 1.21 (s, 3H), 1.16 (s, 3H), 1.08 (s, 3H).

4.6.5 Synthesis of Catalyst C1

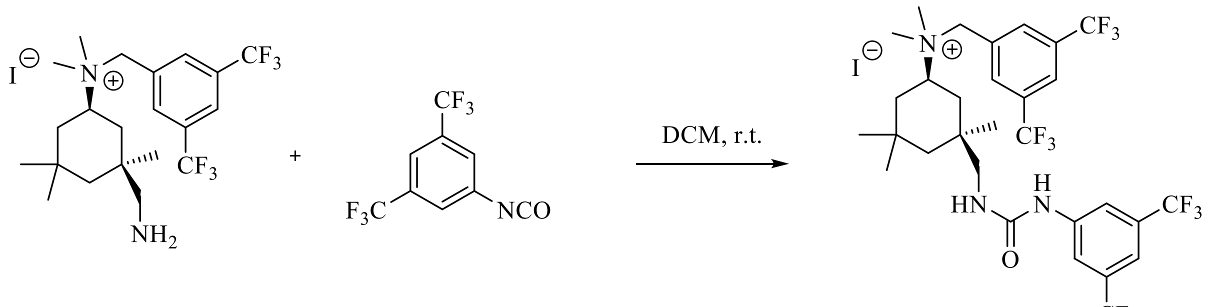

VI **C1**

To a solution of 20.0 mg of **VI** (0.036 mmol, 1 equiv.) in 0.7 mL DCM were added 7.3 µL of 3,5-bis(trifluoromethylphenyl)isocyanate (0.043 mmol, 1.2 equiv.) and the resulting reaction mixture was stirred at room temperature overnight. The solvent was evaporated and the crude product was purified by column chromatography (silica gel, DCM/MeOH = 10/1) to obtain 15.4 mg of product **A14** (0.019 mmol) with a yield of 53 %. R_f (DCM/MeOH = 10/1) = 0.39. ^{1}H NMR (300 MHz, δ, $CDCl_3$, 298 K): 8.87 (s, 1H), 8.26 (s, 1H), 8.01 (s, 1H), 7.97 (s, 2H), 7.37 (s, 1H), 6.91 – 6.88 (m, 1H), 5.24 (q, J = 12.9 Hz, 2H), 3.84 (t, J = 11.8 Hz, 1H), 3.39 – 3.32 (m, 1H), 3.21 (s, 3H), 3.17 (s, 3H), 2.95 (dd, J_1 = 13.8 Hz, J_2 = 3.8 Hz, 1H), 2.53 (d, J = 11.5 Hz, 1H), 1.94 (d, J = 11.5 Hz, 1H), 1.75 (s, 1H), 1.64 – 1.19 (m, 4H), 1.10 (s, 3H), 1.07 (s, 6H). ^{13}C NMR (75 MHz, δ, $CDCl_3$, 298 K): 155.9, 141.8, 133.5, 132.1, 129.7, 125.3, 124.4, 121.7, 117.8, 70.2, 63.5, 52.8, 48.3, 47.5, 46.7, 39.2, 38.1, 35.8, 35.1, 33.5, 27.8, 23.1. HRMS (ESI): calcd *m/z* for $C_{30}H_{34}F_{12}N_3O^+$: 680.2505 $[M]^+$; found: 680.2507.

5. Copies of Product NMR Spectra

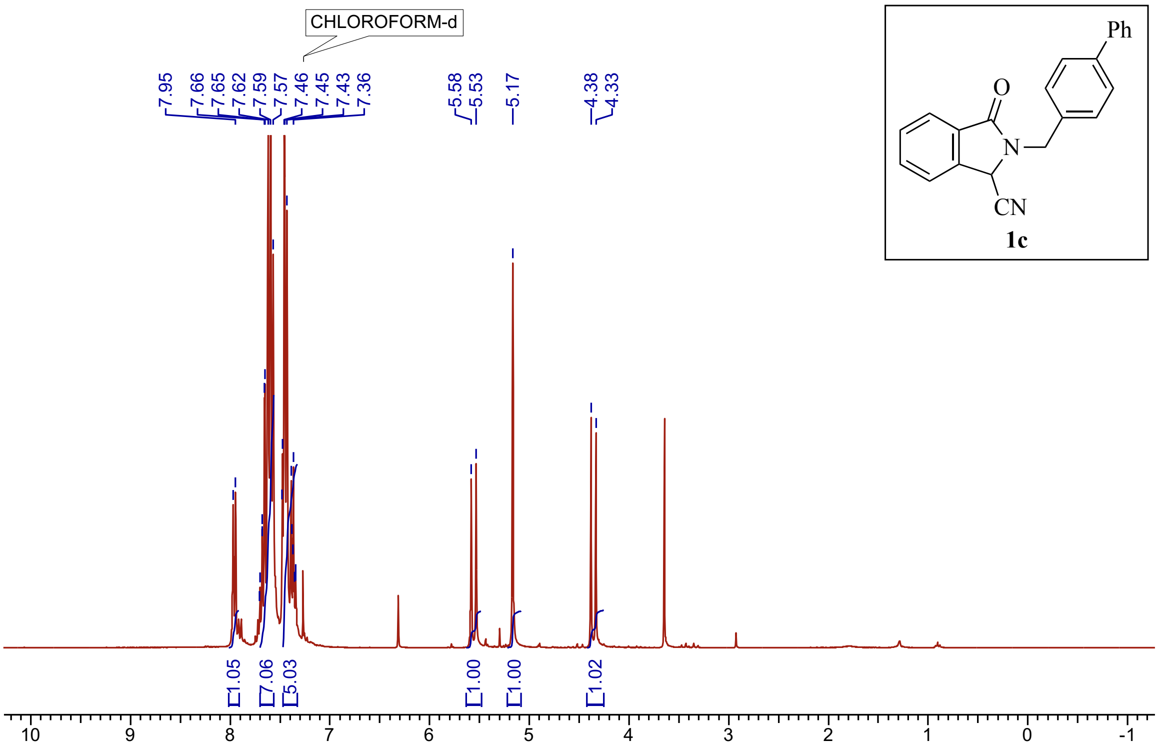

^{1}H-NMR (300 MHz, $CDCl_3$)

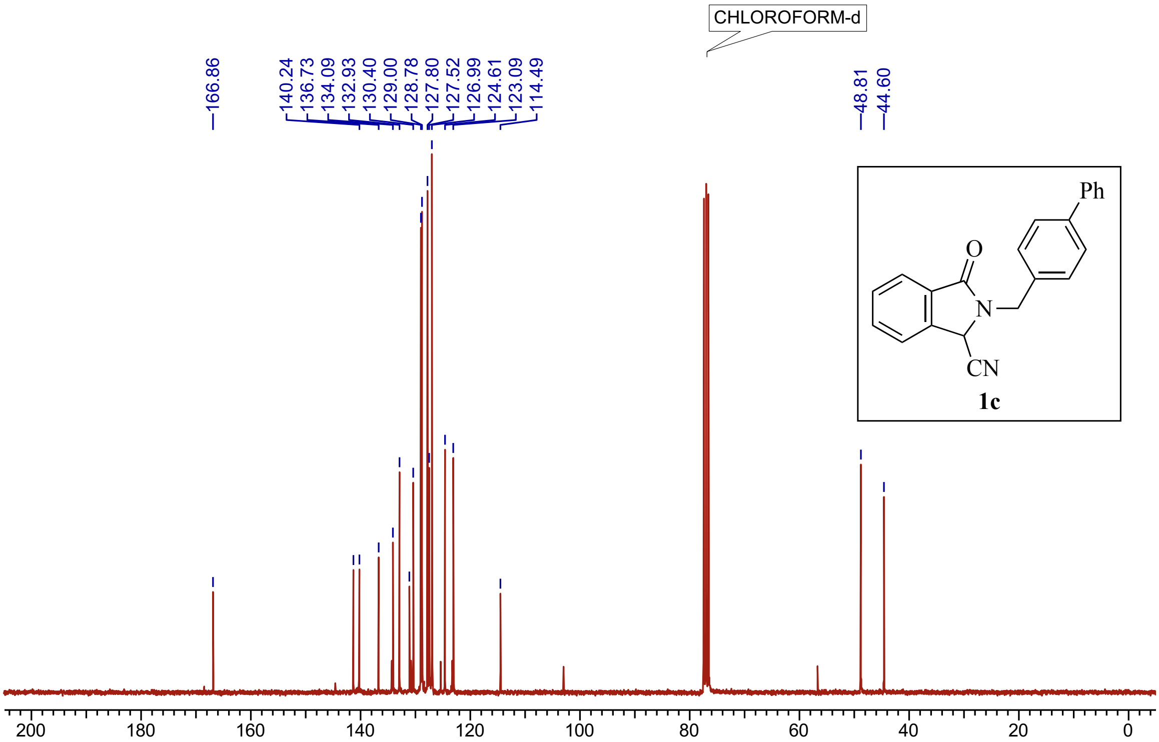

^{13}C-NMR (75 MHz, $CDCl_3$)

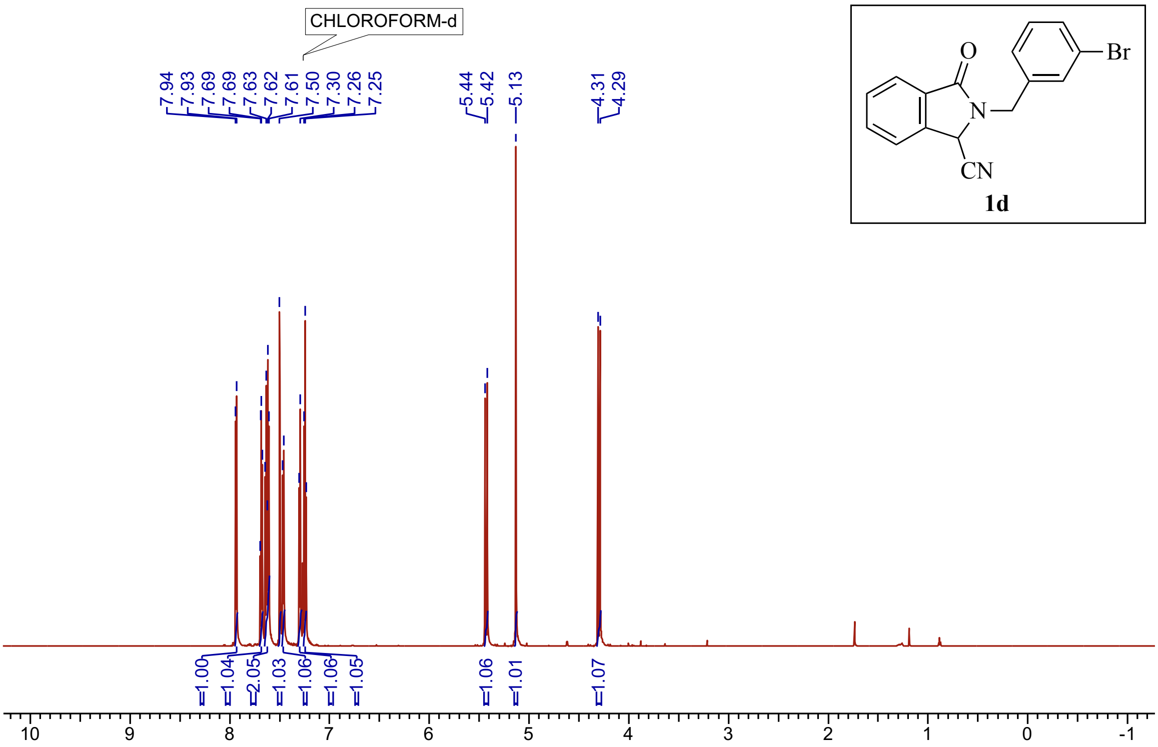

^{1}H-NMR (700 MHz, $CDCl_3$)

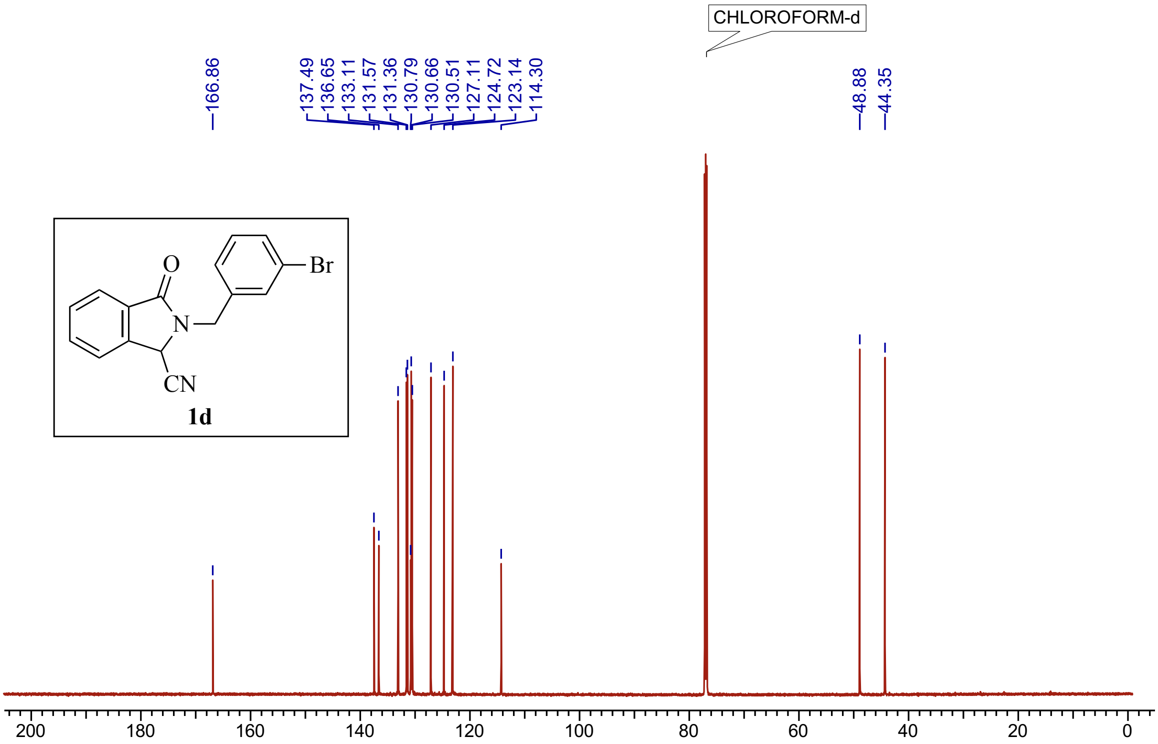

^{13}C-NMR (176 MHz, $CDCl_3$)

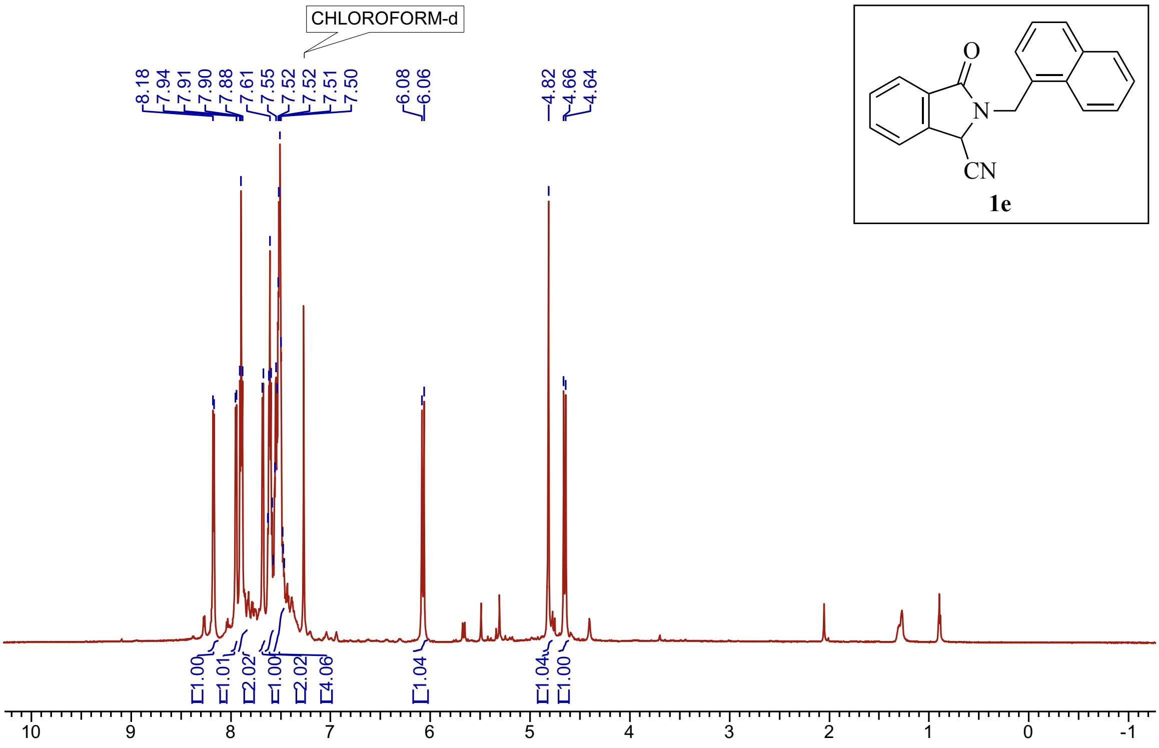

^{1}H-NMR (700 MHz, $CDCl_3$)

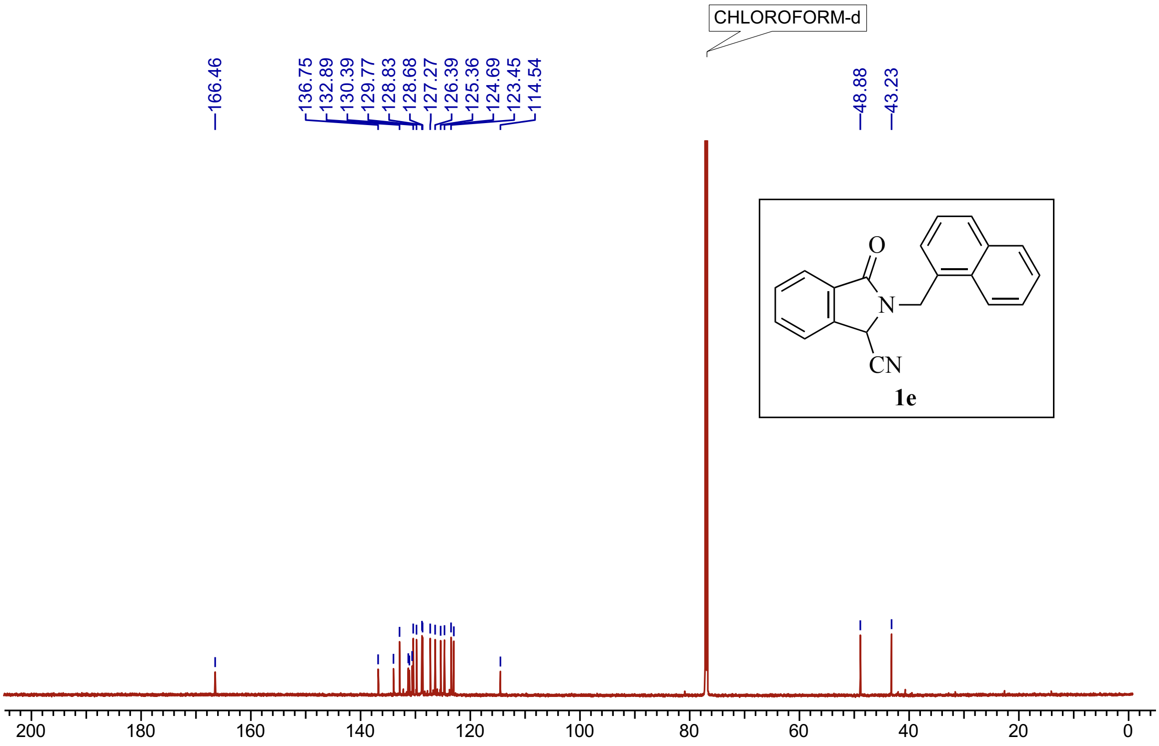

^{13}C-NMR (176 MHz, $CDCl_3$)

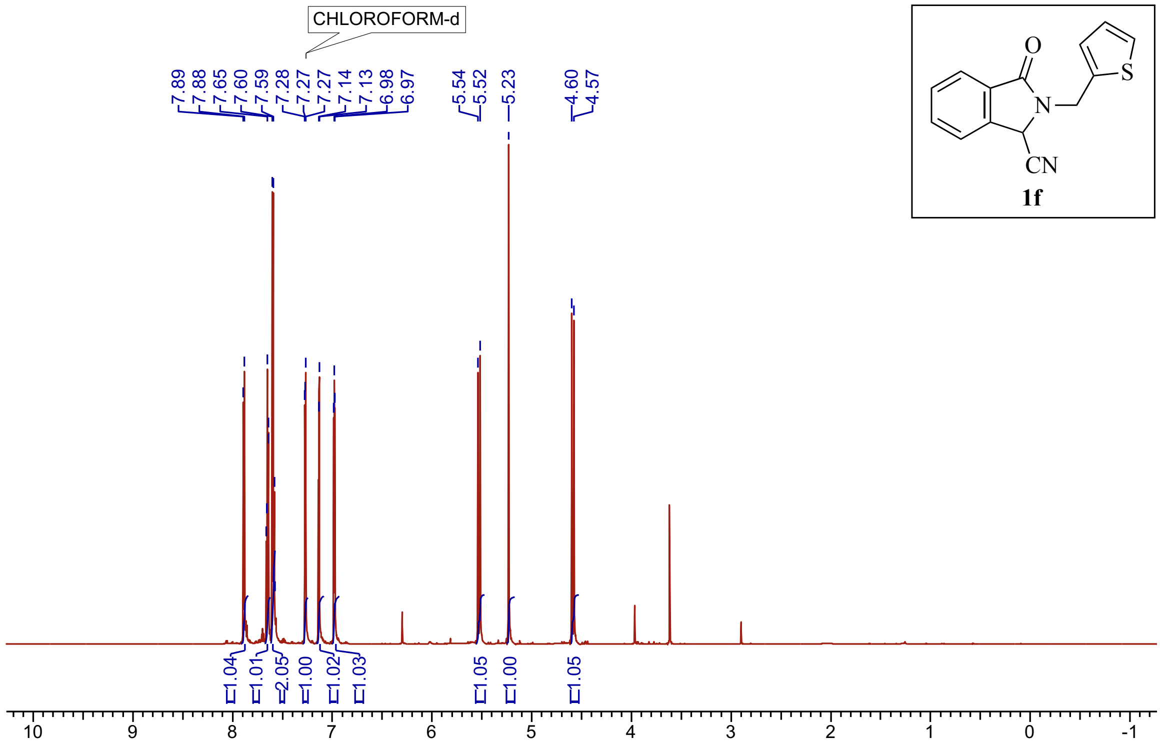

^{1}H-NMR (700 MHz, $CDCl_3$)

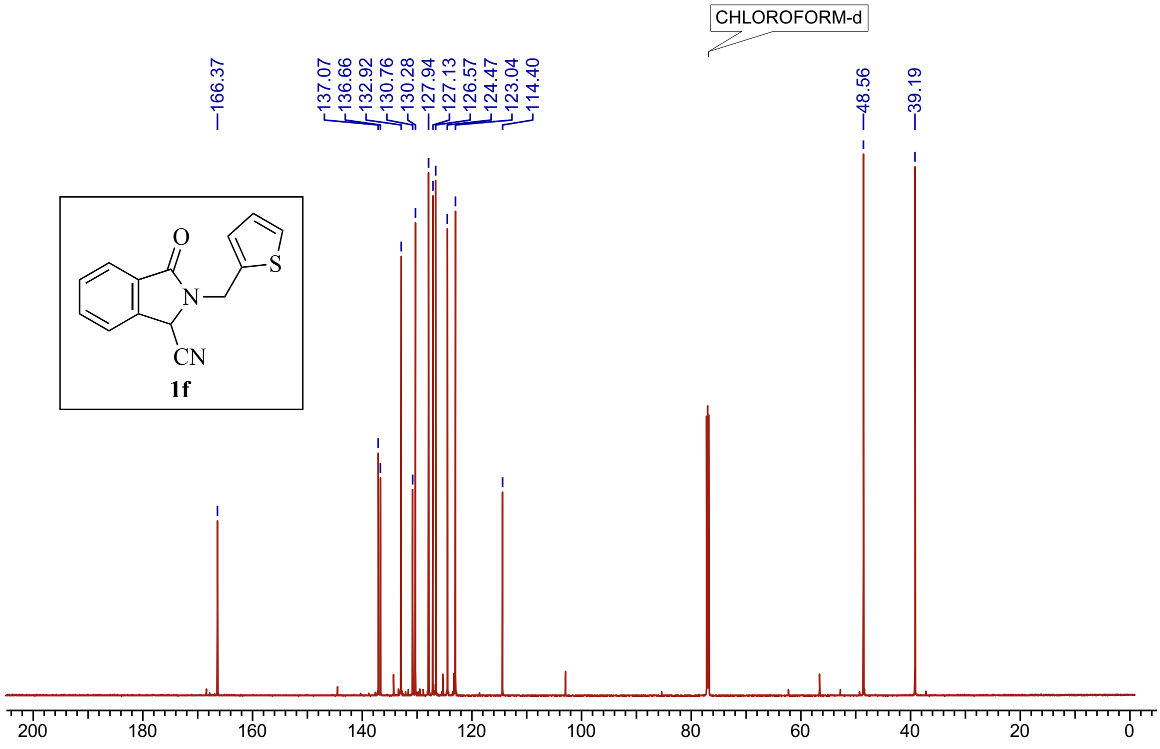

^{13}C-NMR (176 MHz, $CDCl_3$)

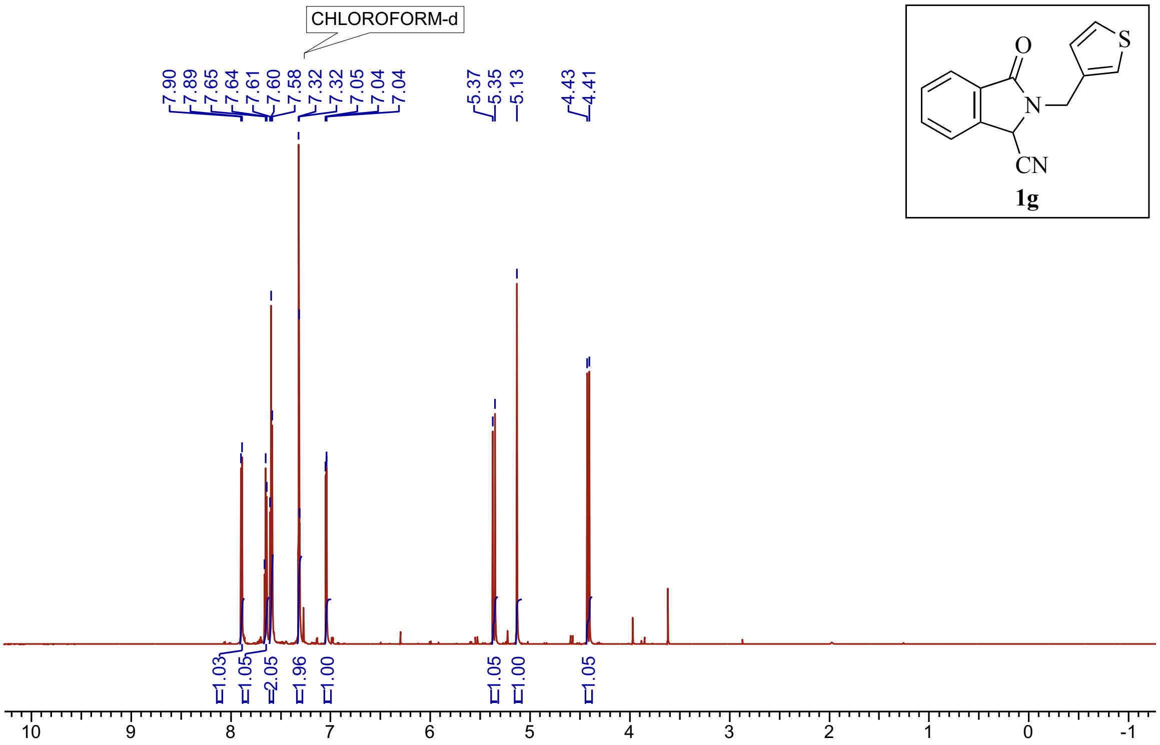

^{1}H-NMR (700 MHz, $CDCl_3$)

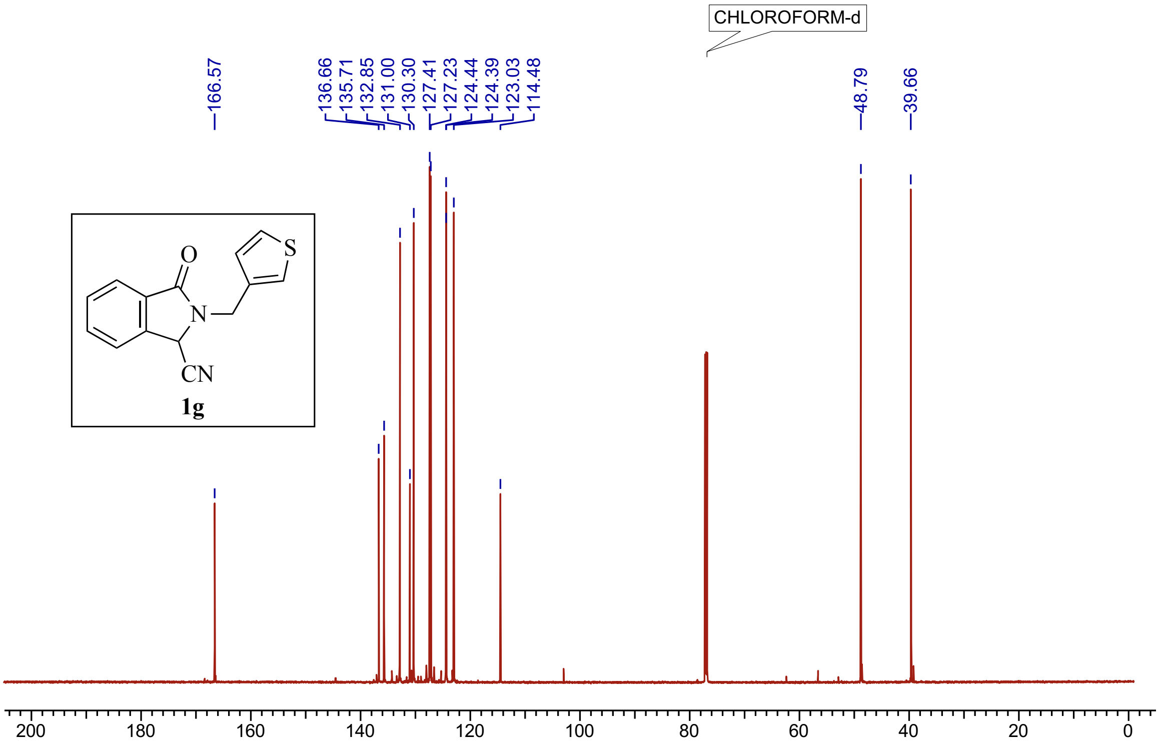

^{13}C-NMR (176 MHz, $CDCl_3$)

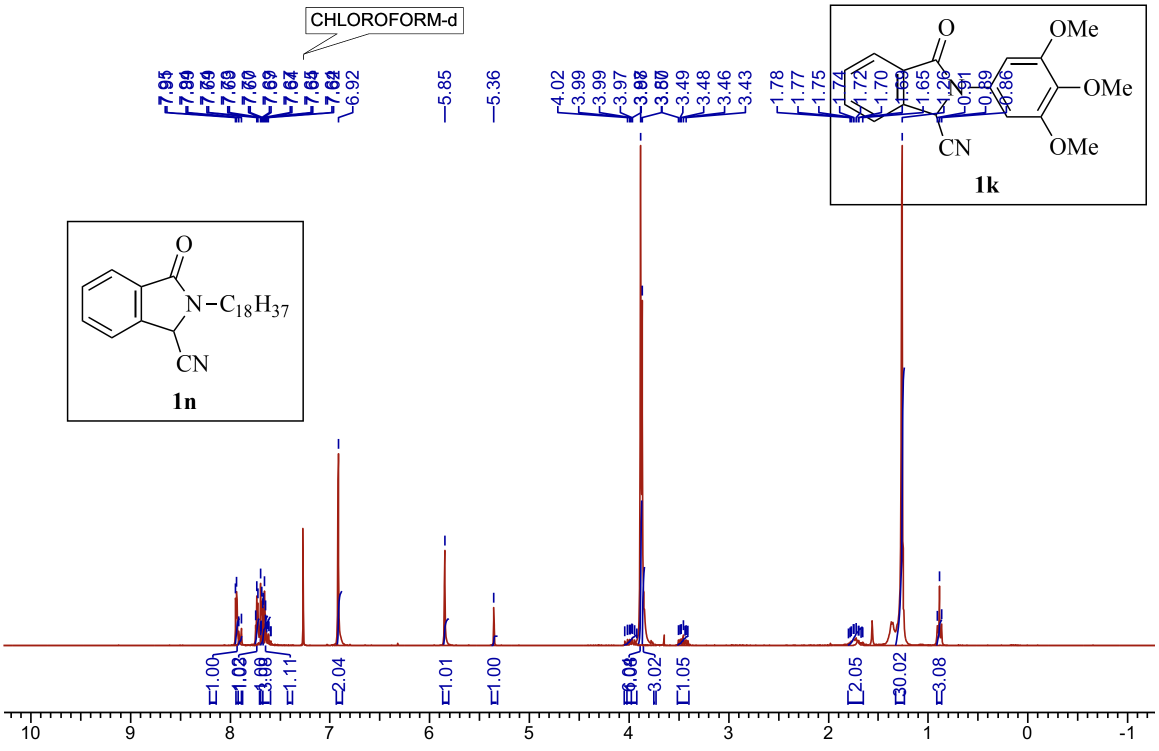

^{1}H-NMR (300 MHz, $CDCl_3$)

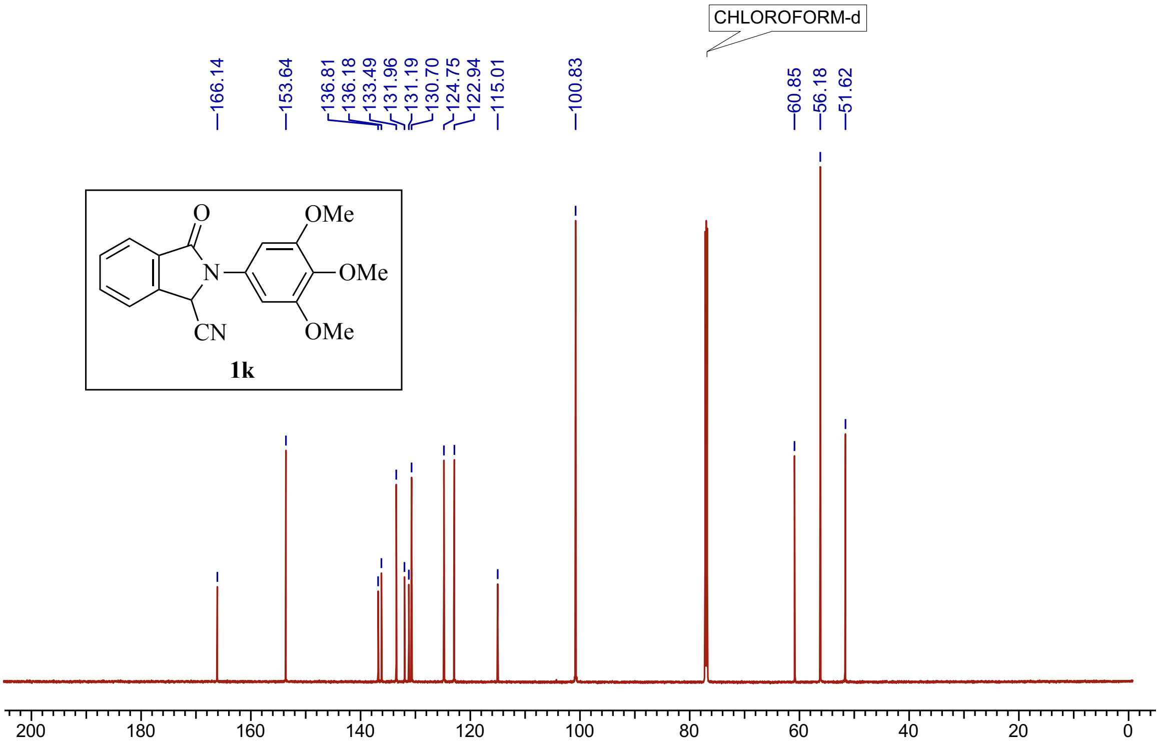

^{13}C-NMR (75 MHz, $CDCl_3$)

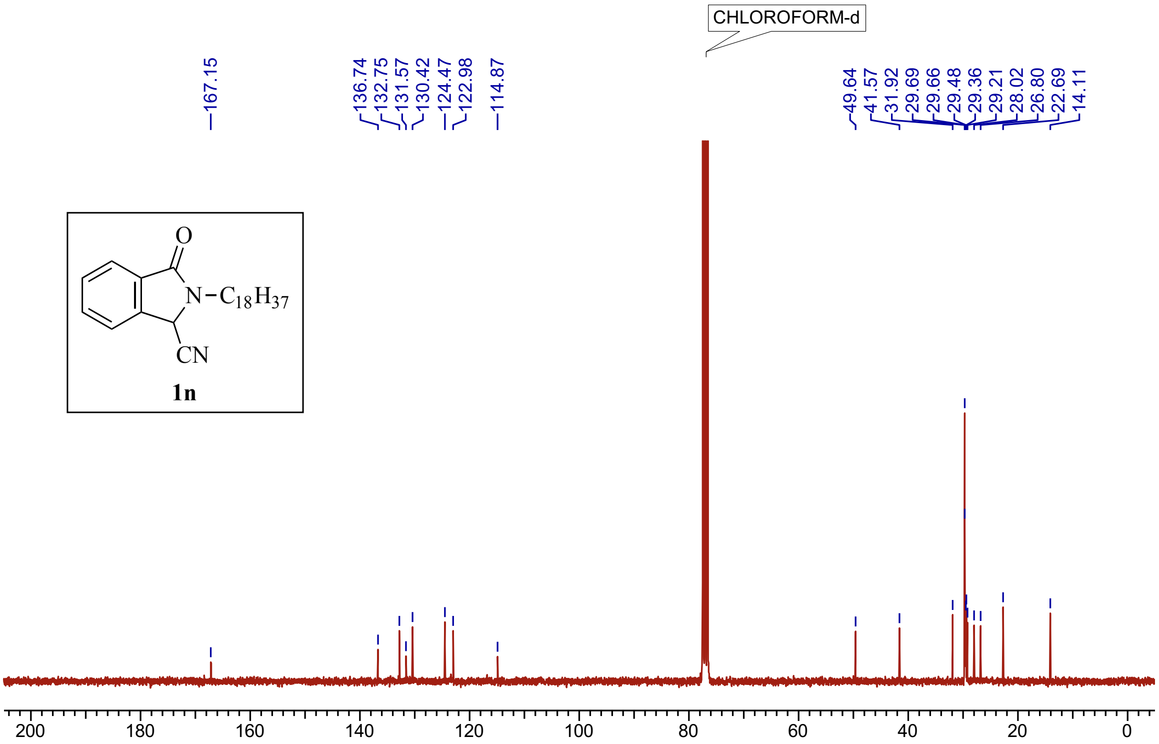

^{13}C-NMR (75 MHz, $CDCl_3$)

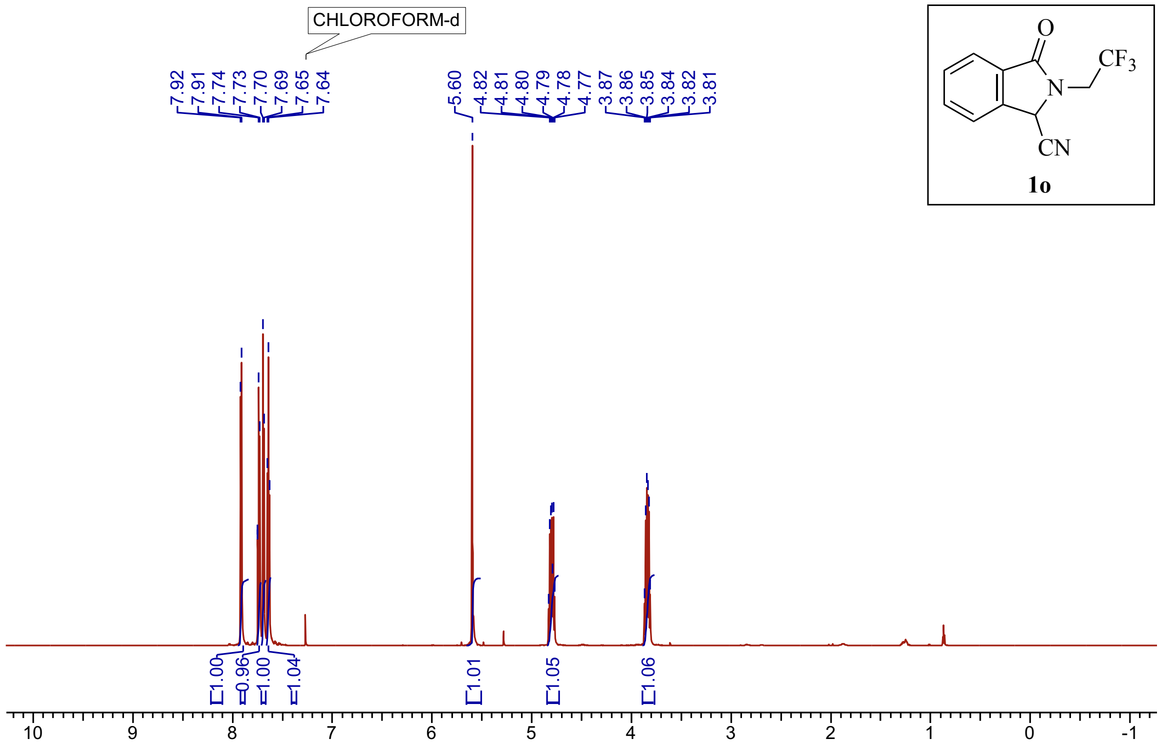

^{1}H-NMR (700 MHz, $CDCl_3$)

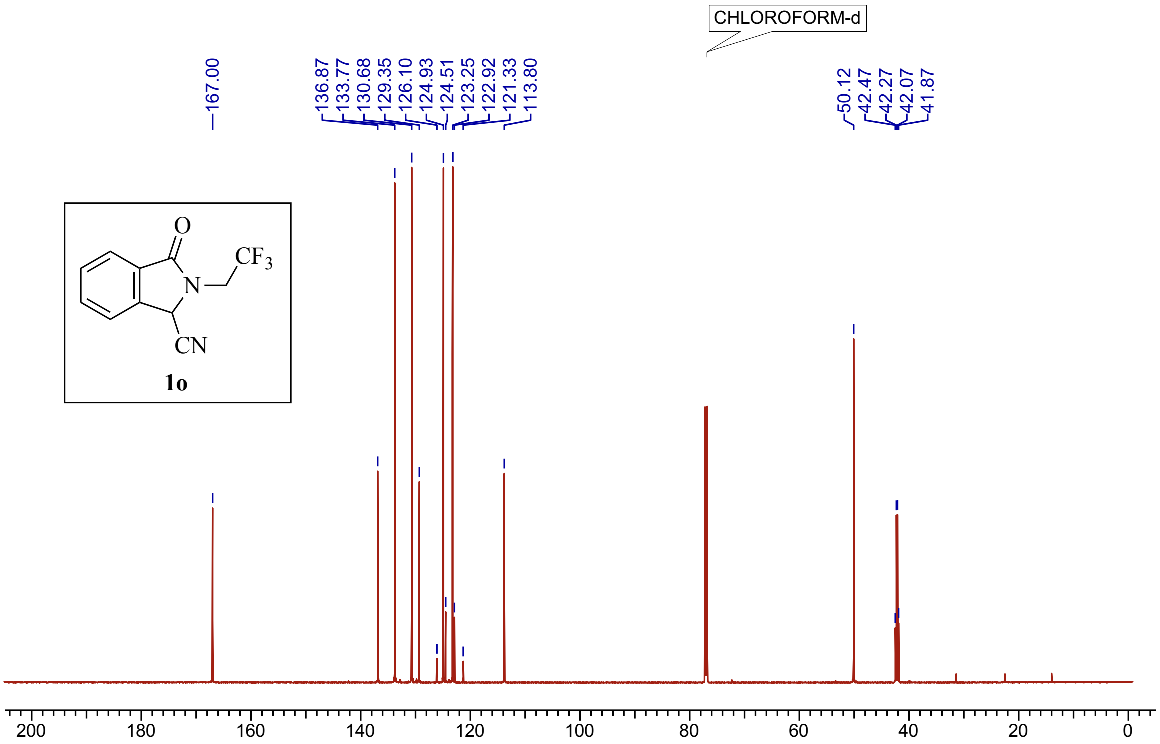

^{13}C-NMR (176 MHz, $CDCl_3$)

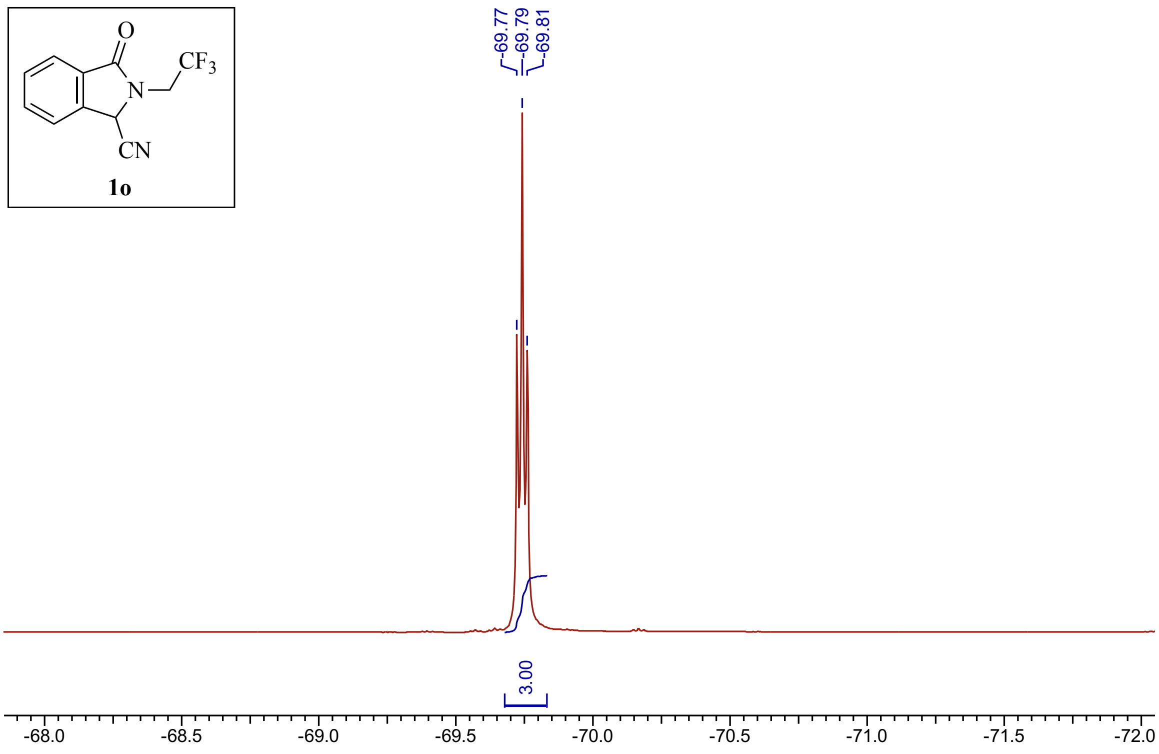

^{19}F-NMR (471 MHz, $CDCl_3$)

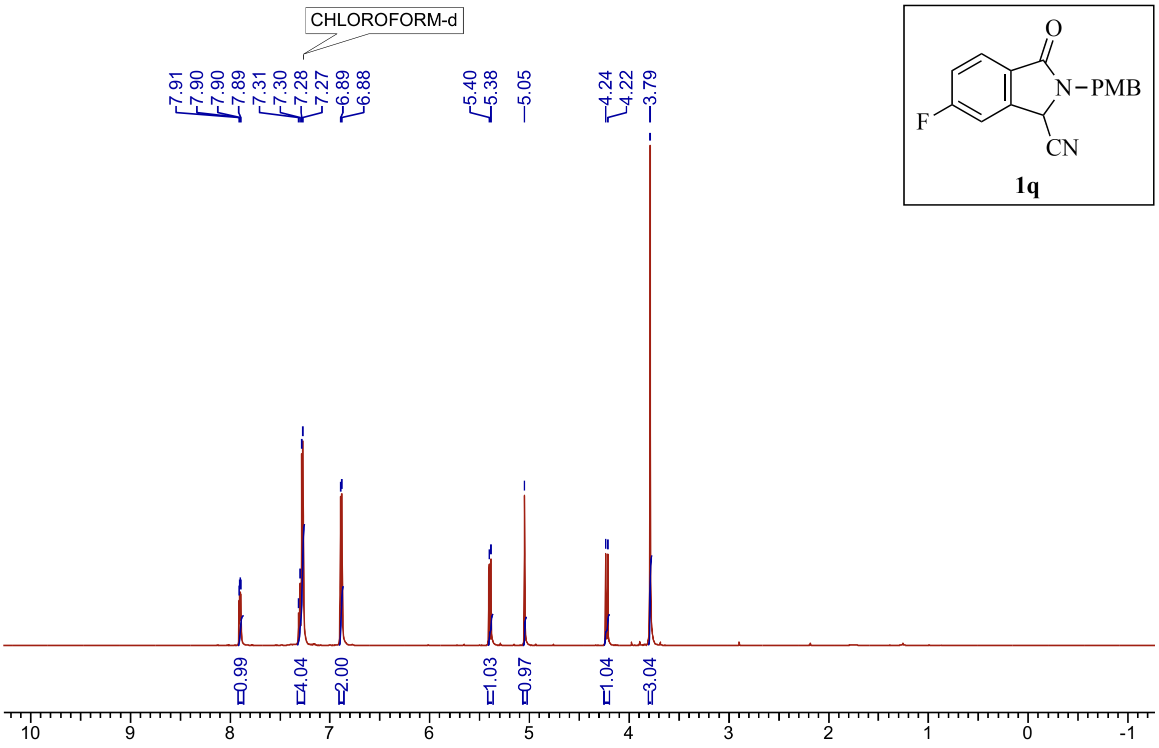

^{1}H-NMR (700 MHz, $CDCl_3$)

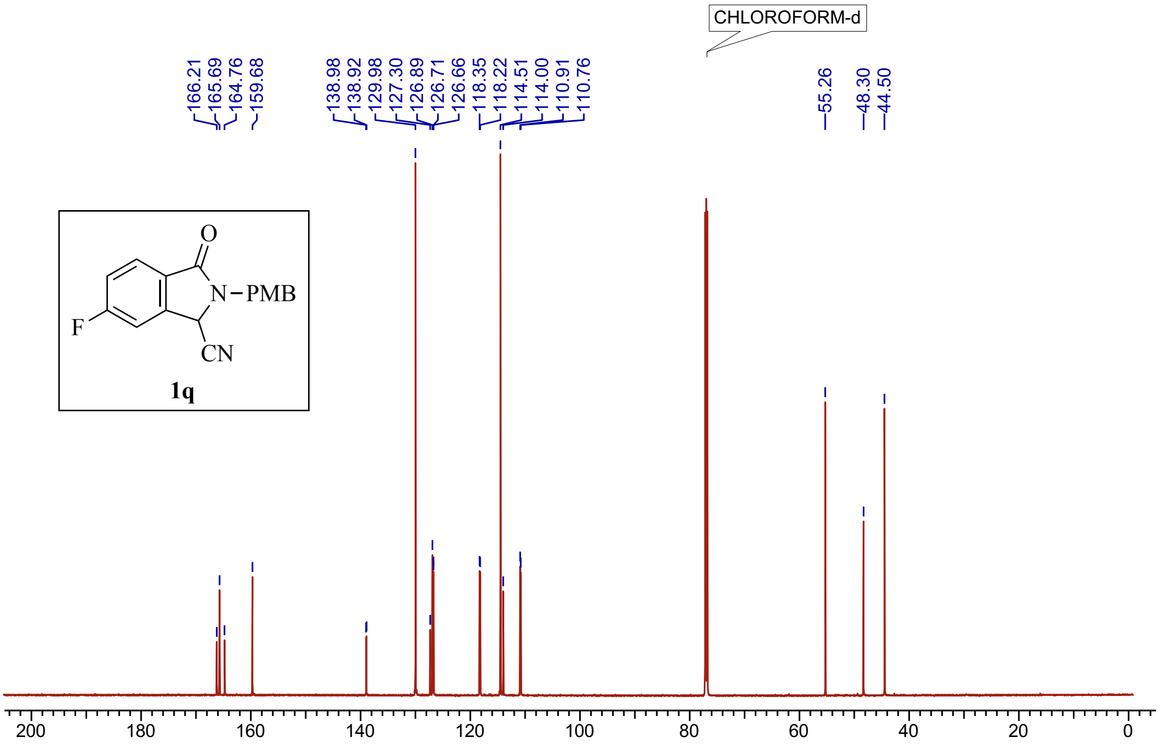

^{13}C-NMR (176 MHz, CDCl$_3$)

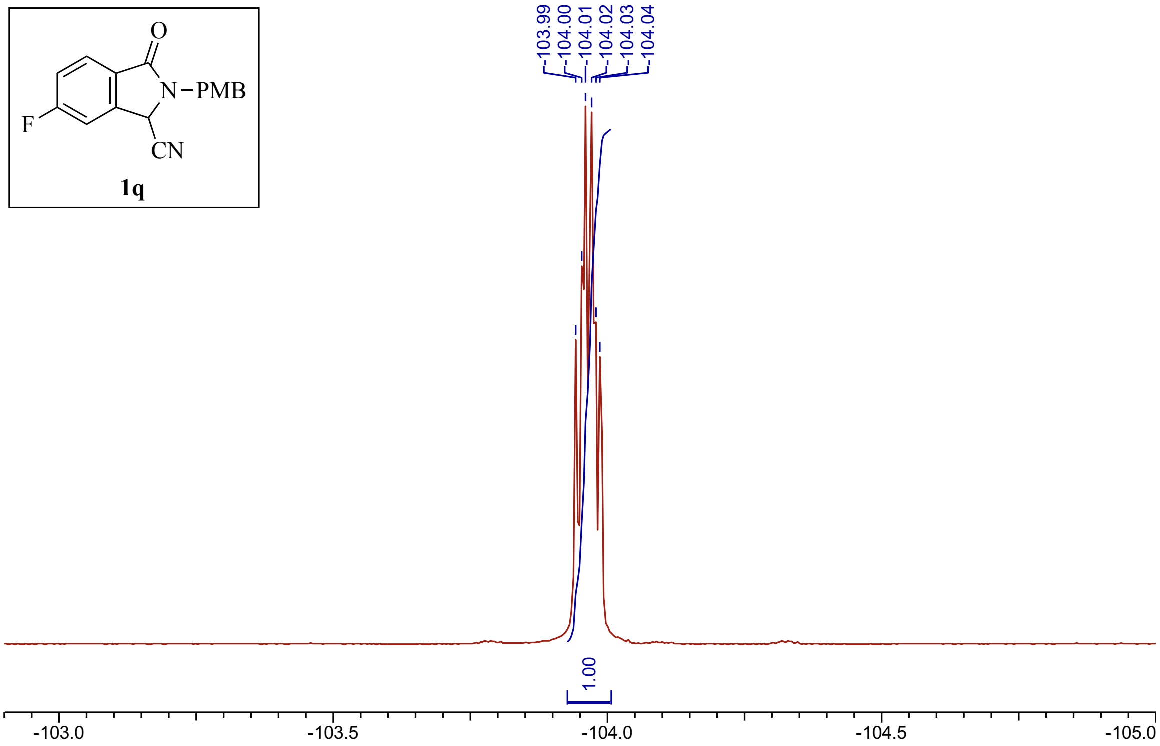

^{19}F-NMR (471 MHz, CDCl$_3$)

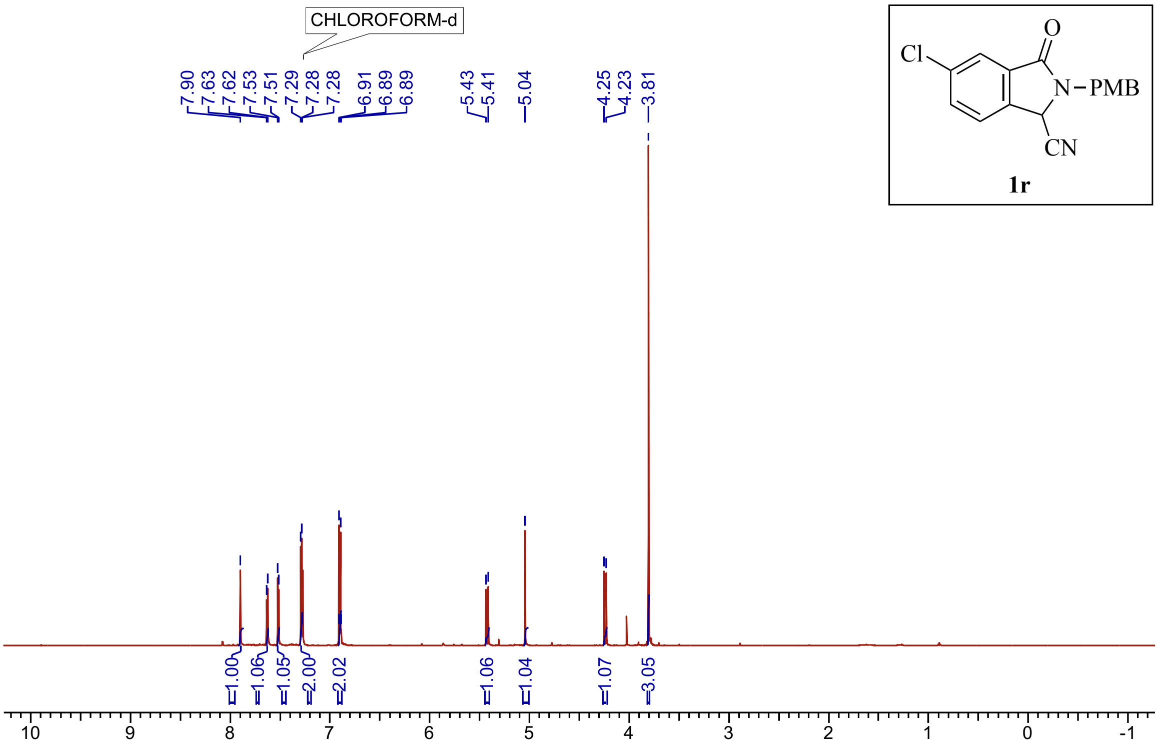

^{1}H-NMR (700 MHz, $CDCl_3$)

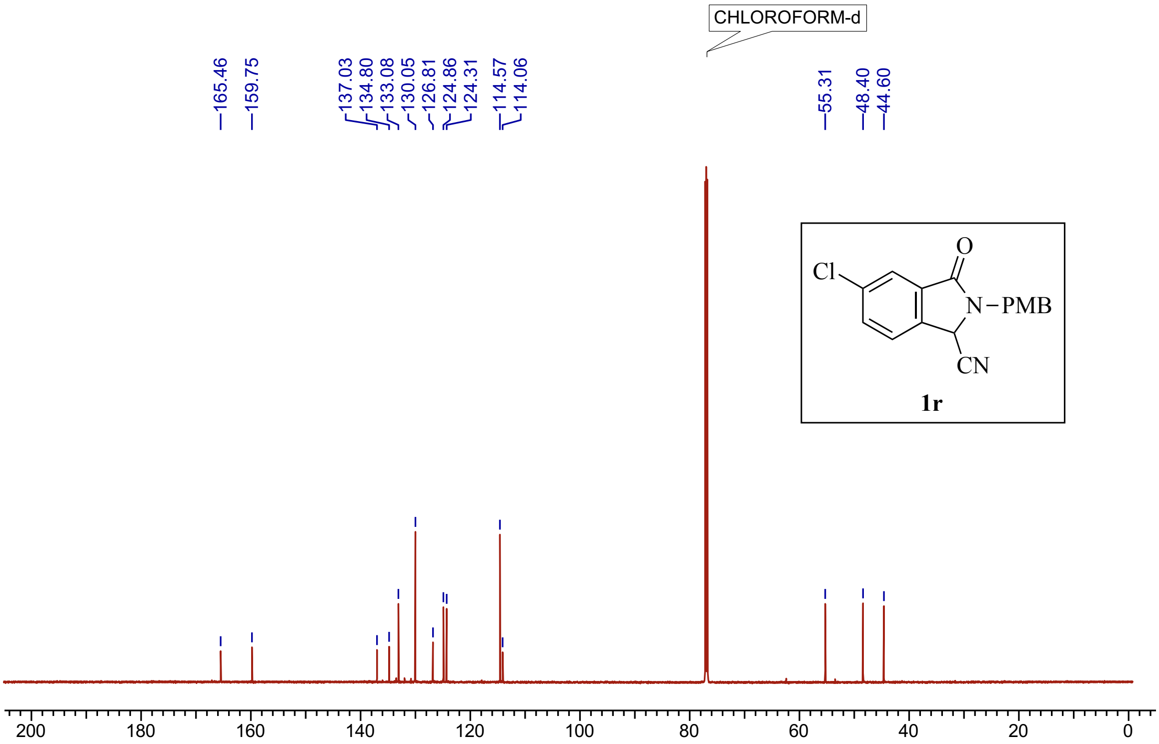

^{13}C-NMR (176 MHz, $CDCl_3$)

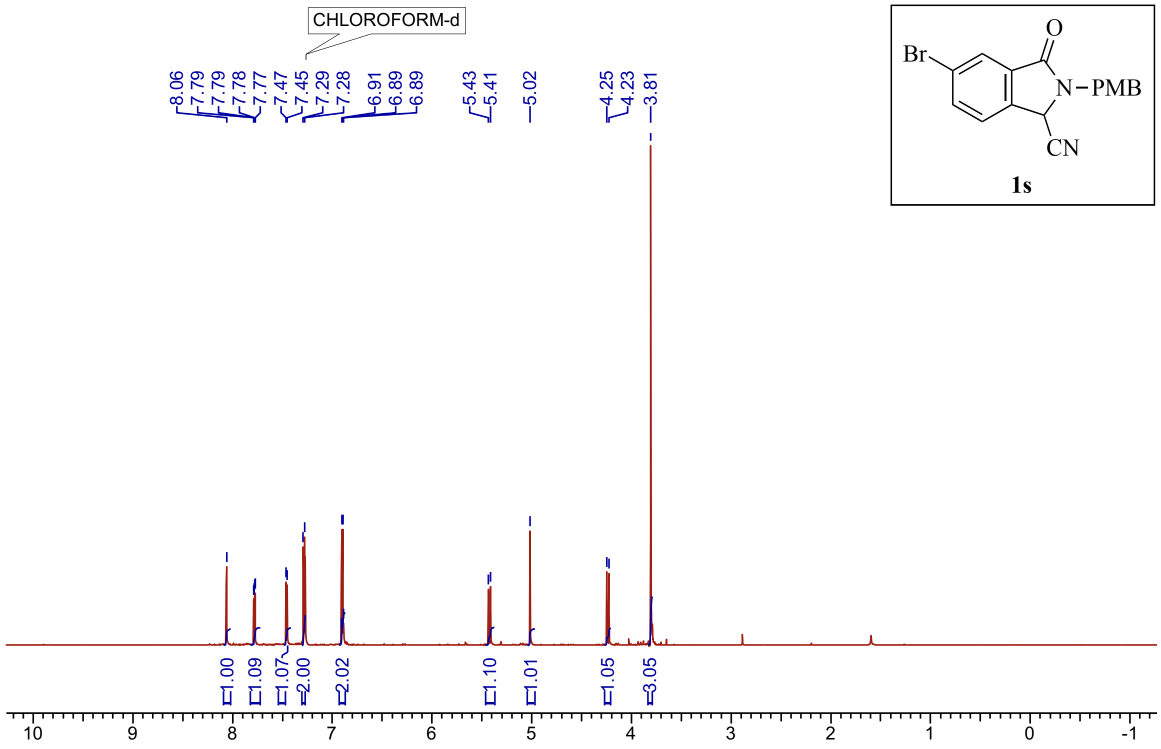

^{1}H-NMR (700 MHz, $CDCl_3$)

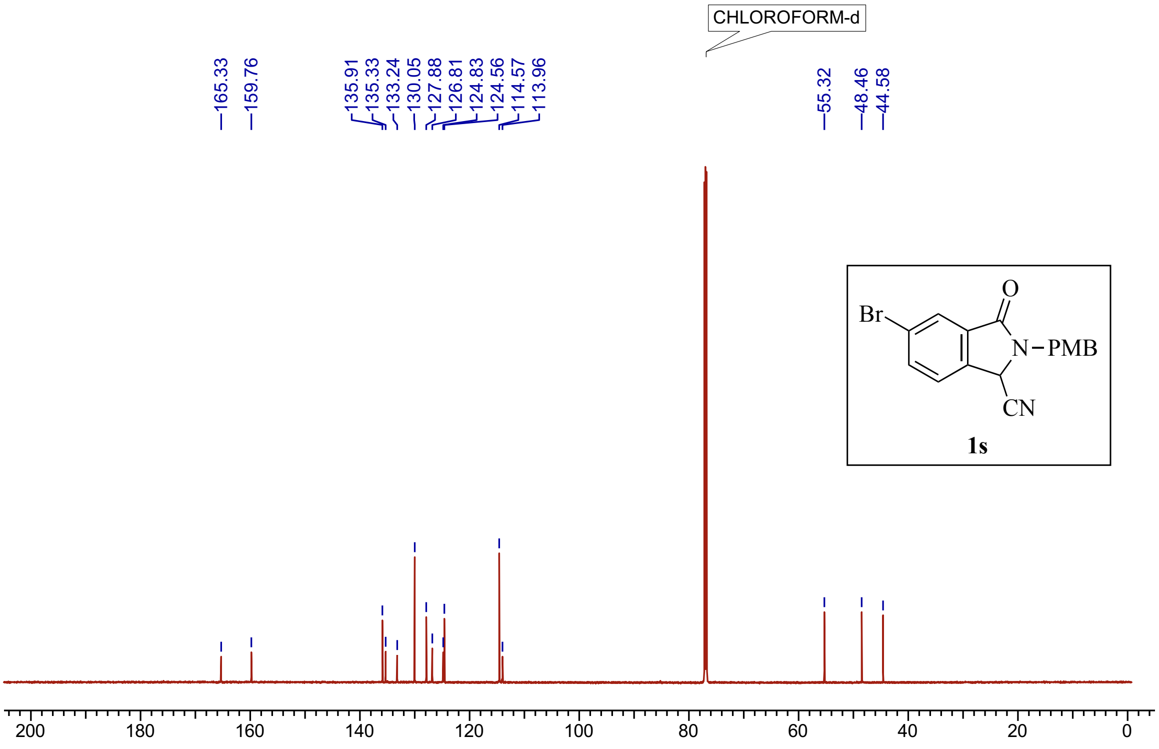

^{13}C-NMR (176 MHz, $CDCl_3$)

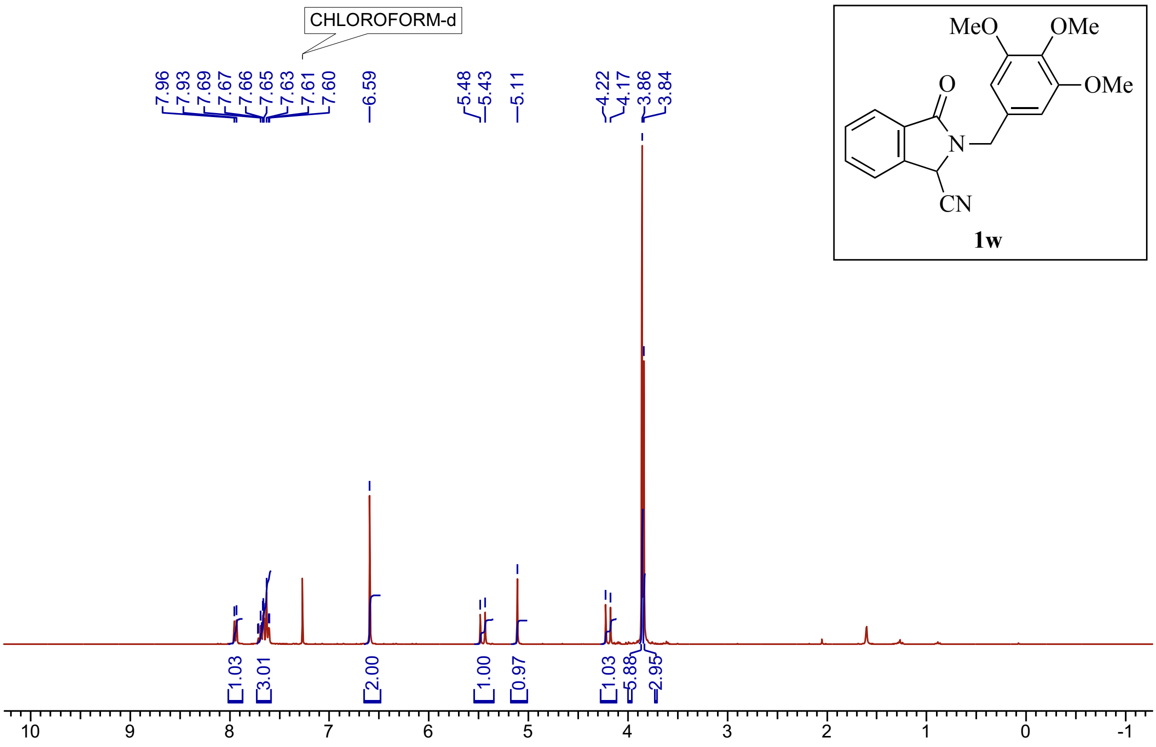

^{1}H-NMR (300 MHz, $CDCl_3$)

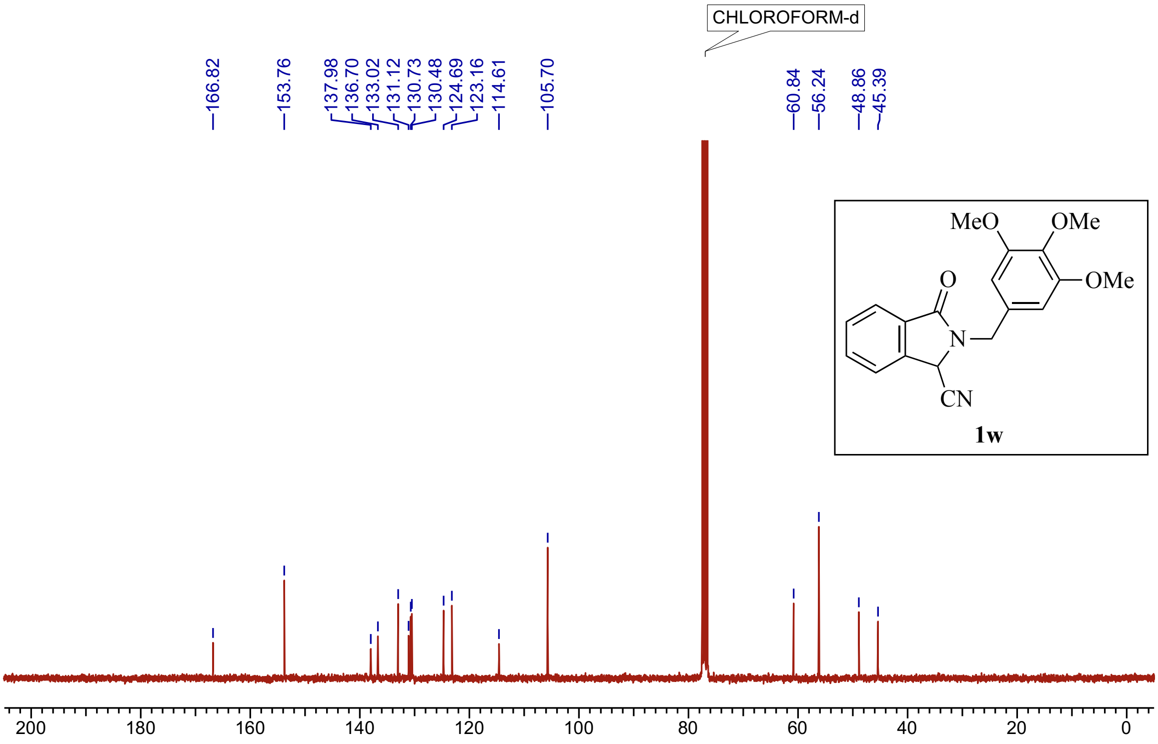

^{13}C-NMR (75 MHz, $CDCl_3$)

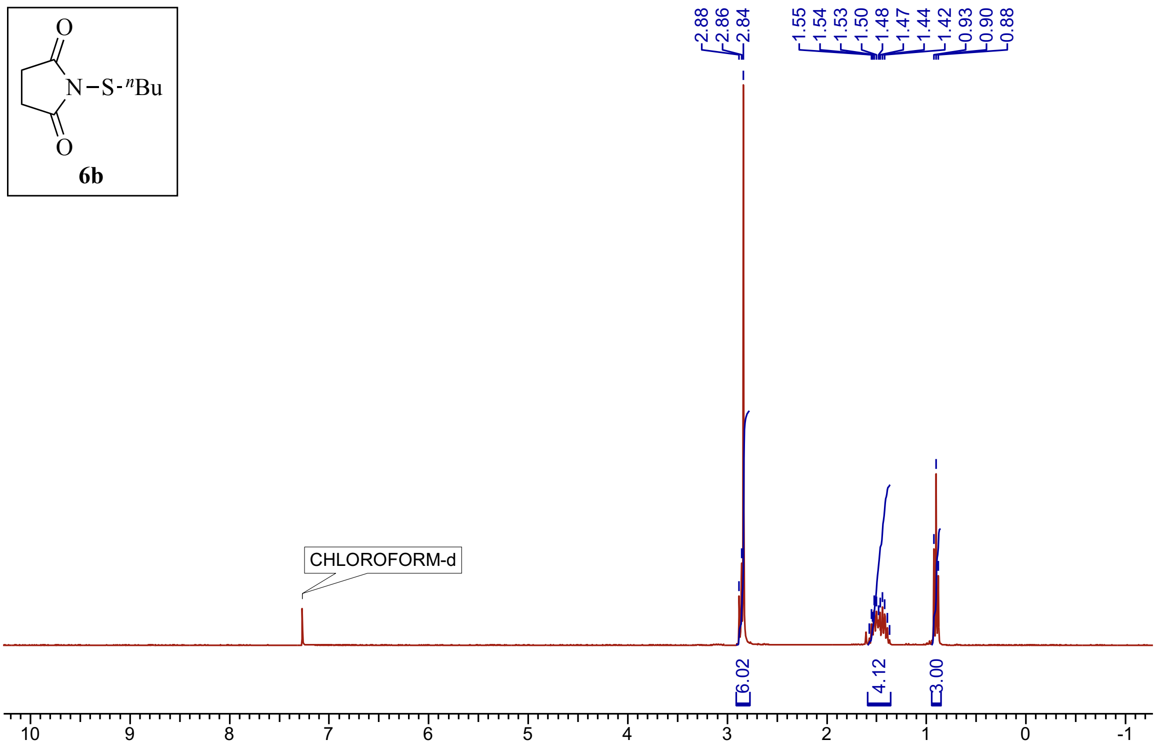

^{1}H-NMR (300 MHz, $CDCl_3$)

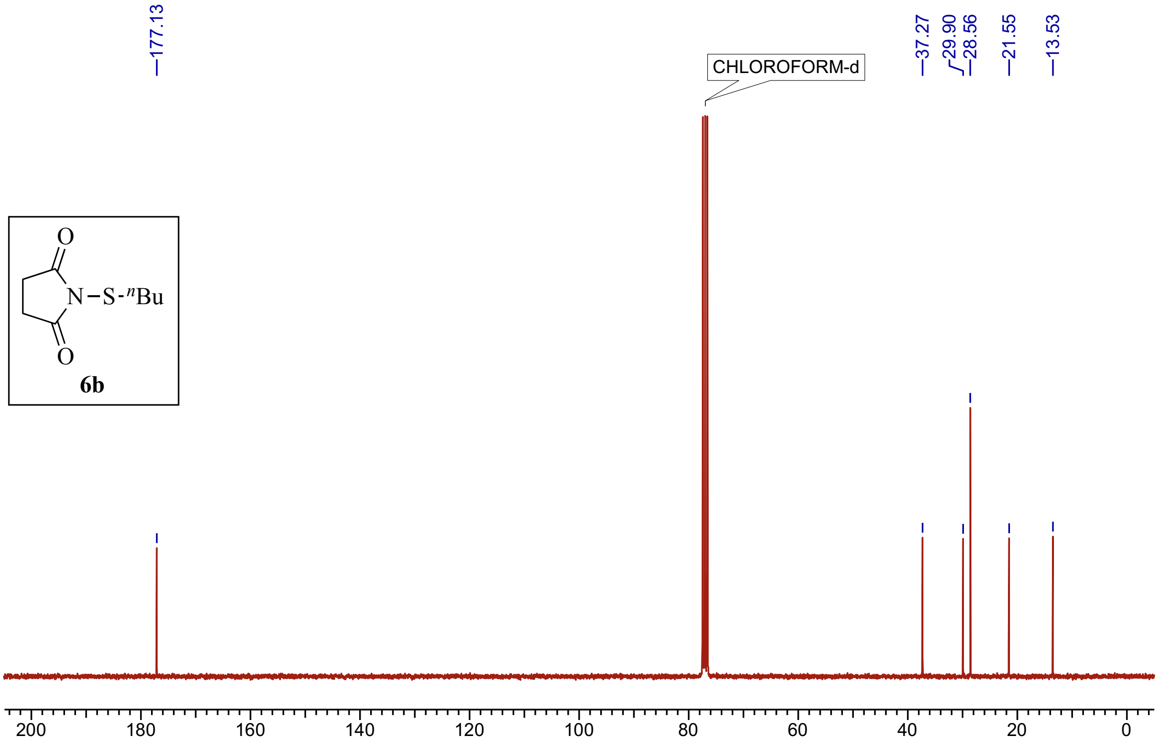

^{13}C-NMR (75 MHz, $CDCl_3$)

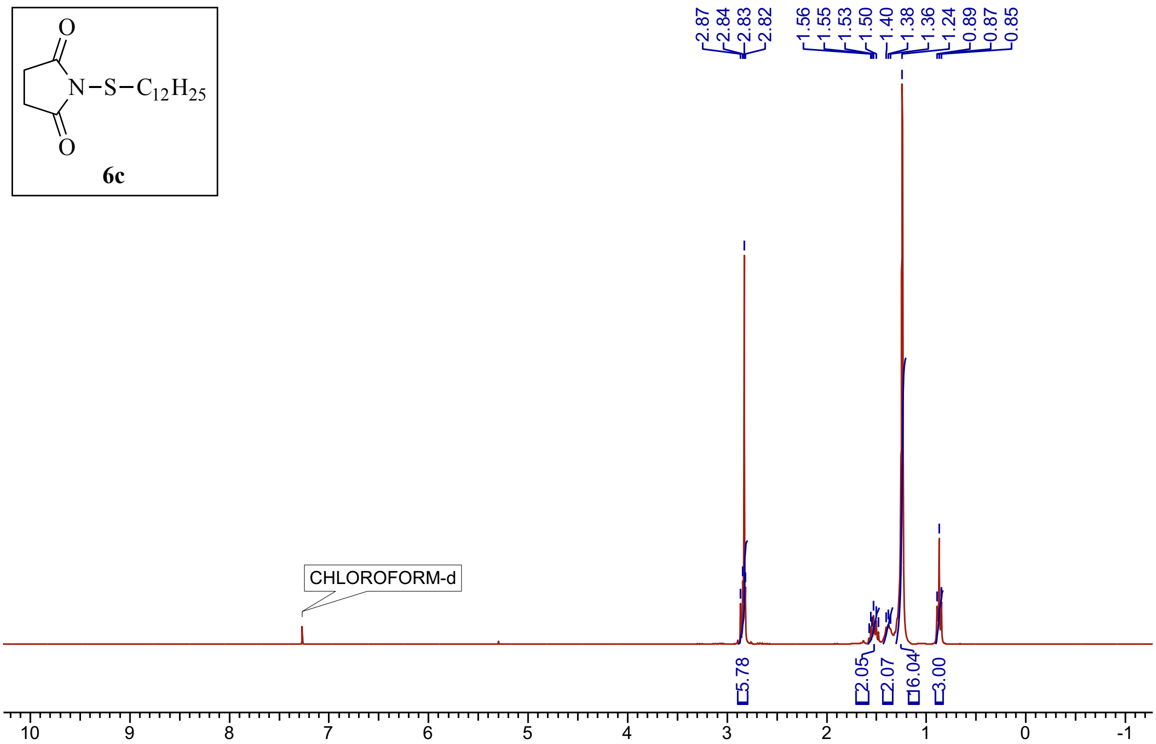

^{1}H-NMR (300 MHz, $CDCl_3$)

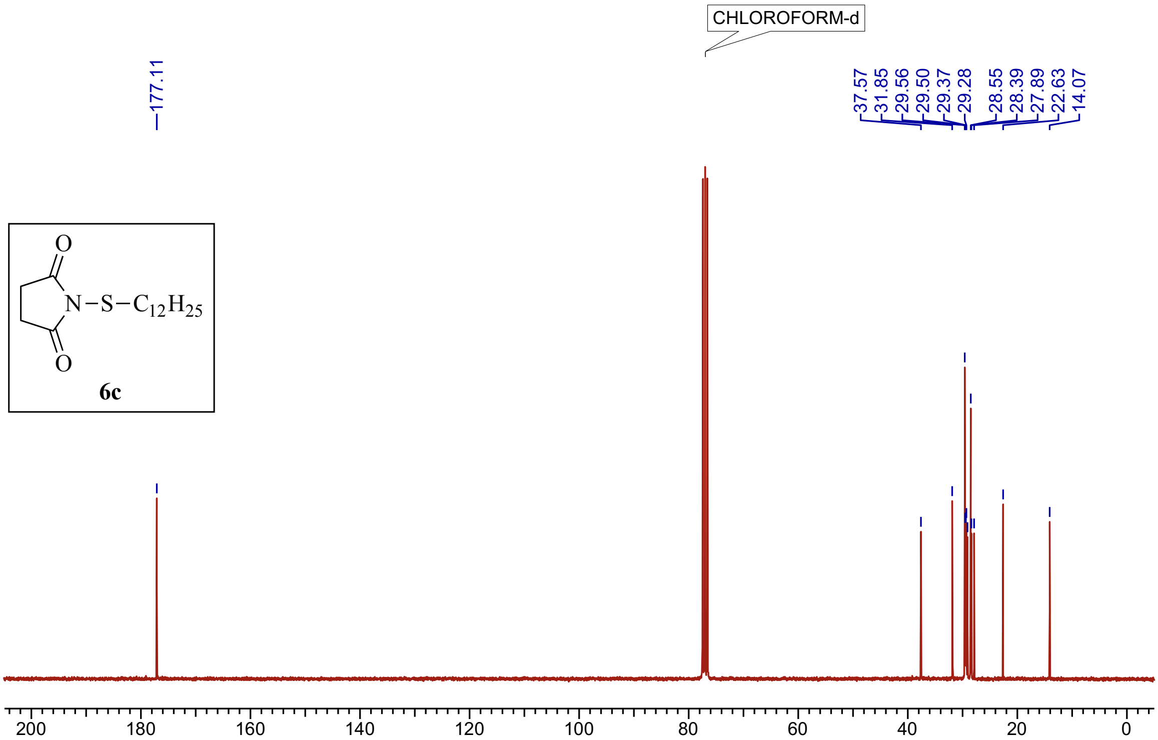

^{13}C-NMR (75 MHz, $CDCl_3$)

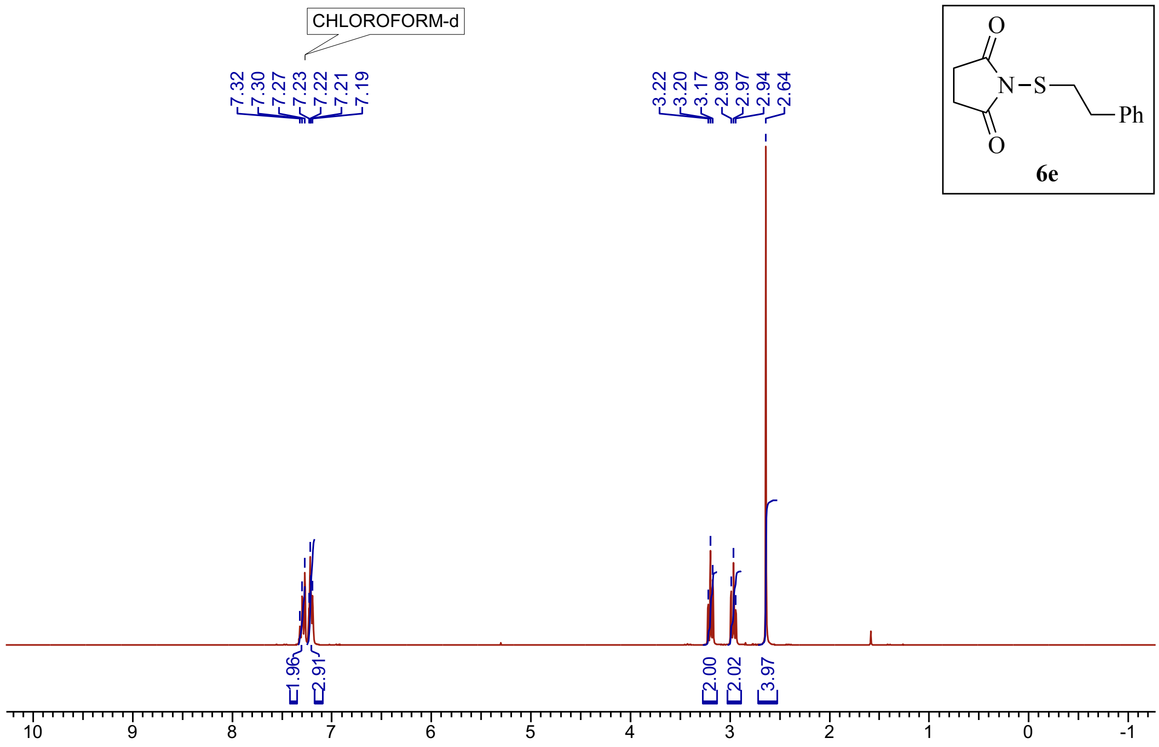

^{1}H-NMR (300 MHz, $CDCl_3$)

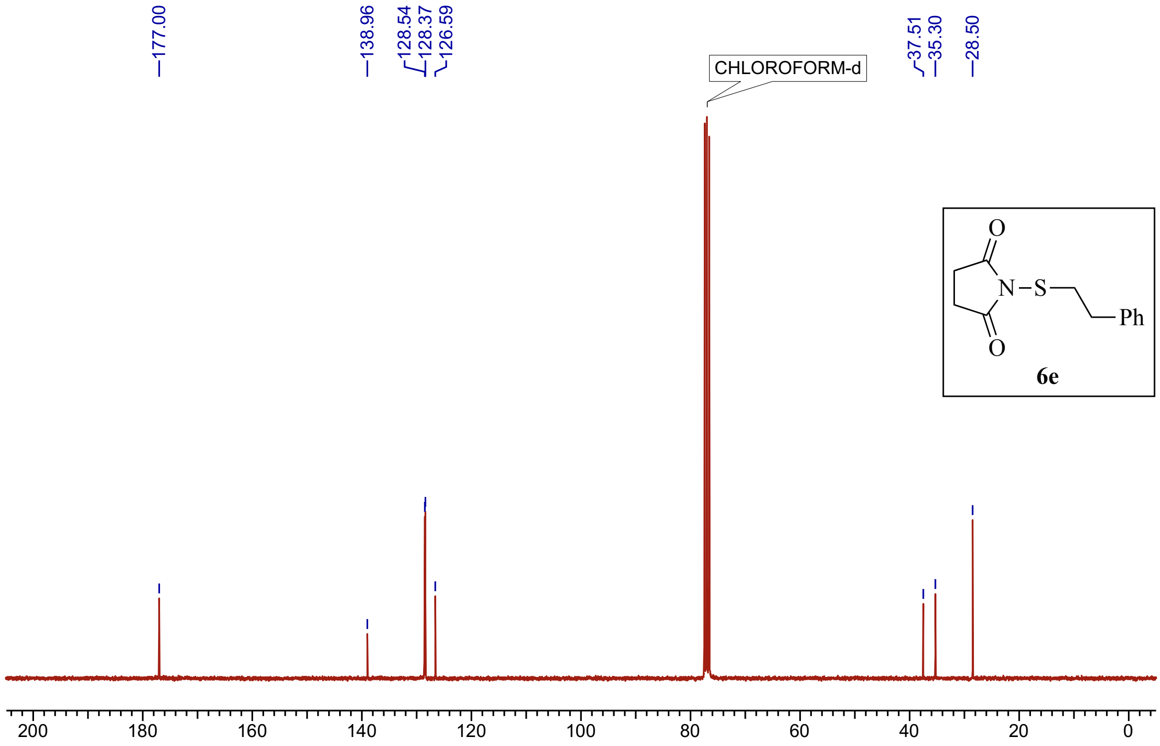

^{13}C-NMR (75 MHz, $CDCl_3$)

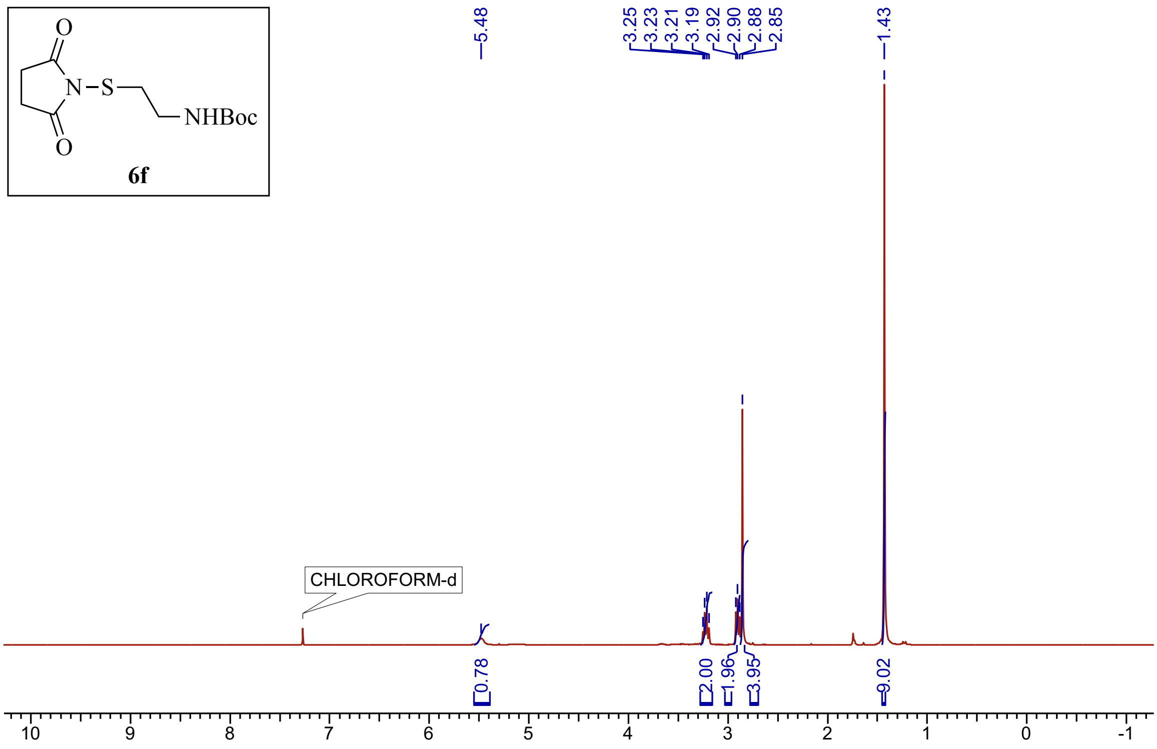

^{1}H-NMR (300 MHz, $CDCl_3$)

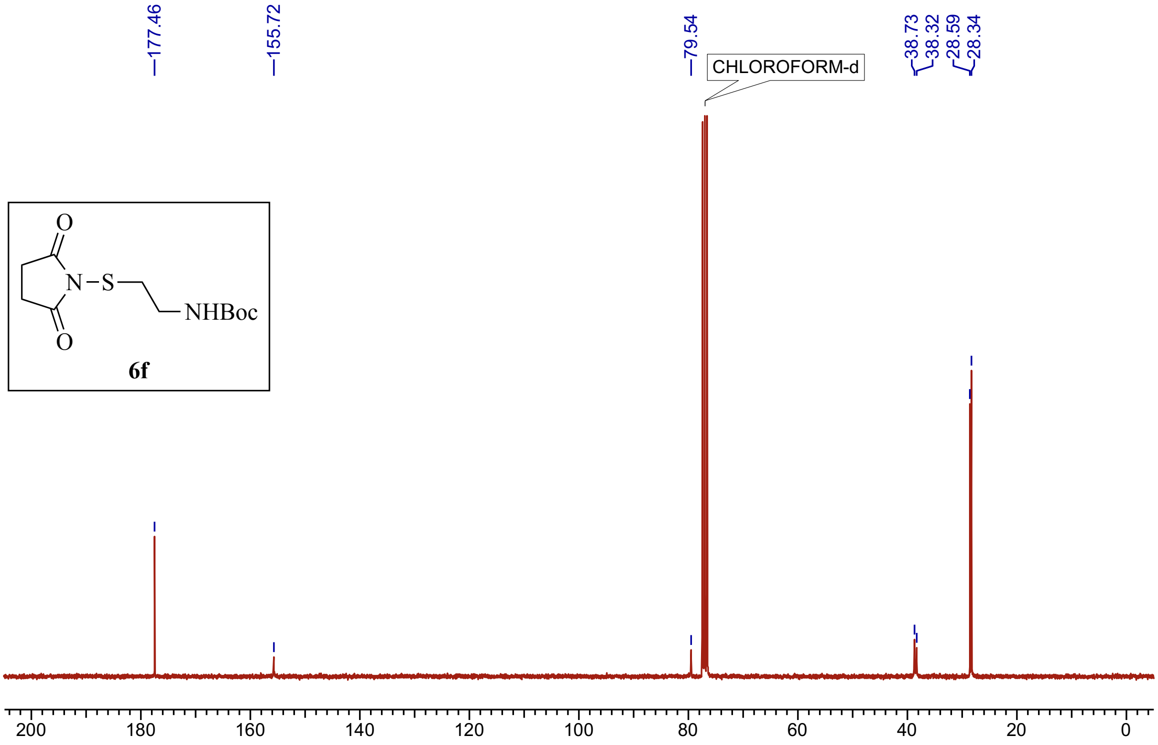

^{13}C-NMR (75 MHz, $CDCl_3$)

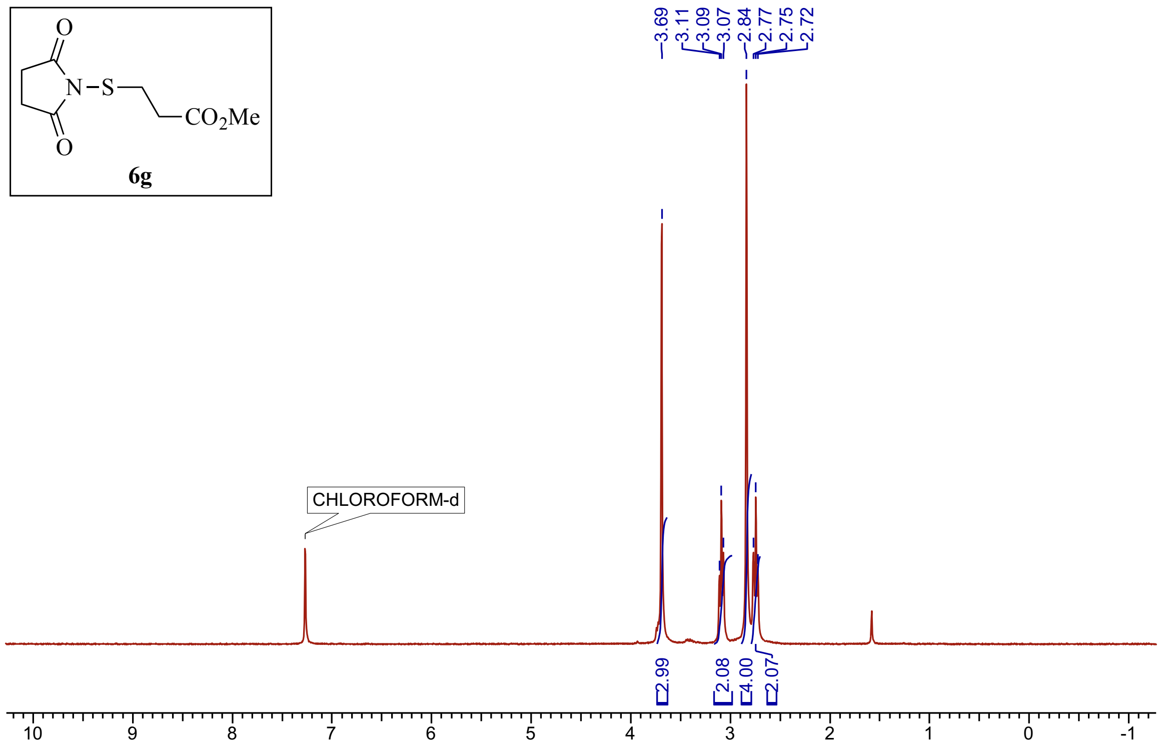

^{1}H-NMR (300 MHz, $CDCl_3$)

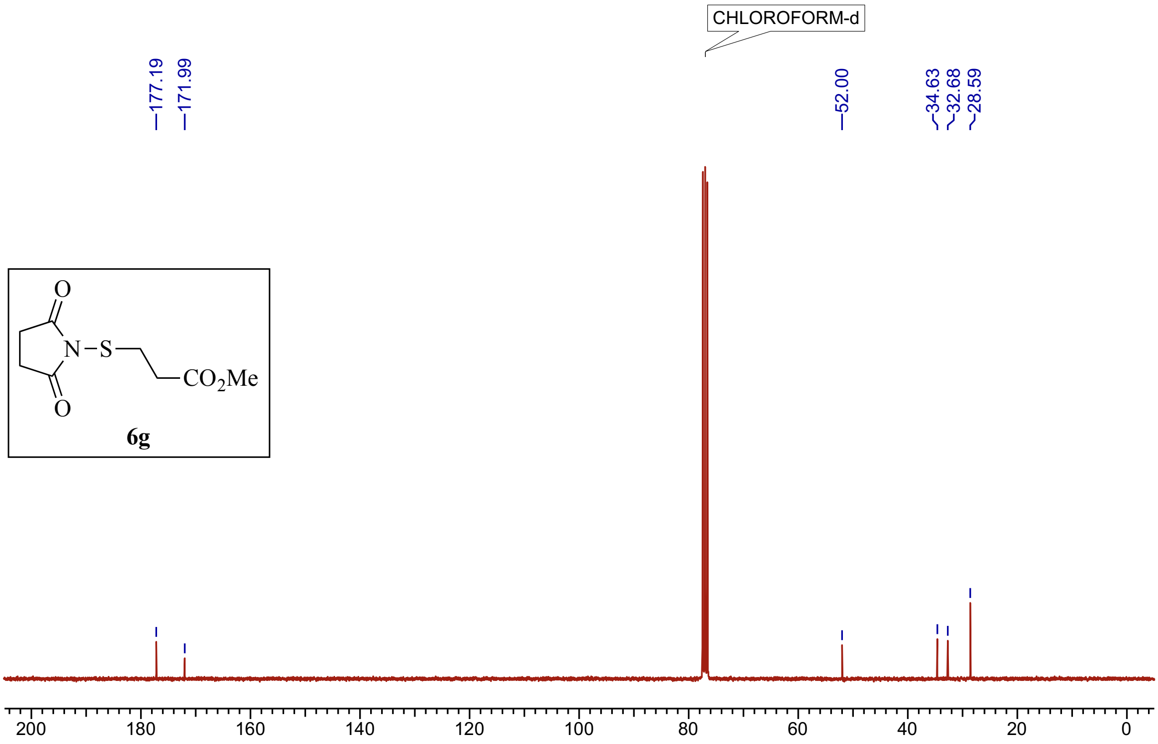

^{13}C-NMR (75 MHz, $CDCl_3$)

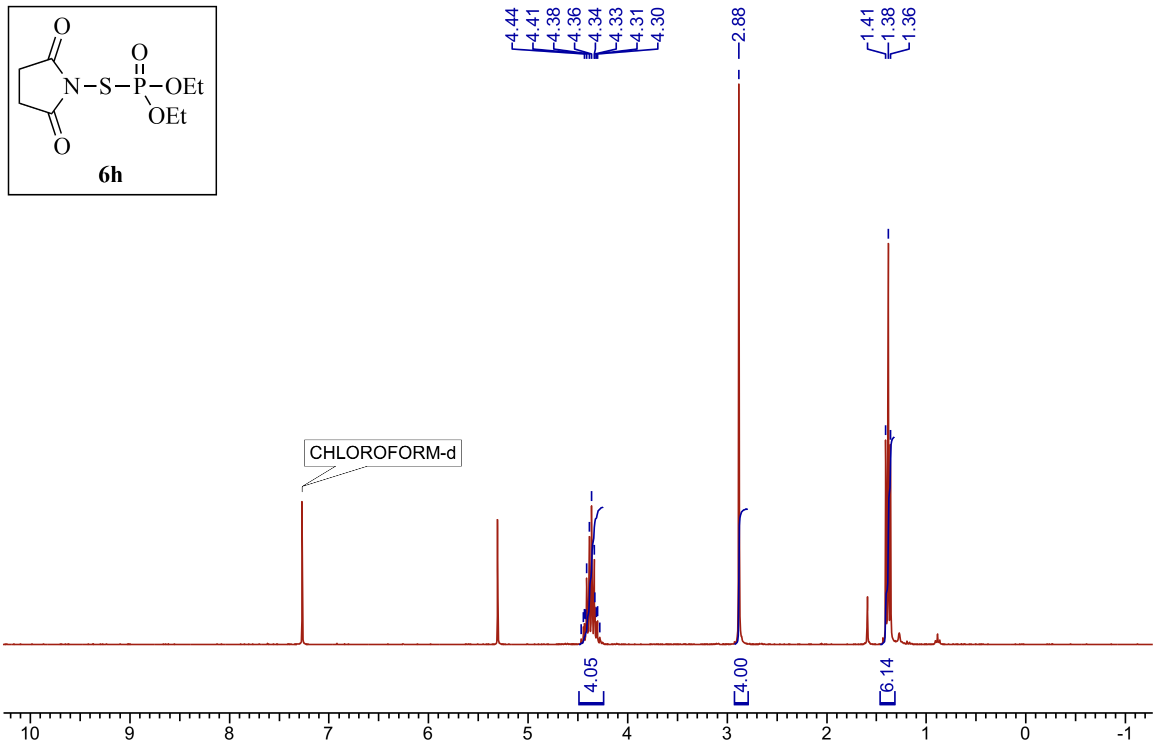

^{1}H-NMR (300 MHz, $CDCl_3$)

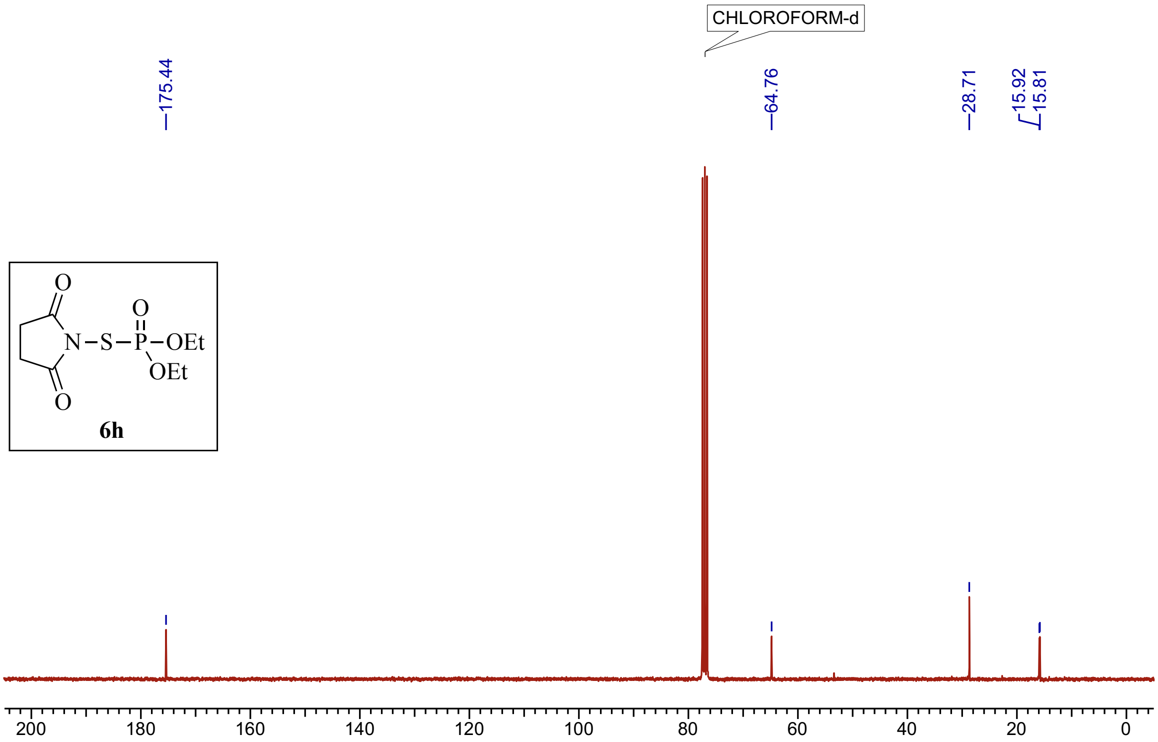

^{13}C-NMR (75 MHz, $CDCl_3$)

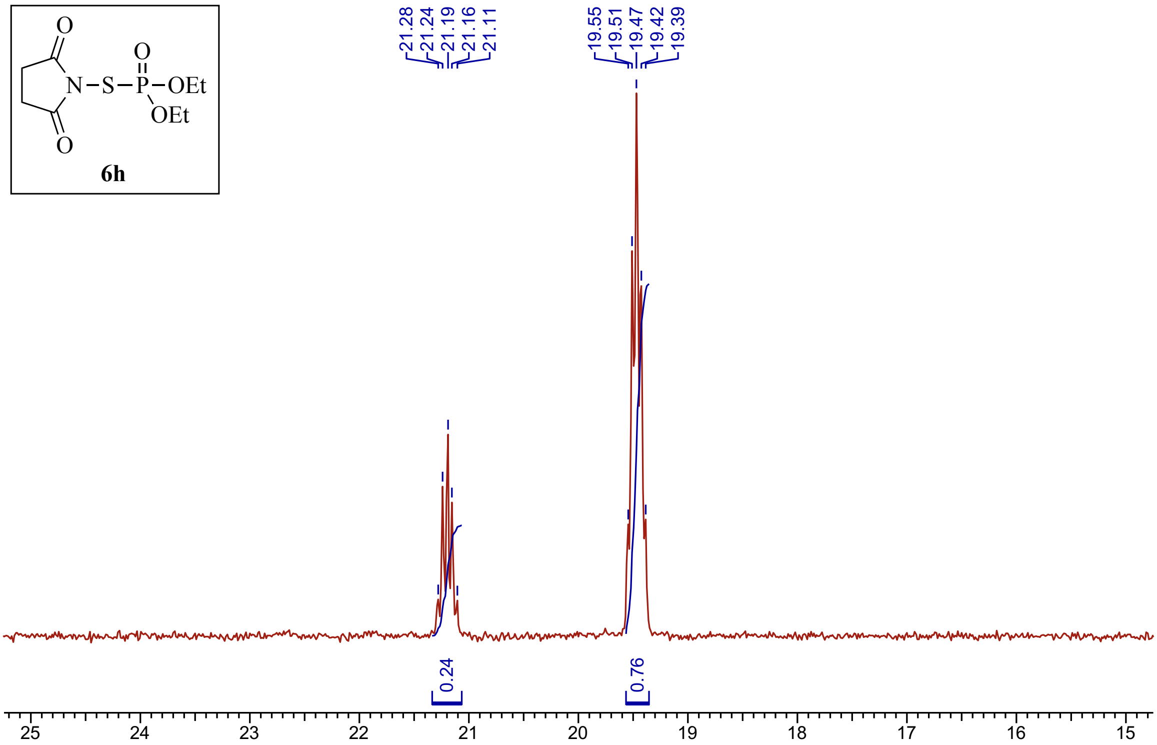

^{31}P-NMR (202 MHz, $CDCl_3$)

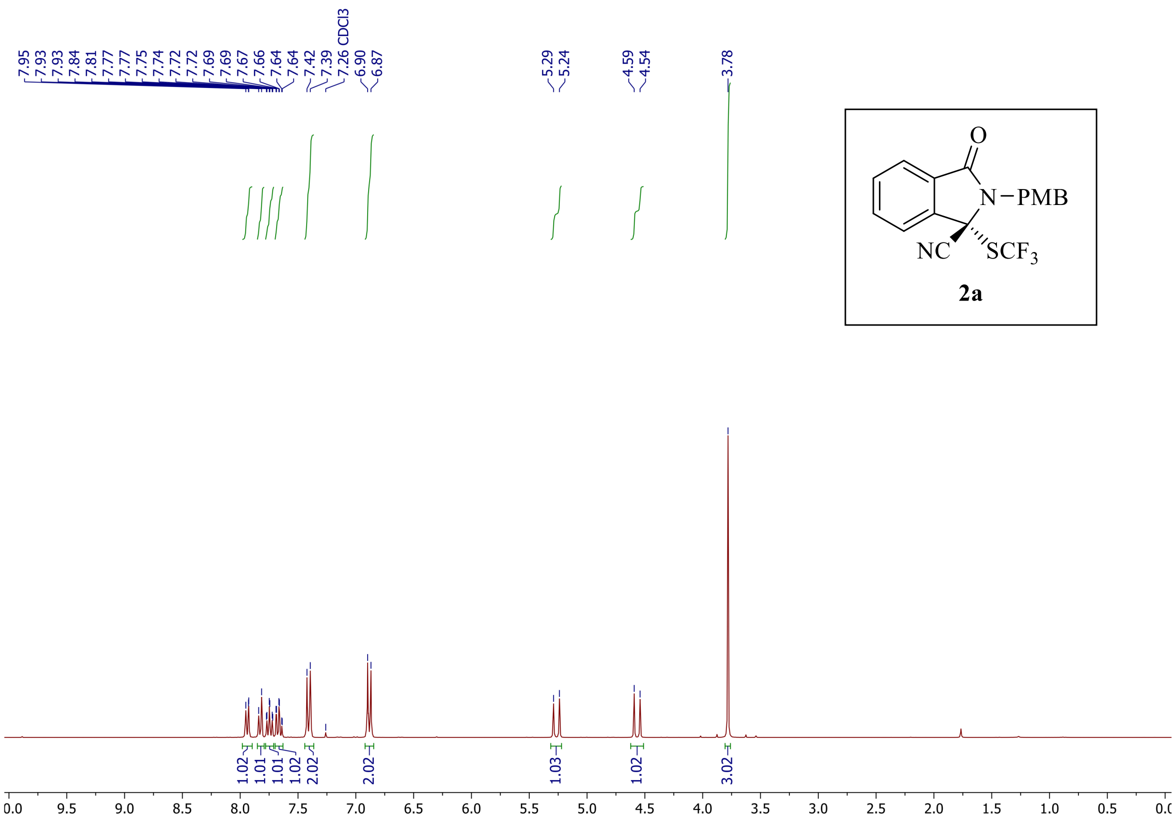

^{1}H-NMR (300 MHz, $CDCl_3$)

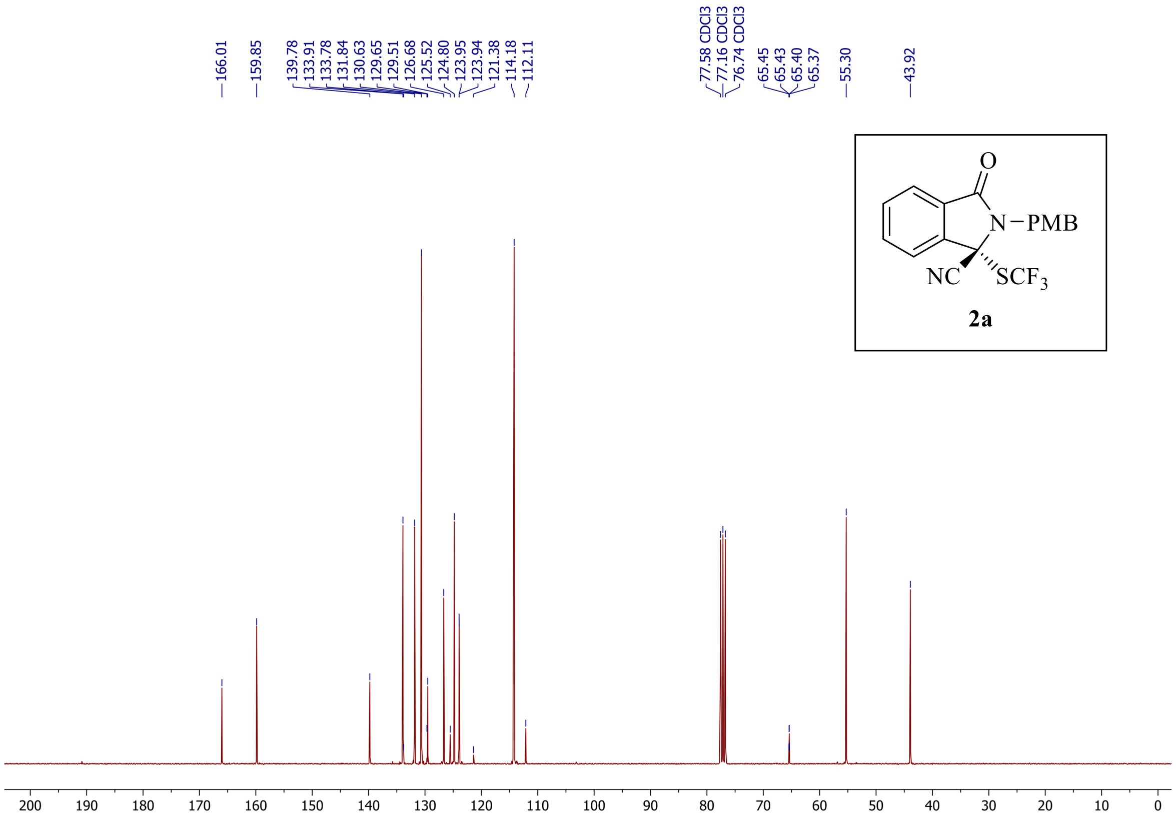

^{13}C-NMR (75 MHz, $CDCl_3$)

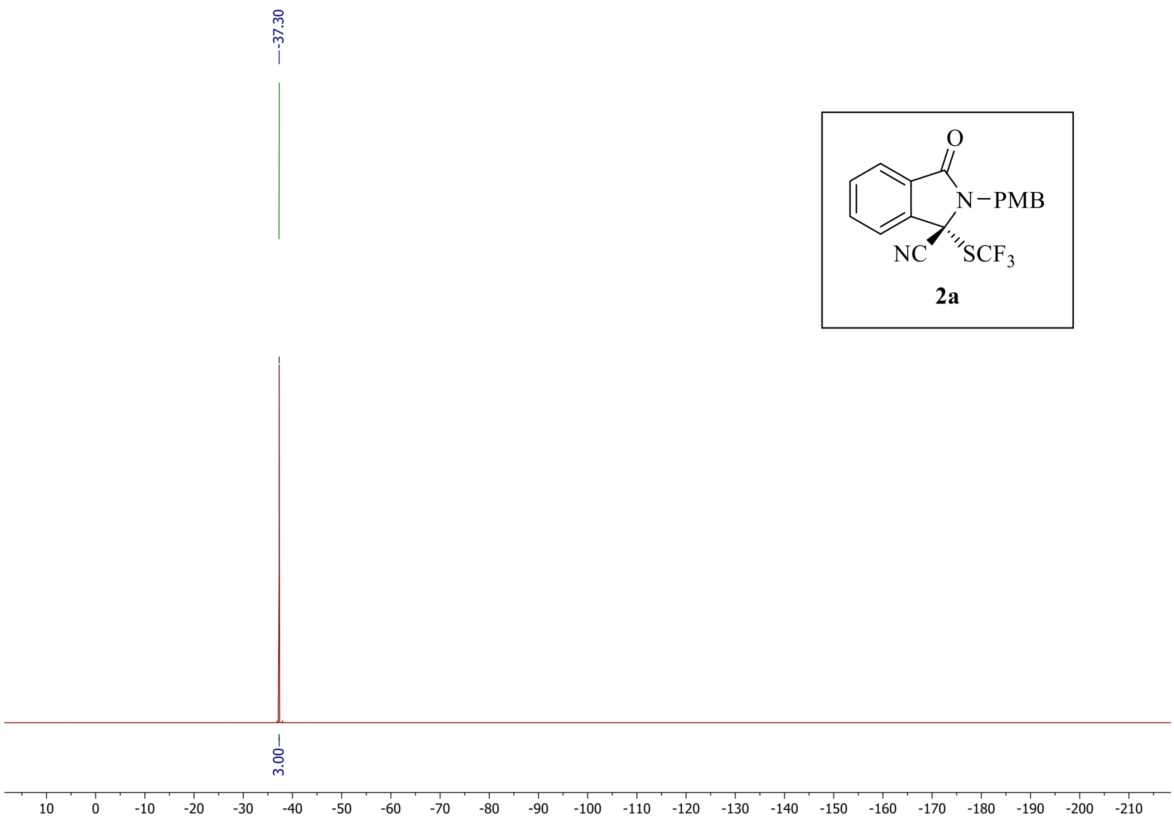

^{19}F-NMR (282 MHz, $CDCl_3$)

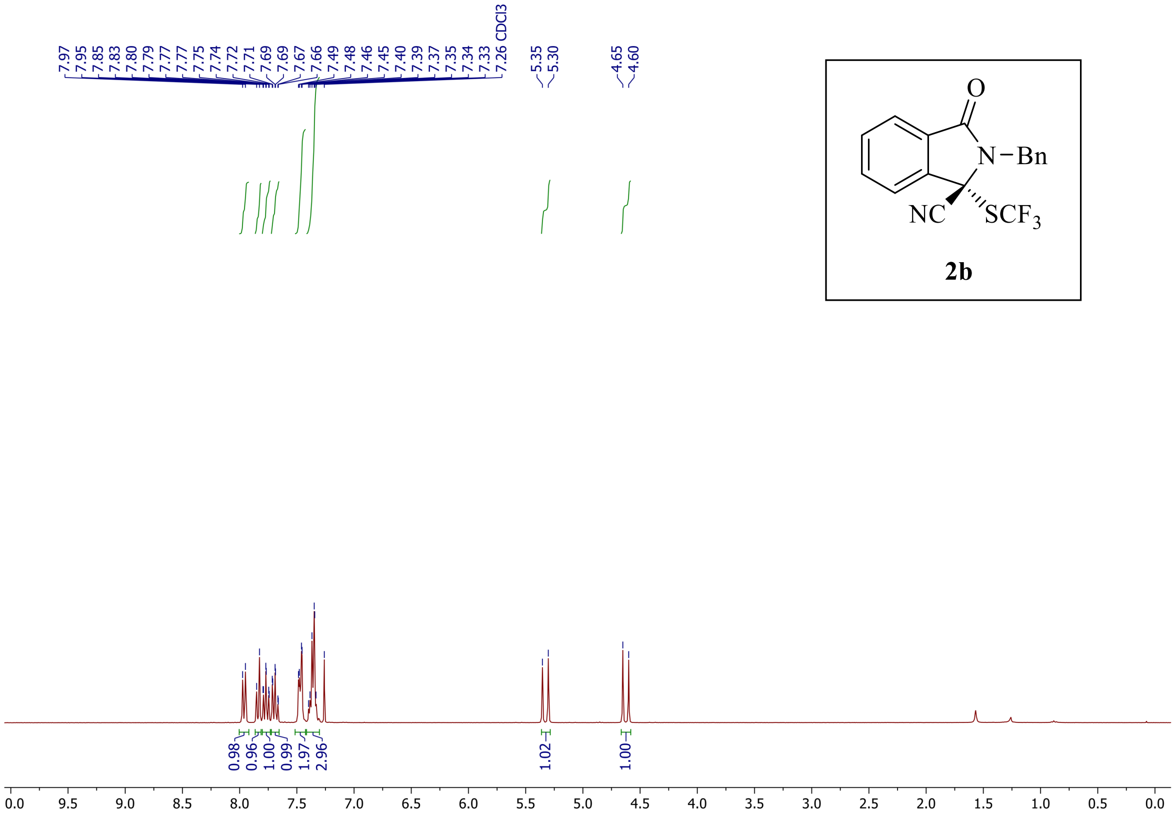

^{1}H-NMR (300 MHz, $CDCl_3$)

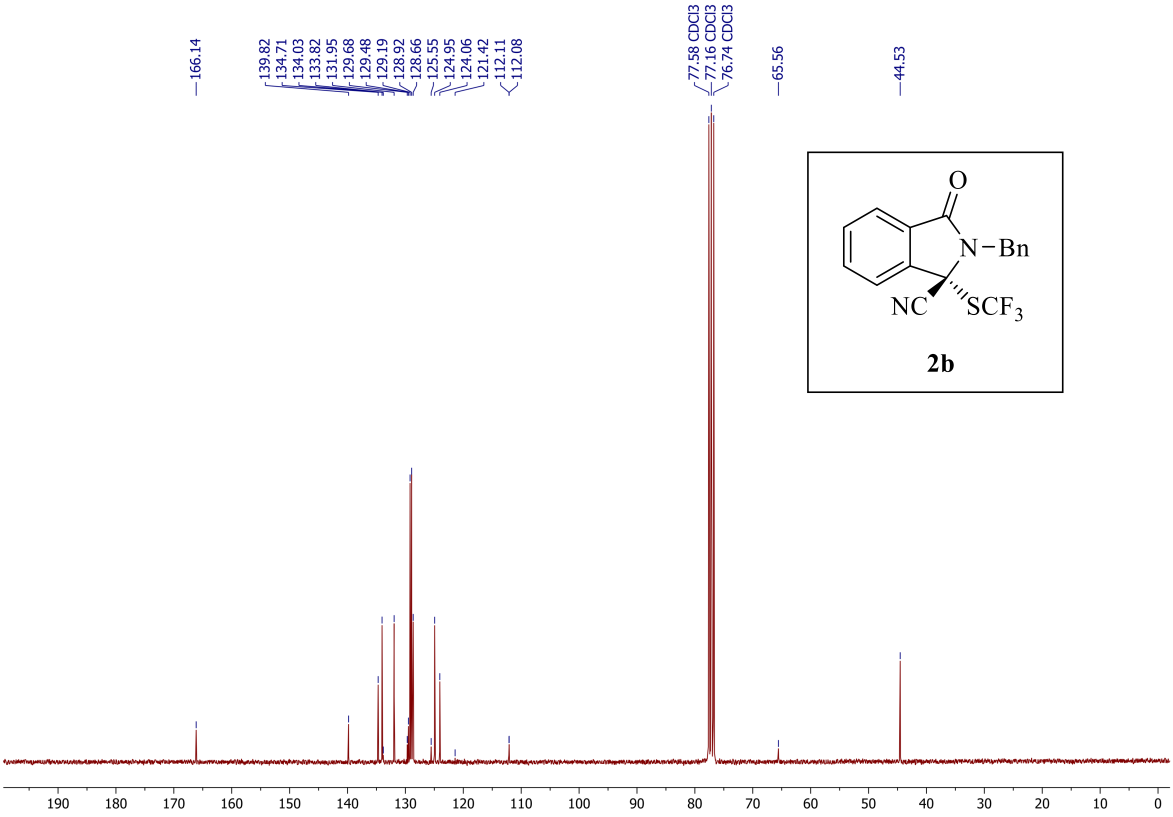

2b

^{13}C-NMR (75 MHz, $CDCl_3$)

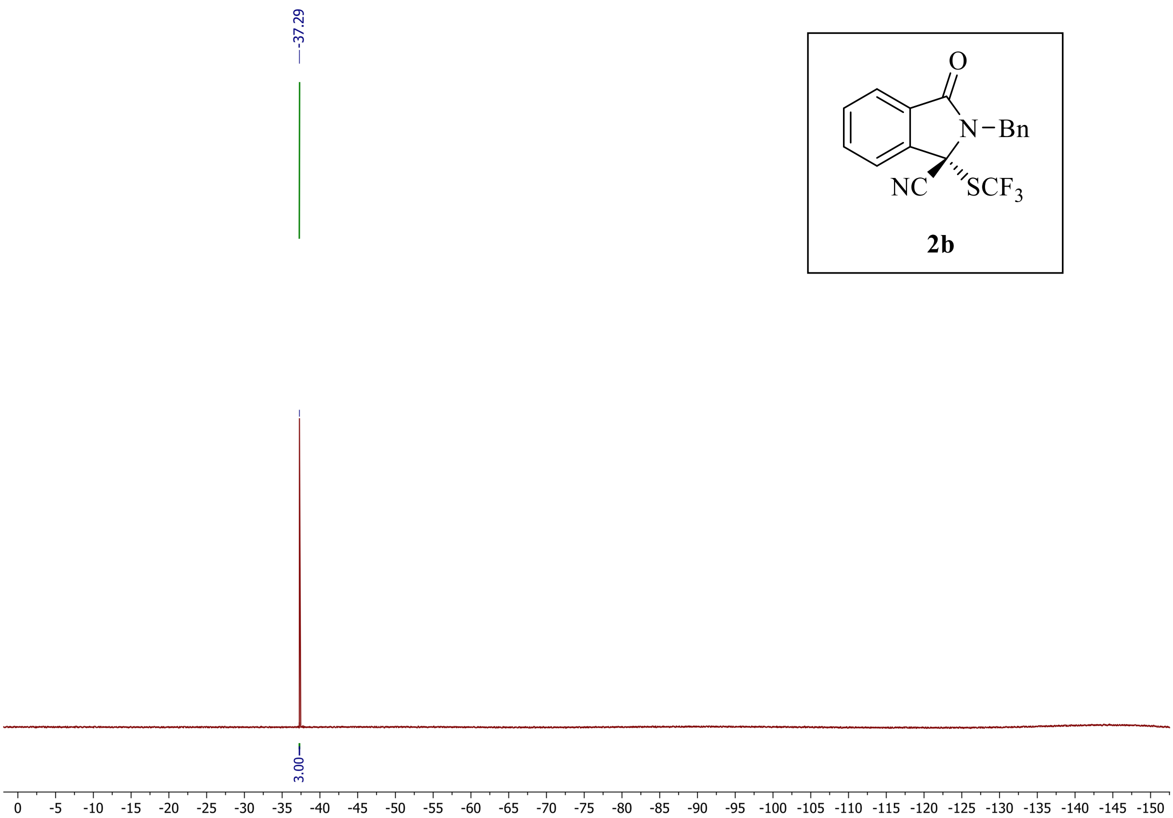

2b

^{19}F-NMR (471 MHz, $CDCl_3$)

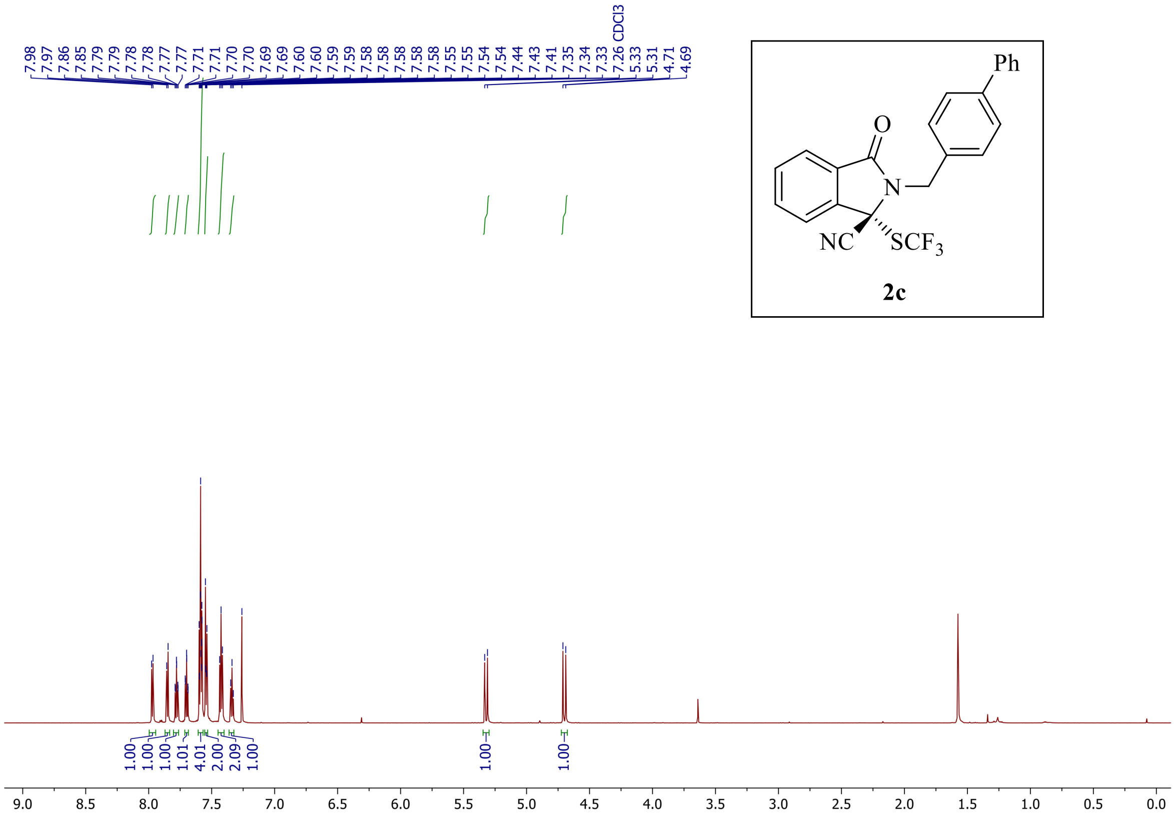

^{1}H-NMR (700 MHz, $CDCl_3$)

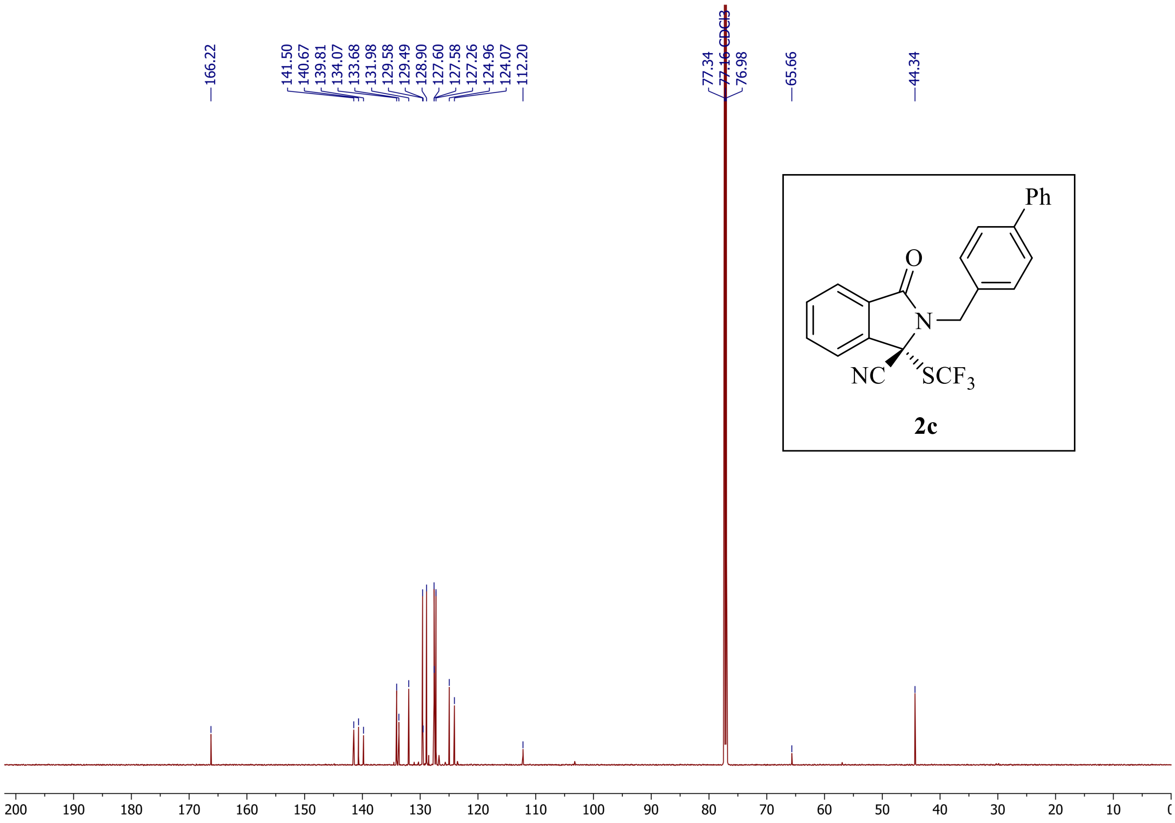

^{13}C-NMR (176 MHz, $CDCl_3$)

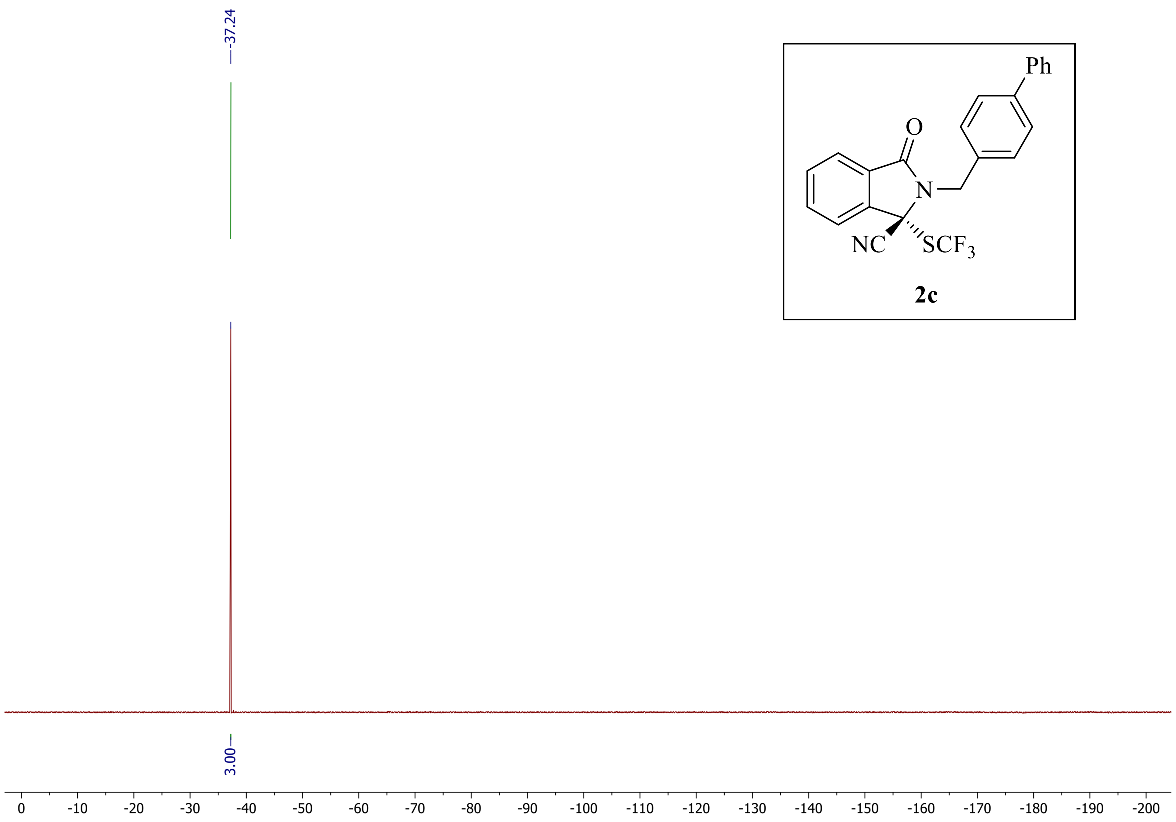

^{19}F-NMR (471 MHz, CDCl$_3$)

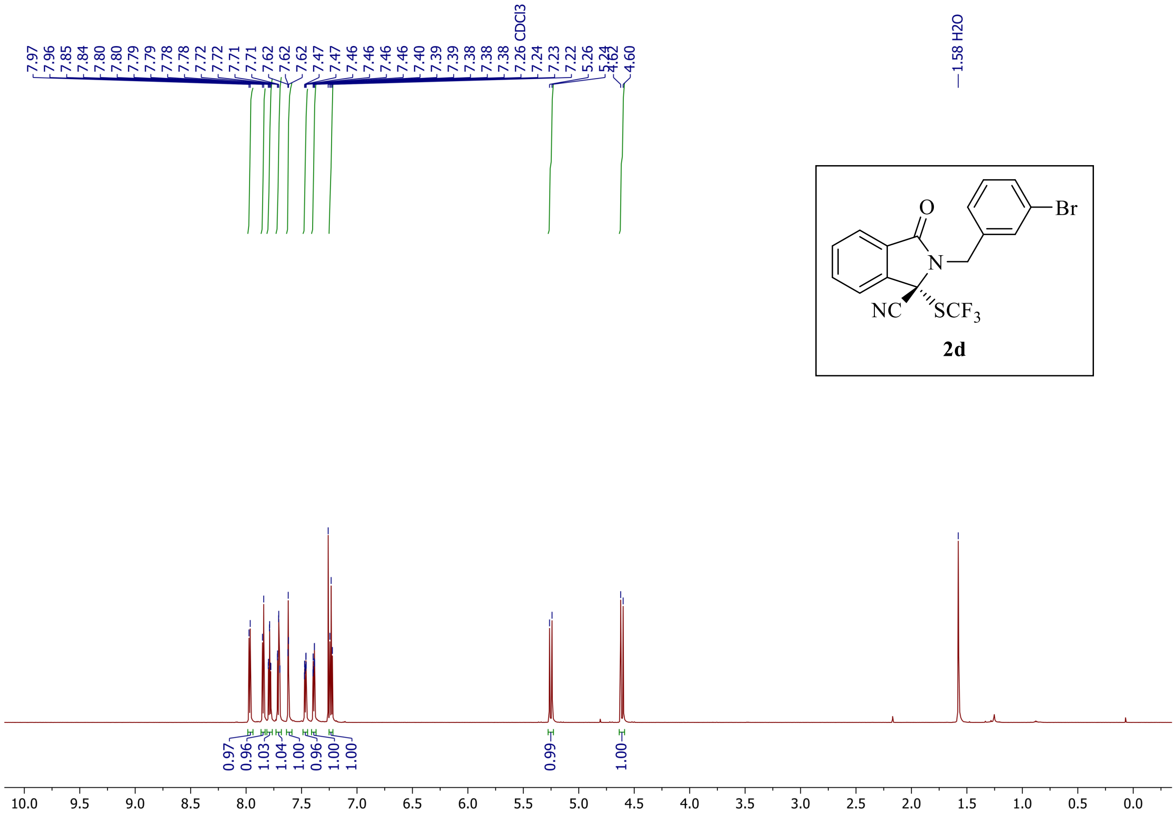

^{1}H-NMR (700 MHz, CDCl$_3$)

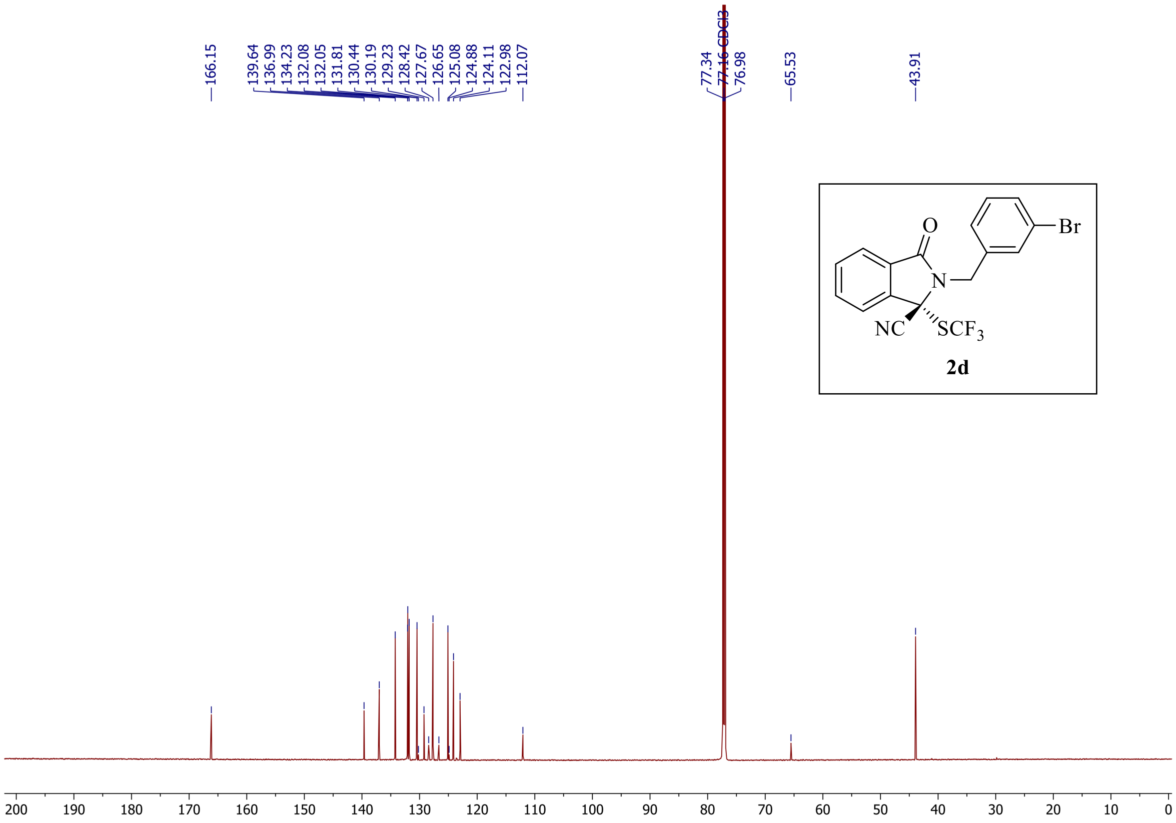

^{13}C-NMR (176 MHz, $CDCl_3$)

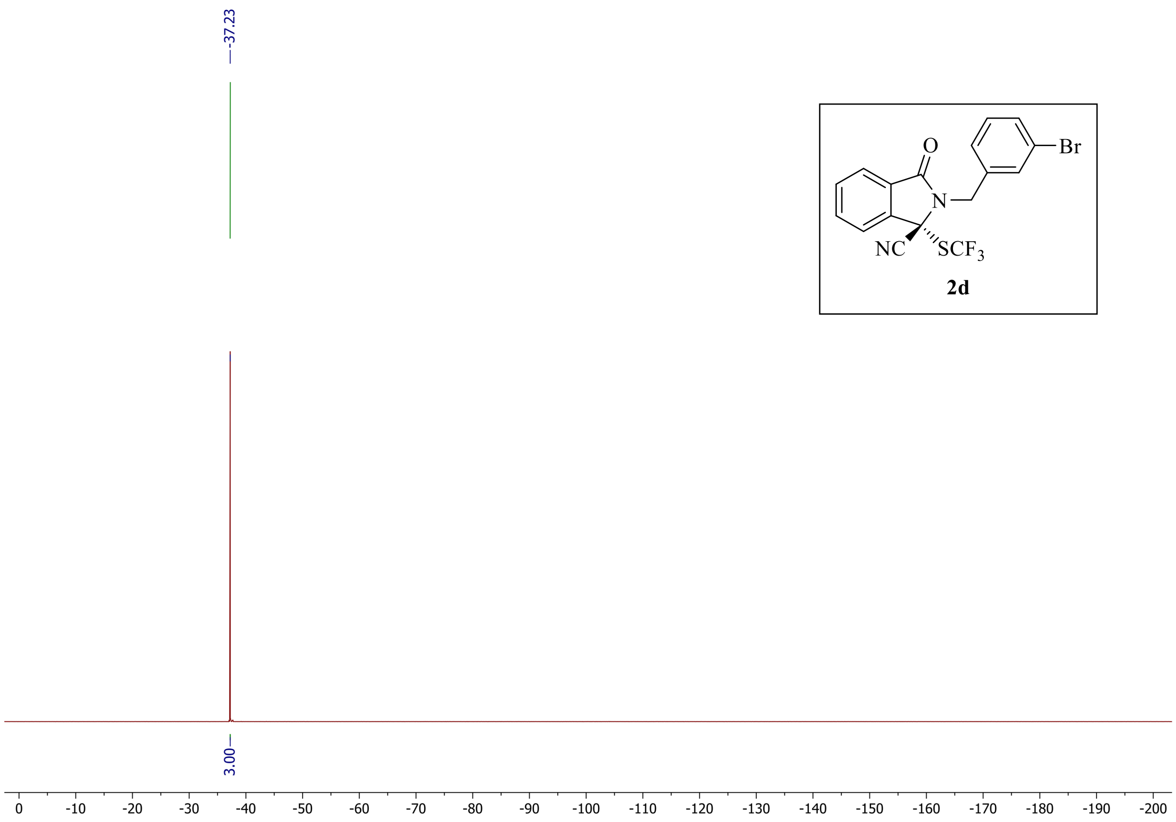

^{19}F-NMR (471 MHz, $CDCl_3$)

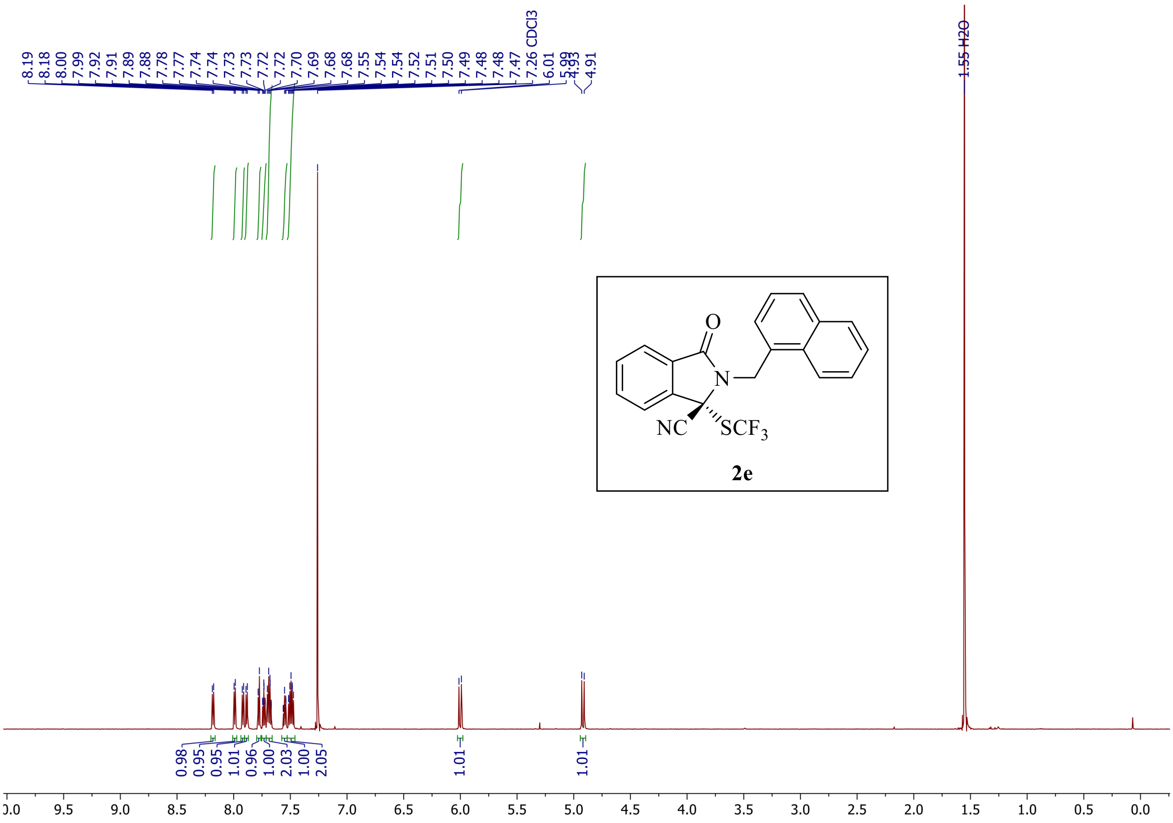

^{1}H-NMR (700 MHz, $CDCl_3$)

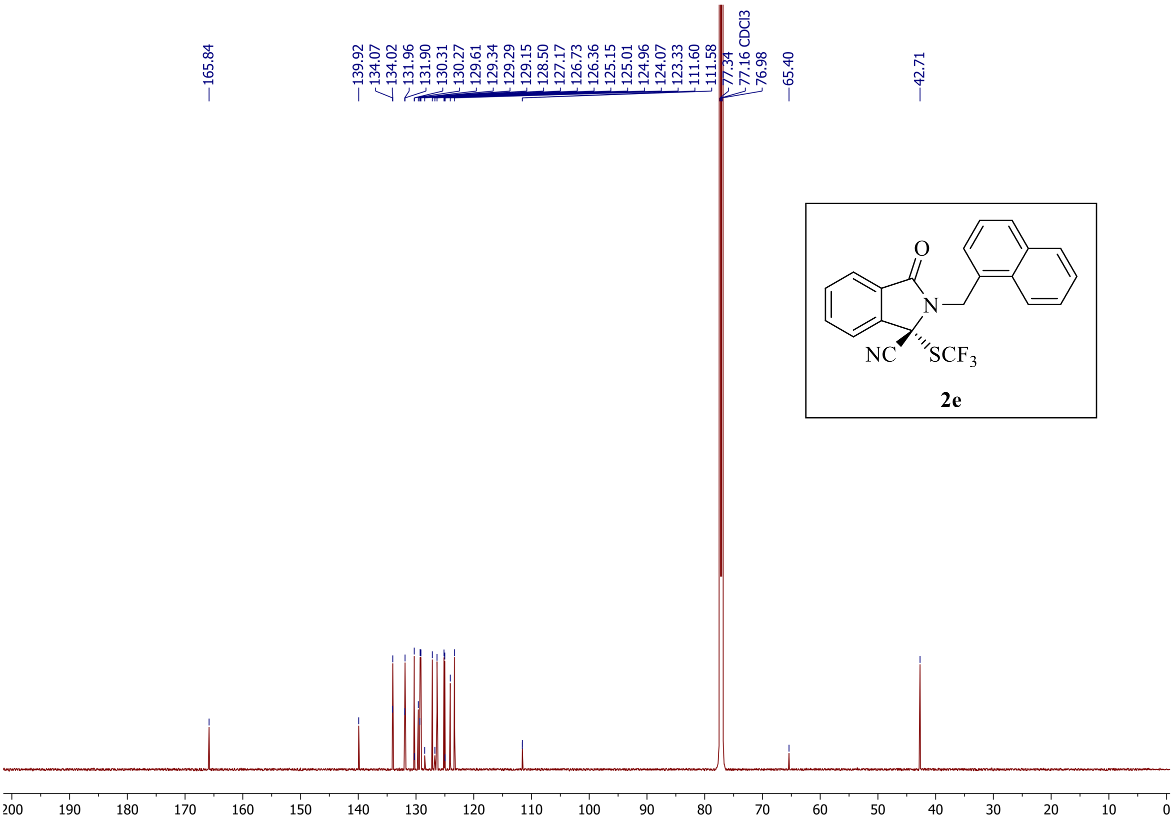

^{13}C-NMR (176 MHz, $CDCl_3$)

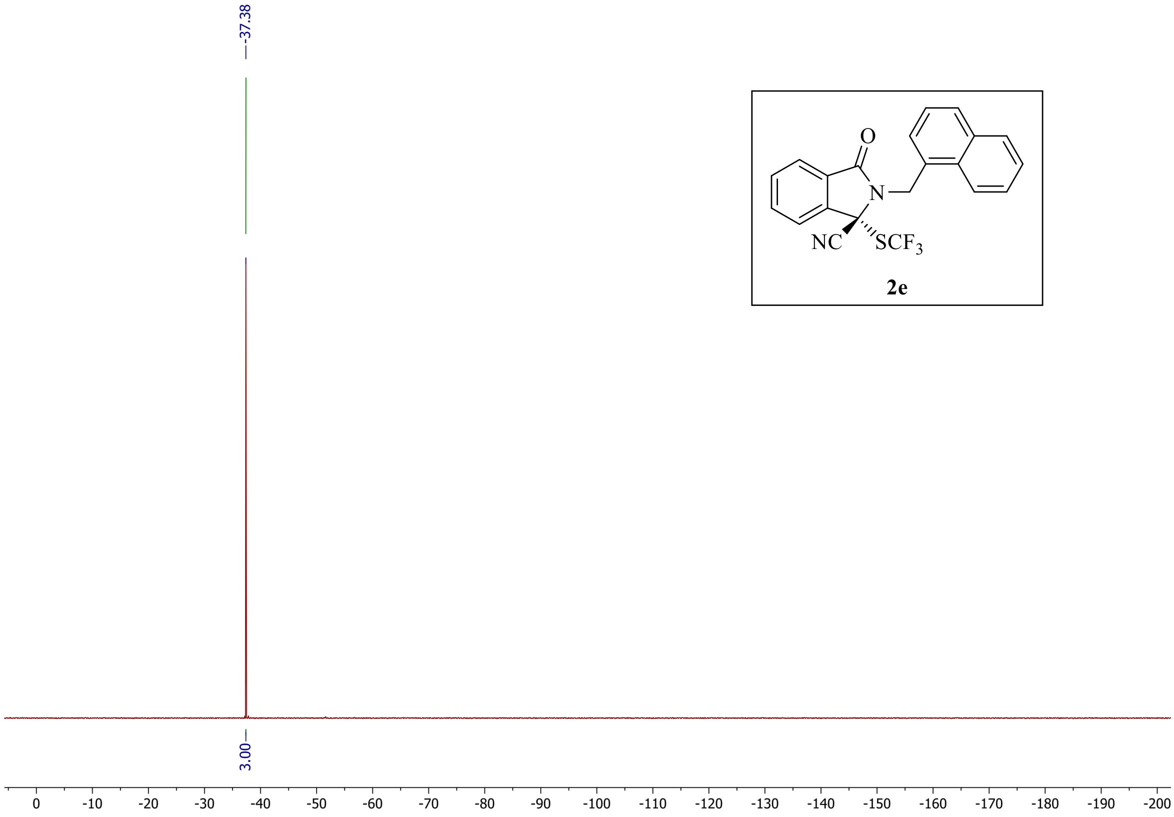

2e

^{19}F-NMR (471 MHz, $CDCl_3$)

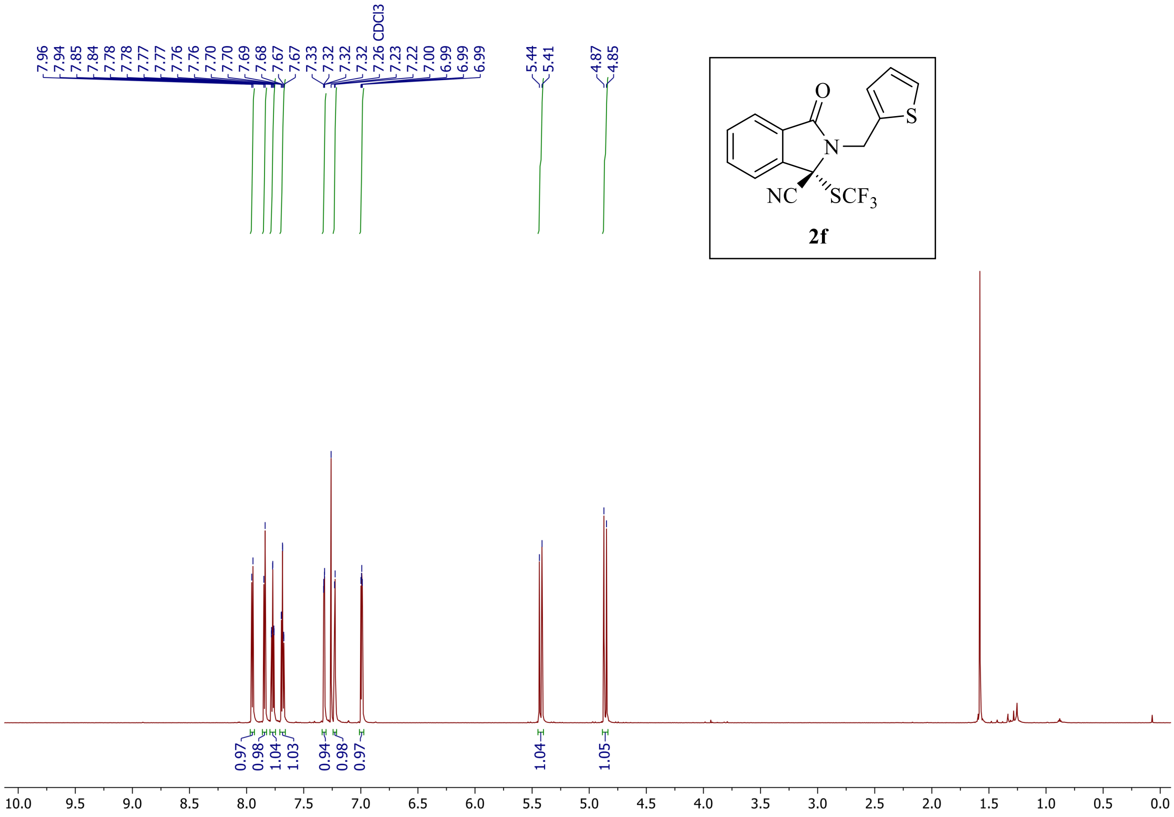

2f

^{1}H-NMR (700 MHz, $CDCl_3$)

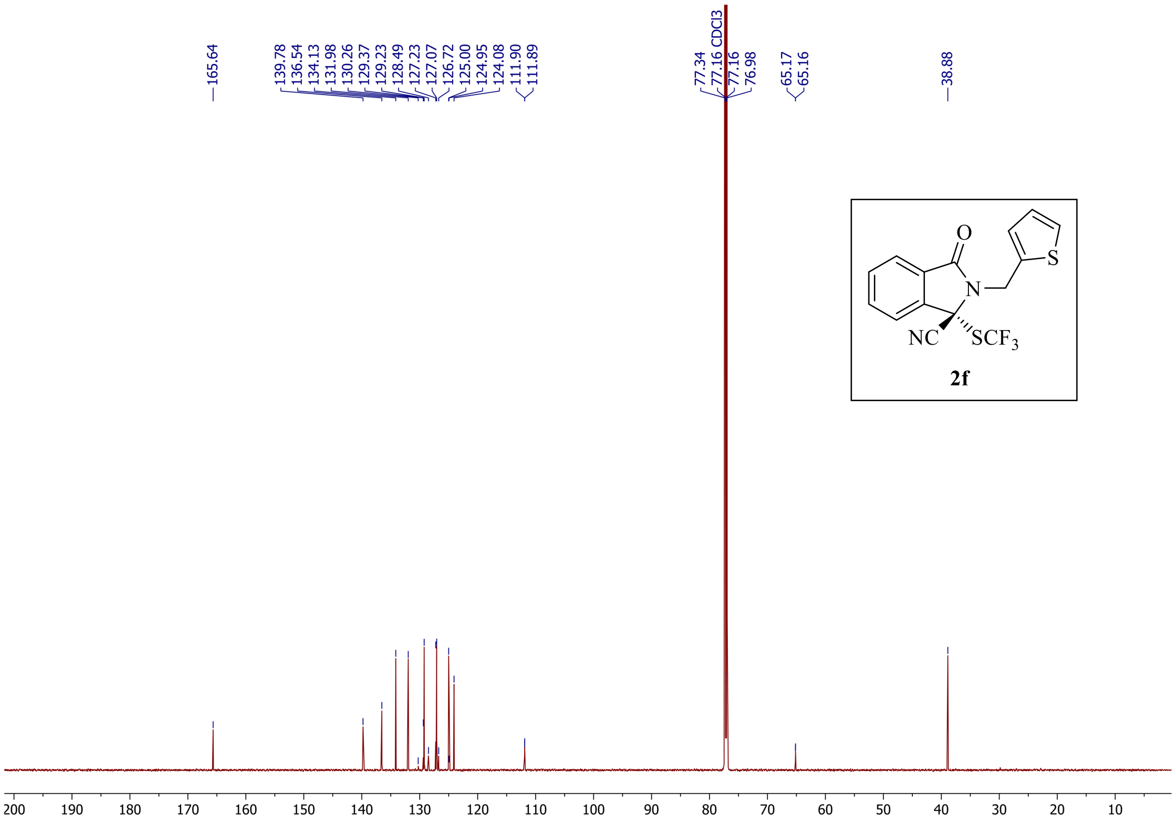

^{13}C-NMR (176 MHz, $CDCl_3$)

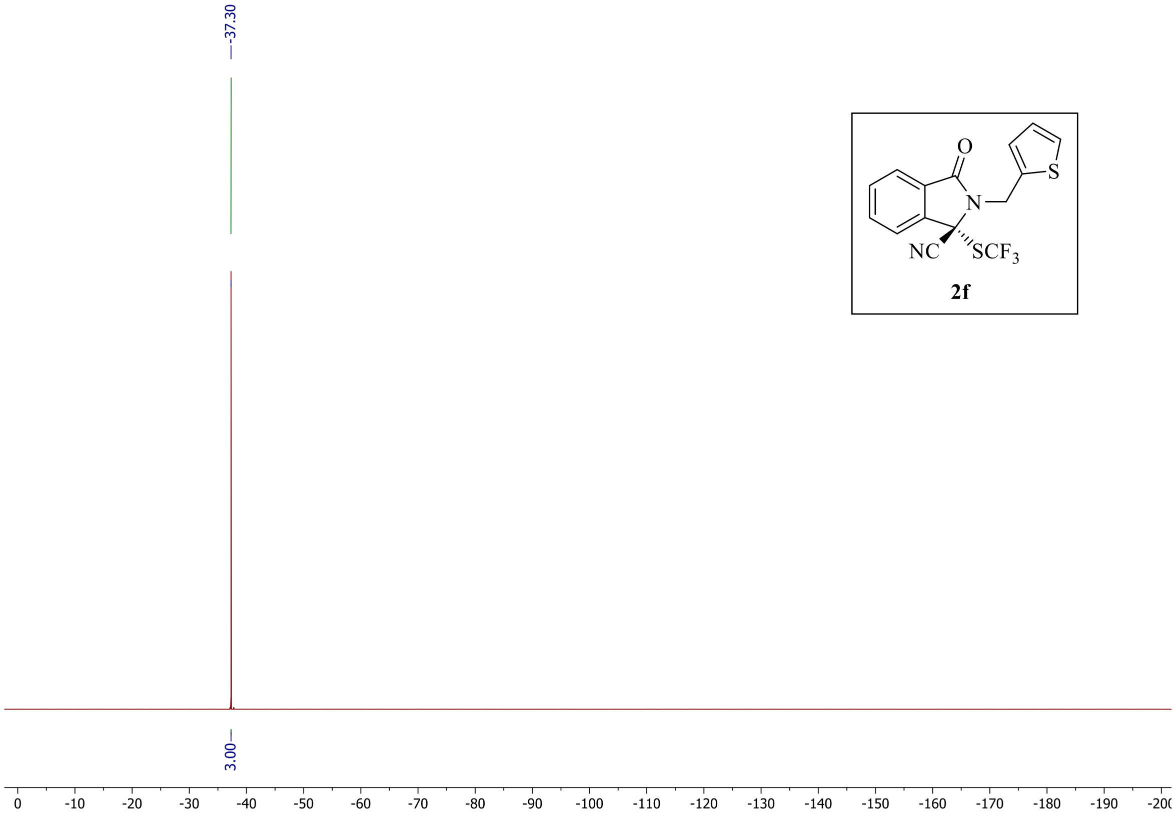

^{19}F-NMR (471 MHz, $CDCl_3$)

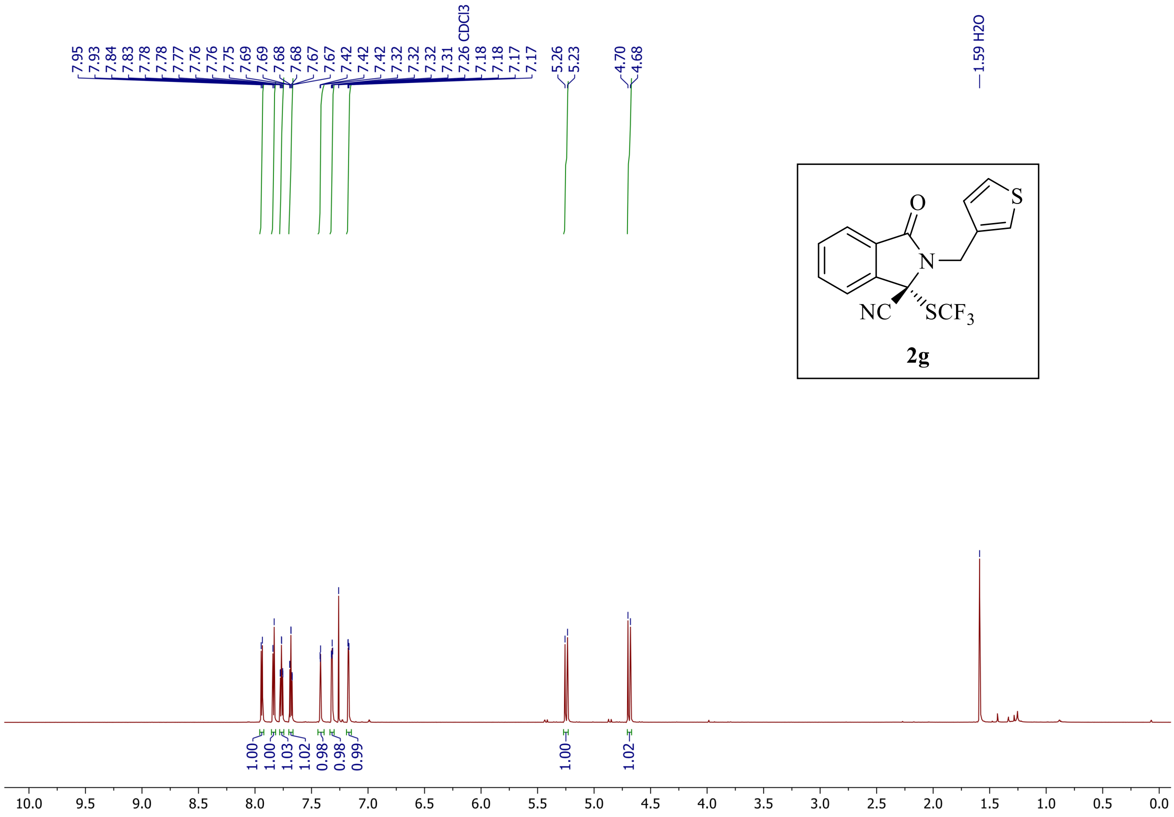

^{1}H-NMR (700 MHz, $CDCl_3$)

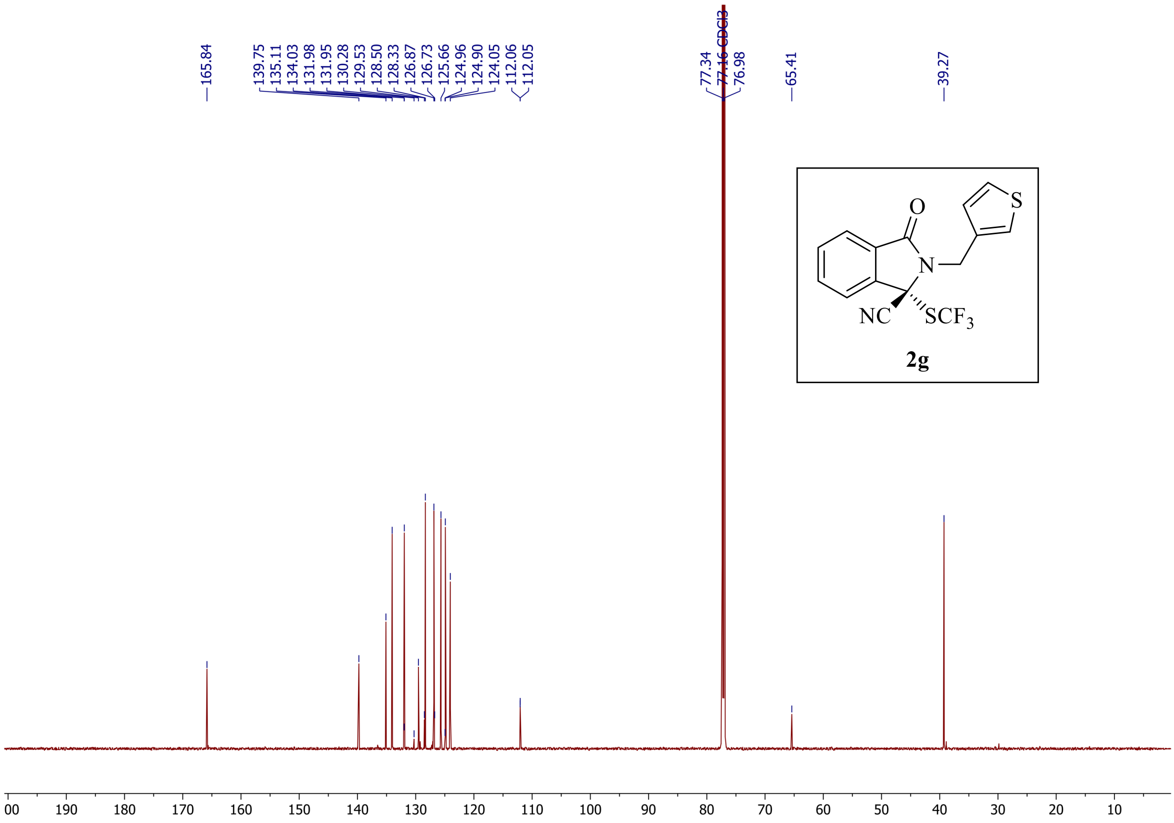

^{13}C-NMR (176 MHz, $CDCl_3$)

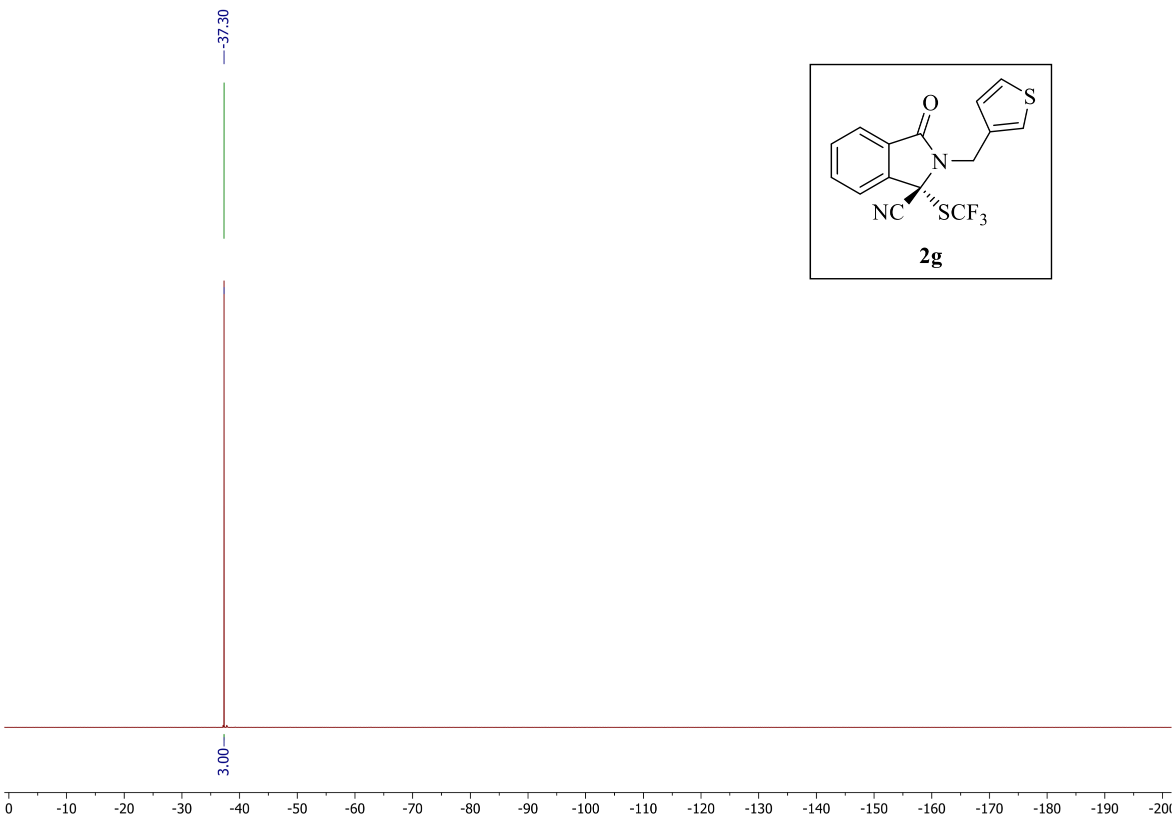

2g

^{19}F-NMR (471 MHz, $CDCl_3$)

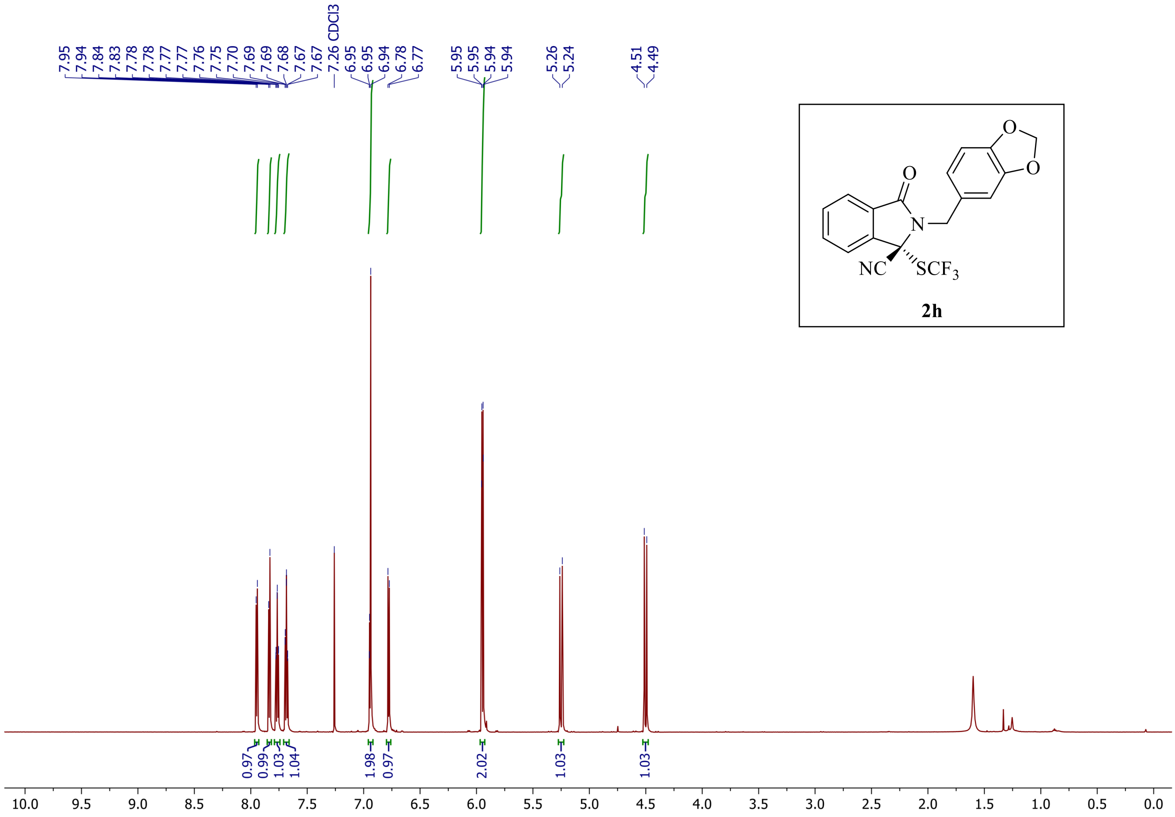

2h

^{1}H-NMR (700 MHz, $CDCl_3$)

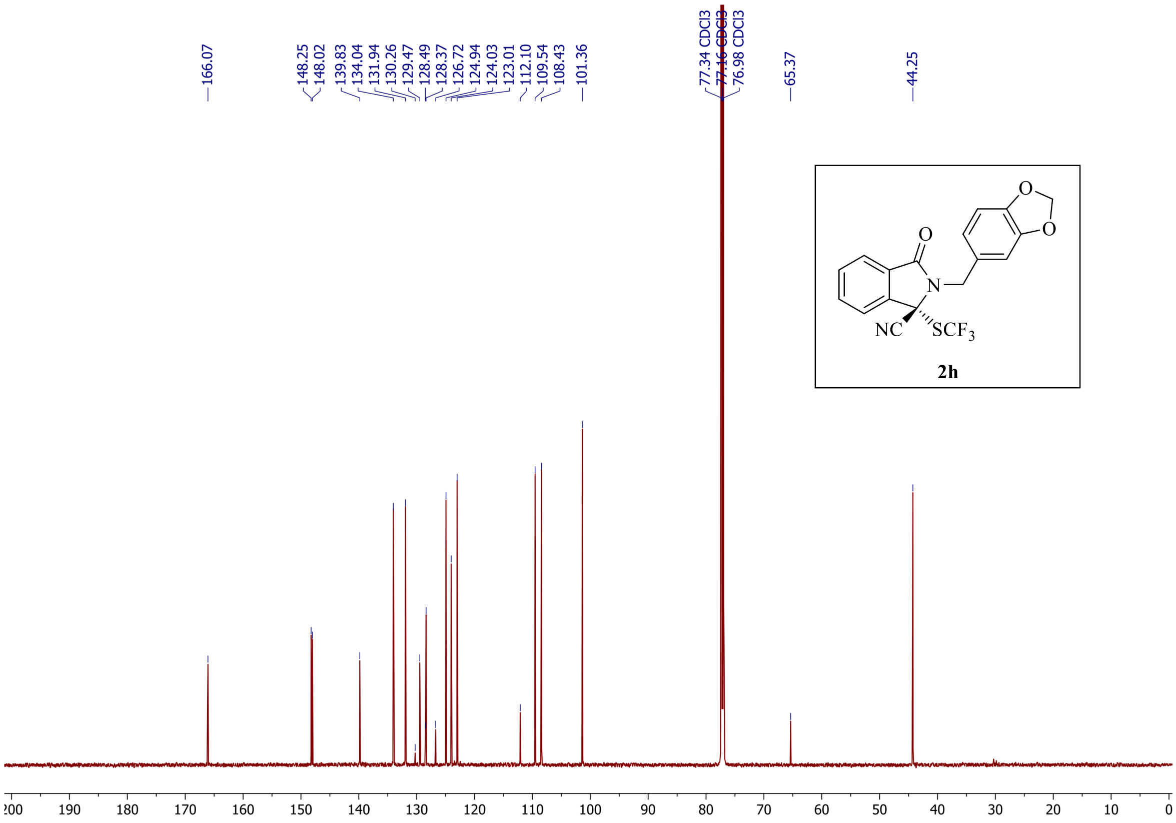

^{13}C-NMR (176 MHz, CDCl$_3$)

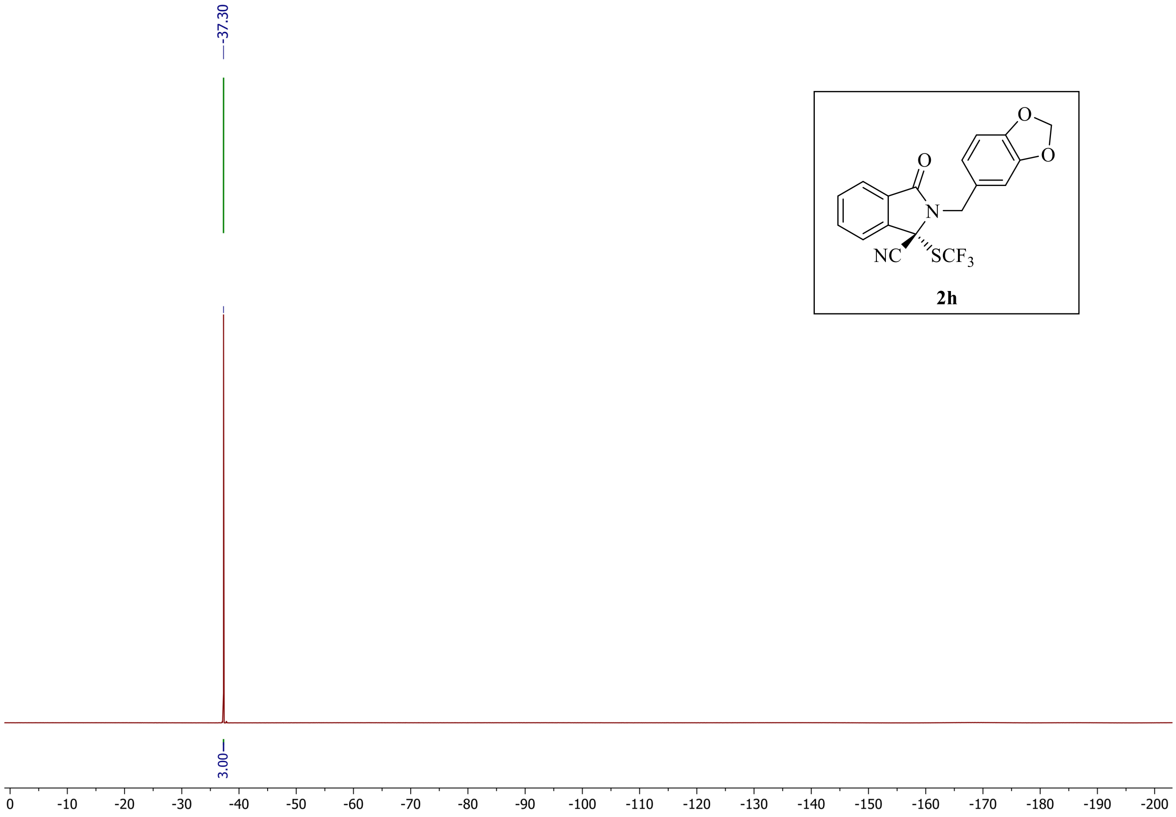

^{19}F-NMR (471 MHz, CDCl$_3$)

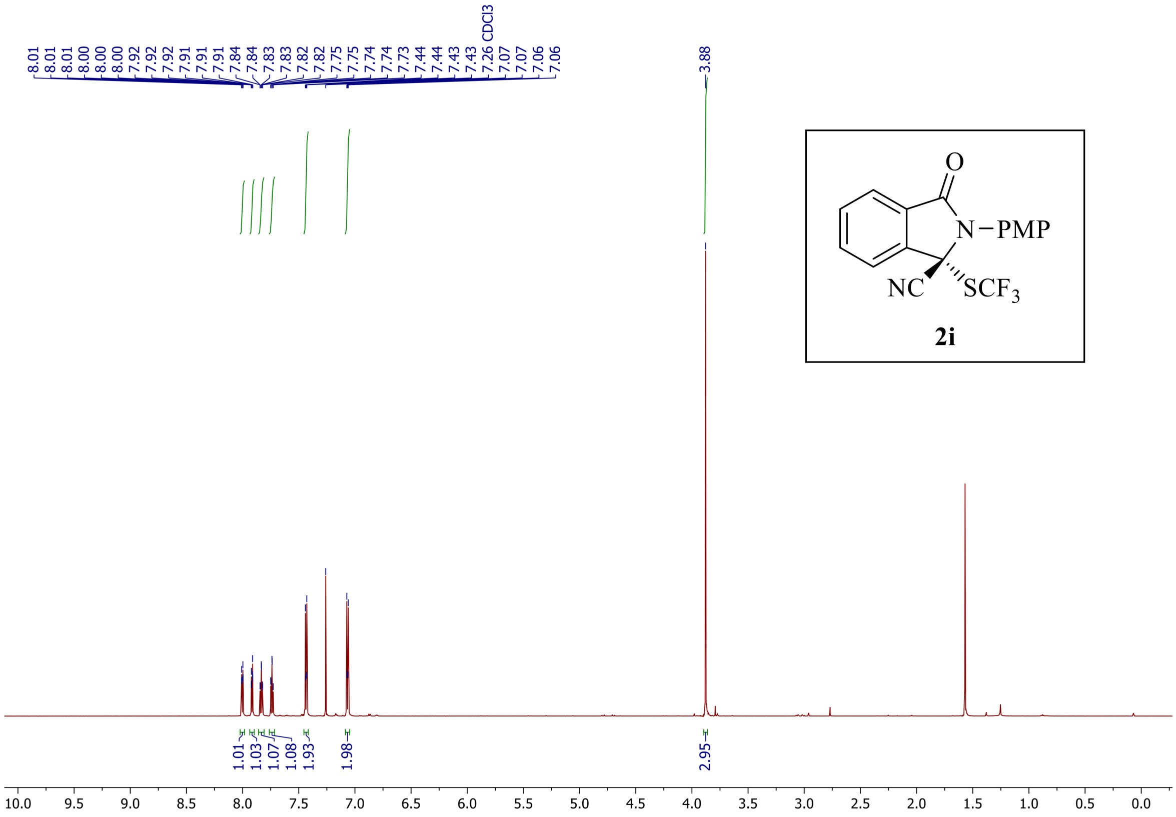

^{1}H-NMR (700 MHz, $CDCl_3$)

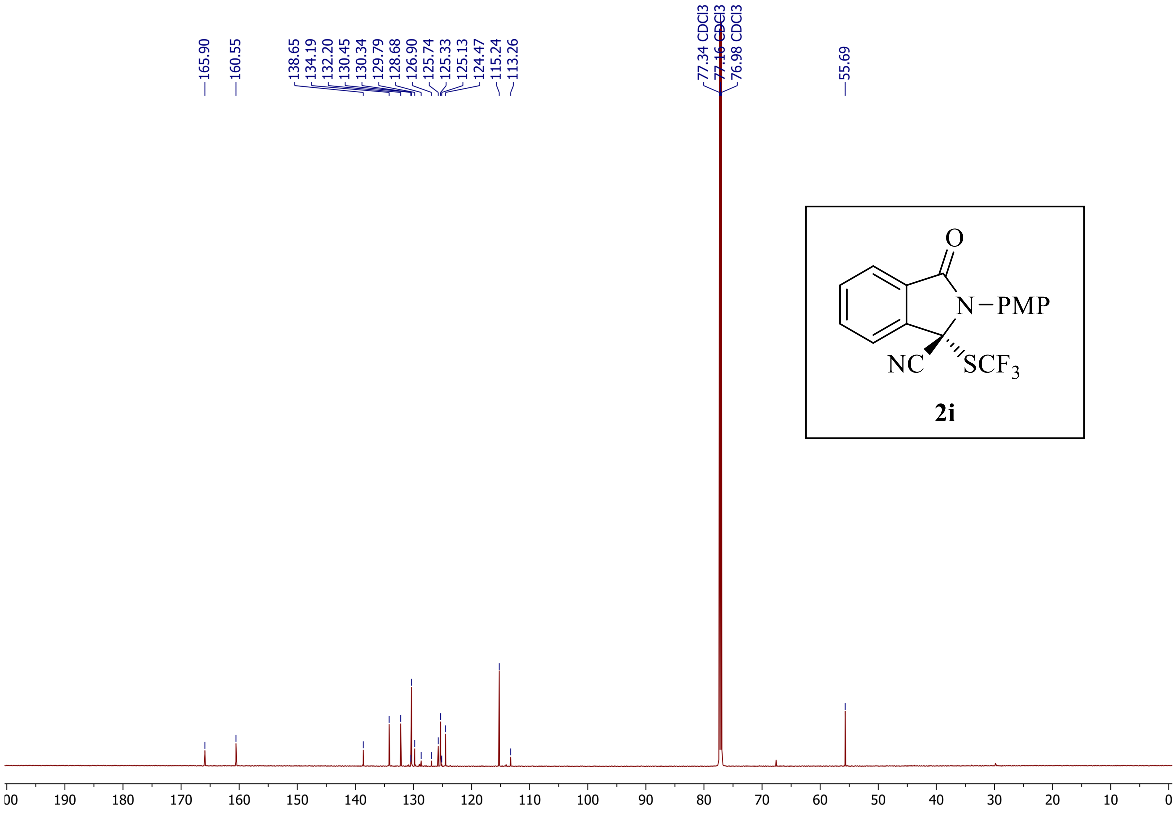

^{13}C-NMR (176 MHz, $CDCl_3$)

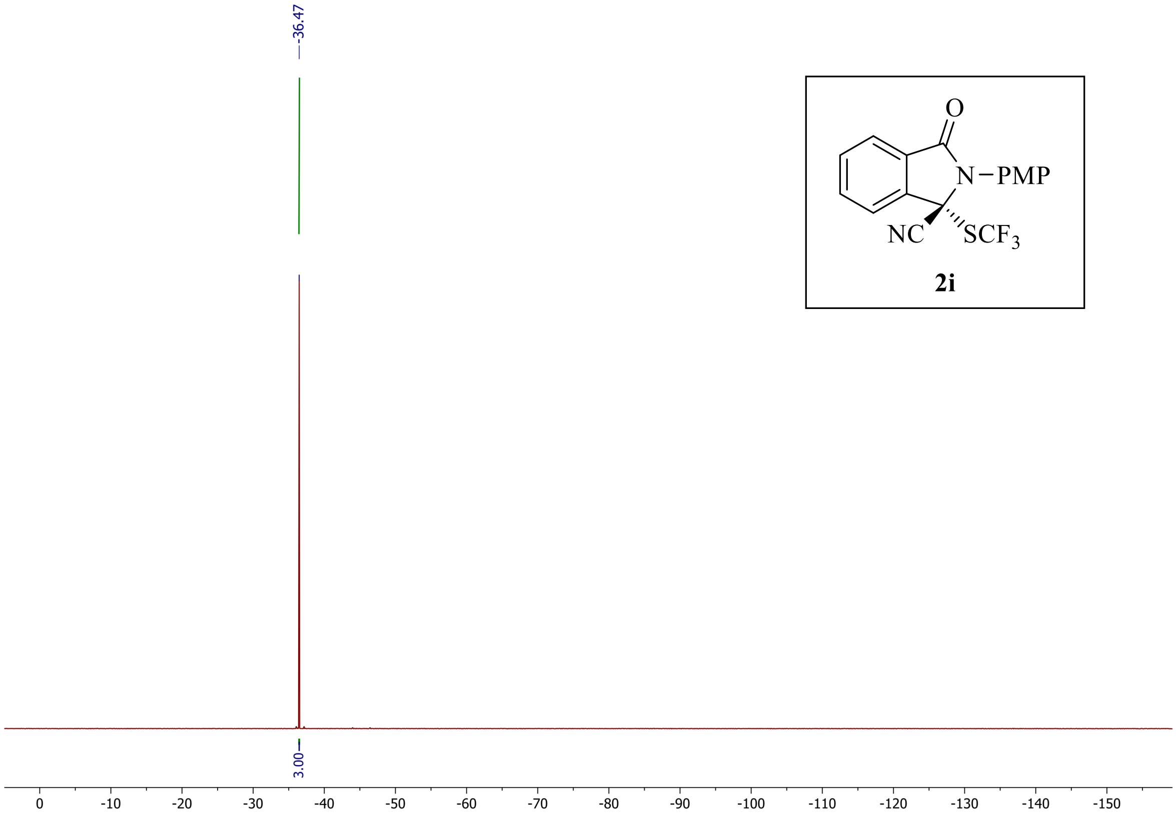

^{19}F-NMR (282 MHz, $CDCl_3$)

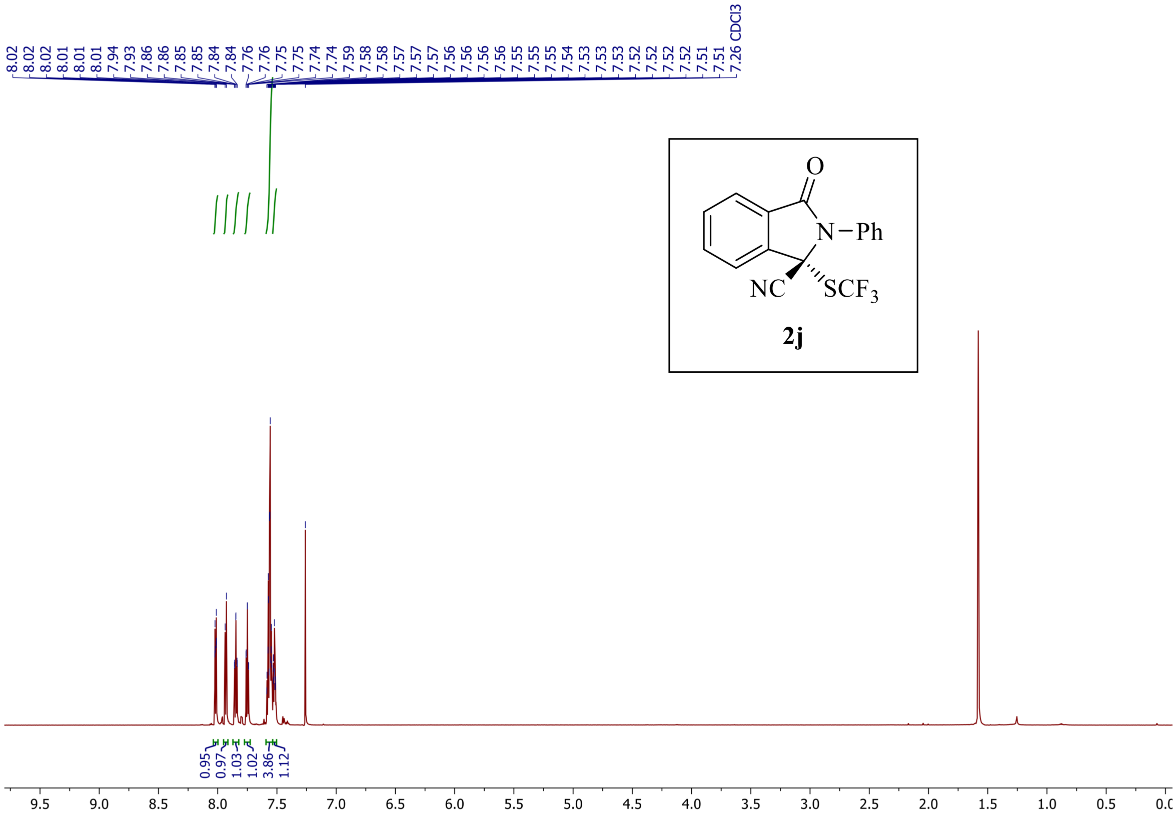

^{1}H-NMR (700 MHz, $CDCl_3$)

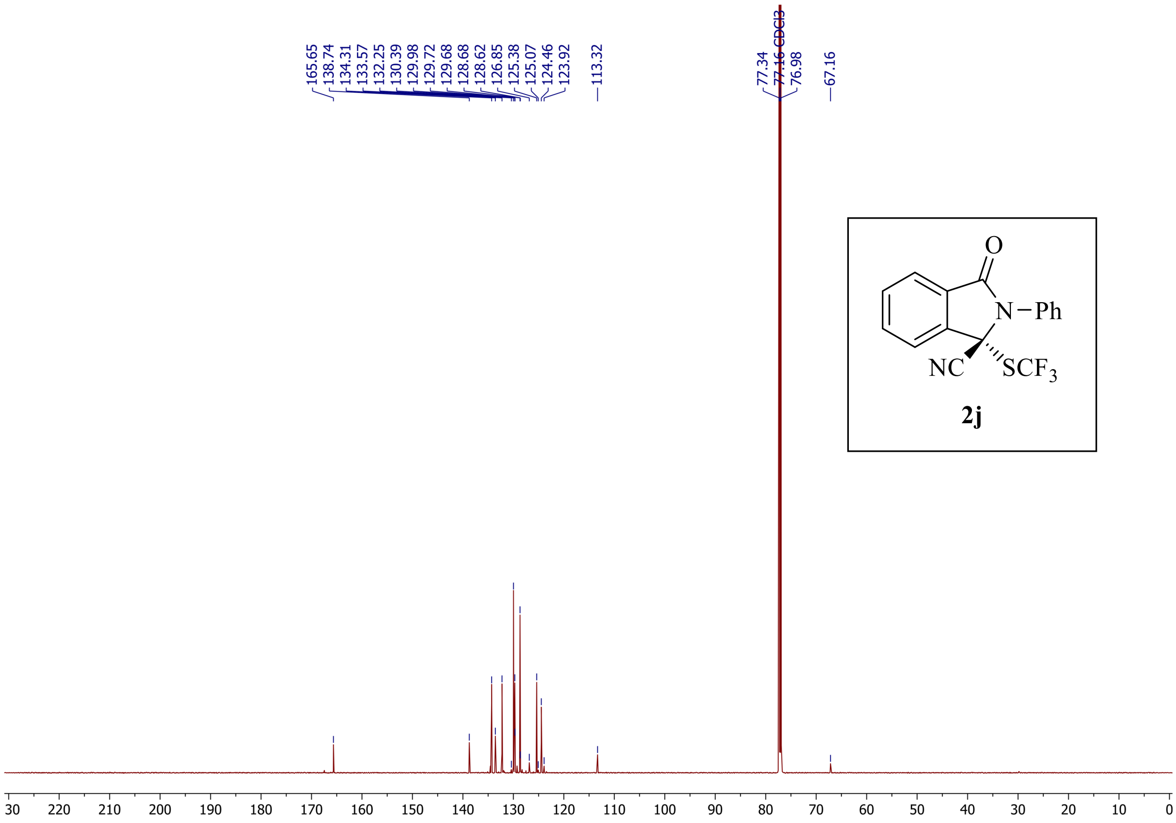

^{13}C-NMR (176 MHz, $CDCl_3$)

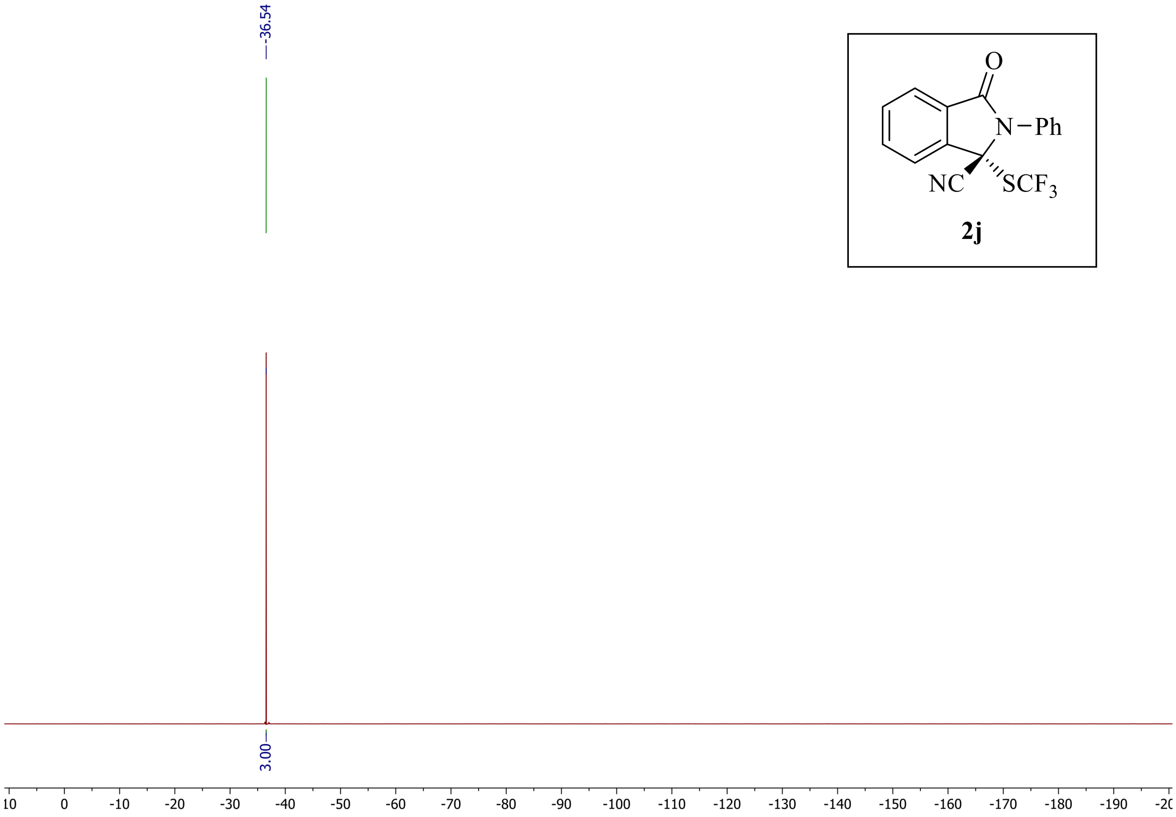

^{19}F-NMR (471 MHz, $CDCl_3$)

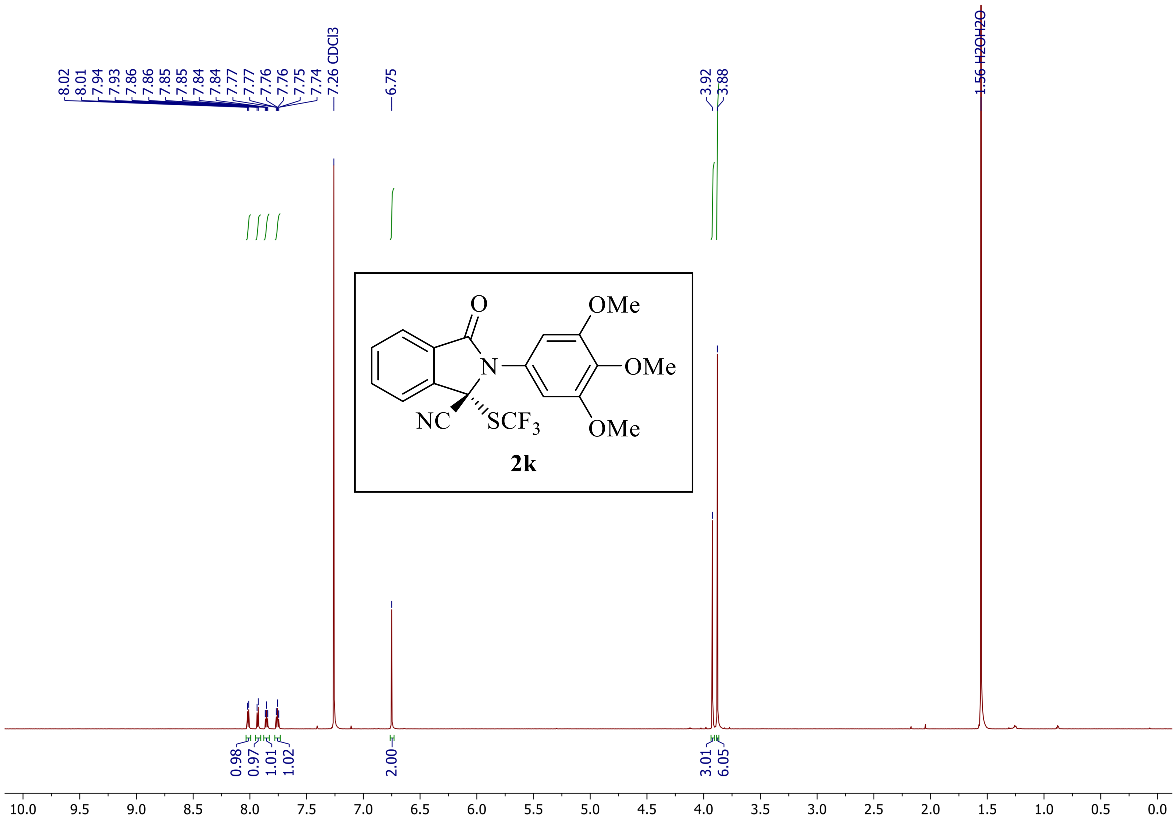

^{1}H-NMR (700 MHz, $CDCl_3$)

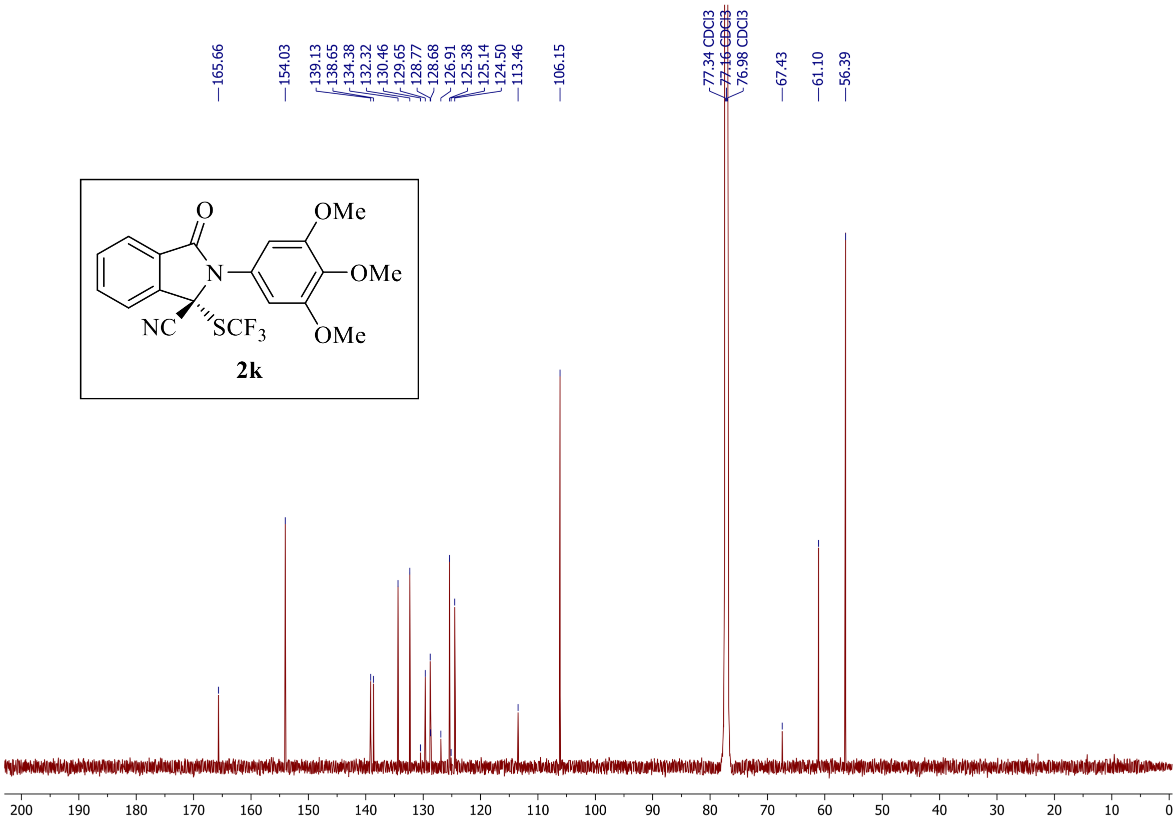

^{13}C-NMR (176 MHz, $CDCl_3$)

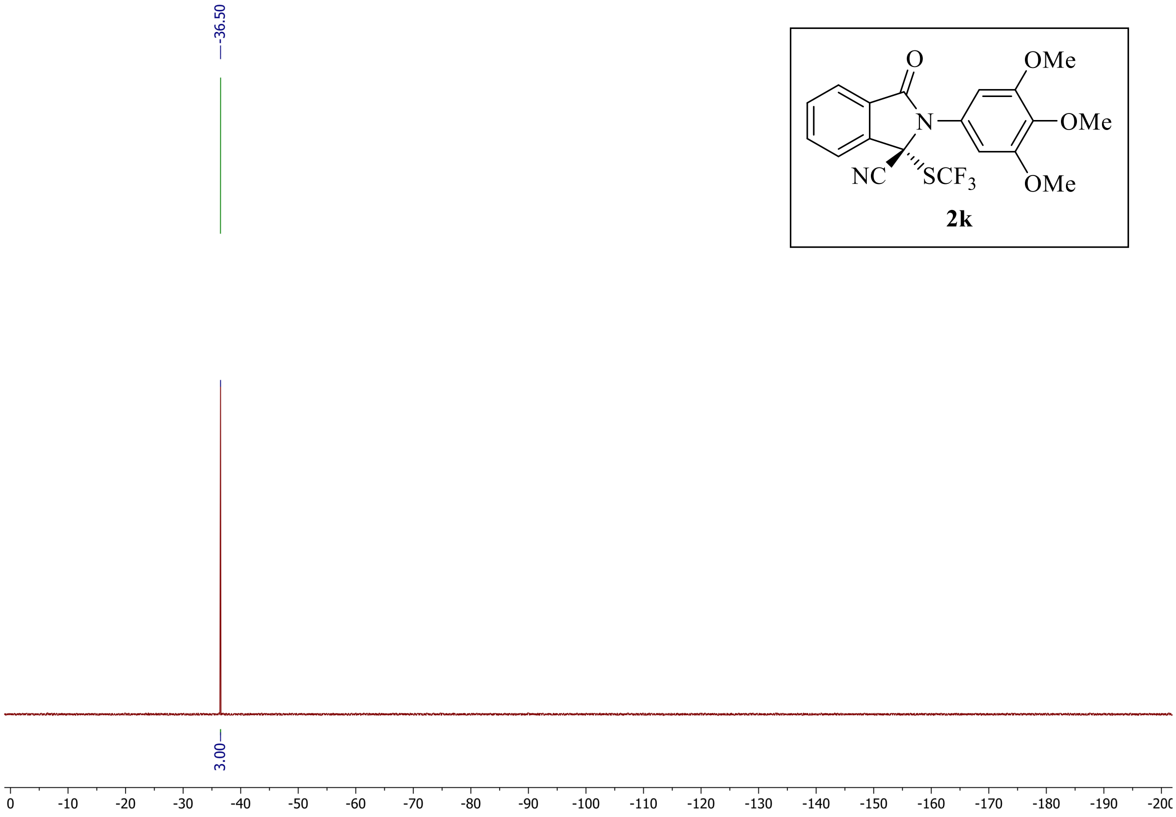

^{19}F-NMR (471 MHz, $CDCl_3$)

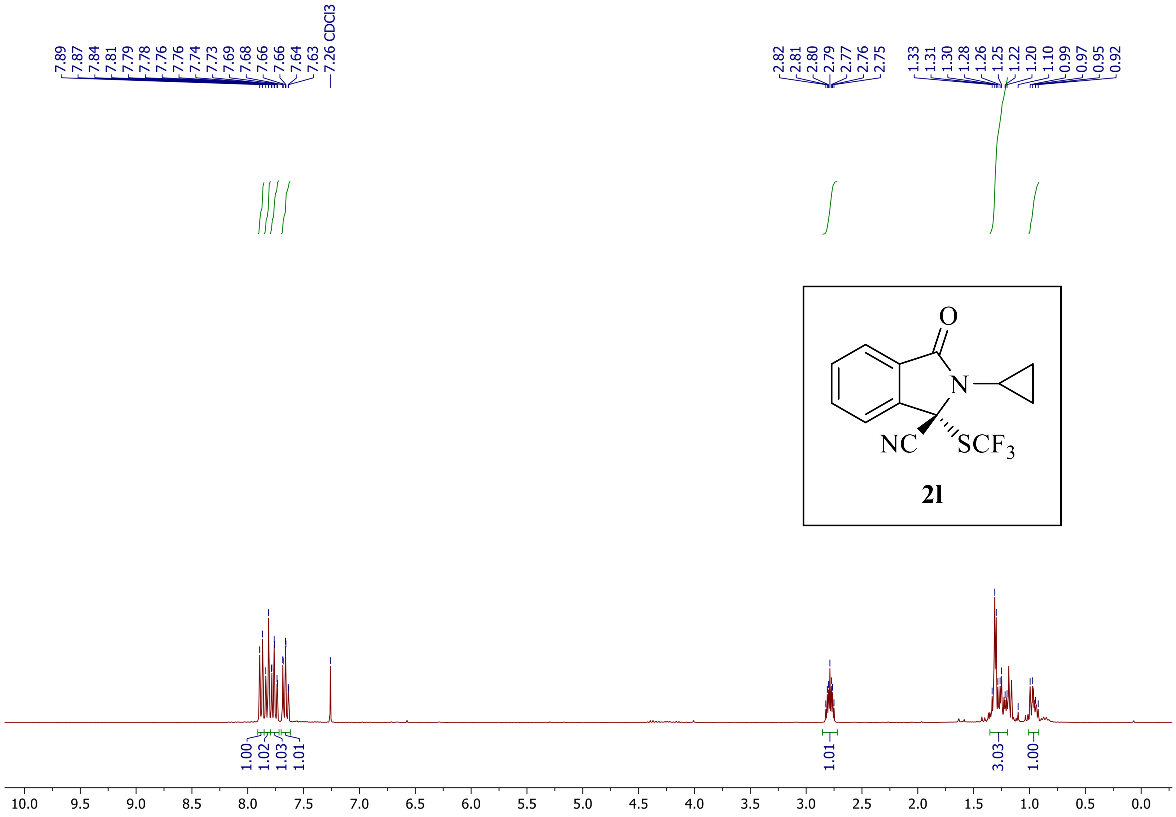

^{1}H-NMR (300 MHz, $CDCl_3$)

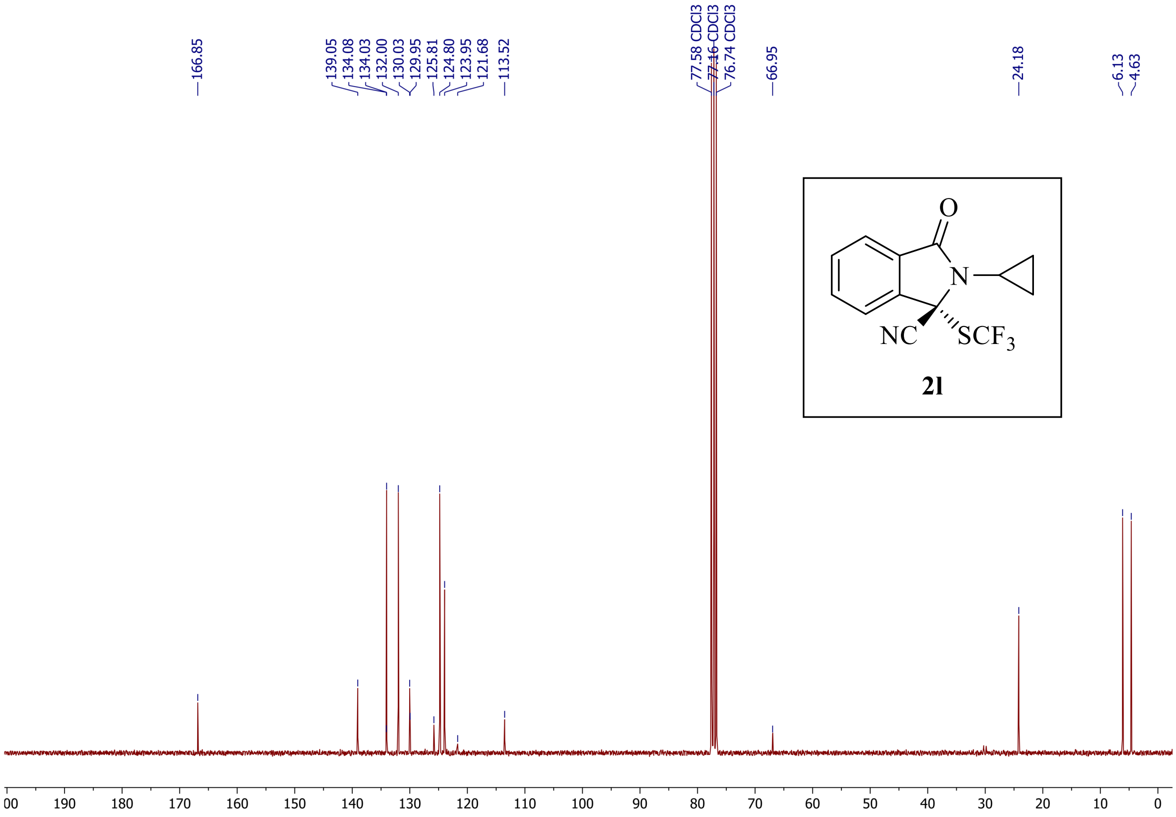

^{13}C-NMR (75 MHz, $CDCl_3$)

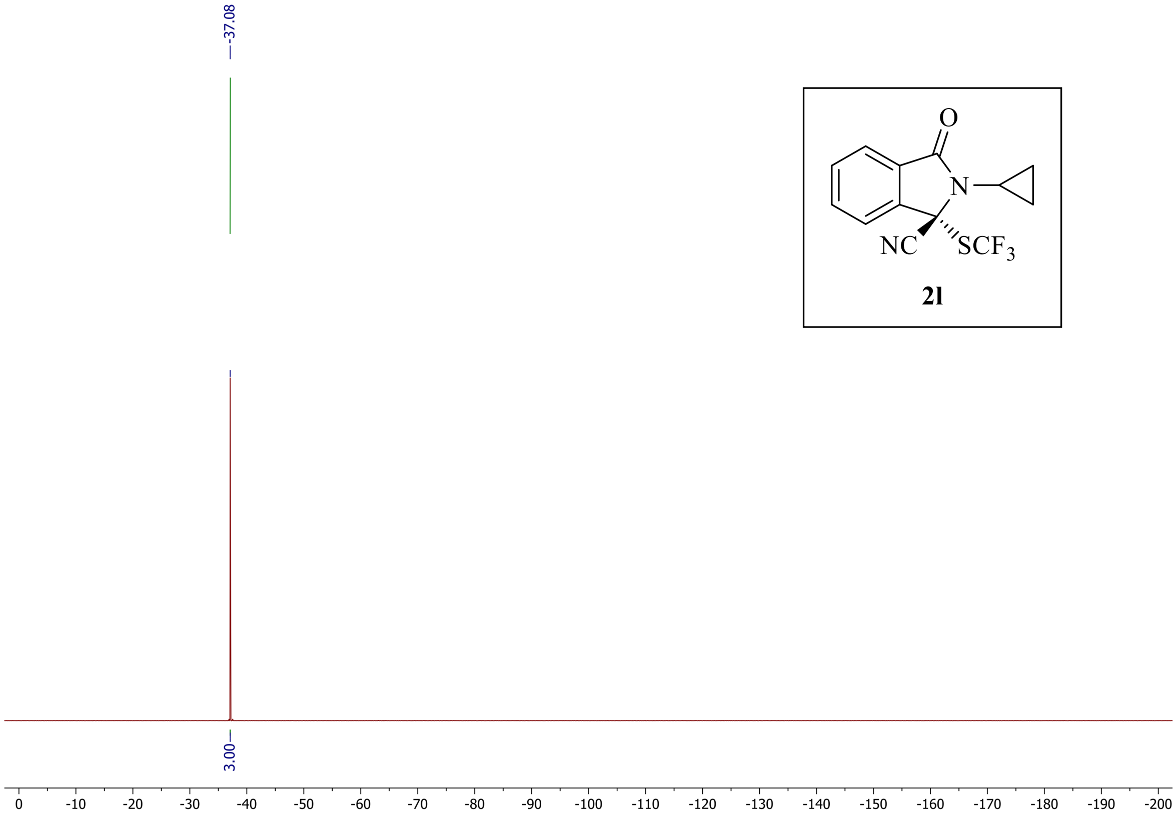

^{19}F-NMR (471 MHz, $CDCl_3$)

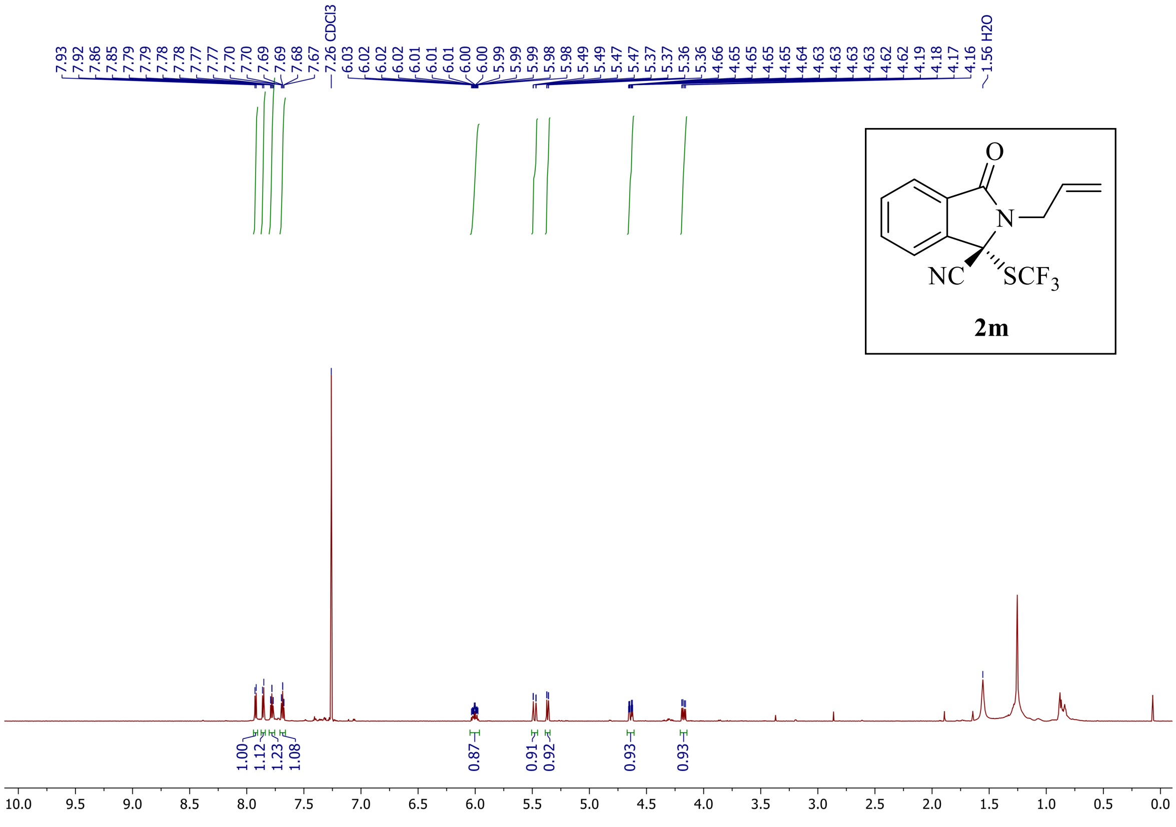

^{1}H-NMR (300 MHz, $CDCl_3$)

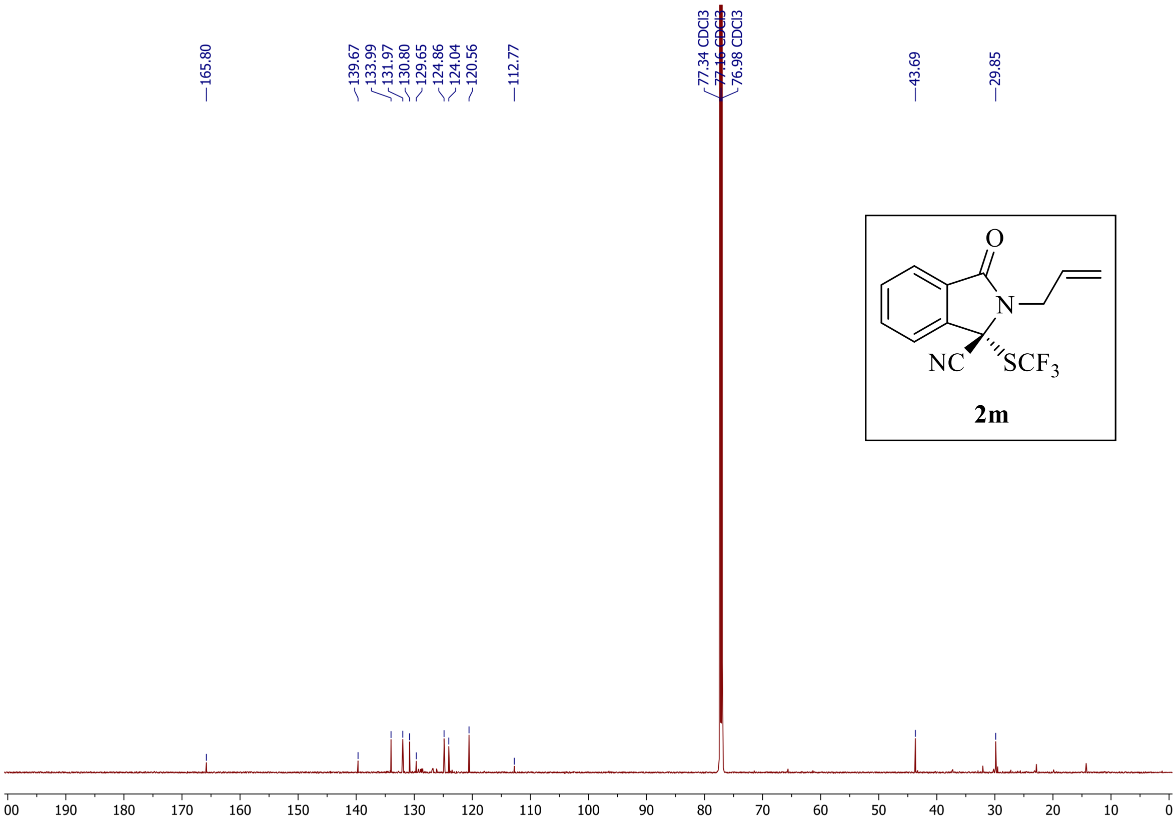

^{13}C-NMR (75 MHz, $CDCl_3$)

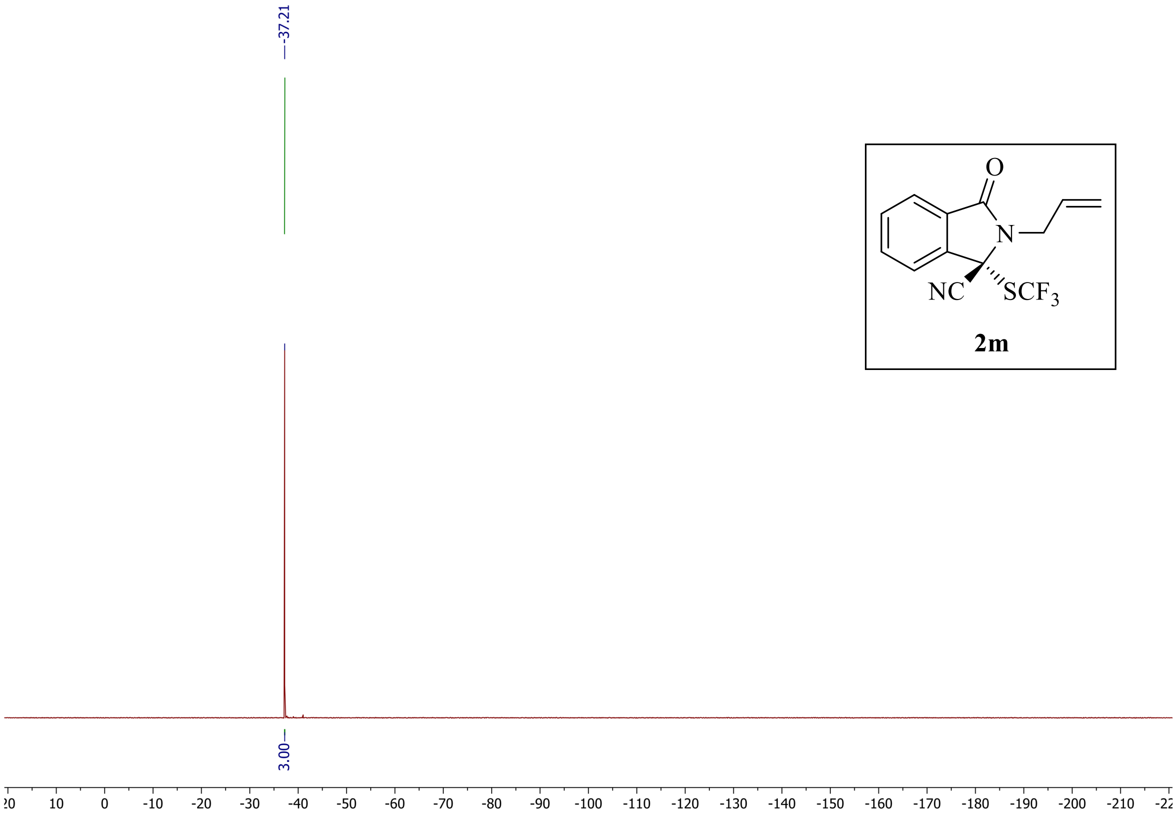

^{19}F-NMR (471 MHz, $CDCl_3$)

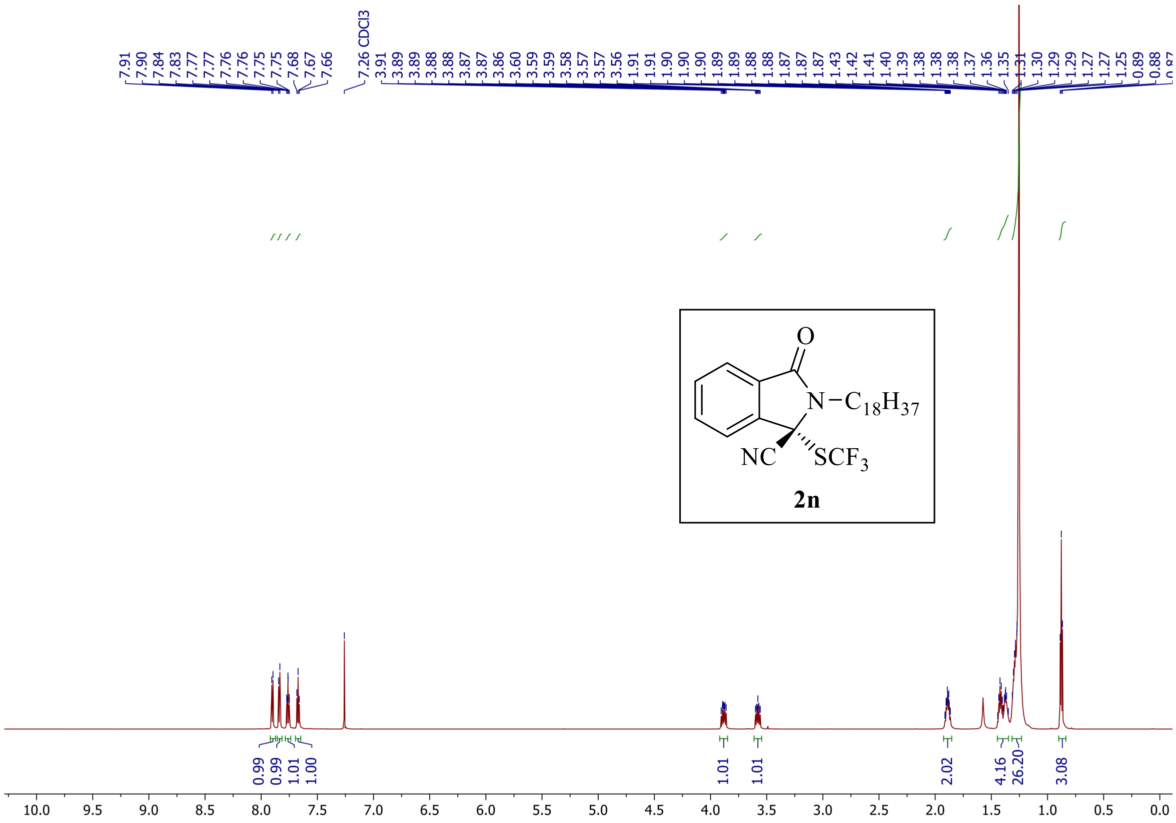

^{1}H-NMR (700 MHz, $CDCl_3$)

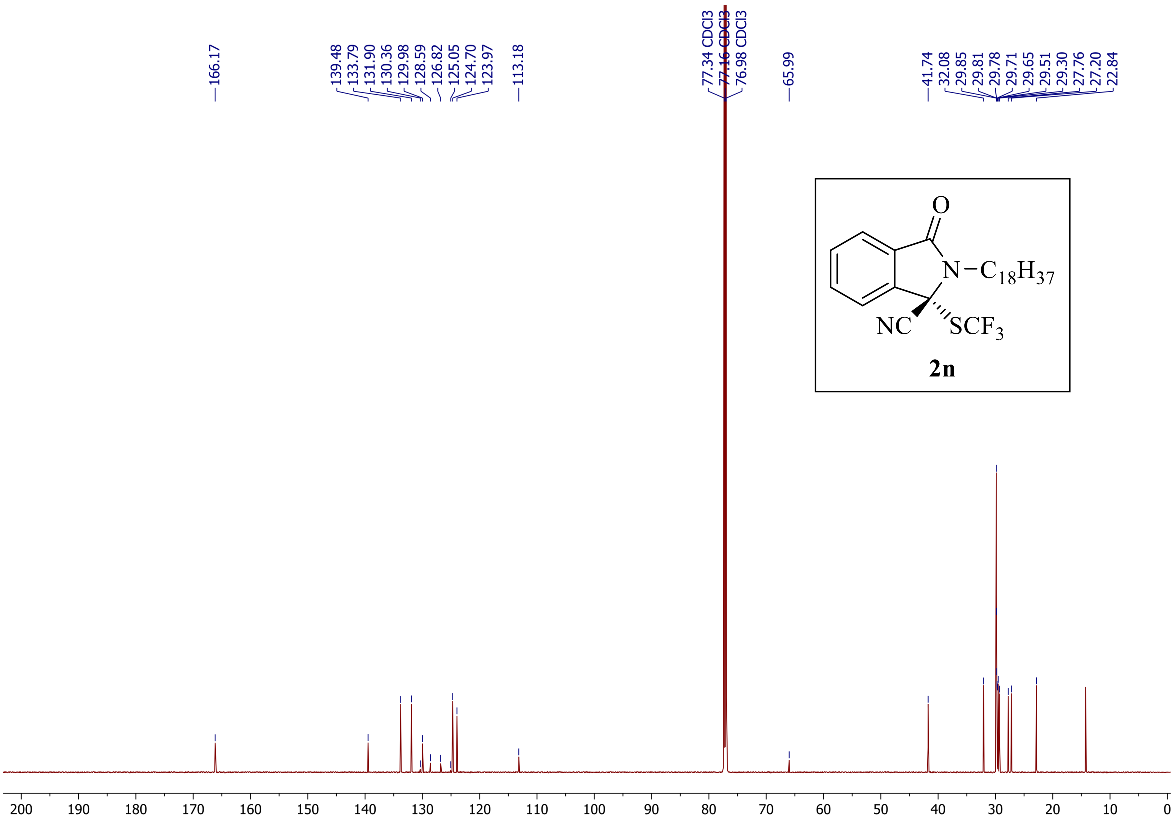

^{13}C-NMR (176 MHz, $CDCl_3$)

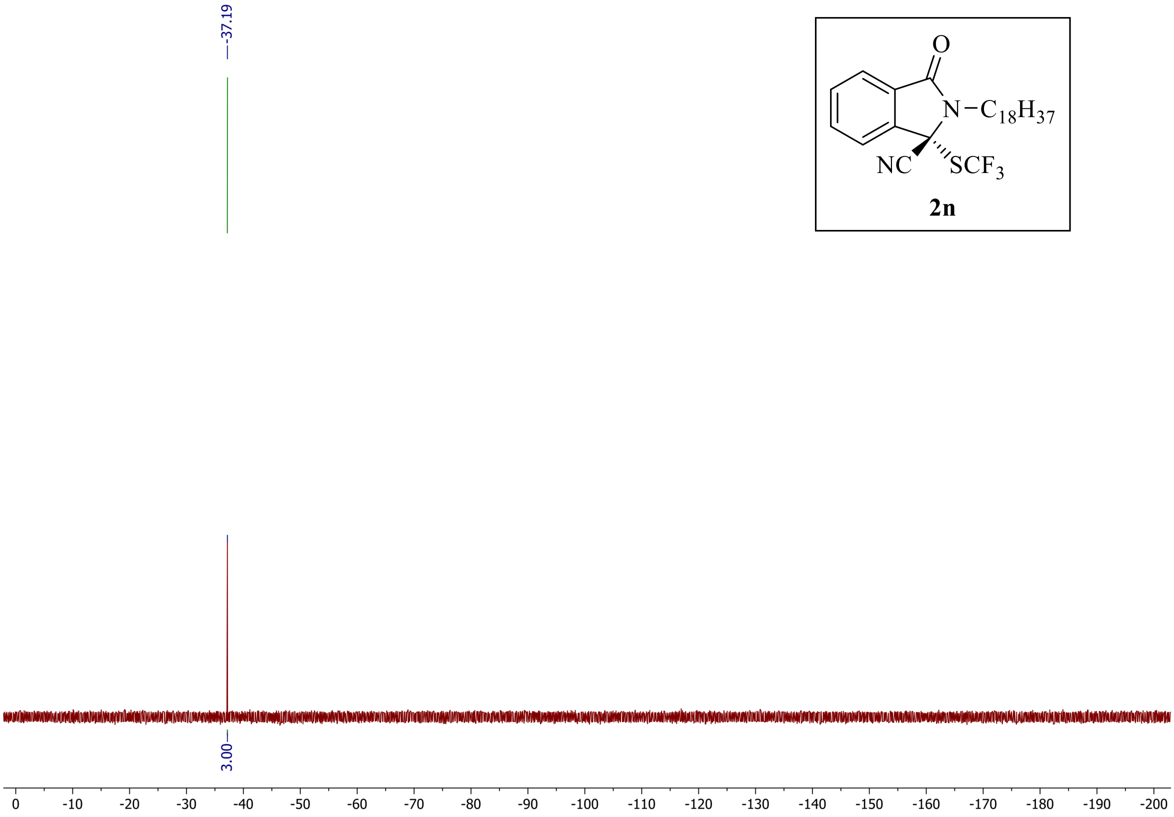

^{19}F-NMR (471 MHz, $CDCl_3$)

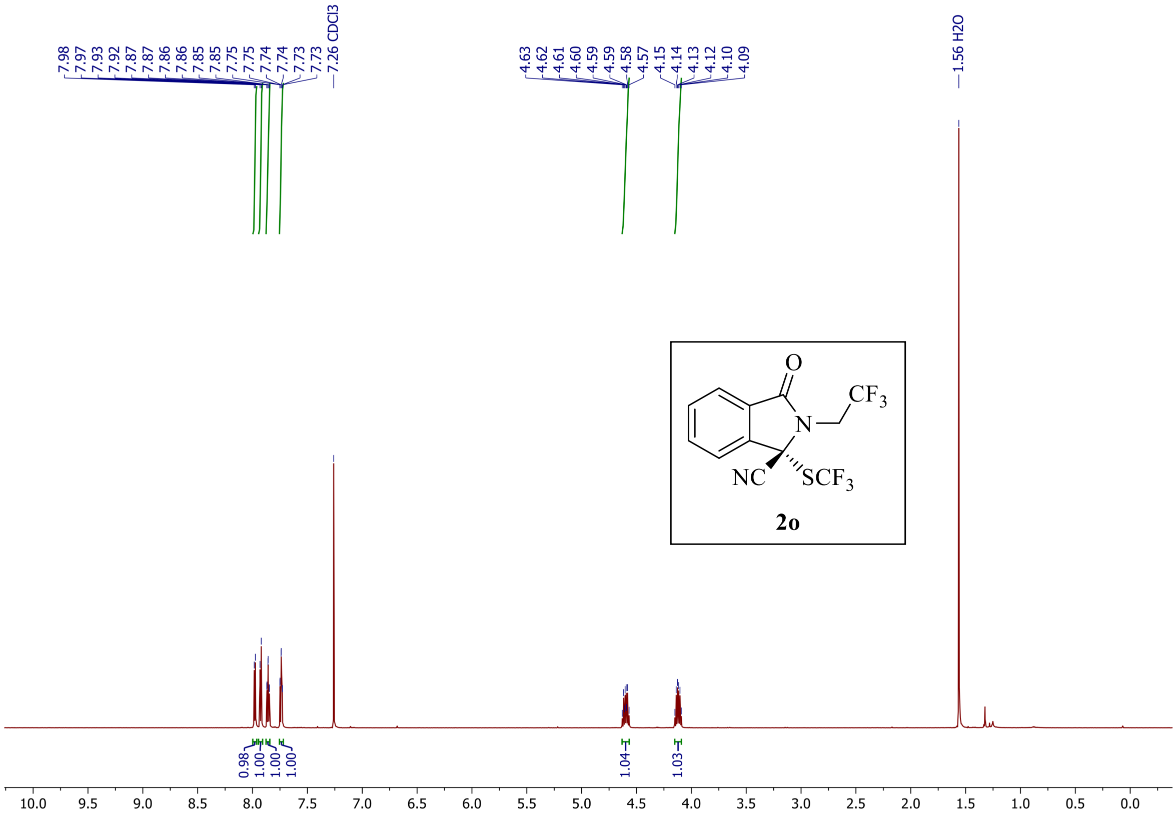

^{1}H-NMR (700 MHz, $CDCl_3$)

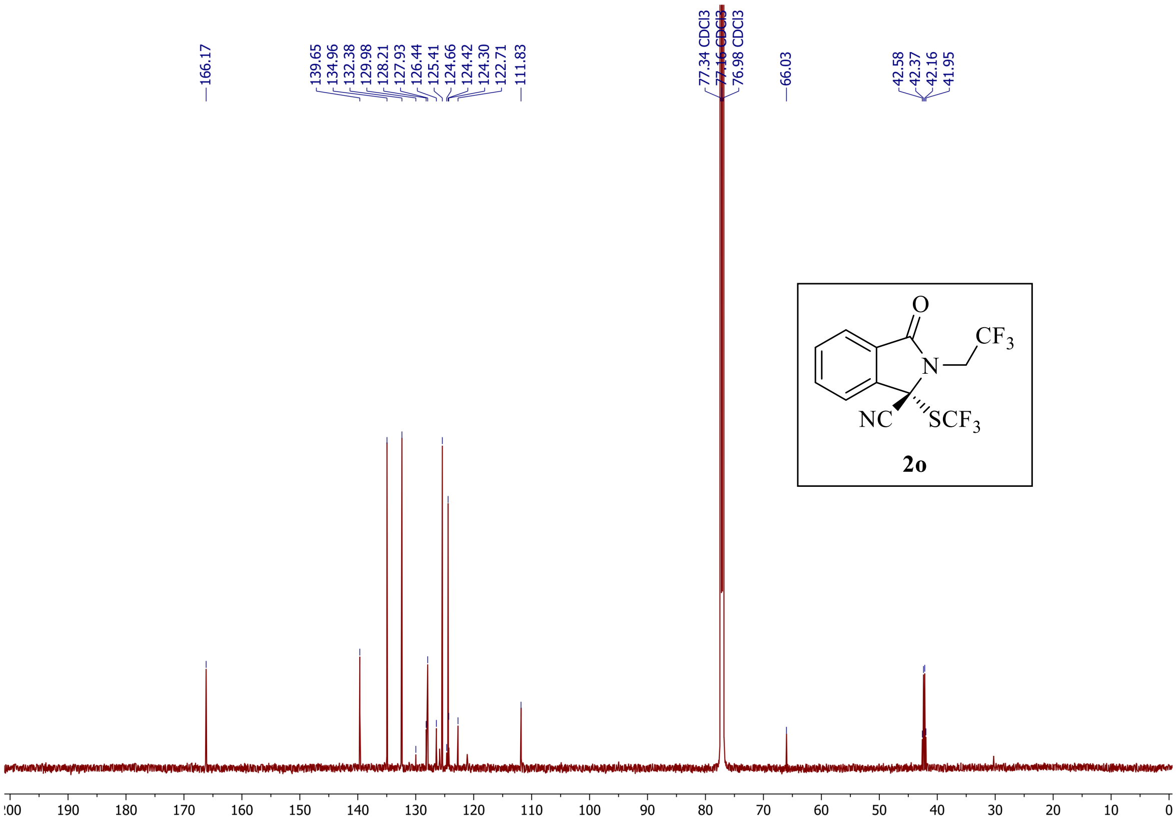

^{13}C-NMR (176 MHz, $CDCl_3$)

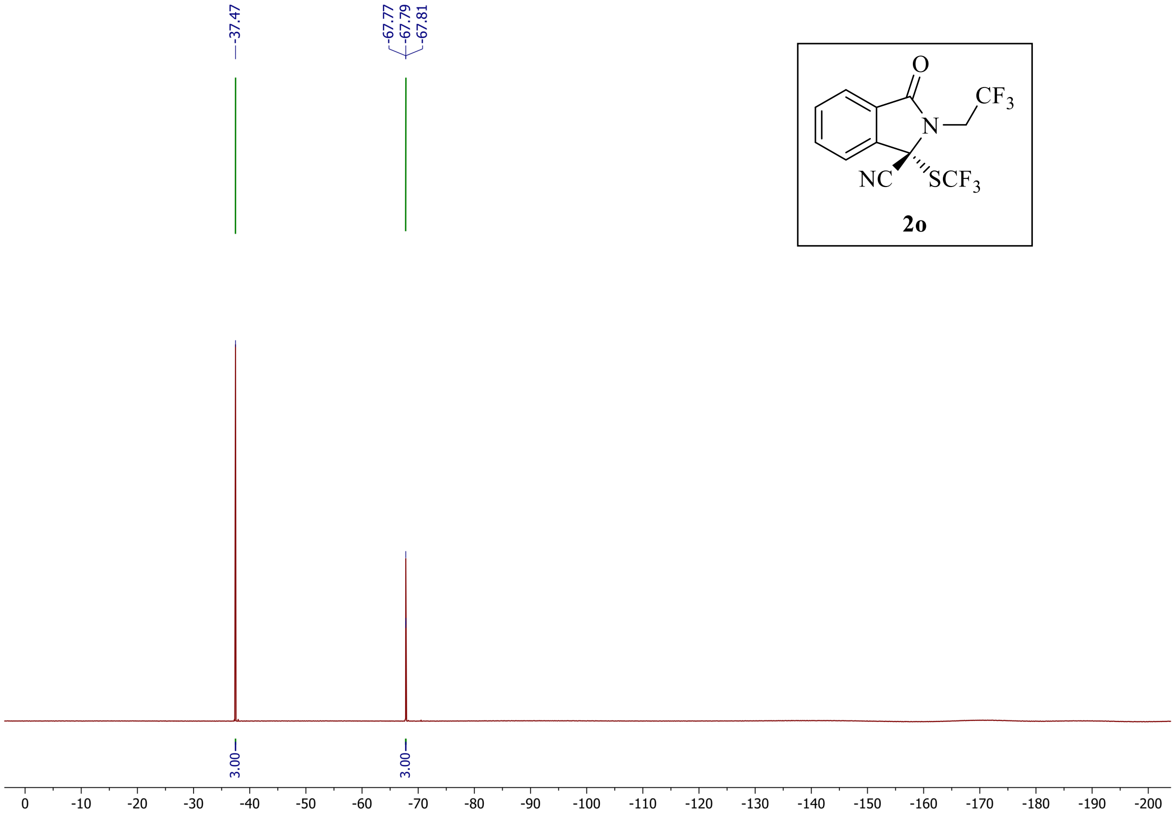

^{19}F-NMR (471 MHz, $CDCl_3$)

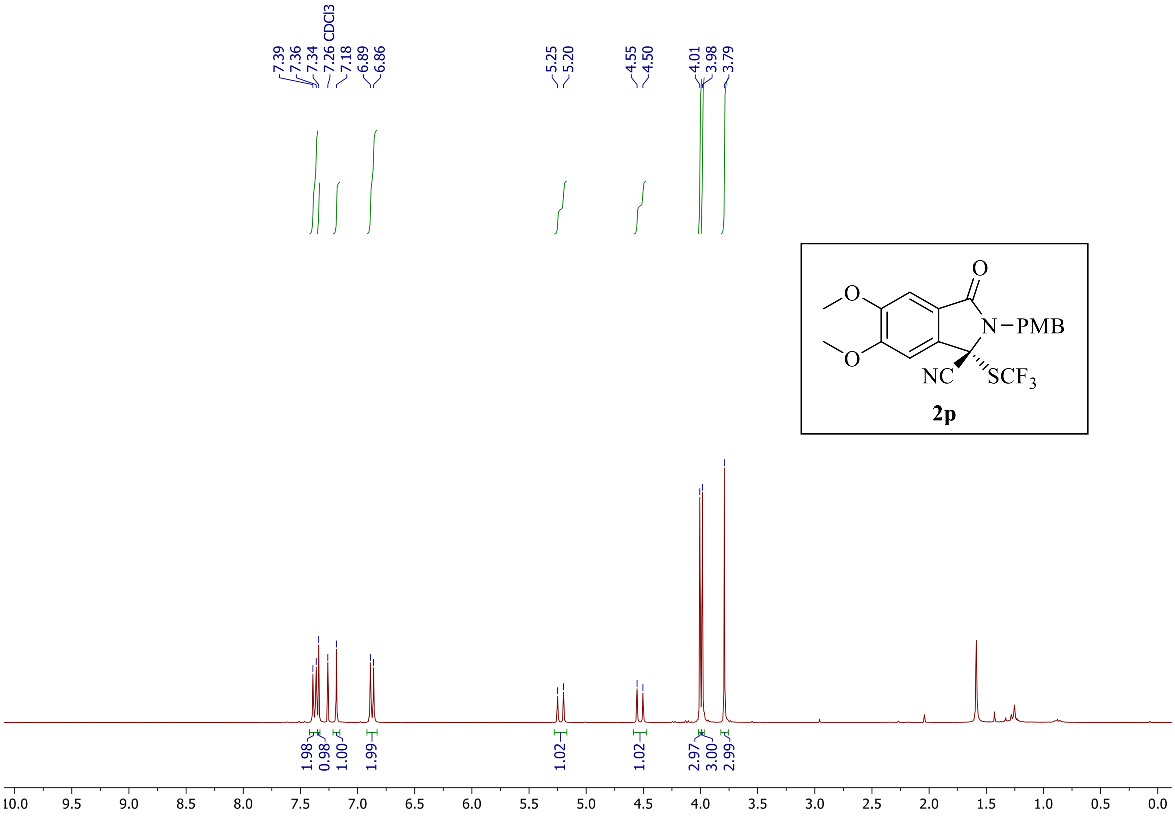

^{1}H-NMR (700 MHz, $CDCl_3$)

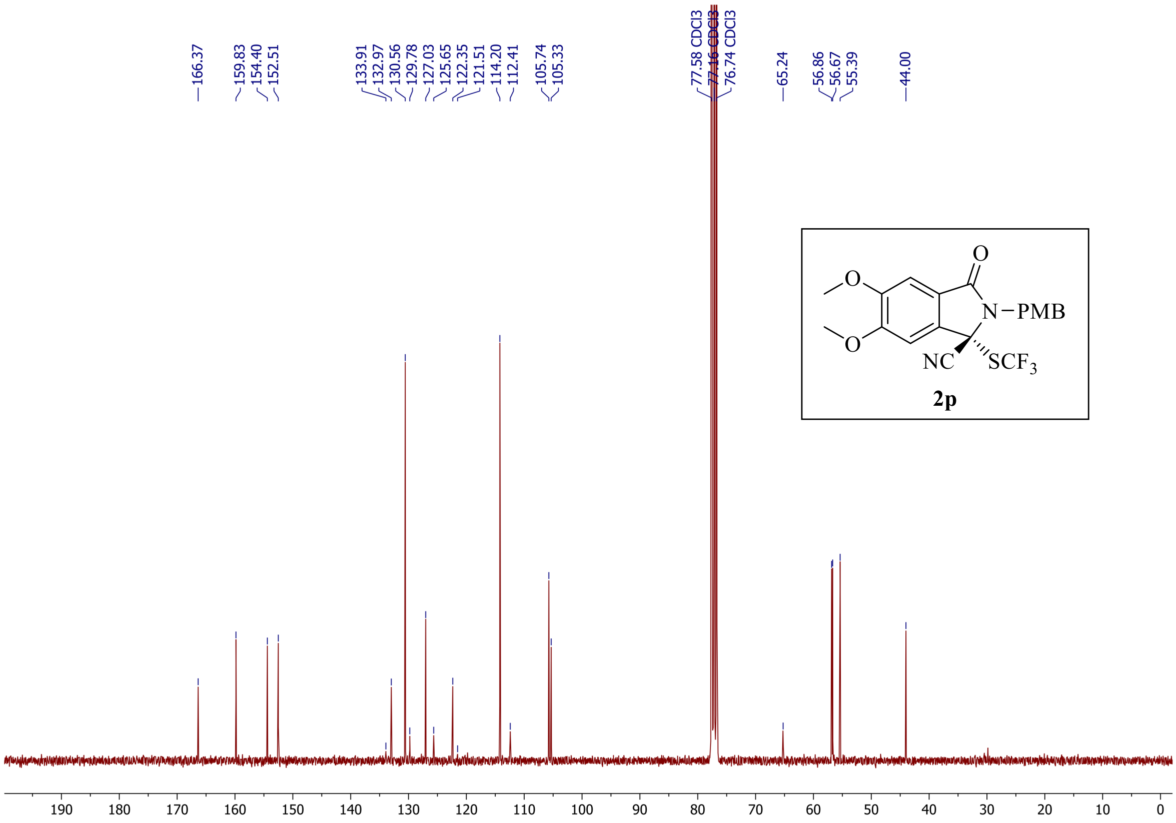

^{13}C-NMR (176 MHz, $CDCl_3$)

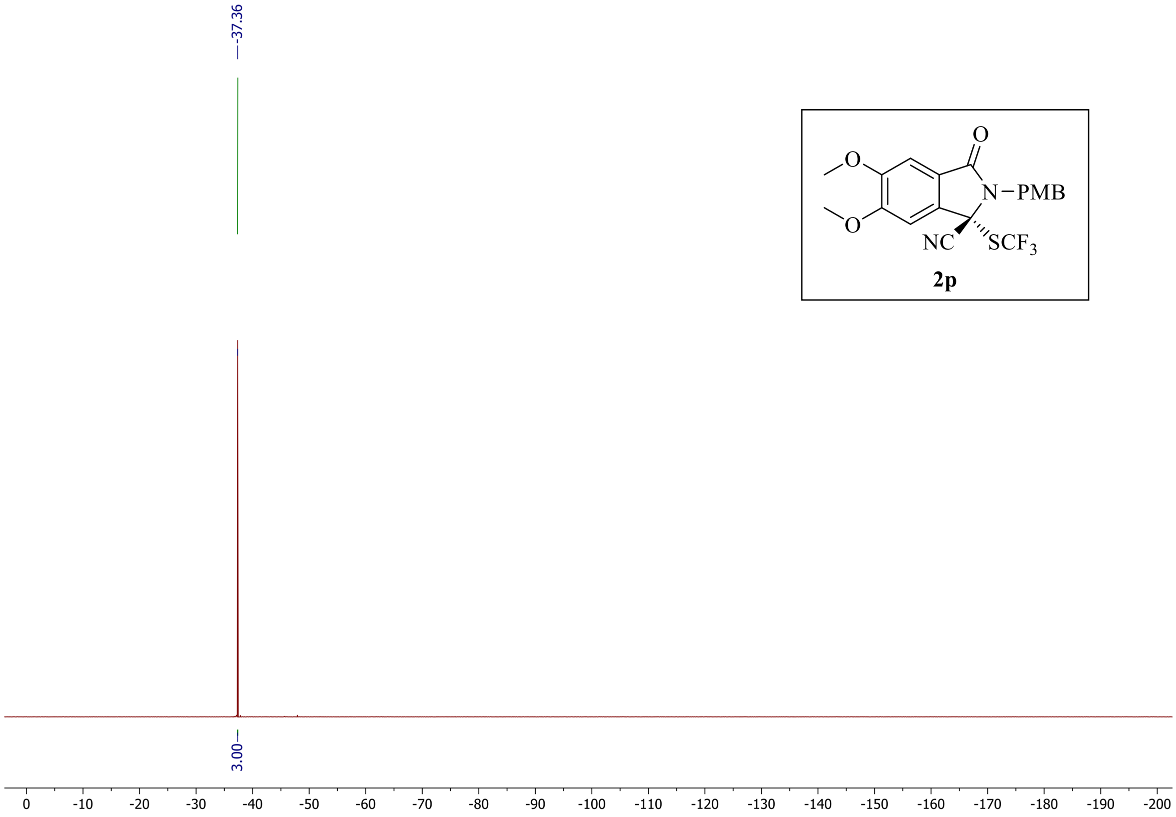

^{19}F-NMR (471 MHz, $CDCl_3$)

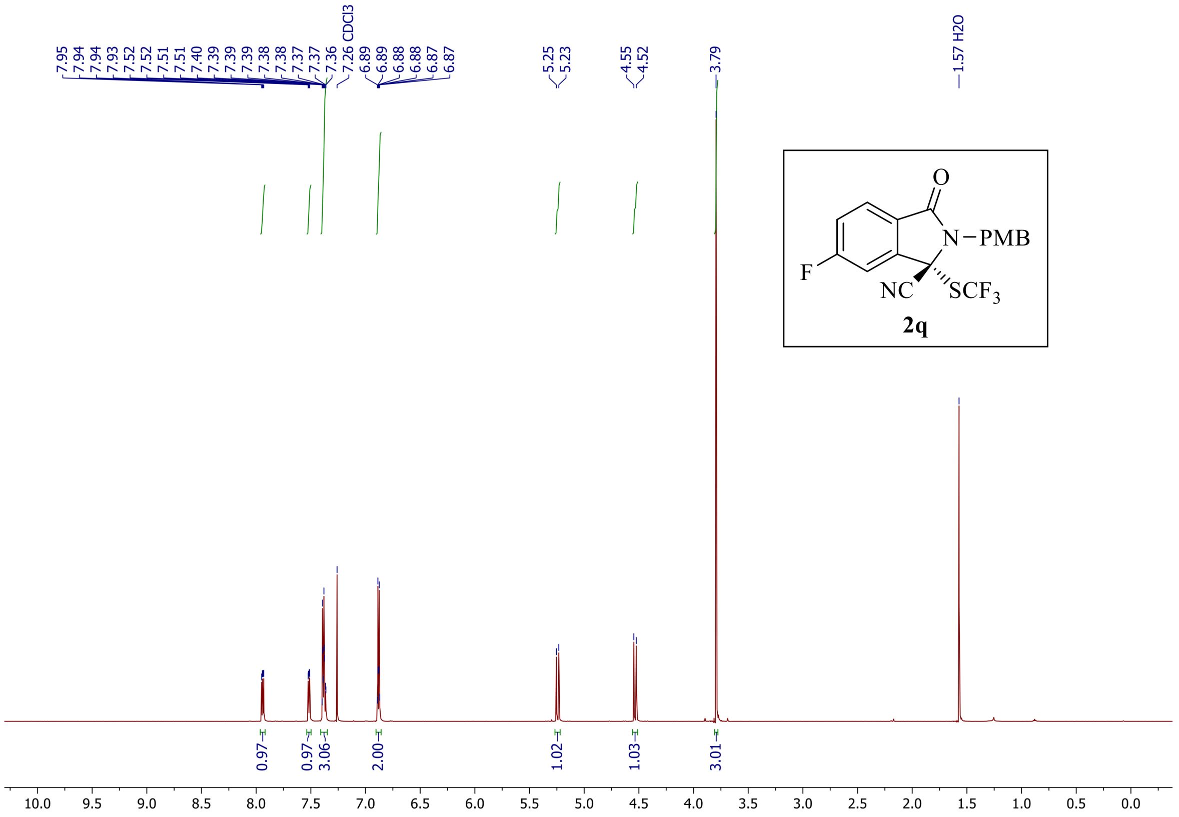

^{1}H-NMR (700 MHz, $CDCl_3$)

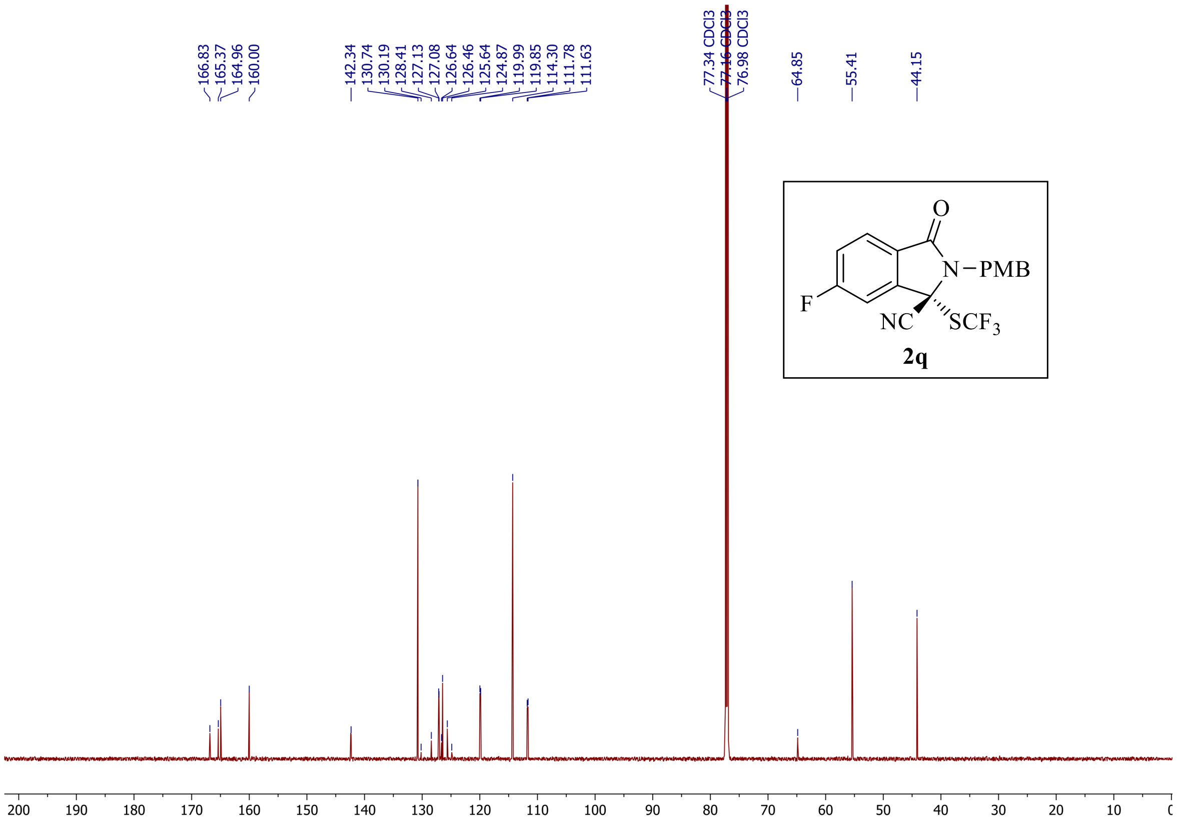

^{13}C-NMR (176 MHz, $CDCl_3$)

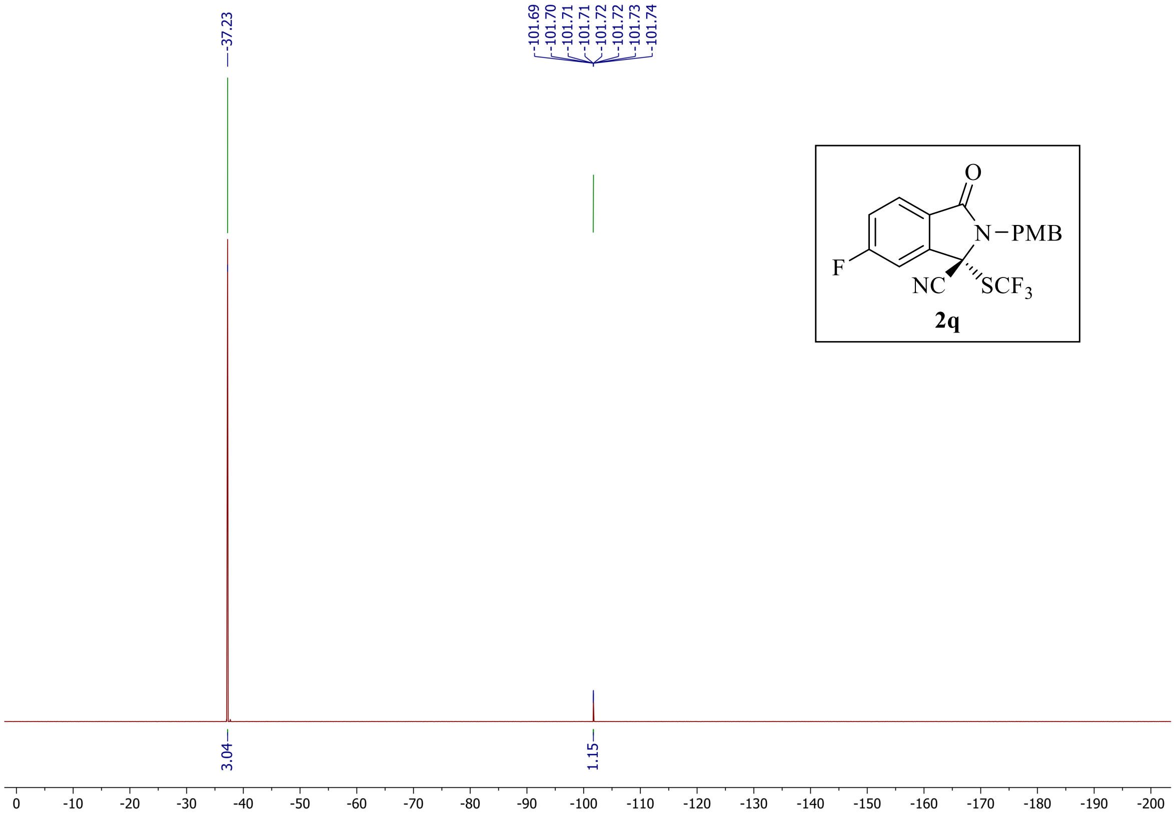

^{19}F-NMR (471 MHz, $CDCl_3$)

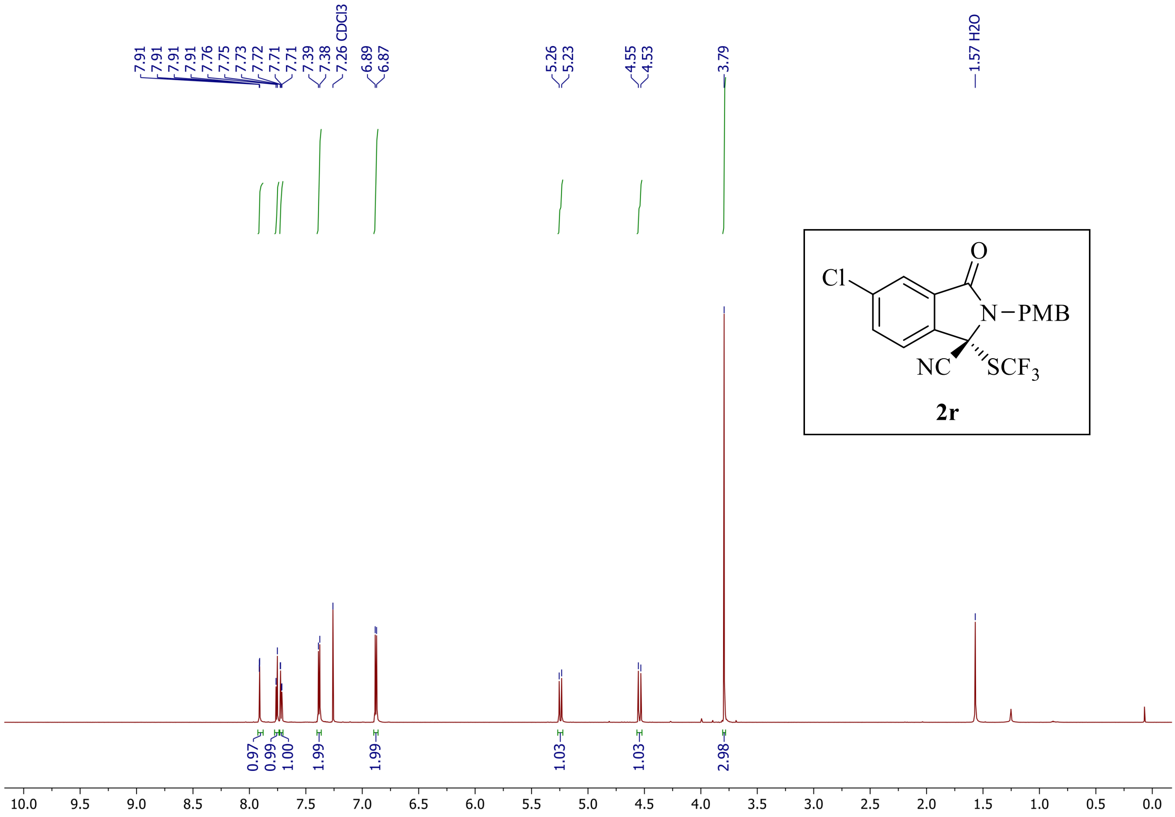

^{1}H-NMR (700 MHz, $CDCl_3$)

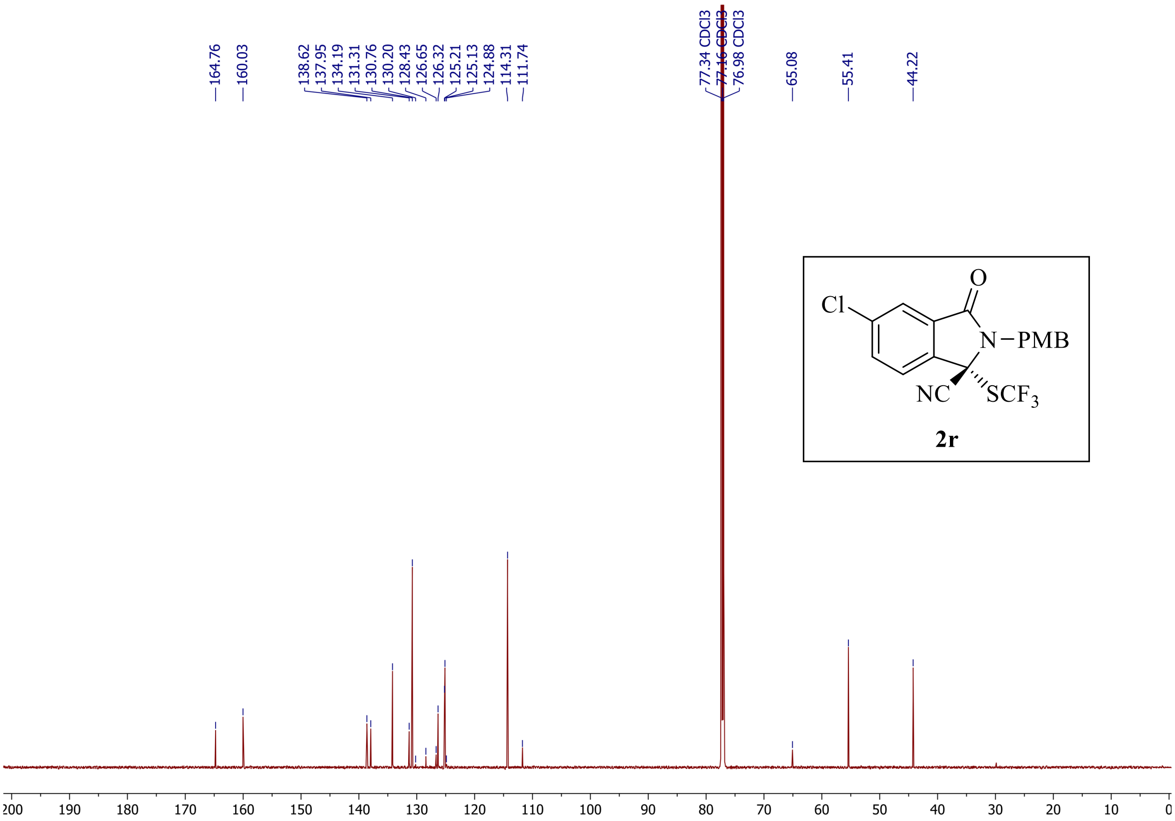

^{13}C-NMR (176 MHz, $CDCl_3$)

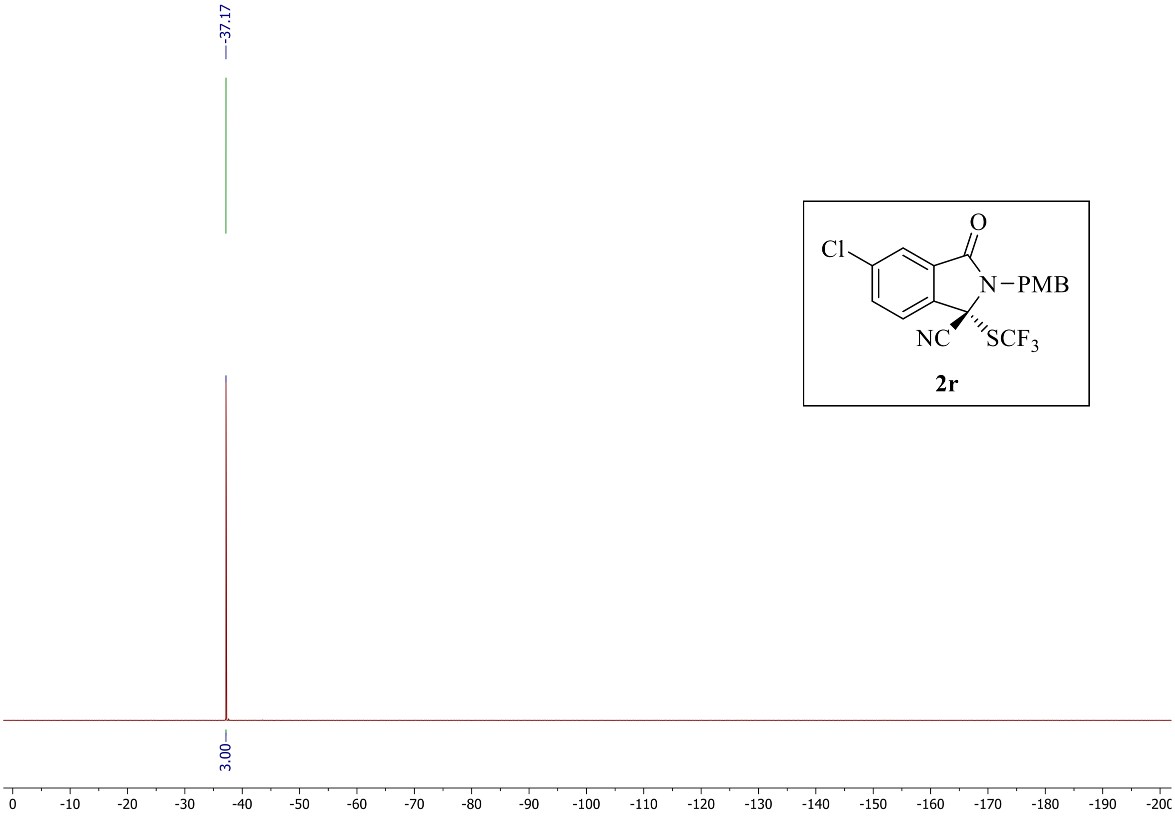

^{19}F-NMR (471 MHz, $CDCl_3$)

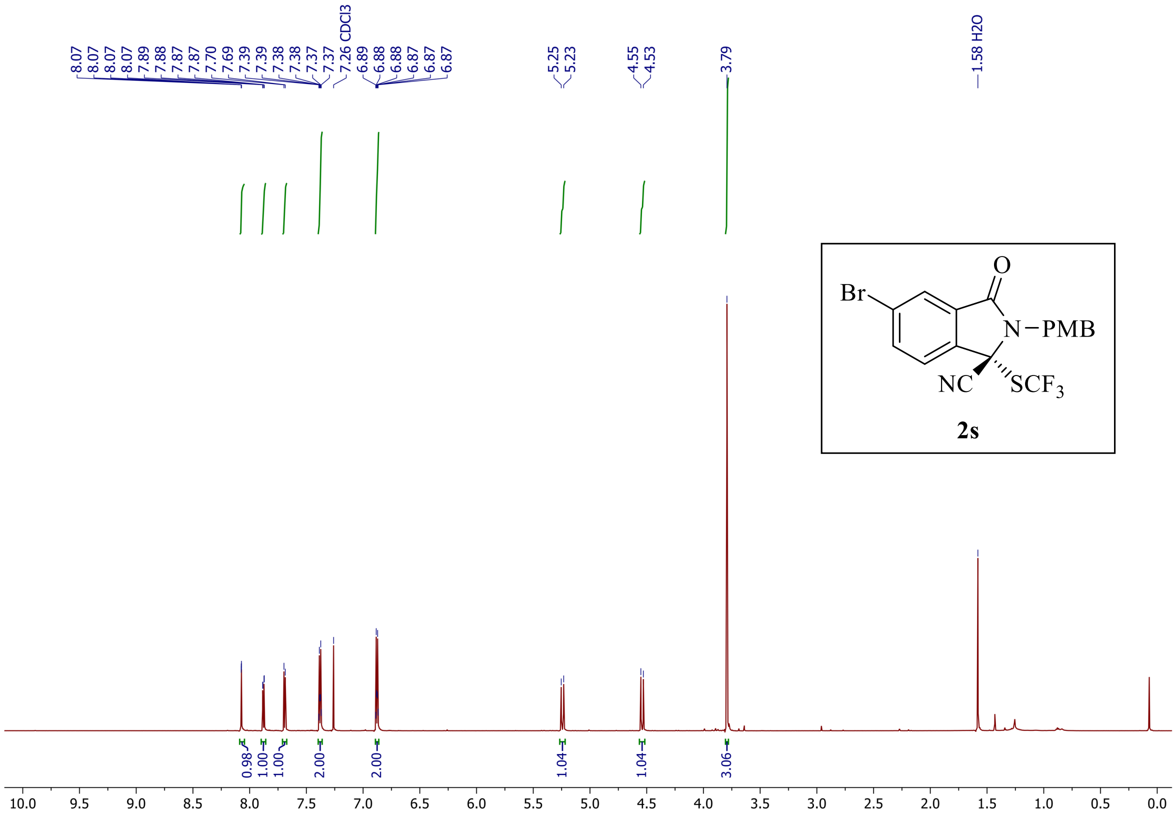

^{1}H-NMR (700 MHz, $CDCl_3$)

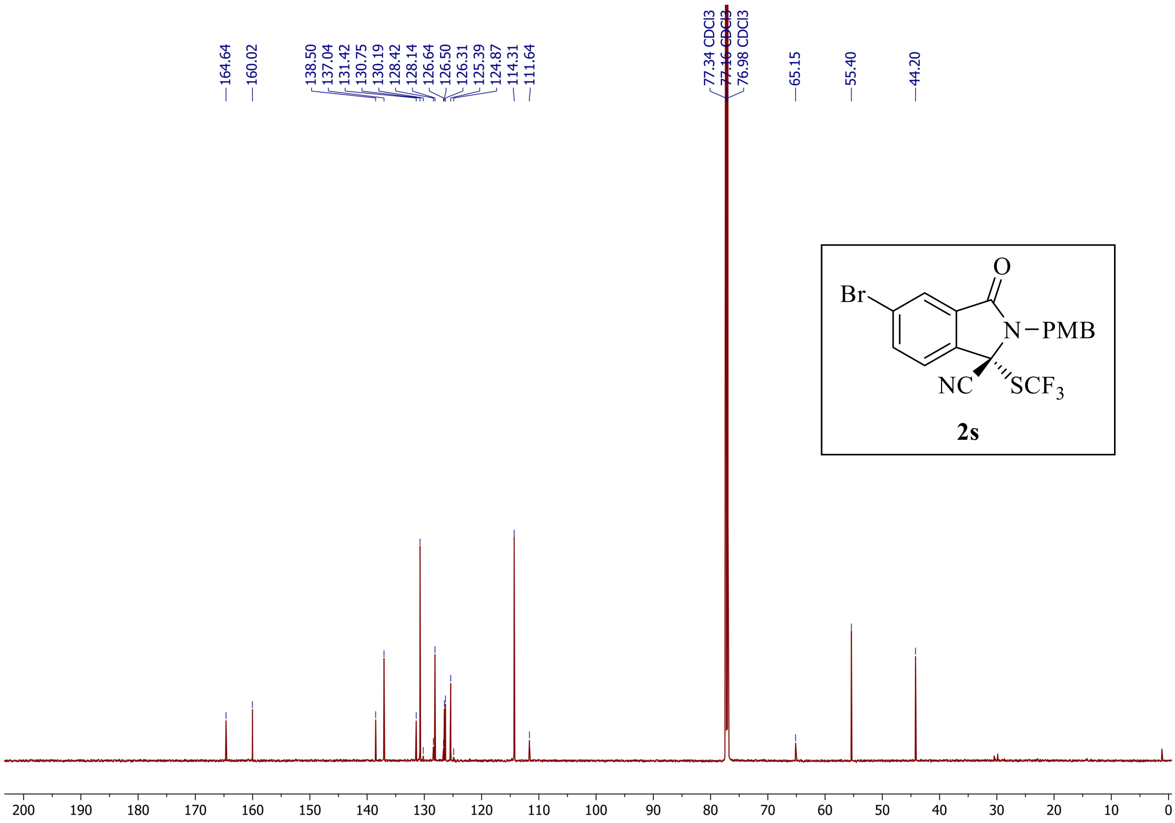

^{13}C-NMR (176 MHz, $CDCl_3$)

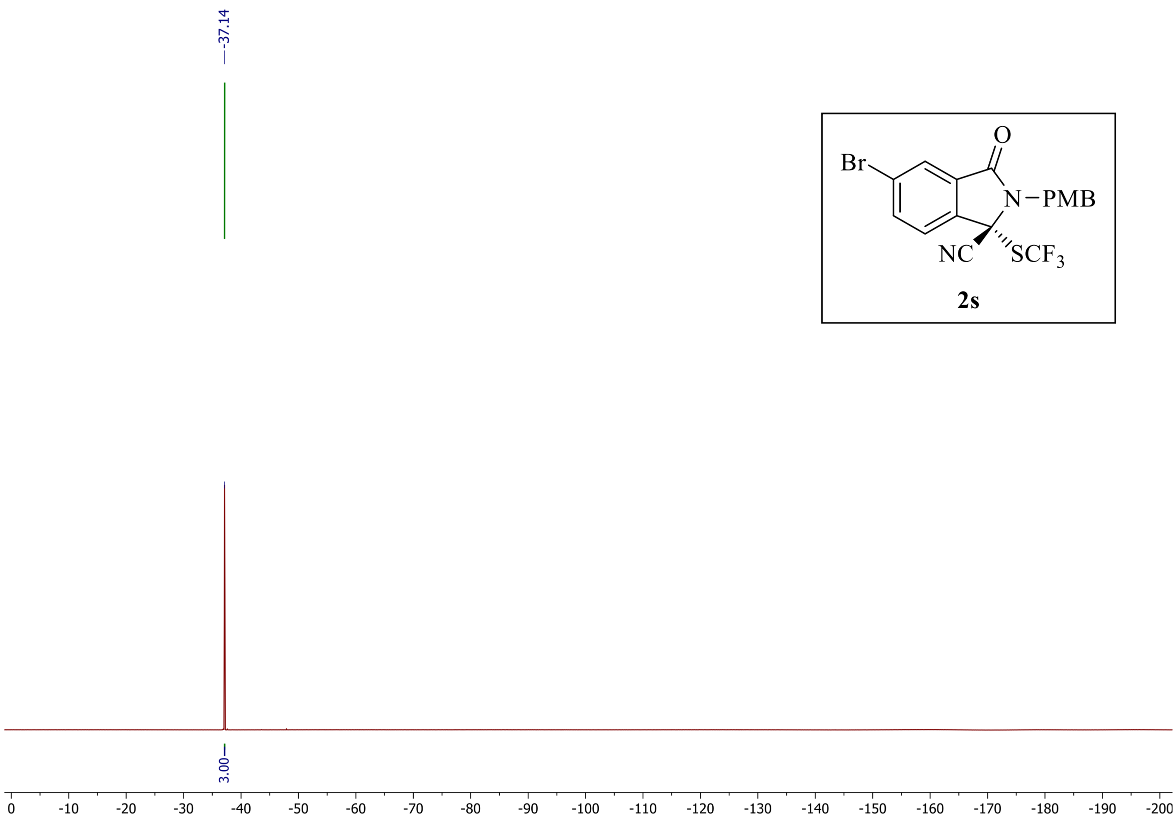

^{19}F-NMR (471 MHz, $CDCl_3$)

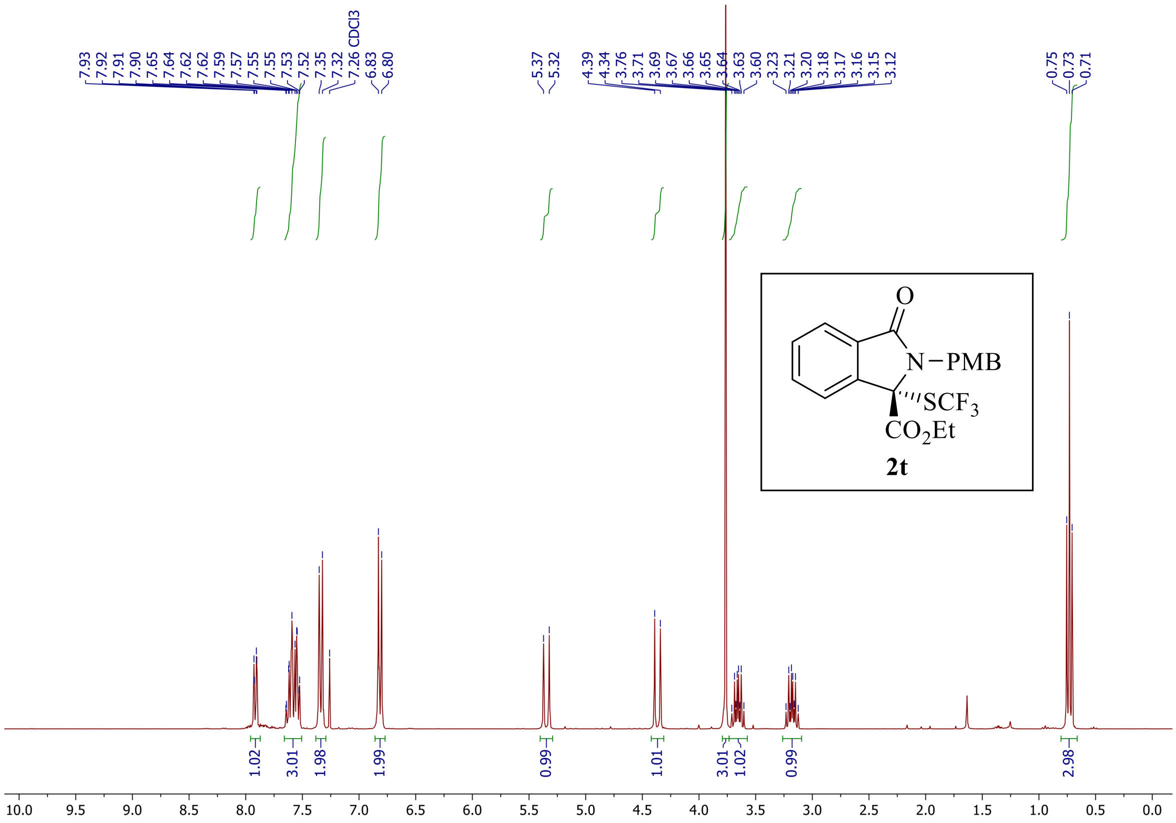

^{1}H-NMR (300 MHz, $CDCl_3$)

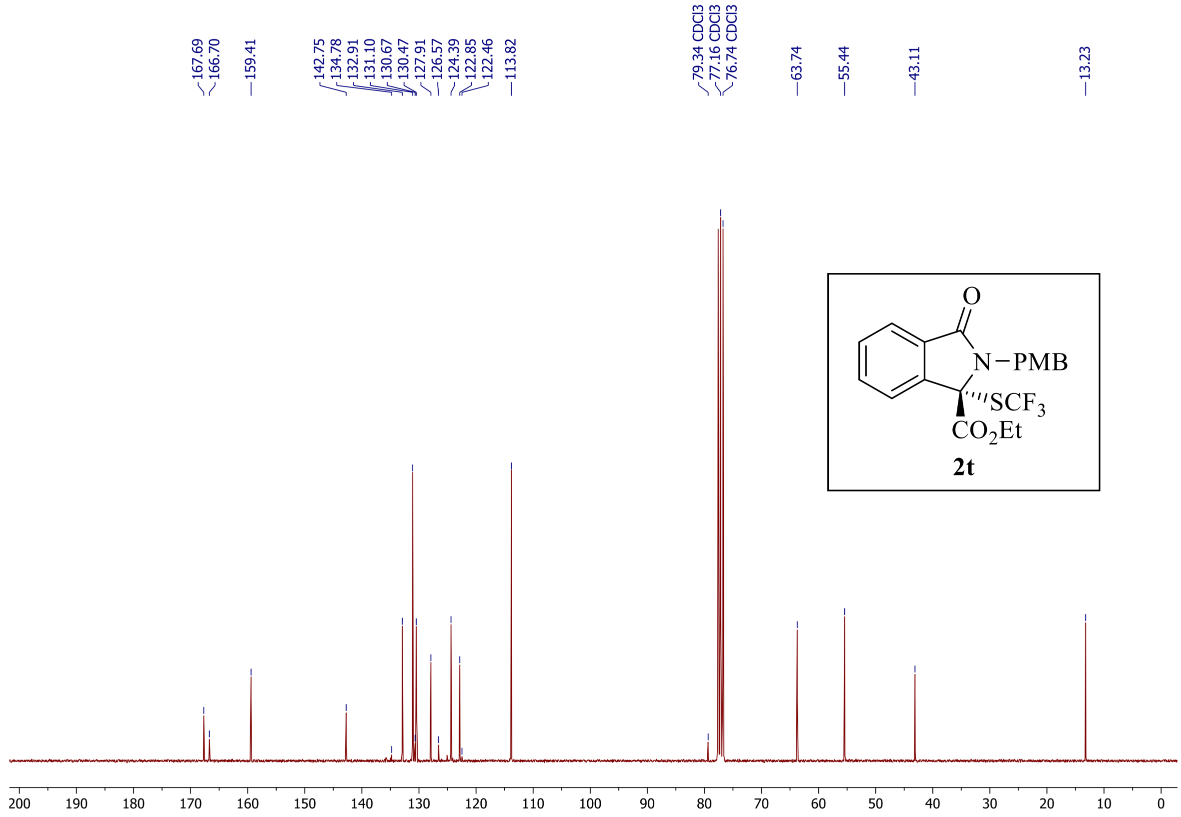

^{13}C-NMR (75 MHz, $CDCl_3$)

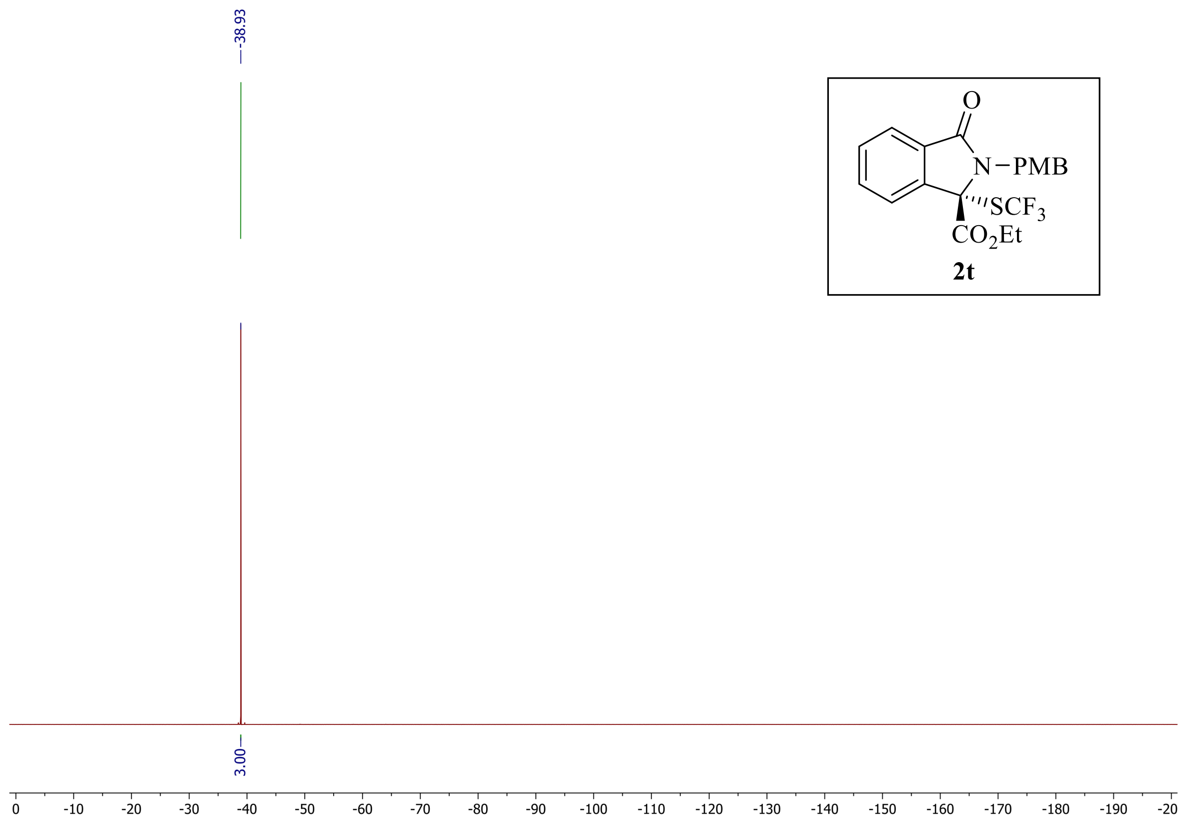

^{19}F-NMR (471 MHz, $CDCl_3$)

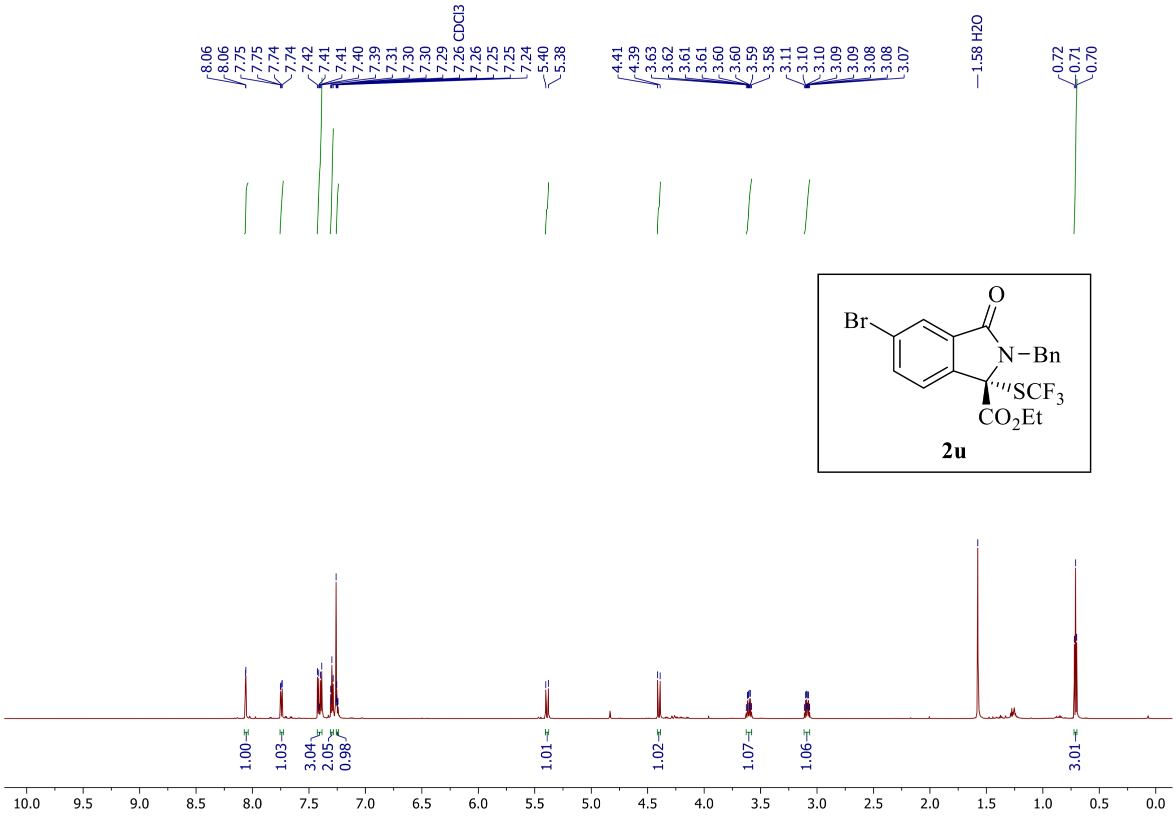

^{1}H-NMR (700 MHz, $CDCl_3$)

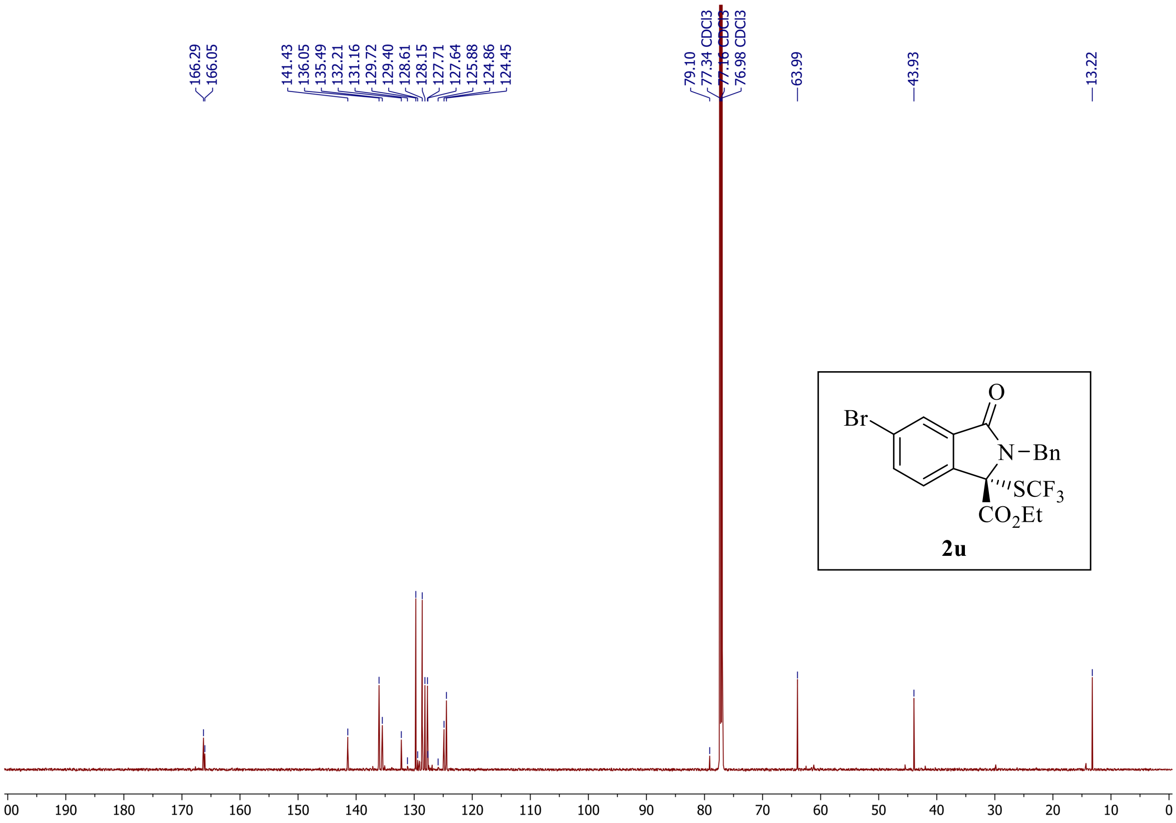

^{13}C-NMR (176 MHz, $CDCl_3$)

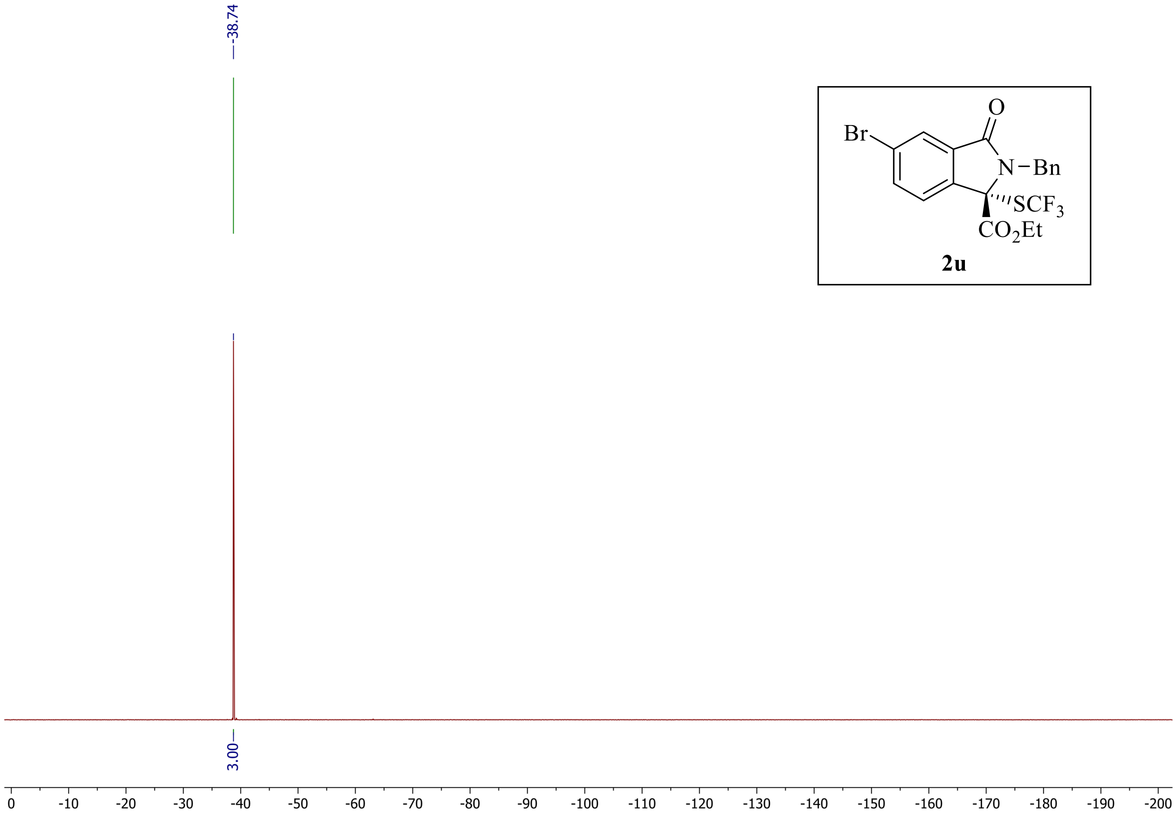

^{19}F-NMR (471 MHz, $CDCl_3$)

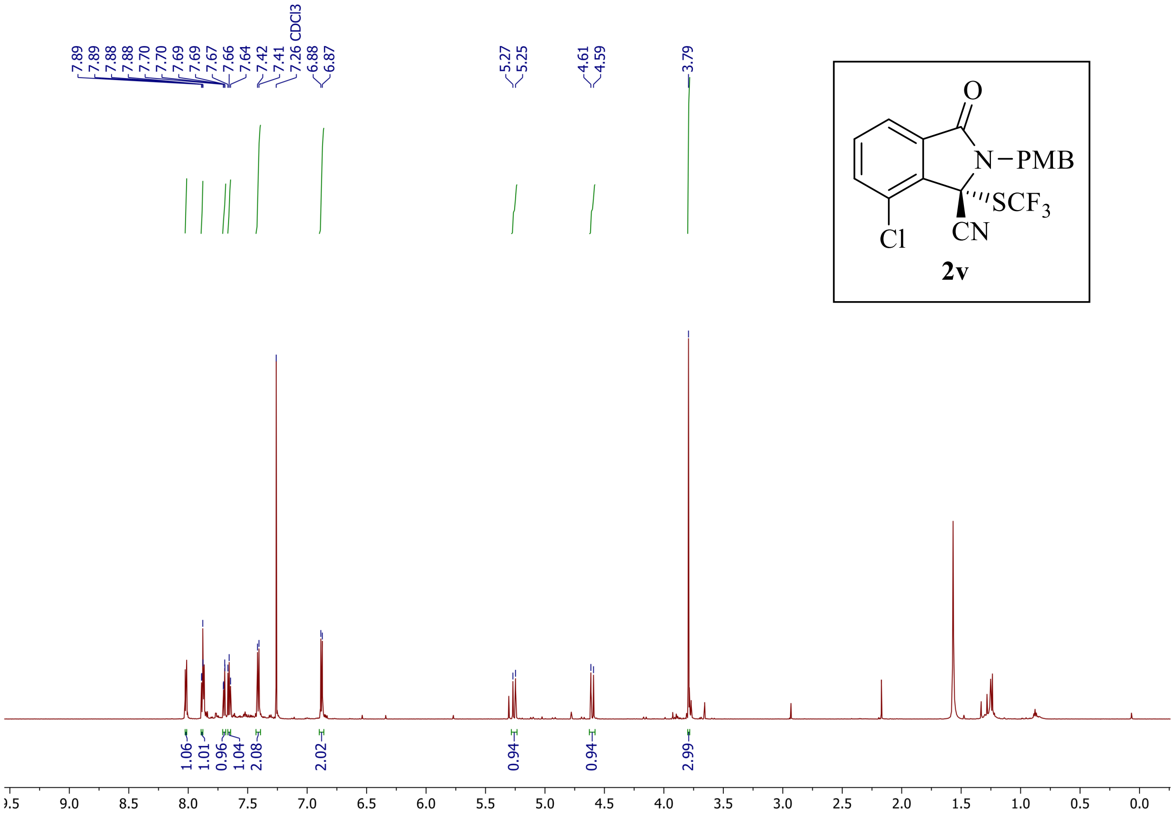

^{1}H-NMR (700 MHz, $CDCl_3$)

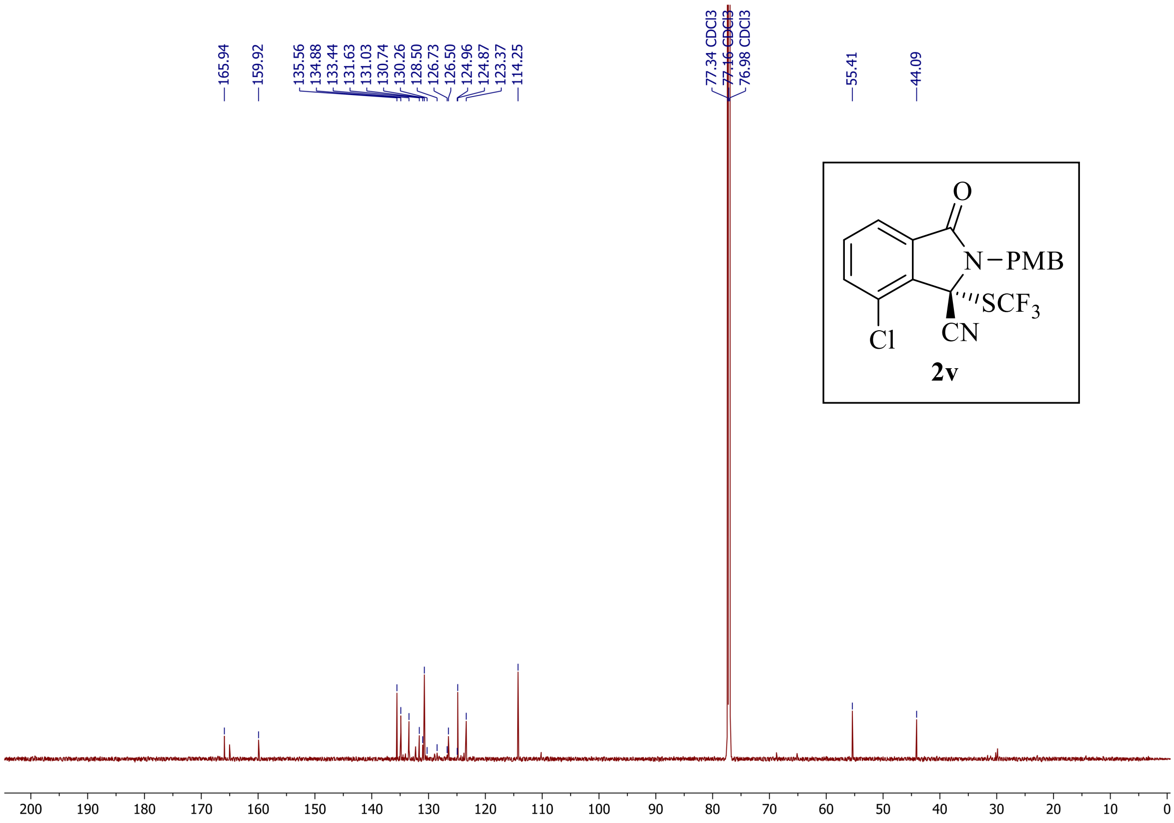

^{13}C-NMR (176 MHz, $CDCl_3$)

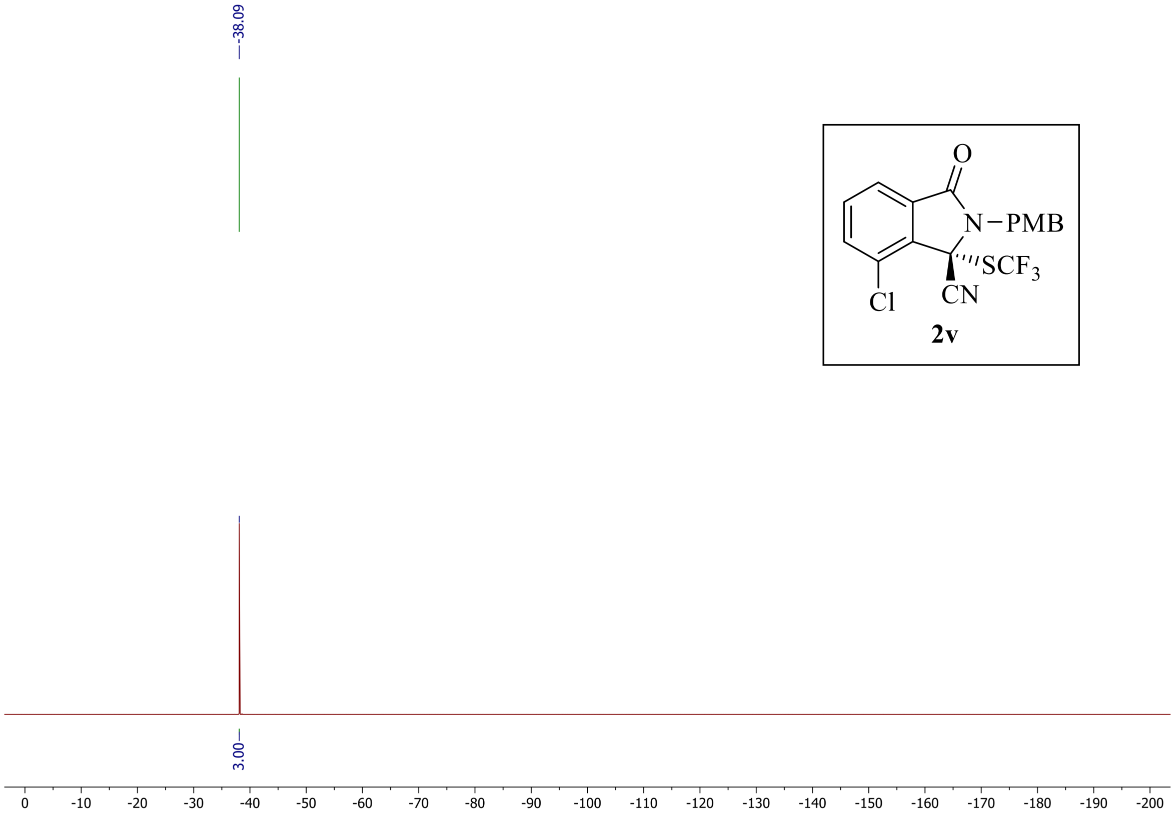

^{19}F-NMR (471 MHz, $CDCl_3$)

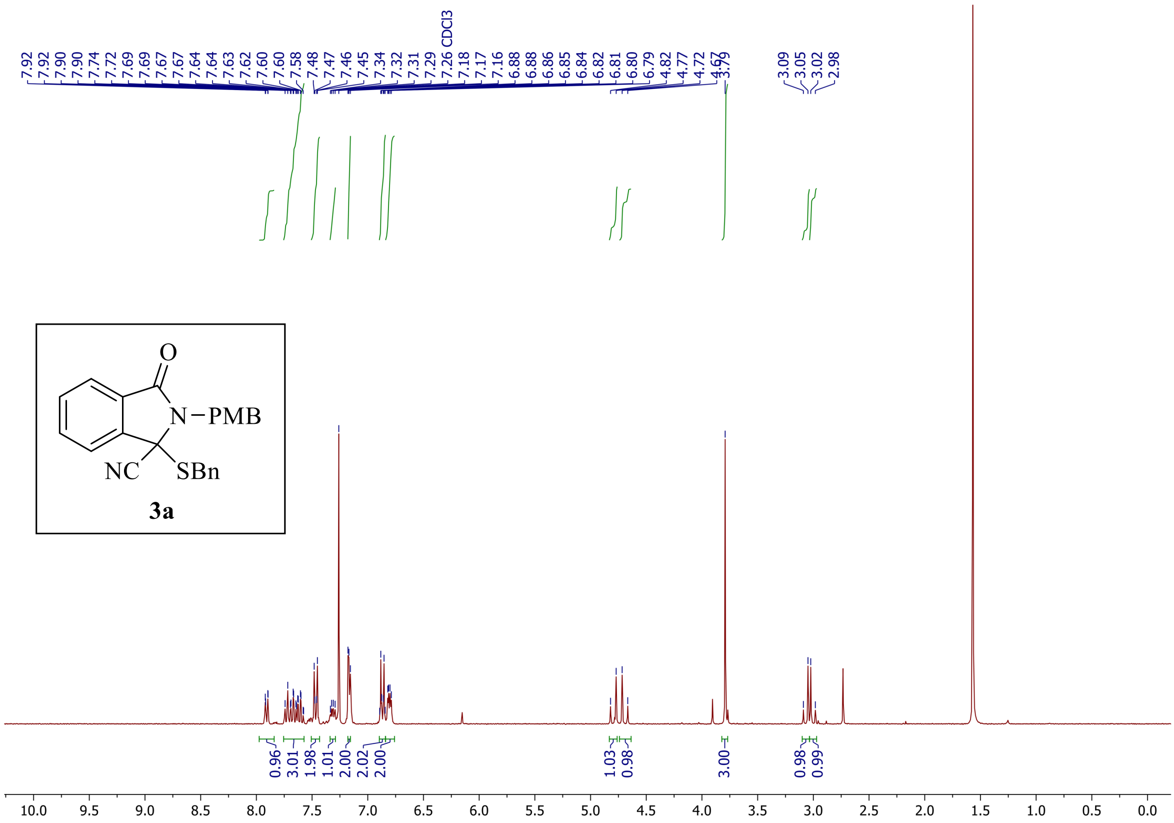

^{1}H-NMR (300 MHz, $CDCl_3$)

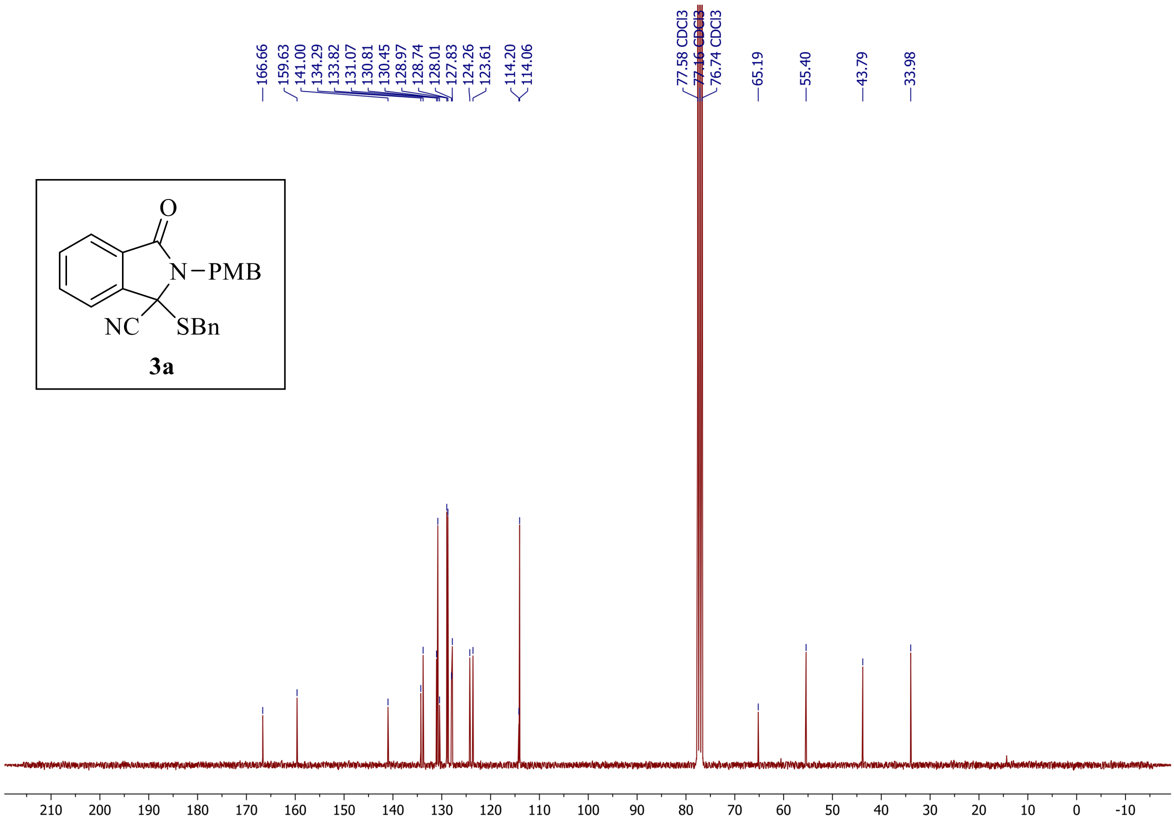

^{13}C-NMR (75 MHz, $CDCl_3$)

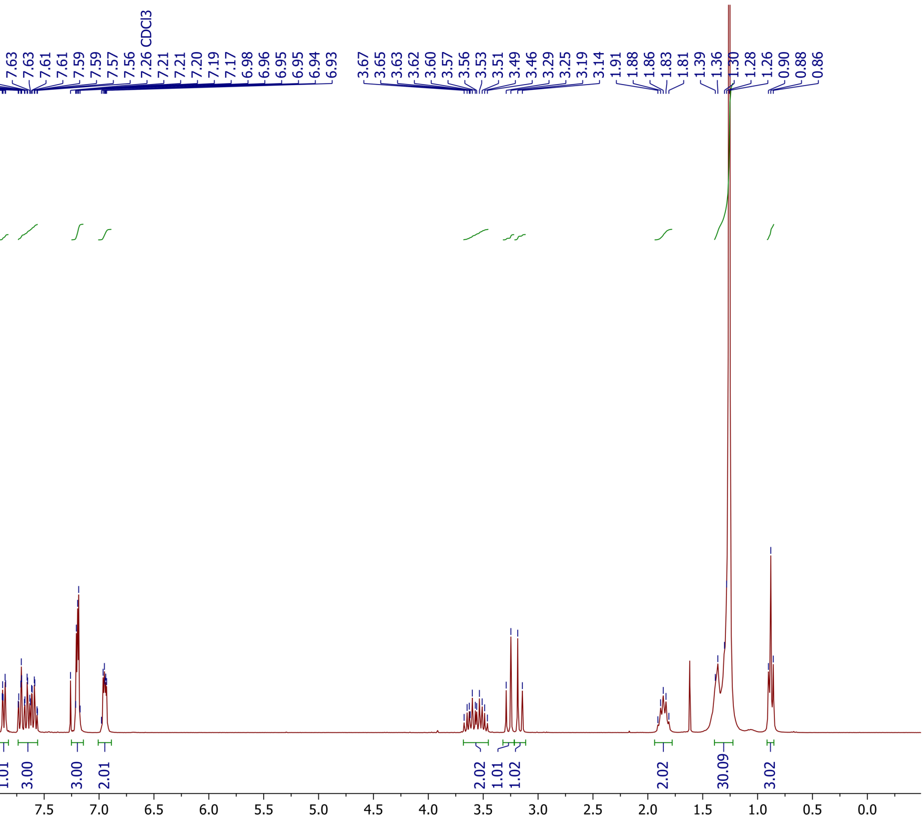

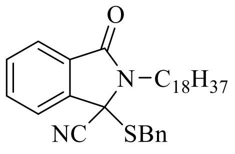

3b

^{1}H-NMR (300 MHz, $CDCl_3$)

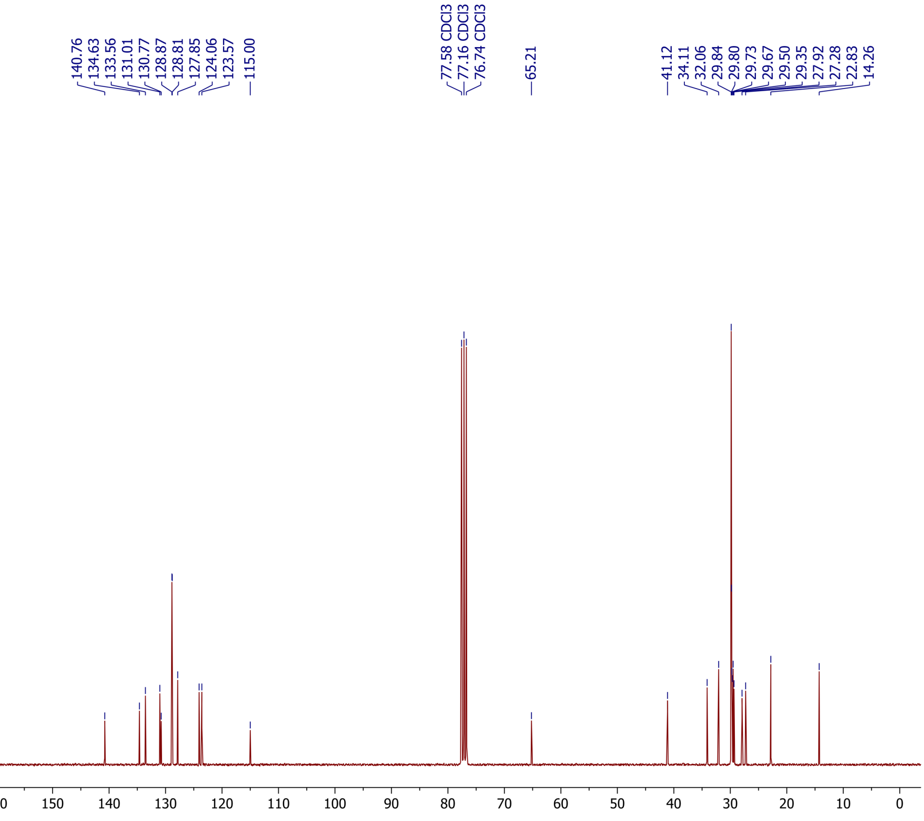

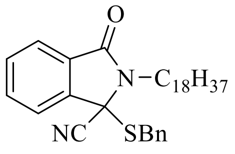

3b

^{13}C-NMR (75 MHz, $CDCl_3$)

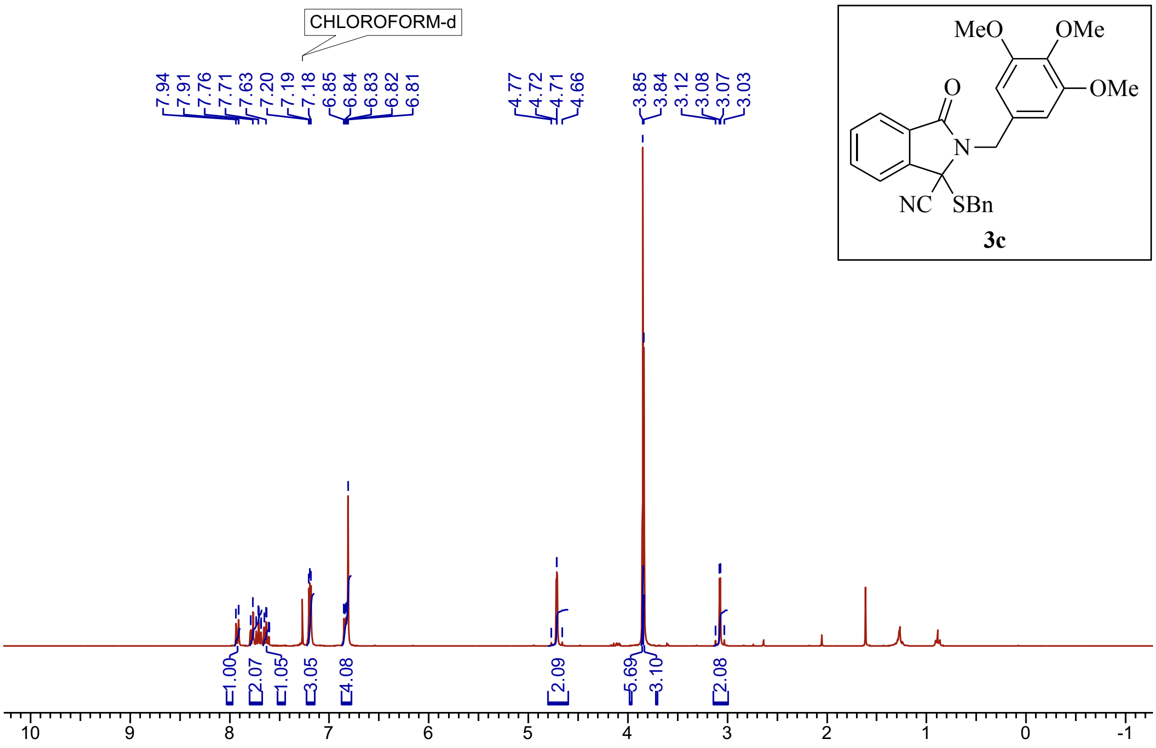

^{1}H-NMR (300 MHz, $CDCl_3$)

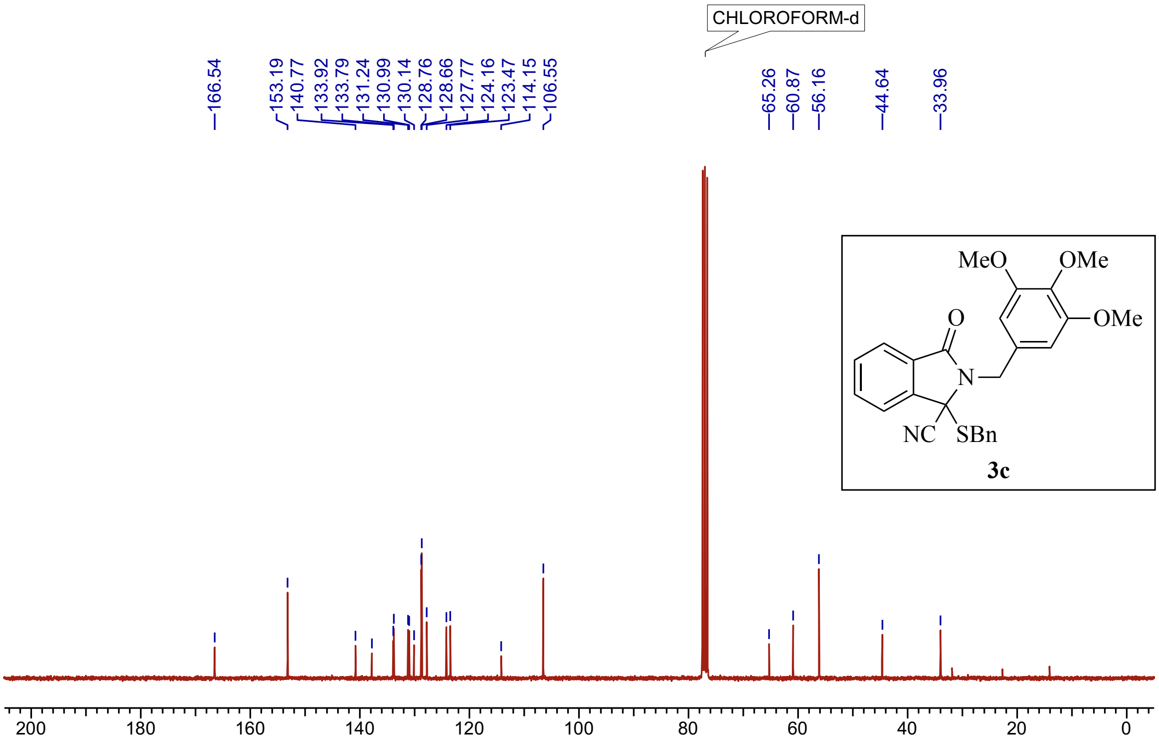

^{13}C-NMR (75 MHz, $CDCl_3$)

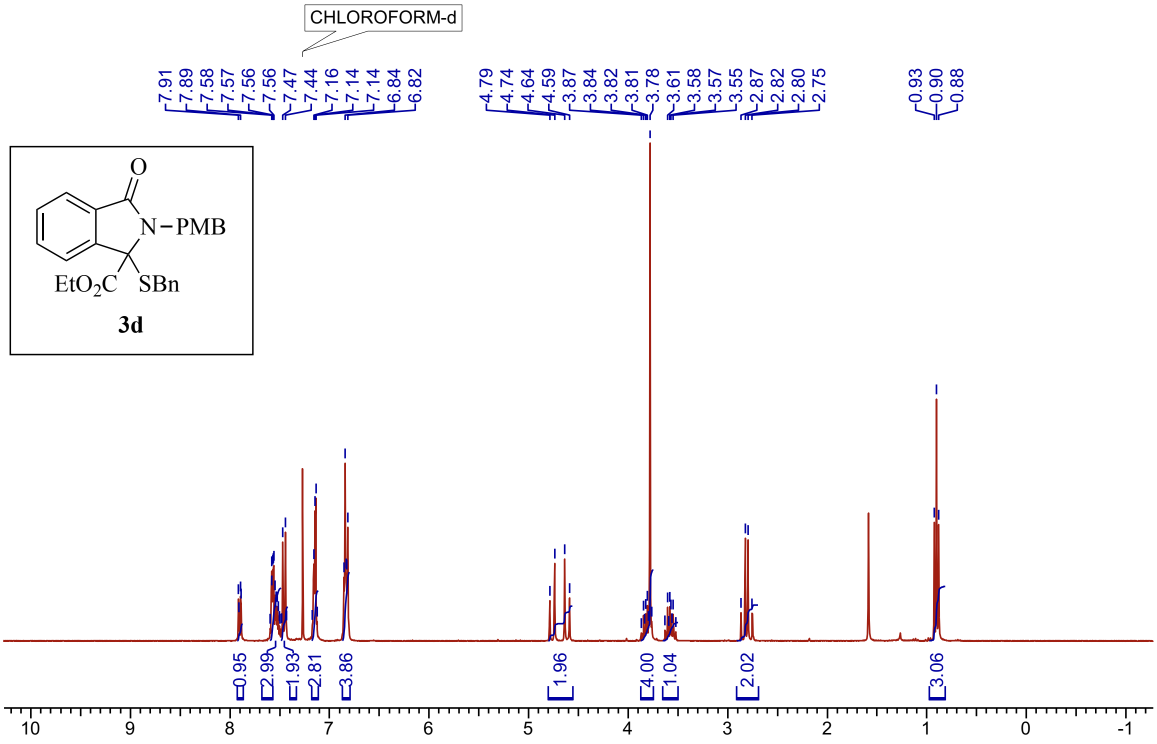

^{1}H-NMR (300 MHz, CDCl$_3$)

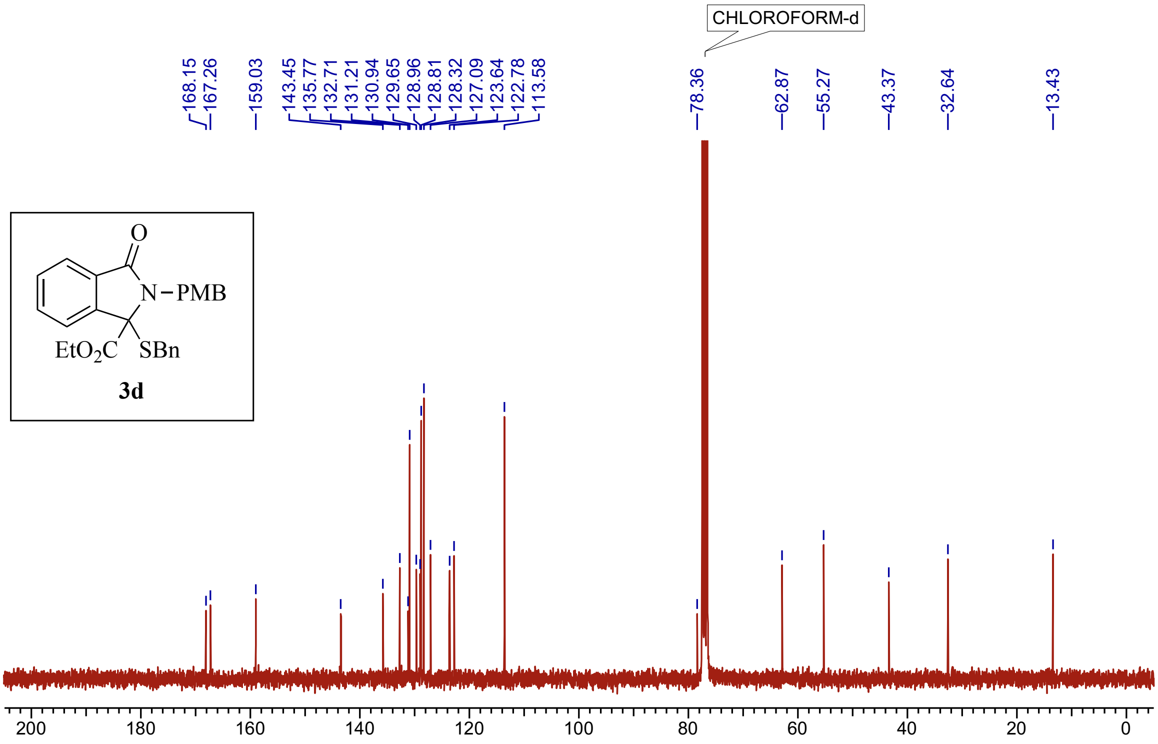

^{13}C-NMR (75 MHz, CDCl$_3$)

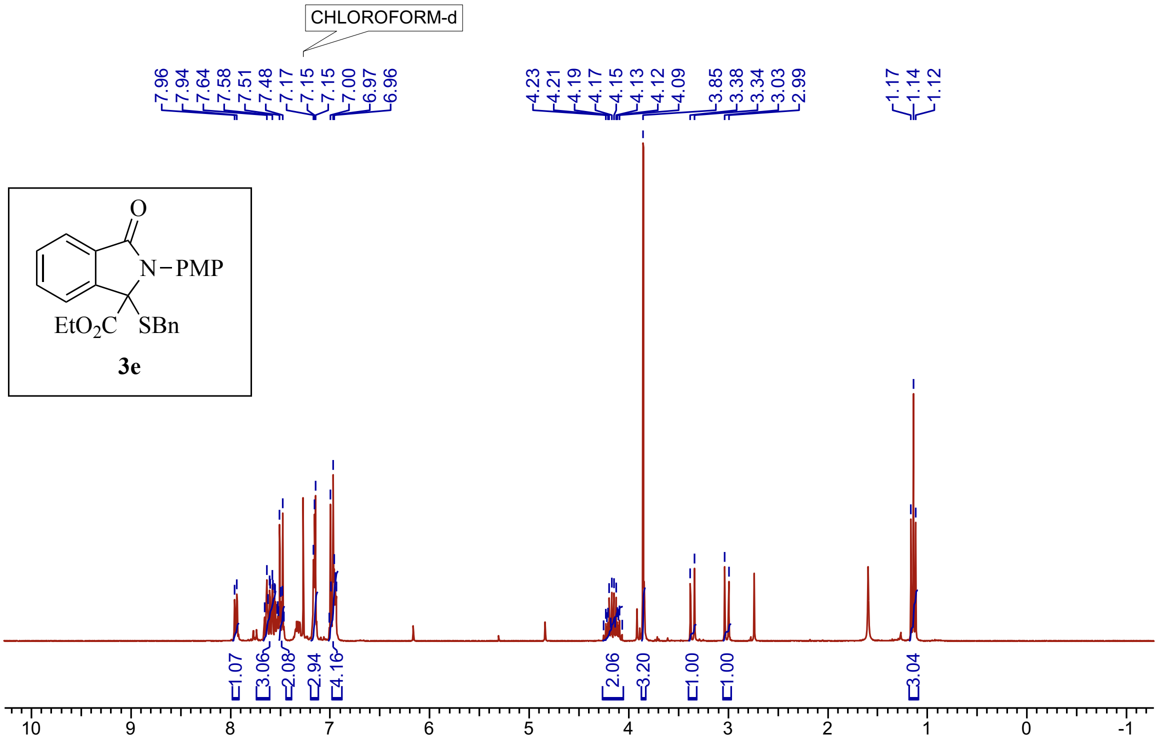

^{1}H-NMR (300 MHz, CDCl$_3$)

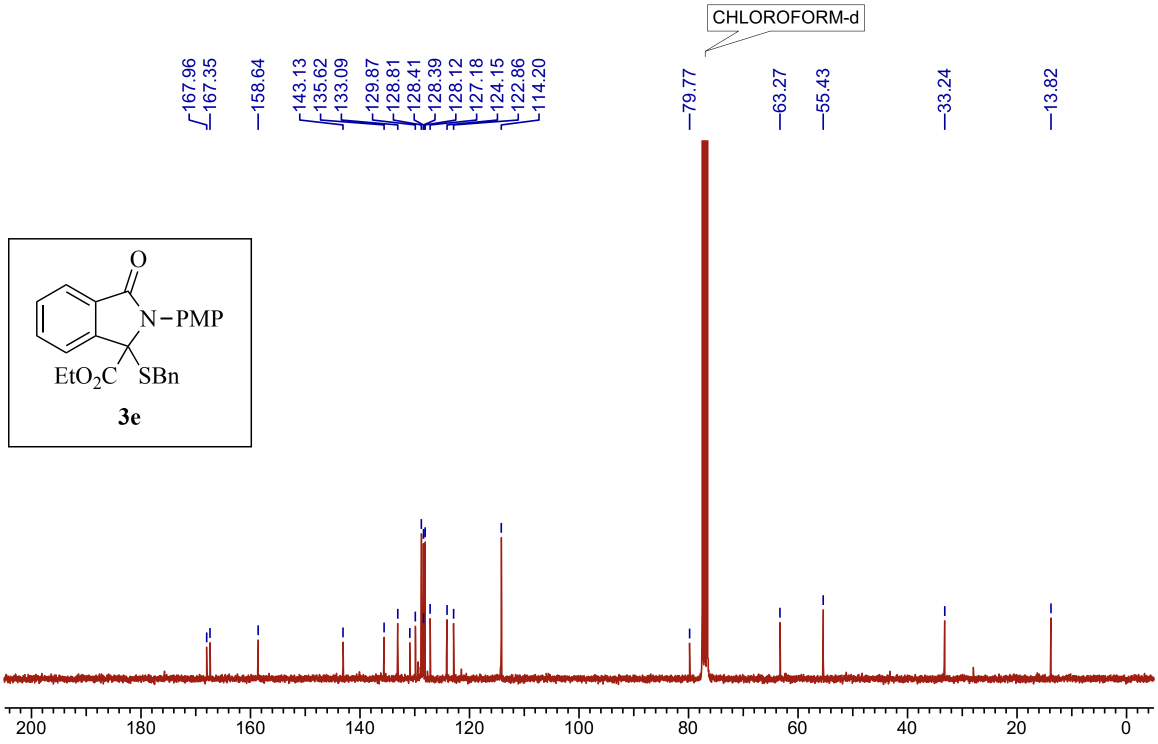

^{13}C-NMR (75 MHz, CDCl$_3$)

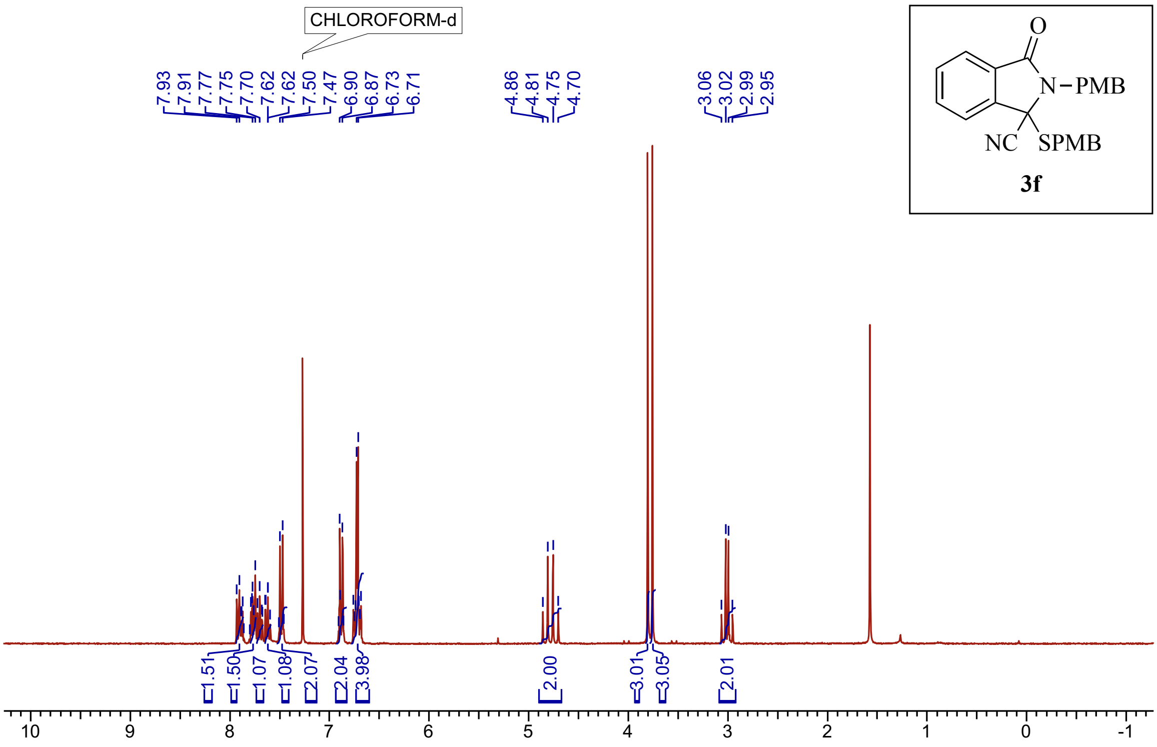

^{1}H-NMR (300 MHz, CDCl$_3$)

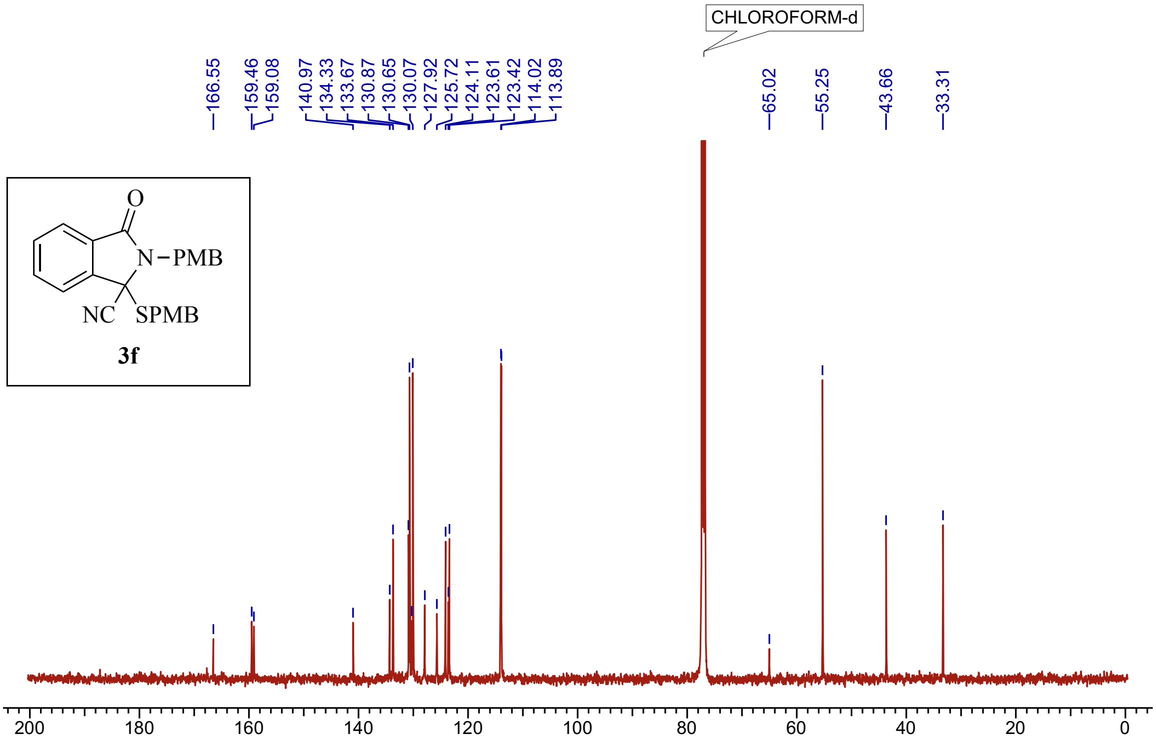

^{13}C-NMR (126 MHz, CDCl$_3$)

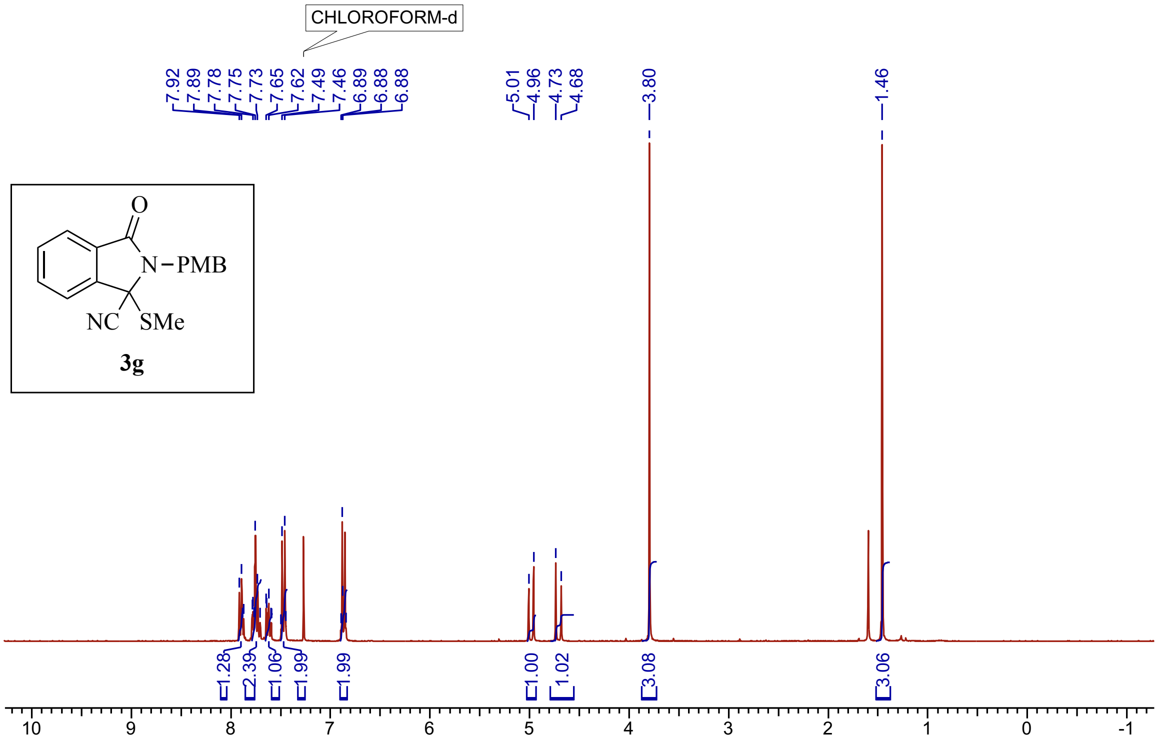

^{1}H-NMR (300 MHz, $CDCl_3$)

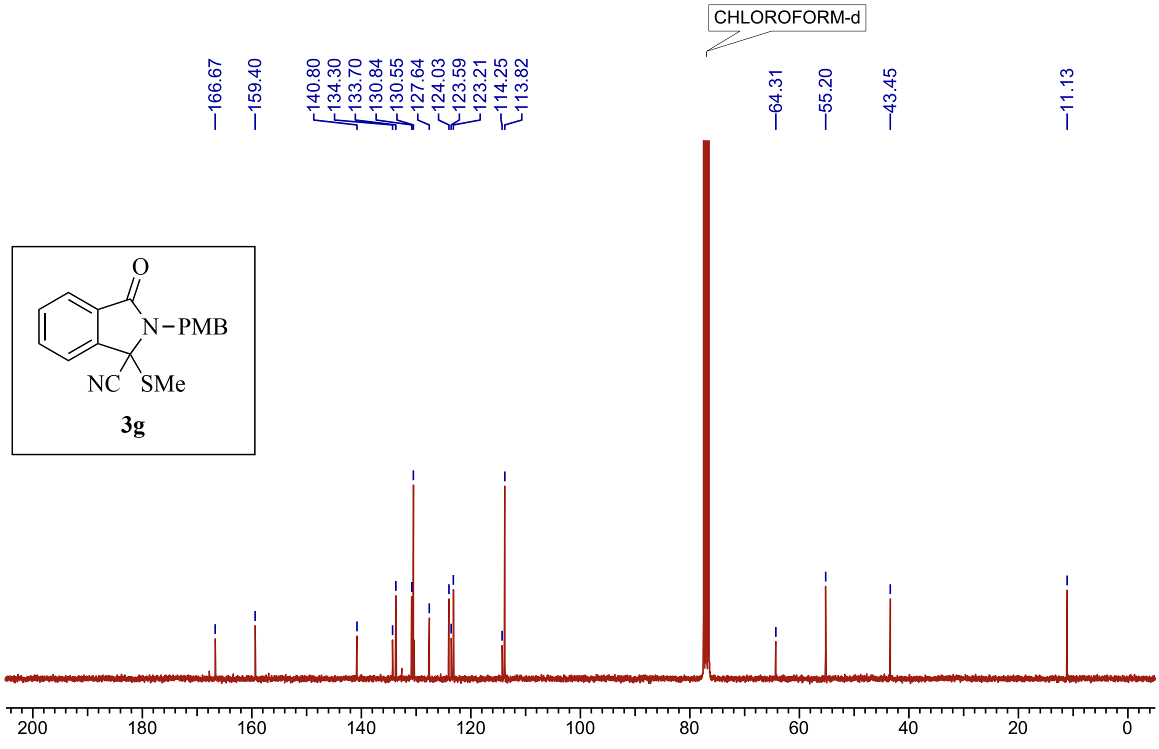

^{13}C-NMR (75 MHz, $CDCl_3$)

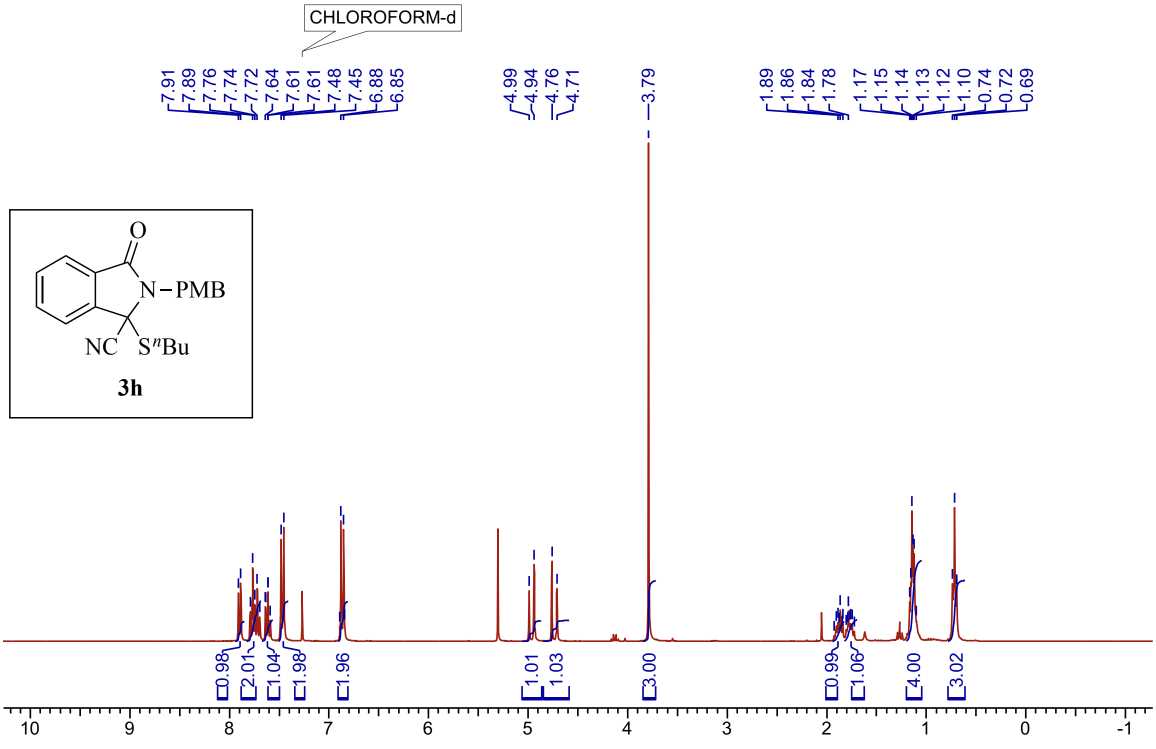

^{1}H-NMR (300 MHz, $CDCl_3$)

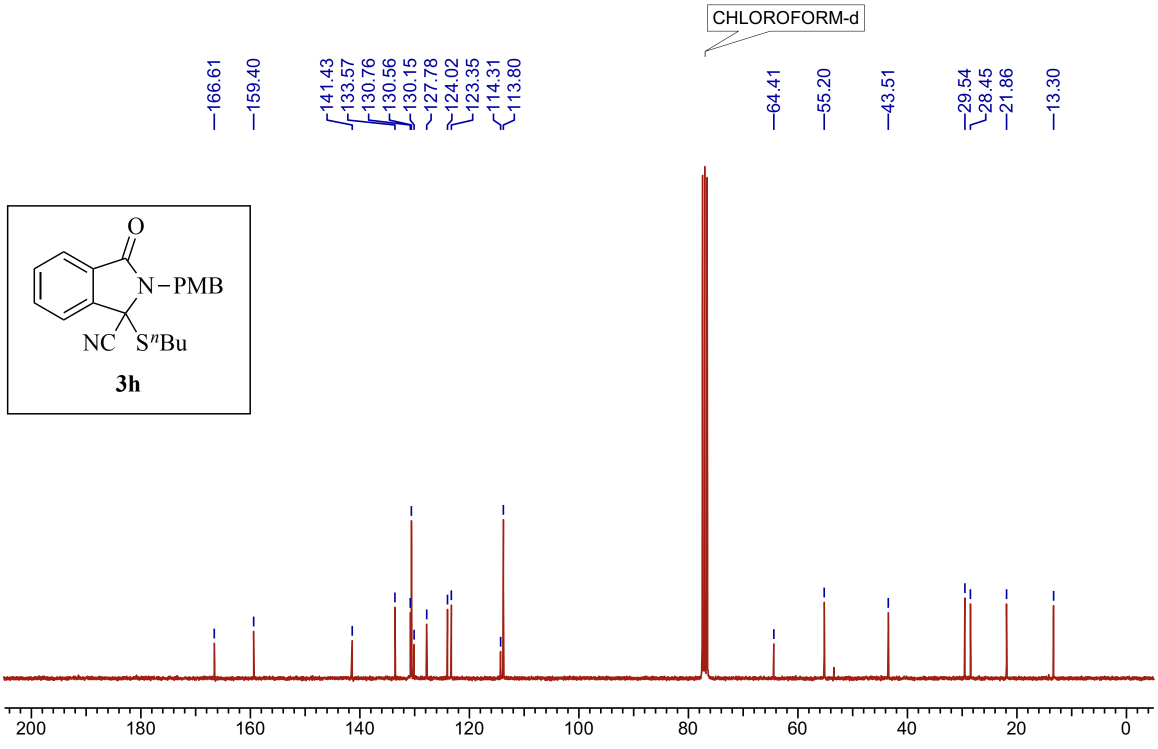

^{13}C-NMR (75 MHz, $CDCl_3$)

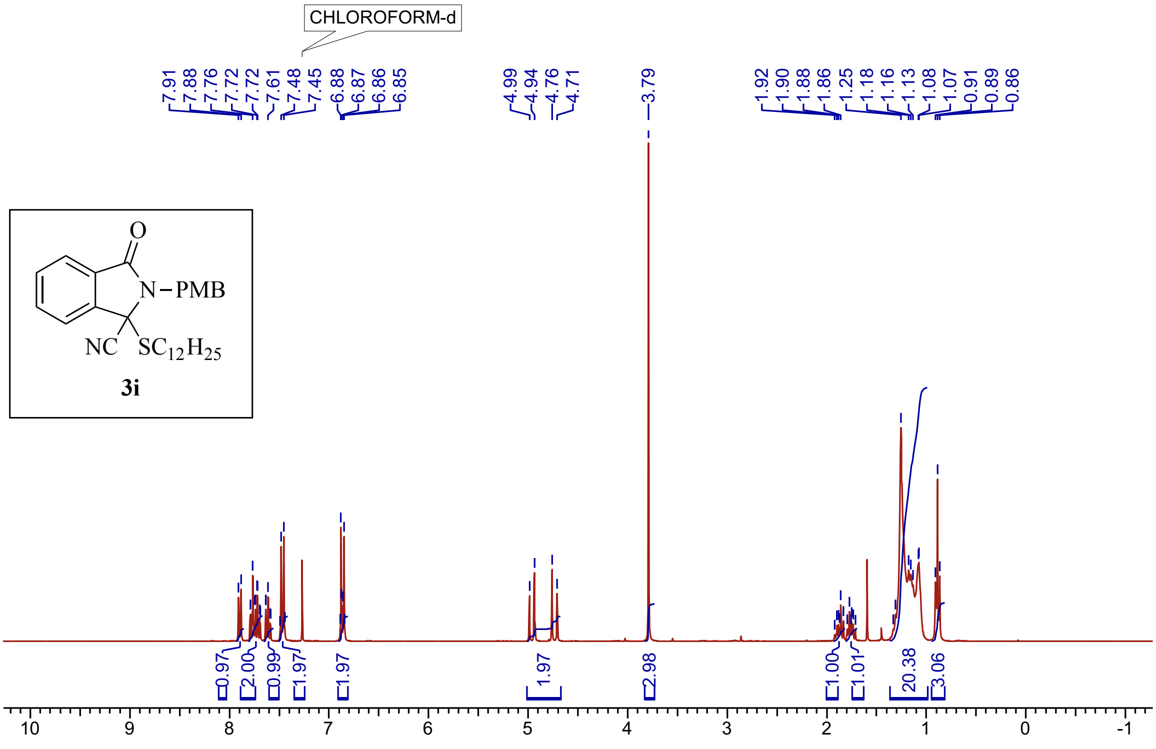

^{1}H-NMR (300 MHz, $CDCl_3$)

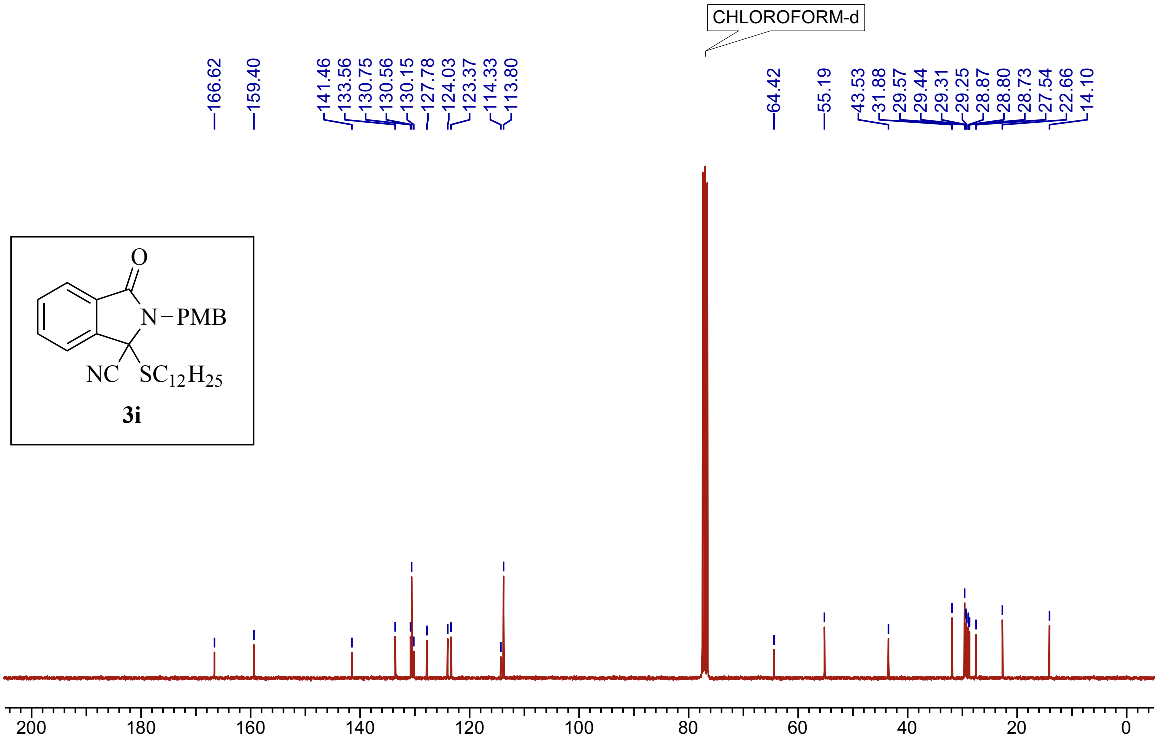

^{13}C-NMR (75 MHz, $CDCl_3$)

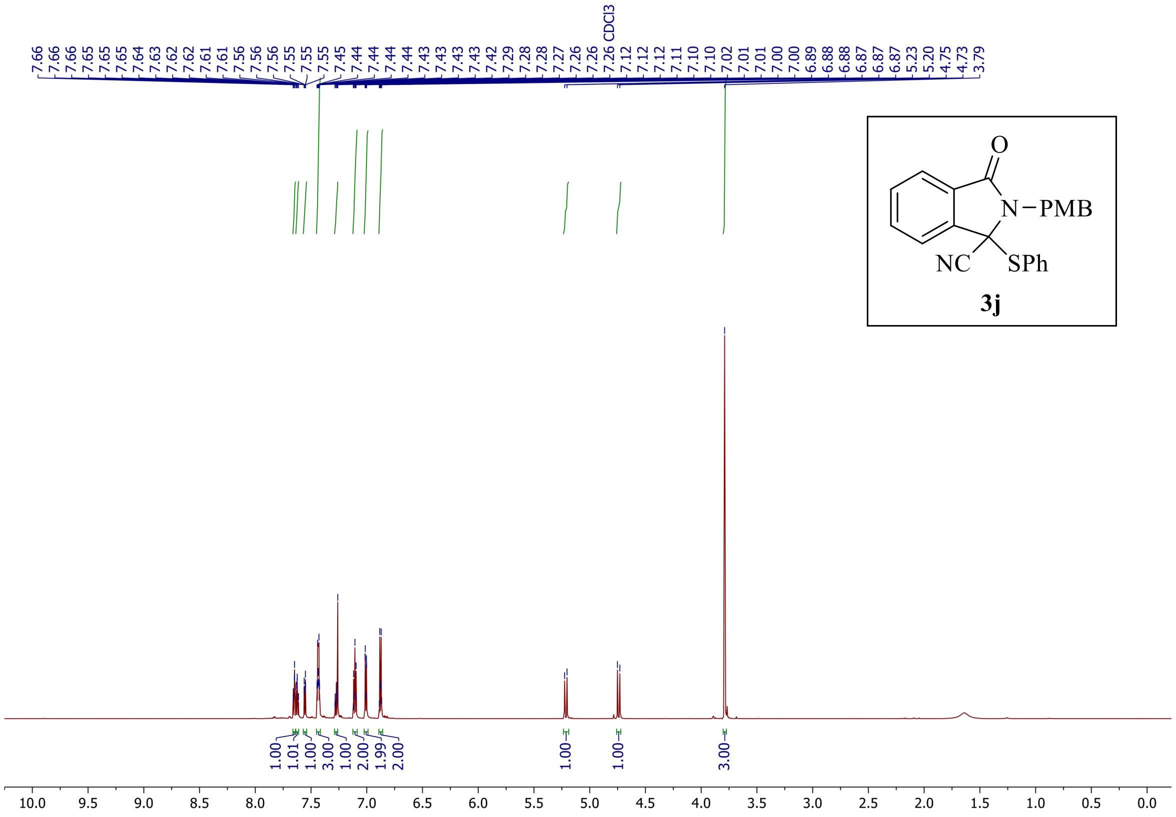

^{1}H-NMR (700 MHz, CDCl$_3$)

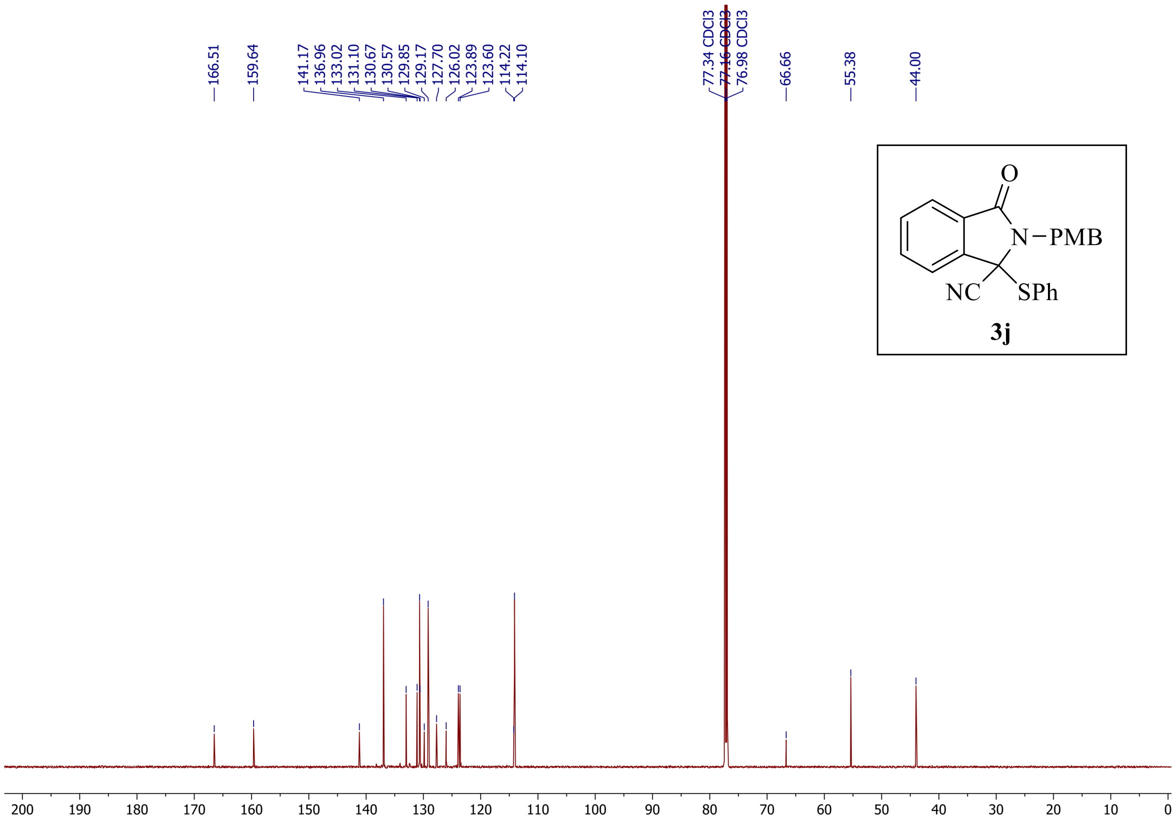

^{13}C-NMR (176 MHz, CDCl$_3$)

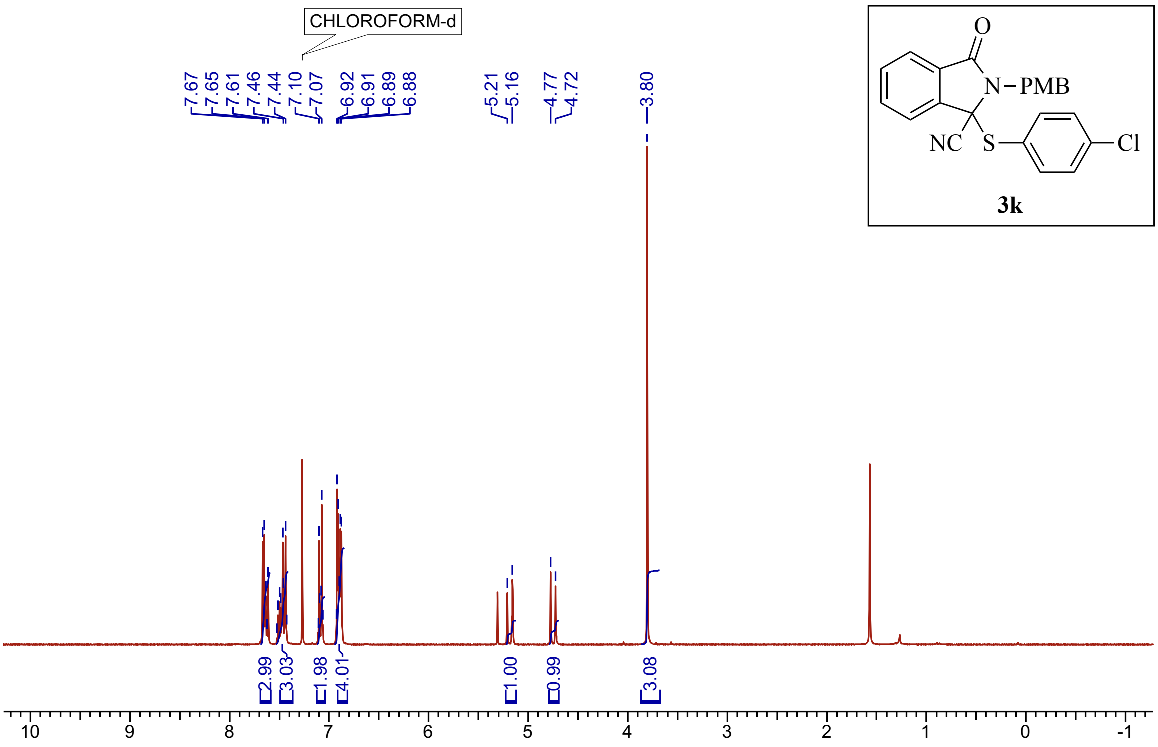

^{1}H-NMR (300 MHz, CDCl$_3$)

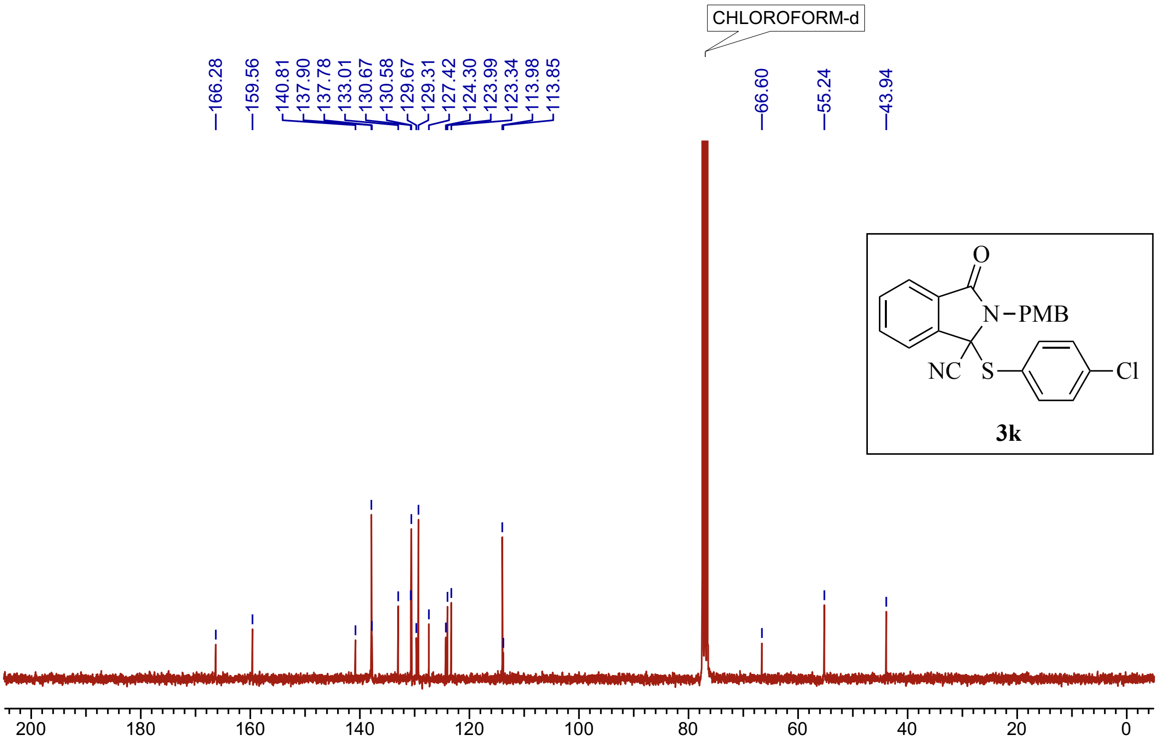

^{13}C-NMR (75 MHz, CDCl$_3$)

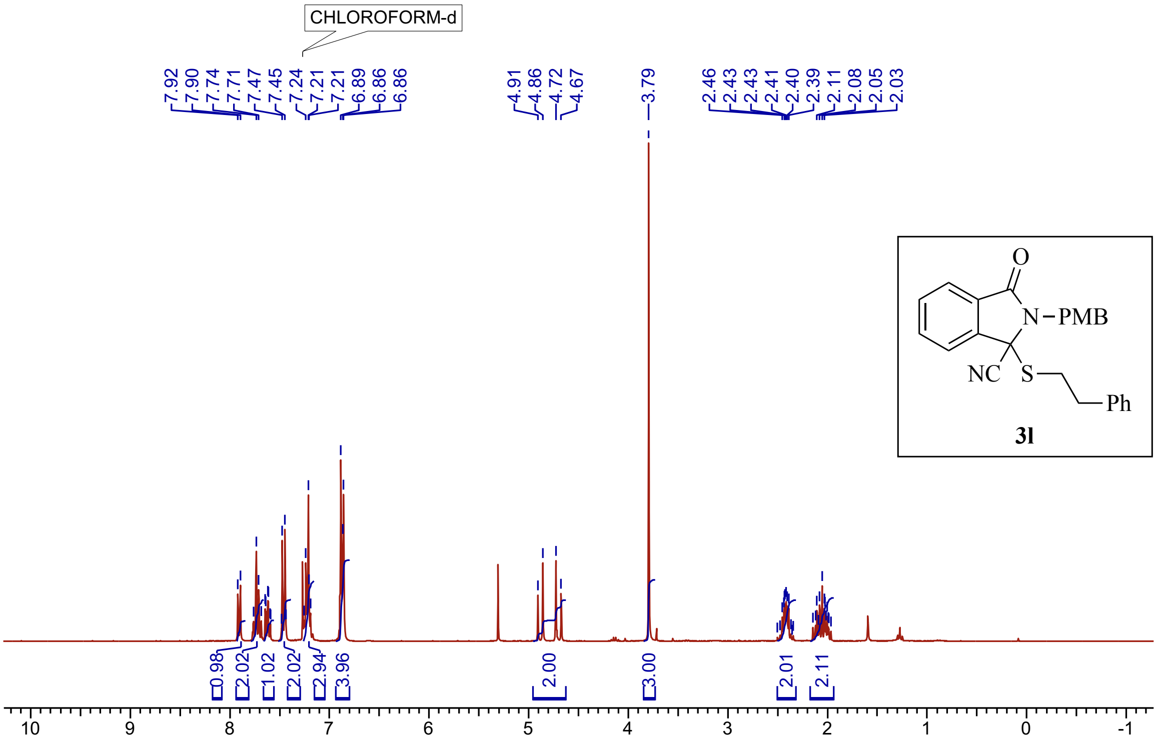

^{1}H-NMR (300 MHz, $CDCl_3$)

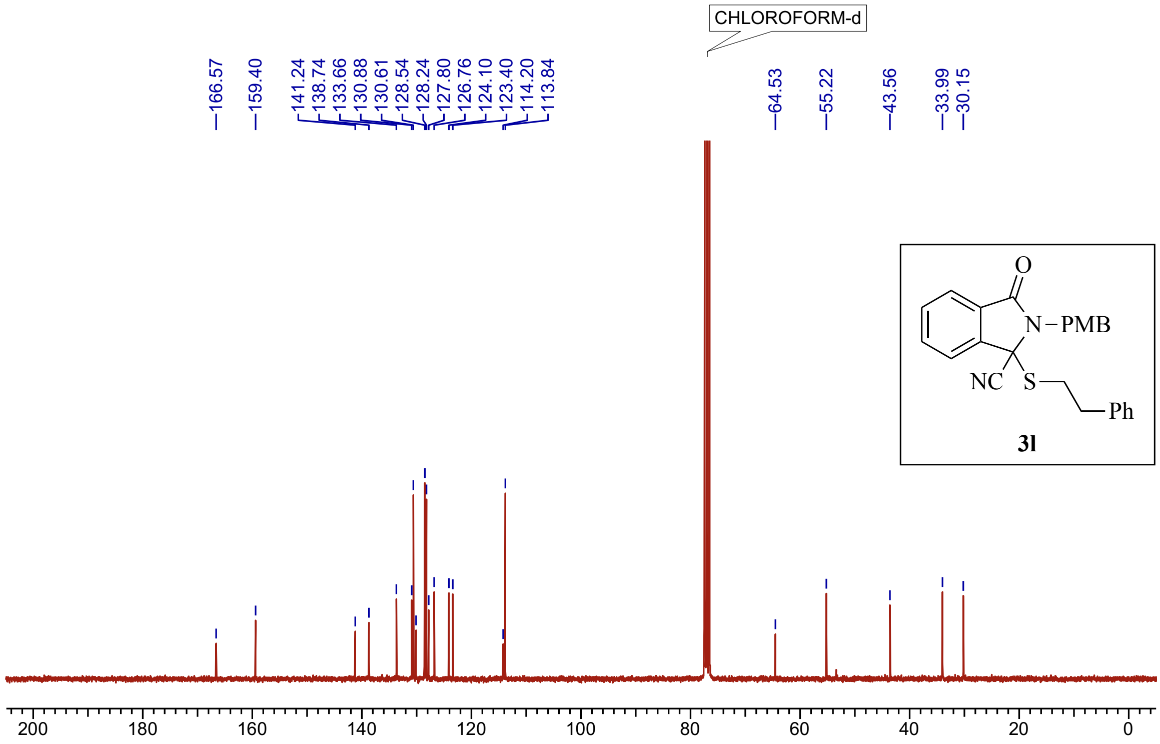

^{13}C-NMR (75 MHz, $CDCl_3$)

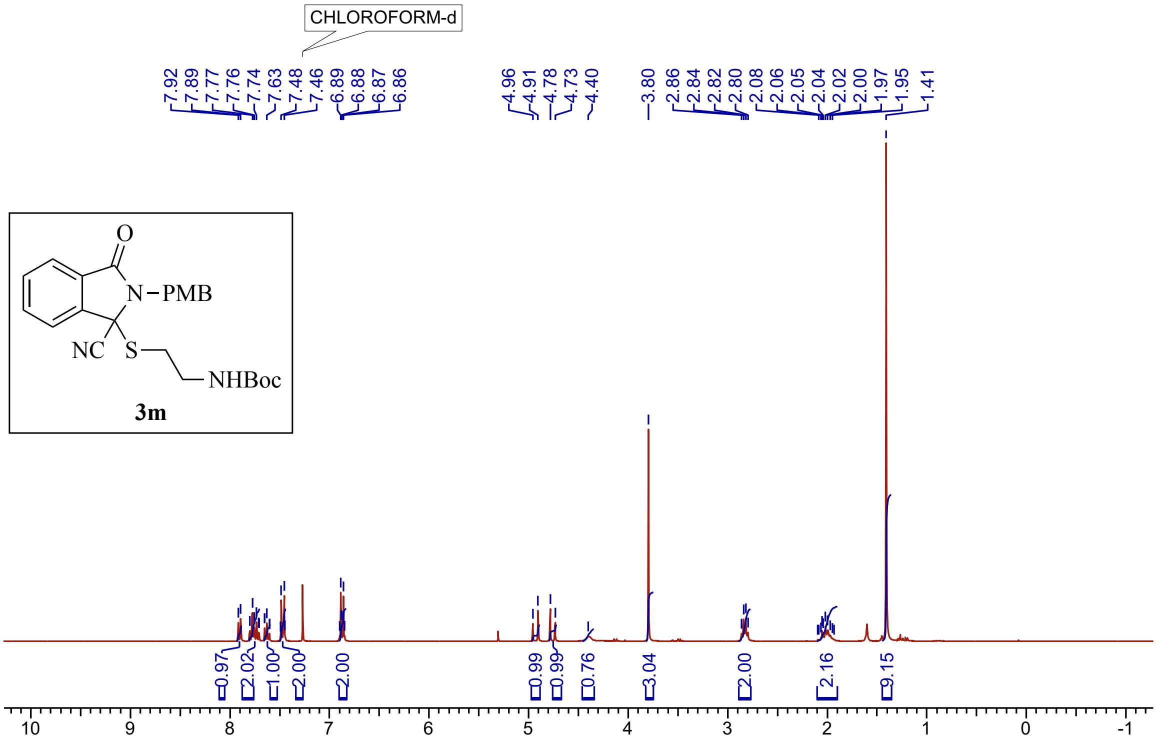

^{1}H-NMR (300 MHz, $CDCl_3$)

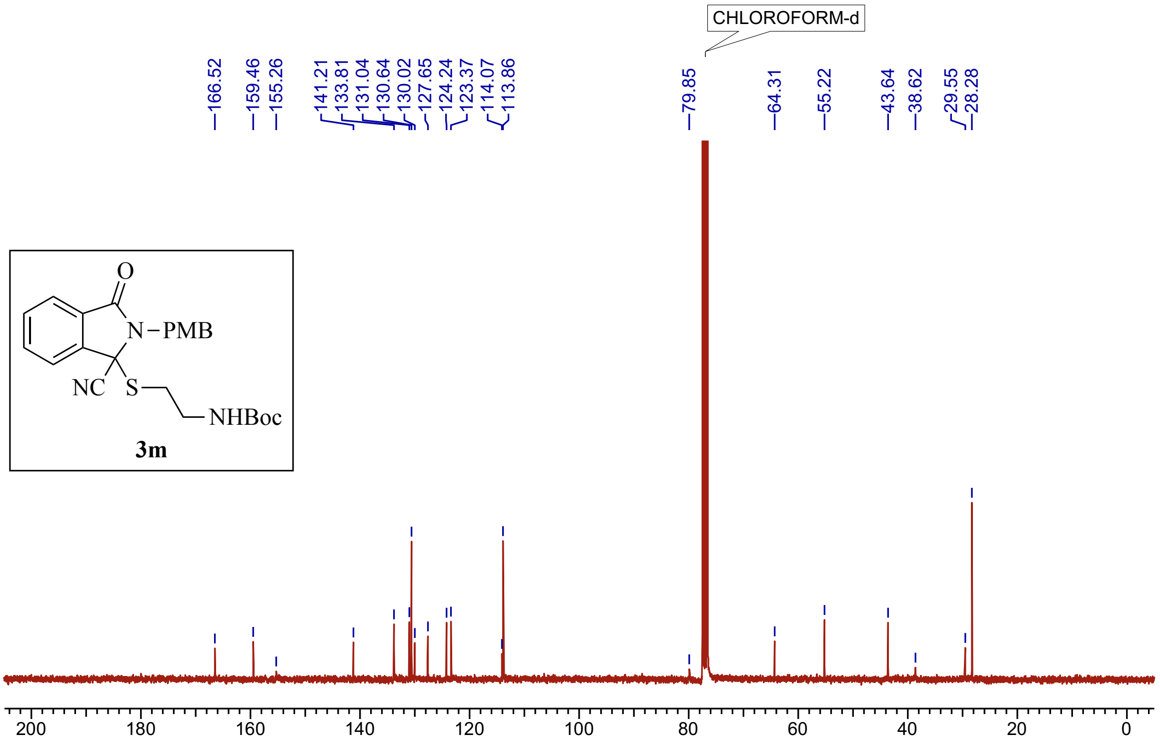

^{13}C-NMR (75 MHz, $CDCl_3$)

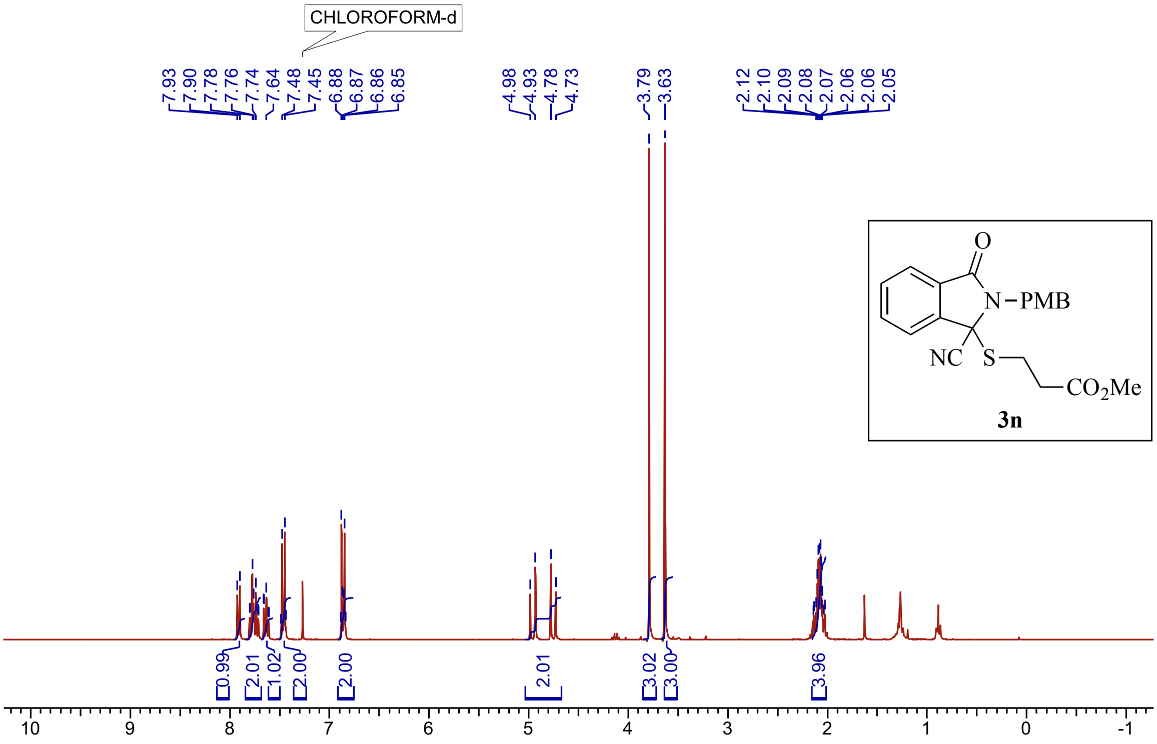

^{1}H-NMR (300 MHz, $CDCl_3$)

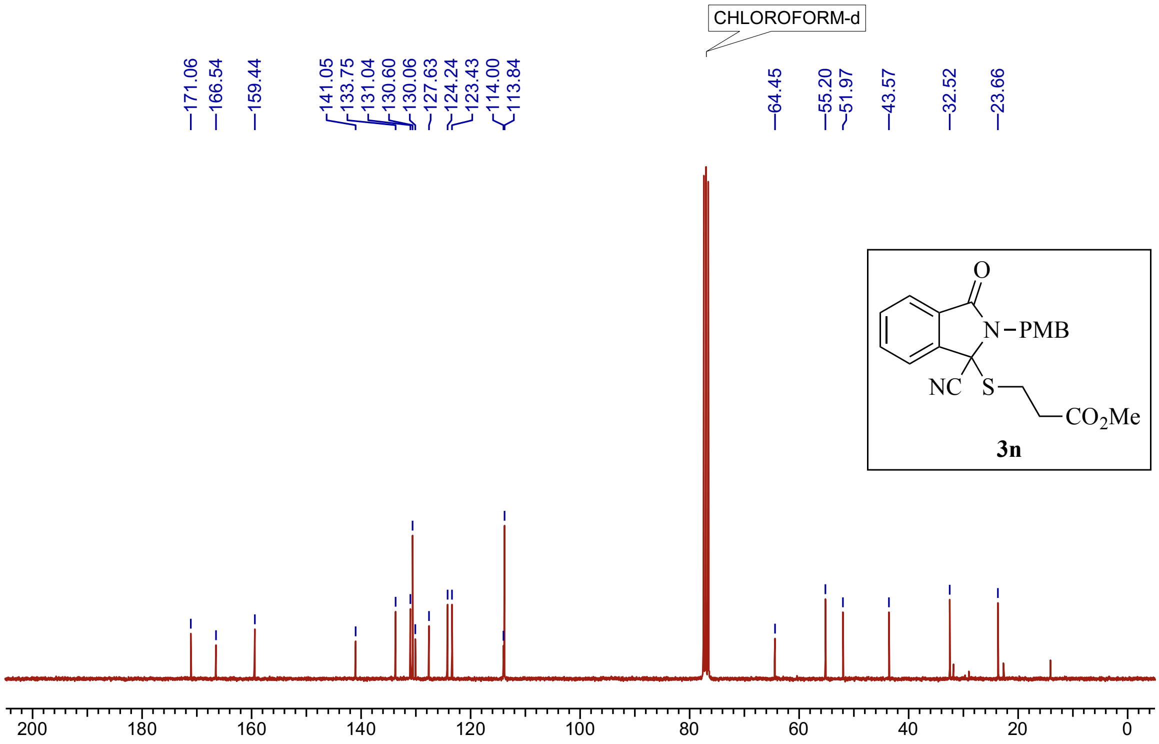

^{13}C-NMR (75 MHz, $CDCl_3$)

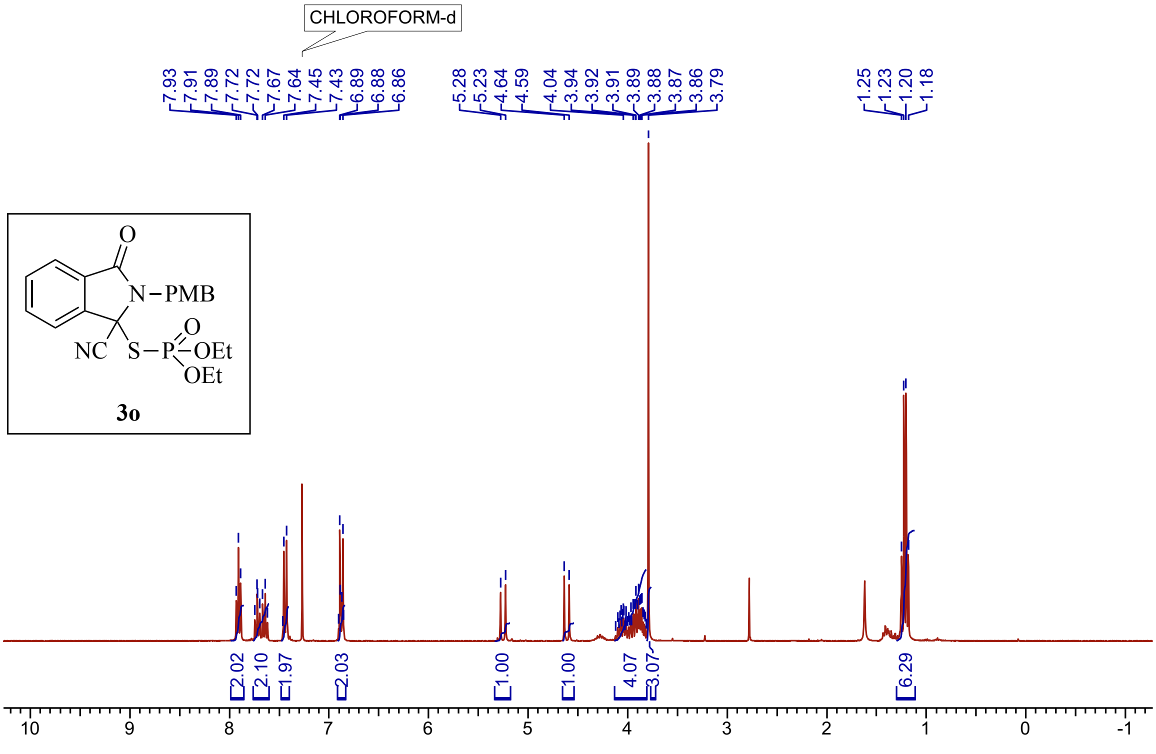

^{1}H-NMR (300 MHz, $CDCl_3$)

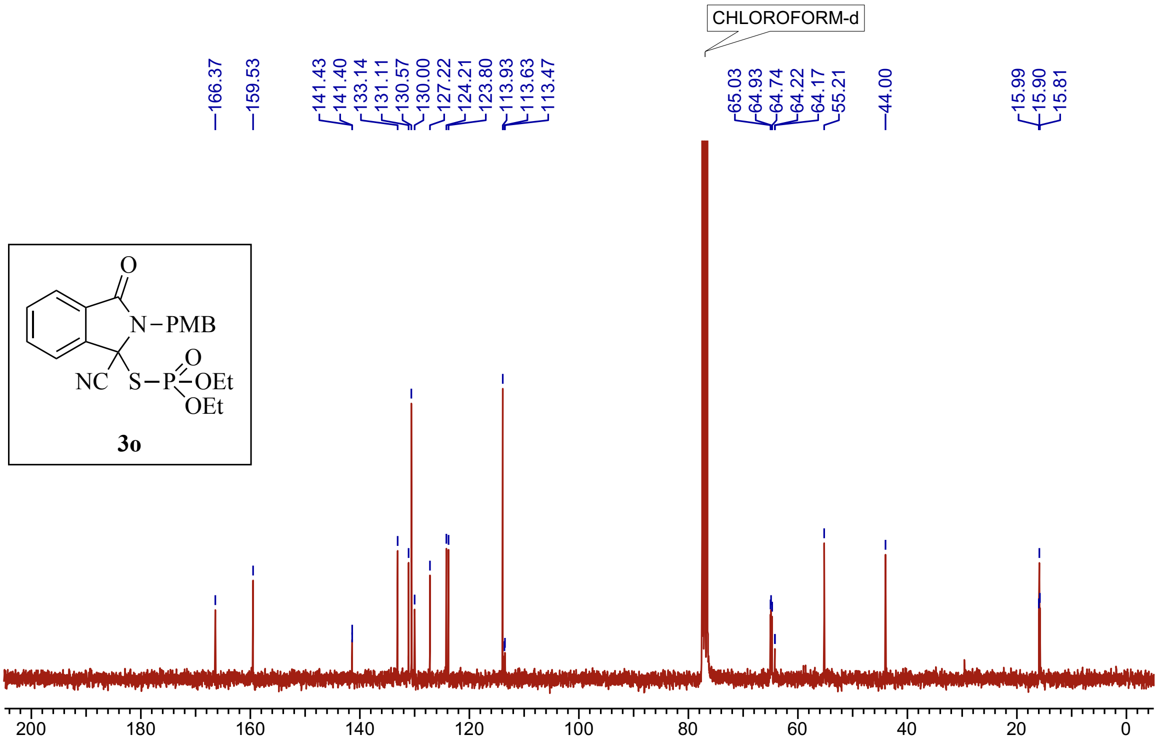

^{13}C-NMR (75 MHz, $CDCl_3$)

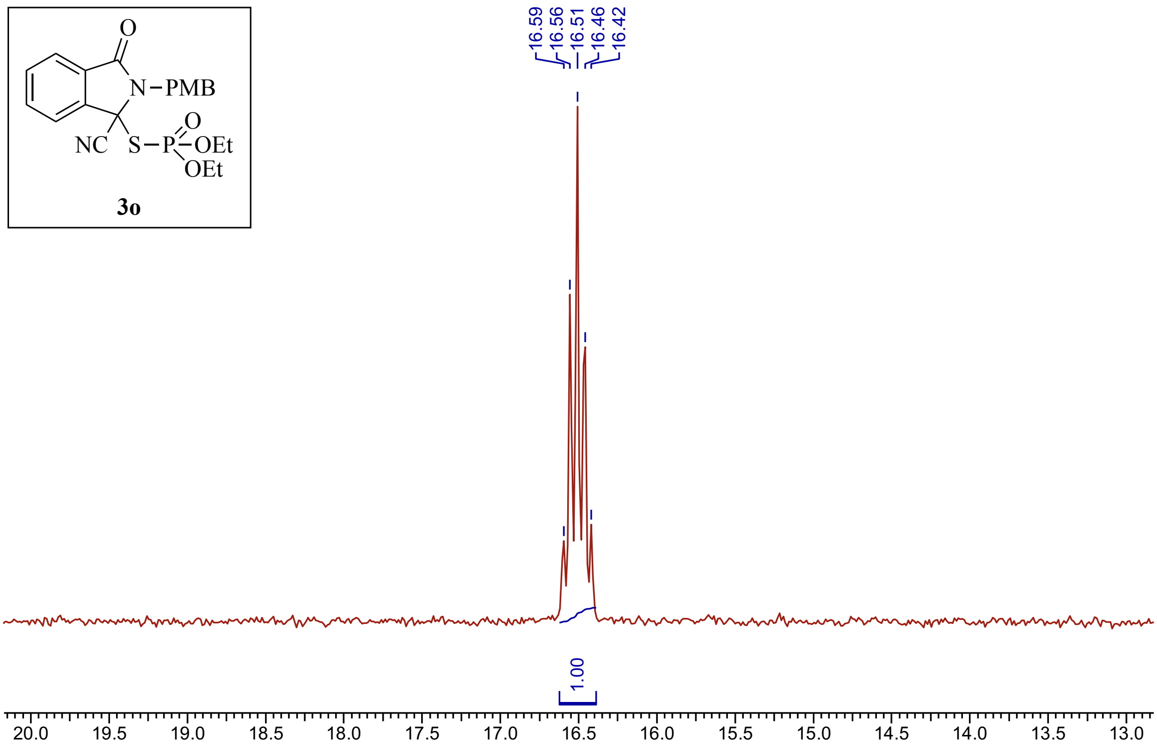

^{31}P-NMR (202 MHz, $CDCl_3$)

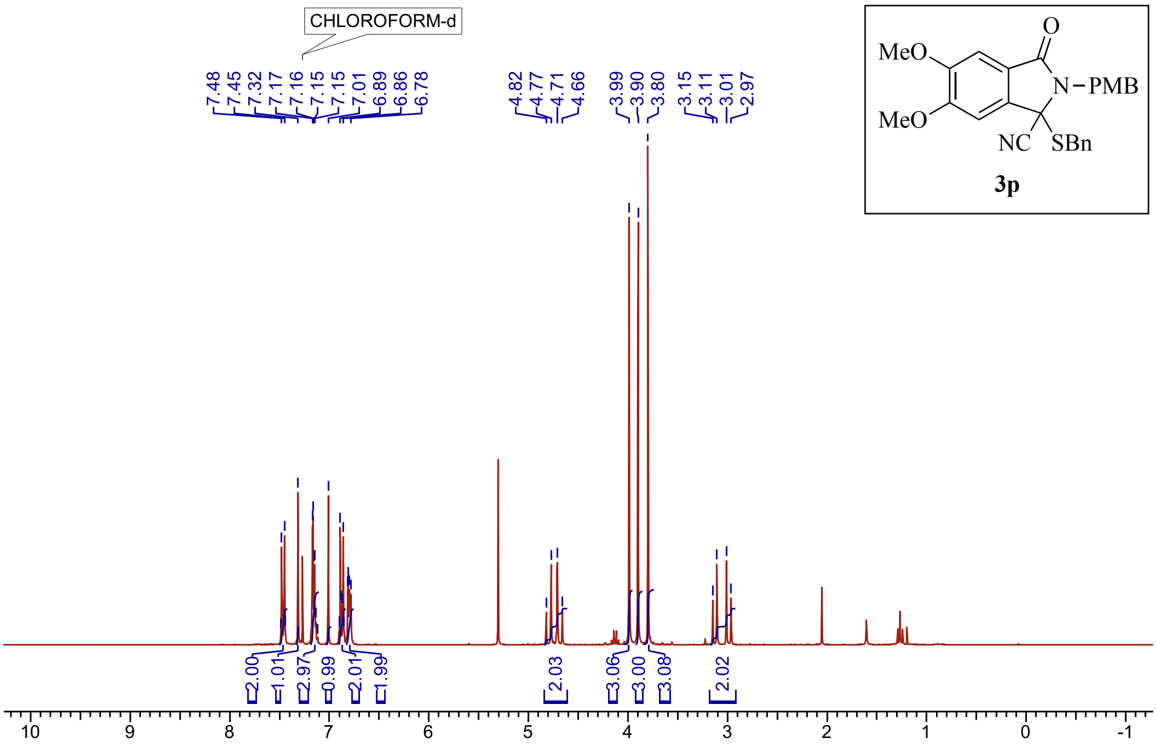

^{1}H-NMR (300 MHz, $CDCl_3$)

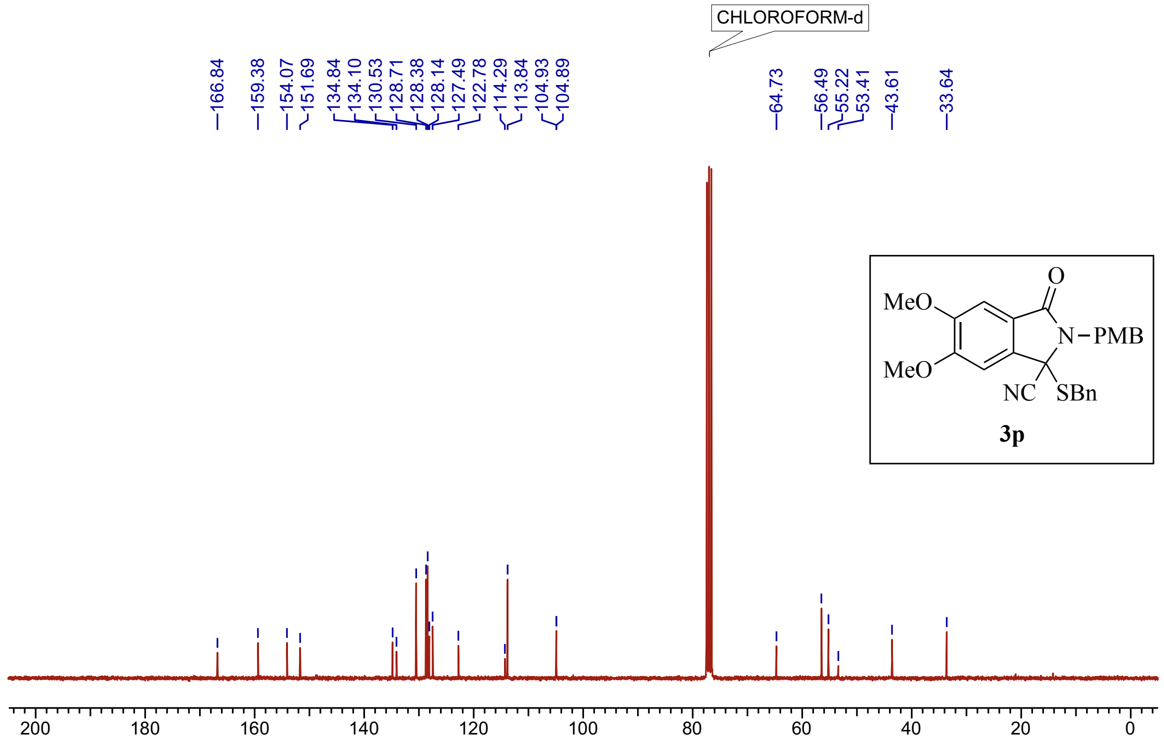

^{13}C-NMR (75 MHz, $CDCl_3$)

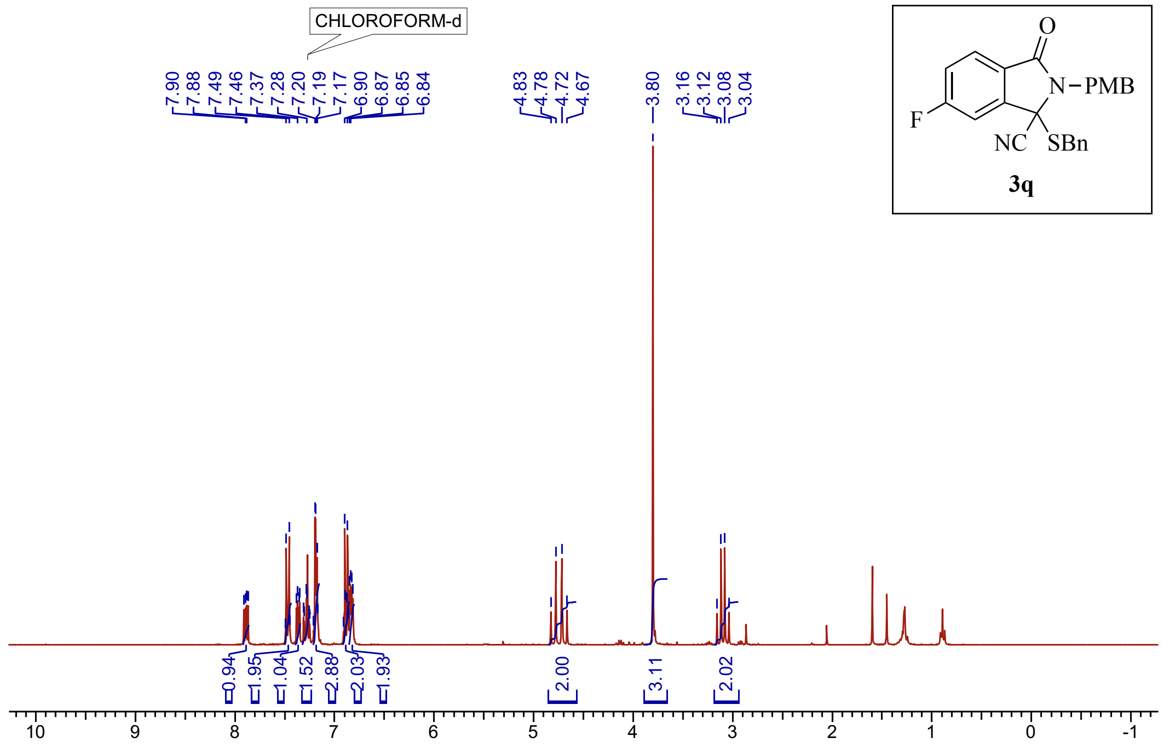

^{1}H-NMR (300 MHz, $CDCl_3$)

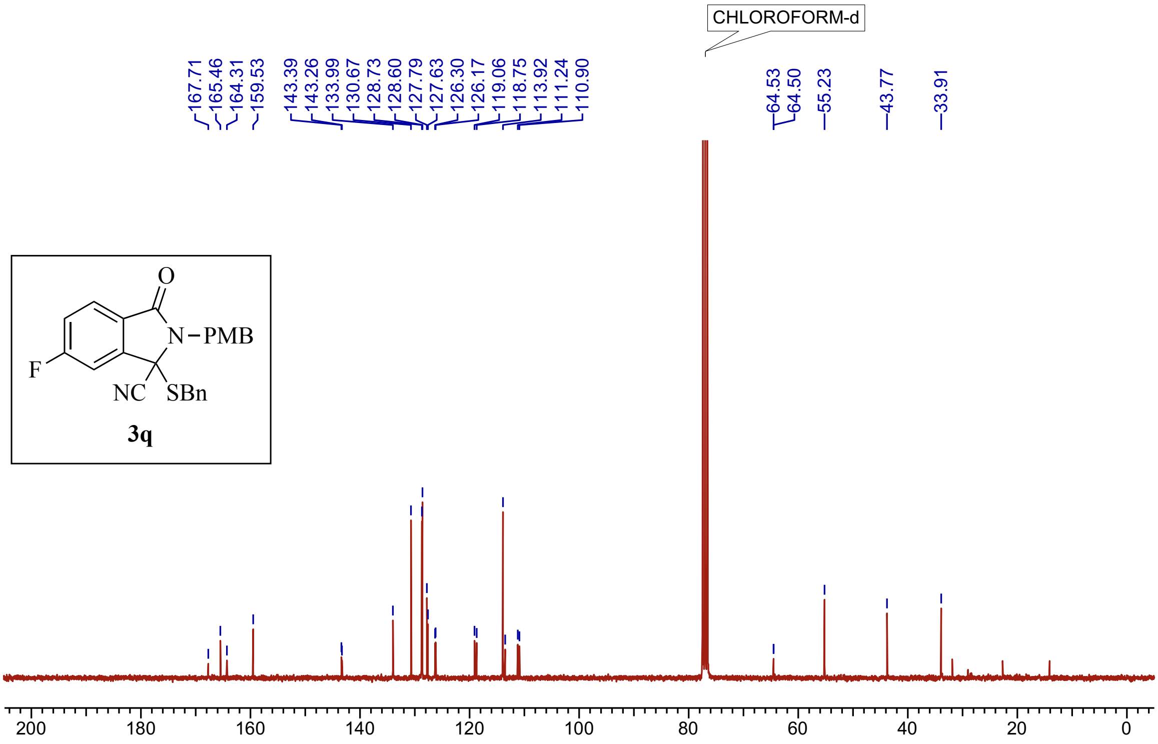

^{13}C-NMR (75 MHz, $CDCl_3$)

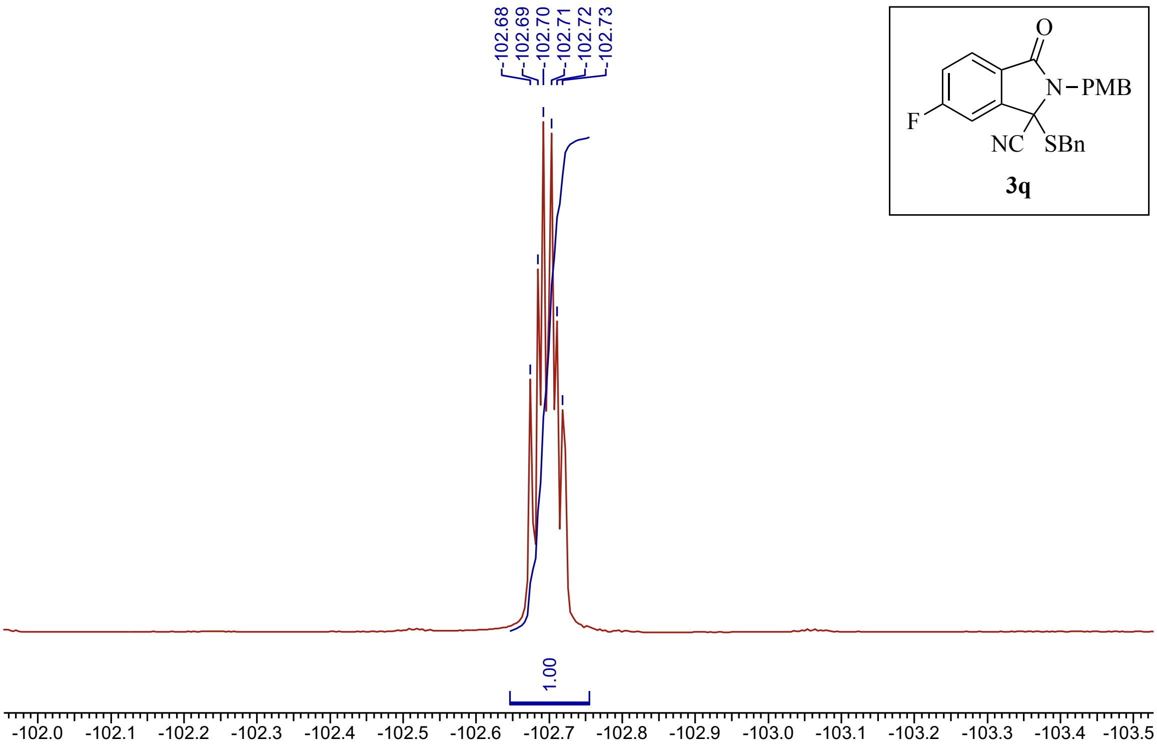

^{19}F-NMR (471 MHz, $CDCl_3$)

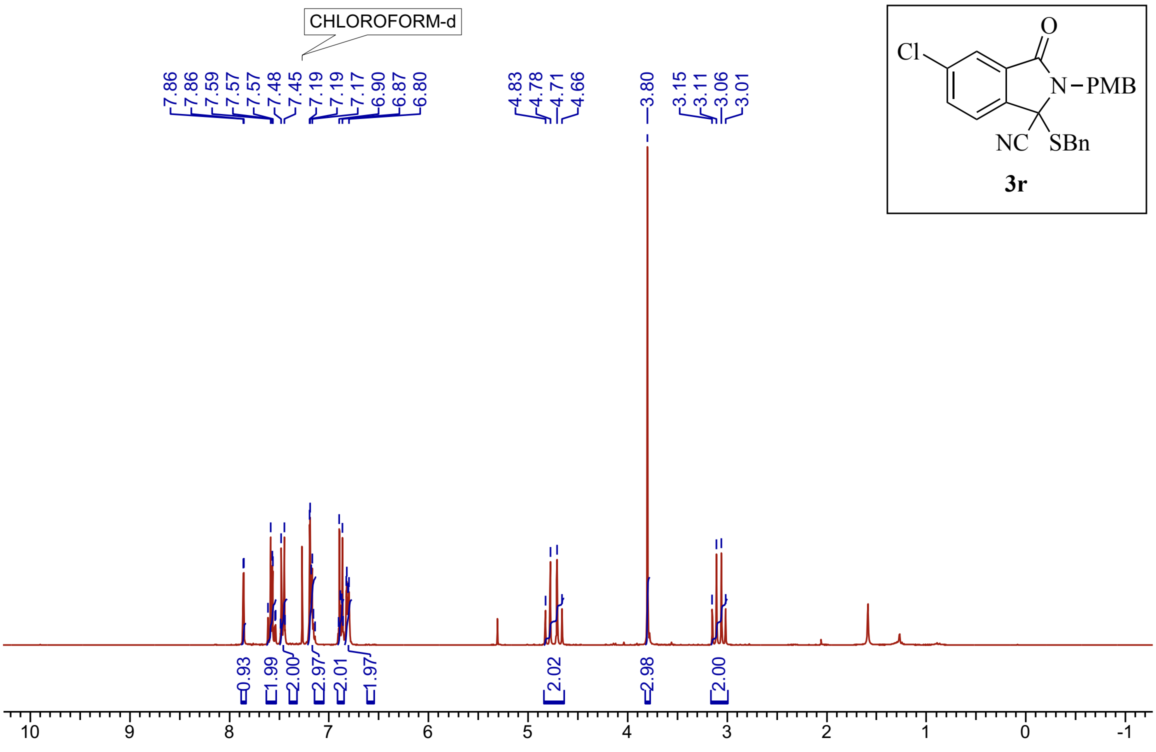

^{1}H-NMR (300 MHz, $CDCl_3$)

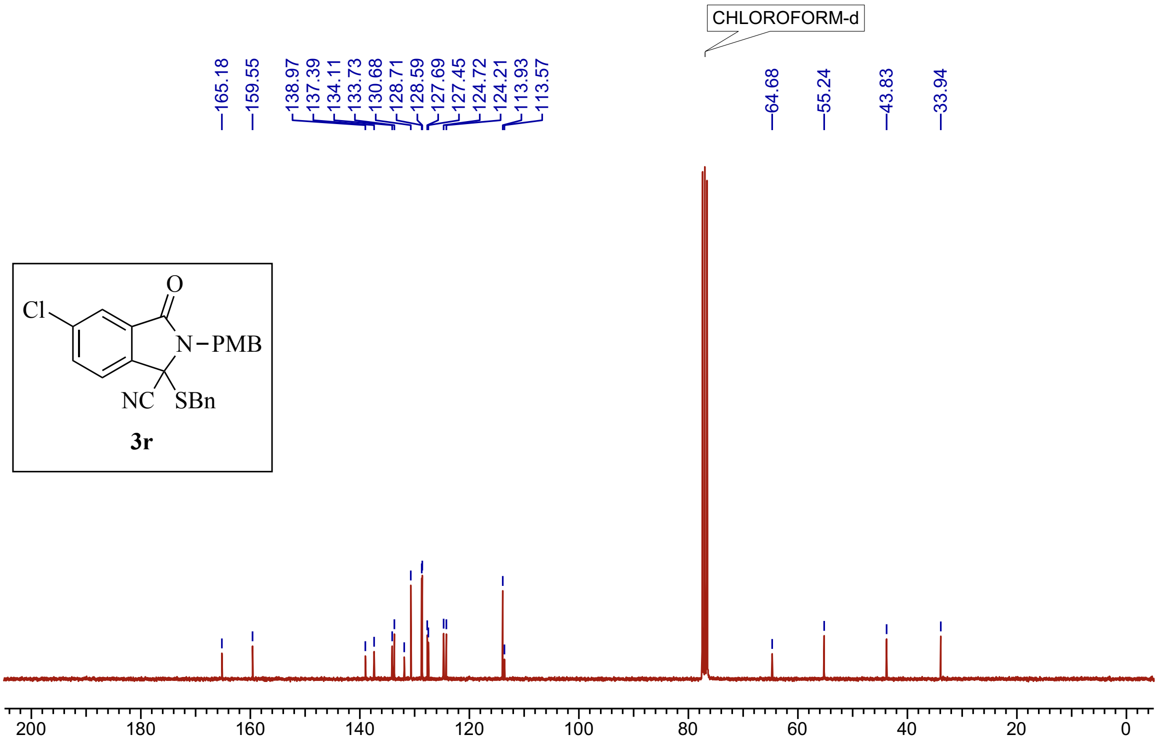

^{13}C-NMR (75 MHz, $CDCl_3$)

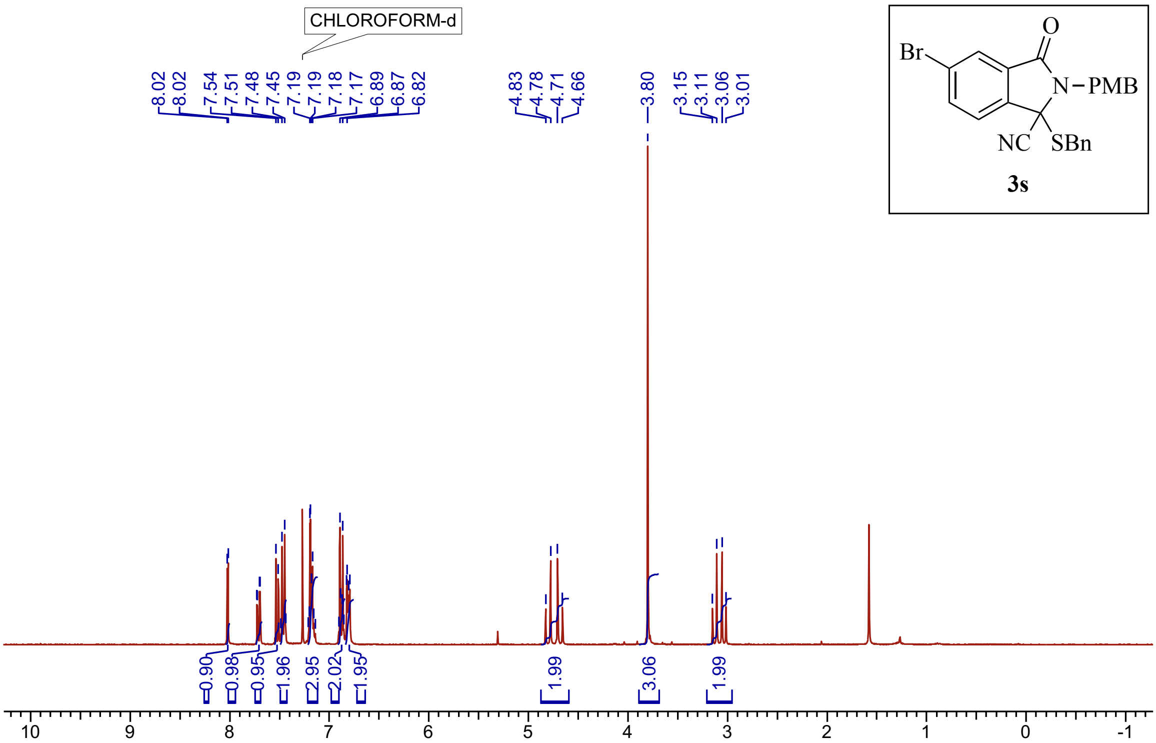

^{1}H-NMR (300 MHz, $CDCl_3$)

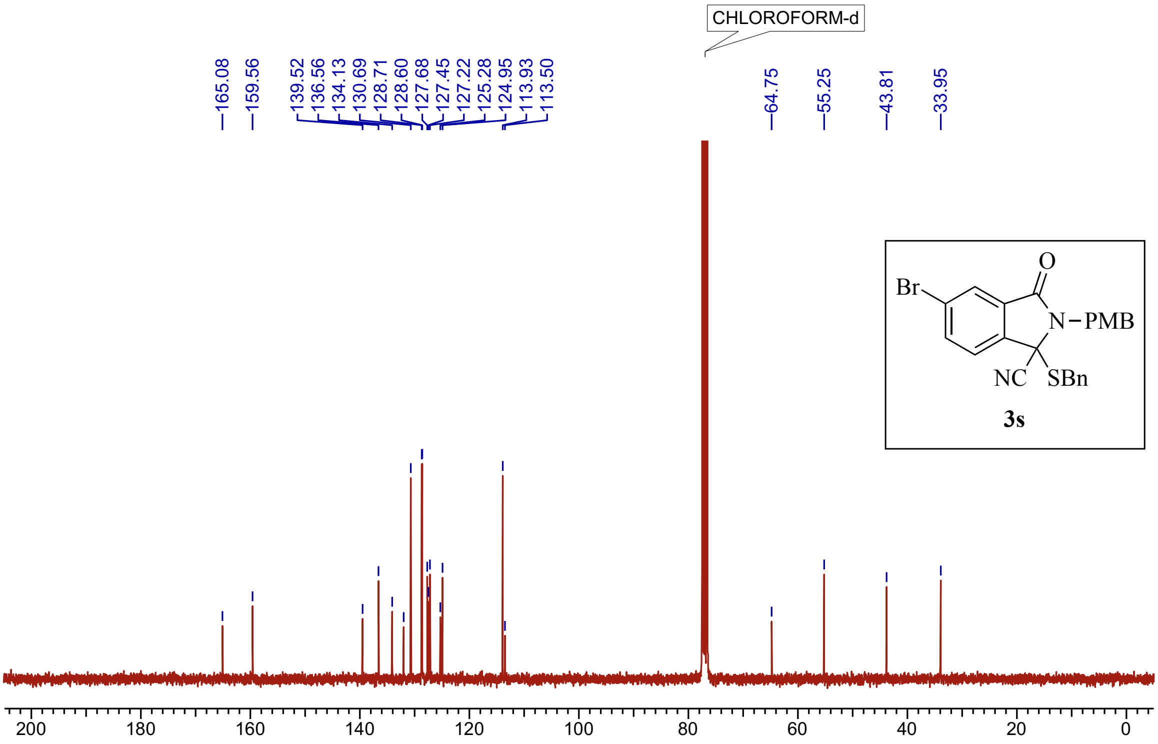

^{13}C-NMR (75 MHz, $CDCl_3$)

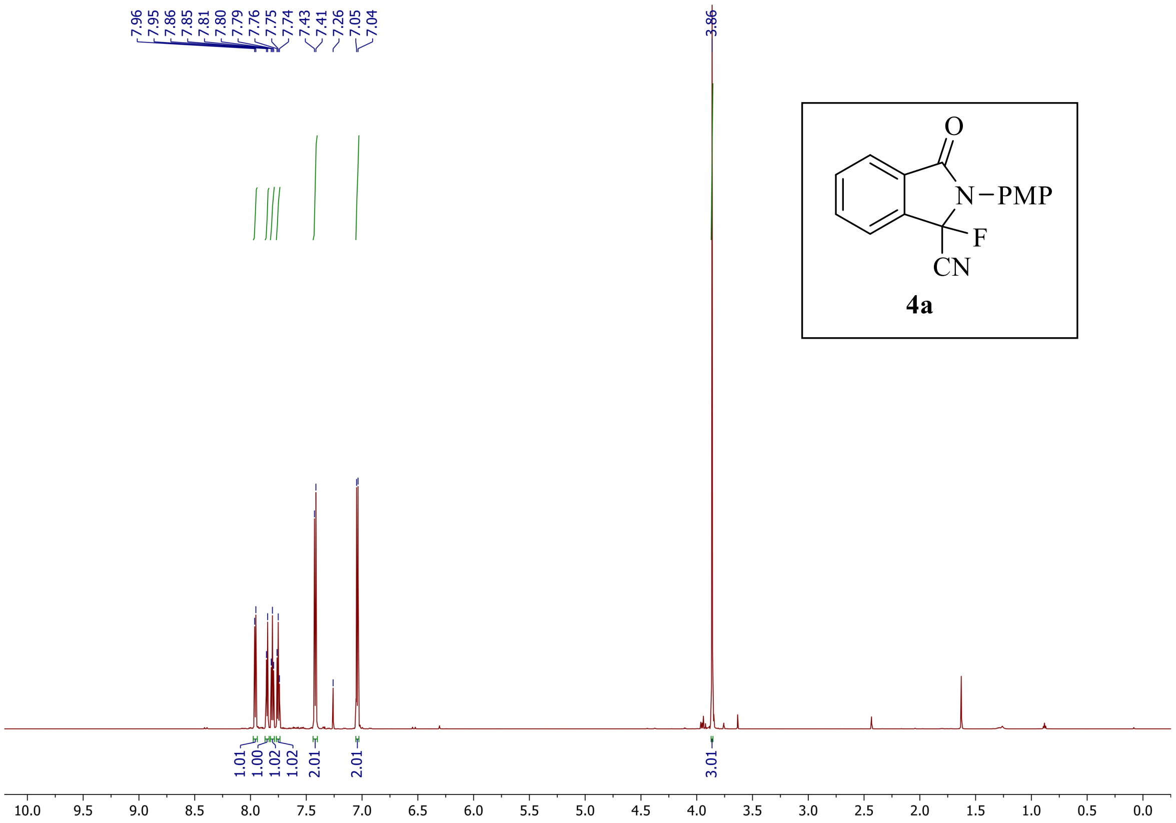

^{1}H-NMR (700 MHz, $CDCl_3$)

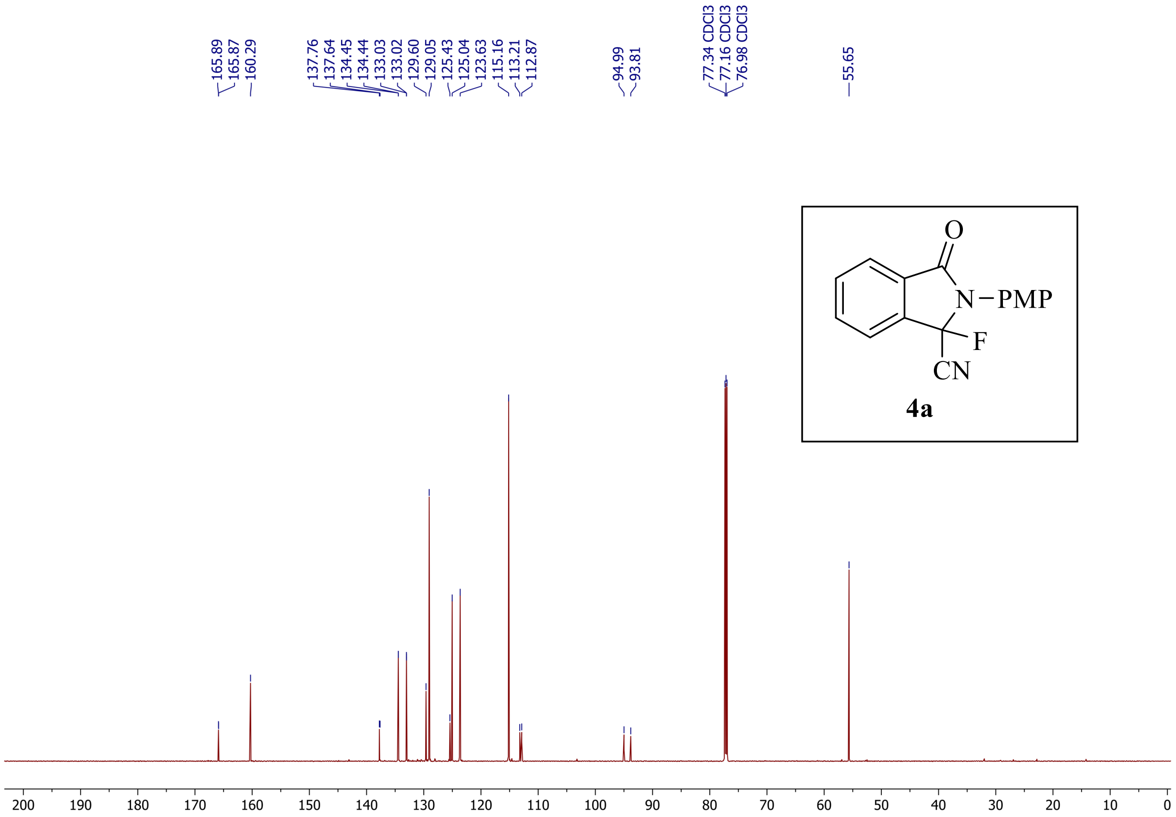

^{13}C-NMR (75 MHz, $CDCl_3$)

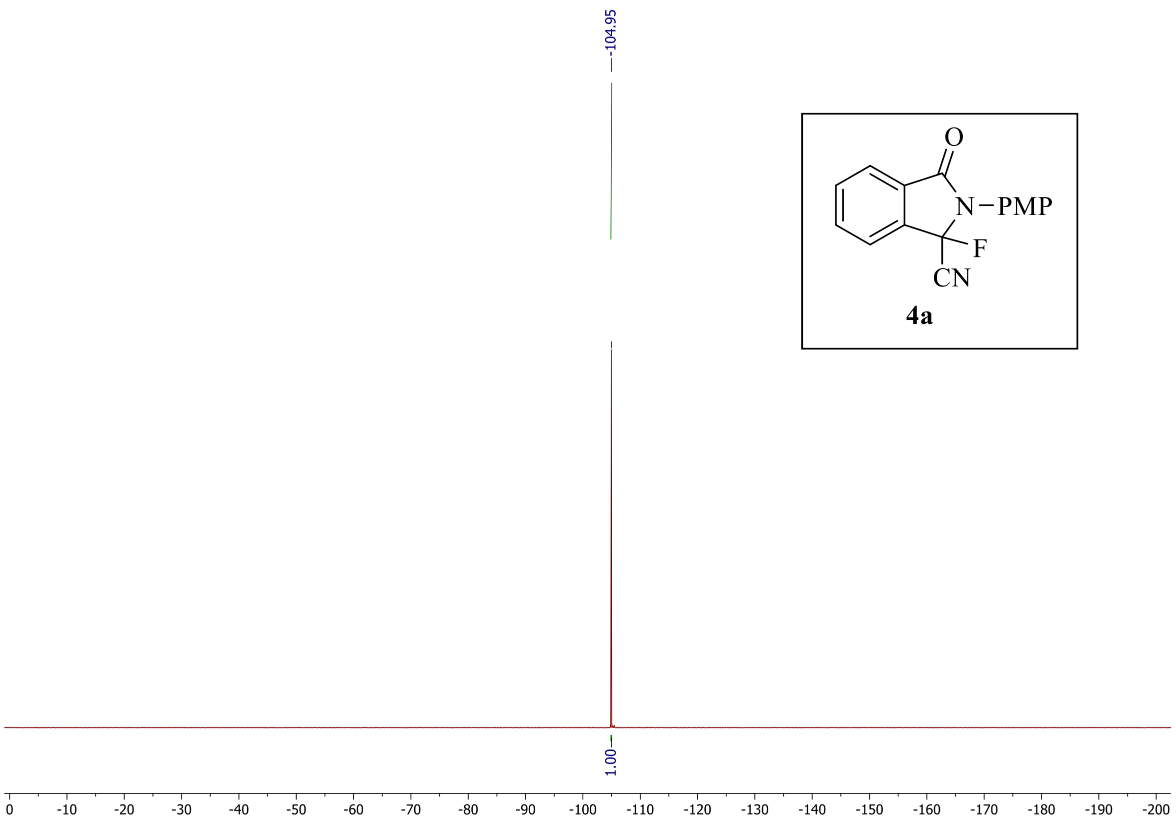

^{19}F-NMR (282 MHz, $CDCl_3$)

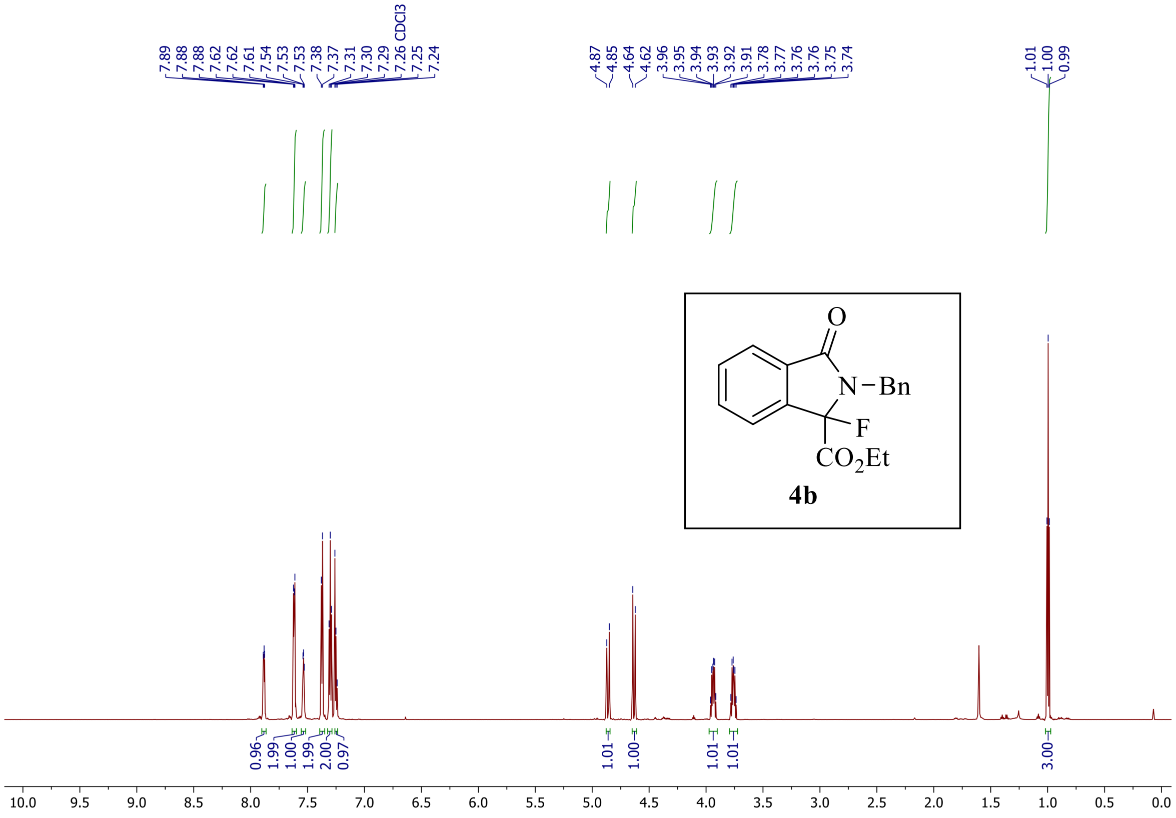

^{1}H-NMR (700 MHz, $CDCl_3$)

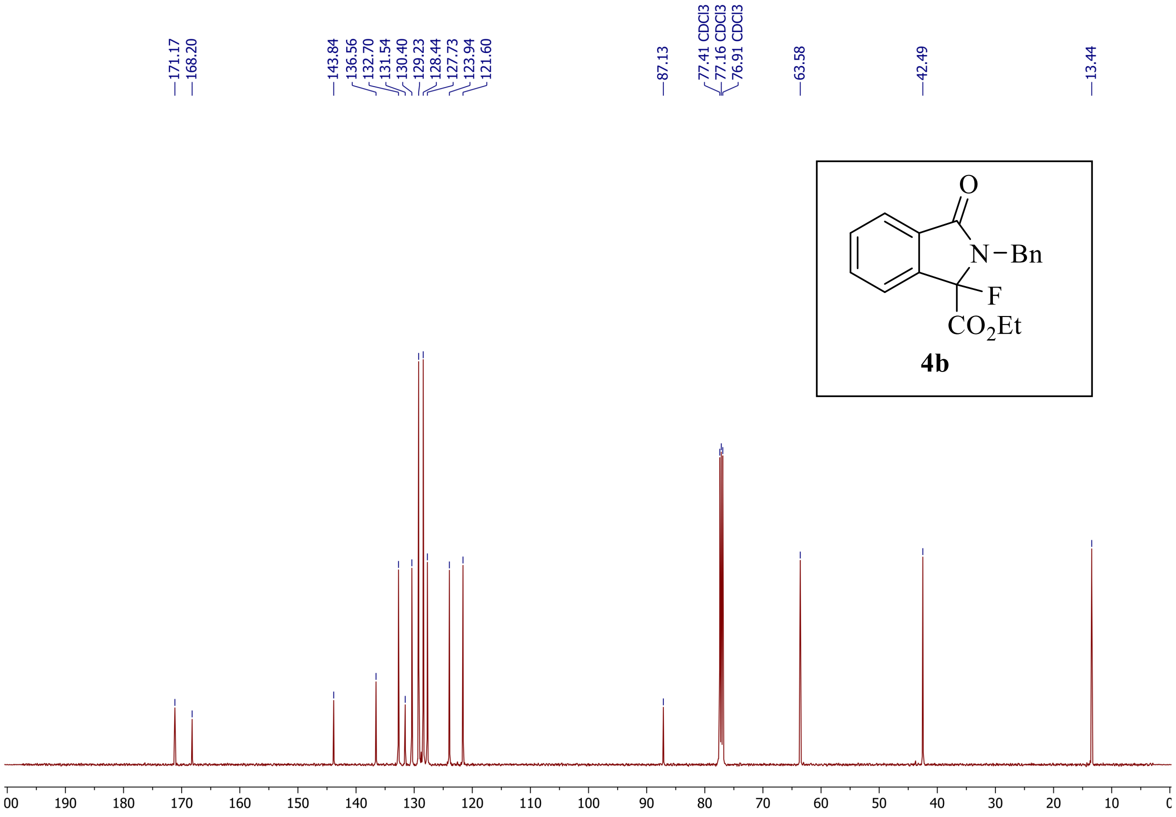

^{13}C-NMR (75 MHz, $CDCl_3$)

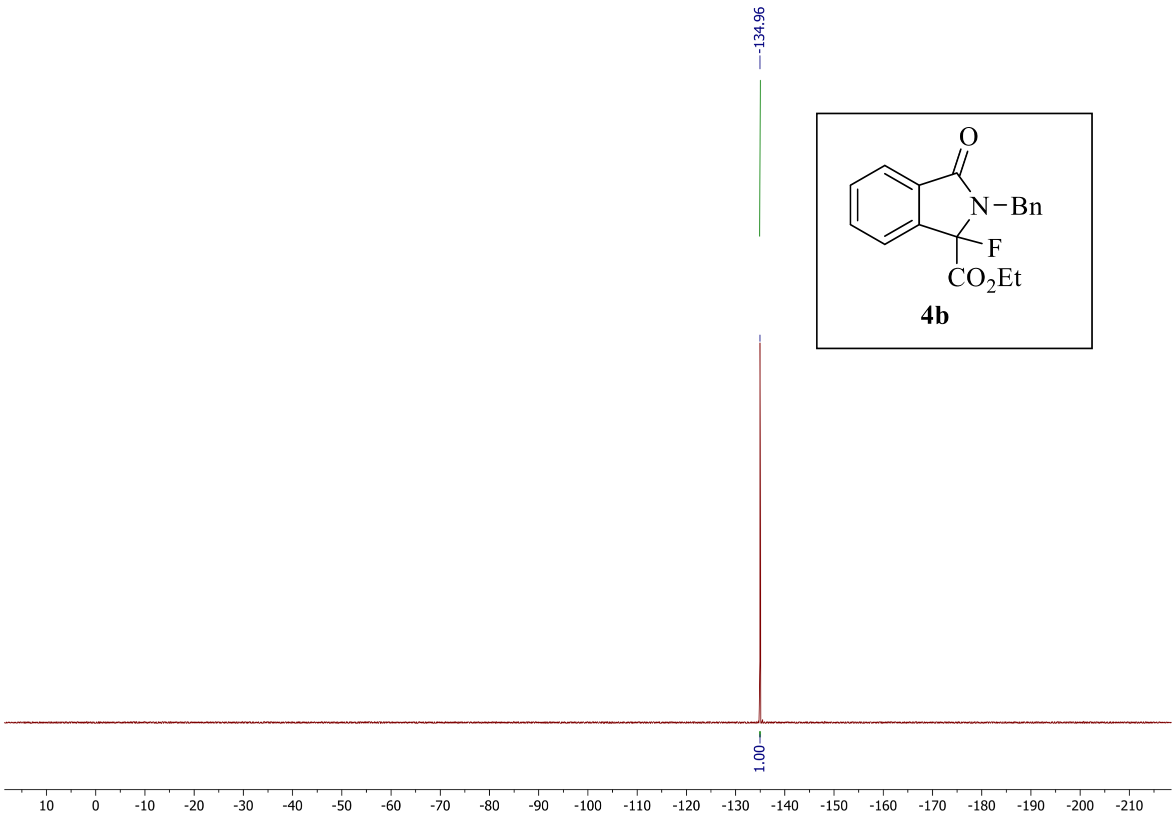

^{19}F-NMR (282 MHz, $CDCl_3$)

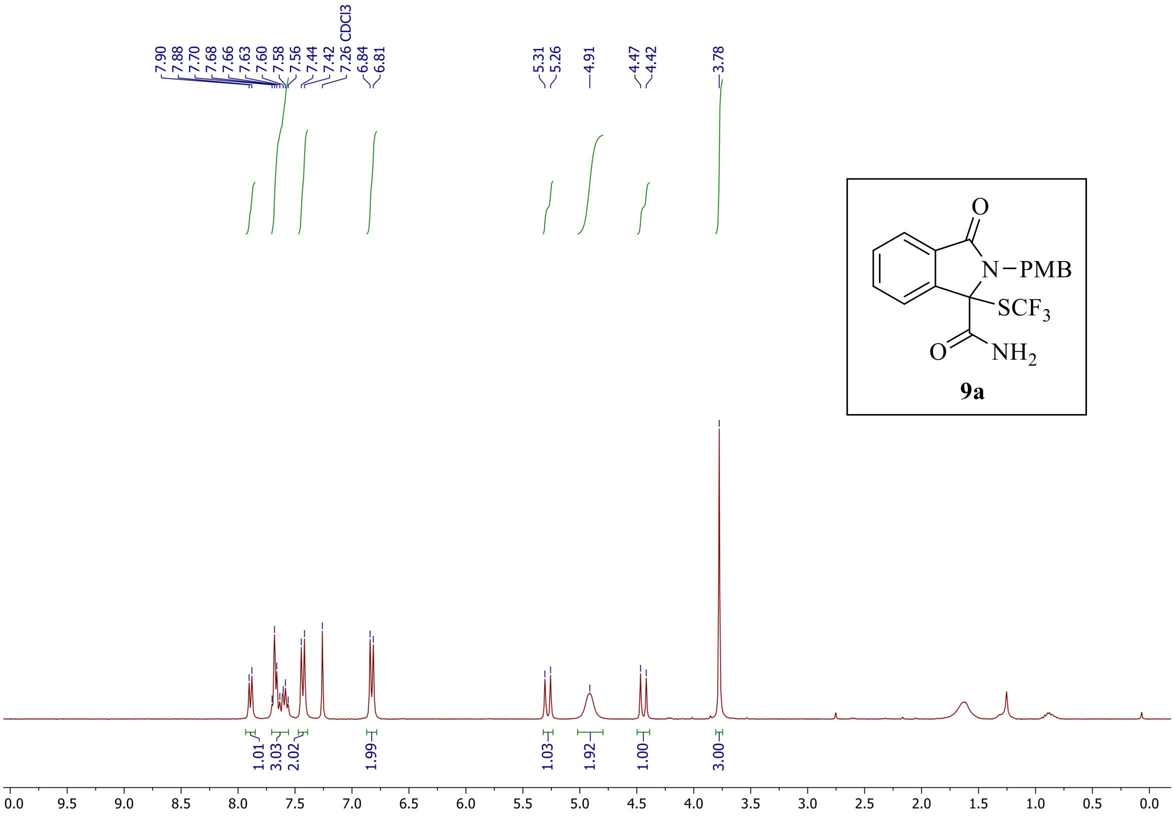

^{1}H-NMR (700 MHz, $CDCl_3$)

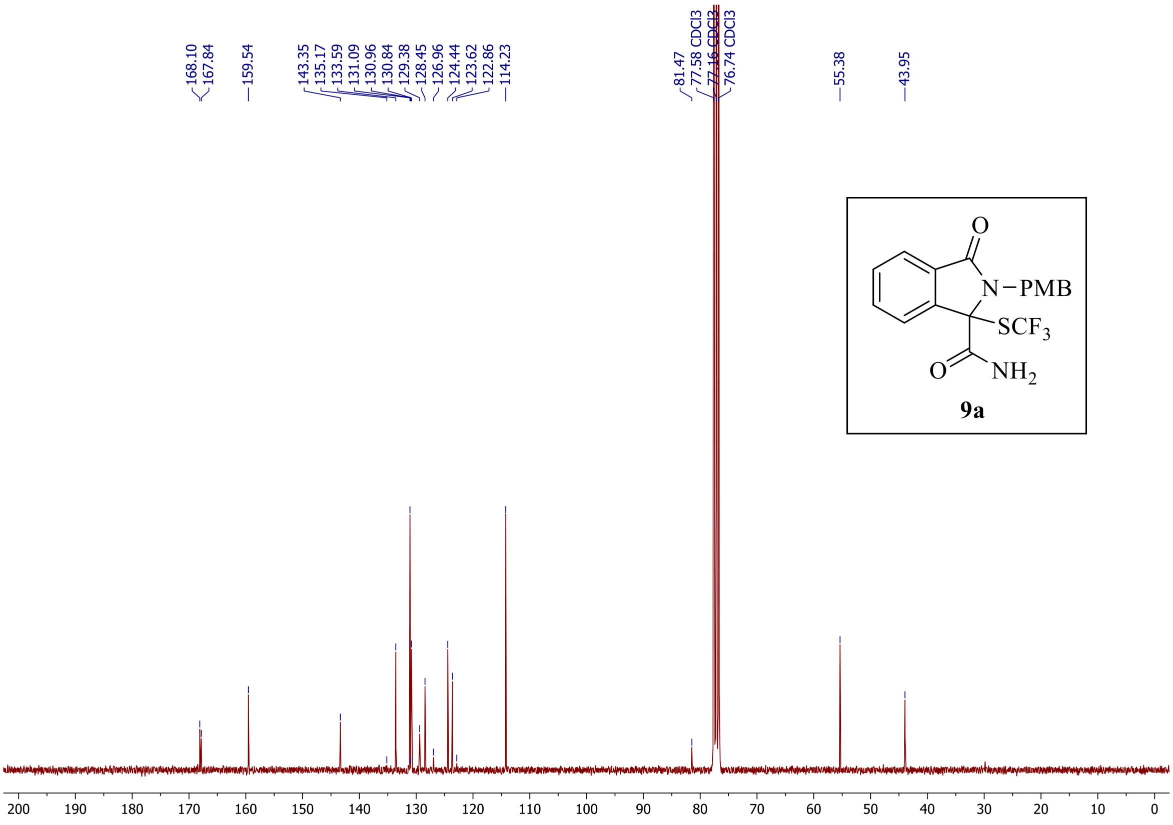

^{13}C-NMR (176 MHz, $CDCl_3$)

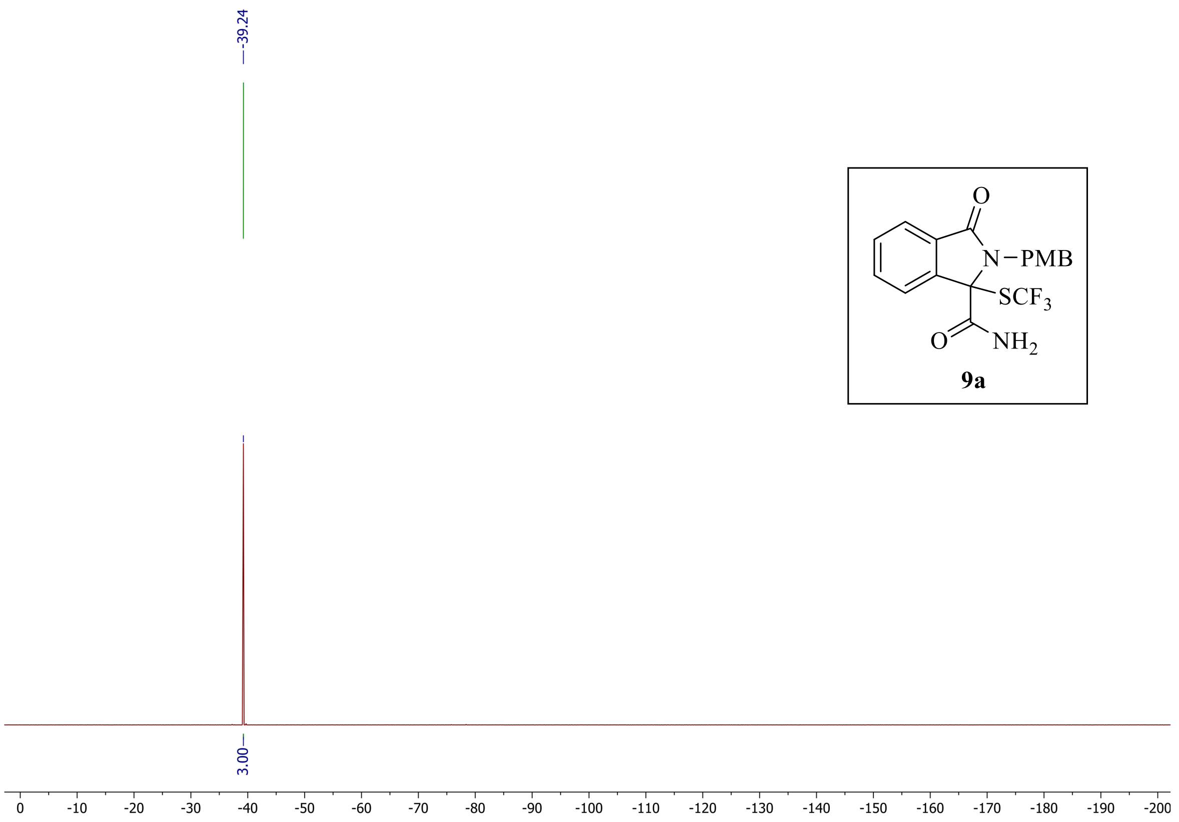

^{19}F-NMR (471 MHz, $CDCl_3$)

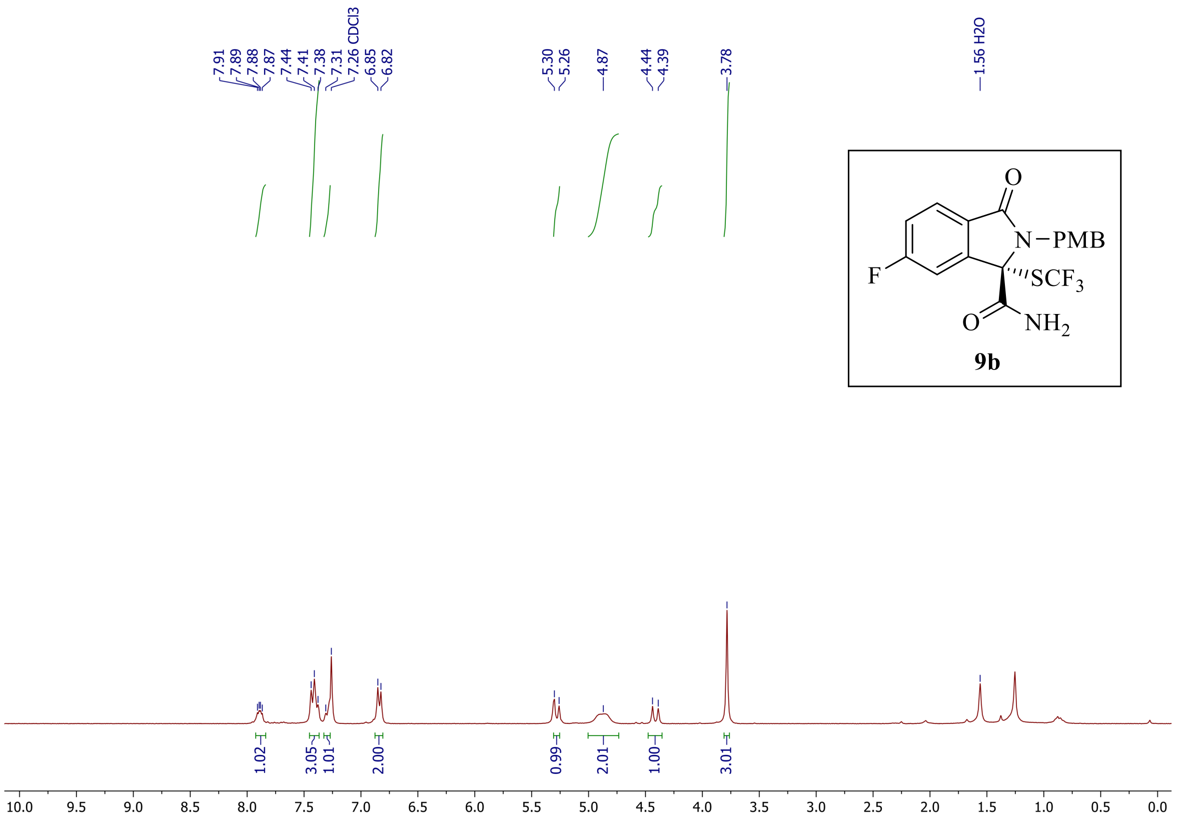

^{1}H-NMR (300 MHz, $CDCl_3$)

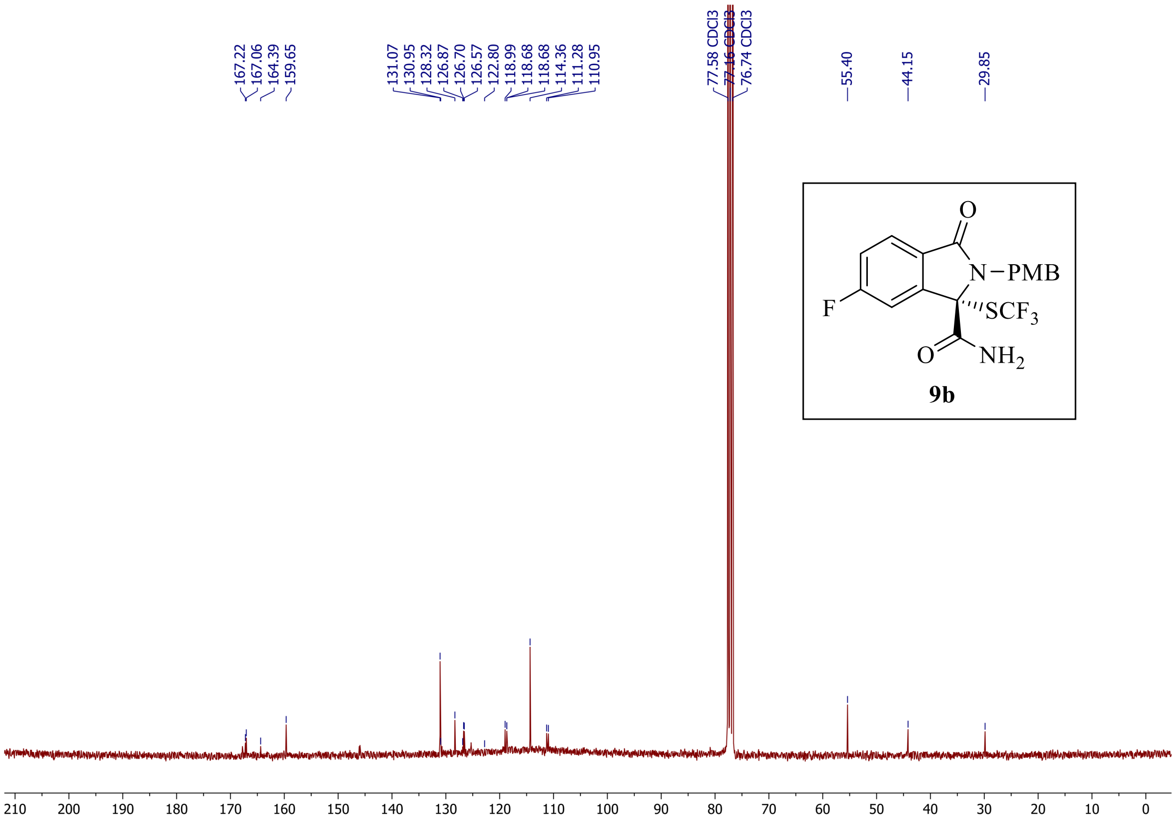

^{13}C-NMR (75 MHz, $CDCl_3$)

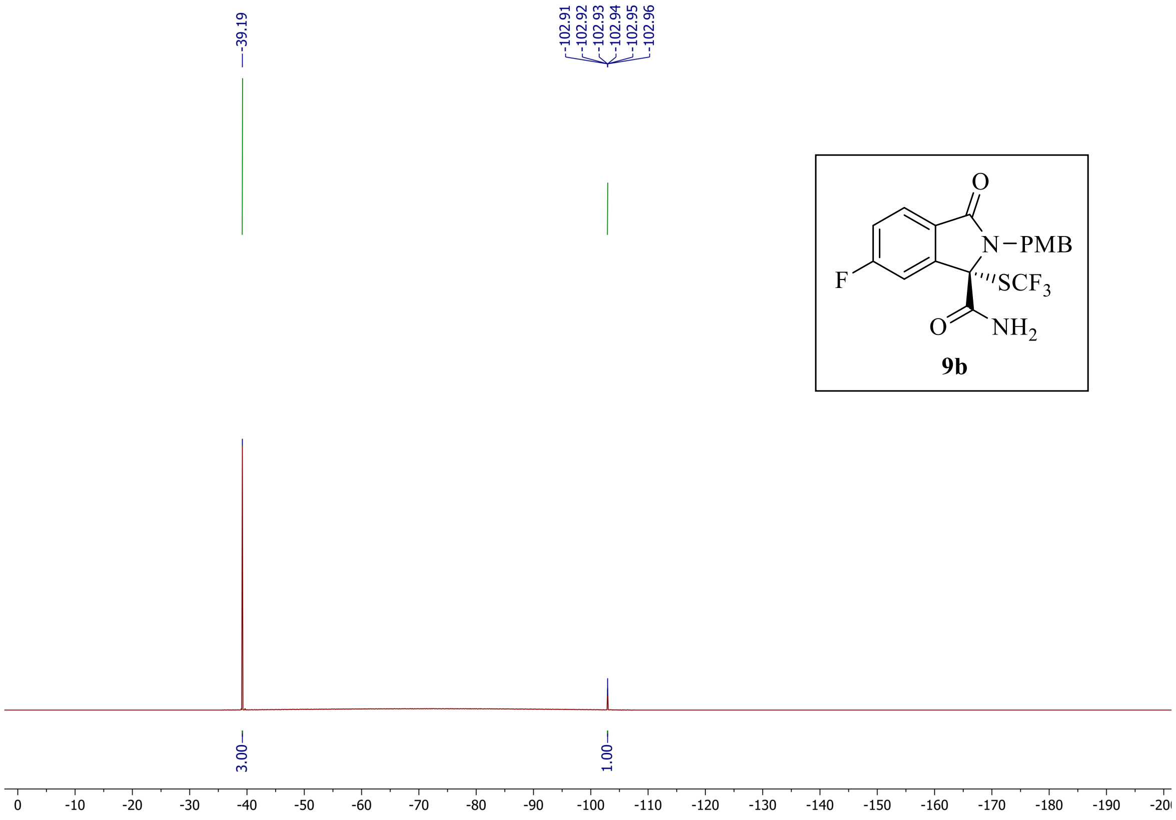

^{19}F-NMR (471 MHz, $CDCl_3$)

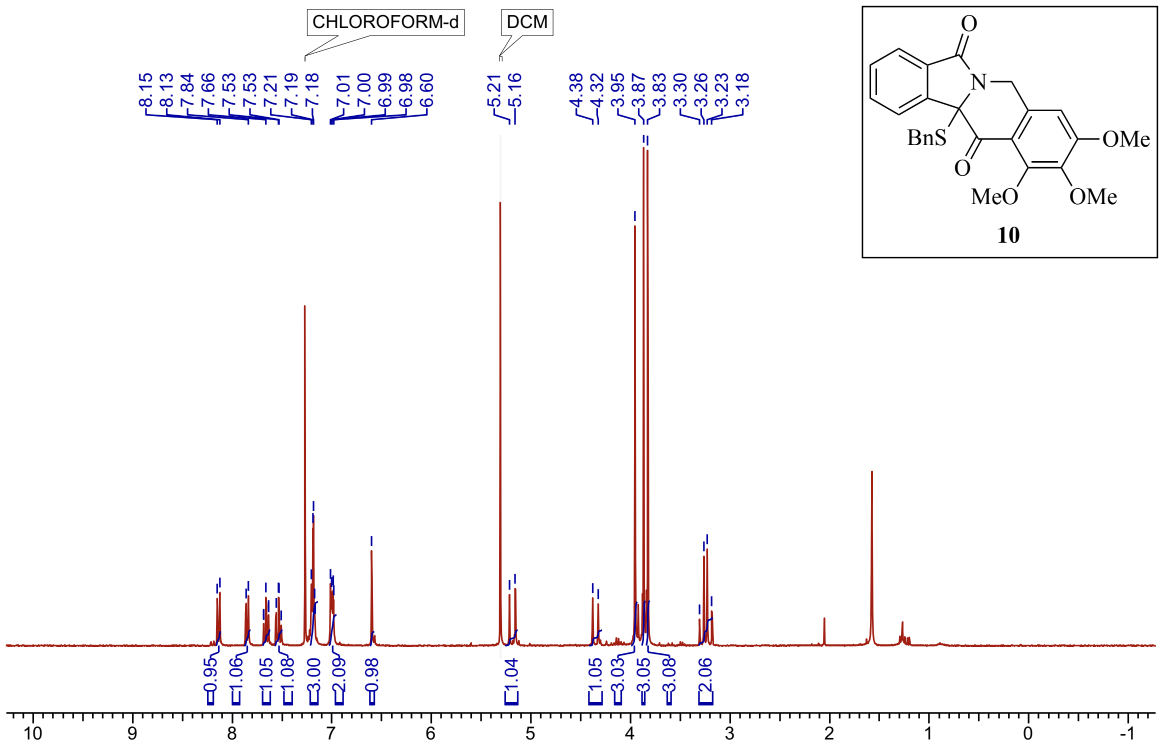

^{1}H-NMR (300 MHz, $CDCl_3$)

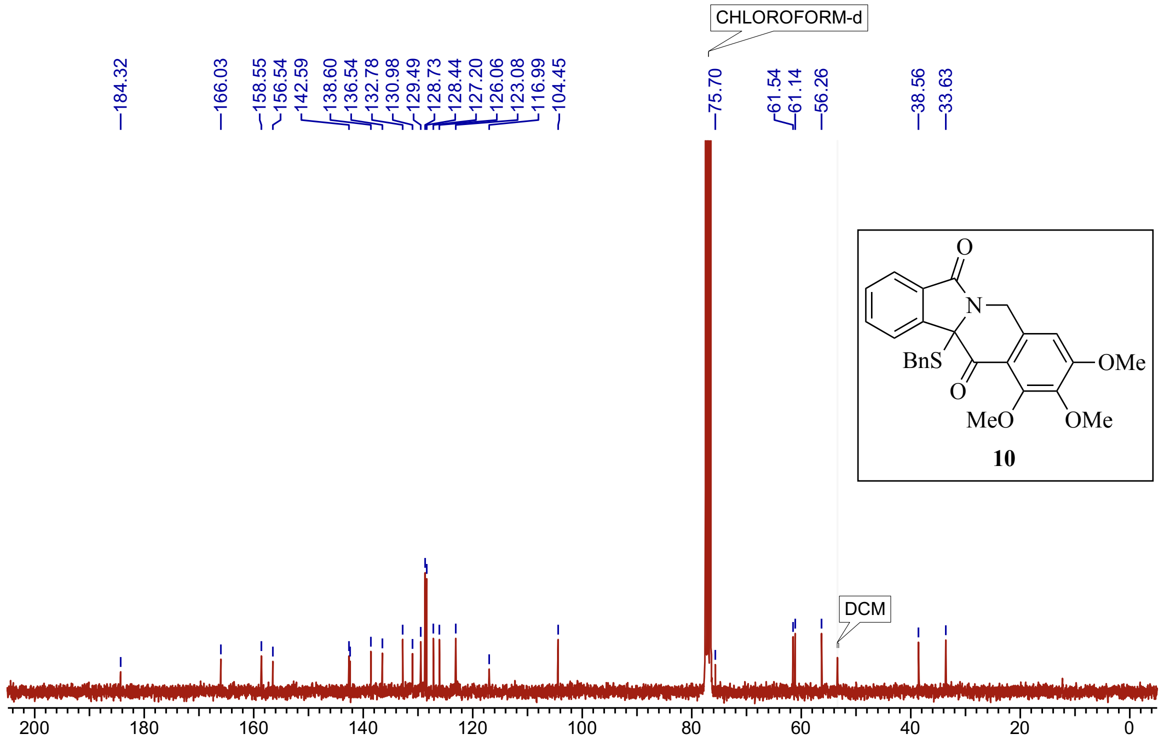

^{13}C-NMR (75 MHz, $CDCl_3$)

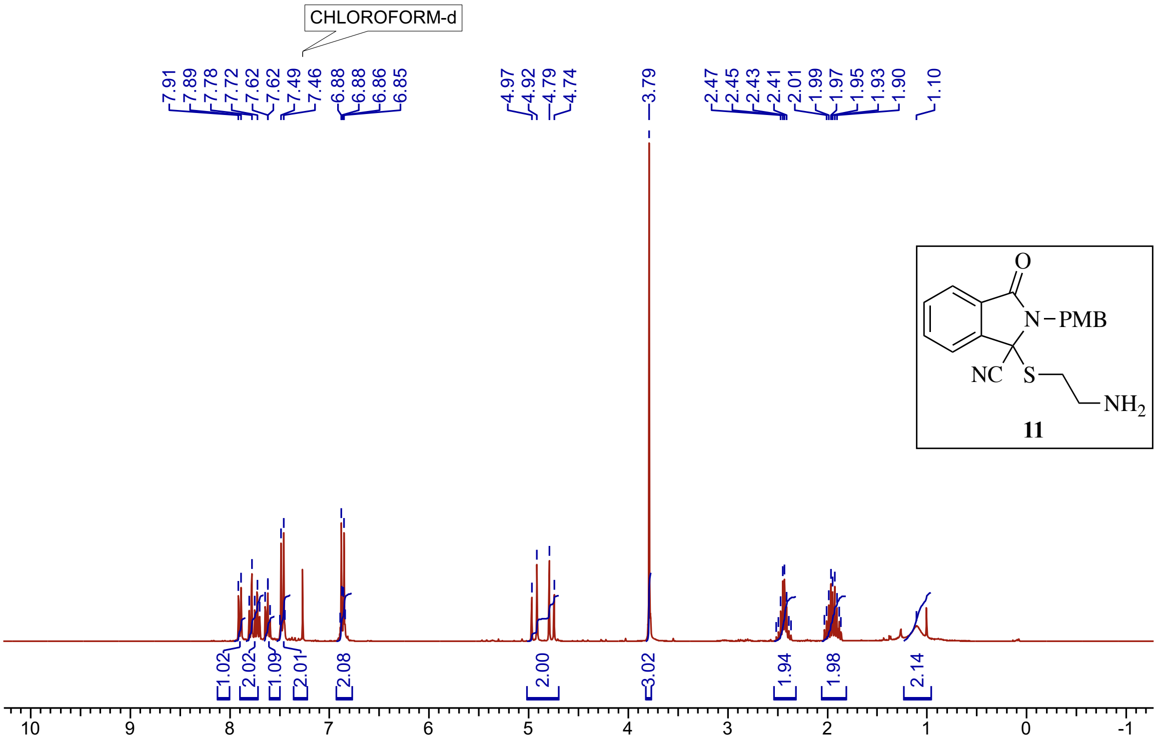

^{1}H-NMR (300 MHz, $CDCl_3$)

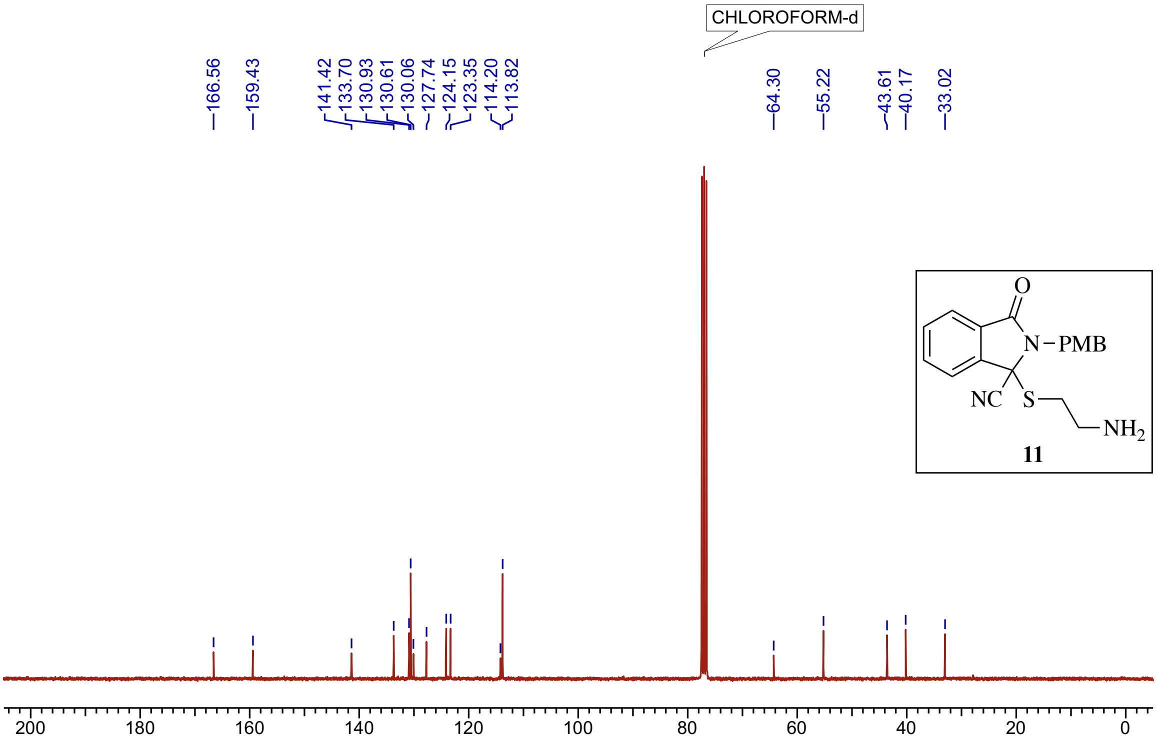

^{13}C-NMR (75 MHz, $CDCl_3$)

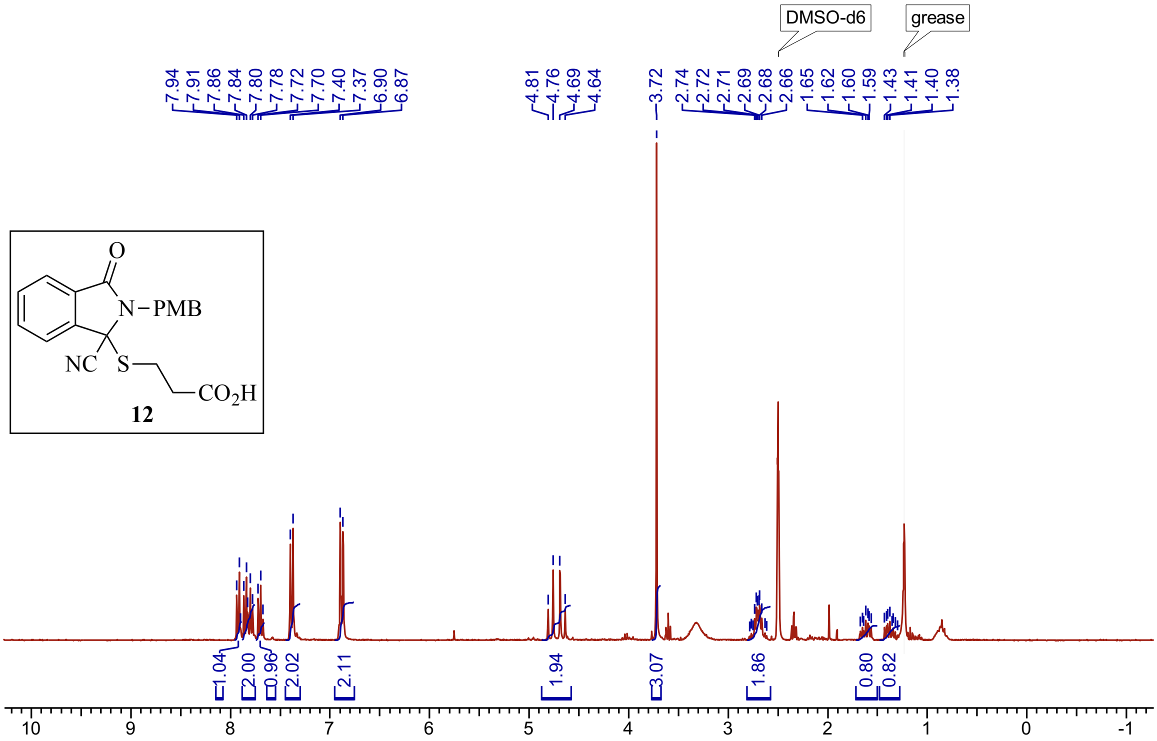

^{1}H-NMR (300 MHz, DMSO-d_6)

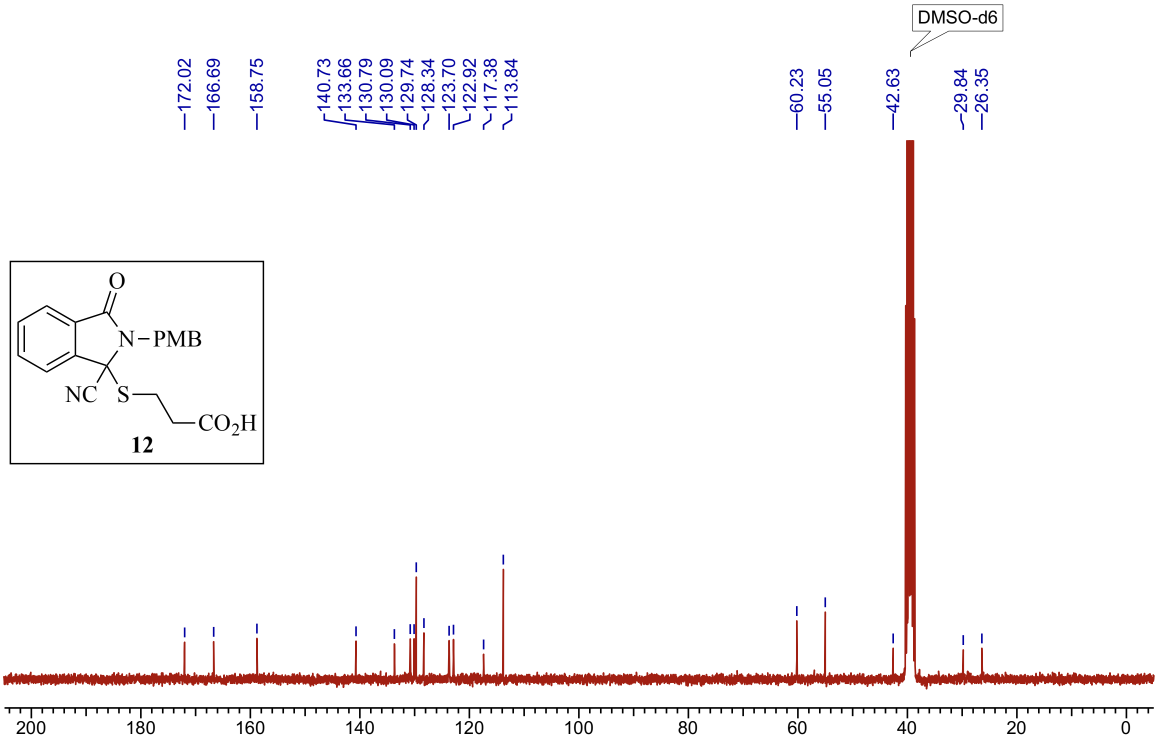

^{13}C-NMR (75 MHz, DMSO-d_6)

6. Copies of Catalyst & Catalyst-Precursor NMR Spectra

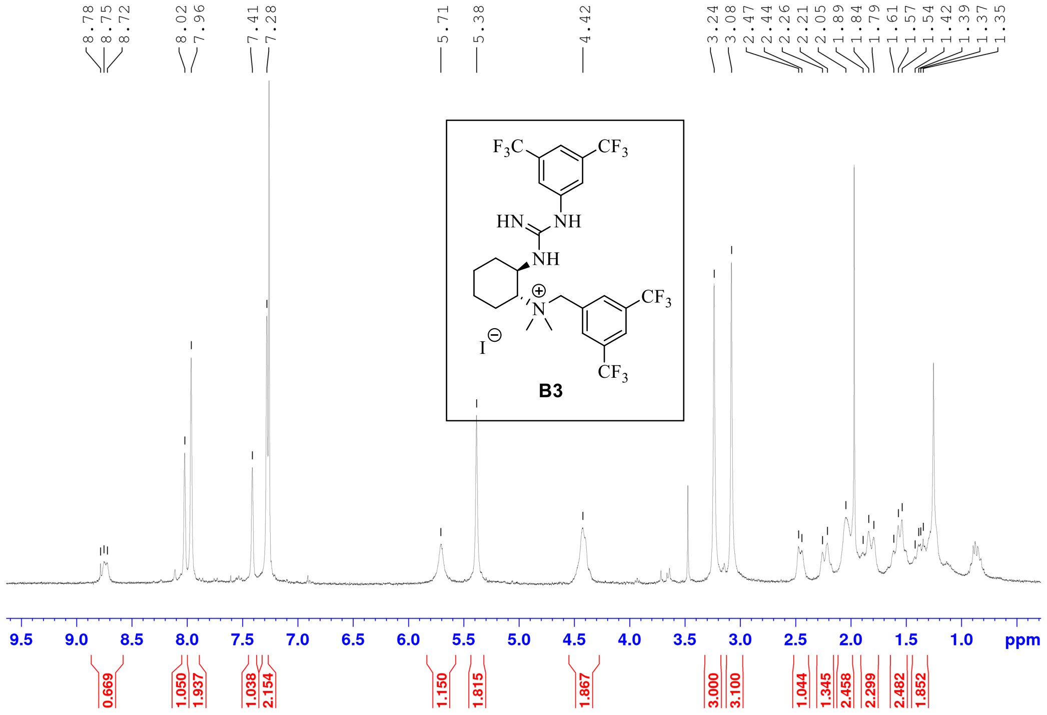

^{1}H-NMR (300 MHz, $CDCl_3$)

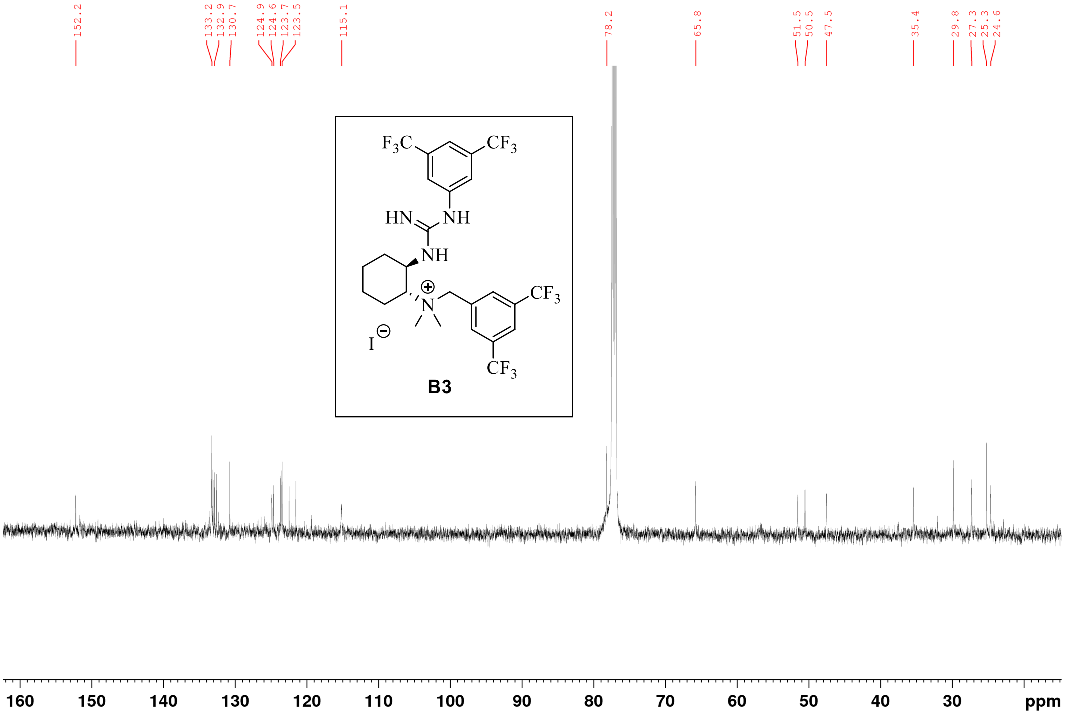

^{13}C-NMR (75 MHz, $CDCl_3$)

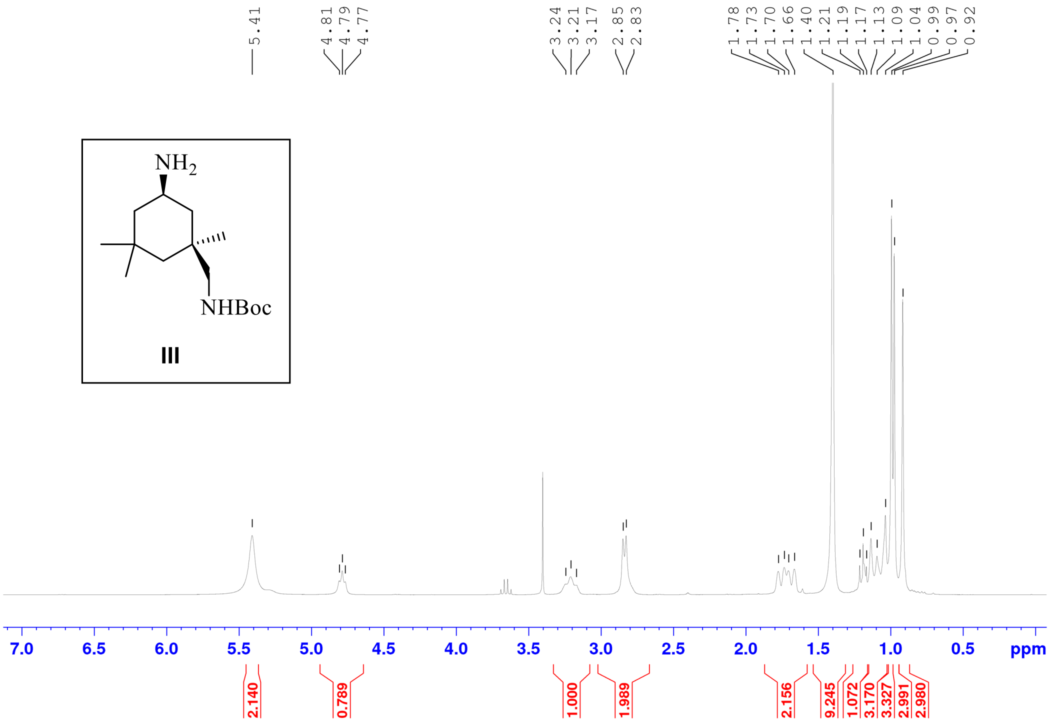

^{1}H-NMR (300 MHz, $CDCl_3$)

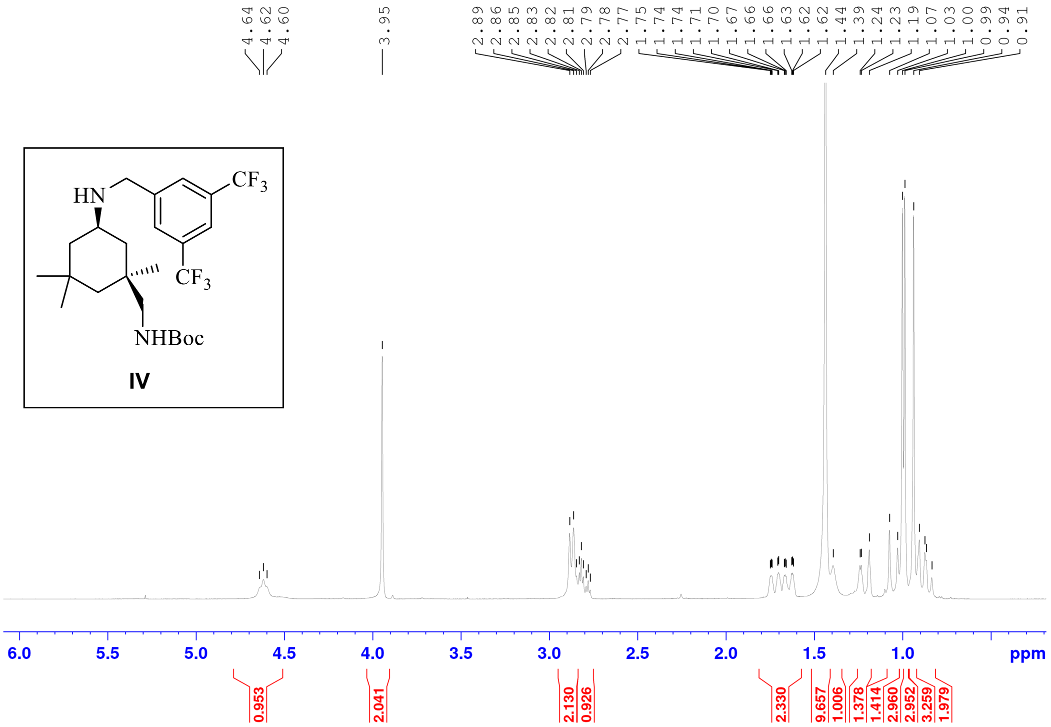

^{1}H-NMR (300 MHz, $CDCl_3$)

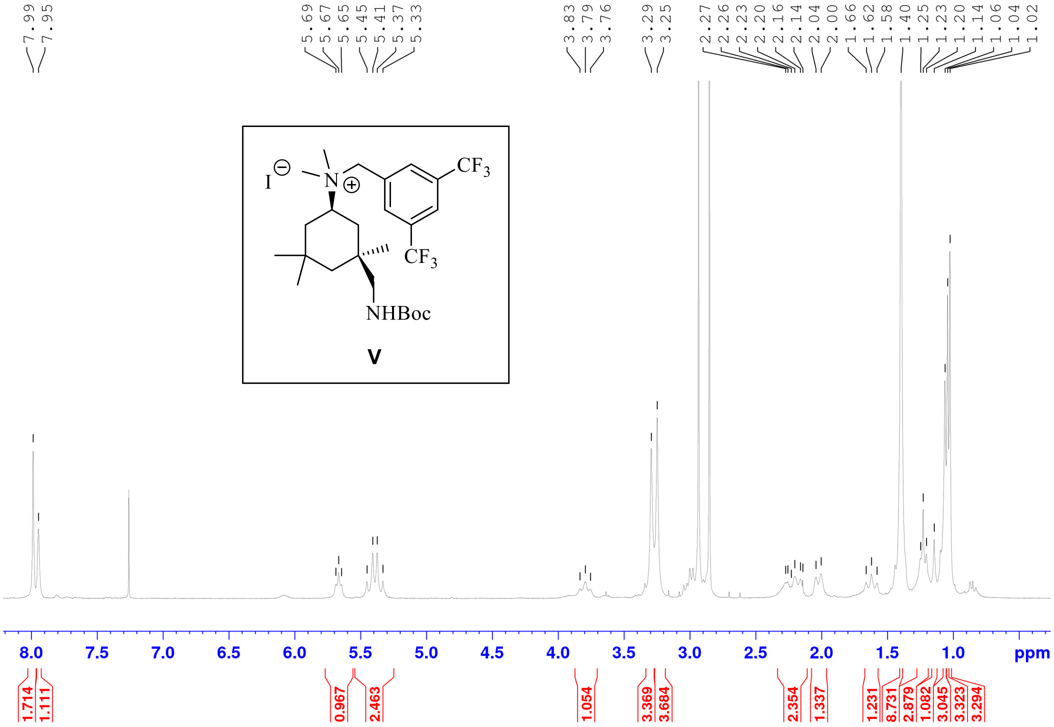

^{1}H-NMR (300 MHz, $CDCl_3$)

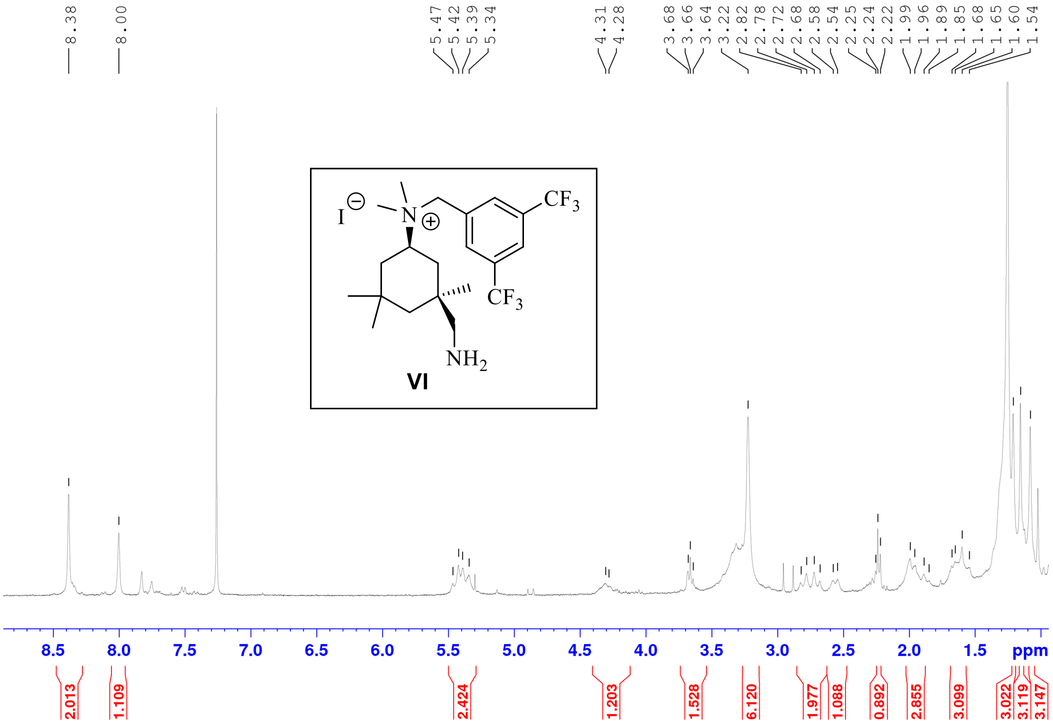

^{1}H-NMR (300 MHz, $CDCl_3$)

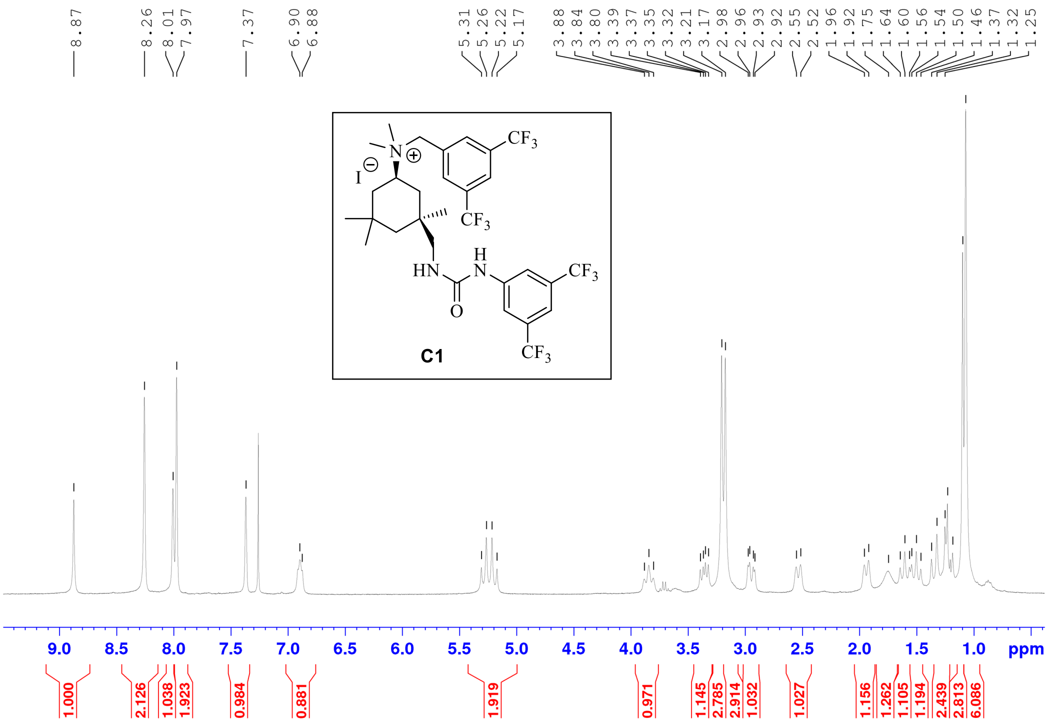

^{1}H-NMR (300 MHz, $CDCl_3$)

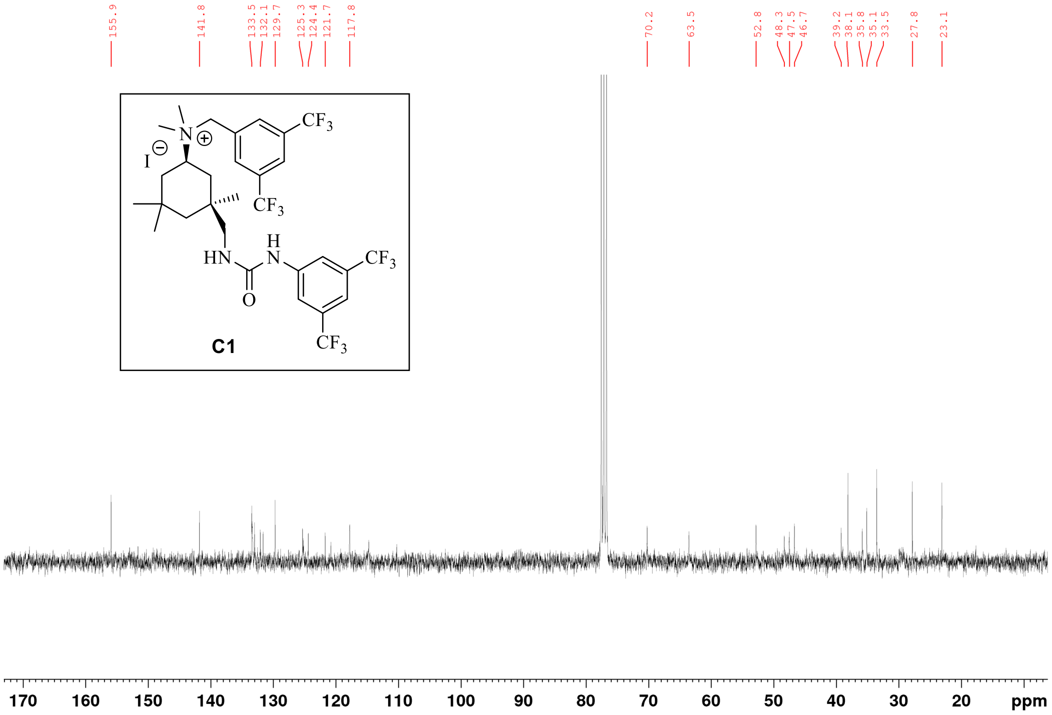

^{13}C-NMR (75 MHz, $CDCl_3$)

7. Copies of ^{19}F-NMR Spectra of Fluoro-Isoindolinone 4a with Kim's Chiral NMR Shift Reagent

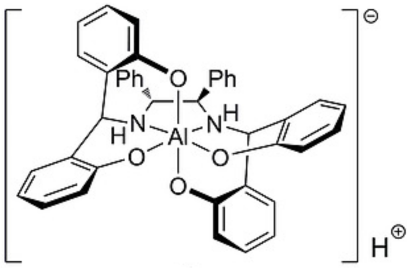

10 eq. in $CDCl_3$

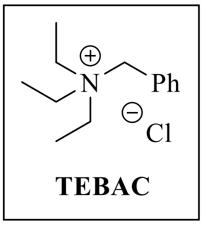

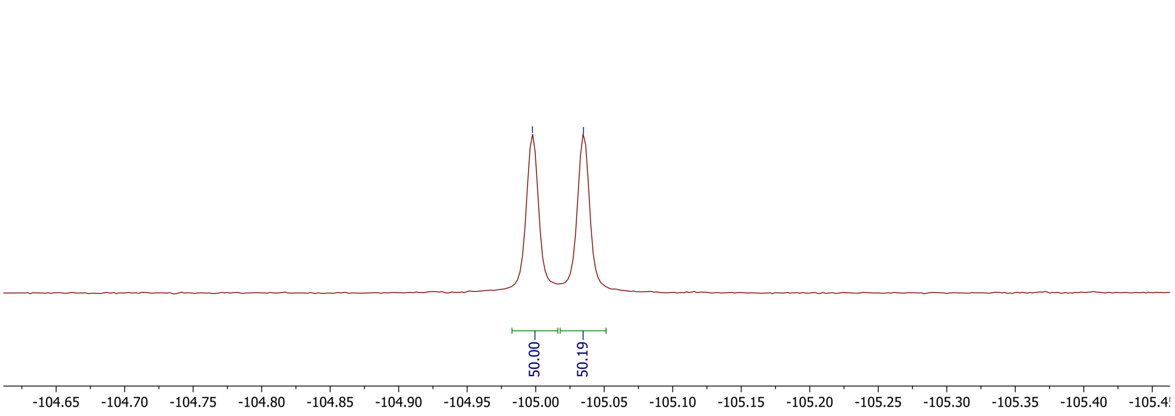

^{19}F-NMR (471 MHz, $CDCl_3$)

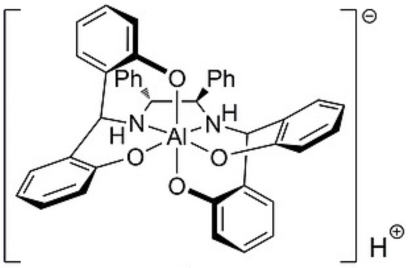

10 eq. in $CDCl_3$

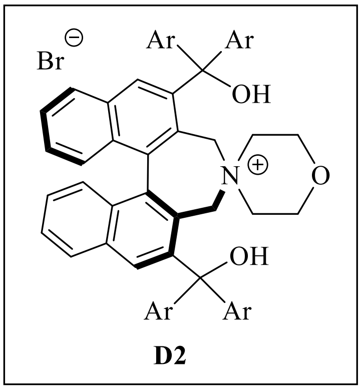

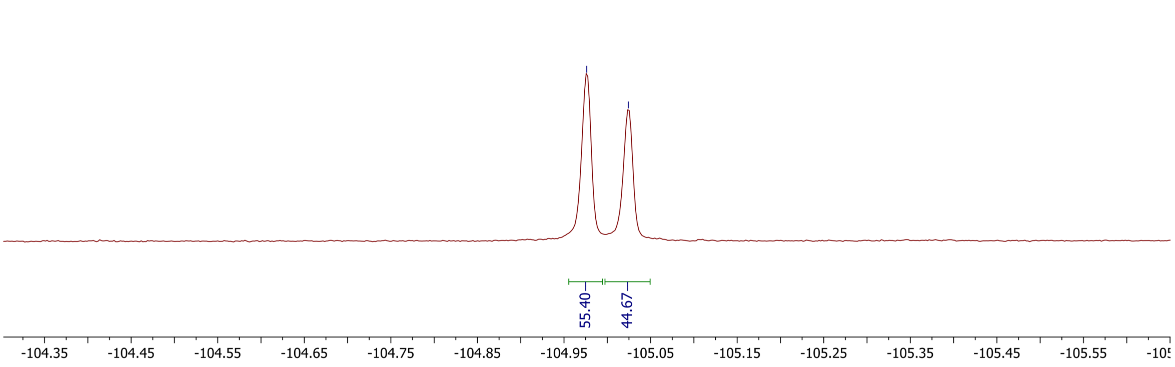

^{19}F-NMR (471 MHz, $CDCl_3$)

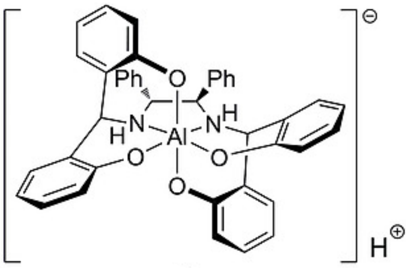

10 eq. in $CDCl_3$

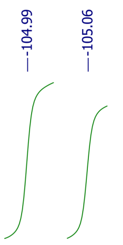

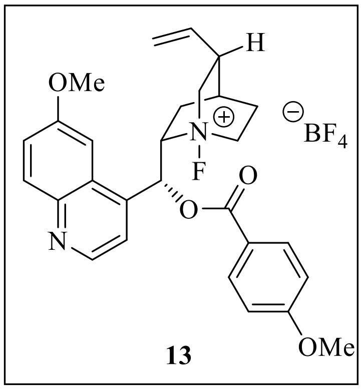

13

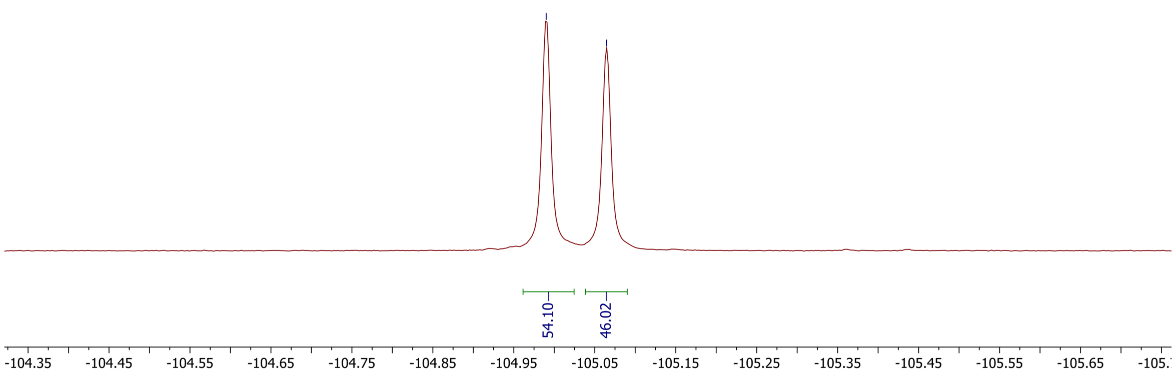

^{19}F-NMR (471 MHz, $CDCl_3$)

8. Copies of HPLC Chromatograms

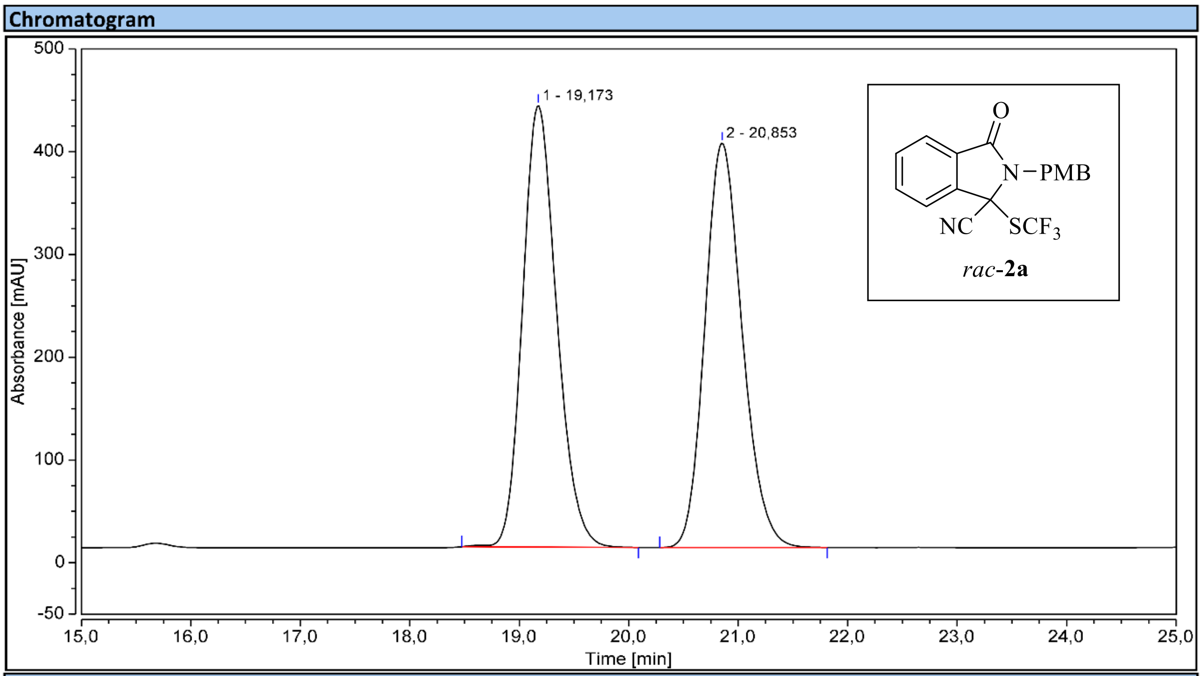

Integration Results

No.	Peak Name	Retention Time min	Area mAU*min	Height mAU	Relative Area %	Relative Height %	Amount n.a.
1		19,173	155,280	429,662	49,97	52,19	n.a.
2		20,853	155,448	393,570	50,03	47,81	n.a.
**Total:**			**310,728**	**823,232**	**100,00**	**100,00**	

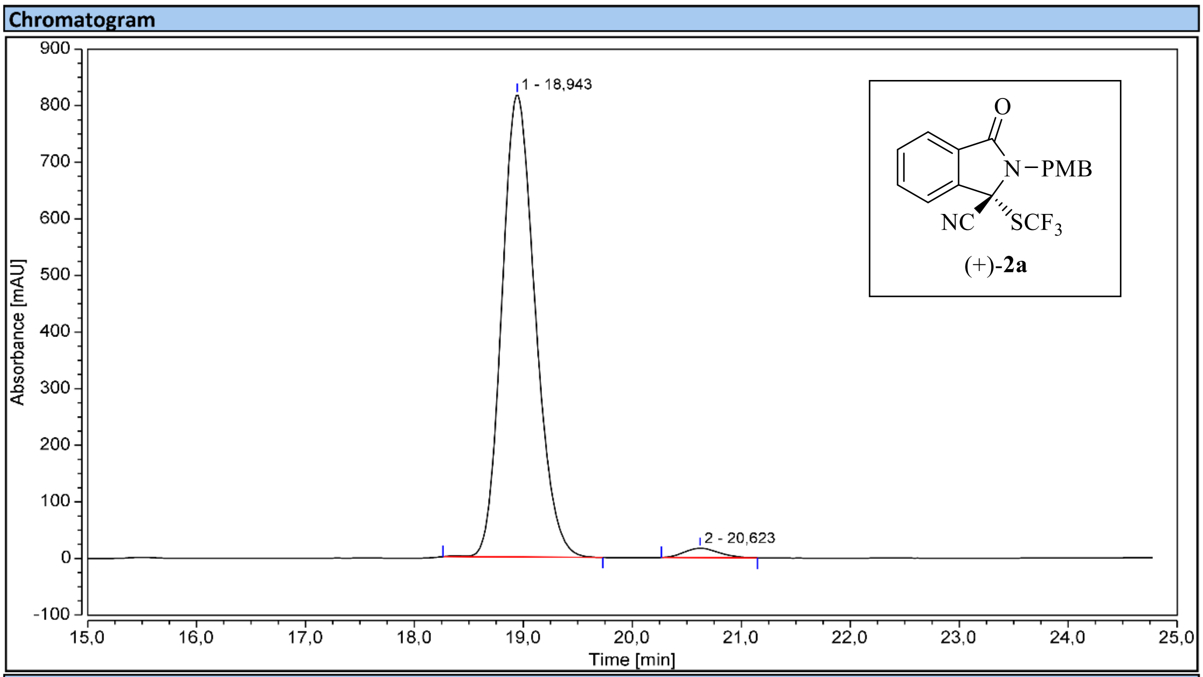

Integration Results

No.	Peak Name	Retention Time min	Area mAU*min	Height mAU	Relative Area %	Relative Height %	Amount n.a.
1		18,943	289,149	817,029	97,91	98,00	n.a.
2		20,623	6,171	16,713	2,09	2,00	n.a.
**Total:**			**295,320**	**833,742**	**100,00**	**100,00**	

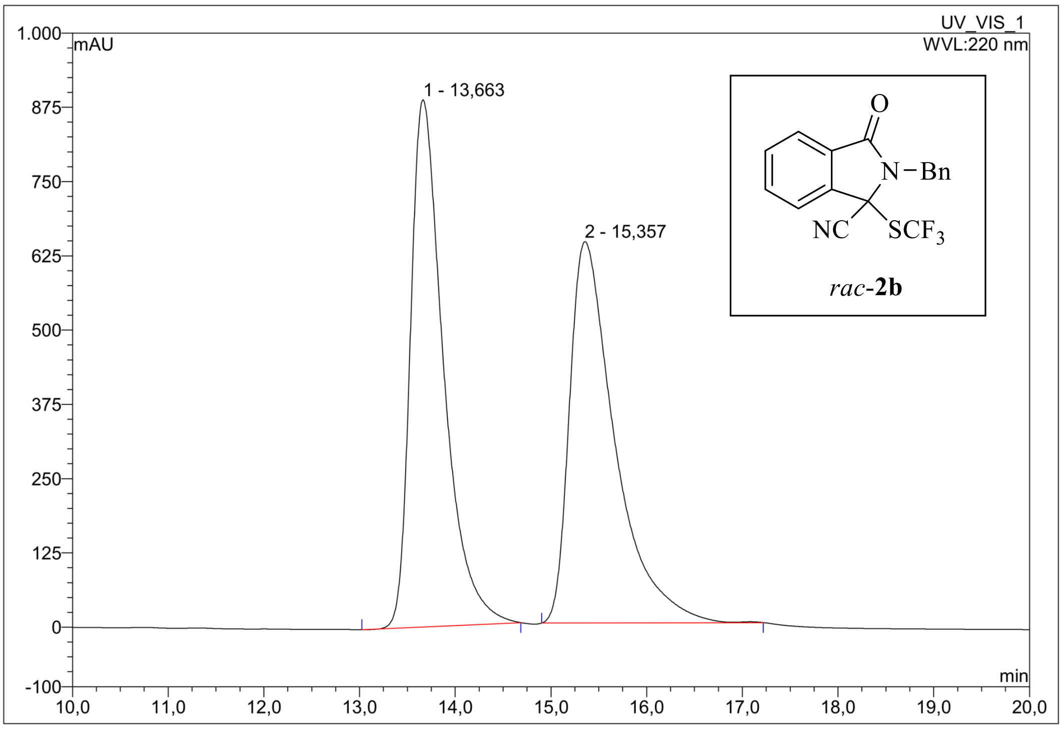

No.	Ret.Time min	Peak Name	Height mAU	Area mAU*min	Rel.Area %	Amount	Type
1	13,66	n.a.	887,188	355,060	50,19	n.a.	BMB*
2	15,36	n.a.	641,753	352,385	49,81	n.a.	BMB*
**Total:**			1528,940	707,446	100,00	0,000	

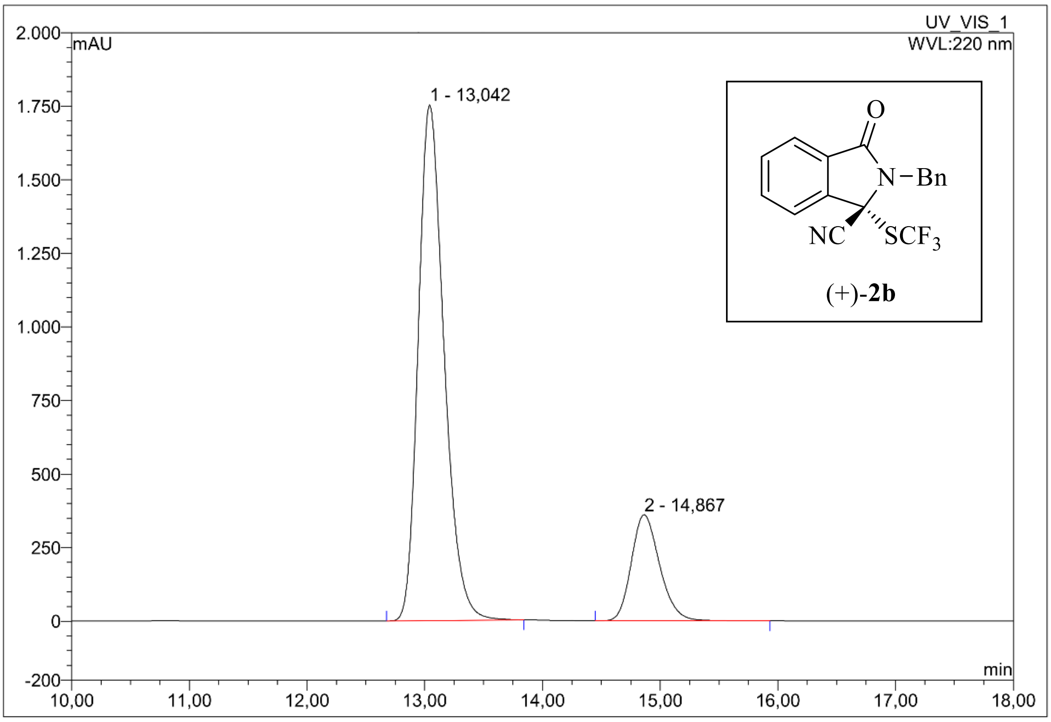

No.	Ret.Time min	Peak Name	Height mAU	Area mAU*min	Rel.Area %	Amount	Type
1	13,04	n.a.	1752,661	445,587	81,61	n.a.	BMB
2	14,87	n.a.	360,057	100,419	18,39	n.a.	BMB
**Total:**			2112,718	546,005	100,00	0,000	

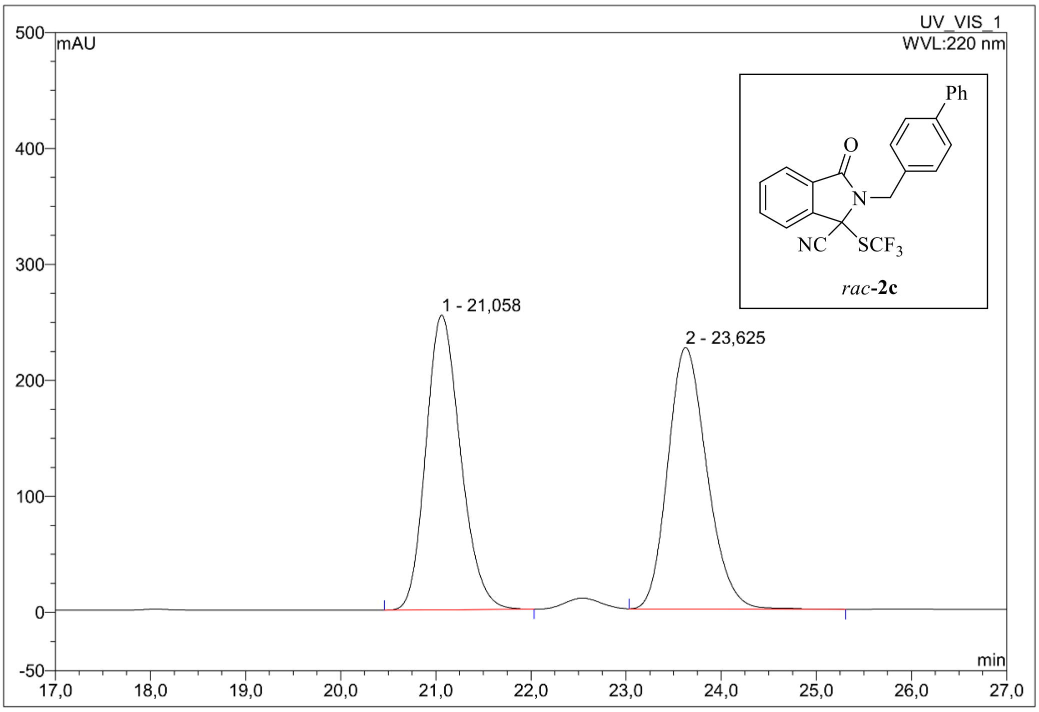

No.	Ret.Time min	Peak Name	Height mAU	Area mAU*min	Rel.Area %	Amount	Type
1	21,06	n.a.	253,918	107,210	49,95	n.a.	BMB*
2	23,63	n.a.	225,381	107,412	50,05	n.a.	BMB*
Total:			479,300	214,623	100,00	0,000	

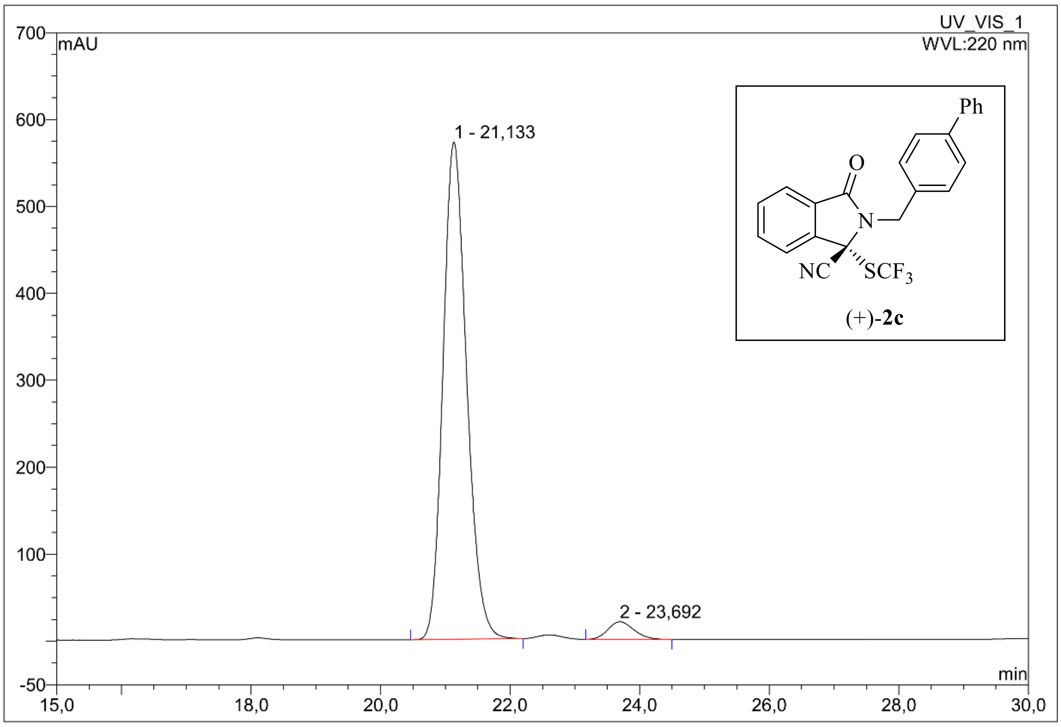

No.	Ret.Time min	Peak Name	Height mAU	Area mAU*min	Rel.Area %	Amount	Type
1	21,13	n.a.	572,160	241,306	96,22	n.a.	BMB*
2	23,69	n.a.	20,278	9,474	3,78	n.a.	BMB*
Total:			592,438	250,780	100,00	0,000	

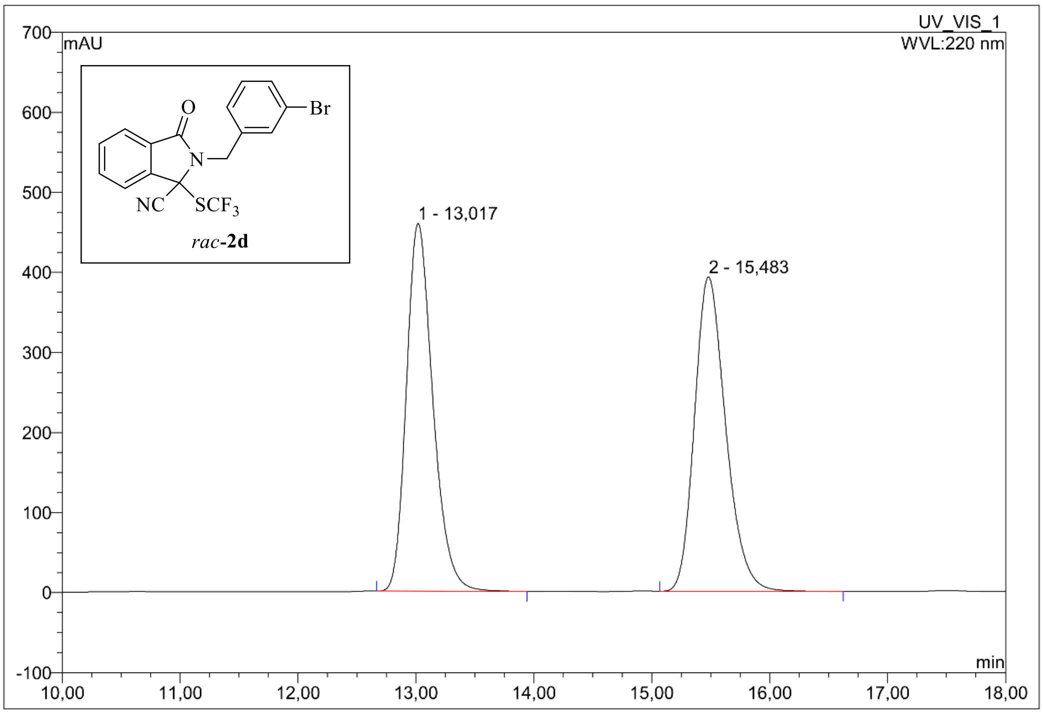

No.	Ret.Time min	Peak Name	Height mAU	Area mAU*min	Rel.Area %	Amount	Type
1	13,02	n.a.	459,602	119,678	49,83	n.a.	BMB
2	15,48	n.a.	392,863	120,497	50,17	n.a.	BMB
**Total:**			852,465	240,176	100,00	0,000	

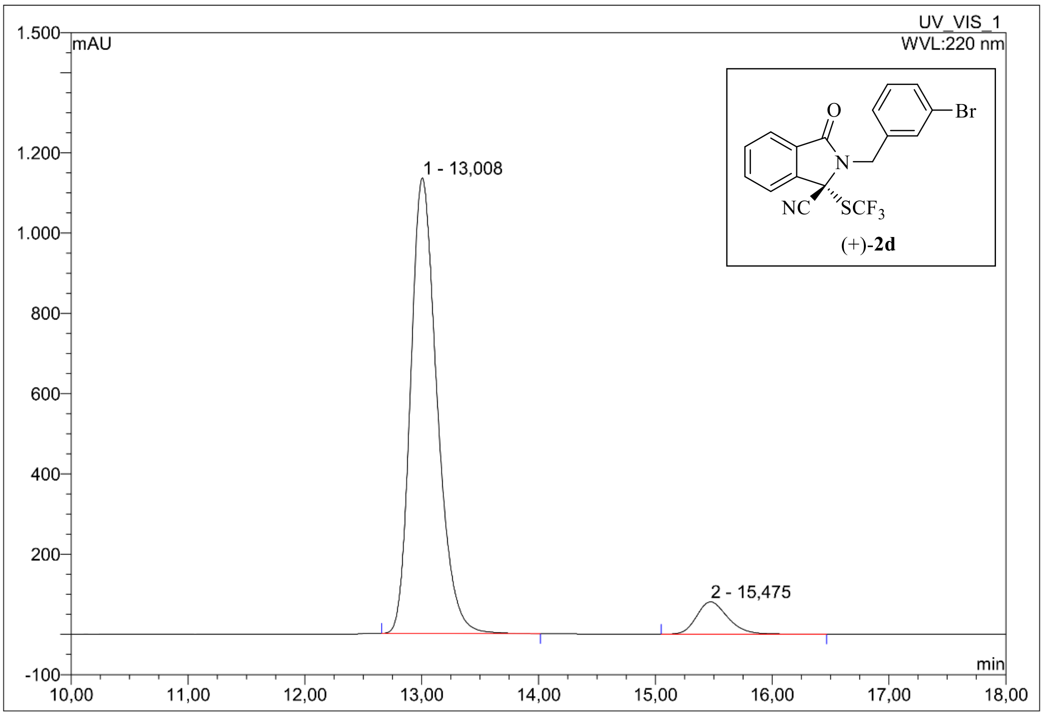

No.	Ret.Time min	Peak Name	Height mAU	Area mAU*min	Rel.Area %	Amount	Type
1	13,01	n.a.	1134,649	297,201	92,35	n.a.	BMB
2	15,48	n.a.	80,332	24,621	7,65	n.a.	BMB
**Total:**			1214,981	321,822	100,00	0,000	

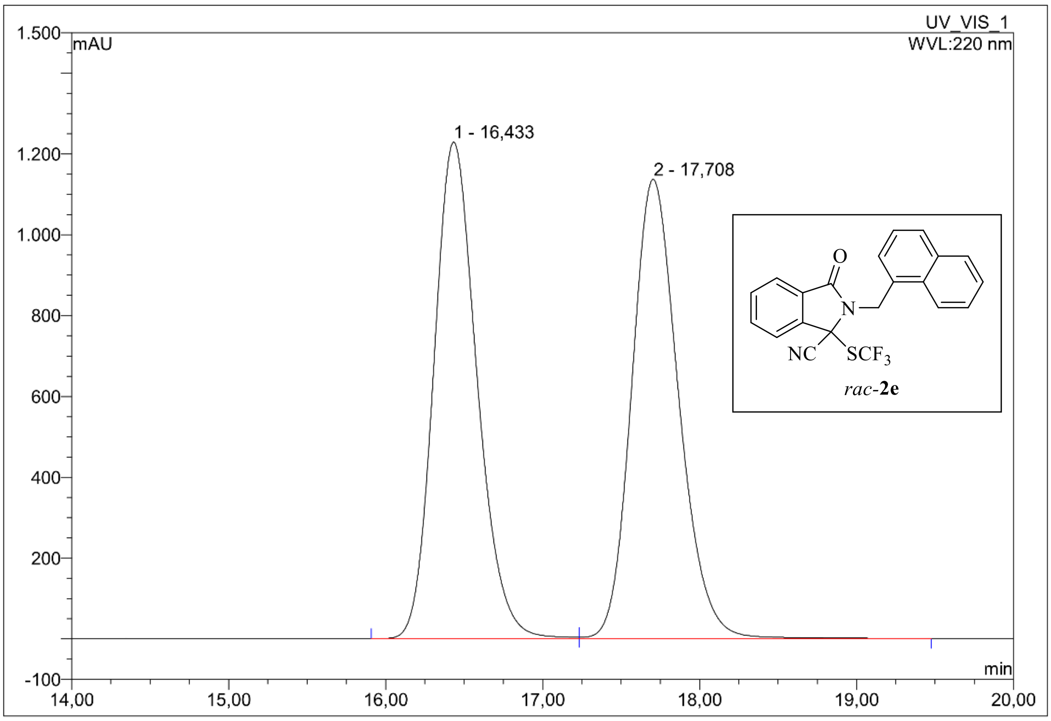

No.	Ret.Time min	Peak Name	Height mAU	Area mAU*min	Rel.Area %	Amount	Type
1	16,43	n.a.	1228,495	391,047	49,90	n.a.	BM
2	17,71	n.a.	1135,725	392,672	50,10	n.a.	MB
Total:			2364,220	783,719	100,00	0,000	

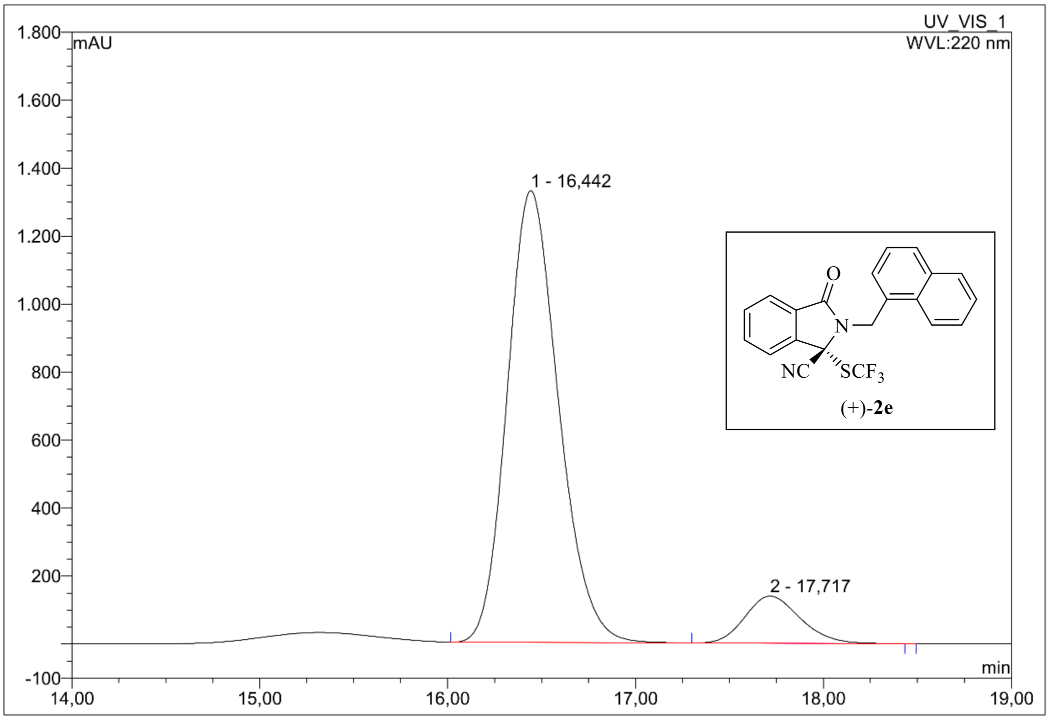

No.	Ret.Time min	Peak Name	Height mAU	Area mAU*min	Rel.Area %	Amount	Type
1	16,44	n.a.	1328,574	420,315	89,93	n.a.	BMB*
2	17,72	n.a.	138,250	47,042	10,07	n.a.	Rd
Total:			1466,824	467,358	100,00	0,000	

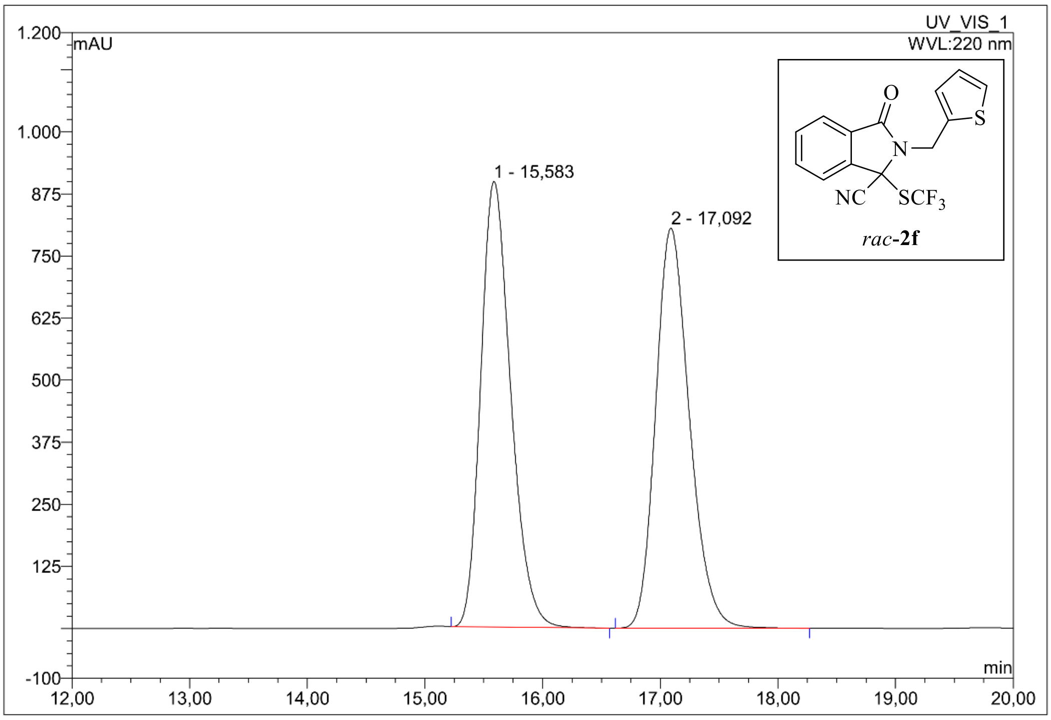

No.	Ret.Time min	Peak Name	Height mAU	Area mAU*min	Rel.Area %	Amount	Type
1	15,58	n.a.	897,681	265,845	49,78	n.a.	BMB
2	17,09	n.a.	805,570	268,213	50,22	n.a.	BMB
Total:			1703,251	534,058	100,00	0,000	

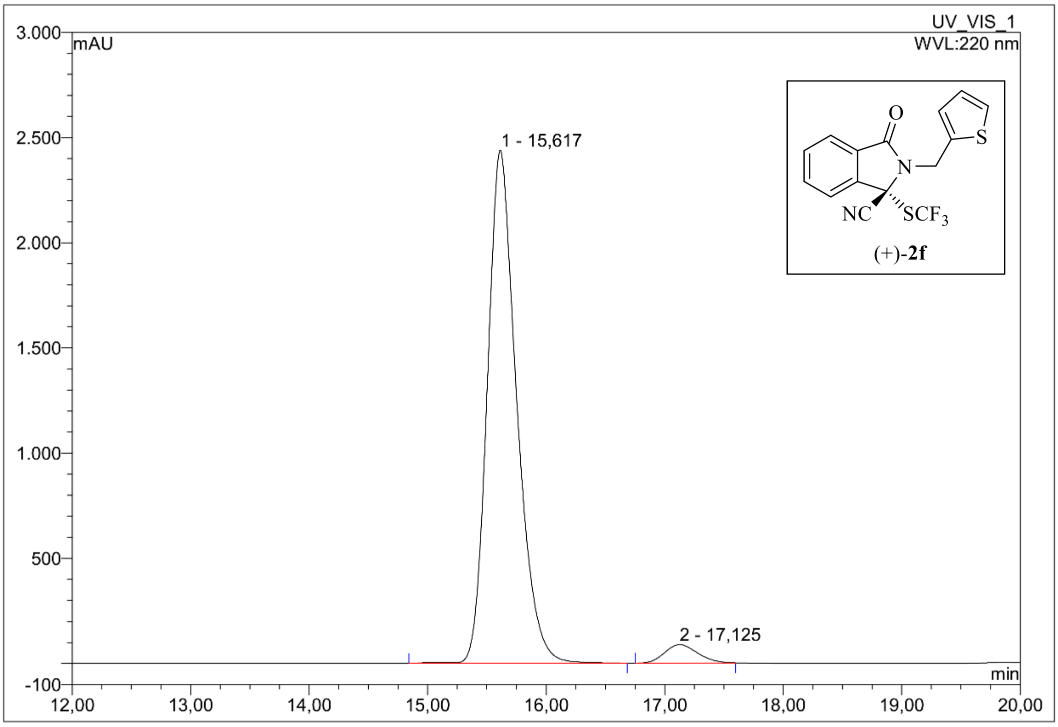

No.	Ret.Time min	Peak Name	Height mAU	Area mAU*min	Rel.Area %	Amount	Type
1	15,62	n.a.	2437,411	697,738	96,12	n.a.	BMB*
2	17,13	n.a.	87,452	28,189	3,88	n.a.	BMB*
Total:			2524,863	725,927	100,00	0,000	

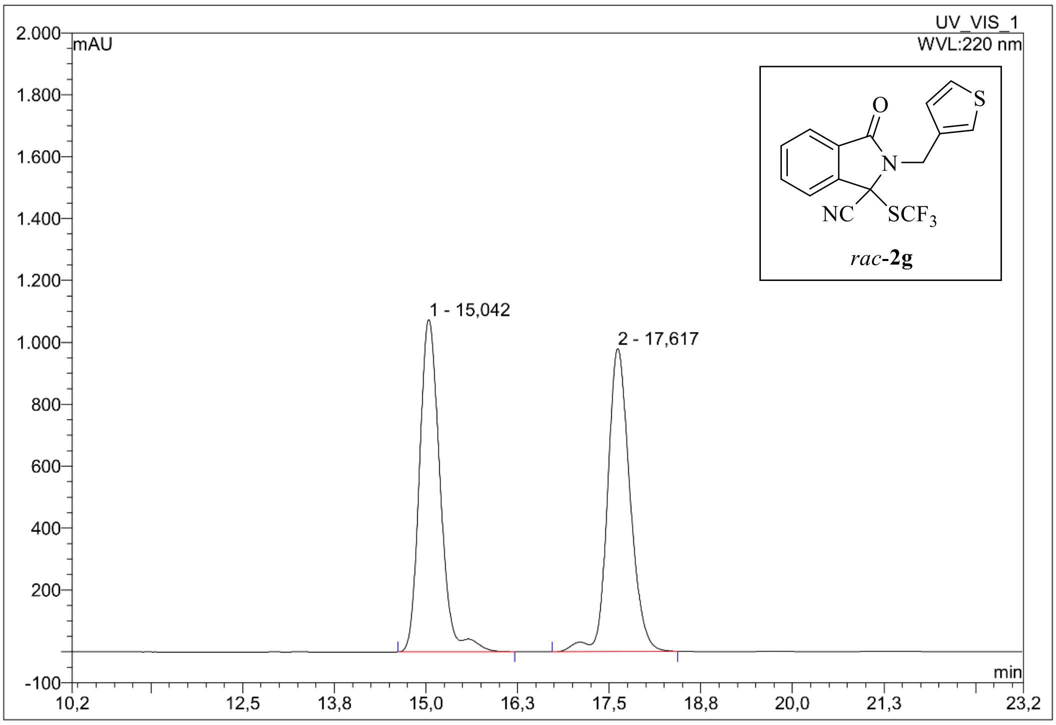

No.	Ret.Time min	Peak Name	Height mAU	Area mAU*min	Rel.Area %	Amount	Type
1	15,04	n.a.	1073,312	350,346	50,21	n.a.	BMB*
2	17,62	n.a.	978,443	347,471	49,79	n.a.	BMB*
Total:			2051,756	697,817	100,00	0,000	

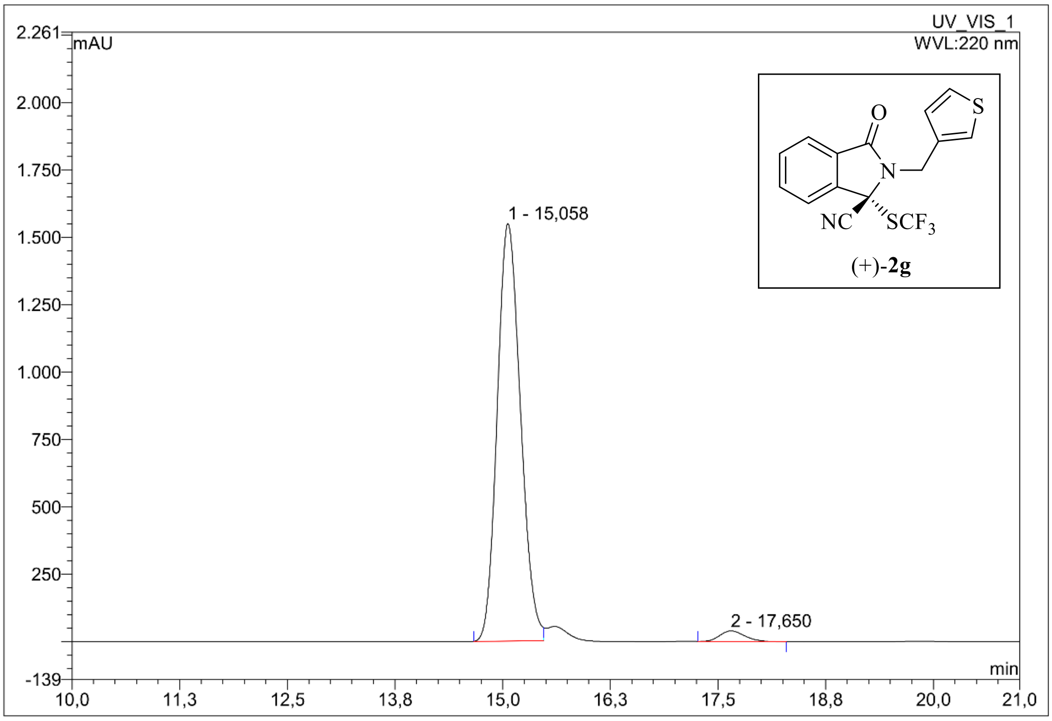

No.	Ret.Time min	Peak Name	Height mAU	Area mAU*min	Rel.Area %	Amount	Type
1	15,06	n.a.	1548,850	484,768	97,35	n.a.	BM *
2	17,65	n.a.	39,348	13,196	2,65	n.a.	BMB
Total:			1588,198	497,964	100,00	0,000	

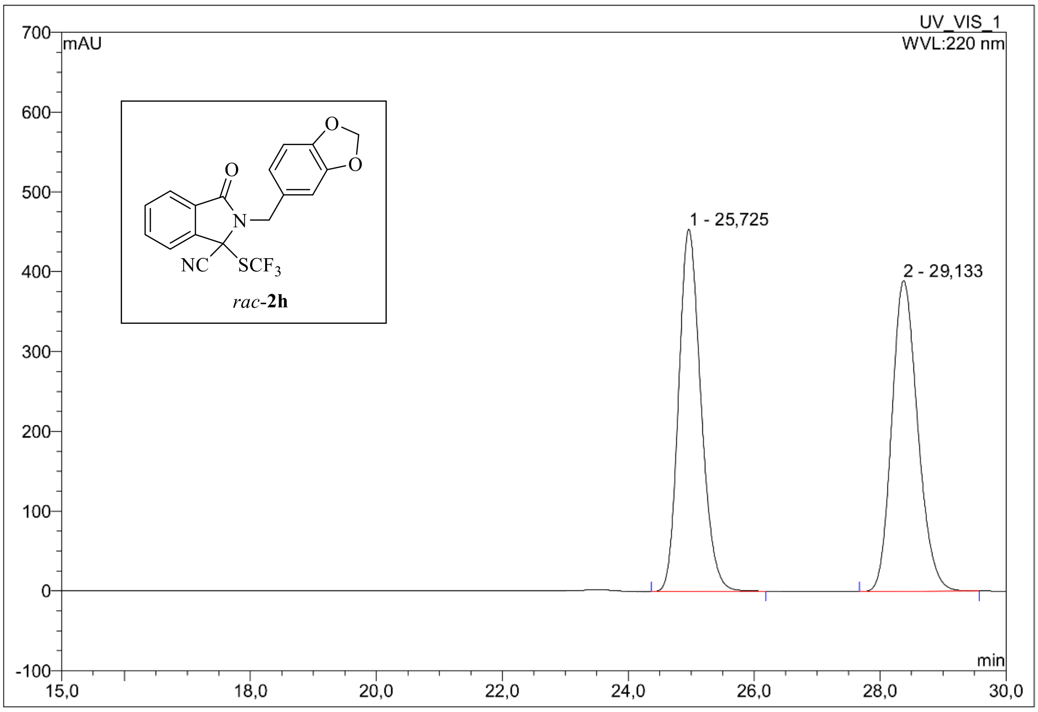

No.	Ret.Time min	Peak Name	Height mAU	Area mAU*min	Rel.Area %	Amount	Type
1	25,73	n.a.	453,797	185,946	50,03	n.a.	BMB
2	29,13	n.a.	389,091	185,738	49,97	n.a.	BMB
Total:			842,888	371,684	100,00	0,000	

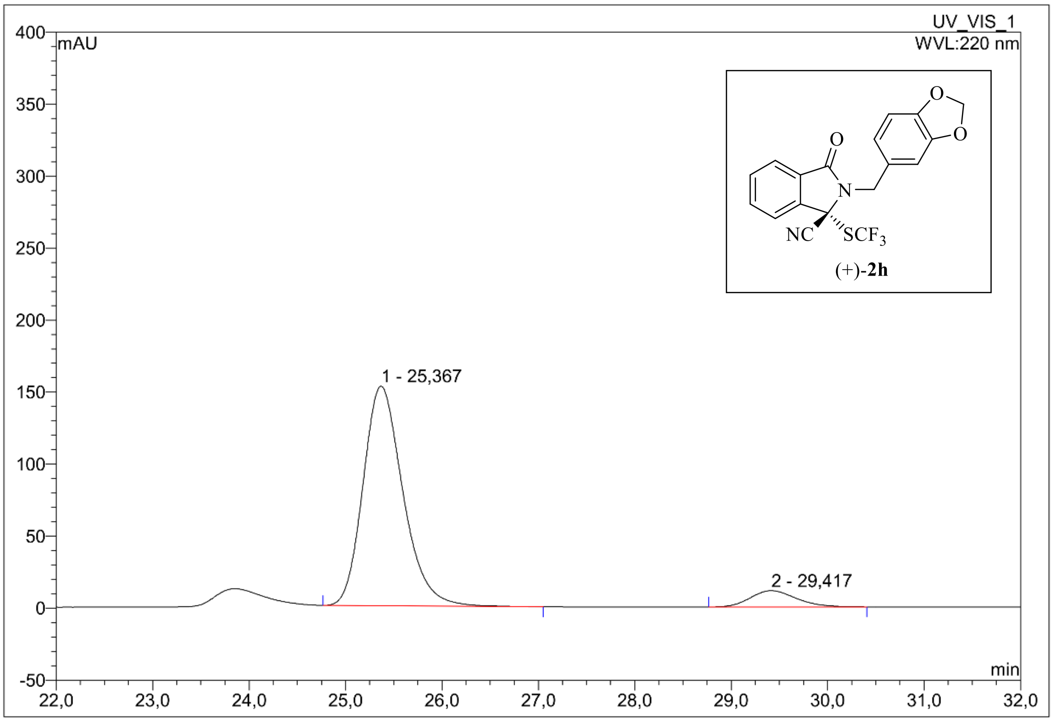

No.	Ret.Time min	Peak Name	Height mAU	Area mAU*min	Rel.Area %	Amount	Type
1	25,37	n.a.	152,574	73,112	92,13	n.a.	BMB*
2	29,42	n.a.	11,302	6,245	7,87	n.a.	BMB
Total:			163,876	79,357	100,00	0,000	

Chromatogram

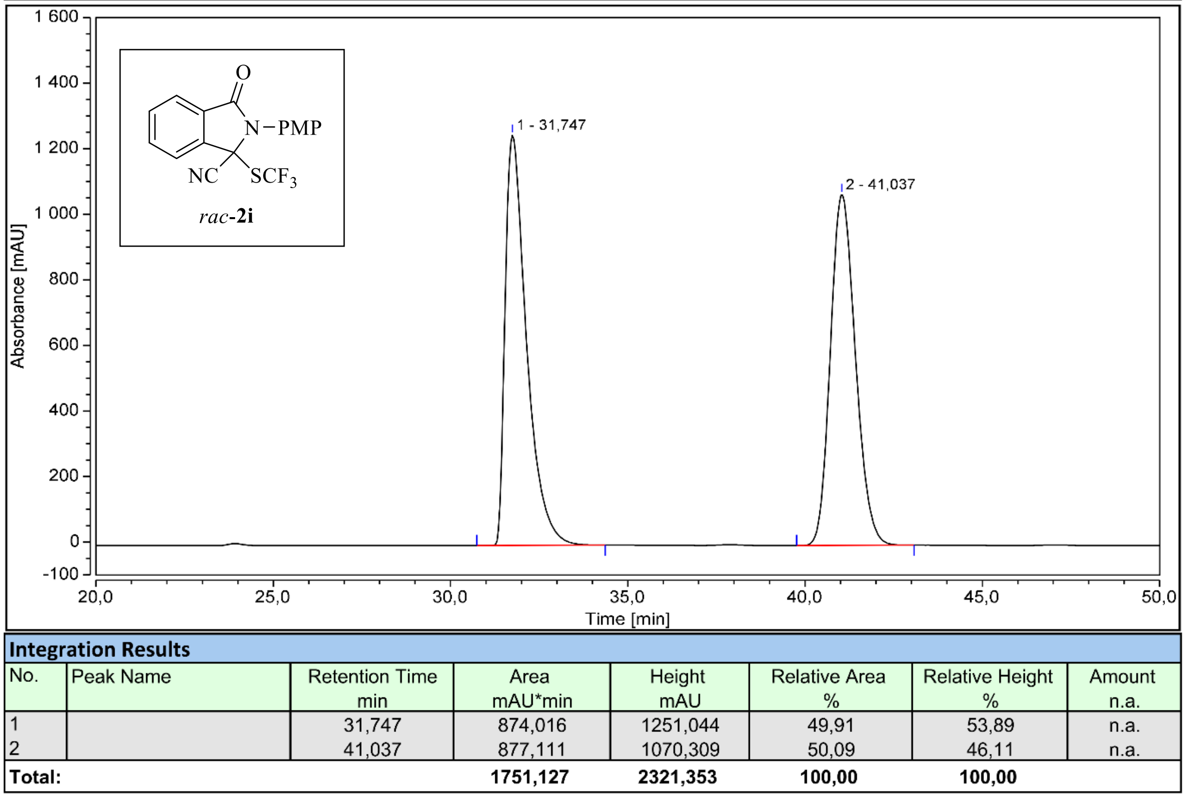

Integration Results

No.	Peak Name	Retention Time min	Area mAU*min	Height mAU	Relative Area %	Relative Height %	Amount n.a.
1		31,747	874,016	1251,044	49,91	53,89	n.a.
2		41,037	877,111	1070,309	50,09	46,11	n.a.
**Total:**			**1751,127**	**2321,353**	**100,00**	**100,00**	

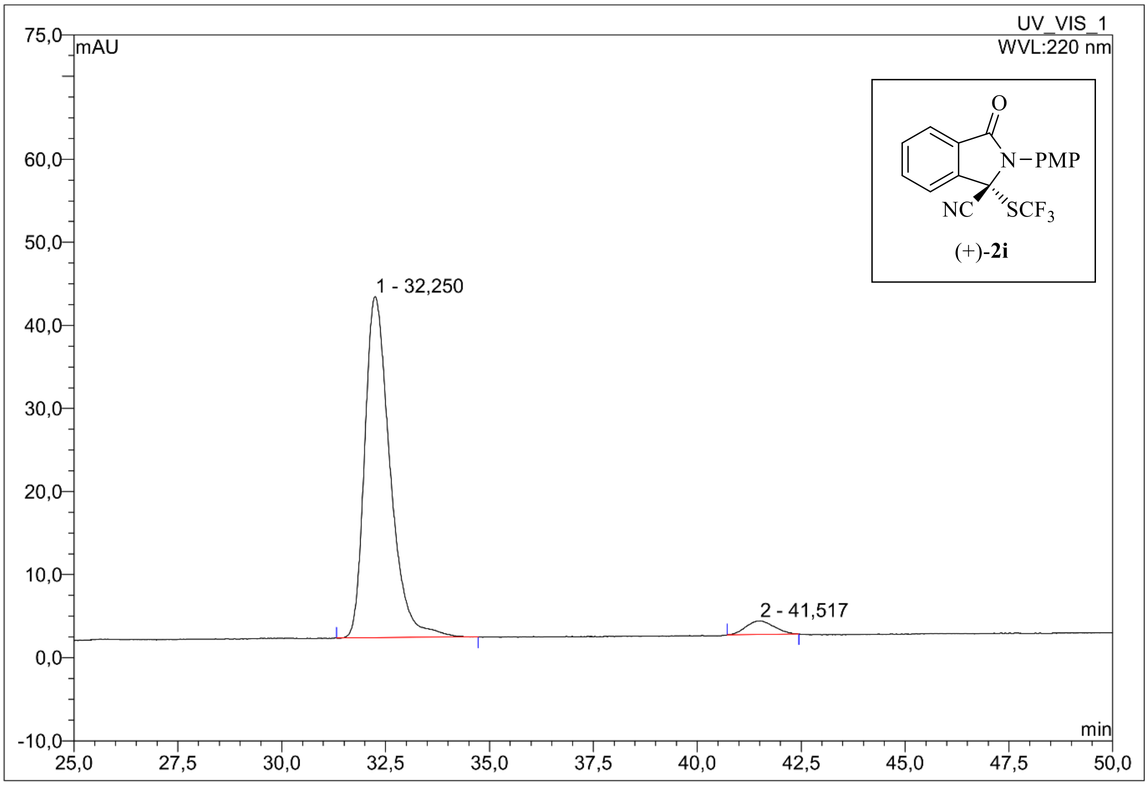

No.	Ret.Time min	Peak Name	Height mAU	Area mAU*min	Rel.Area %	Amount	Type
1	32,25	n.a.	41,055	28,941	95,79	n.a.	BMB*
2	41,52	n.a.	1,638	1,271	4,21	n.a.	BMB*
**Total:**			42,693	30,212	100,00	0,000	

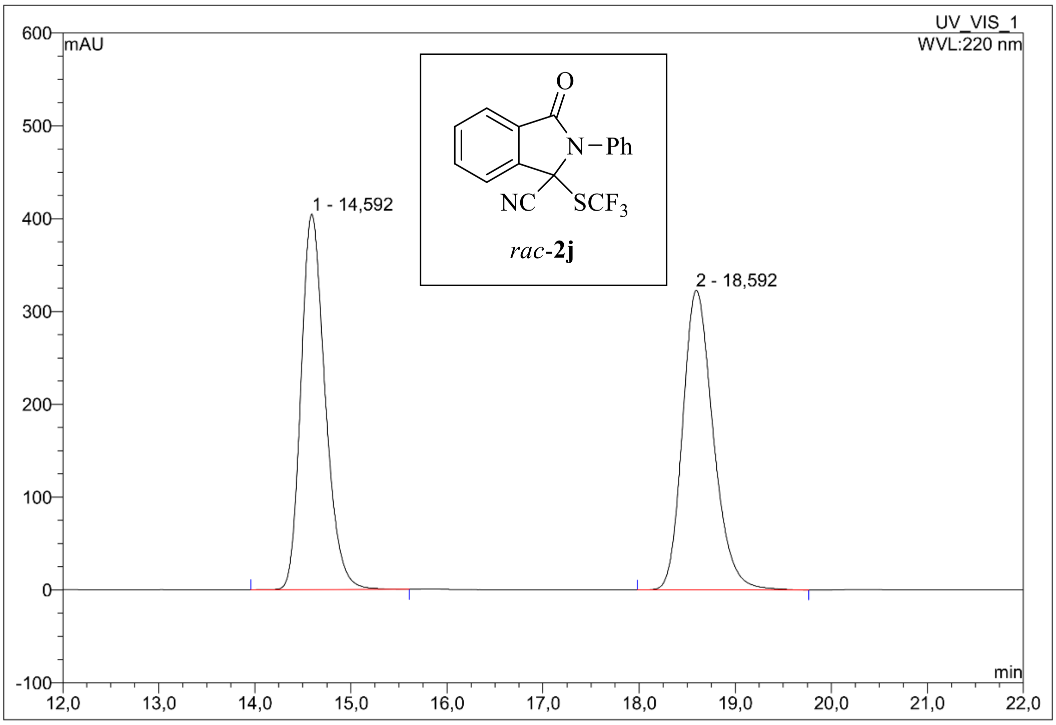

No.	Ret.Time min	Peak Name	Height mAU	Area mAU*min	Rel.Area %	Amount	Type
1	14,59	n.a.	404,457	117,728	49,68	n.a.	BMB
2	18,59	n.a.	322,756	119,243	50,32	n.a.	BMB
**Total:**			727,213	236,971	100,00	0,000	

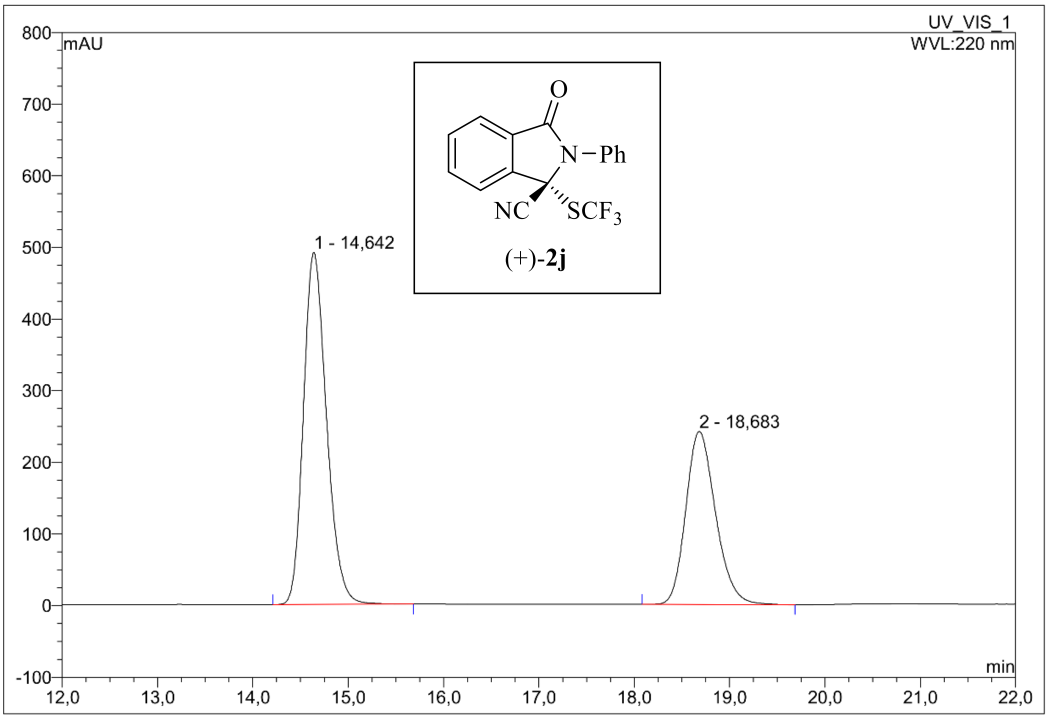

No.	Ret.Time min	Peak Name	Height mAU	Area mAU*min	Rel.Area %	Amount	Type
1	14,64	n.a.	491,236	137,638	61,16	n.a.	BMB
2	18,68	n.a.	241,594	87,410	38,84	n.a.	BMB
**Total:**			732,829	225,047	100,00	0,000	

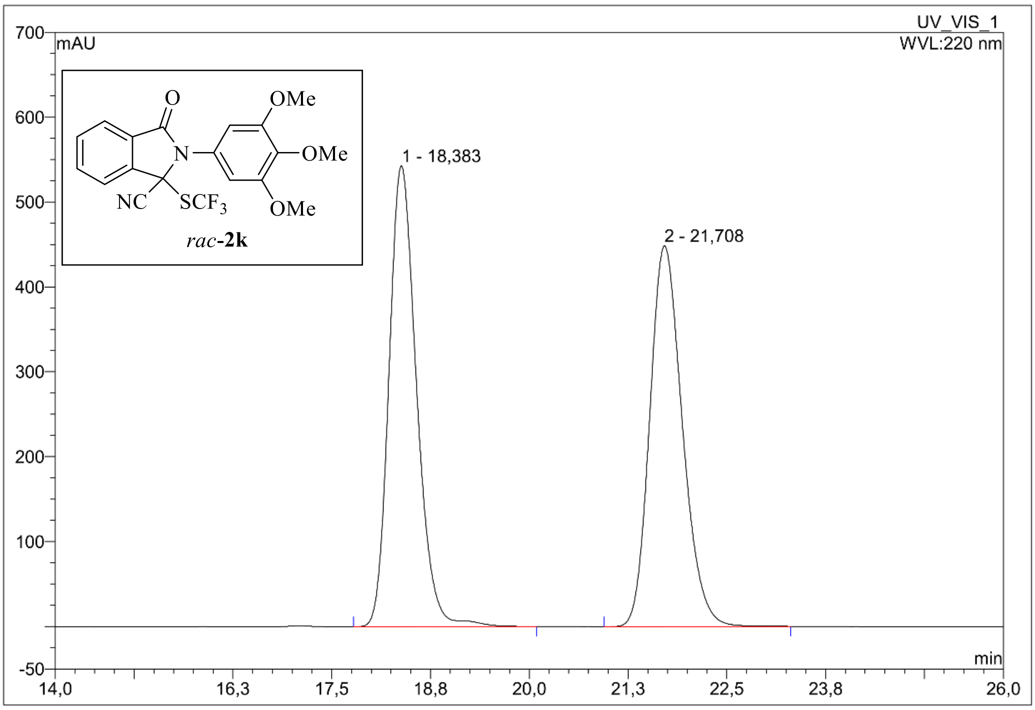

No.	Ret.Time min	Peak Name	Height mAU	Area mAU*min	Rel.Area %	Amount	Type
1	18,38	n.a.	543,191	219,203	50,55	n.a.	BMB
2	21,71	n.a.	448,594	214,392	49,45	n.a.	BMB
Total:			991,785	433,595	100,00	0,000	

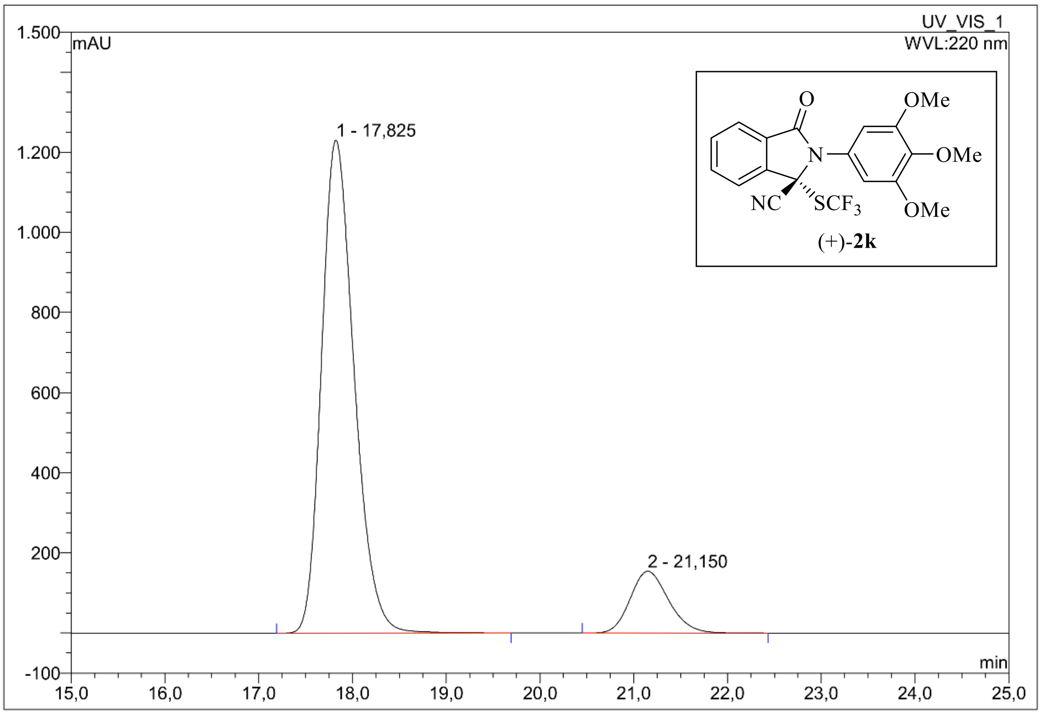

No.	Ret.Time min	Peak Name	Height mAU	Area mAU*min	Rel.Area %	Amount	Type
1	17,83	n.a.	1230,582	496,057	87,13	n.a.	BMB
2	21,15	n.a.	154,204	73,302	12,87	n.a.	BMB
Total:			1384,786	569,360	100,00	0,000	

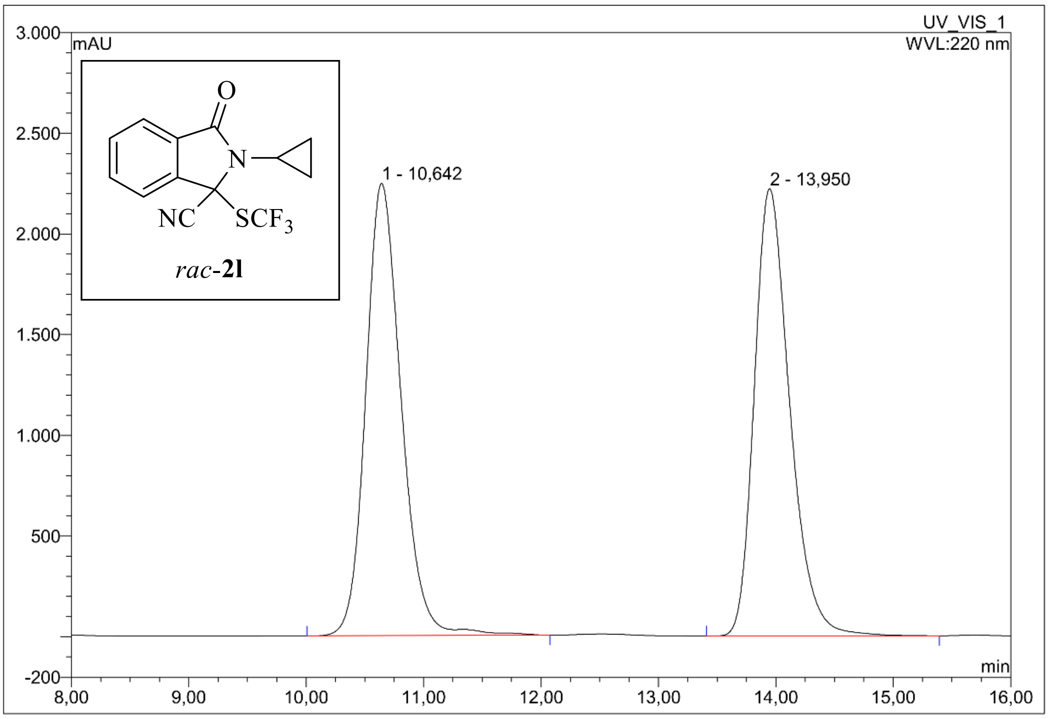

No.	Ret.Time min	Peak Name	Height mAU	Area mAU*min	Rel.Area %	Amount	Type
1	10,64	n.a.	2246,679	786,305	50,30	n.a.	BMB*
2	13,95	n.a.	2220,132	776,820	49,70	n.a.	BMB*
Total:			4466,812	1563,125	100,00	0,000	

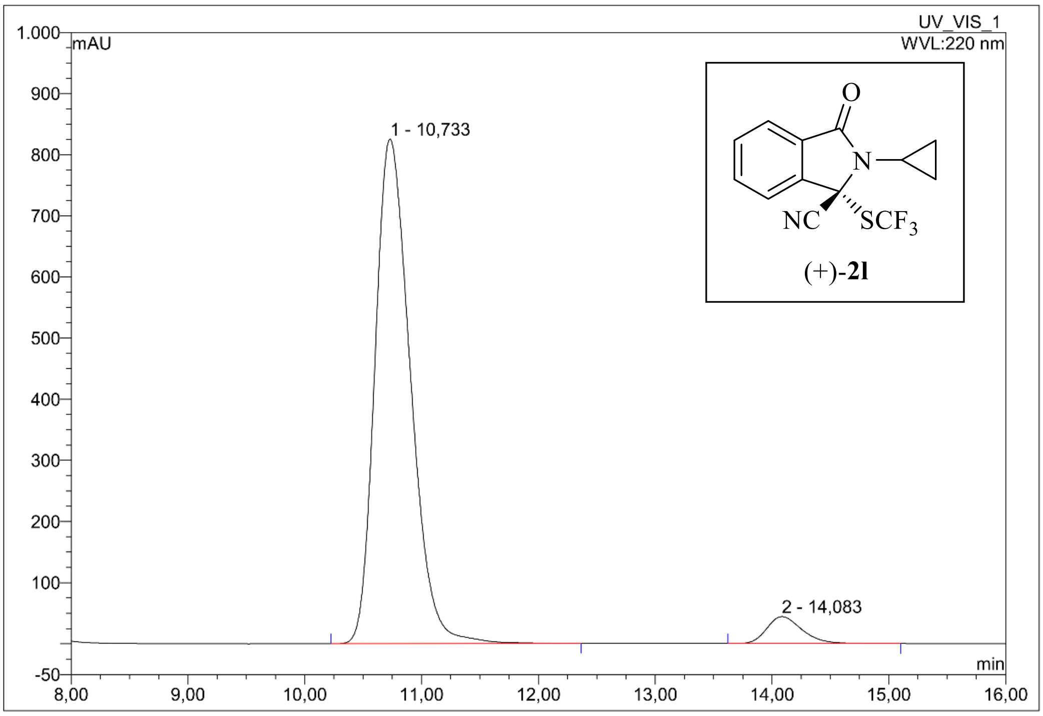

No.	Ret.Time min	Peak Name	Height mAU	Area mAU*min	Rel.Area %	Amount	Type
1	10,73	n.a.	825,247	290,274	94,95	n.a.	BMB
2	14,08	n.a.	44,097	15,435	5,05	n.a.	BMB
Total:			869,344	305,709	100,00	0,000	

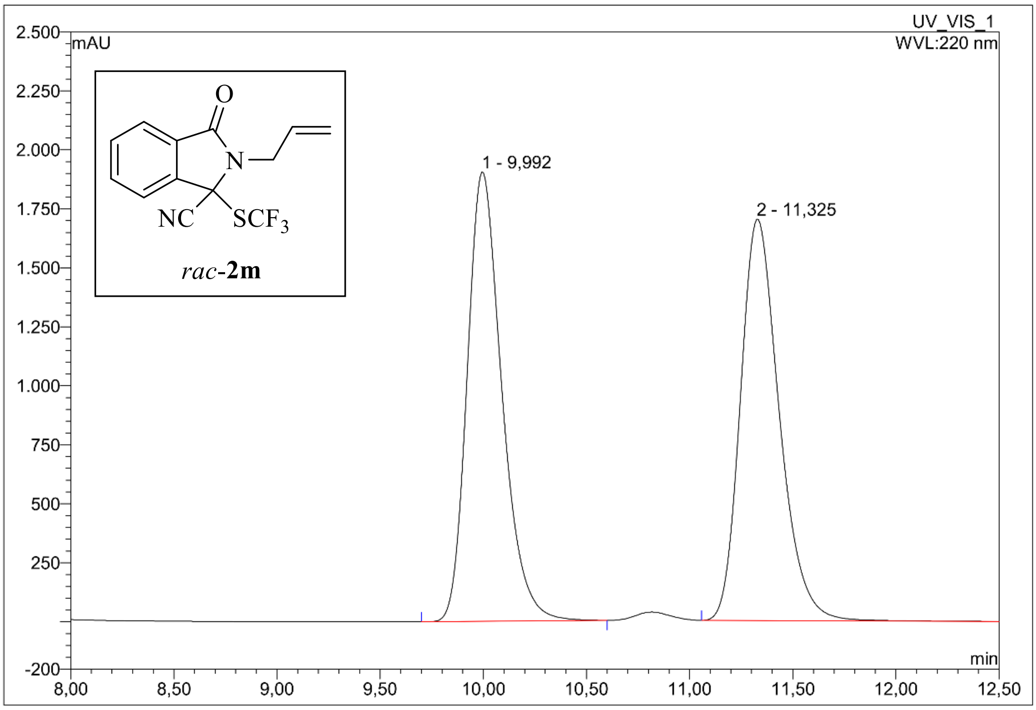

No.	Ret.Time min	Peak Name	Height mAU	Area mAU*min	Rel.Area %	Amount	Type
1	9,99	n.a.	1904,019	369,230	49,94	n.a.	BMB*
2	11,33	n.a.	1700,393	370,067	50,06	n.a.	BMB*
Total:			3604,412	739,298	100,00	0,000	

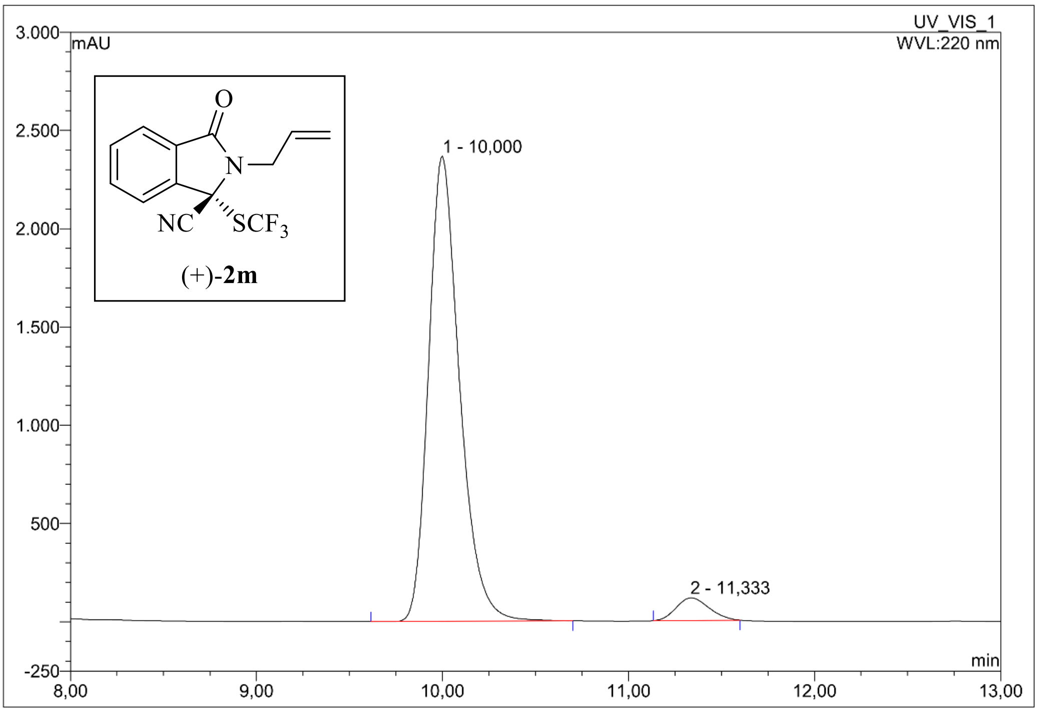

No.	Ret.Time min	Peak Name	Height mAU	Area mAU*min	Rel.Area %	Amount	Type
1	10,00	n.a.	2367,040	458,029	95,12	n.a.	BMB*
2	11,33	n.a.	115,463	23,493	4,88	n.a.	BMB*
Total:			2482,503	481,522	100,00	0,000	

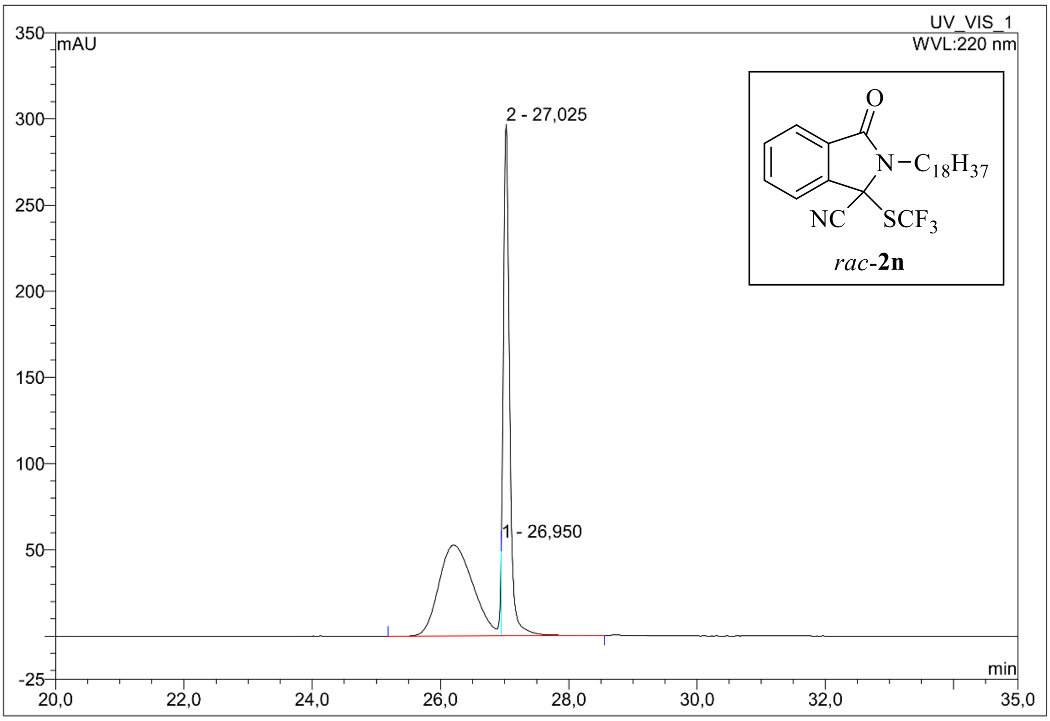

No.	Ret.Time min	Peak Name	Height mAU	Area mAU*min	Rel.Area %	Amount	Type
1	26,95	n.a.	54,808	33,343	49,97	n.a.	BM *
2	27,03	n.a.	296,684	33,387	50,03	n.a.	MB*
Total:			351,491	66,730	100,00	0,000	

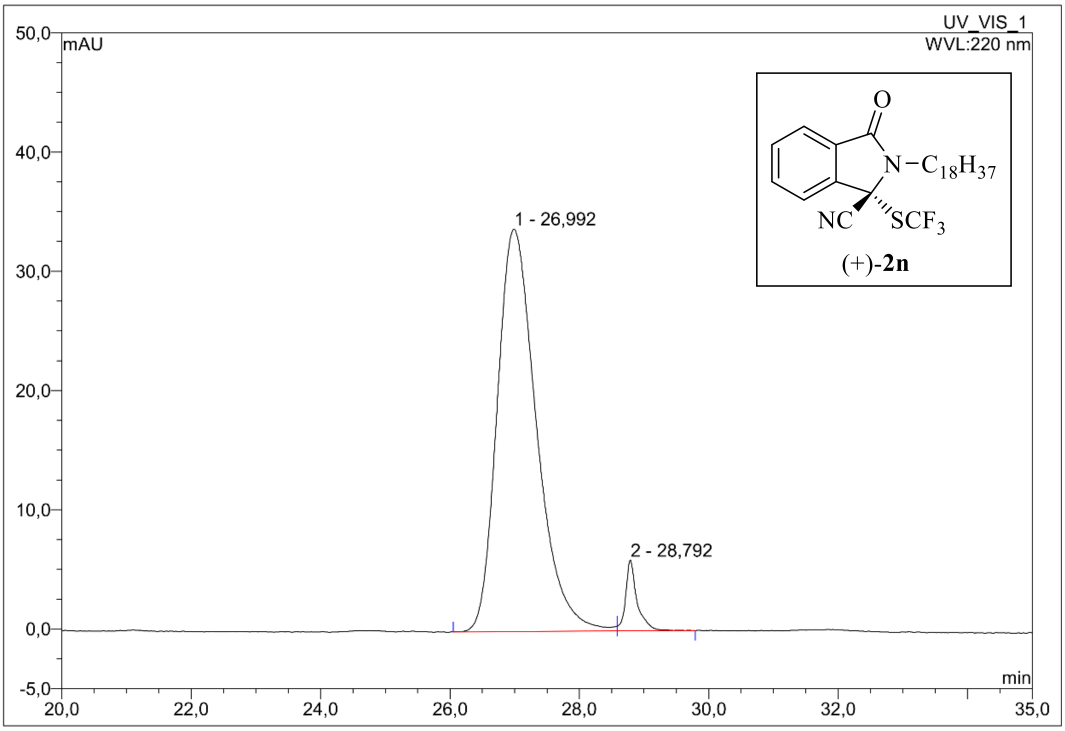

No.	Ret.Time min	Peak Name	Height mAU	Area mAU*min	Rel.Area %	Amount	Type
1	26,99	n.a.	33,760	23,759	95,08	n.a.	BM *
2	28,79	n.a.	5,931	1,229	4,92	n.a.	MB*
Total:			39,691	24,988	100,00	0,000	

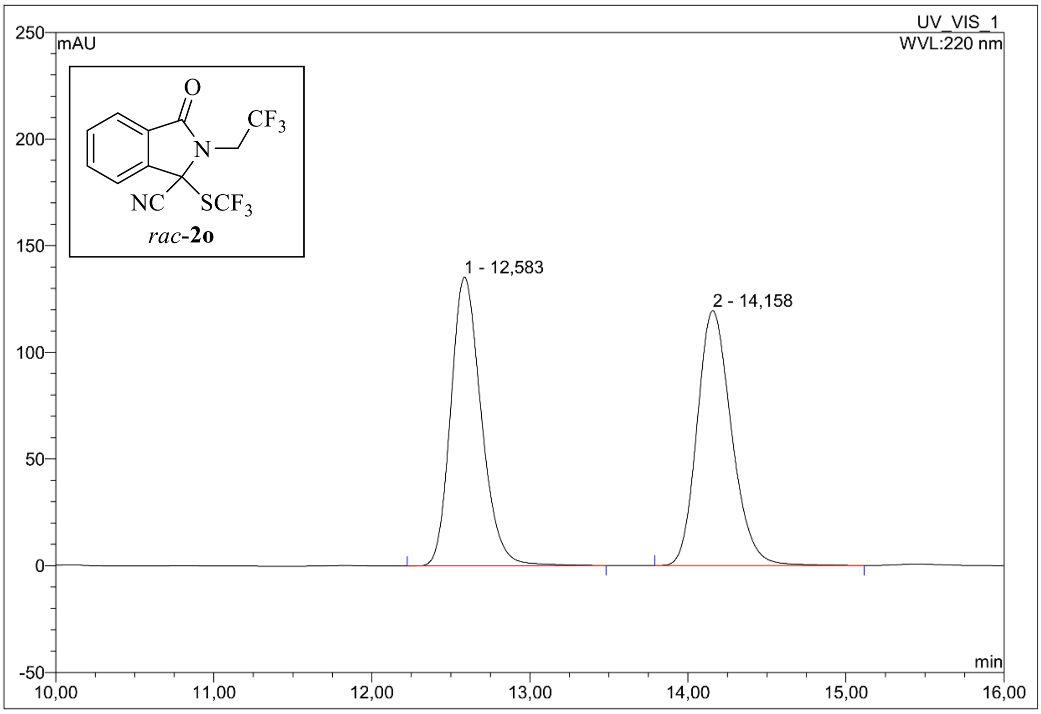

No.	Ret.Time min	Peak Name	Height mAU	Area mAU*min	Rel.Area %	Amount	Type
1	12,58	n.a.	135,366	30,275	50,04	n.a.	BMB
2	14,16	n.a.	119,545	30,228	49,96	n.a.	BMB
Total:			254,911	60,503	100,00	0,000	

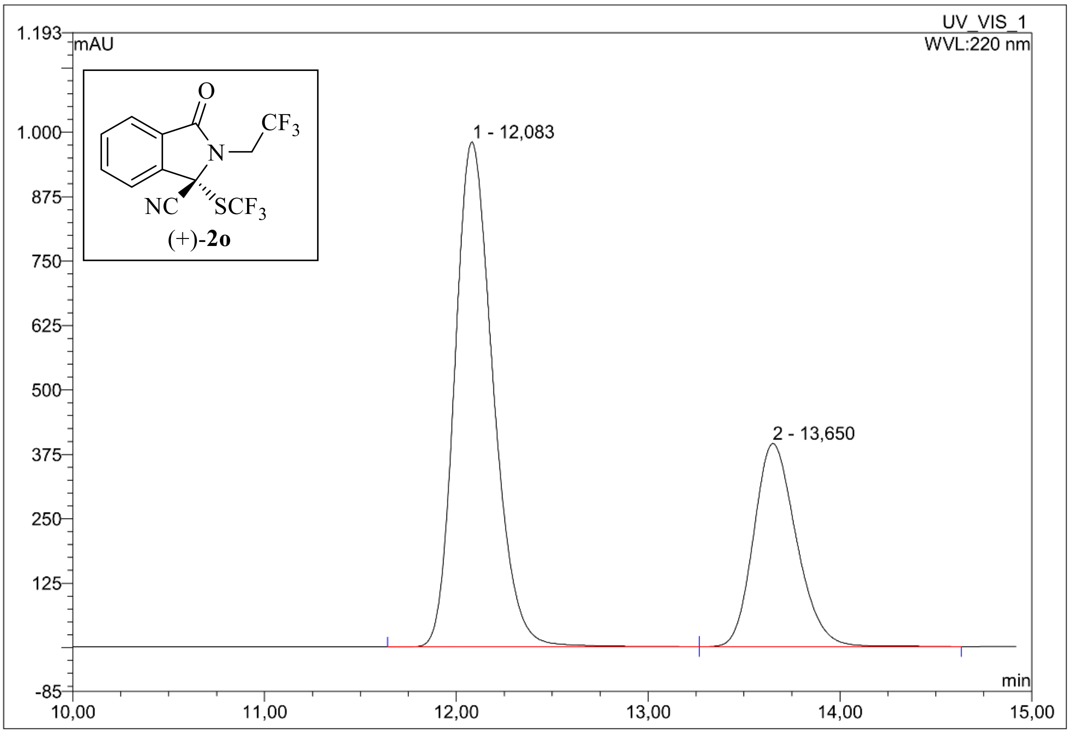

No.	Ret.Time min	Peak Name	Height mAU	Area mAU*min	Rel.Area %	Amount	Type
1	12,08	n.a.	980,185	226,454	68,97	n.a.	BM
2	13,65	n.a.	394,560	101,865	31,03	n.a.	MB
Total:			1374,745	328,319	100,00	0,000	

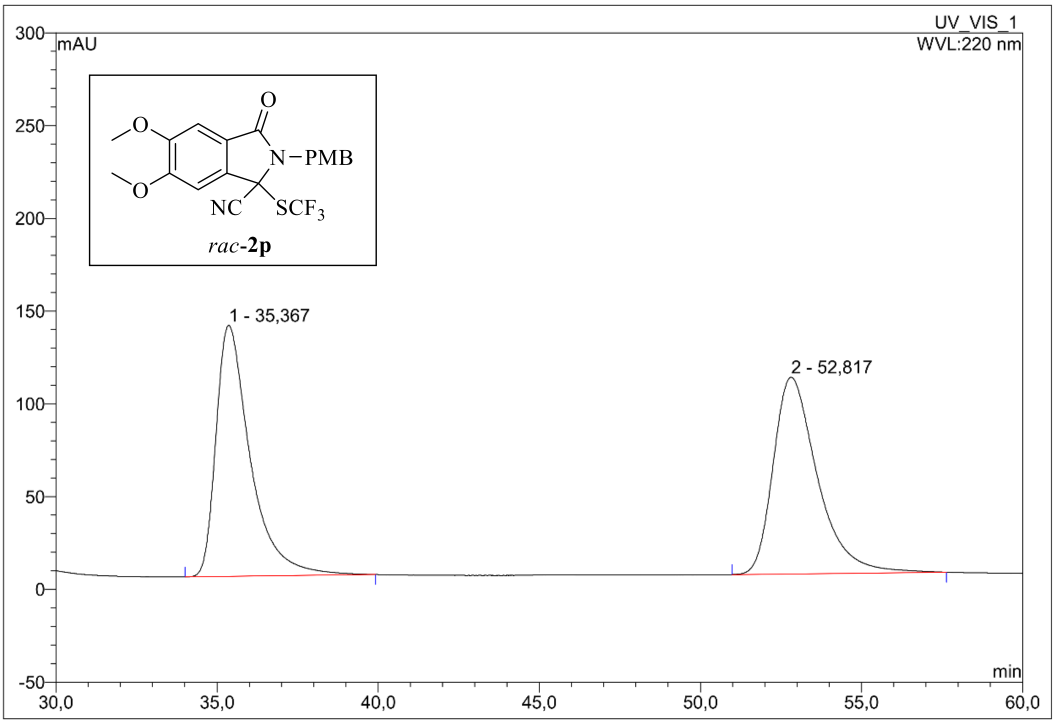

No.	Ret.Time min	Peak Name	Height mAU	Area mAU*min	Rel.Area %	Amount	Type
1	35,37	n.a.	135,338	167,081	49,73	n.a.	BMB
2	52,82	n.a.	106,071	168,905	50,27	n.a.	BMB
Total:			241,410	335,986	100,00	0,000	

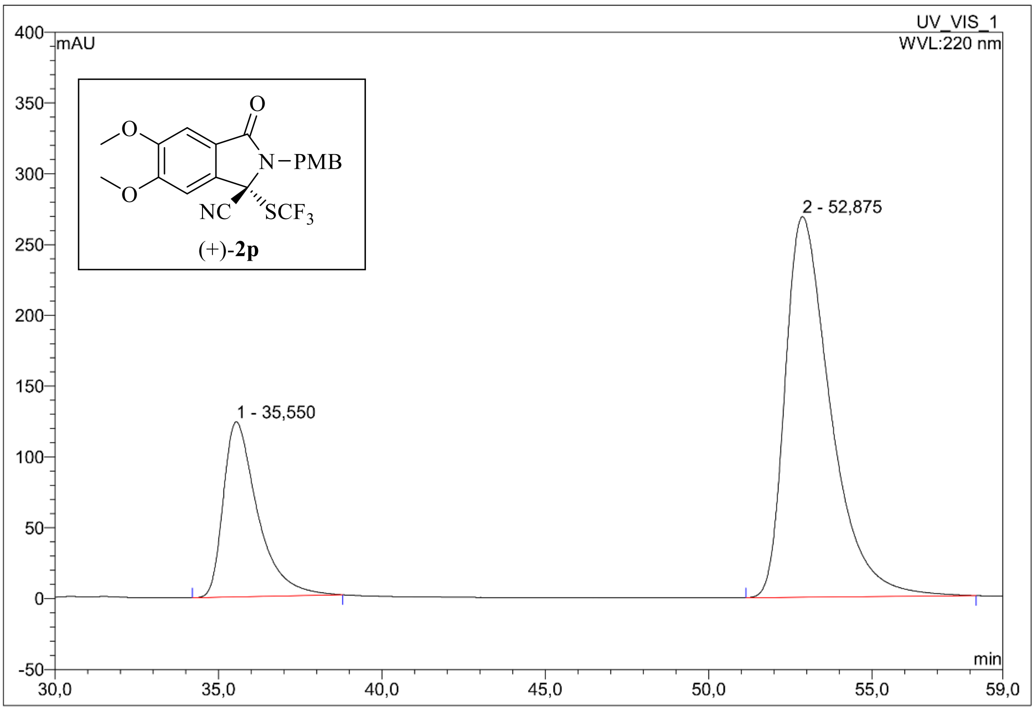

No.	Ret.Time min	Peak Name	Height mAU	Area mAU*min	Rel.Area %	Amount	Type
1	35,55	n.a.	123,636	150,429	25,78	n.a.	BMB
2	52,88	n.a.	268,773	432,972	74,22	n.a.	BMB
Total:			392,409	583,402	100,00	0,000	

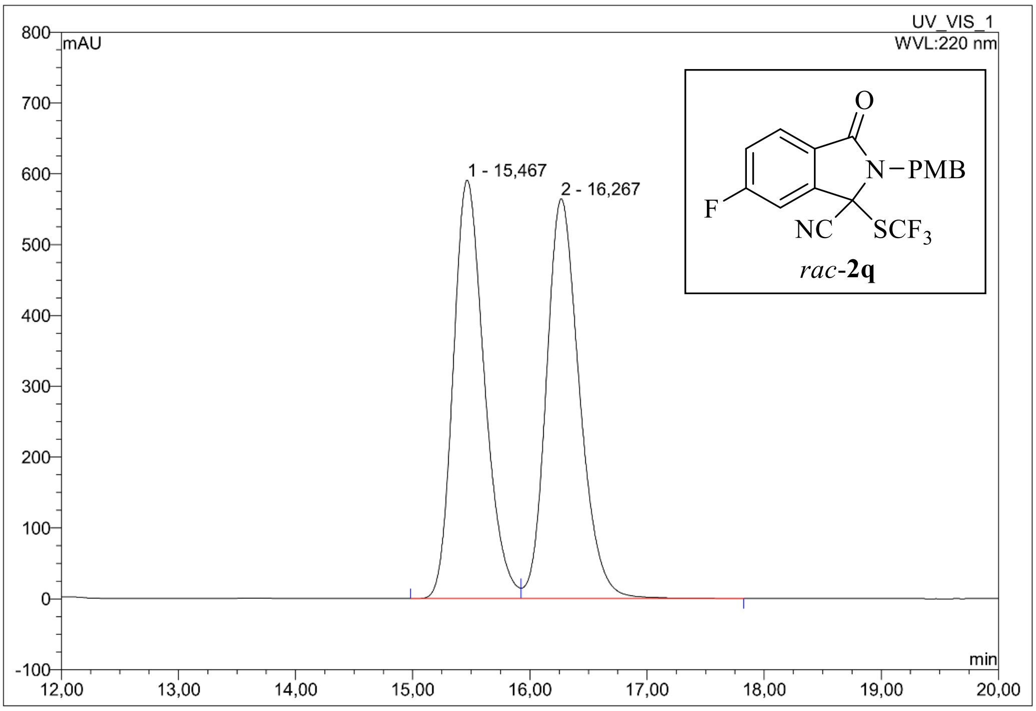

No.	Ret.Time min	Peak Name	Height mAU	Area mAU*min	Rel.Area %	Amount	Type
1	15,47	n.a.	591,091	181,786	49,55	n.a.	BM
2	16,27	n.a.	564,705	185,102	50,45	n.a.	MB
Total:			1155,796	366,888	100,00	0,000	

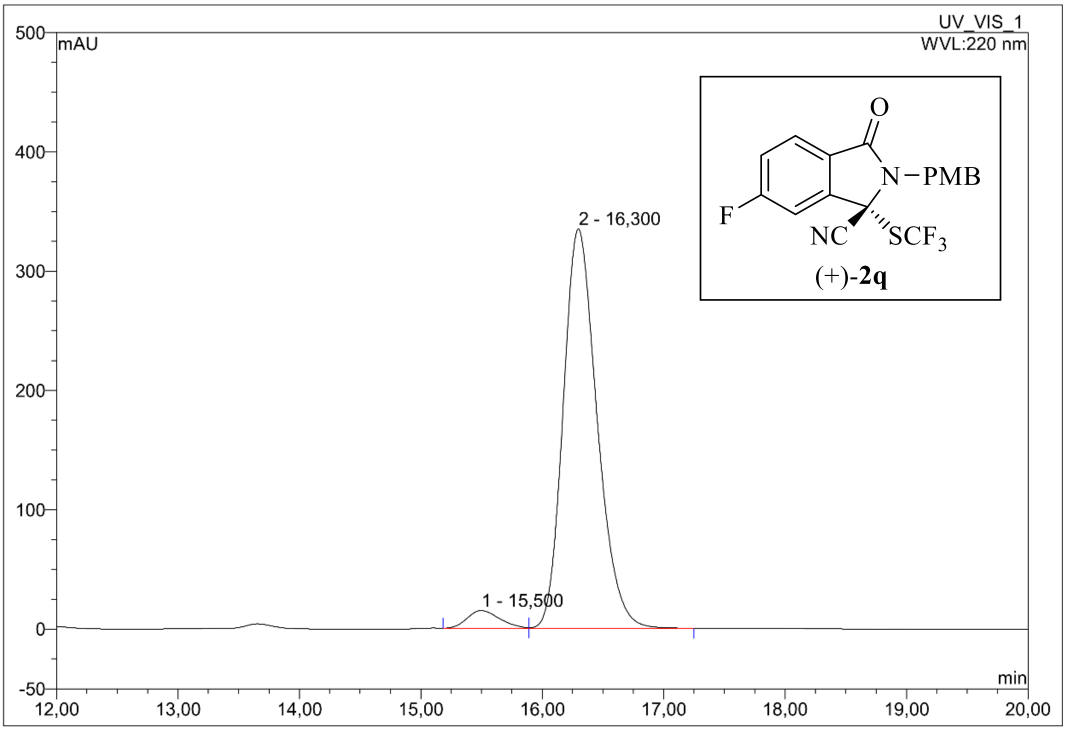

No.	Ret.Time min	Peak Name	Height mAU	Area mAU*min	Rel.Area %	Amount	Type
1	15,50	n.a.	14,800	4,555	4,04	n.a.	BM
2	16,30	n.a.	334,708	108,200	95,96	n.a.	MB
Total:			349,509	112,754	100,00	0,000	

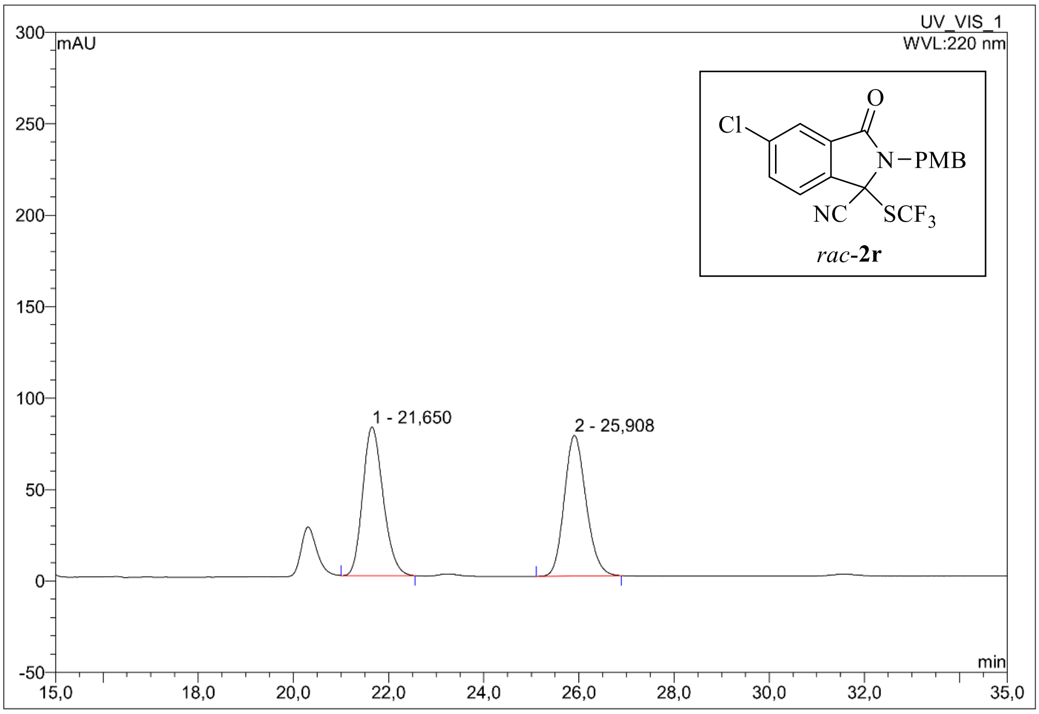

No.	Ret.Time min	Peak Name	Height mAU	Area mAU*min	Rel.Area %	Amount	Type
1	21,65	n.a.	81,197	40,437	49,79	n.a.	BMB*
2	25,91	n.a.	76,897	40,782	50,21	n.a.	BMB*
Total:			158,093	81,220	100,00	0,000	

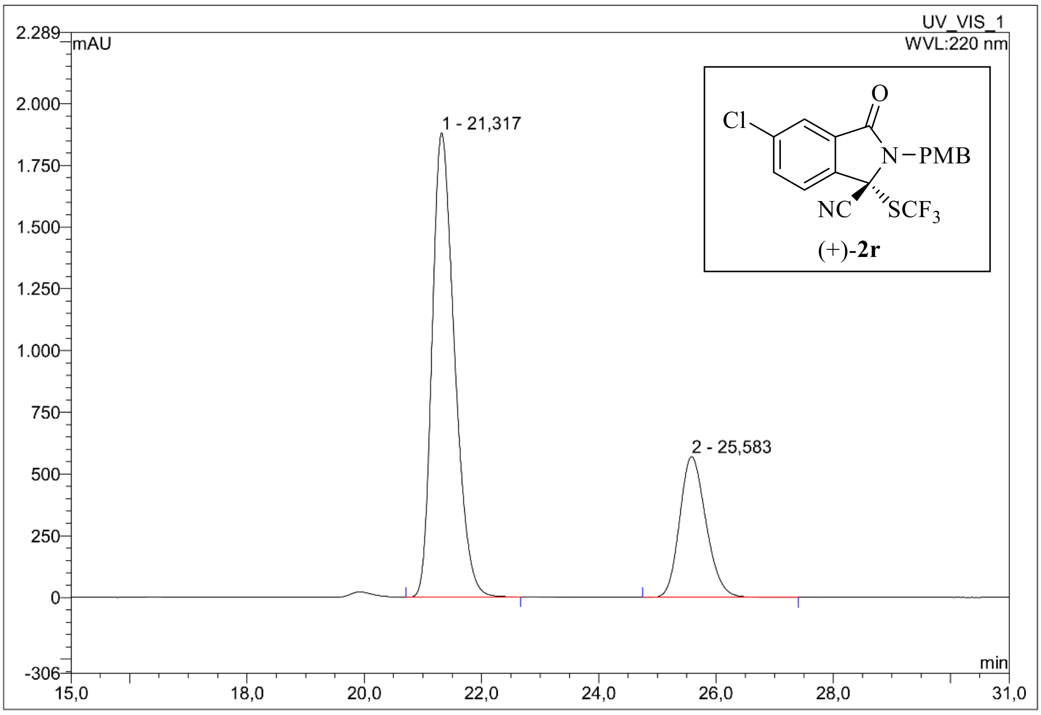

No.	Ret.Time min	Peak Name	Height mAU	Area mAU*min	Rel.Area %	Amount	Type
1	21,32	n.a.	1880,175	830,489	73,62	n.a.	BMB*
2	25,58	n.a.	570,039	297,579	26,38	n.a.	BMB
Total:			2450,214	1128,069	100,00	0,000	

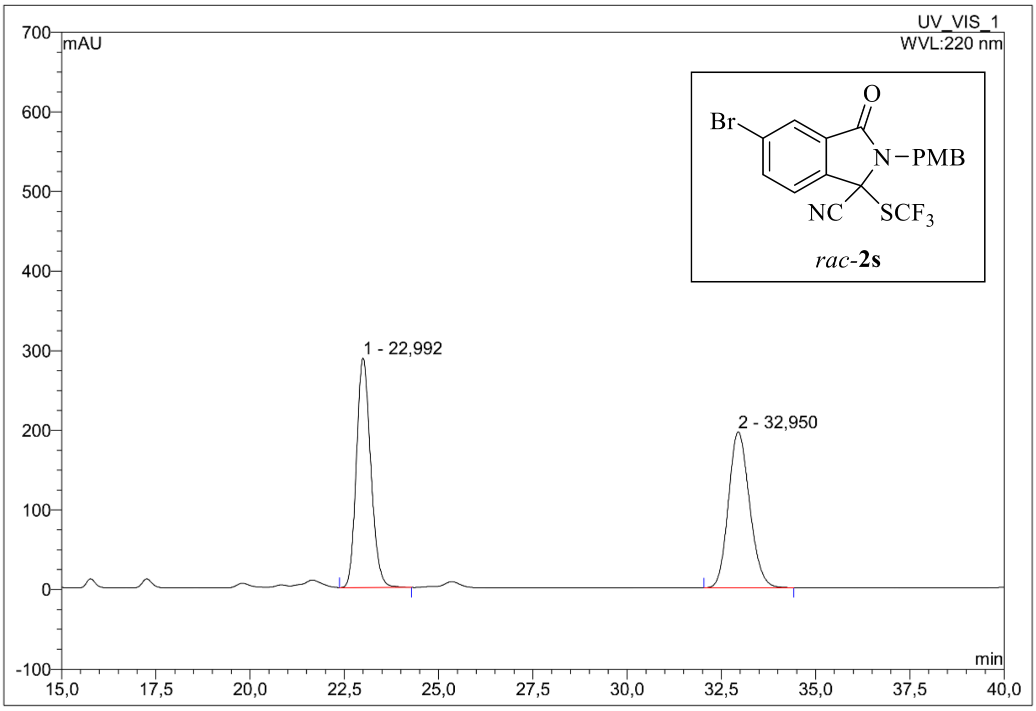

No.	Ret.Time min	Peak Name	Height mAU	Area mAU*min	Rel.Area %	Amount	Type
1	22,99	n.a.	288,145	127,061	49,96	n.a.	BMB
2	32,95	n.a.	195,672	127,276	50,04	n.a.	BMB
Total:			483,817	254,337	100,00	0,000	

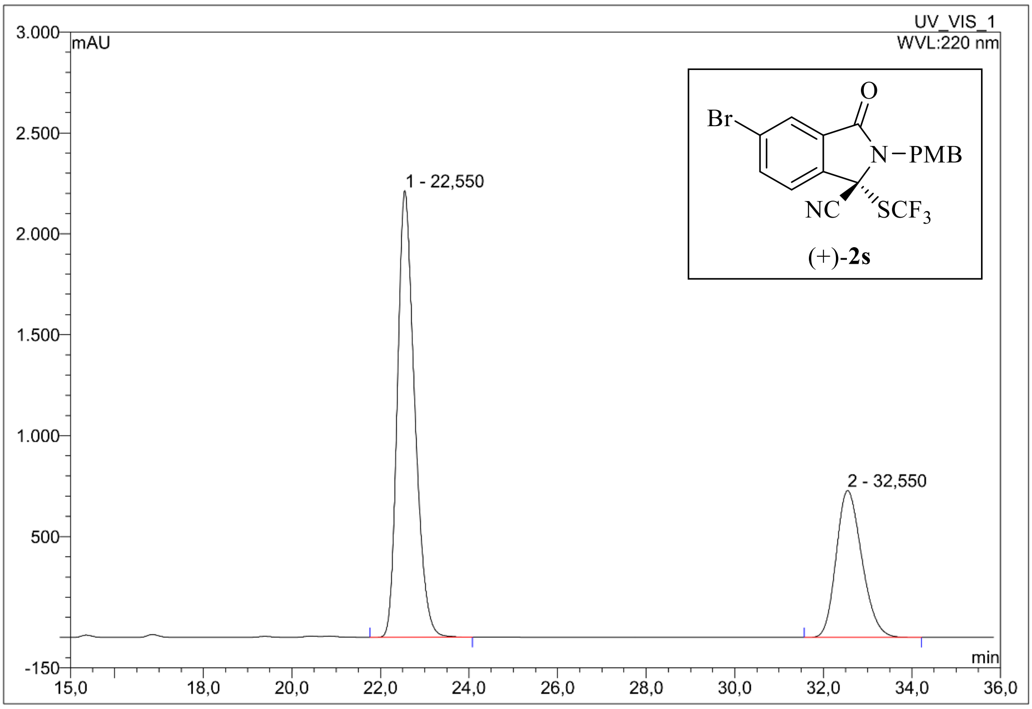

No.	Ret.Time min	Peak Name	Height mAU	Area mAU*min	Rel.Area %	Amount	Type
1	22,55	n.a.	2212,957	999,830	67,11	n.a.	BMB
2	32,55	n.a.	728,241	489,931	32,89	n.a.	BMB
Total:			2941,199	1489,761	100,00	0,000	

Chromatogram

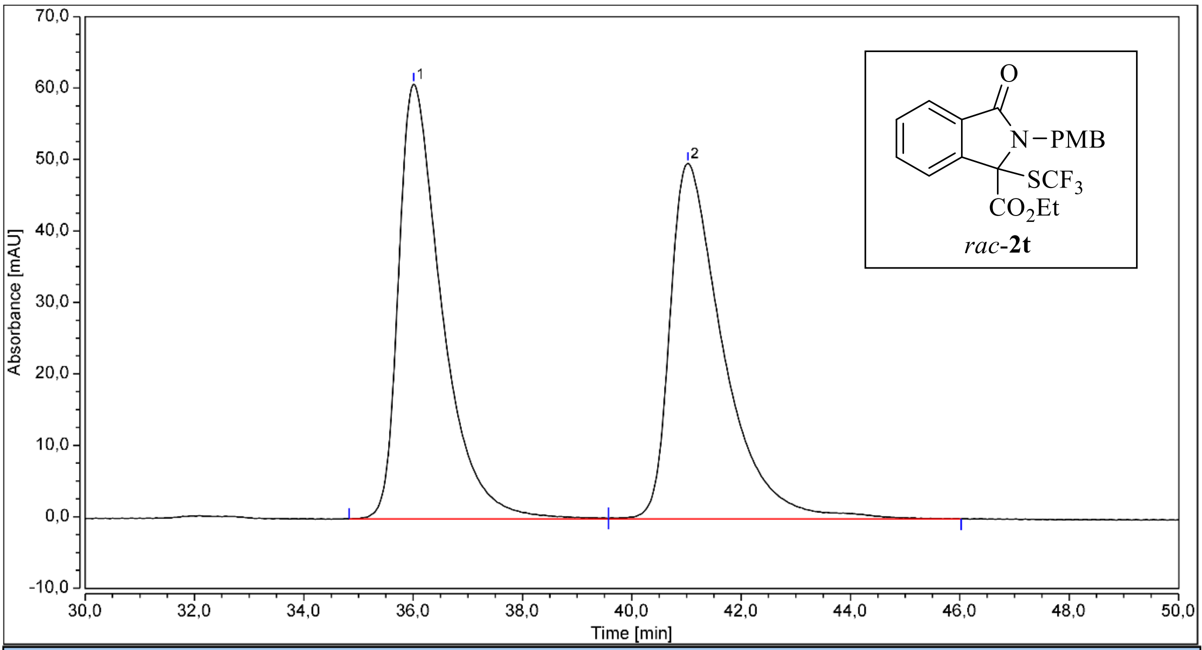

Integration Results

No.	Peak Name	Retention Time min	Area mAU*min	Height mAU	Relative Area %	Relative Height %	Amount n.a.
1		36,010	55,933	60,873	49,82	55,01	n.a.
2		41,027	56,335	49,777	50,18	44,99	n.a.
**Total:**			**112,269**	**110,650**	**100,00**	**100,00**	

Chromatogram

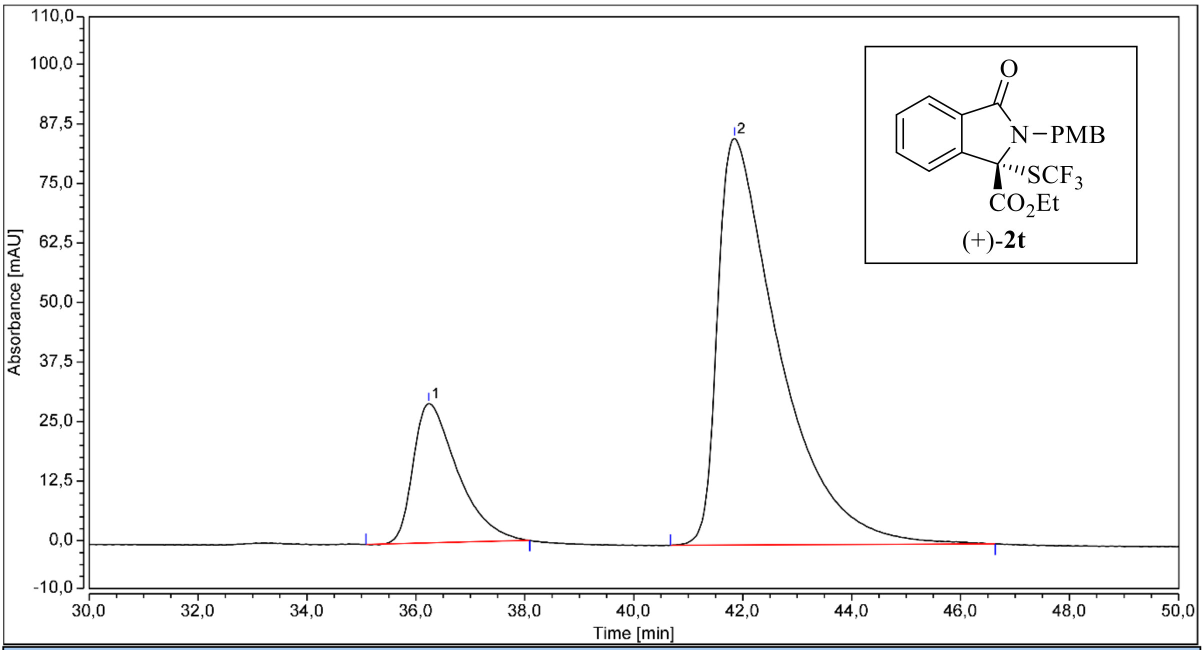

Integration Results

No.	Peak Name	Retention Time min	Area mAU*min	Height mAU	Relative Area %	Relative Height %	Amount n.a.
1		36,237	27,595	29,217	19,77	25,50	n.a.
2		41,847	111,977	85,361	80,23	74,50	n.a.
**Total:**			**139,572**	**114,578**	**100,00**	**100,00**	

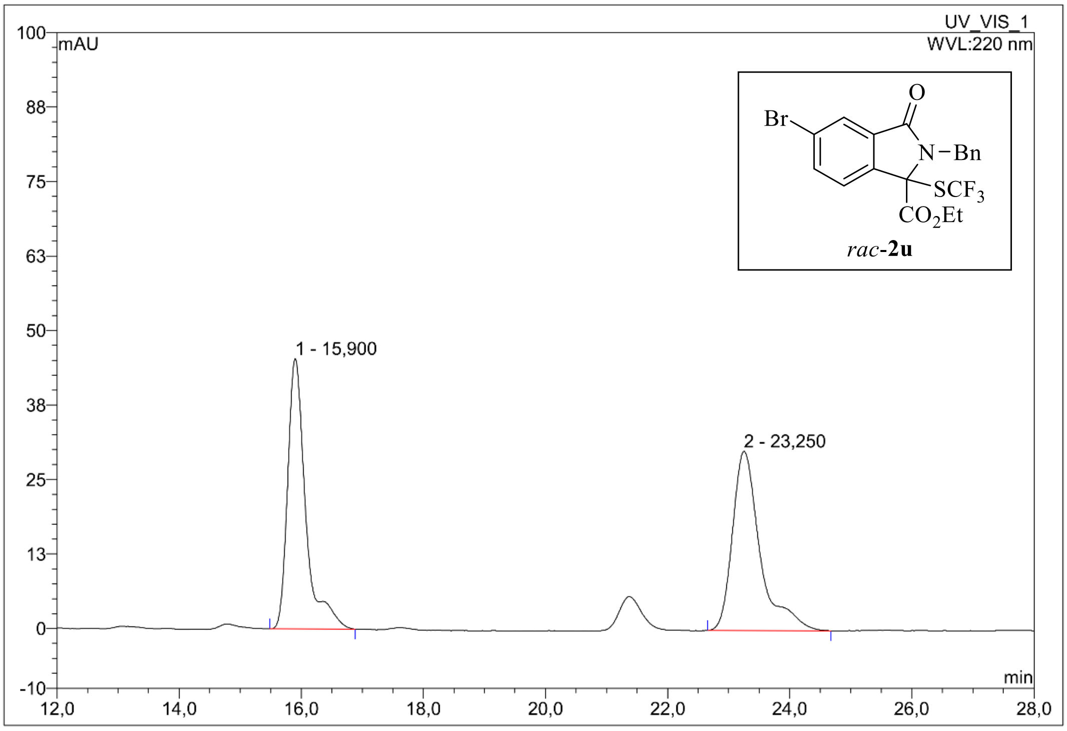

No.	Ret.Time min	Peak Name	Height mAU	Area mAU*min	Rel.Area %	Amount	Type
1	15,90	n.a.	45,366	15,472	49,45	n.a.	BMB
2	23,25	n.a.	30,083	15,817	50,55	n.a.	BMB
Total:			75,450	31,289	100,00	0,000	

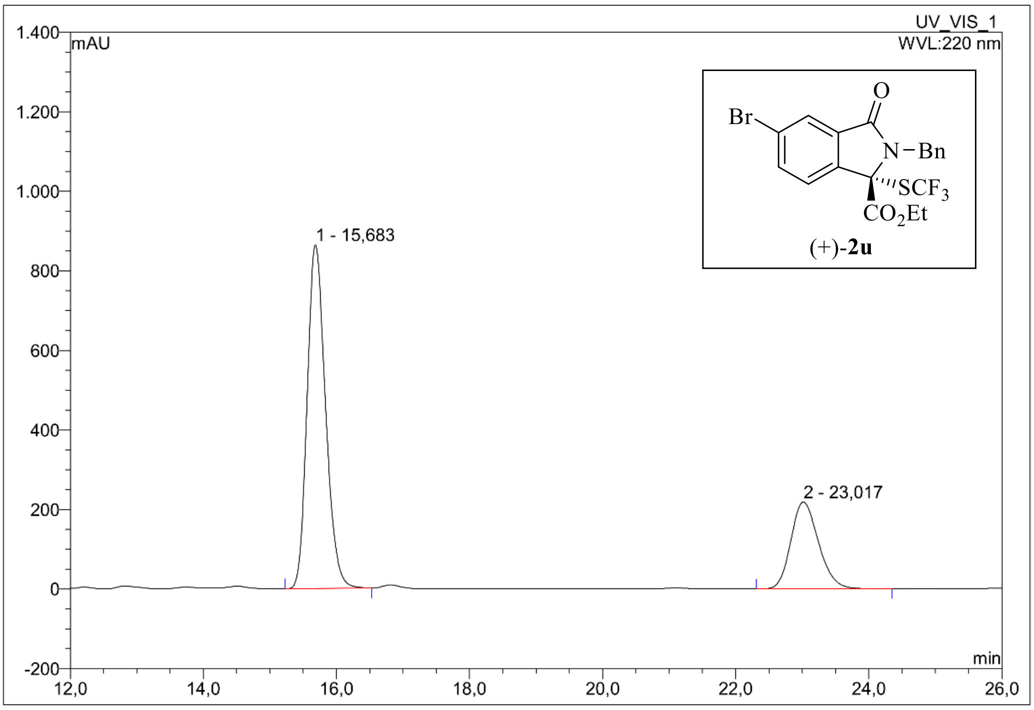

No.	Ret.Time min	Peak Name	Height mAU	Area mAU*min	Rel.Area %	Amount	Type
1	15,68	n.a.	864,103	275,657	72,40	n.a.	BMB*
2	23,02	n.a.	218,382	105,110	27,60	n.a.	BMB
Total:			1082,484	380,767	100,00	0,000	

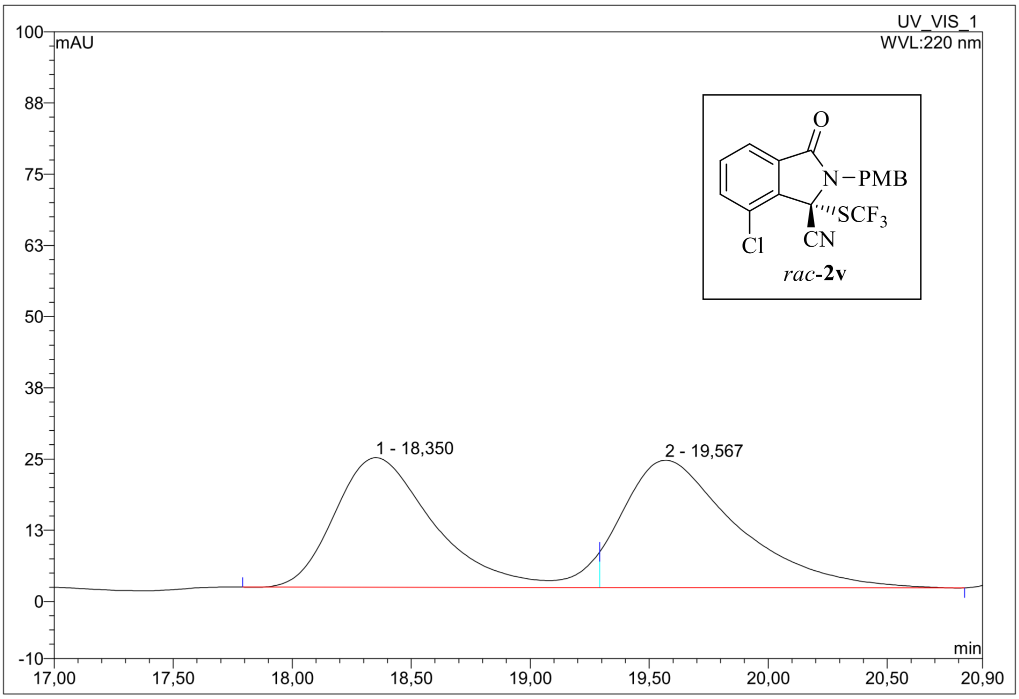

No.	Ret.Time<br>min	Peak Name	Height<br>mAU	Area<br>mAU*min	Rel.Area<br>%	Amount	Type
1	18,35	n.a.	22,744	11,439	48,42	n.a.	BM *
2	19,57	n.a.	22,343	12,186	51,58	n.a.	MB*
Total:			45,086	23,625	100,00	0,000	

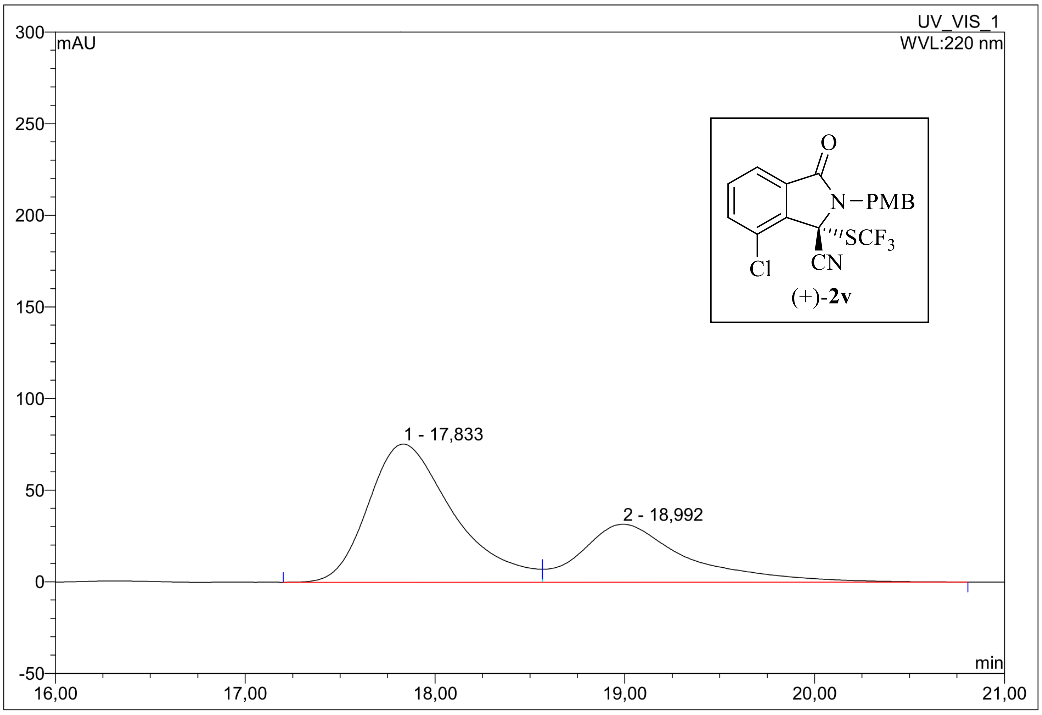

No.	Ret.Time<br>min	Peak Name	Height<br>mAU	Area<br>mAU*min	Rel.Area<br>%	Amount	Type
1	17,83	n.a.	75,397	39,393	66,10	n.a.	BM
2	18,99	n.a.	31,620	20,208	33,90	n.a.	MB
Total:			107,017	59,601	100,00	0,000	

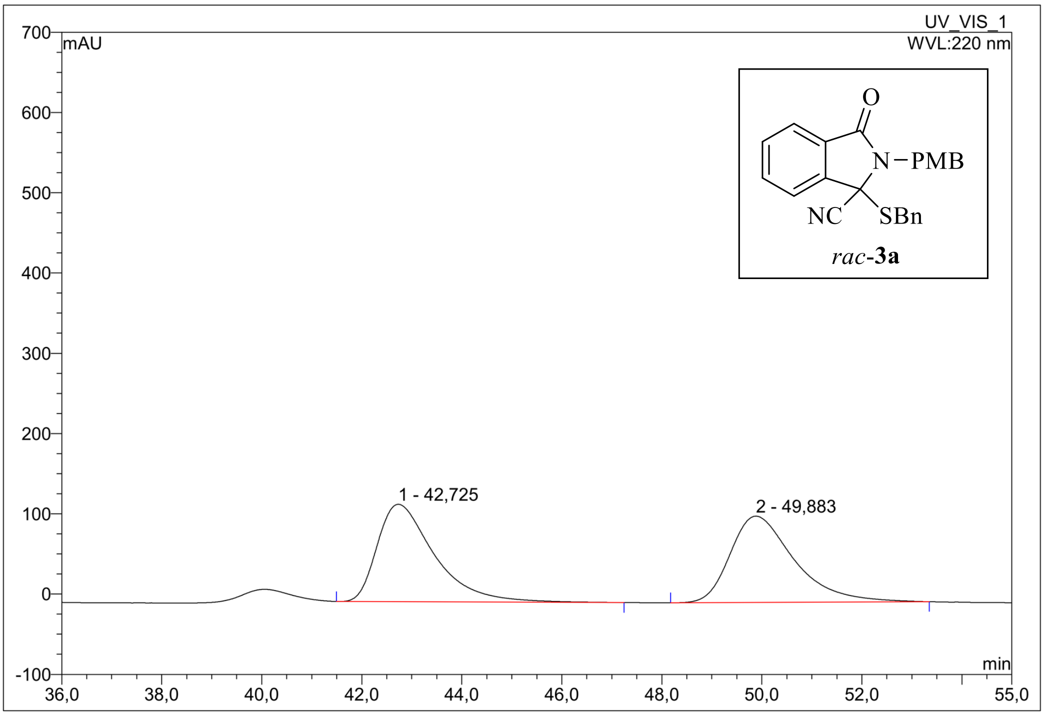

No.	Ret.Time min	Peak Name	Height mAU	Area mAU*min	Rel.Area %	Amount	Type
1	42,73	n.a.	121,378	160,451	50,20	n.a.	BMB*
2	49,88	n.a.	107,489	159,156	49,80	n.a.	BMB*
Total:			228,866	319,608	100,00	0,000	

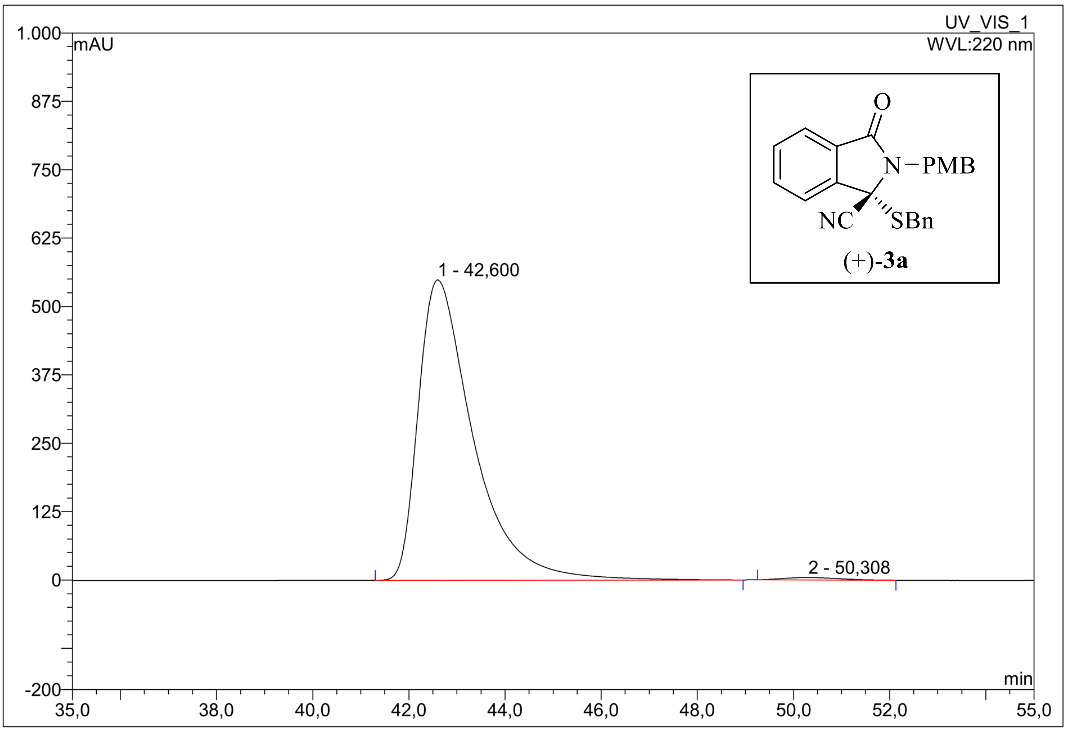

No.	Ret.Time min	Peak Name	Height mAU	Area mAU*min	Rel.Area %	Amount	Type
1	42,60	n.a.	548,928	727,817	99,20	n.a.	BMB*
2	50,31	n.a.	4,456	5,862	0,80	n.a.	BMB*
Total:			553,384	733,680	100,00	0,000	

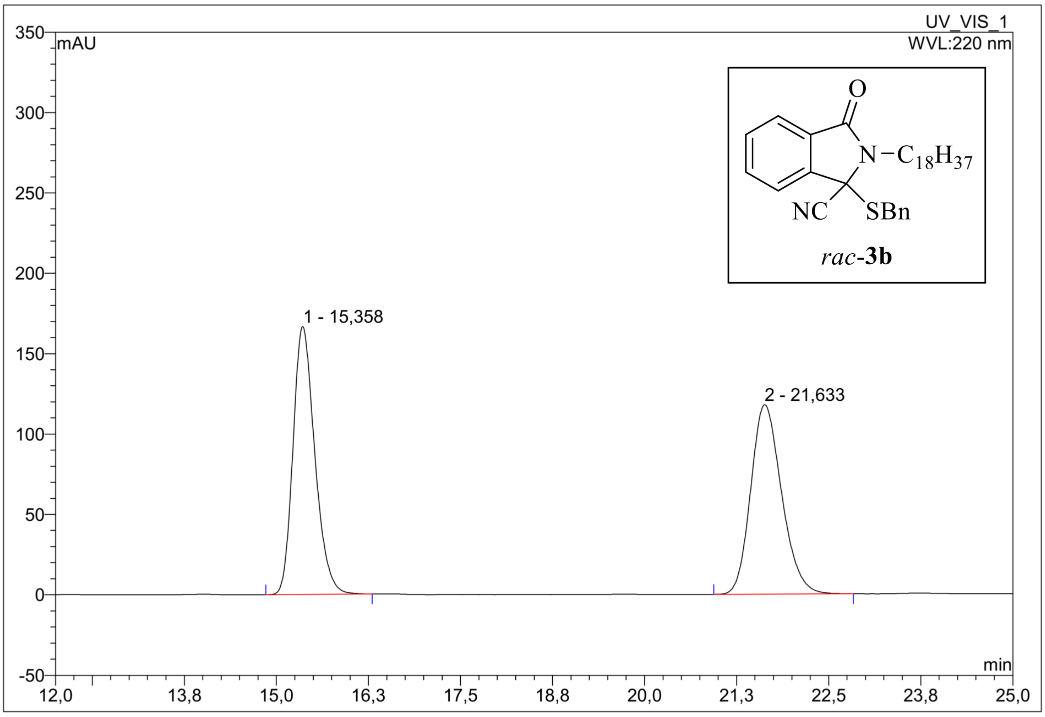

No.	Ret.Time min	Peak Name	Height mAU	Area mAU*min	Rel.Area %	Amount	Type
1	15,36	n.a.	166,653	57,137	49,92	n.a.	BMB
2	21,63	n.a.	117,897	57,315	50,08	n.a.	BMB
Total:			284,551	114,452	100,00	0,000	

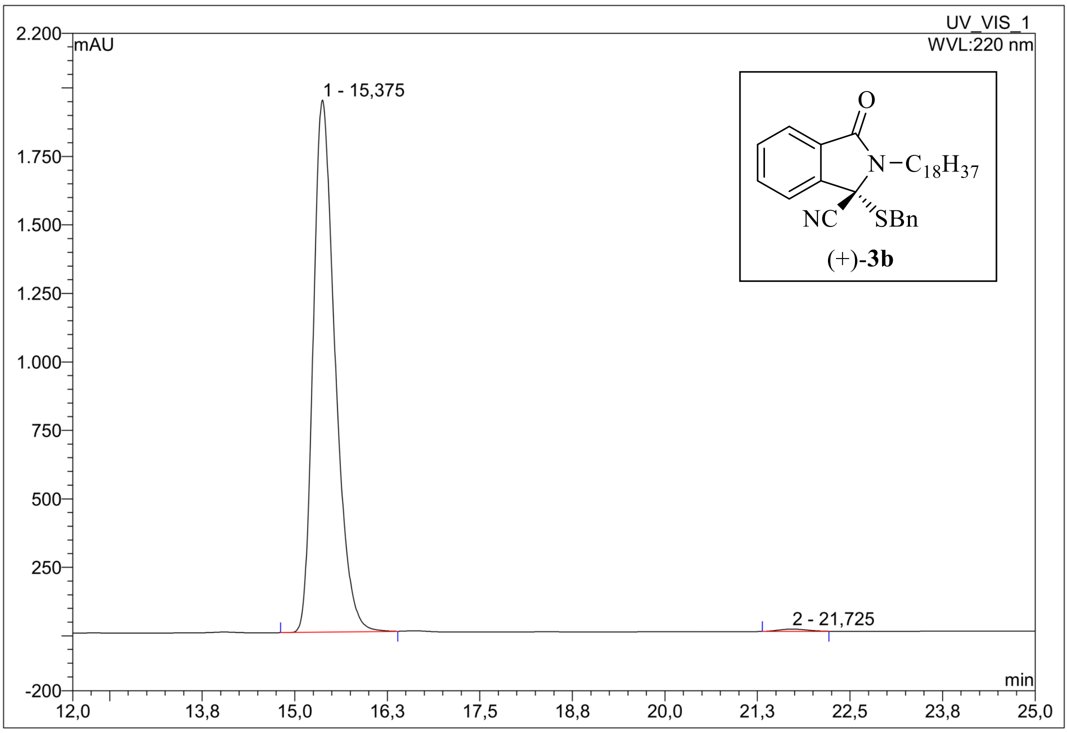

No.	Ret.Time min	Peak Name	Height mAU	Area mAU*min	Rel.Area %	Amount	Type
1	15,38	n.a.	1942,539	664,939	99,45	n.a.	BMB*
2	21,73	n.a.	8,388	3,657	0,55	n.a.	BMB*
Total:			1950,927	668,597	100,00	0,000	

Chromatogram

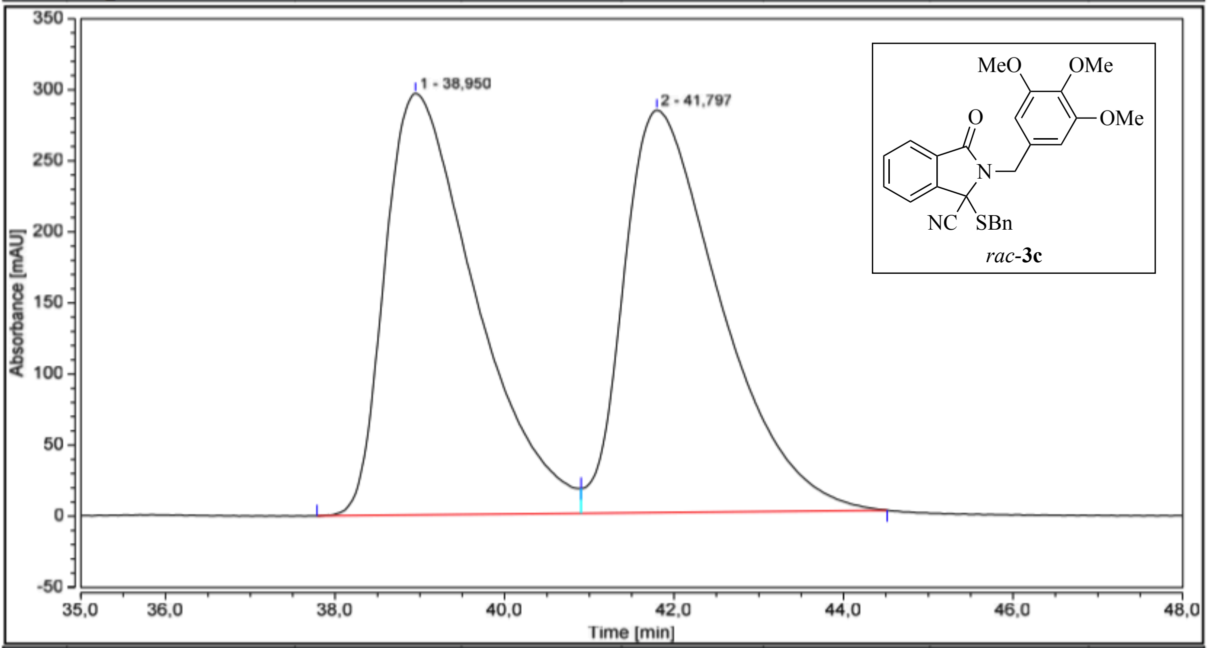

Integration Results

No.	Peak Name	Retention Time min	Area mAU*min	Height mAU	Relative Area %	Relative Height %	Amount n.a.
1		38,950	367,954	296,386	49,47	51,17	n.a.
2		41,797	375,863	282,882	50,53	48,83	n.a.
**Total:**			**743,818**	**579,268**	**100,00**	**100,00**	

Chromatogram

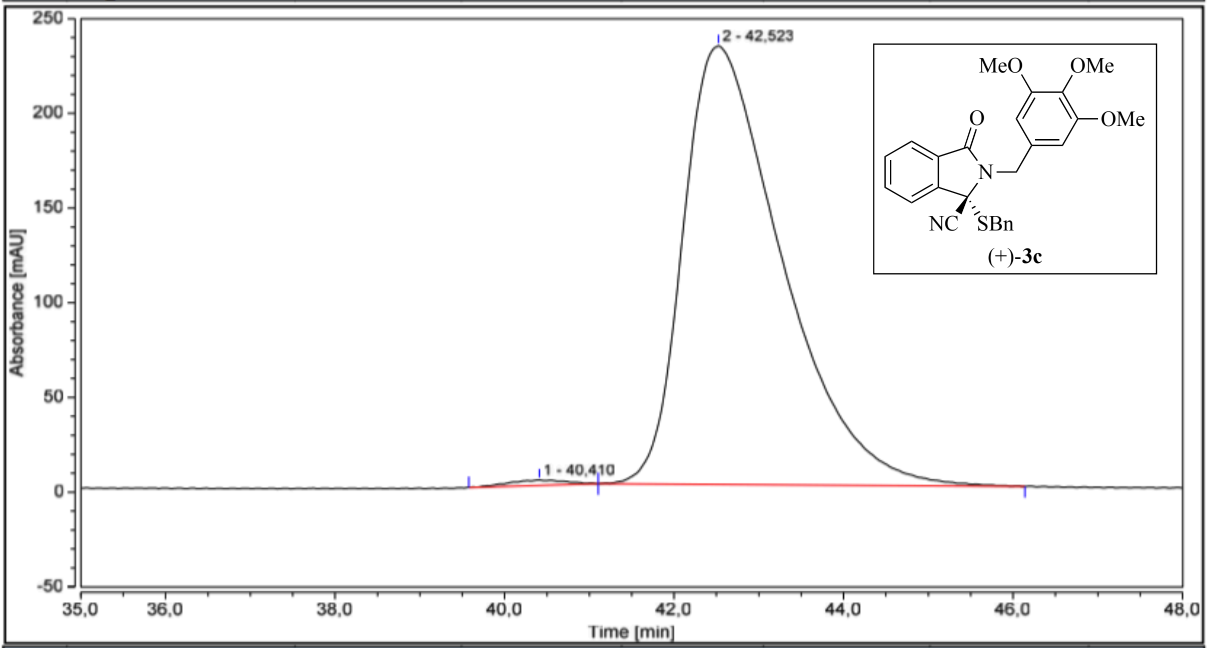

Integration Results

No.	Peak Name	Retention Time min	Area mAU*min	Height mAU	Relative Area %	Relative Height %	Amount n.a.
1		40,410	2,198	2,821	0,69	1,20	n.a.
2		42,523	314,120	231,583	99,31	98,80	n.a.
**Total:**			**316,318**	**234,403**	**100,00**	**100,00**	

Chromatogram

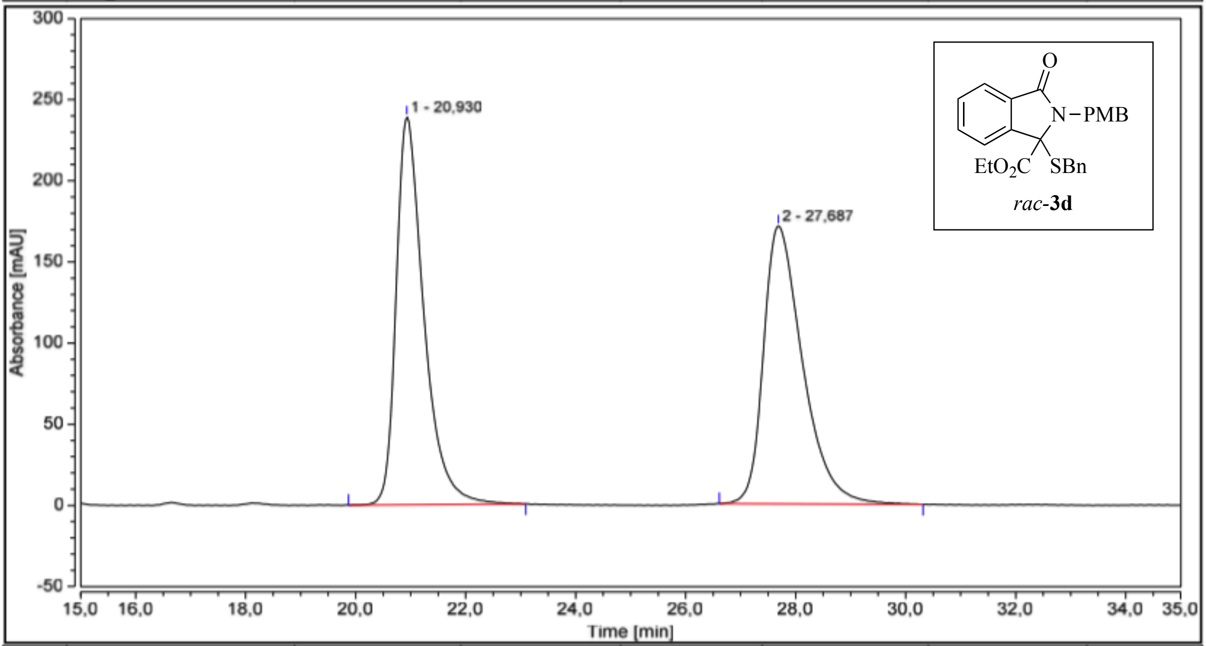

rac-**3d**

Integration Results

No.	Peak Name	Retention Time<br>min	Area<br>mAU*min	Height<br>mAU	Relative Area<br>%	Relative Height<br>%	Amount<br>n.a.
1		20,930	140,187	238,897	49,88	58,26	n.a.
2		27,687	140,843	171,142	50,12	41,74	n.a.
**Total:**			**281,030**	**410,039**	**100,00**	**100,00**	

Chromatogram

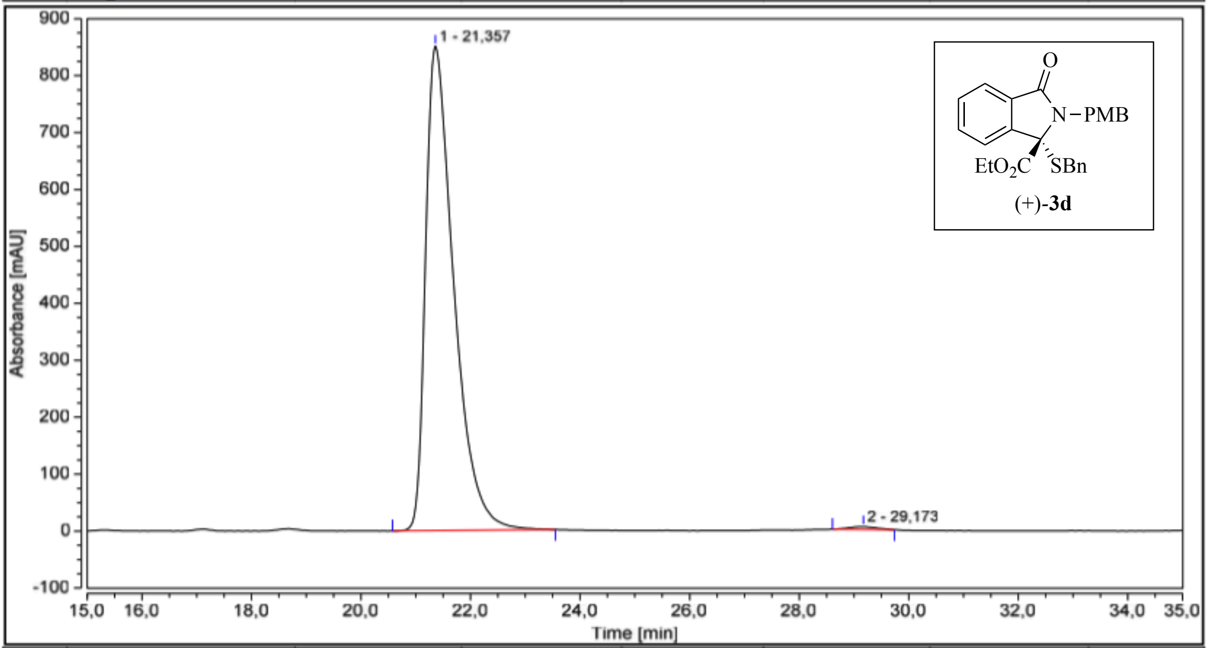

(+)-**3d**

Integration Results

No.	Peak Name	Retention Time<br>min	Area<br>mAU*min	Height<br>mAU	Relative Area<br>%	Relative Height<br>%	Amount<br>n.a.
1		21,357	508,632	850,428	99,44	99,44	n.a.
2		29,173	2,884	4,826	0,56	0,56	n.a.
**Total:**			**511,516**	**855,255**	**100,00**	**100,00**	

Chromatogram

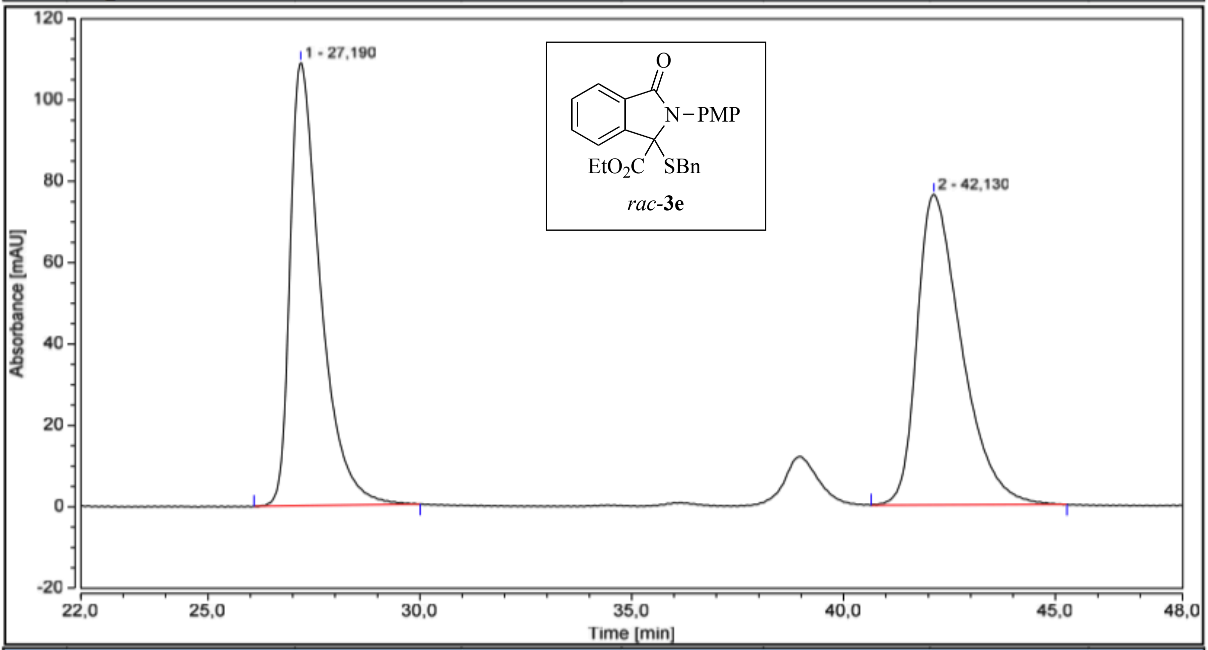

Integration Results

No.	Peak Name	Retention Time min	Area mAU*min	Height mAU	Relative Area %	Relative Height %	Amount n.a.
1		27,190	90,693	108,928	49,42	58,80	n.a.
2		42,130	92,821	76,335	50,58	41,20	n.a.
**Total:**			**183,514**	**185,263**	**100,00**	**100,00**	

Chromatogram

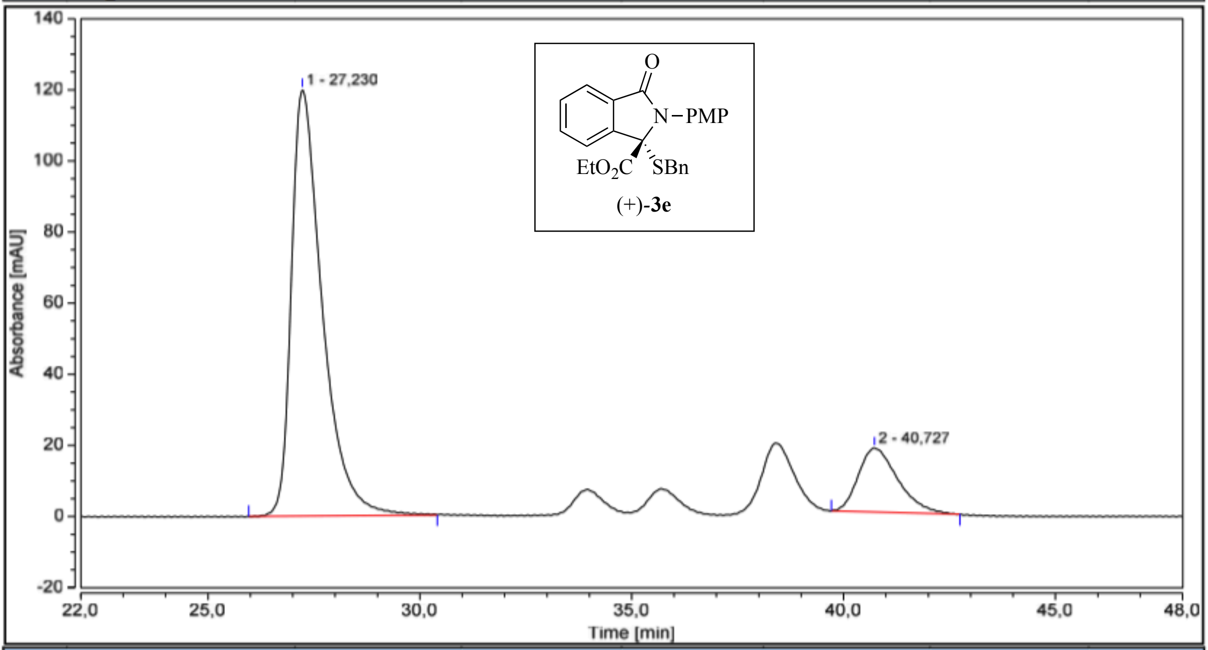

Integration Results

No.	Peak Name	Retention Time min	Area mAU*min	Height mAU	Relative Area %	Relative Height %	Amount n.a.
1		27,230	101,266	119,849	83,88	86,92	n.a.
2		40,727	19,456	18,034	16,12	13,08	n.a.
**Total:**			**120,722**	**137,883**	**100,00**	**100,00**	

Chromatogram

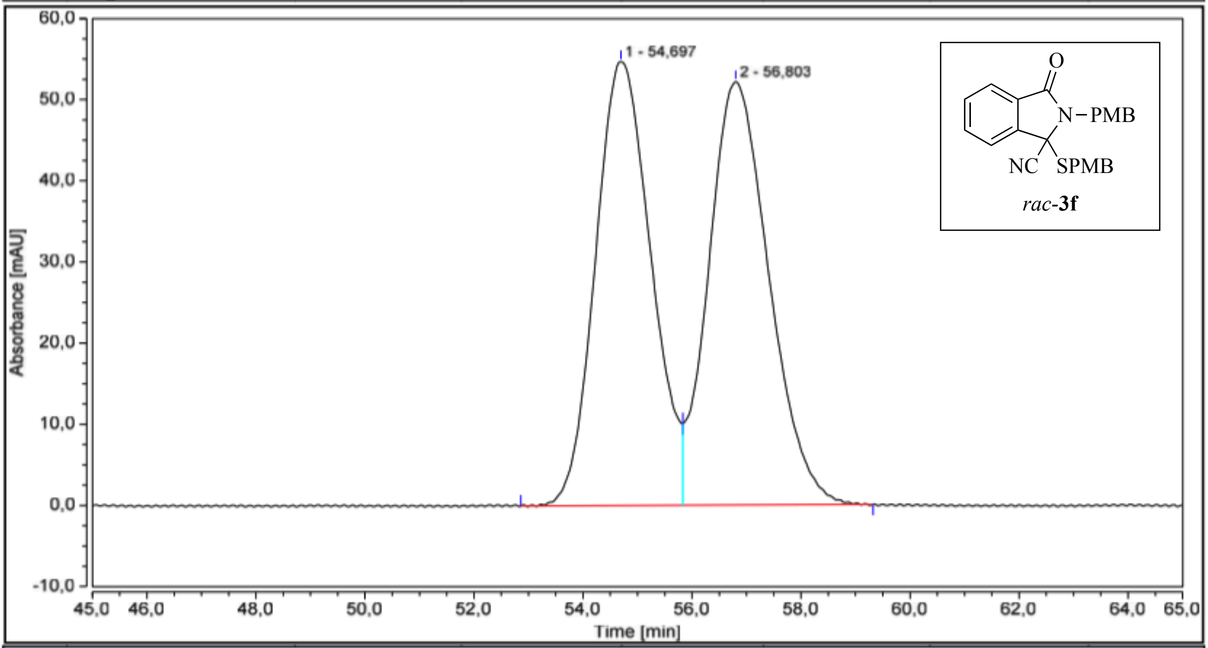

Integration Results

No.	Peak Name	Retention Time min	Area mAU*min	Height mAU	Relative Area %	Relative Height %	Amount n.a.
1		54,697	64,660	54,709	49,07	51,19	n.a.
2		56,803	67,109	52,165	50,93	48,81	n.a.
**Total:**			**131,769**	**106,874**	**100,00**	**100,00**	

Chromatogram

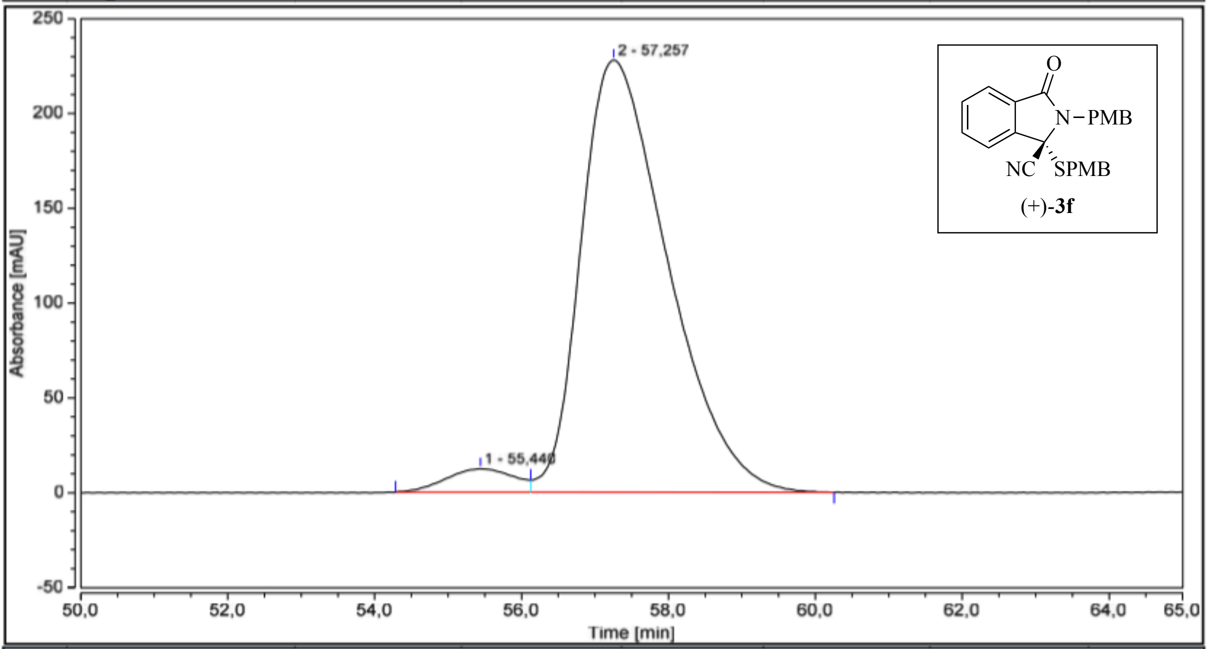

Integration Results

No.	Peak Name	Retention Time min	Area mAU*min	Height mAU	Relative Area %	Relative Height %	Amount n.a.
1		55,440	13,172	12,356	4,05	5,15	n.a.
2		57,257	312,088	227,725	95,95	94,85	n.a.
**Total:**			**325,260**	**240,080**	**100,00**	**100,00**	

Chromatogram

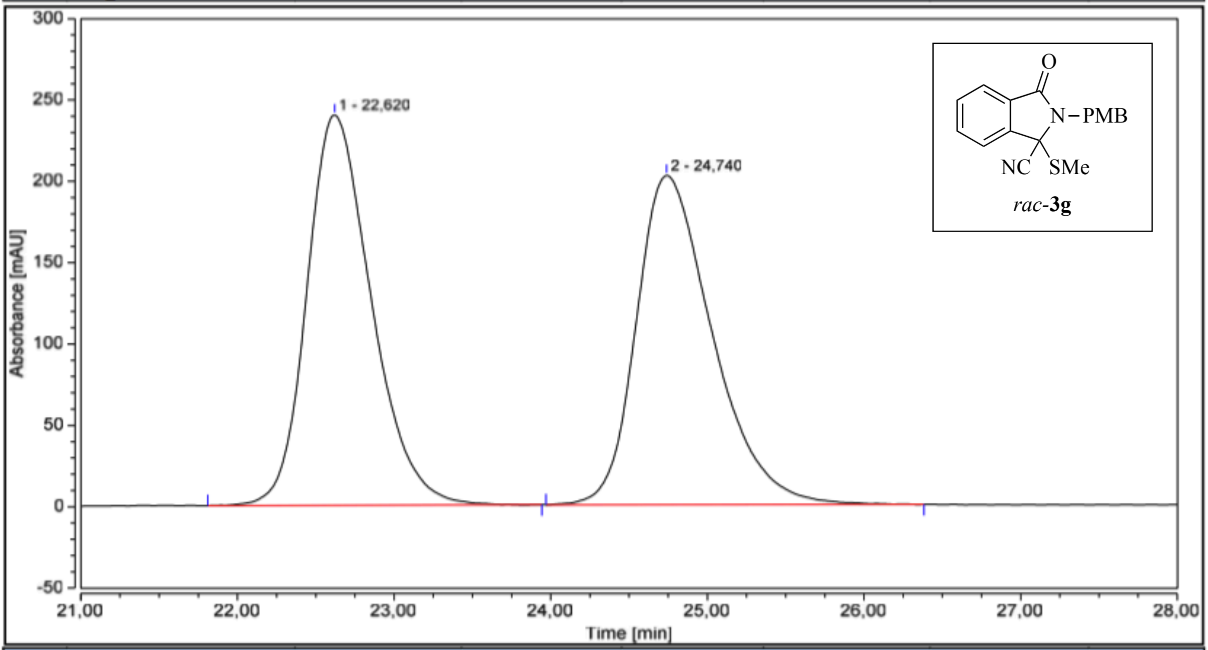

Integration Results

No.	Peak Name	Retention Time min	Area mAU*min	Height mAU	Relative Area %	Relative Height %	Amount n.a.
1		22,620	114,445	239,901	50,07	54,26	n.a.
2		24,740	114,135	202,260	49,93	45,74	n.a.
**Total:**			**228,579**	**442,161**	**100,00**	**100,00**	

Chromatogram

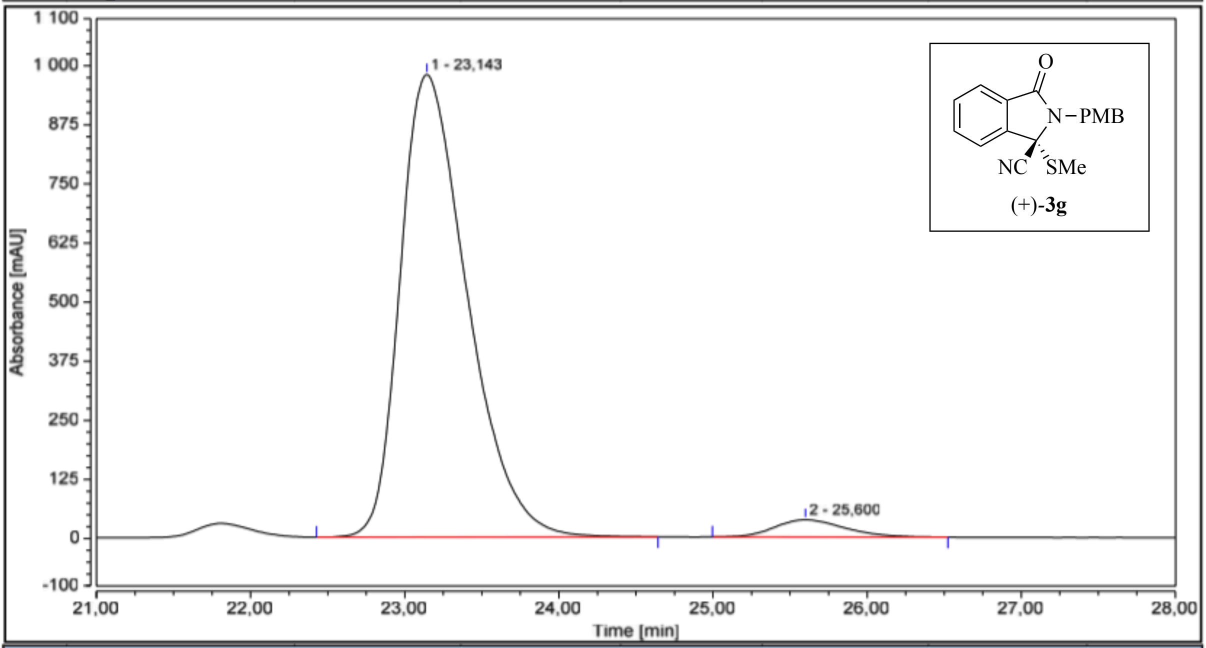

Integration Results

No.	Peak Name	Retention Time min	Area mAU*min	Height mAU	Relative Area %	Relative Height %	Amount n.a.
1		23,143	492,756	978,222	96,23	96,39	n.a.
2		25,600	19,291	36,607	3,77	3,61	n.a.
**Total:**			**512,047**	**1014,830**	**100,00**	**100,00**	

Chromatogram

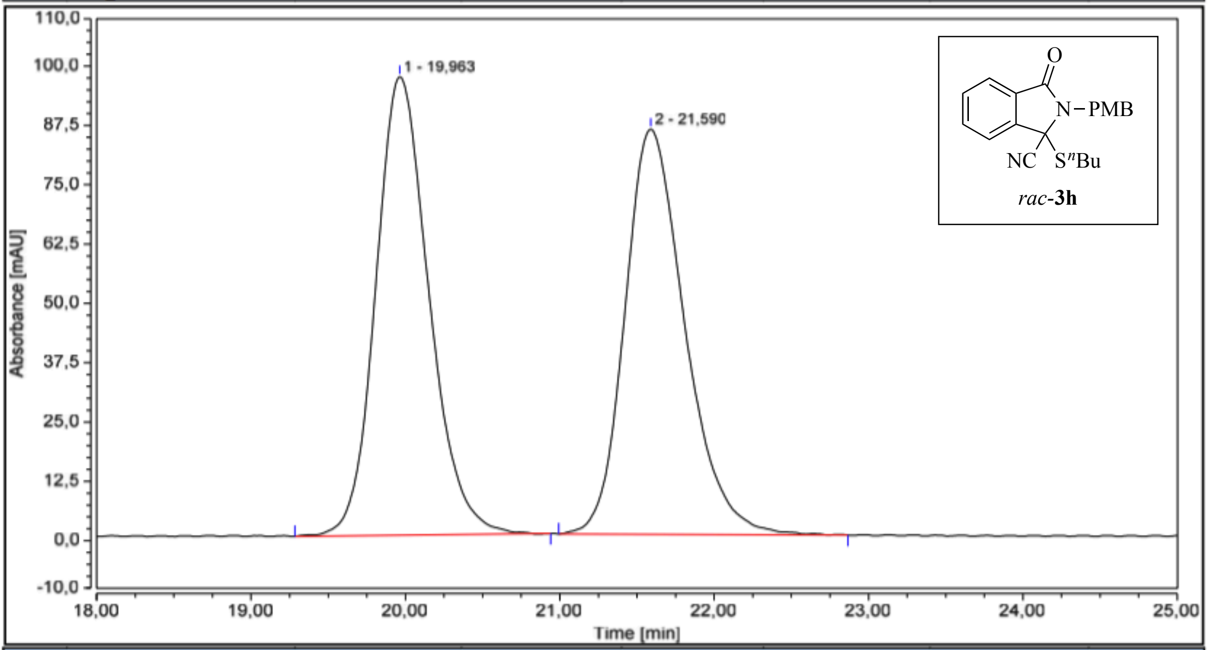

Integration Results

No.	Peak Name	Retention Time min	Area mAU*min	Height mAU	Relative Area %	Relative Height %	Amount n.a.
1		19,963	38,568	96,613	50,08	53,08	n.a.
2		21,590	38,451	85,390	49,92	46,92	n.a.
**Total:**			**77,019**	**182,003**	**100,00**	**100,00**	

Chromatogram

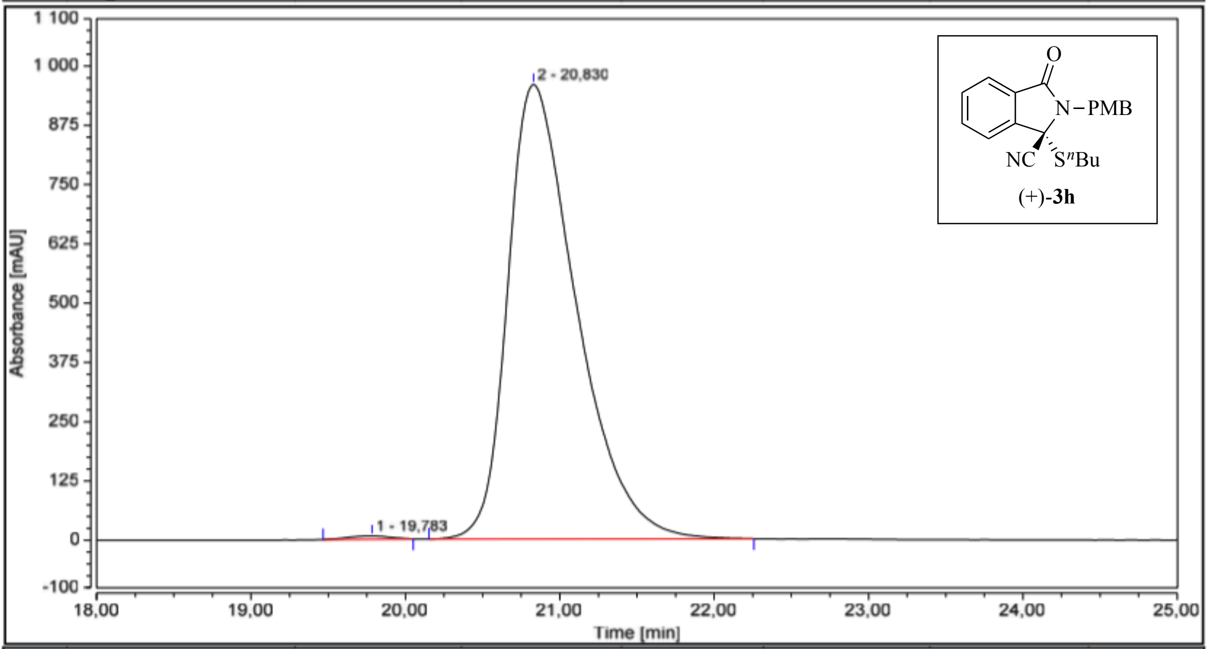

Integration Results

No.	Peak Name	Retention Time min	Area mAU*min	Height mAU	Relative Area %	Relative Height %	Amount n.a.
1		19,783	2,178	6,823	0,44	0,71	n.a.
2		20,830	493,555	958,352	99,56	99,29	n.a.
**Total:**			**495,732**	**965,175**	**100,00**	**100,00**	

Chromatogram

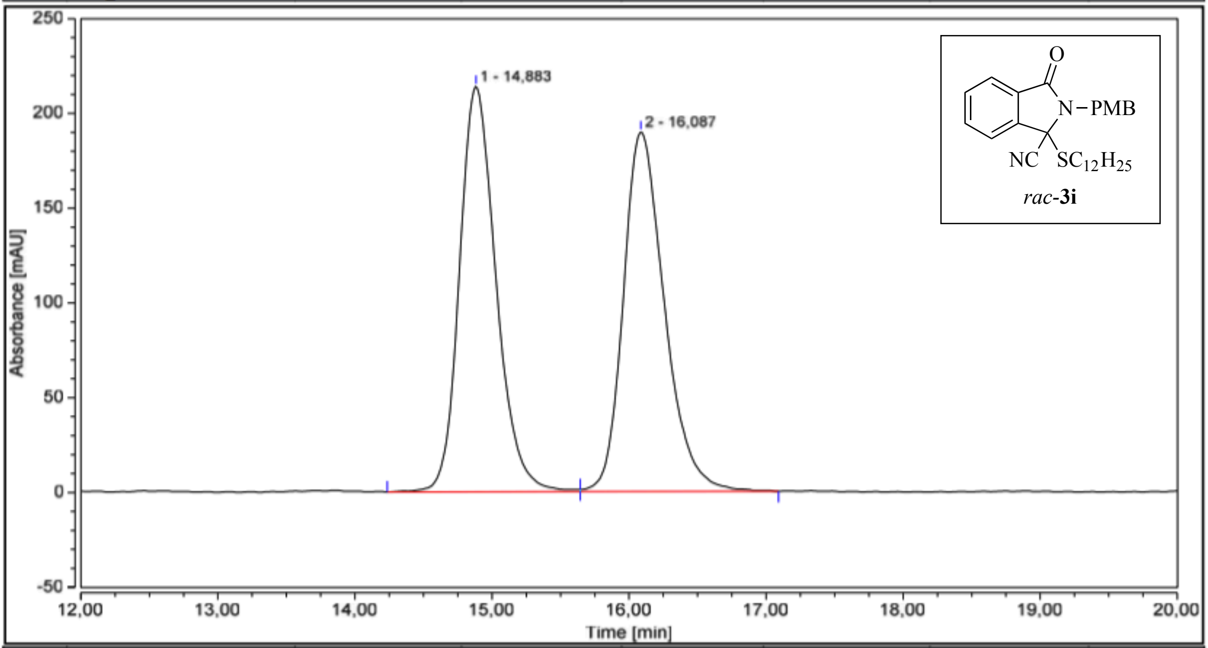

Integration Results

No.	Peak Name	Retention Time min	Area mAU*min	Height mAU	Relative Area %	Relative Height %	Amount n.a.
1		14,883	66,131	213,874	50,05	53,01	n.a.
2		16,087	65,995	189,572	49,95	46,99	n.a.
**Total:**			**132,126**	**403,445**	**100,00**	**100,00**	

Chromatogram

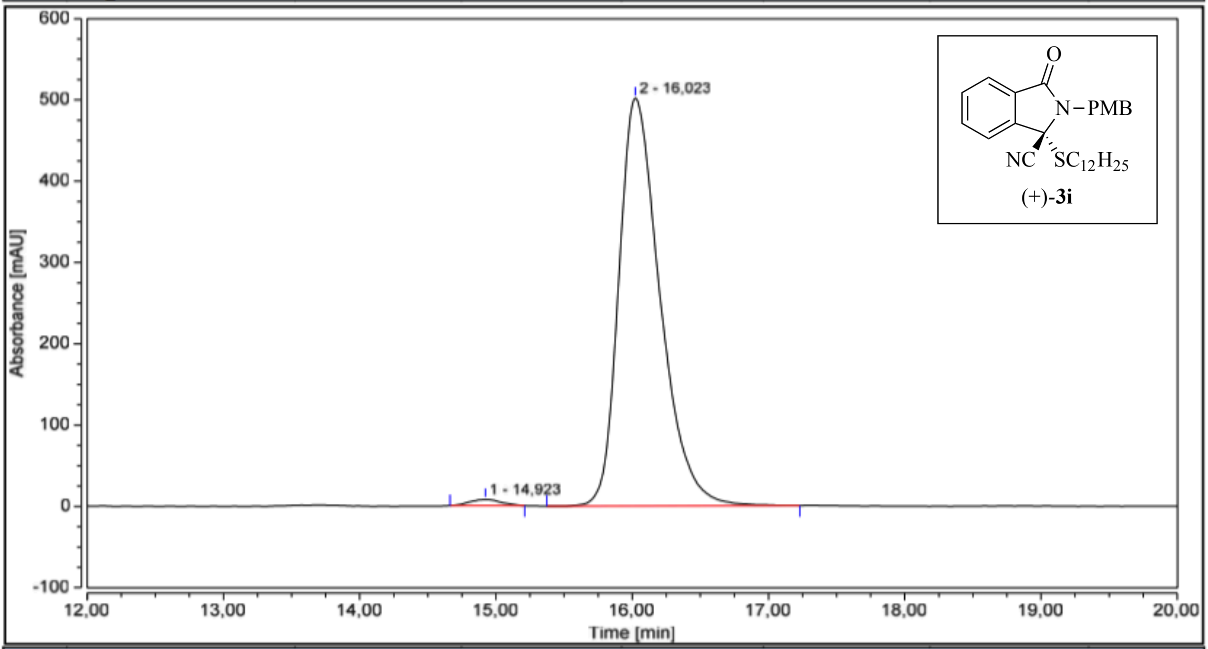

Integration Results

No.	Peak Name	Retention Time min	Area mAU*min	Height mAU	Relative Area %	Relative Height %	Amount n.a.
1		14,923	2,024	7,598	1,14	1,49	n.a.
2		16,023	176,102	501,841	98,86	98,51	n.a.
**Total:**			**178,126**	**509,439**	**100,00**	**100,00**	

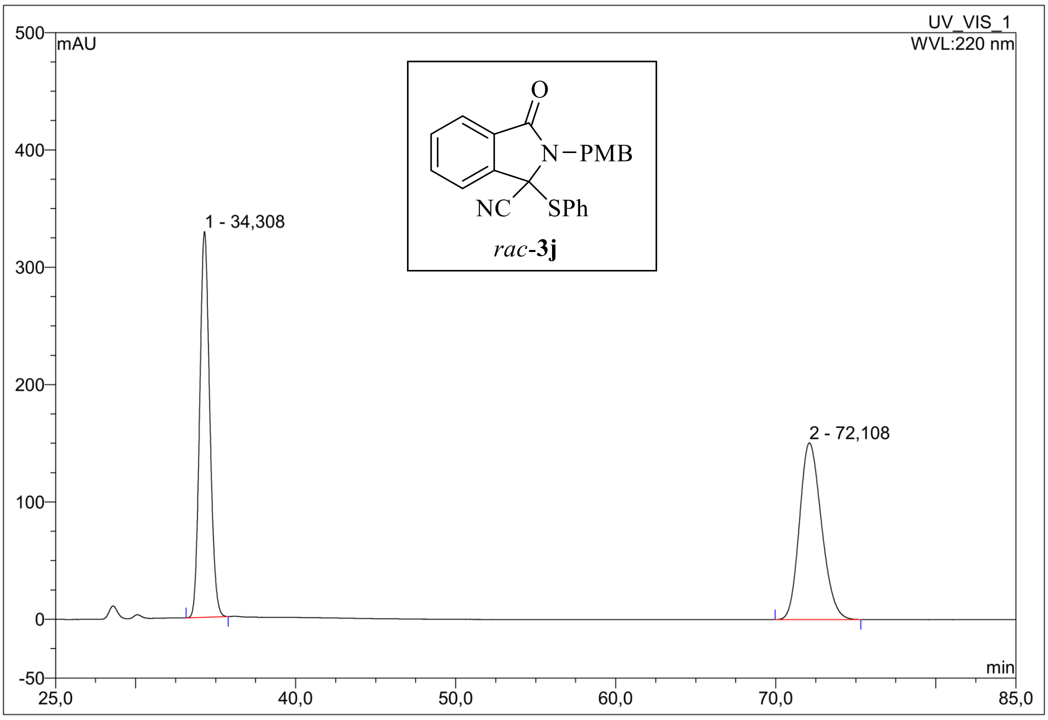

No.	Ret.Time<br>min	Peak Name	Height<br>mAU	Area<br>mAU*min	Rel.Area<br>%	Amount	Type
1	34,31	n.a.	328,732	239,197	49,89	n.a.	BMB
2	72,11	n.a.	150,638	240,243	50,11	n.a.	BMB
Total:			479,370	479,440	100,00	0,000	

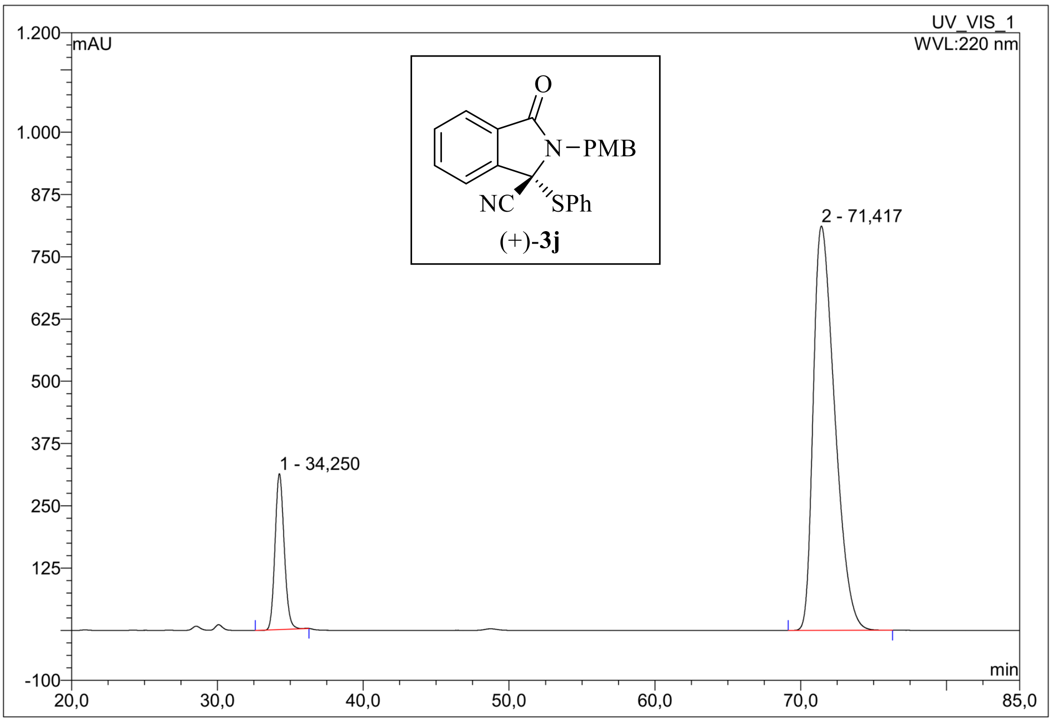

No.	Ret.Time<br>min	Peak Name	Height<br>mAU	Area<br>mAU*min	Rel.Area<br>%	Amount	Type
1	34,25	n.a.	312,818	227,522	14,09	n.a.	BMB*
2	71,42	n.a.	811,718	1387,669	85,91	n.a.	BMB*
Total:			1124,535	1615,191	100,00	0,000	

Chromatogram

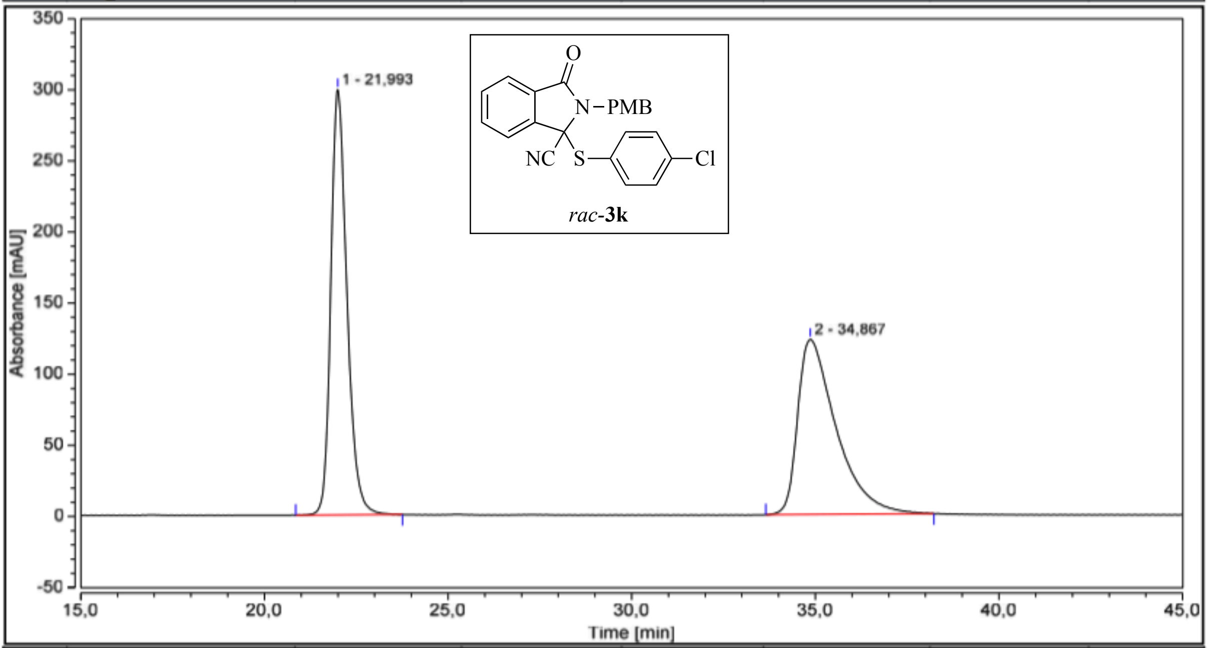

Integration Results

No.	Peak Name	Retention Time min	Area mAU*min	Height mAU	Relative Area %	Relative Height %	Amount n.a.
1		21,993	152,117	299,162	50,51	70,86	n.a.
2		34,867	149,027	123,028	49,49	29,14	n.a.
**Total:**			**301,145**	**422,190**	**100,00**	**100,00**	

Chromatogram

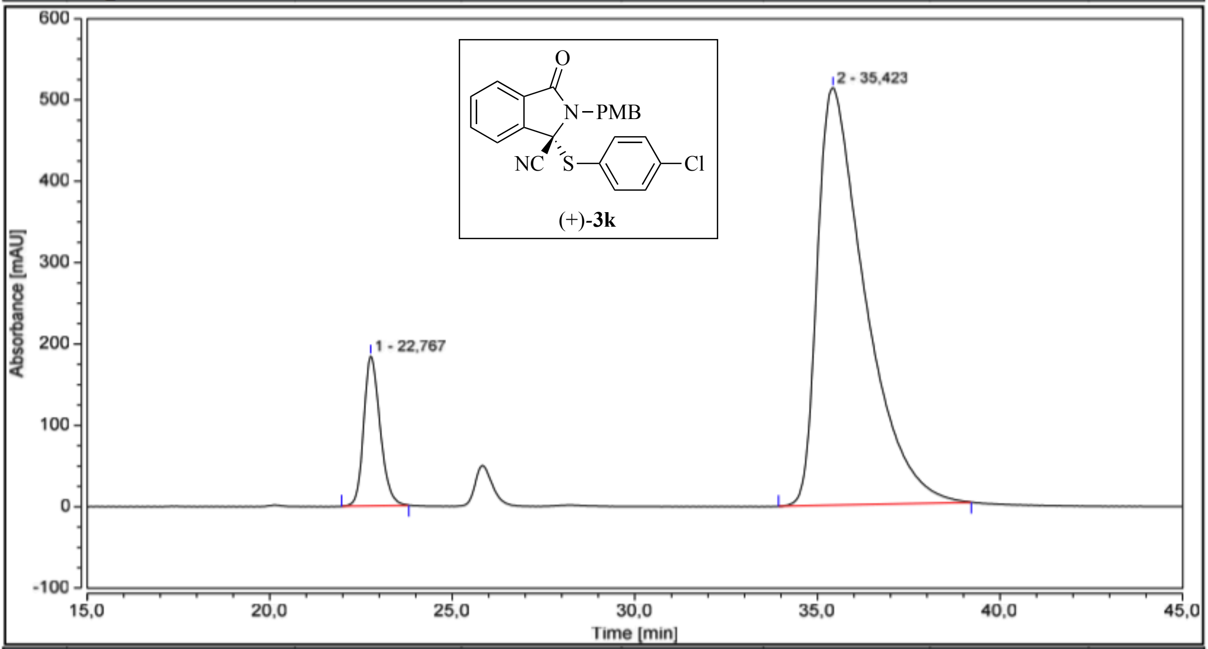

Integration Results

No.	Peak Name	Retention Time min	Area mAU*min	Height mAU	Relative Area %	Relative Height %	Amount n.a.
1		22,767	96,139	184,240	11,01	26,43	n.a.
2		35,423	776,723	512,925	88,99	73,57	n.a.
**Total:**			**872,862**	**697,166**	**100,00**	**100,00**	

Chromatogram

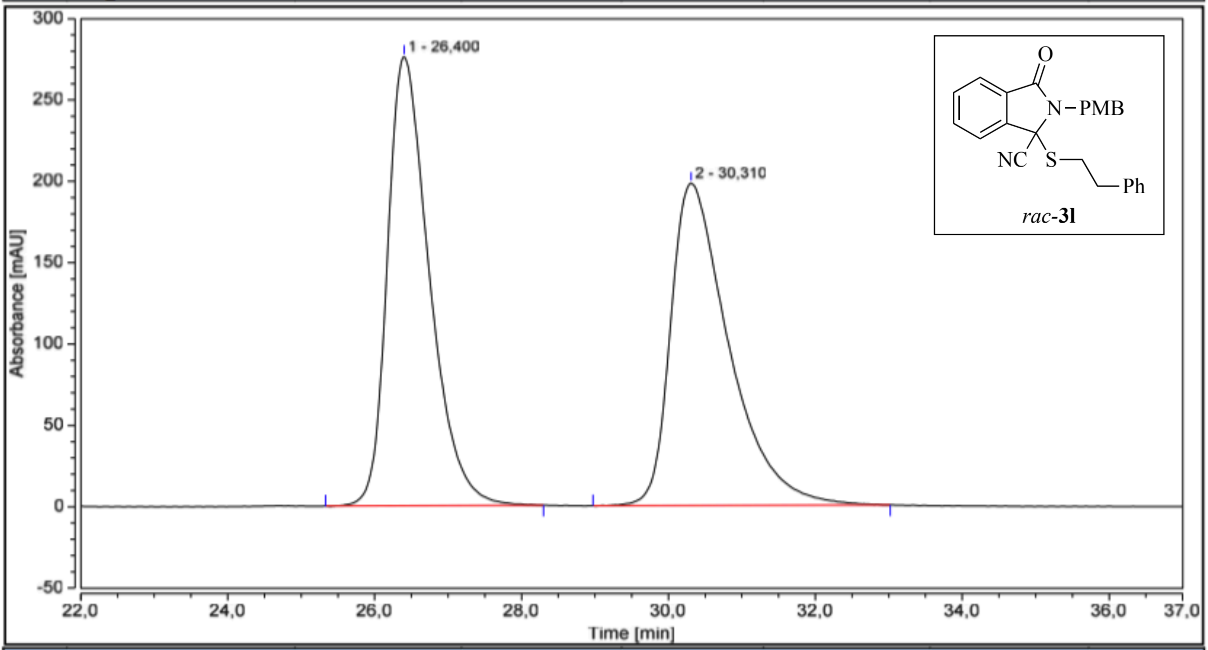

Integration Results

No.	Peak Name	Retention Time min	Area mAU*min	Height mAU	Relative Area %	Relative Height %	Amount n.a.
1		26,400	182,486	276,050	50,04	58,22	n.a.
2		30,310	182,229	198,070	49,96	41,78	n.a.
**Total:**			**364,715**	**474,120**	**100,00**	**100,00**	

Chromatogram

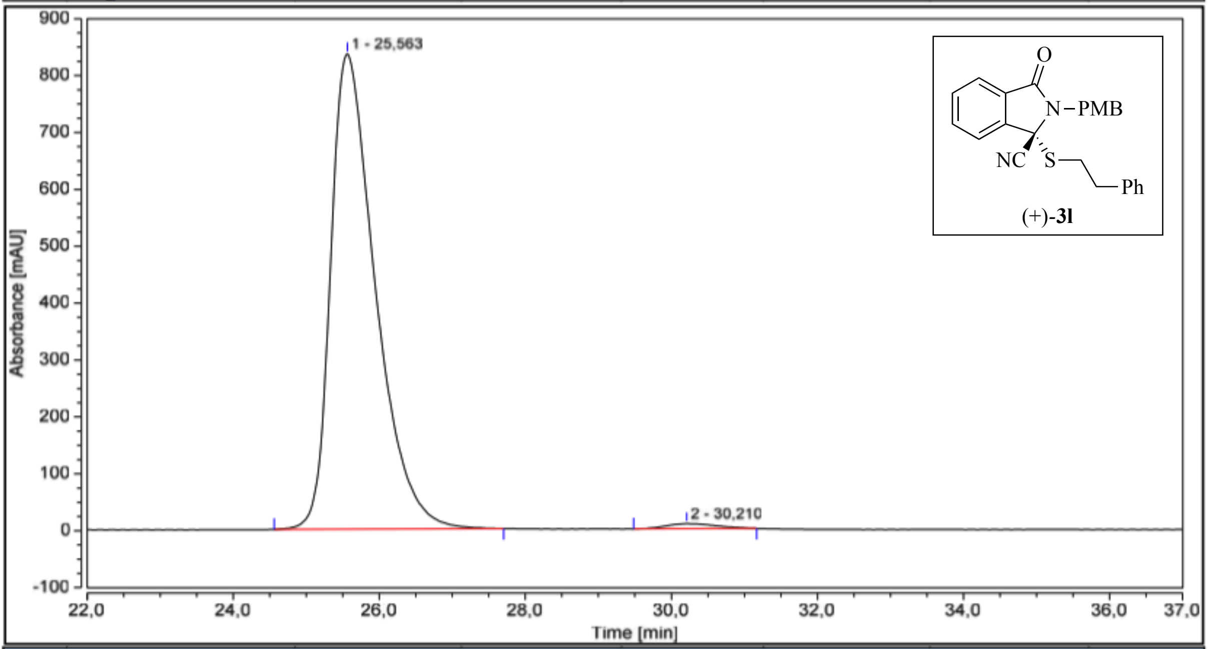

Integration Results

No.	Peak Name	Retention Time min	Area mAU*min	Height mAU	Relative Area %	Relative Height %	Amount n.a.
1		25,563	591,517	835,222	98,81	98,95	n.a.
2		30,210	7,103	8,903	1,19	1,05	n.a.
**Total:**			**598,620**	**844,124**	**100,00**	**100,00**	

Chromatogram

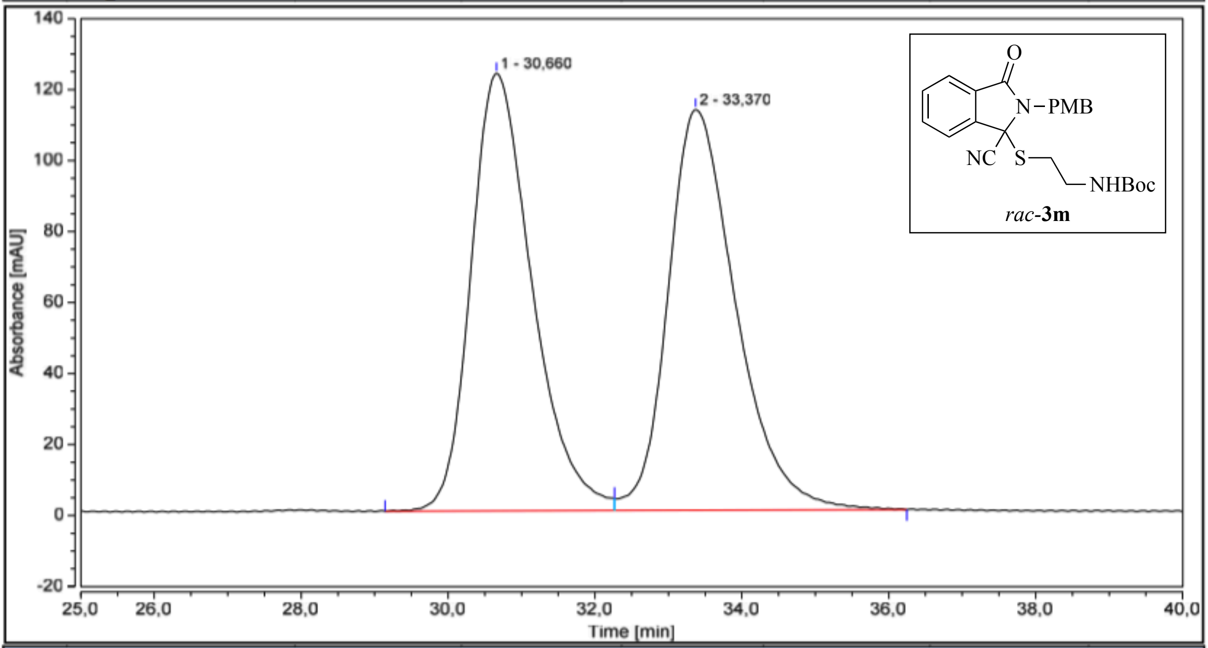

Integration Results

No.	Peak Name	Retention Time min	Area mAU*min	Height mAU	Relative Area %	Relative Height %	Amount n.a.
1		30,660	118,699	123,211	49,59	52,20	n.a.
2		33,370	120,653	112,847	50,41	47,80	n.a.
**Total:**			**239,352**	**236,059**	**100,00**	**100,00**	

Chromatogram

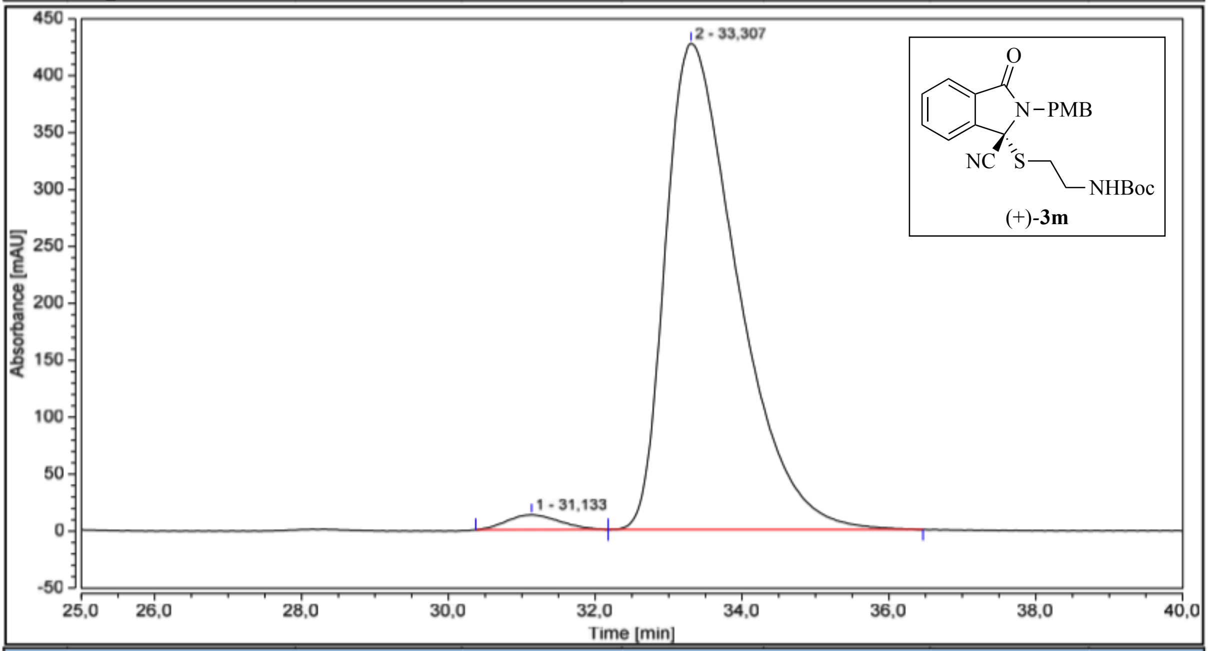

Integration Results

No.	Peak Name	Retention Time min	Area mAU*min	Height mAU	Relative Area %	Relative Height %	Amount n.a.
1		31,133	10,855	13,025	2,19	2,96	n.a.
2		33,307	483,918	426,752	97,81	97,04	n.a.
**Total:**			**494,773**	**439,777**	**100,00**	**100,00**	

Chromatogram

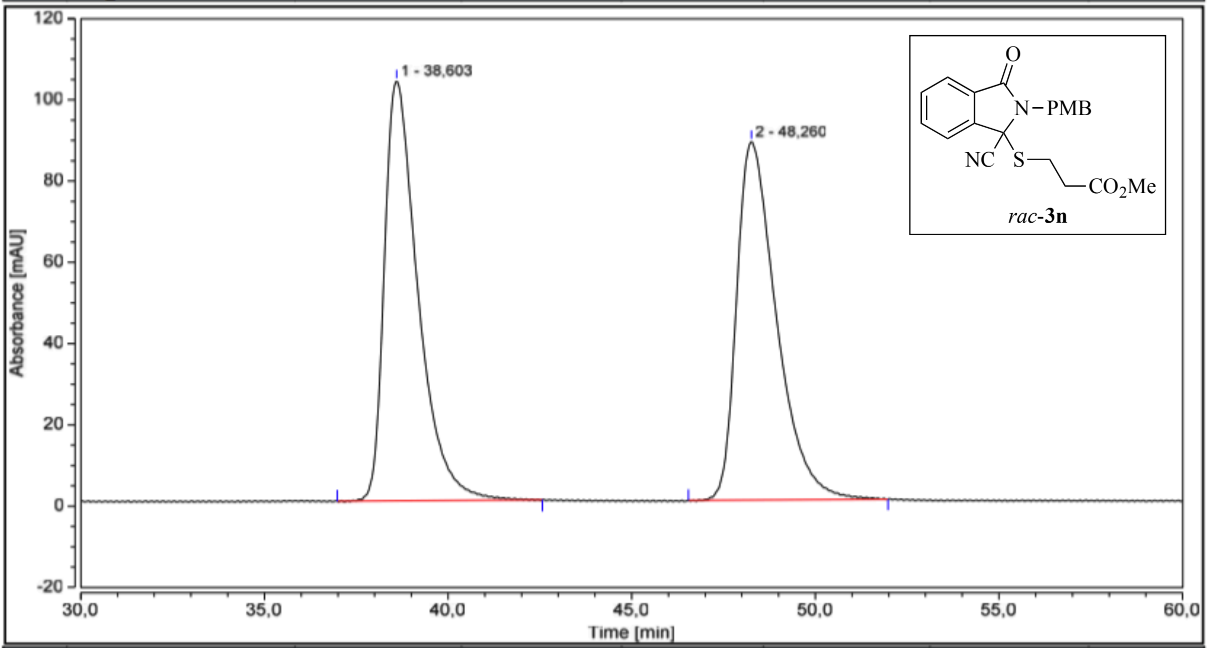

Integration Results

No.	Peak Name	Retention Time min	Area mAU*min	Height mAU	Relative Area %	Relative Height %	Amount n.a.
1		38,603	109,798	103,373	49,75	53,95	n.a.
2		48,260	110,883	88,227	50,25	46,05	n.a.
**Total:**			**220,681**	**191,600**	**100,00**	**100,00**	

Chromatogram

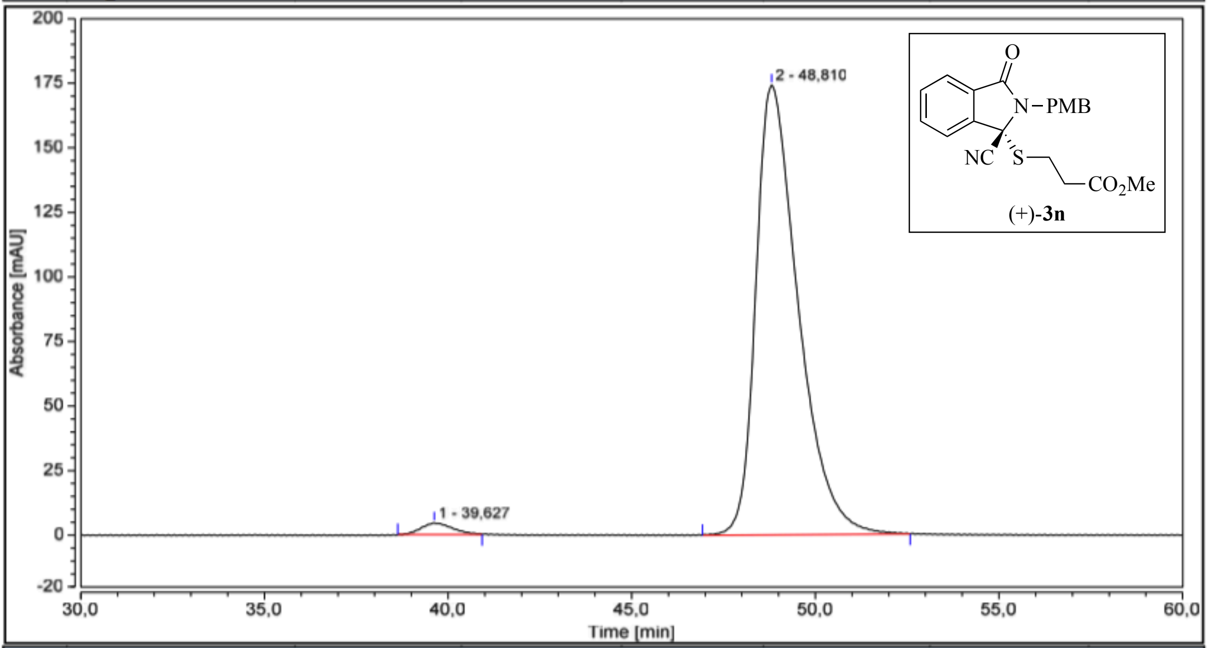

Integration Results

No.	Peak Name	Retention Time min	Area mAU*min	Height mAU	Relative Area %	Relative Height %	Amount n.a.
1		39,627	4,430	4,476	1,87	2,51	n.a.
2		48,810	233,103	174,093	98,13	97,49	n.a.
**Total:**			**237,534**	**178,568**	**100,00**	**100,00**	

Chromatogram

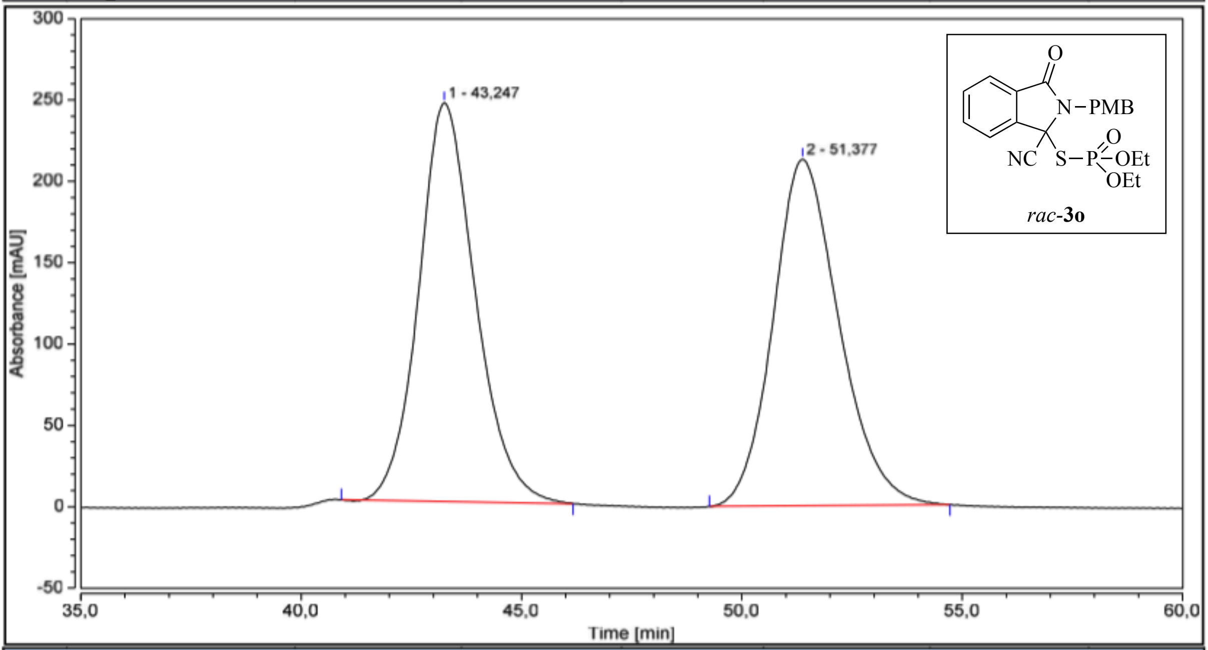

Integration Results

No.	Peak Name	Retention Time min	Area mAU*min	Height mAU	Relative Area %	Relative Height %	Amount n.a.
1		43,247	365,326	245,094	49,72	53,50	n.a.
2		51,377	369,443	212,984	50,28	46,50	n.a.
**Total:**			**734,769**	**458,078**	**100,00**	**100,00**	

Chromatogram

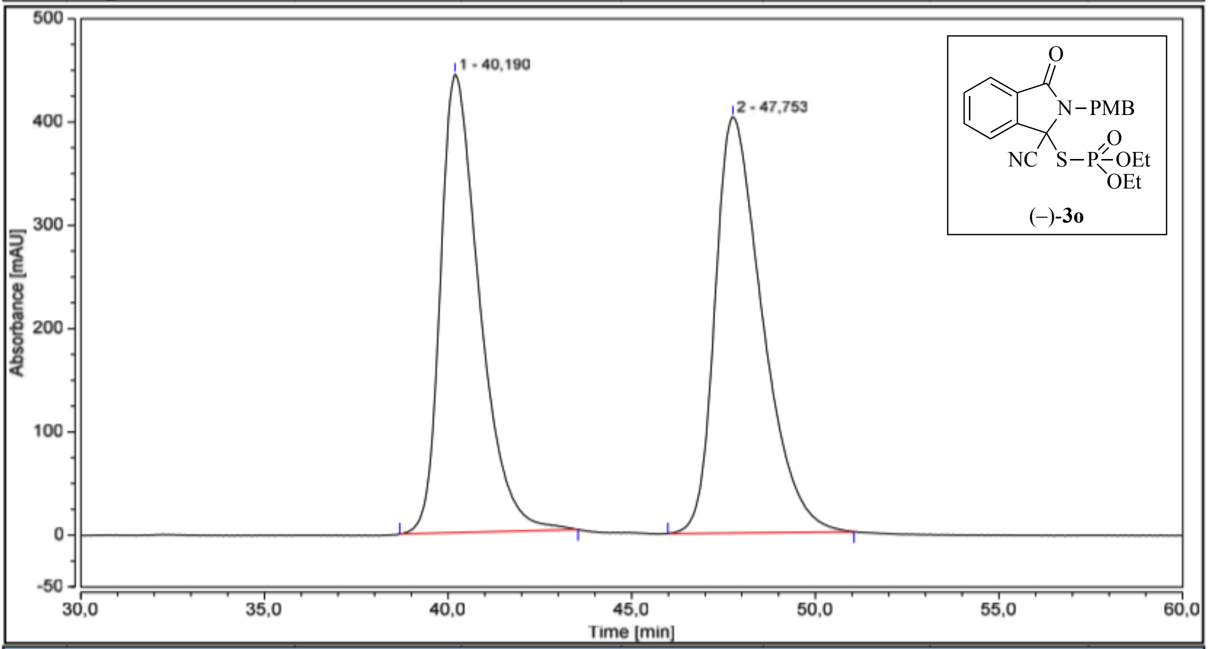

Integration Results

No.	Peak Name	Retention Time min	Area mAU*min	Height mAU	Relative Area %	Relative Height %	Amount n.a.
1		40,190	548,155	443,488	47,70	52,42	n.a.
2		47,753	601,103	402,588	52,30	47,58	n.a.
**Total:**			**1149,258**	**846,077**	**100,00**	**100,00**	

Chromatogram

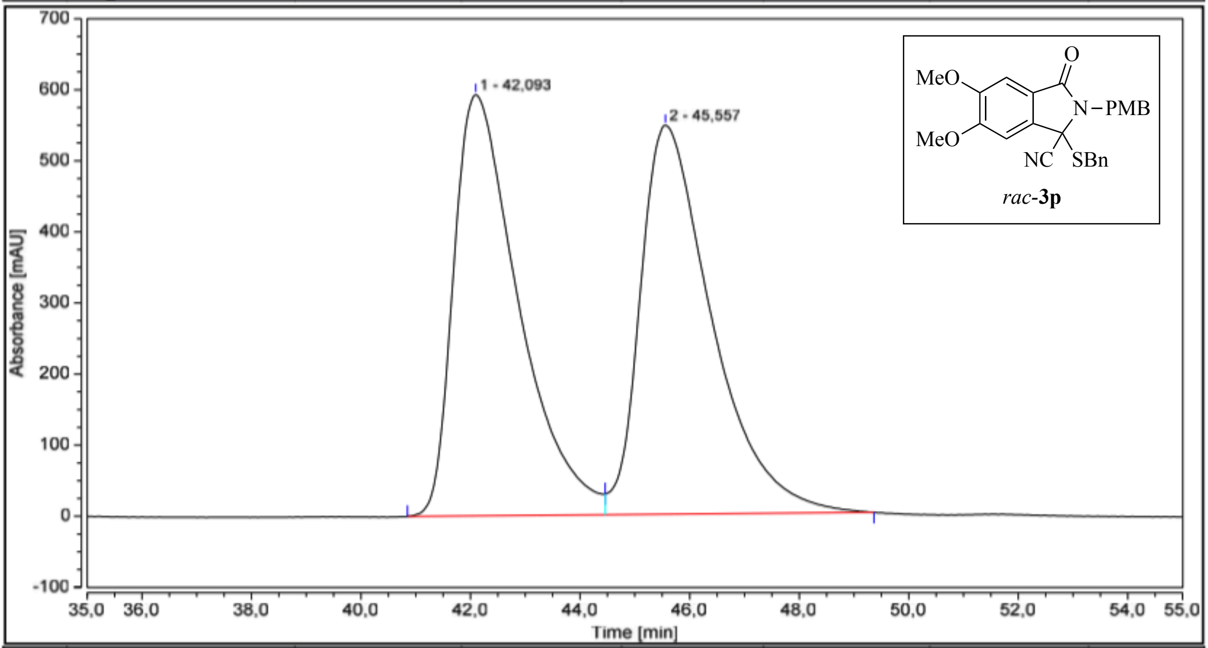

Integration Results

No.	Peak Name	Retention Time min	Area mAU*min	Height mAU	Relative Area %	Relative Height %	Amount n.a.
1		42,093	813,501	592,052	49,53	51,98	n.a.
2		45,557	829,023	546,879	50,47	48,02	n.a.
**Total:**			**1642,524**	**1138,931**	**100,00**	**100,00**	

Chromatogram

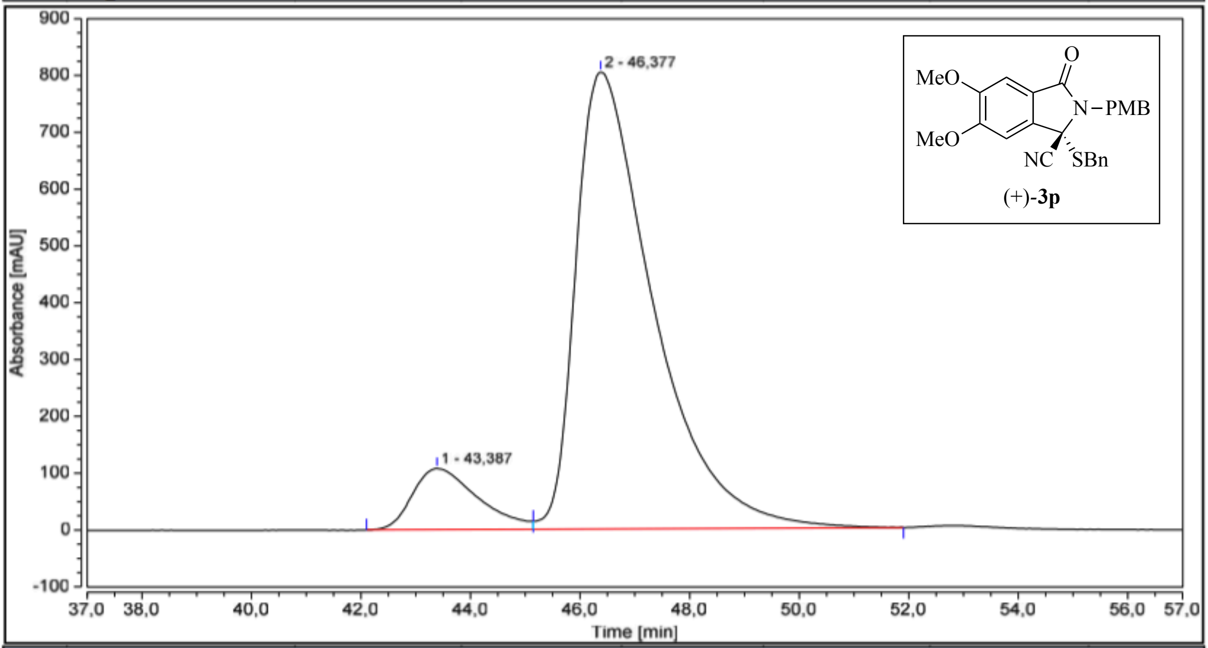

Integration Results

No.	Peak Name	Retention Time min	Area mAU*min	Height mAU	Relative Area %	Relative Height %	Amount n.a.
1		43,387	148,218	107,600	10,10	11,81	n.a.
2		46,377	1319,742	803,692	89,90	88,19	n.a.
**Total:**			**1467,960**	**911,292**	**100,00**	**100,00**	

Chromatogram

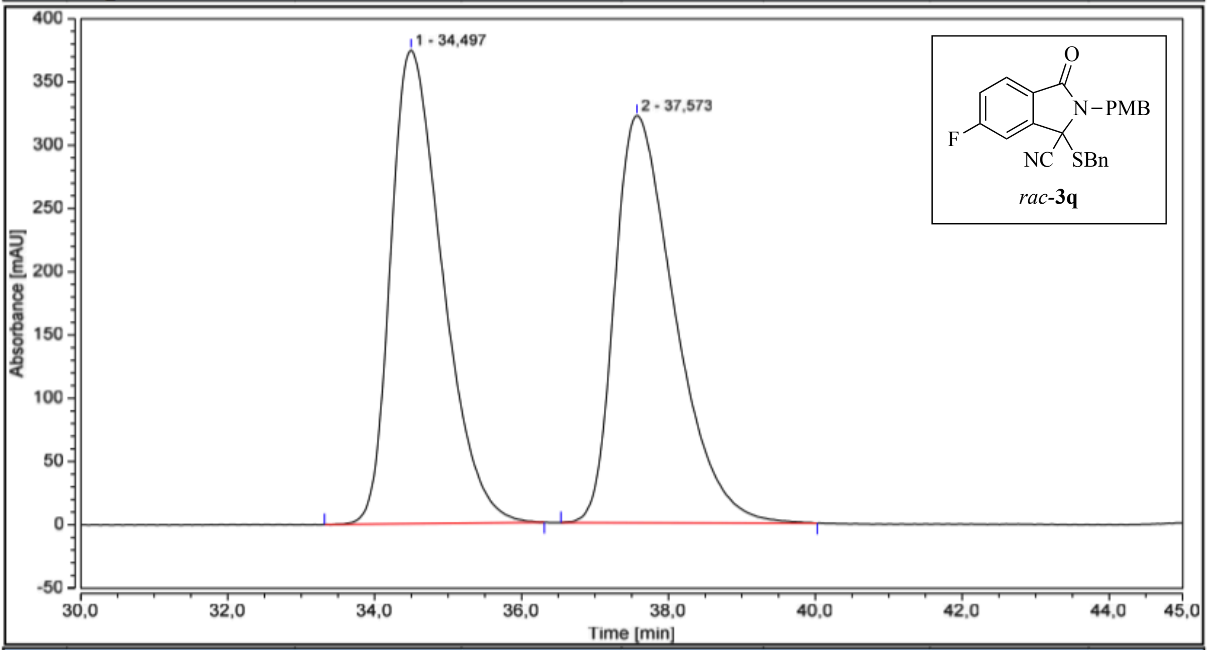

Integration Results

No.	Peak Name	Retention Time min	Area mAU*min	Height mAU	Relative Area %	Relative Height %	Amount n.a.
1		34,497	305,333	374,229	49,96	53,77	n.a.
2		37,573	305,796	321,789	50,04	46,23	n.a.
**Total:**			**611,129**	**696,018**	**100,00**	**100,00**	

Chromatogram

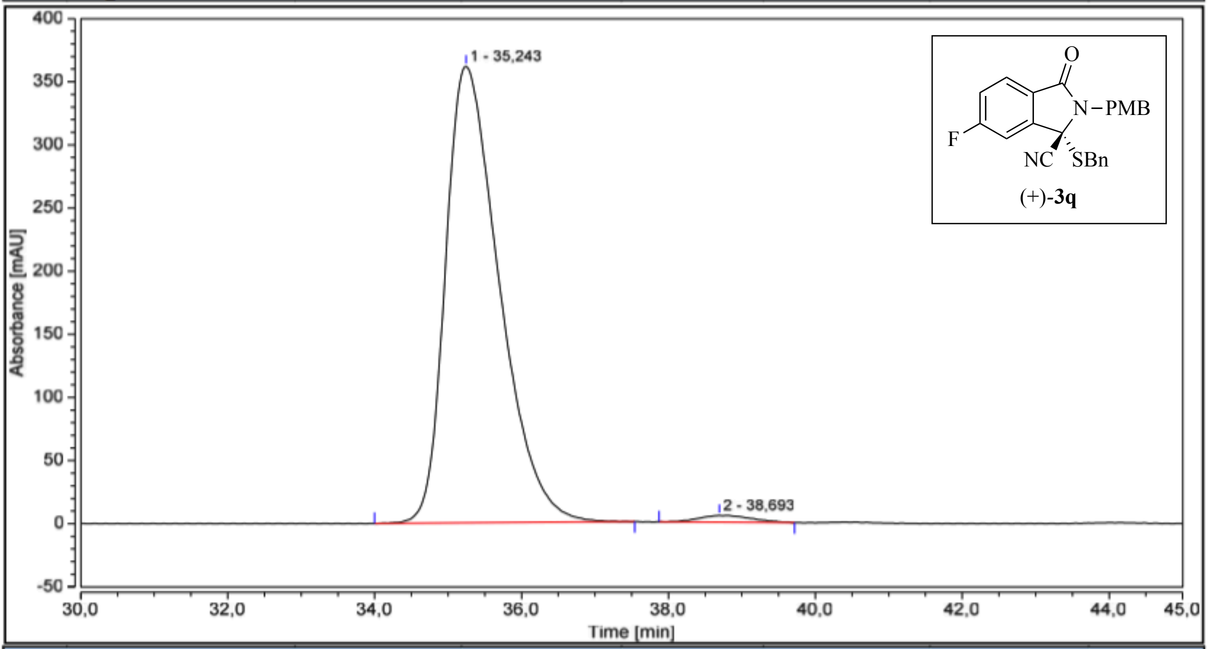

Integration Results

No.	Peak Name	Retention Time min	Area mAU*min	Height mAU	Relative Area %	Relative Height %	Amount n.a.
1		35,243	306,264	361,597	98,59	98,55	n.a.
2		38,693	4,367	5,334	1,41	1,45	n.a.
**Total:**			**310,630**	**366,931**	**100,00**	**100,00**	

Chromatogram

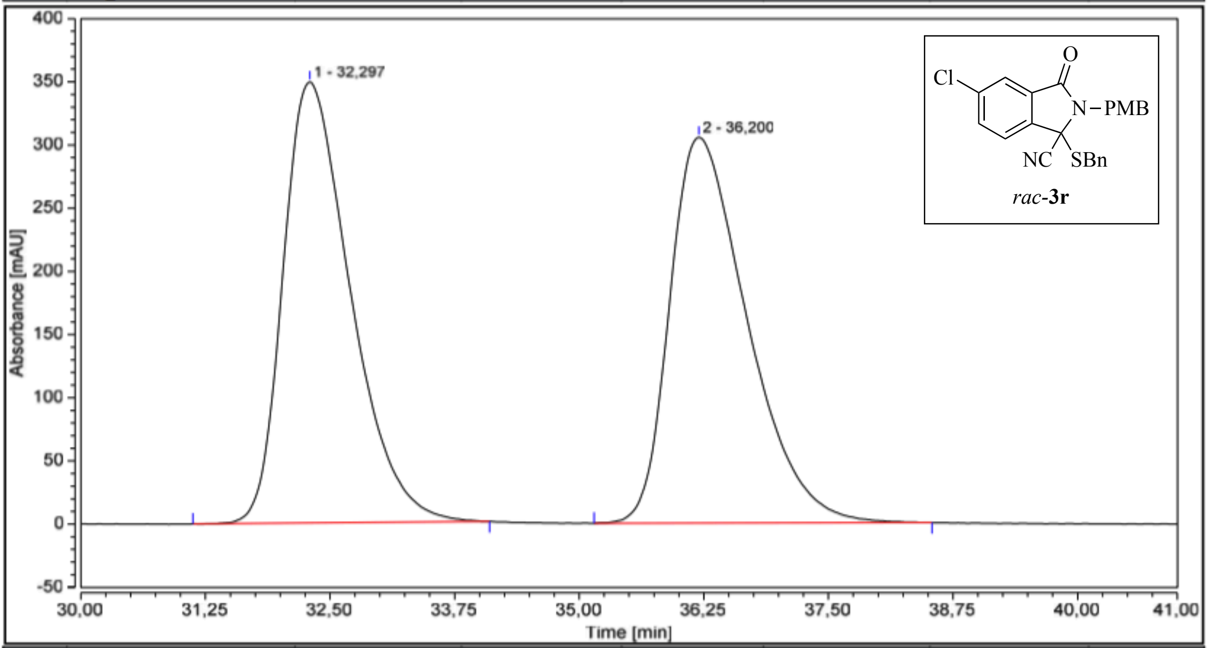

Integration Results

No.	Peak Name	Retention Time<br>min	Area<br>mAU*min	Height<br>mAU	Relative Area<br>%	Relative Height<br>%	Amount<br>n.a.
1		32,297	273,838	348,950	49,83	53,36	n.a.
2		36,200	275,658	305,057	50,17	46,64	n.a.
**Total:**			**549,497**	**654,008**	**100,00**	**100,00**	

Chromatogram

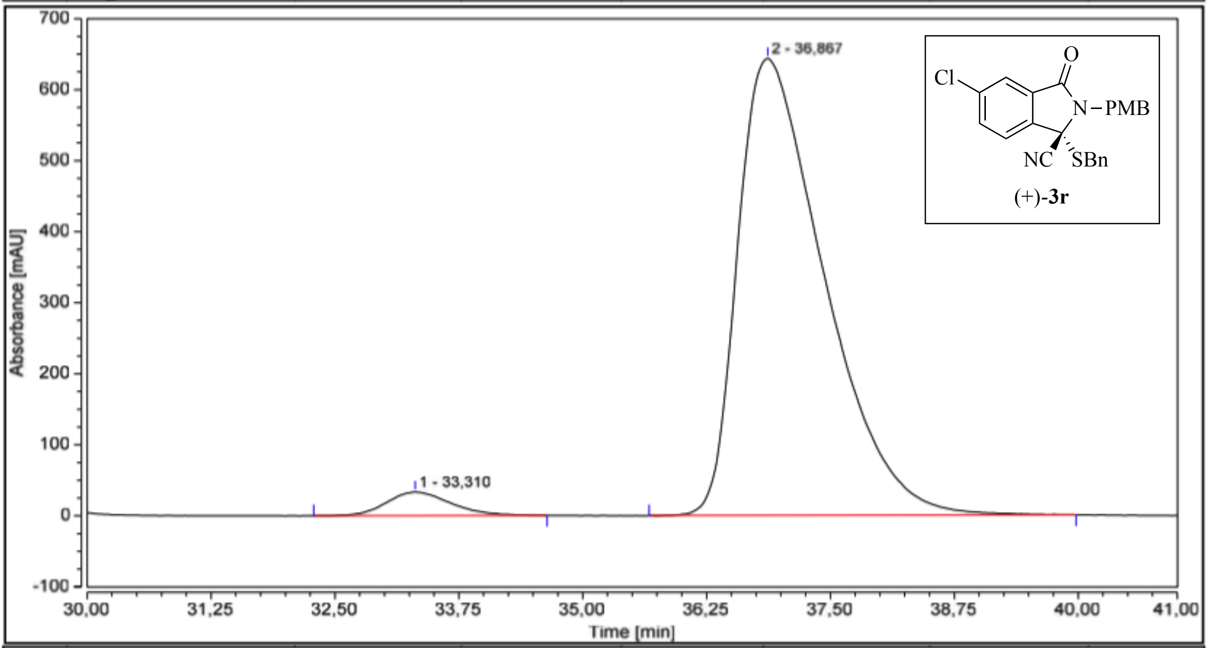

Integration Results

No.	Peak Name	Retention Time<br>min	Area<br>mAU*min	Height<br>mAU	Relative Area<br>%	Relative Height<br>%	Amount<br>n.a.
1		33,310	25,102	33,249	3,72	4,91	n.a.
2		36,867	650,543	643,261	96,28	95,09	n.a.
**Total:**			**675,646**	**676,510**	**100,00**	**100,00**	

Chromatogram

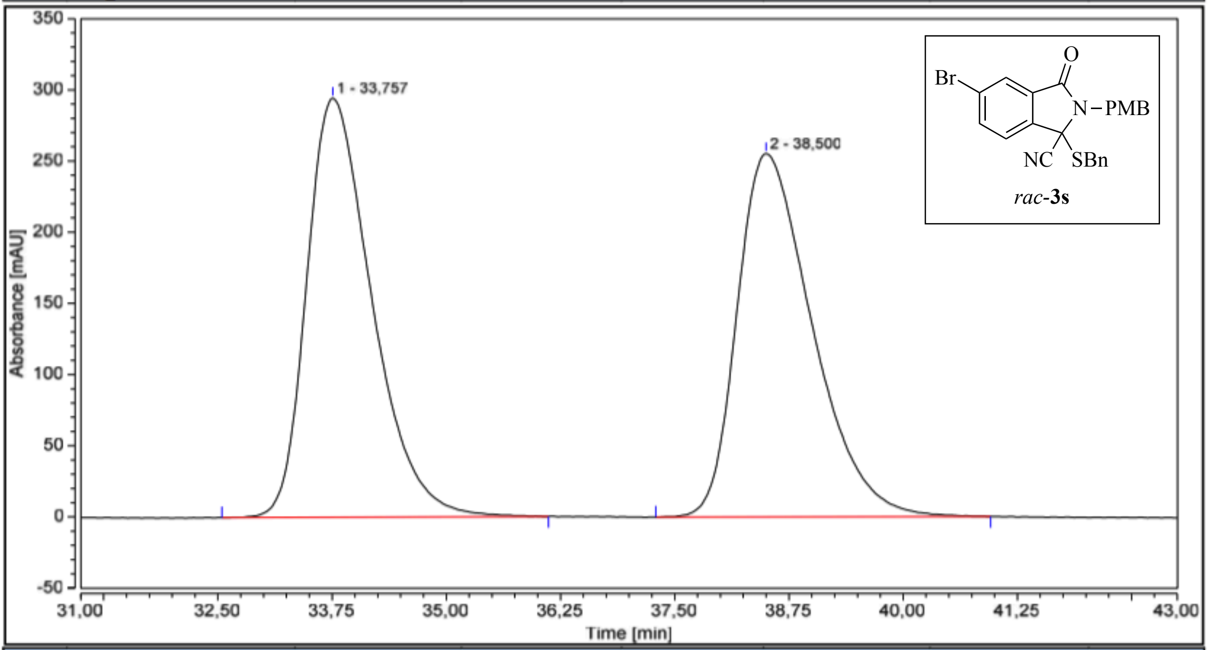

Integration Results

No.	Peak Name	Retention Time<br>min	Area<br>mAU*min	Height<br>mAU	Relative Area<br>%	Relative Height<br>%	Amount<br>n.a.
1		33,757	243,016	294,319	49,94	53,56	n.a.
2		38,500	243,630	255,188	50,06	46,44	n.a.
**Total:**			**486,645**	**549,508**	**100,00**	**100,00**	

Chromatogram

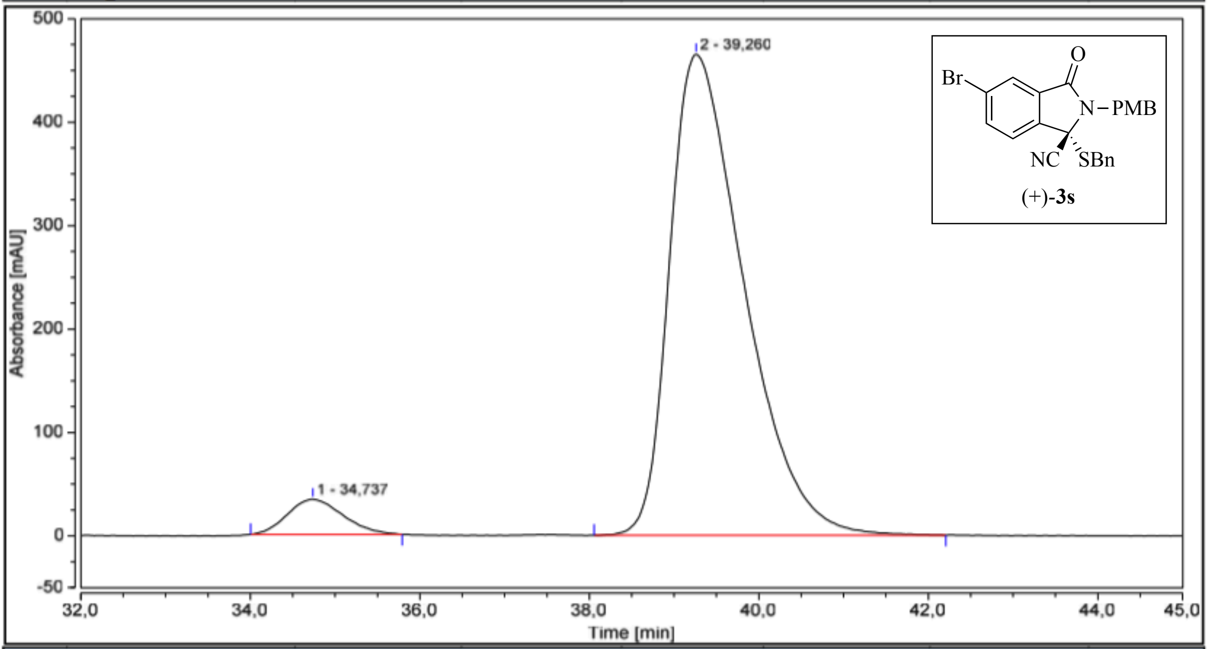

Integration Results

No.	Peak Name	Retention Time<br>min	Area<br>mAU*min	Height<br>mAU	Relative Area<br>%	Relative Height<br>%	Amount<br>n.a.
1		34,737	25,724	33,935	5,17	6,81	n.a.
2		39,260	471,535	464,738	94,83	93,19	n.a.
**Total:**			**497,259**	**498,673**	**100,00**	**100,00**	

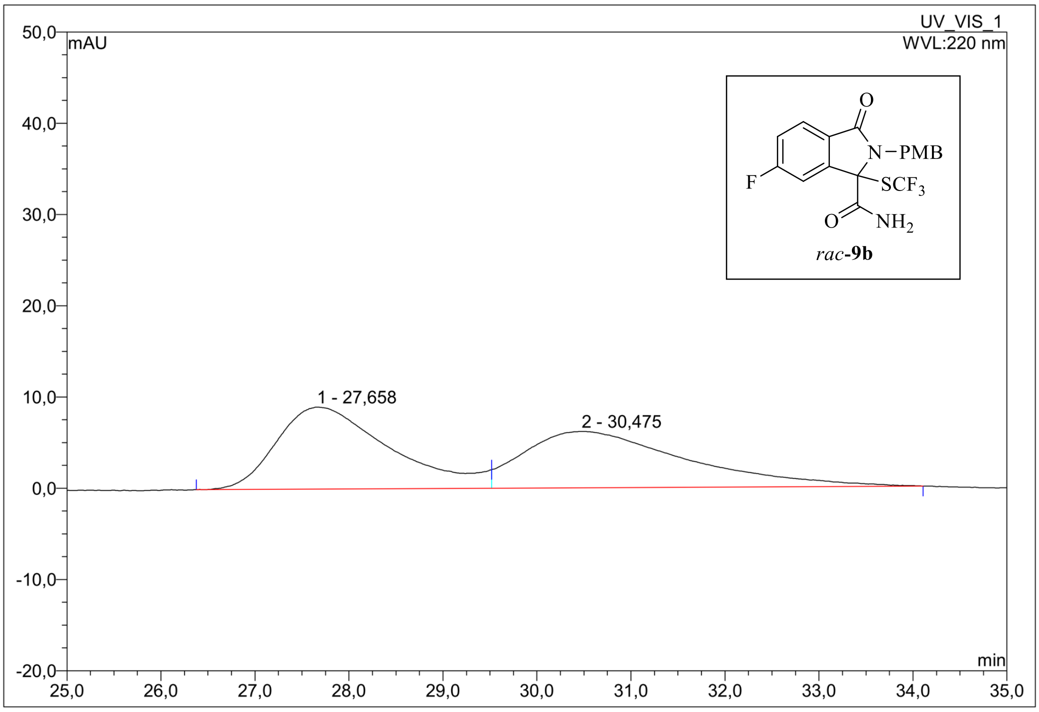

No.	Ret.Time min	Peak Name	Height mAU	Area mAU*min	Rel.Area %	Amount	Type
1	27,66	n.a.	8,990	12,564	50,53	n.a.	BM *
2	30,48	n.a.	6,177	12,299	49,47	n.a.	MB*
Total:			15,167	24,863	100,00	0,000	

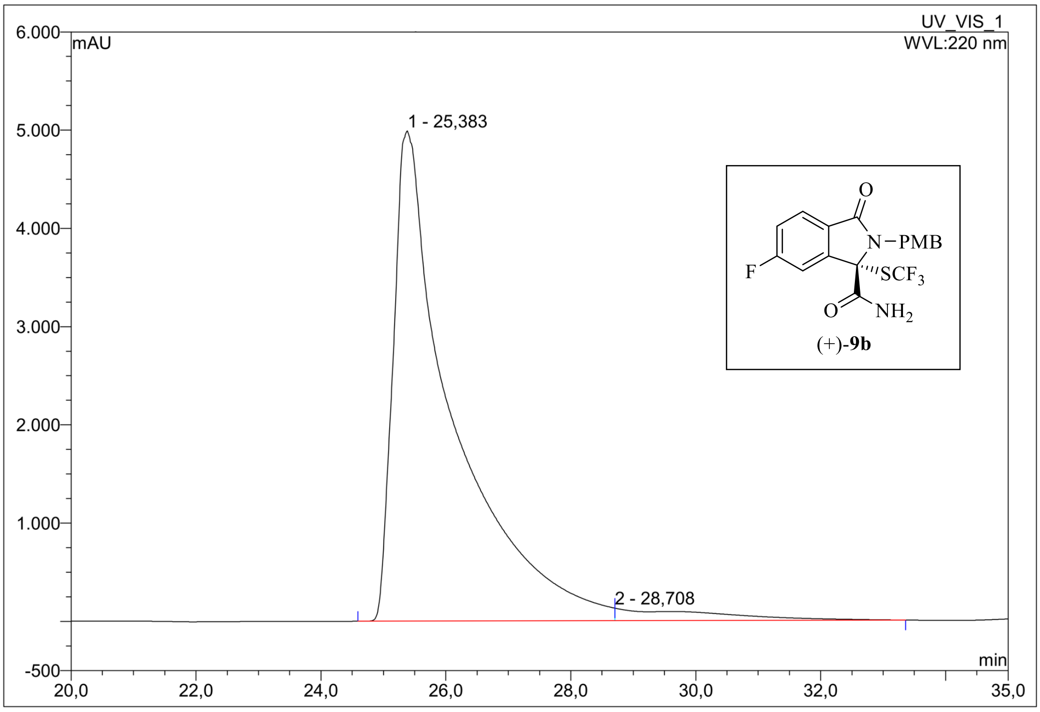

No.	Ret.Time min	Peak Name	Height mAU	Area mAU*min	Rel.Area %	Amount	Type
1	25,38	n.a.	4990,340	5523,679	96,17	n.a.	BM *
2	28,71	n.a.	127,299	220,265	3,83	n.a.	MB*
Total:			5117,639	5743,944	100,00	0,000	

Chromatogram

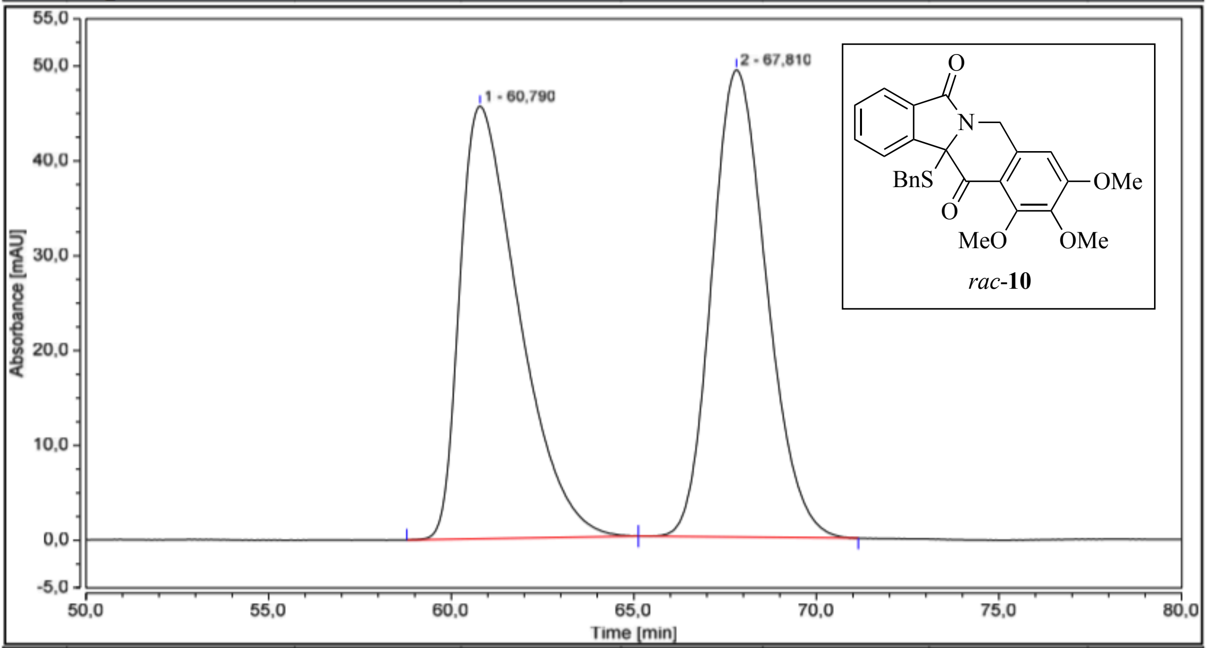

Integration Results

No.	Peak Name	Retention Time min	Area mAU*min	Height mAU	Relative Area %	Relative Height %	Amount n.a.
1		60,790	85,410	45,589	49,87	48,07	n.a.
2		67,810	85,856	49,249	50,13	51,93	n.a.
**Total:**			**171,266**	**94,838**	**100,00**	**100,00**	

Chromatogram

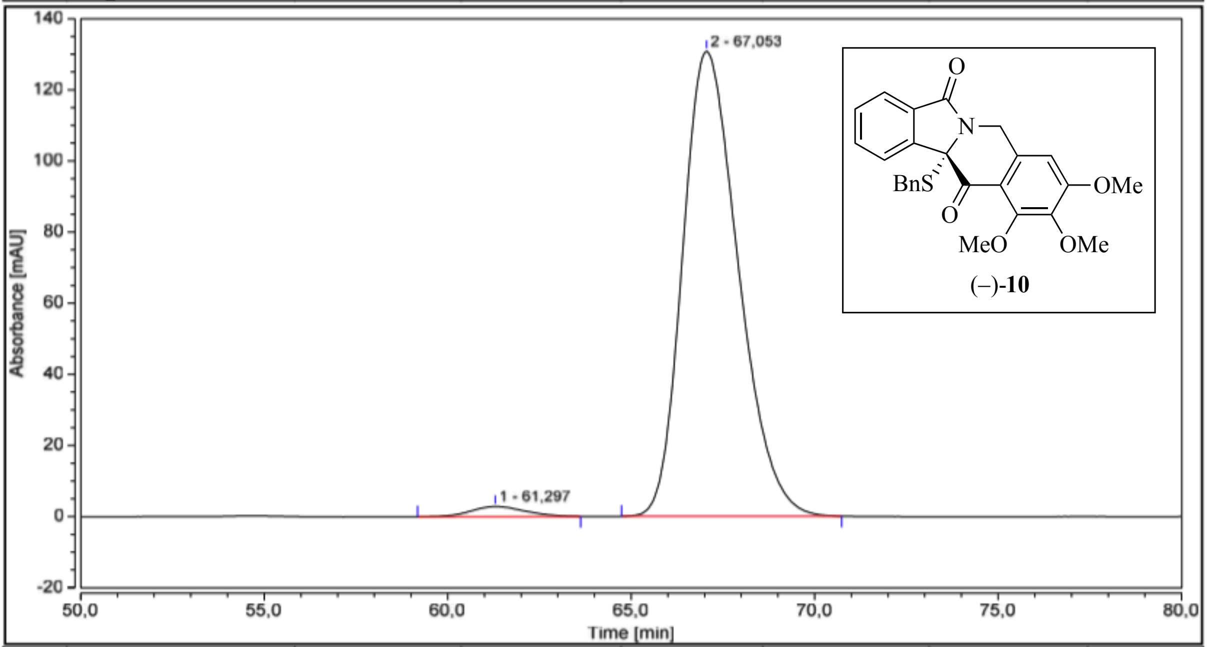

Integration Results

No.	Peak Name	Retention Time min	Area mAU*min	Height mAU	Relative Area %	Relative Height %	Amount n.a.
1		61,297	4,839	2,838	2,06	2,12	n.a.
2		67,053	230,123	130,734	97,94	97,88	n.a.
**Total:**			**234,963**	**133,572**	**100,00**	**100,00**	
